# Supplementary material for: Health education for patients with acute coronary syndrome and type 2 diabetes mellitus: an umbrella review of systematic reviews and meta-analyses
Source: BMJ Open. 2017 Oct 16;7(10):e016857. doi: 10.1136/bmjopen-2017-016857 (PMC5652525; doi:10.1136/bmjopen-2017-016857)
Supplement: Supplementary appendix 1 [file bmjopen-2017-016857supp001.pdf]

Supplementary Annex 1. Searching Strategies

Items	Searching Strategy	Results
	**PubMed**	
#1	(((((((((Acute Coronary Syndrome[MeSH Terms]) OR Myocardial Infarction[MeSH Terms]) OR Angina, Unstable[MeSH Terms]) OR Coronary Artery Disease[MeSH Terms]) OR Coronary Artery Bypass[MeSH Terms]) OR Angina Pectoris[MeSH Terms]) OR "Myocardial Infarct*"[Title/Abstract]) OR "Unstable Angina*"[Title/Abstract]) OR "coronary heart diseas*"[Title/Abstract]) OR "Coronary Artery Disease*"[Title/Abstract]) OR "Unstable Angina Pectori*"[Title/Abstract] Filters: Publication date from 2000/01/01 to 2016/04/31; English	145310
#2	(((((Diabetes Mellitus, Type 2[MeSH Terms]) OR "Type 2 Diabetes Mellitus"[Title/Abstract]) OR "type 2 diabete*"[Title/Abstract]) OR "type ii diabet*"[Title/Abstract]) OR T2DM[Title/Abstract]) OR Adult-Onset Diabetes Mellitus[Title/Abstract] Filters: Publication date from 2000/01/01 to 2016/04/31; English	80218
#3	#1 OR #2	222103
#4	(((((((Health Education[MeSH Terms]) OR Patient Education as Topic[MeSH Terms]) OR Counseling[MeSH Terms]) OR Social Support[MeSH Terms]) OR "Community Health Education*"[Title/Abstract]) OR "Educational Activit*"[Title/Abstract]) OR "Education* intervention*"[Title/Abstract]) OR "Education* program*"[Title/Abstract] Filters: Publication date from 2000/01/01 to 2016/04/31; English	122323
#5	(((Meta-Analysis [Publication Type]) OR Meta-Analysis as Topic[MeSH Terms]) OR "Systematic Revie*"[Title/Abstract]) OR "Meta-analys*"[Title/Abstract] Filters: Publication date from 2000/01/01 to 2016/04/31; English	165265
#6	#3 AND #4 AND #5 Filters: Publication date from 2000/01/01 to 2016/04/31; English	**54**
	**Cochrane Library**	
#1	MeSH descriptor: [Acute Coronary Syndrome] explode all trees	1070
#2	MeSH descriptor: [Myocardial Infarction] explode all trees	9450
#3	MeSH descriptor: [Angina, Unstable] explode all trees	1015
#4	MeSH descriptor: [Coronary Artery Disease] explode all trees	3469

#5	MeSH descriptor: [Coronary Artery Bypass] explode all trees	5321
#6	MeSH descriptor: [Angina Pectoris] explode all trees	4240
#7	"acute coronary syndrome":ti,ab,kw or Unstable Angina*:ti,ab,kw or Myocardial Infarct*:kw or coronary heart diseas*:ti,ab,kw or Coronary Artery Disease*:ti,ab,kw<br>(Word variations have been searched)	29379
#8	#1 or #2 or #3 or #4 or #5 or #6 or #7	34062
#9	MeSH descriptor: [Diabetes Mellitus, Type 2] explode all trees	10474
#10	Type 2 Diabetes Mellitus:ti,ab,kw or type ii diabet*:ti,ab,kw or noninsulin dependent:ti,ab,kw or type 2 diabet*:ti,ab,kw or Adult-Onset Diabetes Mellitus:ti,ab,kw<br>(Word variations have been searched)	20935
#11	#9 or #10	20937
#12	#8 or #11	53777
#13	MeSH descriptor: [Health Education] explode all trees	11291
#14	MeSH descriptor: [Patient Education as Topic] explode all trees	7507
#15	MeSH descriptor: [Counseling] explode all trees	4026
#16	MeSH descriptor: [Rehabilitation Nursing] explode all trees	55
#17	Education*:ti,ab,kw or Community Health Education:ti,ab,kw or Self-Management*:ti,ab,kw or Cardiac Rehabilitation:ti,ab,kw (Word variations have been searched)	40078
#18	#13 or #14 or #15 or #16 or #17	42943
#19	#12 and #18 Publication Year from 2000 to 2016, in Cochrane Reviews (Reviews only) and Other Reviews	120
#20	Cochrane reviews + other reviews	**49 +<br>71=120**
	**Joanna Briggs Institute,_Embase,_MEDLINE**	
#1	Acute Coronary Syndrome.m_titl.	16727
#2	Myocardial Infarction.m_titl.	151398

#3	Unstable Angina.m_titl.	6933
#4	Coronary Artery Disease.m_titl.	54621
#5	Coronary Artery Bypass.m_titl.	30449
#6	Angina Pectoris.m_titl.	17997
#7	Coronary Heart Disease.m_titl.	35397
#8	#1 or #2 or #3 or #4 or #5 or #6	305035
#9	Type 2 Diabetes Mellitus.mp. [mp=ti, ab, ot, nm, hw, kf, px, rx, ui, an, tx, sh, ct, sa, tn, dm, mf, dv, kw]	64926
#10	"type 2 diabet*".m_titl.	102596
#11	"type ii diabet*".m_titl.	6139
#12	Adult-Onset Diabetes Mellitus.mp. [mp=ti, ab, ot, nm, hw, kf, px, rx, ui, an, tx, sh, ct, sa, tn, dm, mf, dv, kw]	307
#13	#9 or #10 or #11 or #12	136982
#14	#8 or #13	439243
#15	Health Education.mp. [mp=ti, ab, ot, nm, hw, kf, px, rx, ui, an, tx, sh, ct, sa, tn, dm, mf, dv, kw]	177285
#16	Patient Education.mp. [mp=ti, ab, ot, nm, hw, kf, px, rx, ui, an, tx, sh, ct, sa, tn, dm, mf, dv, kw]	185631
#17	Counseling.m_titl.	23527
#18	Social Support.m_titl.	11535
#19	"Education*".m_titl.	247901
#20	#15 or #16 or #17 or #18 or #19	577203
#21	#14 and #20	6480
#22	Systematic Review.mp. [mp=ti, ab, ot, nm, hw, kf, px, rx, ui, an, tx, sh, ct, sa, tn, dm, mf, dv, kw]	212014
#23	Meta-Analysis.mp. [mp=ti, ab, ot, nm, hw, kf, px, rx, ui, an, tx, sh, ct, sa, tn, dm, mf, dv, kw]	262009
#24	#22 or #23	381113

#25	#21 and #24	288
#26	limit 25 to English and yr="2000 - 2016"	**263**
	**Cumulative Index to Nursing and Allied Health Literature (CINAHL)**	
#1	TI Acute Coronary Syndrome OR AB Acute Coronary Syndrome	4826
#2	TI Myocardial Infarction OR AB Myocardial Infarction	24578
#3	TI Unstable Angina OR AB Unstable Angina	1353
#4	TI Coronary Artery Disease OR AB Coronary Artery Disease	12930
#5	TI Coronary Heart Disease OR AB Coronary Heart Disease	12321
#6	#1 OR #2 OR #3 OR #4 OR #5	46436
#7	TI Type 2 Diabetes Mellitus OR AB Type 2 Diabetes Mellitus	8952
#8	TI type 2 diabet* OR AB type 2 diabet*	33085
#9	TI Adult-Onset Diabetes Mellitus OR AB Adult-Onset Diabetes Mellitus	23
#10	#7 OR #8 OR #9	33097
#11	#6 OR #10	77923
#12	TI Health Education OR AB Health Education	53415
#13	TI Patient Education OR AB Patient Education	37789
#14	TI Counseling OR AB Counseling	17673
#15	TI Social Support OR AB Social Support	30093
#16	TI Education* OR AB Education*	188291
#17	#12 OR #13 OR #14 OR #15 OR #16	227269
#18	#11 AND #17	4707
#19	TI Systematic Review OR AB Systematic Review	48830

#20	TI Meta-Analysis OR AB Meta-Analysis	28776
#21	#19 OR #20	64611
#22	S18 AND S21 Limiters - Published Date: 20000101-20160431; English Language	**129**
	**Web of Science**	
#1	TS=("Acute Coronary Syndrome*") OR TS=("Myocardial Infarct*") OR TS=("Unstable Angina") OR TS=("Coronary Artery Disease*") OR TS=("Coronary heart disease*")<br>Indexes=SCI-EXPANDED, SSCI, A&HCI, CPCI-S, CPCI-SSH, BKCI-S, BKCI-SSH, ESCI, CCR-EXPANDED, IC Timespan=All years	404699
#2	TS=("Type 2 Diabetes Mellitus") OR TS=("type 2 diabete*") OR TS=("Noninsulin-Dependent Diabete*") OR TS= ("Adult-Onset Diabetes Mellitus")<br>Indexes=SCI-EXPANDED, SSCI, A&HCI, CPCI-S, CPCI-SSH, BKCI-S, BKCI-SSH, ESCI, CCR-EXPANDED, IC Timespan=All years	103548
#3	#2 OR #1<br>Indexes=SCI-EXPANDED, SSCI, A&HCI, CPCI-S, CPCI-SSH, BKCI-S, BKCI-SSH, ESCI, CCR-EXPANDED, IC Timespan=All years	495081
#4	TS=("Health Education") OR TS=("Patient Education")<br>Indexes=SCI-EXPANDED, SSCI, A&HCI, CPCI-S, CPCI-SSH, BKCI-S, BKCI-SSH, ESCI, CCR-EXPANDED, IC Timespan=All years	29830
#5	#4 AND #3<br>Indexes=SCI-EXPANDED, SSCI, A&HCI, CPCI-S, CPCI-SSH, BKCI-S, BKCI-SSH, ESCI, CCR-EXPANDED, IC Timespan=All years	1290
#6	TS=("Systematic Review*") OR TS=("Meta-analysis*")<br>Indexes=SCI-EXPANDED, SSCI, A&HCI, CPCI-S, CPCI-SSH, BKCI-S, BKCI-SSH, ESCI, CCR-EXPANDED, IC Timespan=All years	149663
#7	(#6 AND #5) AND LANGUAGE: (English)<br>Indexes=SCI-EXPANDED, SSCI, A&HCI, CPCI-S, CPCI-SSH, BKCI-S, BKCI-SSH, ESCI, CCR-EXPANDED, IC Timespan=2000-2016	**68**
	**Journals@Ovid**	
#1	Acute Coronary Syndrome.m_titl.	3021
#2	Myocardial Infarction.m_titl.	24269
#3	Unstable Angina.m_titl.	1038
#4	Coronary Artery Disease.m_titl.	10106
#5	Coronary Artery Bypass.m_titl.	6546
#6	Angina Pectoris.m_titl.	1480

#7	Coronary Heart Disease.m_titl.	6487
#8	#1 or #2 or #3 or #4 or #5 or #6	51548
#9	Type 2 Diabetes Mellitus. m_titl.	5097
#10	"type 2 diabet*".m_titl.	26434
#11	"type ii diabet*".m_titl.	980
#12	Adult-Onset Diabetes Mellitus. m_titl.	391
#13	#9 or #10 or #11 or #12	27795
#14	#8 or #13	78792
#15	Health Education. m_titl.	1135
#16	Patient Education. m_titl.	1556
#17	Counseling.m_titl.	5532
#18	Social Support.m_titl.	2667
#19	"Education*".m_titl.	50950
#20	#15 or #16 or #17 or #18 or #19	58969
#21	#14 and #20	510
#22	Systematic Review.mp. [mp=ti, ab, ot, nm, hw, kf, px, rx, ui, an, tx, sh, ct, sa, tn, dm, mf, dv, kw]	117790
#23	Meta-Analysis.mp. [mp=ti, ab, ot, nm, hw, kf, px, rx, ui, an, tx, sh, ct, sa, tn, dm, mf, dv, kw]	215069
#24	#22 or #23	282984
#25	#21 and #24	61
#26	limit 26 to yr="2000 - 2016"	**58**

Supplementary Annex 2.

Citation Matrix: Non-repeated trials (rows) included in the 15 systematic review and/or meta-analyses (column)--ACS.

Author-year (Trial reference)	Barth, Jacob, Daha, & Critchley, 2015	Devi et al., 2015	Ghisi, Abdallah, Grace, Thomas, & Oh, 2014	Kotb, Hsieh, & Wells, 2014	Brown, Clark, Dalal, Welch, & Taylor, 2013	Dickens et al., 2013	Aldcroft, Taylor, Blackstock, & O'Halloran P, 2011	Brown, Clark, Dalal, Welch, & Taylor, 2011	Huttunen-Lenz, Song, & Poland, 2010	Goulding, Furze, & Birks, 2010	Auer, Gaume, Rodondi, Cornuz, & Ghali, 2008	J. Barth, Critchley, & Bengel, 2008	Fernandez et al., 2007	J. Barth, Critchley, & Bengel, 2006	Clark, Hartling, Vandermeer, & McAlister, 2005
Allen JK. Coronary risk factor modification in women after coronary artery bypass surgery. Nursing Research 1996;45(5):260-5.	X											X			
Burt A, Thornley R, Illingworth D, White P, Shaw TR, Turner R. Stopping smoking after myocardial infarction. Lancet 1974;1(7852):304-6.	X											X		X	
Carlsson R, Lindberg G, Westin L, Israelsson B. Influence of coronary nursing managment follow up on lifestyle after acute myocardial infarction. Heart 1997;77(3):256-9.	X											X			
Ockene J, Kristeller JL, Goldberg R, Ockene I, Merriam P, Barrett S, et al. Smoking cessation and severity of disease: the Coronary Artery Smoking Intervention Study. Health Psychology 1992;11(2):119-26.	X											X		X	
DeBusk R, Miller NH, Superko R, Dennis CA, Thomas RJ, et al. A case-management system for coronary risk factor modification after acute myocardial infarction. Annals of Internal Medicine 1994;120(9):721-9.	X										X	X			X
Dornelas EA, Sampson RA, Gray J F, Waters D, Thompson PD. A randomized controlled trial of smoking cessation counseling after myocardial infarction. Preventive Medicine 2000;30(4):261-8.	X								X			X	X	X	
Hajek P, Taylor TZ, Mills P. Brief intervention during hospital admission to help patients to give up smoking after myocardial infarction and bypass surgery: randomised controlled trial. BMJ 2002;324:87-9.	X								X			X	X	X	
Heller RF, Knapp JC, Valenti LA, Dobson AJ. Secondary prevention after acute myocardial infarction. American Journal of Cardiology 1993;72(11):759-62.	X											X	X	X	
Moreno Ortigosa A, Ochoa Gomez FJ, Ramalle-Gomara E, Saralegui Reta I, Fernandez Esteban MV, Quintana Diaz M. Efficacy of an smoking cessationg intervention for patients with myocardial infarction [Eficacia de una intervencion para dejar de fumar en pacientes con infarto de miocardio]. Medicina Clinica 2000;114:209-10.	X											X		X	
Quist-Paulsen P, Gallefoss F. Randomised controlled trial of smoking cessation intervention after admission for coronary heart disease. BMJ 2003;327(7426):1254-7.	X								X			X		X	
Reid R, Pipe A, Higginson LK, D'Angelo MS, Cooke D, Dafoe W. Stepped care approach to smoking cessation in patients hospitalized for coronary artery disease. Journal	X											X		X	

of Cardiopulmonary Rehabilitation 2003;23(3):176-82.															
Rigotti NA, McKool KM, Shiffmann S. Predictors of smoking cessation after coronary artery bypass graft surgery. Annals of Internal Medicine 1994;120(4):287-93.	x								x			x		x	
Sivarajan ES, Newton KM, Almes MJ, Kempf TM, Mansfield LW, Bruce RA. Limited effects of outpatient teaching and counseling after myocardial infarction: a controlled study. Heart & Lung 1983;12(1):65-73.	x													x	
Taylor CB, Houston-Miller N, Killen JD, DeBusk RF. Smoking cessation after acute myocardial infarction: effects of a nurse-managed intervention. Annals of Internal Medicine 1990;113(2):118-23.	x								x				x	x	
Van Elderen T, Maes S, Seegers G. Effects of a group health education program for coronary heart patients after the hospitalization phase. Tijdschrift voor Psychologie & Gezondheid 1991;19(3):129-44.	x											x			
van Elderen-van Kemenade T, Maes S, van den Broek Y. Effects of a health education programme with telephone follow-up during cardiac rehabilitation. British Journal of Clinical Psychology 1994;33(Pt 3):367-78.	x		x	x							x	x		x	
Antypas K, Wangberg SC. An Internet- and mobile-based tailored intervention to enhance maintenance of physical activity after cardiac rehabilitation: short-term results of a randomized controlled trial. Journal of Medical Internet Research 2014;16(3):e77.		x													
Devi R, Powell J, Singh S. A web-based program improves physical activity outcomes in a primary care angina population: randomized controlled trial. Journal of Medical Internet Research 2014;16(9):e186.		x													
Frederix I, Van Driessche N, Hansen D, Berger J, Bonne K, Alders T, et al. Increasing the medium-term clinical benefits of hospital-based cardiac rehabilitation by physical activity telemonitoring in coronary artery disease patients. European Journal of Preventive Cardiology 2015;22(2):150-8.		x													
Lear SA, Singer J, Banner-Lukaris D, Horvat D, Park JE, Bates J, et al. Randomized trial of a virtual cardiac rehabilitation program delivered at a distance via the internet. Circulation: Cardiovascular Quality and Outcomes 2014;7(6):952-9.		x													
Lindsay S, Bellaby P, Smith S, Baker R. Enabling healthy choices: is ICT the highway to health improvement?. Health (London, England: 1997) 2008;12(3):313-31.		x													
Maddison R, Pfaeffli L, Whittaker R, Stewart R, Kerr A, Jiang Y, et al. A mobile phone intervention increases physical activity in people with cardiovascular disease: Results from the HEART randomized controlled trial. European Journal of Preventive Cardiology 2014 May 9 [Epub ahead of print].		x													
Reid RD, Morrin LI, Beaton LJ, Papadakis S, Kocourek J, McDonnell L, et al. Randomized trial of an internet-based computer-tailored expert system for physical activity in patients with heart disease. European Journal of Preventive Cardiology 2012;19(6):1357-64.		x													

Southard BH, Southard DR, Nuckolls J. Clinical trial of an Internet-based case management system for secondary prevention of heart disease. Journal of Cardiopulmonary Rehabilitation and Prevention 2003;23(5):341-8.		X			X	X		X					X		
Varnfield M, Karunanithi M, Lee CK, Honeyman E, Arnold D, Ding H, et al. Smartphone-based home care model improved use of cardiac rehabilitation in postmyocardial infarction patients: results from a randomised controlled trial. Heart 2014;100(22):1770-9.		X													
Vernooij JW, Kaasjager HA, van der Graaf Y, Wierdsma J, Grandjean HM, Hovens MM, et al. Internet based vascular risk factor management for patients with clinically manifest vascular disease: randomised controlled trial. BMJ (Clinical research ed.) 2012;344:e3750.		X													
Zutz A, Ignaszewski A, Bates J, Lear SA. Utilization of the Internet to deliver cardiac rehabilitation at a distance: a pilot study. Telemedicine Journal and e-Health 2007;13(3):323-30.		X													
Dale L, Whittaker R, Jiang Y, Stewart R, Rolleston A, Maddison R. Improving coronary heart disease self-management using mobile technologies (Text4Heart): a randomised controlled trial protocol. Trials 2014;15:71.		X													
ISRCTN29243064. Effectiveness of a comprehensive telerehabilitation program for the heart. http://www.controlled-trials.com/ISRCTN29243064 (accessed January 2015).		X													
NCT02228603. How to enhance physical activity after cardiac rehabilitation? A randomised controlled study comparing two follow-up training exercise programs. https://clinicaltrials.gov/show/NCT02228603 (accessed January 2015).		X													
NCT02350192. Effectiveness of an e-health educational intervention for cardiovascular disease adults in improving total exercise and outcomes. https://clinicaltrials.gov/ct2/show/NCT02350192 (accessed January 2015).		X													
Redfern J, Usherwood T, Harris MF, Rodgers A, Hayman N, Panaretto K, et al. A randomised controlled trial of a consumer-focused e-health strategy for cardiovascular risk management in primary care: the Consumer Navigation of Electronic Cardiovascular Tools (CONNECT) study protocol. BMJ Open 2014; Vol. 4, issue 2. [DOI: 10.1136/bmjopen-2013-004523]		X													
Reinwand D, Kuhlmann T, Wienert J, Vries HD, Lippke S. Designing a theory- and evidence-based tailored eHealth rehabilitation aftercare program in Germany and the Netherlands: study protocol. BMC Public Health 2013;13:1081.		X													
Shah B, Adams M, Peterson E, Powers B, Oddone E, Royal K, et al. Secondary Prevention Risk Interventions Via Telemedicine and Tailored Patient Education (SPRITE): A randomized trial to improve postmyocardial infarction management. Circulation: Cardiovascular Quality and Outcomes 2011;4(2):235-42.		X													

T. Buckley, S. McKinley, R. Gallagher, K. Dracup, D.K. Moser, L.M. Aitken<br>The effect of education and counselling on knowledge, attitudes and beliefs about responses to acute myocardial infarction symptoms<br>Eur J Cardiovasc Nurs, 6 (2007), pp. 105–111			x							x					
J.J. Carlson, G.J. Norman, D.L. Feltz, B.A. Franklin, J.A. Johnson, S.K. Locke<br>Self-efficacy, psychosocial factors, and exercise behavior in traditional versus modified cardiac rehabilitation<br>J Cardpulm Rehabil, 21 (2001), pp. 363–373			x												
S.S. Chan, D.Y. Leung, D.C. Wong, C.P. Lau, V.T. Wong, T.H. Lam<br>A randomized controlled trial of stage-matched intervention for smoking cessation in cardiac-outpatients<br>Addiction, 107 (2012), pp. 829–837			x												
K.M. Cordasco, S.M. Asch, D.S. Bell, J.J. Guterman, S. Gross-Schulman, L. Ramer, et al.<br>A low-literacy medication education tool for safety-net hospital patients<br>Am J Prev Med, 37 (2009), pp. S209–S216			x												
M.H. Eckman, R. Wise, A.C. Leonard, E. Dixon, C. Burrows, F. Khan, et al.<br>Impact of health literacy on outcomes and effectiveness of an educational intervention in patients with chronic diseases<br>Patient Educ Couns, 87 (2012), pp. 143–151			x												
P. Giannuzzi, P.L. Temporelli, R. Marchioli, A.P. Maggioni, G. Balestroni, V. Ceci, et al.<br>Global secondary prevention strategies to limit event recurrence after myocardial infarction: results of the GOSPEL study, a multicenter, randomized controlled trial from the Italian Cardiac Rehabilitation Network<br>Arch Intern Med, 168 (2008), pp. 2194–2204			x												
K.B. Heidal, N.M. Lewis, S.M. Evans, L. Boeckner<br>Nutrition education intervention increases total n − 3 fatty acid intakes in heart patients living in the Midwest<br>Nutr Res, 27 (2007), pp. 33–37			x												
L. Horlick, R. Cameron, W. Firor, U. Bhalerao, R. Baltzan<br>The effects of education and group discussion in the post myocardial infarction patient<br>J Psychosom Res, 28 (1984), pp. 485–492			x												
H.S. Jeong, J.S. Chae, J.S. Moon, Y.S. Yoo<br>An individualized teaching program for atherosclerotic risk factor reduction in patients with myocardial infarction<br>Yonsei Med J, 43 (2002), pp. 93–100			x												
X. Jiang, J.W. Sit, T.K. Wong<br>A nurse-led cardiac rehabilitation programme improves health behaviours and cardiac physiological risk			x												

parameters: evidence from Chengdu, China<br>J Clin Nurs, 16 (2007), pp. 1886–1897															
M. Kummel, T. Vahlberg, A. Ojanlatva, R. Karki, T. Mattila, S.L. Kivela<br>Effects of an intervention on health behaviors of older coronary artery bypass (CAB) patients<br>Arch Gerontol Geriatr, 46 (2008), pp. 227–244			X												
W.S. Leslie, C.R. Hankey, D. Matthews, J.E.P. Currall, M.E.J. Lean<br>A transferable programme of nutritional counselling for rehabilitation following myocardial infarction: a randomised controlled study<br>Eur J Clin Nutr, 58 (2004), pp. 778–786			X												
E. Lidell, B. Fridlund<br>Long-term effects of a comprehensive rehabilitation programme after myocardial infarction<br>Scand J Caring Sci, 10 (1996), pp. 67–74			X							X					
S. Lindsay, S. Smith, P. Bellaby, R. Baker<br>The health impact of an online heart disease support group: a comparison of moderated versus unmoderated support<br>Health Educ Res, 24 (2009), pp. 646–654			X												
R.A. Mayou, D.R. Thompson, A. Clements, C.H. Davies, S.J. Goodwin, K. Normington, et al.<br>Guideline-based early rehabilitation after myocardial infarction: a pragmatic randomised controlled trial<br>J Psychosom Res, 52 (2002), pp. 89–95			X								X				
S. McKinley, K. Dracup, D.K. Moser, B. Riegel, L.V. Doering, H. Meischke, et al.<br>The effect of a short one-on-one nursing intervention on knowledge, attitudes and beliefs related to response to acute coronary syndrome in people with coronary heart disease: a randomized controlled trial<br>Int J Nurs Stud, 46 (2009), pp. 1037–1046			X												
S.M. Moore, M.A. Dolansky<br>Randomized trial of a home recovery intervention following coronary artery bypass surgery<br>Res Nurs Health, 24 (2001), pp. 93–104			X			X									
B. Oldenburg, A. Martin, J. Greenwood, L. Bernstein, R. Allan<br>A controlled trial of a behavioral and educational intervention following coronary artery bypass surgery<br>J Cardpulm Rehabil, 15 (1995), pp. 39–46			X												
J.E. Otterstad<br>Influence on lifestyle measures and five-year coronary risk by a comprehensive lifestyle intervention programme in patients with coronary heart disease<br>Eur J Cardiovasc Prev Rehabil, 10 (2003), pp. 429–437			X												X
P.M. Smith, E. Burgess			X						X						

Smoking cessation initiated during hospital stay for patients with coronary artery disease: a randomized controlled trial<br>CMAJ, 180 (2009), pp. 1297–1303															
T.M. Van Elderen, S. Maes, G. Seegers, H. Kragten, L. Relik-Can Wely<br>Effects of a post-hospitalization group health education programme for patients with coronary heart disease<br>Psychol Health, 9 (1994), pp. 317–330			X											X	
J. Wolkanin-Bartnik, H. Pogorzelska, A. Bartnik<br>Patient education and quality of home-based rehabilitation in patients older than 60 years after acute myocardial infarction<br>J Cardiopulm Rehabil Prev, 31 (2011), pp. 249–253			X												
C. Froger-Bompas, B. Laviolle, P. Guillo, C. Letellier, K. Ligier, J.C. Daubert, et al.<br>Sustained positive impact of a coronary rehabilitation programme on adherence to dietary recommendations<br>Arch Cardiovasc Dis, 102 (2009), pp. 97–104			X												
B.J. Linde, N.M. Janz<br>Effect of a teaching program on knowledge and compliance of cardiac patients<br>Nurs Res, 28 (1979), pp. 282–286			X												
J. Oliveira, F. Ribeiro, H. Gomes<br>Effects of a home-based cardiac rehabilitation program on the physical activity levels of patients with coronary artery disease<br>J Cardiopulm Rehabil Prev, 28 (2008), pp. 392–396			X												
R. Song, H. Lee<br>Managing health habits for myocardial infarction (MI) patients<br>Int J Nurs Stud, 38 (2001), pp. 375–380			X												
B.L. Verges, B. Patois-Verges, M. Cohen, J.M. Casillas<br>Comprehensive cardiac rehabilitation improves the control of dyslipidemia in secondary prevention<br>J Cardpulm Rehabil, 18 (1998), pp. 408–415			X												
T. Yoshida, M. Kohzuki, K. Yoshida, M. Hiwatari, M. Kamimoto, C. Yamamoto, et al.<br>Physical and psychological improvements after phase II cardiac rehabilitation in patients with myocardial infarction<br>Nurs Health Sci, 1 (1999), pp. 163–170			X												
G.F. Fletcher<br>Rehabilitation after coronary angioplasty—is it effective<br>Arch Phys Med Rehabil, 67 (1986), pp. 517–519			X												
C.J. Frame, C.G. Green, D.G. Herr, M.L. Taylor<br>A 2-year stage of change evaluation of dietary fat and fruit and vegetable intake behaviors of cardiac rehabilitation patients			X												

Am J Health Promot, 17 (2003), pp. 361–368															
S. Pelletier, S. Kundrat, C.M. Hasler<br>Effects of a functional foods nutrition education program with cardiac rehabilitation patients<br>J Cardpulm Rehabil, 23 (2003), pp. 334–340			X												
L. Jensen<br>Self-administered cardiac medication program evaluation<br>Can J Cardiovasc Nurs, 13 (2003), pp. 35–44			X												
J. Lisspers, C. Hofman-Bang, R. Nordlander, L. Ryden, O. Sundin, A. Ohman, et al.<br>Multifactorial evaluation of a program for lifestyle behavior change in rehabilitation and secondary prevention of coronary artery disease<br>Scand Cardiovasc J, 33 (1999), pp. 9–16			X												
A. Palomaki, S. Miilunpalo, P. Holm, E. Makinen, K. Malminiem<br>Effects of preventive group education on the resistance of LDL against oxidation and risk factors for coronary heart disease in bypass surgery patients<br>Ann Med, 34 (2002), pp. 272–283			X												
M.T. Timlin, K.V. Shores, M. Reicks<br>Behavior change outcomes in an outpatient cardiac rehabilitation program<br>J Am Diet Assoc, 102 (2002), pp. 664–671			X												
C. Bellman, K. Hambraeus, J. Lindbäck, B. Lindahl<br>Achievement of secondary preventive goals after acute myocardial infarction: a comparison between participants and nonparticipants in a routine patient education program in Sweden<br>J Cardiovasc Nurs, 24 (2009), pp. 362–368			X												
K. Dracup, A.I. Meleis, S. Clark, A. Clyburn, L. Shields, M. Staley<br>Group counseling in cardiac rehabilitation: effect on patient compliance<br>Patient Educ Couns, 6 (1984), pp. 169–177			X				X								
Z. Irmak, H. Fesci<br>Effects of nurse-managed secondary prevention program on lifestyle and risk factors of patients who had experienced myocardial infarction<br>Appl Nurs Res, 23 (2010), pp. 147–152			X												
J. Marshall, S. Penckofer, J. Llewellyn<br>Structured postoperative teaching and knowledge and compliance of patients who had coronary artery bypass surgery<br>Heart Lung, 15 (1986), pp. 76–82			X												
T. Van Elderen, E. Dusseldorp<br>Lifestyle effects of group health education for patients with coronary heart disease			X												

Psychol Health, 16 (2001), pp. 327–341															
P. Davidson, M. Digiacomo, R. Zecchin, M. Clarke, G. Paul, K. Lamb, et al.<br>A cardiac rehabilitation program to improve psychosocial outcomes of women with heart disease<br>J Womens Health, 17 (2008), pp. 123–134			X												
Arthur HM, Smith KM, Kodis J, McKelvie R (2002) A controlled trial of hospital versus home-based exercise in cardiac patients. Medicine and Science in Sports and Exercise. 34(10): 15441550				X											
Bambauer KZ, Aupont O, Stone PH, Locke SE, Mullan MG, et al. (2005) "The effect of a telephone counseling intervention on self-rated health of cardiac patients.".<br>Psychosomatic Medicine 67(4): 539–545.				X											
Bazargani RH, Besharat MA, Ehsan HB, Nejatian M, Hosseini K (2011) The efficacy of Chronic Disease Self Management Programs and Tele-health on psychosocial adjustment by increasing self-efficacy in patients with CABG. Procedia - Social and Behavioral Sciences 30: 817–821.				X											
Beckie T (1989) A supportive-educative telephone program: Impact on knowledge and anxiety after coronary artery bypass graft surgery. Heart and Lung: Journal of Critical Care 18(1): 46–55.				X											
Gallagher R, McKinley S, Dracup K (2003) Effects of a telephone counseling intervention on psychosocial adjustment in women following a cardiac event. Heart and Lung: Journal of Acute and Critical Care 32(2): 79–87.				X		X									
Hanssen TA, Nordrehaug JE, Eide GE, Rokne B (2007) Improving outcomes after myocardial infarction: A randomized controlled trial evaluating effects of a telephone follow-up intervention. European Journal of Cardiovascular Prevention and Rehabilitation 14 (3): 429–437.				X				X							
Hanssen TA, Nordrehaug JE, Eide GE, Hanestad BR (2009) Does a telephone follow-up intervention for patients discharged with acute myocardial infarction have long-term effects on health-related quality of life? A randomised controlled trial. Journal of Clinical Nursing 18(9): 1334–1345.				X											
Hartford K, Wong C, Zakaria D (2002) Randomized controlled trial of a telephone intervention by nurses to provide information and support to patients and their partners after elective coronary artery bypass graft surgery: Effects of anxiety. Heart and Lung: Journal of Acute and Critical Care, 31(3), 199–206.				X											
Holmes-Rovner M, Stommel M, Corser WD, Olomu A, Holtrop JS, et al. (2008) Does outpatient telephone coaching add to hospital quality improvement following				X		X									

hospitalization for acute coronary syndrome? Journal of General Internal Medicine 23(9): 1464–70.															
Ma Y, Ockene IS, Rosal MC, Merriam PA, Ockene JK, et al. (2010) Randomized trial of a pharmacist-delivered intervention for improving lipid-lowering medication adherence among patients with coronary heart disease. Cholesterol 2010: 1–11.				X											
McLaughlin TJ, Aupont O, Bambauer K, Stone P, Mullan MG, et al. (2005) Improving psychologic adjustment to chronic illness in cardiac patients: The role of depression and anxiety. Journal of General Internal Medicine 20(12): 1084–1090.				X											
Mittag O, China C, Hoberg E, Juers E, Kolenda K, et al. (2006) Outcomes of cardiac rehabilitation with versus without a follow-up intervention rendered by telephone (Luebeck follow-up trial): overall and gender-specific effects. International journal of rehabilitation research Internationale Zeitschrift fur Rehabilitationsforschung. Revue internationale de recherches de readaptation 29(4): 295–302.				X		X									
Neubeck L, Redfern J, Briffa T, Ascanio R, Freedman SB, et al. (2009) Cardiovascular risk benefits of the CHOICE (Choice of Health Options In prevention of Cardiovascular Events) program are maintained for four years: Randomised controlled trial. European Heart Journal Conference: European Society of Cardiology, ESC Congress 2009 Barcelona Spain. Conference Start: 20090829 Conference End: 20090902. Conference Publication: (var.pagings) 30: 76.				X											
Neubeck L, Freedman SB, Briffa T, Bauman A, Redfern J (2011) Four-year follow-up of the choice of health options in prevention of cardiovascular events randomized controlled trial. European Journal of Cardiovascular Prevention and Rehabilitation.18(2)278286				X											
Redfern J, Briffa T, Ellis E, Freedman SB (2008) Patient-centered modular secondary prevention following acute coronary syndrome: A randomized controlled trial. Journal of Cardiopulmonary Rehabilitation and Prevention.28(2)107117				X											
Redfern J, Briffa T, Ellis E, Freedman SB (2009) Choice of secondary prevention improves risk factors after acute coronary syndrome: 1-Year follow-up of the CHOICE (Choice of Health Options in prevention of Cardiovascular Events) randomised controlled trial. Heart 95 (6): 468–475.				X											
Redfern J, Menzies M, Briffa T, Freedman SB (2010) Impact of medical consultation frequency on modifiable risk factors and medications at 12 months after acute coronary syndrome in the CHOICE randomised controlled trial. International Journal of Cardiology 145 (3): 481–486.				X											
Reid RD, Pipe AL, Quinlan B, Oda J (2007) Interactive				X					X						

voice response telephony to promote smoking cessation in patients with heart disease: A pilot study. Patient Education and Counseling 66 (3): 319–326.															
Smith KM, Arthur HM, McKelvie RS, Kodis J (2004) Differences in sustainability of exercise and health-related quality of life outcomes following home or hospital-based cardiac rehabilitation. European Journal of Cardiovascular Prevention and Rehabilitation 11(4): 313–319.				X											
Smith KM (2007) Sustainability of exercise capacity and quality of life after home or hospital based exercise training in low-risk patients following coronary artery bypass graft surgery: a six-year follow-up of a randomized controlled trial, McMaster University (Canada): 176 p–176 p.				X											
Smith KM, McKelvie RS, Thorpe KE, Arthur HM (2011) Six-year follow-up of a randomised controlled trial examining hospital versus home-based exercise training after coronary artery bypass graft surgery. Heart 97(14): 1169–1174.				X											
Stevens B (1985) The effectiveness of patient education follow-up by telephone on knowledge of post-myocardial infarction patients. (M.Nus., University of Alberta (Canada)). ProQuest Dissertations and Theses (303461144).				X											
Tranmer JE, Parry MJE (2004) Enhancing postoperative recovery of cardiac surgery patients - A randomized clinical trial of an advanced practice nursing intervention. Western Journal of Nursing Research 26(5): 515–532.				X											
Vale MJ, Jelinek MV, Best JD, Dart AM, Grigg LE, et al. (2003) Coaching patients on achieving cardiovascular health (COACH): A multicenter randomized trial in patients with coronary heart disease. Archives of Internal Medicine–. 163(22)27752783				X		X							X		X
Vale MJ, Jelinek MV, Best JD, Santamaria JD (2002) Coaching patients with coronary heart disease to achieve the target cholesterol: A method to bridge the gap between evidence-based medicine and the "real world" - randomized controlled trial. Journal of Clinical Epidemiology. 55(3)245252				X											X
Clark NM, Janz NK, Becker MH, . Impact of self-management education on the functional health status of older adults with heart disease. The Gerontologist 1992; 32: 438–443.					X			X							
Clark NM, Janz NK, Dodge JA, . Self-Management of heart disease by older adults. Res Aging 1997; 19: 362–382.					X										
Clark NM, Janz NK, Dodge JA, . Changes in functional health status of older women with heart disease: evaluation of a program based on self-regulation. J					X			X							

Gerontol B Psychol Sci Soc Sci 2000; 55B: S117–S126.															
Wheeler JR. Can a disease self-management programme reduce health care costs: the case of older women with heart disease. Med Care 2003; 41: 706–715					X										
Clark NM, Janz NK, Dodge JA, . Heart disease management by women: does intervention format matter? Health Educ Behav 2009; 36: 394–409					X			X							
Cupples ME, McKnight A, O'Neill C, . The effect of personal health education on the quality of life of patients with angina in general practice. Health Educ J 1996; 55: 75–83					X										
Cupples ME, McKnight A. Five year follow up of patients at high cardiovascular risk who took part in randomised controlled trial of health promotion. Brit Med J 1999; 319: 687–688.					X			X							X
Cupples ME, McKnight A. Randomised controlled trial of health promotion in general practice for patients at high cardiovascular risk. Brit Med J 1994; 309: 993–996					X										X
O'Neill C, Normand C, Cupples M, . Cost effectiveness of personal health education in primary care for people with angina in the greater Belfast area of Northern Ireland. J Epidemiol Community Health 1996; 50: 538–540.					X										
Esposito D, Brown R, Chen A, . Impacts of a disease management program for dually eligible beneficiaries. Health Care Finan Rev 2008; 30: 27–45.					X			X							
Hanssen TA, Nordrehaug JE, Eide BE, . Improving outcomes after myocardial infarction: a randomized controlled trial evaluating effects of a telephone follow-up intervention. Eur J Cardiovasc Prevent Rehab 2007; 14(3): 429–437.					X										
Hanssen TA, Nordrehaug JE, Eide GE, . Can telephone follow-up after discharge improve life style factors after a myocardial infarction? A randomized controlled trial. 7th Annual Cardiovascular Nursing Spring Meeting of the European Society of Cardiology Council on Cardiovascular Nursing and Allied Professions: changing practice to improve care Manchester, UK 23–24 March 2007. Eur J Cardiovasc Nurs 2007; 6: S43–S44.					X										
Hanssen TA, Nordrehaug JE, Eide GE, . Does a telephone follow-up intervention for patients discharges with acute myocardial infarction have long-term effects on health-related quality of life? A randomised controlled trial. J Clin Nurs 2009; 18: 1334–1345					X										
Hanssen TA, Nordrehaug JE, Eide GE, . Evaluating the effect of a combined reactive and proactive telephone follow-up intervention after acute myocardial infarction. A randomized controlled trial. Eur J Cardiovasc Nurs 2006;					X										

5: S46–S46.															
Lie I, Arnesen H, Sandvik L, . Health-related quality of life after coronary artery bypass grafting. The impact of a randomised controlled home-based intervention program. Qual Life Res 2009; 18: 201–207.					X			X							
Hofman-Bang C, Lisspers J, Nordlander R, . Two-year results of a controlled study of residential rehabilitation for patients treated with percutaneous transluminal coronary angioplasty. A randomized study of a multifactorial programme. Eur Heart J 1999; 20: 1465–1474.					X	X		X							
Lisspers J, Sundin O, Hofman-Bang C, . Behavioural effects of a comprehensive, mutifactorial program for lifestyle change after percutaneous transluminal coronary angioplasty: a prospective, randomized, controlled study. J Psychosom Res 1999; 46: 143–154.					X										
Lisspers J, Sundin O, Ohman A, . Long-term effects of lifestyle behavior change in coronary artery disease: effects on recurrent coronary events after percutaneous coronary intervention. Health Psychol 2005; 24: 41–48.					X										
P.RE.COR. Group. Comparison of a rehabilitation programme, a counselling programme and usual care after an acute myocardial infarction: results of a long-term randomized trial. Eur Heart J 1991; 12: 612–616.					X			X							X
Brown R, Peikes D, Chen A, . 15-Site randomized trial of coordinated care in medicare FFS. Health Care Finan Rev 2008; 30: 5–25					X			X							
Peikes D, Chen A, Schore J, . Effects of care coordination on hospitalization, quality of care, and health care expenditures among medicare beneficiaries 15 randomized trials. J Amer Med Assoc 2009; 301: 603–618.					X										
Pogosova GV, Kalinina AM, Spivak EI, . Efficacy of an educational preventive technology in patients with stable angina in ambulatory conditions. Kardiologiia 2008; 48(7): 4–9.					X			X							
Tingstrom PR, Kamwendo K, Bergdahl B. Effects of a problem-based learning rehabilitation programme on quality of life in patients with coronary artery disease. Eur J Cardiovasc Nurs 2005; 4: 324–330					X			X							
Appels A, B„r F, van der PG, et al. Effects of treating exhaustion in angioplasty patients on new coronary events: results of the randomized Exhaustion Intervention Trial (EXIT). Psychosomatic Medicine. 2005;67(2):217-223.						X									
Ashton RC, Whitworth GC, Seldomridge JA, et al. The effects of self-hypnosis on quality of life following coronary artery bypass surgery: preliminary results of a prospective, randomized trial. Journal of Alternative and						X									

Complementary Medicine 1995;1(3):285-290.															
Barr Taylor CB, Houston-Miller N, Ahn DK, Haskell W, DeBusk RF. The effects of exercise training programs on psychosocial improvement in uncomplicated postmyocardial infarction patients. Journal of Psychosomatic Research. 1986;30(5):581-587.						x									
Berkman LF, Blumenthal J, Burg M, et al. Effects of treating depression and low perceived social support on clinical events after myocardial infarction: the Enhancing Recovery in Coronary Heart Disease Patients (ENRICHD) Randomized Trial. JAMA : the journal of the American Medical Association. 2003;289(23):3106-3116.						x									
Blumenthal JA, Sherwood A, Babyak MA, et al. Effects of exercise and stress management training on markers of cardiovascular risk in patients with ischemic heart disease: a randomized controlled trial. JAMA : the journal of the American Medical Association. 2005;293(13):1626-1634.						x									
Brown MA, Munford AM, Munford PR. Behavior therapy of psychological distress in patients after myocardial infarction or coronary bypass. Journal of Cardiopulmonary Rehabilitation. 1993;13(3):201-210.						x									
Burgess AW, Lerner DJ, D'Agostino RB, Vokonas PS, Hartman CR, Gaccione P. A randomized control trial of cardiac rehabilitation. Social Science & Medicine. 1987;24(4):359-370.						x									
Claesson M, Birgander LS, Lindahl B, et al. Women's hearts-stress management for women with ischemic heart disease: explanatory analyses of a randomized controlled trial. Journal of Cardiopulmonary Rehabilitation. 2005;25(2):93-102.						x									
de Klerk JE, du Plessis WF, Steyn HS, Botha M. Hypnotherapeutic ego strengthening with male South African coronary artery bypass patients. The American Journal of Clinical Hypnosis. 2004;47(2):79-92.						x									
Dugmore LD, Tipson RJ, Phillips MH, et al. Changes in cardiorespiratory fitness, psychological wellbeing, quality of life, and vocational status following a 12 month cardiac exercise rehabilitation programme. Heart. 1999;81(4):359-366						x									x
Engblom E, Korpilahti K, H„m„l„inen H, R”nnemaa T, Puukka P. Quality of life and return to work 5 years after coronary artery bypass surgery. Long-term results of cardiac rehabilitation. Journal of Cardiopulmonary Rehabilitation. 1997;17(1):29-36.						x									x
Frasure-Smith N, Lesperance F, Prince RH, et al. Randomised trial of home-based psychosocial nursing intervention for patients recovering from myocardial infarction. Lancet. 1997;350(9076):473-479.						x									x
Freedland KE, Skala JA, Carney RM, et al. Treatment of depression after coronary artery bypass surgery: A randomized controlled trial. Archives of General Psychiatry. 2009;66(4):387-396.						x									

Fridlund B, H”gstedt B, Lidell E, Larsson PA. Recovery after myocardial infarction. Effects of a caring rehabilitation programme. Scandinavian Journal of Caring Sciences. 1991;5(1):23-32.						X									X
Frizelle DJ, Lewin RJ, Kaye G, et al. Cognitive-behavioural rehabilitation programme for patients with an implanted cardioverter defibrillator: a pilot study. British Journal of Health Psychology. 2004;9(3):381-392.						X									
Furze G, Dumville JC, Miles JNV, Irvine K, Thompson DR, Lewin RJP. "Prehabilitation" prior to CABG surgery improves physical functioning and depression. International Journal of Cardiology. 2009;132:51-58.						X									
Goodman H, Parsons A, Davison J, et al. A randomised controlled trial to evaluate a nurse-led programme of support and lifestyle management for patients awaiting cardiac surgery 'Fit for surgery: Fit for life' study. European Journal of Cardiovascular Nursing. 2008;7(3):189-195.						X									
Hughes AR, Mutrie N, Macintyre PD. Effect of an exercise consultation on maintenance of physical activity after completion of phase III exercise-based cardiac rehabilitation. European Journal of Cardiovascular Prevention and Rehabilitation 2007;14(1):114-121.						X									
Johnston M, Foulkes J, Johnston DW, Pollard B, Gudmundsdottir H. Impact on patients and partners of inpatient and extended cardiac counseling and rehabilitation: a controlled trial. Psychosomatic Medicine. 1999;61(2):225-233.						X					X				X
Jones DA, West RR. Psychological rehabilitation after myocardial infarction: multicentre randomised controlled trial. BMJ (Clinical research ed.). 1996;313(7071):1517-1521.						X									X
Karlsson MR, Edstr”m-Ploss C, Held C, Henriksson P, Billing E, Wall,n NH. Effects of expanded cardiac rehabilitation on psychosocial status in coronary artery disease with focus on type D characteristics. Journal of Behavioral Medicine. 2007;30(3):253-261.						X									
Koertge J, Janszky I, Sundin O, et al. Effects of a stress management program on vital exhaustion and depression in women with coronary heart disease: a randomized controlled intervention study. Journal of Internal Medicine. 2008;263(3):281-293.						X									
Leserman J, Stuart EM, Mamish ME, Benson H. The efficacy of the relaxation response in preparing for cardiac surgery. Behavioral Medicine. 1989;15(3):111-117.						X									
Lesperance F, Frasure-Smith N, Koszycki D, et al. Effects of citalopram and interpersonal psychotherapy on depression in patients with coronary artery disease: the Canadian Cardiac Randomized Evaluation of Antidepressant and Psychotherapy Efficacy (CREATE) trial. JAMA : the journal of the American Medical Association. 2007;297(4):367-379.						X									
Lewin B, Robertson IH, Cay EL, Irving JB, Campbell M.						X									

Effects of self-help post-myocardial-infarction rehabilitation on psychological adjustment and use of health services. Lancet. 1992;339(8800):1036-1040.															
Lewin RJ, Coulton S, Frizelle DJ, Kaye G, Cox H. A brief cognitive behavioural preimplantation and rehabilitation programme for patients receiving an implantable cardioverter-defibrillator improves physical health and reduces psychological morbidity and unplanned readmissions. Heart. 2009;95:63-69.						X									
Lewin RJ, Furze G, Robinson J, et al. A randomised controlled trial of a self-management plan for patients with newly diagnosed angina. The British Journal of General Practice 2002;52(476):194-201.						X				X					
Lewin RJ, Thompson DR, Elton RA. Trial of the effects of an advice and relaxation tape given within the first 24 h of admission to hospital with acute myocardial infarction. International Journal of Cardiology. 2002;82(2):107-114.						X					X		X		
Lie I, Arnesen H, Sandvik L, Hamilton G, Bunch EH. Effects of a home-based intervention program on anxiety and depression 6 months after coronary artery bypass grafting: a randomized controlled trial. Journal of Psychosomatic Research. 2007;62(4):411-418.						X									
Linden B. Evaluation of a home-based rehabilitation programme for patients recovering from acute myocardial infarction. Intensive & Critical Care Nursing 1995;11(1):10-19.						X					X				
McHugh F, Lindsay GM, Hanlon P, et al. Nurse led shared care for patients on the waiting list for coronary artery bypass surgery: a randomised controlled trial. Heart 2001;86(3):317-323.						X									X
McLaughlin TJ, Aupont O, Bambauer KZ, et al. Improving psychologic adjustment to chronic illness in cardiac patients. The role of depression and anxiety. Journal of General Internal Medicine 2005;20(12):1084-1090.						X									
Michalsen A, Grossman P, Lehmann N, et al. Psychological and quality-of-life outcomes from a comprehensive stress reduction and lifestyle program in patients with coronary artery disease: results of a randomized trial. Psychotherapy and Psychosomatics. 2005;74(6):344-352.						X									
Murchie P, Campbell NC, Ritchie LD, Deans HG, Thain J. Effects of secondary prevention clinics on health status in patients with coronary heart disease: 4 year follow-up of a randomized trial in primary care. Family Practice. 2004;21(5):567-574.						X									X
Newton M, Mutrie N, McArthur JD. The effects of exercise in a coronary rehabilitation programme. Scottish Medical Journal. 1991;36(2):38-41.						X									
Oldridge N, Guyatt G, Jones N, et al. Effects on quality of life with comprehensive rehabilitation after acute myocardial infarction. The American Journal of Cardiology. 1991;67(13):1084-1089.						X									X
Paul-Labrador M, Polk D, Dwyer JH, et al. Effects of a randomized controlled trial of transcendental meditation						X									

on components of the metabolic syndrome in subjects with coronary heart disease. Archives of Internal Medicine. 2006;166(11):1218-1224.															
Postlethwaite R, Stirling G, Peck CL. Stress inoculation for acute pain: a clinical trial. Journal of Behavioral Medicine. 1986;9(2):219-227.						X									
Scholz U, Knoll N, Sniehotta FF, Schwarzer R. Physical activity and depressive symptoms in cardiac rehabilitation: long-term effects of a self-management intervention. Social Science & Medicine 2006;62(12):3109-3120.						X									
Schrader G, Cheok F, Hordacre AL, Marker J, Wade V. Effect of psychiatry liaison with general practitioners on depression severity in recently hospitalised cardiac patients: a randomised controlled trial. The Medical Journal of Australia. 2005;182(6):272-276.						X									
Sebregts EH, Falger PR, Appels A, Kester AD, B„r FW. Psychological effects of a short behavior modification program in patients with acute myocardial infarction or coronary artery bypass grafting. A randomized controlled trial. Journal of Psychosomatic Research. 2005;58(5):417-424.						X									
Seki E, Watanabe Y, Sunayama S, et al. Effects of phase III cardiac rehabilitation programs on health-related quality of life in elderly patients with coronary artery disease: Juntendo Cardiac Rehabilitation Program (J-CARP). Circulation Journal 2003;67(1):73-77.						X									X
Shuldham CM, Fleming S, Goodman H. The impact of pre-operative education on recovery following coronary artery bypass surgery. A randomized controlled clinical trial. European Heart Journal. 2002;23(8):666-674.						X									
Sneed NV, Finch NJ, Michel Y. The effect of psychosocial nursing intervention on the mood state of patients with implantable cardioverter defibrillators and their caregivers. Progress In Cardiovascular Nursing. 1997;12(2):4-14.						X									
Sorlie T, Busund R, Sexton J, Sexton H, Sorlie D. Video information combined with individualized information sessions: Effects upon emotional well-being following coronary artery bypass surgery-A randomized trial. Patient Education & Counseling. 2007;65(2):180-188.						X									
Stern MJ, Gorman PA, Kaslow L. The group counseling v exercise therapy study. A controlled intervention with subjects following myocardial infarction. Archives of Internal Medicine. 1983;143(9):1719-1725.						X									X
Thompson DR, Meddis R. A prospective evaluation of in hospital counselling for first time myocardial infarction men. Journal of Psychosomatic Research. 1990;34(3):237-248.						X									
Trzcieniecka-Green A, Steptoe A. The effects of stress management on the quality of life of patients following acute myocardial infarction or coronary bypass surgery. European Heart Journal. 1996;17(11):1663-1670.						X									
Unden AL, Schenck-Gustafsson K, Axelsson PO, Karlsson I, Orth-Gom,r K, Ydrefors AM. Positive effects of						X									

increased nurse support for male patients after acute myocardial infarction. Quality of Life Research 1993;2(2):121-127.															
Barth J. Depression as a Risk Factor for Mortality in Patients With Coronary Heart Disease: A Meta-analysis. 2004.						x									
Merswolken M. Treating anxiety in patients with coronary heart disease: a randomized controlled trial. 2011.						x									
utwalli HA. Randomized controlled evaluation shows the effectiveness of a home-based cardiac rehabilitation program. 2012.						x									
Oranta O. The outcomes of interpersonal counselling on depressive symptoms and distress after myocardial infarction. 2010.						x									
Poortaghi S. General health improves with home-based cardiac rehabilitation program. 2011.						x									
Utriyaprasit K. Recovery after coronary artery bypass surgery: effect of an audiotape information programme. 2010.						x									
Izawa KP, Watanabe S, Omiya K, et al.. Effect of the self-monitoring approach on exercise maintenance during cardiac rehabilitation: a randomized controlled trial. Am J Phys Med Rehabil. 2005;84:313–321.a							x								
Yates BC, Anderson T, Hertzog M, Ott C, Williams J. Effectiveness of follow-up booster sessions in improving physical status after cardiac rehabilitation: health, behavioral and clinical outcomes. App Nurs Res. 2005;18:59–62							x								
Hofman-Bang C, Lisspers J, Nordiander R, et al.. Two-year results of a controlled study of residential rehabilitation for patients treated with percutaneous transluminal coronary angioplasty: a randomized study of a multifactorial program. Eur Heart J. 1999;20:1465–1474.							x								
Hsin-Hsin L, Yun-Fang T, Pyng-Jing L, Pei-Kwei T. Effects of a therapeutic lifestyle-change programme on cardiac risk factors after coronary artery bypass graft. J Clin Nurs. 2010;19:60–69.							x								
Lewin B, Robertson IH, Cay EL, Irving JB, Campbell M. Effects of self-help post myocardial infarction rehabilitation on psychological adjustment and use of health services. Lancet. 1992;339:1036–1040.							x								
Lewin RJP, Furze G, Robinson J, Griffith K, Wiseman S, Boyle R. A randomized controlled trial of a self-management plan for patients with newly diagnosed angina. Br J Gen Pract. 2001;52:194–201.							x								
Bolman, C. Vries, de, H., Breukelen, van, G. Evaluation of nurse managed minimal contact smoking cessation intervention for cardiac patients Health Education Research 17 99–116 2002b.									x						

Burt, A., Thornley, P., Illingworth, D., White, P., Shaw, T. R., Turner, R. Stopping smoking after myocardial infarction Lancet 1 304–306 1974.									X						
Feeney, G., McPherson, A., Connor, J., McAlister, A., Young, M., Garrahy, P. Randomized controlled trial of two cigarette quit programmes in coronary care patients after acute myocardial infarction Internal Medicine Journal 31 470–475 2001.									X				X	X	
Johnson, J. L., Budz, B., Mackay, M., Miller, M. Evaluation of a nurse-delivered smoking cessation intervention for hospitalized patients with cardiac disease Heart and Lung 28 55–64 1999.									X						
Mohiuddin, S. M., Mooss, A. N., Hunter, C. B., Grollmes, T. L., Cloutier, D. A., Hilleman, D. E. Intensive smoking cessation intervention reduces mortality in high-risk smokers with cardiovascular disease Chest 131 446–452 2007.									X						
Ockene, J., Kristeller, J. L., Goldberg, R., Ockene, I., Merriam, P., Barrett, S. Smoking cessation and severity of disease: The coronary artery smoking cessation intervention study Health Psychology 11 119–126 1992.									X						
Reid, R., Pipe, A., Higginson, L., Johnson, K., D'Angelo, M. S., Cooke, D., Dafoe, W. Stepped care approach to smoking cessation in patients hospitalized for coronary artery disease Journal of Cardiopulmonary Rehabilitation 23 176–182 2003.									X				X		
Barnason S. & Zimmerman L. (1995) A comparison of patient teaching outcomes among postoperative coronary artery bypass graft (CABG) patients. Progress in Cardiovascular Nursing 10(4), 11–20.										X					
Bengtsson K. (1983) Rehabilitation after myocardial infarction. A controlled study. Scandinavian Journal of Rehabilitation Medicine 15(1), 1–9.										X					X
Bolman C., Brug J., Bär F., Martinali J. & Van Den Borne B. (2005) Long-term efficacy of a checklist to improve patient education in cardiology. Patient Education and Counseling 56, 240–248.										X					
Cupples S.A. (1991) Effects of timing and reinforcement of preoperative education on knowledge and recovery of patients having coronary artery bypass graft surgery. Heart and Lung 20(6), 654–660.										X					
Furze G., Dumville J.C., Miles J.N.V., Irvine K., Thompson D.R. & Lewin R.J.P. (2009) “Prehabilitation” prior to CABG surgery improves physical functioning and depression. International Journal of Cardiology 132, 51–58.										X					
Martinali J., Bolman C., Brug J., Van Den Borne B. & Bar F. (2001) A checklist to improve patient education in a cardiology outpatient setting. Patient Education and										X					

Counseling 42, 231–238.															
Petrie K.J., Cameron L.D., Ellis C.J., Buick D. & Weinman J. (2002) Changing illness perceptions after myocardial infarction: an early intervention randomized controlled trial. Psychosomatic Medicine 64, 580–586.										X					
Pozen M.W., Stechmiller J.A., Harris W., Smith S., Fried D. & Voigt G.C. (1977) A nurse rehabilitator's impact on patients with myocardial infarction. Medical Care 15(10), 830–837.										X	X				
Tullman D.F., Haugh K.H., Dracup K.A. & Bourguignon C. (2007) A randomized controlled trial to reduce delay in older adults seeking help for symptoms of acute myocardial infarction. Research in Nursing & Health 30, 485–497.										X					
Eagle KA, Montoye CK, Riba AL, DeFranco AC, Parrish R, Skorcz S, Baker PL, Faul J, Jani SM, Chen B. Guideline-based standardized care is associated with substantially lower mortality in medicare patients with acute myocardial infarction: the American College of Cardiology's Guidelines Applied in Practice (GAP) projects in Michigan. J Am Coll Cardiol. 2005;46:1242–1248.											X				
Fonarow GC, Gawlinski A, Moughrabi S, Tillisch JH. Improved treatment of coronary heart disease by implementation of a Cardiac Hospitalization Atherosclerosis Management Program (CHAMP). Am J Cardiol. 2001;87:819–822.											X				
Oldenburg B, Perkins RJ, Andrews G. Controlled trial of psychological intervention in myocardial infarction. J Consult Clin Psychol. 1985;53:852–859											X				
Hedback B, Perk J. 5-year results of a comprehensive rehabilitation programme after myocardial infarction. Eur Heart J. 1987;8:234–242											X				
Maeland JG, Havik OE. The effects of an in-hospital educational programme for myocardial infarction patients. Scand J Rehabil Med. 1987;19:57–65.											X				
Fletcher V. An individualized teaching programme following primary uncomplicated myocardial infarction. J Adv Nurs. 1987;12:195–200											X				
Thompson DR. A randomized controlled trial of in-hospital nursing support for first time myocardial infarction patients and their partners: effects on anxiety and depression. J Adv Nurs. 1989;14:291–297.											X				
Fridlund B, Hogstedt B, Lidell E, Larsson PA. Recovery after myocardial infarction: effects of a caring rehabilitation programme. Scand J Caring Sci. 1991;5:23–32.											X				

Nordmann A, Heilmbauer I, Walker T, Martina B, Battegay E. A case-management program of medium intensity does not improve cardiovascular risk factor control in coronary artery disease patients: the Heartcare I Trial. Am J Med. 2001;110:543–550.											X				
Jeong HS, Chae JS, Moon JS, Yoo YS. An individualized teaching program for atherosclerotic risk factor reduction in patients with myocardial infarction. Yonsei Med J. 2002;43:93–100.											X				
Echeverry D, Dike M, Jovanovic L, Wollitzer AO, Westphal S, Mudaliar S, Washington C, Davidson MB. Efforts to improve subsequent treatment of cardiovascular risk factors in older patients with diabetes hospitalized for a cardiac event. Am J Manag Care. 2005;11:758–764.											X		X		
Scalzi CC, Burke LE, Greenland S. Evaluation of an inpatient educational program for coronary patients and families. Heart Lung. 1980;9:846–853.											X				
Kushnir B, Fox K, Tomlinson I, Aber CP. The effect of a pre-discharge consultation on the resumption of work, sexual activity, and driving following acute myocardial infarction. Scand J Rehabil Med. 1976;8:155–159.											X				
Young DT, Kottke TE, McCall MM, Blume D. A prospective controlled study of in-hospital myocardial infarction rehabilitation. J Card Rehabil. 1982;2:32–40.											X				
Holmboe ES, Meehan TP, Radford MJ, Wang Y, Marciniak TA, Krumholz HM. Use of critical pathways to improve the care of patients with acute myocardial infarction. Am J Med. 1999;107:324–331.											X				
Kucenic MJ, Meyers DG. Impact of a clinical pathway on the care and costs of myocardial infarction. Angiology. 2000;51:393–404.											X				
Cheah J. Clinical pathways: an evaluation of its impact on the quality of care in an acute care general hospital in Singapore. Singapore Med J. 2000;41:335–346.											X				
Hickey A, Scott I, Denaro C, Stewart N, Bennett C, Theile T. Using clinical indicators in a quality improvement programme targeting cardiac care. Int J Qual Health Care. 2004;16:i11–i25.											X				
Scott IA, Denaro CP, Hickey AC, Bennett C, Mudge AM, Sanders DC, Thiele J, Flores JL. Optimising care of acute coronary syndromes in three Australian hospitals. Int J Qual Health Care. 2004;16:275–284.											X				
Lappe JM, Muhlestein JB, Lappe DL, Badger RS, Bair TL, Brockman R, French TK, Hofmann LC, Horne BD, Kralick-Goldberg S, Nicponski N, Orton JA, Pearson RR, Renlund DG, Rimmasch H, Roberts C, Anderson JL. Improvements in 1-year cardiovascular clinical outcomes associated with a hospital-based discharge medication											X				

program. Ann Intern Med. 2004;141:446–453.															
Grace C, Summerbell C. Does provision of additional dietary information affect actual or only reported compliance to a low-fat diet over 12 weeks in hyperlipidaemic individuals? Report of a pilot study. J Hum Nutr Diet 1996; 9: 303–7.													X		
Dalgård C, Thurøe A, Haastrup B, Haghfelt T, Stender S. Saturated fat intake is reduced in patients with ischemic heart disease 1 year after comprehensive counseling but not after brief counseling. J Am Diet Assoc 2001; 101: 1420–9.													X		
Levetan CS, Dawn KR, Murray JF, Popma JJ, Ratner RE, Robbins DC. Impact of computer-generated personalized goals on cholesterol lowering. Value Health 2005; 8: 639–46.													X		
Bolman CVH, van Breukelen G. Evaluation of a nurse-managed minimal-contact smoking cessation intervention for cardiac inpatients. Health Educ Res 2002; 17: 99–116.													X		
Sivarajan Froelicher ESS, Miller NH, Christoperson DJ et al. High rates of sustained smoking cessation in women hospitalized with cardiovascular disease: the Women's Initiative for Nonsmoking(WINS). Circulation 2004; 109: 587–93													X		
Feder G, Griffiths C, Eldridge S, Spence M. Effect of postal prompts to patients and general practitioners on the quality of primary care after a coronary event (POST): randomised controlled trial. Br Med J 1999; 318: 1522.													X		
Mahler HI, Kulik JA, Tarazi RY. Effects of a videotape information intervention at discharge on diet and exercise compliance after coronary bypass surgery. J Cardiopulm Rehabil 1999; 19:170–7.													X		
Allen JK: Coronary risk factor modification in woman after coronary artery by pass surgery.Nursing Research. 1996,45:260–265.															X
Carlsson R, Lindberg G, Westin L, Israelsson B: Influence of coronary nursing management follow up on lifestyle after acute myocardial infarction.Heart. 1997,77:256–259.															X
DeBusk RF, Miller N, Superko HR, et al.: Acase-management system for coronary risk factor modification after acute myocardial infarction.Annals of Internal Medicine. 1994,120:721–729.															X
Lisspers J, Sundin O, Hofman-Bang C, et al.: Behavioral effects of a comprehensive, multifactorial program for lifestyle change after percutaneous transluminal coronary angioplasty: A prospective, randomized, controlled study.Journal of Psychosomatic Research. 1999,46:143–154.															X
Mitsibounas DN, Tsouna-Hadjis ED, Sideris DA, Rotas															X

AR: Effects of group psychsocial intervention on coronary risk factors.Psychotherapy and Psychosomatics. 1992,58:97–102.															
Campbell NC, Thain J, Deans HG, Ritchie LD, Rawles JM, Squair JL. Secondary prevention clinics for coronary heart disease: randomised trial of effect on health. BMJ. 1998;316:1434-7.															X
Campbell NC, Ritchie LD, Thain J, Deans HG, Rawles JM, Squair JL. Secondary prevention in coronary heart disease: a randomised trial of nurse led clinics in primary care. Heart. 1998;80:447-52															X
Engblom E, Rönnemaa T, Hämäläinen H, Kallio V, Vänttinen E, Knuts LR. Coronary heart disease risk factors before and after bypass surgery: results of a controlled trial on multifactorial rehabilitation. Eur Heart J. 1992;13:232-7.															X
Taylor CB, Miller NH, Smith PM, DeBusk RF. The effect of a home-based, case-managed, multifactorial risk-reduction program on reducing psychological distress in patients with cardiovascular disease. J Cardiopulm Rehabil. 1997;17:157-62.															X
Murchie P, Campbell NC, Ritchie LD, Simpson JA, Thain J. Secondary prevention clinics for coronary heart disease: four year follow up of a randomised controlled trial in primary care. BMJ. 2003;326:84															X
Schuler G, Hambrecht R, Schlierf G, Niebauer J, Hauer K, Neumann J, et al. Regular physical exercise and low-fat diet. Effects on progression of coronary artery disease. Circulation. 1992;86:1-11.a															X
Niebauer J, Hambrecht R, Velich T, Hauer K, Marburger C, Kälberer B, et al. Attenuated progression of coronary artery disease after 6 years of multifactorial risk intervention: role of physical exercise. Circulation. 1997;96:2534-41.															X
Ornish D, Brown SE, Scherwitz LW, Billings JH, Armstrong WT, Ports TA, et al. Can lifestyle changes reverse coronary heart disease? The Lifestyle Heart Trial. Lancet. 1990;336:129-33															X
Ornish D, Scherwitz LW, Billings JH, Brown SE, Gould KL, Merritt TA, et al. Intensive lifestyle changes for reversal of coronary heart disease. JAMA. 1998;280:2001-7.															X
Naylor MD, Brooten D, Campbell R, Jacobsen BS, Mezey MD, Pauly MV, et al. Comprehensive discharge planning and home follow-up of hospitalized elders: a randomized clinical trial. JAMA. 1999;281:613-20.															X
Naylor MD, McCauley KM. The effects of a discharge planning and home follow-up intervention on elders hospitalized with common medical and surgical cardiac conditions. J Cardiovasc Nurs. 1999;14:44-54.															X
Dorossiev D. Rehabilitation and Comprehensive Secondary Prevention after Acute Myocardial Infarction. Report on a Study. EURO Reports and Studies No. 84. Copenhagen: World Health Organization; 1983.															X

Kallio V, Hämäläinen H, Hakkila J, Luurila OJ. Reduction in sudden deaths by a multifactorial intervention programme after acute myocardial infarction. Lancet. 1979;2:1091-4															X
Hämäläinen H, Luurila OJ, Kallio V, Knuts LR, Arstila M, Hakkila J. Long-term reduction in sudden deaths after a multifactorial intervention programme in patients with myocardial infarction: 10-year results of a controlled investigation. Eur Heart J. 1989;10:55-62															X
Hämäläinen H, Luurila OJ, Kallio V, Knuts LR. Reduction in sudden deaths and coronary mortality in myocardial infarction patients after rehabilitation. 15 year follow-up study. Eur Heart J. 1995;16:1839-44.															X
Sivarajan ES, Bruce RA, Lindskog BD, Almes MJ, Belanger L, Green B. Treadmill test responses to an early exercise program after myocardial infarction: a randomized study. Circulation. 1982;65:1420-8.															X
Vermeulen A, Lie KI, Durrer D. Effects of cardiac rehabilitation after myocardial infarction: changes in coronary risk factors and long-term prognosis. Am Heart J. 1983;105:798-801															X
Bell JM. A Comparison of a Multi-Disciplinary Home Based Cardiac Rehabilitation Programme with Comprehensive Conventional Rehabilitation in Post-Myocardial Infarction Patients [PhD thesis]. London: University of London; 1998.															X
Toobert DJ, Glasgow RE, Radcliffe JL. Physiologic and related behavioral outcomes from the Women's Lifestyle Heart Trial. Ann Behav Med. 2000;22:1-9.															X
Sundin O, Lisspers J, Hofman-Bang C, Nygren A, Rydén L, Ohman A. Comparing multifactorial lifestyle interventions and stress management in coronary risk reduction. Int J Behav Med. 2003;10:191-204.															X
Yu CM, Li LS, Ho HH, Lau CP. Long-term changes in exercise capacity, quality of life, body anthropometry, and lipid profiles after a cardiac rehabilitation program in obese patients with coronary heart disease. Am J Cardiol. 2003;91:321-5.															X
Marchionni N, Fattirolli F, Fumagalli S, Oldridge N, Del Lungo F, Morosi L, et al. Improved exercise tolerance and quality of life with cardiac rehabilitation of older patients after myocardial infarction: results of a randomized, controlled trial. Circulation. 2003;107:2201-6.															X
Masley S, Phillips S, Copeland JR. Group office visits change dietary habits of patients with coronary artery disease—the dietary intervention and evaluation trial (D.I.E.T.). J Fam Pract. 2001;50:235-9.															X
Ornish D, Scherwitz LW, Doody RS, Kesten D, McLanahan SM, Brown SE, et al. Effects of stress management training and dietary changes in treating ischemic heart disease. JAMA. 1983;249:54-9.															X
Fitzgerald JF, Smith DM, Martin DK, Freedman JA, Katz															X

BP. A case manager intervention to reduce readmissions. Arch Intern Med. 1994;154:1721-9.															
Naylor M, Brooten D, Jones R, Lavizzo-Mourey R, Mezey M, Pauly M. Comprehensive discharge planning for the hospitalized elderly. A randomized clinical trial. Ann Intern Med. 1994;120:999-1006															X
Carlsson R. Serum cholesterol, lifestyle, working capacity and quality of life in patients with coronary artery disease. Experiences from a hospital-based secondary prevention programme. Scand Cardiovasc J Suppl. 1998;50:1-20															X
Jolly K, Bradley F, Sharp S, Smith H, Thompson S, Kinmonth AL, et al. Randomised controlled trial of follow up care in general practice of patients with myocardial infarction and angina: final results of the Southampton heart integrated care project (SHIP). The SHIP Collaborative Group. BMJ. 1999;318:706-11.															X
Allison TG, Farkouh ME, Smars PA, Evans RW, Squires RW, Gabriel SE, et al. Management of coronary risk factors by registered nurses versus usual care in patients with unstable angina pectoris (a chest pain evaluation in the emergency room [CHEER] substudy). Am J Cardiol. 2000;86:133-8															X
Moher M, Yudkin P, Wright L, Turner R, Fuller A, Schofield T, et al. Cluster randomised controlled trial to compare three methods of promoting secondary prevention of coronary heart disease in primary care. BMJ. 2001;322:1338.															X
Higgins HC, Hayes RL, McKenna KT. Rehabilitation outcomes following percutaneous coronary interventions (PCI). Patient Educ Couns. 2001;43:219-30.															X
Allen JK, Blumenthal RS, Margolis S, Young DR, Miller ER 3rd, Kelly K. Nurse case management of hypercholesterolemia in patients with coronary heart disease: results of a randomized clinical trial. Am Heart J. 2002;144:678-86															X
Lear SA, Ignaszewski A, Linden W, Brozic A, Kiess M, Spinelli JJ, et al. The Extensive Lifestyle Management Intervention (ELMI) following cardiac rehabilitation trial. Eur Heart J. 2003;24:1920-7.															X
Young W, Rewa G, Goodman SG, Jaglal SB, Cash L, Lefkowitz C, et al. Evaluation of a community-based inner-city disease management program for postmyocardial infarction patients: a randomized controlled trial. CMAJ. 2003;169:905-10.															X
Lichtman JH, Amatruda J, Yaari S, Cheng S, Smith GL, Mattera JA, et al. Clinical trial of an educational intervention to achieve recommended cholesterol levels in patients with coronary artery disease. Am Heart J. 2004;147:522-8.															X
Haskell WL, Alderman EL, Fair JM, Maron DJ, Mackey SF, Superko HR, et al. Effects of intensive multiple risk factor reduction on coronary atherosclerosis and clinical cardiac events in men and women with coronary artery disease. The Stanford Coronary Risk Intervention Project															X

(SCRIP). Circulation. 1994;89:975-90.															
Allison TG, Squires RW, Johnson BD, Gau GT. Achieving National Cholesterol Education Program goals for low-density lipoprotein cholesterol in cardiac patients: importance of diet, exercise, weight control, and drug therapy. Mayo Clin Proc. 1999;74:466-73															X
Stagmo M, Westin L, Carlsson R, Israelsson B. Long-term effects on cholesterol levels and the utilization of lipid-lowering drugs of a hospital-based programme for secondary prevention of coronary artery disease. J Cardiovasc Risk. 2001;8:243-8.															X
Wilhelmsen L, Sanne H, Elmfeldt D, Grimby G, Tibblin G, Wedel H. A controlled trial of physical training after myocardial infarction. Effects on risk factors, nonfatal reinfarction, and death. Prev Med. 1975;4:491-508.															X
Shaw LW. Effects of a prescribed supervised exercise program on mortality and cardiovascular morbidity in patients after myocardial infarction. The National Exercise and Heart Disease Project. Am J Cardiol. 1981;48:39-46															X
Carson P, Phillips R, Lloyd M, Tucker H, Neophytou M, Buch NJ, et al. Exercise after myocardial infarction: a controlled trial. J R Coll Physicians Lond. 1982;16:147-51.															X
Ballantyne FC, Clark RS, Simpson HS, Ballantyne D. The effect of moderate physical exercise on the plasma lipoprotein subfractions of male survivors of myocardial infarction. Circulation. 1982;65:913-8.															X
Miller NH, Haskell WL, Berra K, DeBusk RF. Home versus group exercise training for increasing functional capacity after myocardial infarction. Circulation. 1984;70:645-9.															X
Roviaro S, Holmes DS, Holmsten RD. Influence of a cardiac rehabilitation program on the cardiovascular, psychological, and social functioning of cardiac patients. J Behav Med. 1984;7:61-81.															X
Erdman RA, Duivenvoorden HJ, Verhage F, Kazemier M, Hugenholtz PG. Predictability of beneficial effects in cardiac rehabilitation: a randomized clinical trial of psychosocial variables. J Cardiopulm Rehabil. 1986;6:206-13.															X
Agren B, Olin C, Castenfors J, Nilsson-Ehle P. Improvements of the lipoprotein profile after coronary bypass surgery: additional effects of an exercise training program. Eur Heart J. 1989;10:451-8.															X
Bethell HJ, Mullee MA. A controlled trial of community based coronary rehabilitation. Br Heart J. 1990;64:370-5															X
Bertie J, King A, Reed N, Marshall AJ, Ricketts C. Benefits and weaknesses of a cardiac rehabilitation programme. J R Coll Physicians Lond. 1992;26:147-51.															X
Fletcher BJ, Dunbar SB, Felner JM, Jensen BE, Almon L, Cotsonis G, et al. Exercise testing and training in physically disabled men with clinical evidence of coronary artery disease. Am J Cardiol. 1994;73:170-4															X
Holmbäck AM, Säwe U, Fagher B. Training after myocardial infarction: lack of long-term effects on physical capacity and psychological variables. Arch Phys Med															X

Rehabil. 1994;75:551-4.															
Wosornu D, Bedford D, Ballantyne D. A comparison of the effects of strength and aerobic exercise training on exercise capacity and lipids after coronary artery bypass surgery. Eur Heart J. 1996;17:854-63.															X
Heldal M, Sire S, Dale J. Randomised training after myocardial infarction: short and long-term effects of exercise training after myocardial infarction in patients on beta-blocker treatment. A randomized, controlled study. Scand Cardiovasc J. 2000;34:59-64.															X
Ståhle A, Lindquist I, Mattsson E. Important factors for physical activity among elderly patients one year after an acute myocardial infarction. Scand J Rehabil Med. 2000;32:111-6.															X
Belardinelli R, Paolini I, Cianci G, Piva R, Georgiou D, Purcaro A. Exercise training intervention after coronary angioplasty: the ETICA trial. J Am Coll Cardiol. 2001;37:1891-900.															X
	16	20	42	26	24	62	7	13	14	13	27	18	17	19	74

ACS = acute coronary syndrome;

% overlap 63/286= 22.0%

CA= Covered Area = (16+20+42+26+24+62+7+13+14+13+27+18+17+19+74)/ (15x286) = 392/4290=9.1%

CCA= Corrected Covered Area = (392-286)/ (4290-286) = 106/4004= 2.6% (slight)

Supplementary Annex 3.

Citation Matrix: Non-repeated trials (rows) included in the 36 systematic review and/or meta-analyses (column)--T2DM.

Author-year (Trial reference)	1	2	3	4	5	6	7	8	9	10	11	12	13	14	15	16	17	18	19	20	21	22	23	24	25	26	27	28	29	30	31	32	33	34	35	36
K. Cai, S. Zhang, M. Lu, L. Shao The effects of intensive education for elderly patients with diabetes mellitus. Pract Geriatr, 18 (2004), pp. 313–315	X																																			
Y. Chen, W. Xue, S. Lin, S. Huang, D. Li. ntensive diabetes education in the role of diabetes control. J Sun Yat-sen Univ (Med Sci), 3S (2004), pp. 201–203	X																																			
L. Chen, M. Zhu. Clinical investigation of the effect of type 2 diabetes health education. Strait Pharm J, 21 (2009), pp. 217–218	X																																			
Y. Fang. Coordinated health education model in diabetes education. Today Nurse, 3 (2011), pp. 148–149	X																																			
B. Li, Y. Liu, J. Tian, J. Yuan. Impact of diabetes education for type 2 diabetes on glycated haemoglobin and health cost.Jiangsu J Prev Med, 23 (2012), pp. 55–56	X																																			
B.-h. Li, F.-h. Zhang, C. Zhang, M. Yang Effect of different education models on blood glucose in diabetic patients J Qilu Nurs, 19 (2013), pp. 26–27	X																																			
M. Li, T. Li, B.-y. Shi, C.-x. Gao Effect of motivational interviewing on the quality of life in type 2 diabetes patients with poor control of blood glucose Chin J Nurs, 49 (2014), pp. 6–10	X																																			
X. Li, Q. Zhou, F. Zou, L. Wu, H. Chen, Z. Liu Effectiveness of systematic self-management education on blood sugar level of patients in the community with type 2 diabetes J Central South Univ (Med Sci), 37 (2012), pp. 355–357	X																																			
Y. Li Evaluation of the standardized management effect of diabetes in community Chin Prim Health Care, 25 (2011), pp. 33–35	X																																			
Q. Liu, X. Pan, W. Feng, J. Kong Effectiveness of health education to the patients with diabetes mellitus J Nurs Sci, 20 (2005), pp. 54–55	X																																			
Z.-p. Liu, L.-l. Han, J.-r. Deng, X.-r. Liu, X.-r. Zhang, Q.-f. Li Effect of short-term intensified education on glucose	X																																			

control of type 2 diabetic patients<br>Chin J Nurs Educ, 4 (2007), pp. 249–251																																				
L.-m. Luo, K.-J. Yang, Z.-J. Zhang, Y.-H. Li, C.-H. Hu, X.-L. Feng<br>Role of health education on diabetic community chronic disease management<br>China Pharm, 24 (2013), pp. 3020–3022	X																																			
Y. Lu<br>Observation on the effect of diabetes intensive education in type 2 diabetes mellitus patients<br>Med Inform, 21 (2008), pp. 690–692	X																																			
X. Mei<br>Analysis of compliance and effectiveness of community diabetes education program<br>J Nanchang Univ (Med Sci), 50 (2010), pp. 83–84	X																																			
G. Pang<br>The impact of intensive diabetes education on community patients<br>J Med Theory Pract, 21 (2008), pp. 839–840	X																																			
Y. Qi, C. Chen, L. Yan<br>Impact of cooking class on diabetic patients eating habits<br>Chin J Nurs, 41 (2006), pp. 126–127	X																																			
B. Sun, B. Ban, H.-l. Sun, S. Zhang, S. Jie<br>Effects of diabetes education and psychological intervention on comprehensive treatment of type 2 diabetes<br>Chin J Clin Psychol, 13 (2005), pp. 483–485	X																						X													
M. Sun, X. Li, F. Wang, D. Zhu<br>Study on the effect of diabetes education<br>J Pract Diagn Ther, 19 (2005), pp. 422–423	X																																			
Z. Sun, M. Lv<br>90 cases of individualised type 2 diabetes education<br>J Qilu Nurs, 14 (2008), pp. 106–107	X																																			
H. Wang, S. Hu<br>Comparison of clinical applicability of individual intervention with intensive group education for diabetic patients<br>People’s Mil Surg, 53 (2009), pp. 234–235	X																																			
X. Wang, H. Zou, H. Jie, S. Shen, J. Zhang<br>Effect of individualised diabetes education and care for aged patients with diabetes<br>Chin J Clin Res, 26 (2013), pp. 90–91	X																																			
Y. Wei<br>Effectiveness of intensive education for patients with diabetes mellitus<br>Chin J Health Educ, 21 (2005), pp. 53–55	X																																			
S.-q. Wu, G.-x. Jin, Y.-f. Chen	X																																			

Effect observation of sample diet education on diet control of patients with diabetes<br>J Nurs (China), 13 (2006), pp. 17–19																																				
Y. Wu, K. Wang, X. Yang<br>The effect of diabetes self-management education curriculum for patients with diabetes<br>Guide China Med, 11 (2013), pp. 395–397	X																																			
X.-L. Xiao, Q.-Q. Hu, Y.-P. Liu<br>Effects of family involvement in health education for patients with type 2 diabetes<br>Chin J Nurs, 47 (2012), pp. 313–316	X																																			
B. Xie, C.-p. Ju, Z.-l. Sun, J. Han, L. Liu<br>Effect of education network for diabetes self-management on self-efficacy in patients with type 2 diabetes mellitus<br>Chin Gen Pract, 15 (2012), pp. 3610–3616	X																																			
H. Xie, C. Cheng, D. Chen<br>Intensive diabetes education for poorly controlled elderly diabetic patients in the community<br>J Pract Med Tech, 14 (2007), pp. 3958–3959	X																																			
S. Xu<br>Effect of continued outpatient health education on diabetes patients<br>Mod Clin Nurs, 6 (2007), pp. 28–30	X																																			
L. Yan, C. Zhu, X. Tan<br>“Diabetes conversation” map interactive education for diabetic patients<br>Chin J Misdiagn, 11 (2011), pp. 6383–6384	X																																			
X. Yan, N. Li, H. Liu, H. Sha, P. Li<br>Effect of “interactive groups” diabetes education model on diabetes patients in the village<br>Nurs Pract Res, 9 (2012), pp. 13–14	X																																			
S. Yao, Y. Liu, R. Ji, G. Wang<br>Effectiveness of diabetes education for patients with type 2 diabetes<br>Hebei J Tradit Chin Med, 34 (2012), pp. 1723–1724	X																																			
W. Yu, Y.-l. Bi, J. Han, et al.<br>Affect of the MUST education mode on compliance of patients with type 2 diabetes mellitus<br>J Shandong Univ (Health Sci), 49 (2011), pp. 1–4	X																																			
Y. Zeng, A. Wang, M. Gong<br>Clinical observation of communication management targeted at patients with type 2 diabetes<br>J Pract Diabetol, 5 (2009), pp. 48–49	X																																			
F. Zhang, B. Xu<br>Influence of diabetes education on patients lifestyle and metabolic indicators	X																																			

Mod Med J China, 12 (2010), pp. 116–117																																				
J. Zhang, Y. Zhang, X. Huang<br>“Diabetes conversation” map interactive education for type 2 diabetes patients<br>Guide China Med, 9 (2011), pp. 330–331	X																																			
L. Zhang<br>Analysis on the therapeutic effects of diabetes education<br>Tianjin J Tradit Chin Med, 26 (2009), pp. 22–23	X																																			
X.-q. Zhang, L.-f. Fan, Y.-g. Zheng<br>Effect of health education applied in promoting compliance of diet treatment of patients with diabetes mellitus<br>Mod Nurs, 12 (2006), pp. 1082–1084	X																																			
X. Zheng, H. Tang, Y. Huang<br>Strengthen health education on observed efficacy in patients with type 2 diabetes<br>Youjiang Med J, 33 (2005), pp. 349–350	X																																			
C. Zhong, J. Li, X. Yang<br>Effect of interactive diabetes education for patients with diabetes<br>Jilin Med J, 27 (2006), pp. 1490–1491	X																																			
A. Zhou, Y. Ying, X. Dai, et al.<br>Application of text message to diabetic education<br>J Nurs Sci, 27 (2012), pp. 17–19	X																																			
M.-l. Gu, X.-g. Zhu, X.-f. Xu<br>The study of comprehensive intervention for patients with type 2 diabetes in community of Shanghai<br>Chin Gen Pract, 15 (2012), pp. 107–109	X																																			
Z.-M. Huang<br>Impact of intensive diabetes education targeted at patients with type 2 diabetes<br>Shanghai J Prev Med, 21 (2009), pp. 323–324	X																																			
C. Liu, W. Sun, J. Wang, J. Liu<br>Effect of community management of type 2 diabetic patients<br>Chin J Prev Control Chronic Dis, 20 (2012), pp. 624–625	X																																			
J. Sun, Y. Wang, X. Chen, et al.<br>An integrated intervention program to control diabetes in overweight Chinese women and men with type 2 diabetes<br>Asia Pac J Clin Nutr, 17 (2008), pp. 514–524	X																																			
X. Wang, X. Zhou, H. Zhen, J. Sun, C. Cui<br>Impact of stratified intensive education for patients with diabetes<br>J Med Theory Pract, 18 (2005), pp. 336–337	X																																			
X. Wang, X. Zhou, H. Zhen, J. Sun, C. Cui	X																																			

Impact of stratified intensive education for patients with diabetes<br>J Med Theory Pract, 18 (2005), pp. 336–337																																				
B. Yang, C. Ma, X. Feng, et al.<br>Effect of face-to-face education on metabolic improvement in Tibetan diabetic patients<br>J Clin Rehabil Tissue Eng Res, 11 (2007), pp. 10467–10470	X																																			
G.T. Ko, J.K. Li, E.C. Kan, M.K. Lo<br>Effects of a structured health education programme by a diabetic education nurse on cardiovascular risk factors in Chinese type 2 diabetic patients: a 1-year prospective randomized study<br>Diabet Med, 21 (2004), pp. 1274–1279	X		X	X																		X		X												
A. Lee, C.F. Siu, K.T. Leung, L.C. Lau, C.C. Chan, K.W. Kwok<br>General practice and social service partnership for better clinical outcomes, patient self efficacy and lifestyle behaviours of diabetic care: randomised control trial of a chronic care model Postgrad Med J, 87 (2011), pp. 688–693	X				X																															
S.M. Chen, D. Creedy, H.S. Lin, J. Wollin<br>Effects of motivational interviewing intervention on self-management, psychological and glycemic outcomes in type 2 diabetes: a randomized controlled trial<br>Int J Nurs Stud, 49 (2012), pp. 637–644	X																																			
M.C. Huang, C.C. Hsu, H.S. Wang, S.J. Shin<br>Prospective randomized controlled trial to evaluate effectiveness of registered dietitian-led diabetes management on glycemic and diet control in a primary care setting in Taiwan<br>Diabetes Care, 33 (2010), pp. 233–239	X																																			
M.C. Huang, C.C. Hsu, H.S. Wang, S.J. Shin<br>Prospective randomized controlled trial to evaluate effectiveness of registered dietitian-led diabetes management on glycemic and diet control in a primary care setting in Taiwan<br>Diabetes Care, 33 (2010), pp. 233–239	X																																			
M. Jian, L. Liu, H.-c. Chiang<br>Building a certificate-based secure and quick response telemedical health-care system for diabetic patients<br>J Intern Med Taiwan, 17 (2006), pp. 61–72	X																																			
T.I. Lee, Y.T. Yeh, C.T. Liu, P.L. Chen<br>Development and evaluation of a patient-oriented education system for diabetes management<br>Int J Med Inform, 76 (2007), pp. 655–663	X																			X																
Agurs-Collins TD, Kumanyika SK, Ten Have TR, Adams-Campbell LL. A randomized controlled trial of weight reduction and exercise for diabetes management in		X			X		X			X		X				X					X	X				X				X				X	X	X

older African-American subjects. Diabetes Care 1997; 20: 1503–1511.																																				
Anderson RM, Funnell MM, Nwankwo R, Gillard ML, Oh M, Fitzgerald JT. Evaluating a problem-based empowerment program for African Americans with diabetes: results of a randomized controlled trial. Ethn Dis 2005; 15: 671–678.		X			X		X			X						X					X	X				X										
Babamoto KS, Sey KA, Camilleri AJ, Karlan VJ, Catalasan J, Morisky DE. Improving diabetes care and health measures among hispanics using community health workers: results from a randomized controlled trial. Health Educ Behav 2009; 36: 113–126.		X					X			X																										
Baradaran HR, Knill-Jones RP, Wallia S, Rodgers A. A controlled trial of the effectiveness of a diabetes education programme in a multi-ethnic community in Glasgow [ISRCTN28317455]. BMC Public Health 2006; 18: 134.		X					X			X							X				X					X	X									
Bellary S, O'Hare JP, Raymond NT, Gumber A, Mughal S, Szczepura A et al. Enhanced diabetes care to patients of south Asian ethnic origin (the United Kingdom Asian Diabetes Study): a cluster randomised controlled trial. Lancet 2008; 371: 1769–1776.		X					X														X															
Brown SA, Garcia AA, Kouzekanani K, Hanis CL. Culturally competent diabetes self-management education for Mexican Americans: the Starr County border health initiative. Diabetes Care 2002; 25: 259–268.		X			X		X			X						X	X				X	X				X		X				X				
Carter EL, Nunlee-Bland G, Callender C. A patient-centric, provider-assisted diabetes telehealth self-management intervention for urban minorities. Perspect Health Inf Manag: Winter 2011.		X					X																													
Crowley MJ, Powers BJ, Olsen MK, Grubber JM, Koropchak C, Rose CM et al. The Cholesterol, Hypertension, And Glucose Education (CHANGE) study: results from a randomized controlled trial in African Americans with diabetes. Am Heart J 2013; 166: 179–186.		X					X																													
D'Eramo Melkus G, Chyun D, Vorderstrasse A, Newlin K, Jefferson V, Langerman S. The effect of a diabetes education, coping skills training, and care intervention on physiological and psychosocial outcomes in black women with type 2 diabetes. Biol Res Nurs 2010; 12:7–19.		X			X		X																													
DePue JD, Dunsiger S, Seiden AD, Blume J, Rosen RK, Goldstein MG. Nurse-community health worker team improves diabetes care in American Samoa: results of a randomized controlled trial. Diabetes Care 2013; 36: 1947–1953.		X					X																													
Gary TL, Batts-Turner M, Yeh HC, Hill-Briggs F, Bone LR, Wang NY et al. The effects of a nurse case manager		X					X																													

and a community health worker team on diabetic control, emergency department visits, and hospitalizations among urban African Americans with type 2 diabetes mellitus: a randomized controlled trial. Arch Intern Med 2009; 169: 1788–1794.																																				
Gucciardi E, Demelo M, Lee RN, Grace SL. Assessment of two culturally competent diabetes education methods: individual versus individual plus group education in Canadian Portuguese adults with type 2 diabetes. Ethn Health 2007; 12: 163–187.		X					X									X										X										
Hawthorne K, Tomlinson S. One-to-one teaching with pictures–flashcard health education for British Asians with diabetes. Br J Gen Pract 1997; 47: 301–304.		X					X			X						X					X			X		X	X							X		X
Kattelmann KK, Conti K, Ren C. The medicine wheel nutrition intervention: a diabetes education study with the Cheyenne River Sioux Tribe. J Am Diet Assoc 2009; 109: 1532–1539.		X					X																													
Keyserling TC, Samuel-Hodge CD, Ammerman AS, Ainsworth BE, Henríquez-Roldán CF, Elasy TA et al. A randomized trial of an intervention to improve self-care behaviors of African-American women with type 2 diabetes: impact on physical activity. Diabetes Care 2002; 25: 1576–1583.		X			X		X			X		X										X														
Khan MA, Shah S, Grudzien A, Onyejekwe N, Banskota P, Karim S et al. A diabetes education multimedia program in the waiting room setting. Diabetes Therapy 2011; 2: 178–188.		X					X																													
Kim MT, Han HR, Song HJ, Lee JE, Kim J, Ryu JP et al. A community-based, culturally tailored behavioral intervention for Korean Americans with type 2 diabetes. Diabetes Educator 2009; 35: 986–944.		X			X		X									X																				
Lorig K, Ritter PL, Villa F, Piette JD. Spanish diabetes self-management with and without automated telephone reinforcement: two randomized trials. Diabetes Care 2008; 31: 408–414.		X			X		X																													
Lujan J, Ostwald SK, Ortiz M. Promotora diabetes intervention for Mexican Americans. Diabetes Educator 2007; 33: 660–670.		X			X		X			X							X																			
Middelkoop BJ, Geelhoed-Duijvestijn PH, van der Wal G. Effectiveness of culture-specific diabetes care for Surinam South Asian patients in the Hague: a randomized controlled trial/controlled before-and-after study. Diabetes Care 2001; 24: 1997–1998		X					X			X						X					X					X	X									
O'Hare JP, Raymond NT, Mughal S, Dodd L, Hanif W, Ahmad Y et al. Evaluation of delivery of enhanced diabetes care to patients of South Asian ethnicity: the United Kingdom Asian Diabetes Study (UKADS).		X					X									X					X					X	X									

Diabetic Med 2004; 221: 1357–1365.																																				
Osborn CY, Amico KR, Cruz N, O'Connell AA, Perez-Escamilla R, Kalichman SC et al. A brief culturally tailored intervention for Puerto Ricans with type 2 diabetes. Health Educ Behav 2010; 37: 849–862.		X					X																													
Philis-Tsimikas A, Fortmann A, Lleva-Ocana L, Walker C, Gallo LC. Peer-led diabetes education programs in high-risk Mexican Americans improve glycemic control compared with standard approaches: a Project Dulce promotora randomized trial. Diabetes Care 2011; 34: 1926–1931		X			X		X			X																										
Rosal MC, Olendzki B, Reed GW, Gumieniak O, Scavron J, Ockene I. Diabetes self-management among low-income Spanish-speaking patients: a pilot study. Ann Behav Med 2005; 29: 225–235.		X			X		X			X						X	X				X	X				X										
Rothschild SK, Martin MA, Swider SM, Tumialán Lynas CM, Janssen I, Avery EF et al. Mexican American trial of community health workers: a randomized controlled trial of a community health worker intervention for Mexican Americans with type 2 diabetes mellitus. Am J Public Health 2014; 104: 1540–1548.		X			X		X																													
Samuel-Hodge CD, Keyserling TC, Park S, Johnston LF, Gizlice Z, Bangdiwala SI. A randomized trial of a church-based diabetes self-management program for African Americans with type 2 diabetes. Diabetes Educator 2009; 35: 439–453.		X			X		X																													
Sixta CS, Ostwald S. Texas-Mexico border intervention by promotores for patients with type 2 diabetes. Diabetes Educ 2008; 34: 299–309.		X			X		X																													
Skelly AH, Carlson JR, Leeman J, Holditch-Davis D, Soward AC. Symptom-focused management for African American women with Type 2 diabetes: a pilot study. Appl Nurs Res 2005; 18: 213–220.		X			X		X					X										X														
Skelly AH, Carlson J, Leeman J, Soward A, Burns D. Controlled trial of nursing interventions to improve health outcomes of older African American women with type 2 diabetes. Nurs Res 2009; 58: 410–418.		X			X		X			X																										
Spencer MS, Rosland AM, Kieffer EC, Sinco BR, Valerio M, Palmisano G et al. Effectiveness of a community health worker intervention among African American and Latino adults with type 2 diabetes: a randomized controlled trial. Am J Public Health 2011; 101: 2253–2260.		X			X		X																													
Toobert DJ, Strycker LA, Barrera M Jr, Osuna D, King DK, Glasgow RE. Outcomes from a multiple risk factor diabetes self-management trial for Latinas: ¡Viva Bien!. Ann Behav Med 2011; 41: 310–323.		X																																		

Vincent D, Pasvogel A, Barrera L. A feasibility study of a culturally tailored diabetes intervention for Mexican Americans. Biol Res Nurs 2007; 9: 130–141.		X			X					X						X																				
A.R.G. Look, R.R. Wing Long-term effects of a lifestyle intervention on weight and cardiovascular risk factors in individuals with type 2 diabetes mellitus: four-year results of the Look AHEAD trial. Arch Intern Med, 170 (2010), pp. 1566–1575			X	X																																
D.A. Dobrosielski, B.B. Gibbs, P. Ouyang, S. Bonekamp, J.M. Clark, N.Y. Wang, et al. Effect of exercise on blood pressure in type 2 diabetes: a randomized controlled trial J Gen Intern Med, 27 (2012), pp. 1453–1459			X	X																																
W. Wisse, M. Boer Rookhuizen, M.D. de Kruif, J. van Rossum, I. Jordans, H. ten Cate, et al. Prescription of physical activity is not sufficient to change sedentary behavior and improve glycemic control in type 2 diabetes patients Diabetes Res Clin Pract, 88 (2010), pp. e10–e13			X	X																																
S. Balducci, S. Zanuso, A. Nicolucci, P. De Feo, S. Cavallo, P. Cardelli, et al. Effect of an intensive exercise intervention strategy on modifiable cardiovascular risk factors in subjects with type 2 diabetes mellitus: a randomized controlled trial: the Italian Diabetes and Exercise Study (IDES) Arch Intern Med, 170 (2010), pp. 1794–1803			X	X																																
A. Kirk, N. Mutrie, P. MacIntyre, M. Fisher Effects of a 12-month physical activity counselling intervention on glycaemic control and on the status of cardiovascular risk factors in people with type 2 diabetes Diabetologia, 47 (2004), pp. 821–832			X	X																		X														
K.J. Coppell, M. Kataoka, S.M. Williams, A.W. Chisholm, S.M. Vorgers, J.I. Mann Nutritional intervention in patients with type 2 diabetes who are hyperglycaemic despite optimised drug treatment—Lifestyle Over and Above Drugs in Diabetes (LOADD) study: randomised controlled trial BMJ, 341 (2010), p. c3337			X	X																																
M. Uusitupa, J. Laitinen, O. Siitonen, E. Vanninen, K. Pyorala The maintenance of improved metabolic control after intensified diet therapy in recent type 2 diabetes Diabetes Res Clin Pract, 19 (1993), pp. 227–238			X	X																										X					X	X
M. Ali, F. Schifano, P. Robinson, G. Phillips, L. Doherty, P. Melnick, et al. Impact of community pharmacy diabetes monitoring and education programme on diabetes management: a randomized controlled study Diabet Med, 29 (9) (2012), pp. e326–e333			X	X																																

H. Mohamed, B. Al-Lenjawi, P. Amuna, F. Zotor, H. Elmahdi<br>Culturally sensitive patient-centred educational programme for self-management of type 2 diabetes: a randomized controlled trial<br>Prim Care Diabetes, 7 (2013), pp. 199–206			x																																	
C.W. Chan, S.C. Siu, C.K. Wong, V.W. Lee<br>A pharmacist care program: positive impact on cardiac risk in patients with type 2 diabetes<br>J Cardiovasc Pharmacol Ther, 17 (2012), pp. 57–64			x	x																																
M.A. Sevick, M. Korytkowski, R.A. Stone, B. Piraino, D. Ren, S. Sereika, et al.<br>Biophysiologic outcomes of the Enhancing Adherence in Type 2 Diabetes (ENHANCE) trial<br>J Acad Nutr Diet, 112 (2012), pp. 1147–1157			x	x	x																															
W. Crasto, J. Jarvis, K. Khunti, T.C. Skinner, L.J. Gray, J. Brela, et al.<br>Multifactorial intervention in individuals with type 2 diabetes and microalbuminuria: the Microalbuminuria Education and Medication Optimisation (MEMO) study<br>Diabetes Res Clin Pract, 93 (2011), pp. 328–336			x	x																																
M.A. Salinero-Fort, E. Carrillo-de Santa Pau, F.J. Arrieta-Blanco, J.C. Abanades-Herranz, C. Martin-Madrazo, B. Rodes-Soldevila, et al. Effectiveness of PRECEDE model for health education on changes and level of control of HbA1c, blood pressure, lipids, and body mass index in patients with type 2 diabetes mellitus<br>BMC Public Health, 11 (2011), p. 267			x		x																															
H. Sone, S. Tanaka, S. Iimuro, S. Tanaka, K. Oida, Y. Yamasaki, et al.<br>Long-term lifestyle intervention lowers the incidence of stroke in Japanese patients with type 2 diabetes: a nationwide multicentre randomised controlled trial (the Japan Diabetes Complications Study)<br>Diabetologia, 53 (2010), pp. 419–428			x	x																																
S.L. Krein, M.L. Klamerus, S. Vijan, J.L. Lee, J.T. Fitzgerald, A. Pawlow, et al.<br>Case management for patients with poorly controlled diabetes: a randomized trial<br>Am J Med, 116 (2004), pp. 732–739			x	x																		x														
M. Trento, P. Passera, M. Bajardi, M. Tomalino, G. Grassi, E. Borgo, et al.<br>Lifestyle intervention by group care prevents deterioration of type II diabetes: a 4-year randomized controlled clinical trial. Diabetologia, 45 (2002), pp.<br>1231–1239			x	x													x	x				x						x				x				
AdachiMYamaokaKWatanabeMNishikawaMKobayashiI HidaEet alEffects of lifestyle education program for type 2 diabetes patients in clinics: a cluster randomized controlled trial.BMC Public Health201313467.					x																															

AdolfssonETWalker-EngströmMLSmideBWikbladKPatient education in type 2 diabetes: a randomized controlled 1-year follow-up study.Diabetes Res Clin Pract20077634150					X												X					X														
AmoakoESkellyAHRossenEKOutcomes of an intervention to reduce uncertainty among African American women with diabetes.West J Nurs Res20083092842					X					X																										
AndersonDRChristison-LagayJVillagraVLiuHDziuraJManaging the space between visits: a randomized trial of disease management for diabetes in a community health center.J Gen Intern Med201025111622					X																															
AndersonRMFunnellMMAikensJEKreinSLFitzgeraldJTNwankwoRet alEvaluating the efficacy of an empowerment-based self-management consultant intervention: results of a two-year randomized controlled trial.Ther Patient Educ20091311					X																															
AndersonRMFunnellMMButlerPMArnoldMSFitzgeraldJTFesteCCPatient empowerment. Results of a randomized controlled trial.Diabetes Care1995189439					X																													X		X
Anderson-LoftinWBarnett SBunnP SullivanPHusseyJTavakoli A Soul food light: culturally competent diabetes education.Diabetes Educ20053155563					X					X		X				X																				
BaksiAKAl-MrayatMHoganDWhittingstallEWilsonPWexJ Peer advisers compared with specialist health professionals in delivering a training programme on self-management to people with diabetes: a randomized controlled trial.Diabet Med200825107682					X																															
BarrattRFrostGMillwardDJTrubyHA randomised controlled trial investigating the effect of an intensive lifestyle intervention v.standard care in adults with type 2 diabetes immediately after initiating insulin therapy. Br J Nutr200899102531					X																															
Beverly EA, Fitzgerald SM, Brooks KM, Hultgren BA, Ganda OP,Munshi M, et al. Impact of reinforcement of diabetes self-care on poorly controlled diabetes: a randomized controlled trial. Diabetes Educ. 2013;39:504-14. [PMID: 23640303] doi:10.1177/0145721713486837					X																															
Bond GE, Burr R, Wolf FM, Price M, McCurry SM, Teri L. The effects of a web-based intervention on the physical outcomes associated with diabetes among adults age 60 and older: a randomized trial. Diabetes Technol Ther. 2007;9:52-9					X																															
Bozzetto L, Annuzzi G, Costabile G, Costagliola L, Giorgini M, Alderisio A, et al. A CHO/fibre diet reduces and a MUFA diet increases postprandial lipaemia in type 2 diabetes: no supplementary effects of low-volume physical training. Acta Diabetol. 2014;51:385-93					X																															
Bradshaw BG, Richardson GE, Kumpfer K, Carlson J,					X																															

Stanchfield J, Overall J, et al. Determining the efficacy of a resiliency training approach in adults with type 2 diabetes. Diabetes Educ. 2007;33: 650-9																																				
Brown SA, Blozis SA, Kouzekanani K, Garcia AA, Winchell M, Hanis CL. Dosage effects of diabetes self-management education for Mexican Americans: the Starr County Border Health Initiative. Diabetes Care. 2005;28:527-32					X					X																		X								
Brown SA, Garcı´a AA, Winter M, Silva L, Brown A, Hanis CL. Integrating education, group support, and case management for diabetic Hispanics. Ethn Dis. 2011;21:20-6					X																															
Castejo AM, Caldero JL, Perez A, Millar C, McLaughlinMiddlekauff J, Sangasubana N, et al. A community-based pilot study of a diabetes pharmacist intervention in Latinos: impact on weight and hemoglobin A1c. J Health Care Poor Underserved. 2013;24:48-60.					X																															
Chan JC, Sui Y, Oldenburg B, Zhang Y, Chung HH, Goggins W, et al; JADE and PEARL Project Team. Effects of telephone-based peer support in patients with type 2 diabetes mellitus receiving integrated care: a randomized clinical trial. JAMA Intern Med. 2014;174: 972-81.					X																															
Chan LS. Chronic disease self-management in Hong Kong Chinese older adults living in the community. Dissertation Abstracts International: Section B: The Sciences and Engineering. 2012;74:4-B E.					X																															
Cheong SH, McCargar LJ, Paty BW, Tudor-Locke C, Bell RC. The First Step First Bite Program: guidance to increase physical activity and daily intake of low-glycemic index foods. J Am Diet Assoc. 2009; 109:1411-6.					X																															
Chlebowy DO, El-Mallakh P, Myers J, Kubiak N, Cloud R, Wall MP. Motivational interviewing to improve diabetes outcomes in African Americans adults with diabetes. West J Nurs Res. 2015;37:566-80.					X																															
Clark M, Hampson SE, Avery L, Simpson R. Effects of a tailored lifestyle self-management intervention in patients with type 2 diabetes. Br J Health Psychol. 2004;9:365-79.					X																		X													
Cooper H, Booth K, Gill G. A trial of empowerment-based education in type 2 diabetes—global rather than glycaemic benefits. Diabetes Res Clin Pract. 2008;82:165-71.					X																															
Corkery E, Palmer C, Foley ME, Schechter CB, Frisher L, Roman SH. Effect of a bicultural community health worker on completion of diabetes education in a Hispanic population. Diabetes Care. 1997; 20:254-7.					X							X																						X		
Cramer JS, Sibley RF, Bartlett DP, Kahn LS, Loffredo L. An adaptation of the diabetes prevention program for use with high-risk, minority patients with type 2 diabetes.					X																	X														

Diabetes Educ. 2007;33:503-8																																				
Dasgupta K, Grover SA, Da Costa D, Lowensteyn I, Yale JF, Rahme E. Impact of modified glucose target and exercise interventions on vascular risk factors. Diabetes Res Clin Pract. 2006;72:53-60.					X																															
Davis RM, Hitch AD, Salaam MM, Herman WH, Zimmer-Galler IE, Mayer-Davis EJ. Tele Health improves diabetes self-management in an under served community: diabetes Tele Care. Diabetes Care. 2010;33:1712-7.					X																															
Deakin TA, Cade JE, Williams R, Greenwood DC. Structured patient education: the diabetes X-PERT Programme makes a difference. Diabet Med. 2006;23:944-54.					X												X											X								
Dunstan DW, Daly RM, Owen N, Jolley D, Vulikh E, Shaw J, et al. Home-based resistance training is not sufficient to maintain improved glycemic control following supervised training in older individuals with type 2 diabetes. Diabetes Care. 2005;28:3-9.					X																															
Dunstan DW, Mori TA, Puddey IB, Beilin LJ, Burke V, Morton AR, et al. The independent and combined effects of aerobic exercise and dietary fish intake on serum lipids and glycemic control in NIDDM. A randomized controlled study. Diabetes Care. 1997;20: 913-21.					X																															
Eakin EG, Winkler EA, Dunstan DW, Healy GN, Owen N, Marshall AM, et al. Living well with diabetes: 24-month outcomes from a randomized trial of telephone-delivered weight loss and physical activity intervention to improve glycemic control. Diabetes Care. 2014; 37:2177-85.					X																															
Edelman D, Dolor RJ, Coffman CJ, Pereira KC, Granger BB, Lindquist JH, et al. Nurse-led behavioral management of diabetes and hypertension in community practices: a randomized trial. J Gen Intern Med. 2015;30:626-33.					X																															
Fisher L, Hessler D, Glasgow RE, Arean PA, Masharani U, Naranjo D, et al. REDEEM: a pragmatic trial to reduce diabetes distress. Diabetes Care. 2013;36:2551-8.					X																															
Foster GD, Borradaile KE, Vander Veur SS, Leh Shantz K, Dilks RJ, Goldbacher EM, et al. The effects of a commercially available weight loss program among obese patients with type 2 diabetes: a randomized study. Postgrad Med. 2009;121:113-8.					X																															
Foster GD, Wadden TA, Lagrotte CA, Vander Veur SS, Hesson LA, Homko CJ, et al. A randomized comparison of a commercially available portion-controlled weight-loss intervention with a diabetes self-management education program. Nutr Diabetes. 2013;3:e63					X																															
Frosch DL, Uy V, Ochoa S, Mangione CM. Evaluation of a behavior support intervention for patients with poorly controlled diabetes. Arch Intern Med. 2011;171:2011-7.					X																															

Gagliardino JJ, Arrechea V, Assad D, Gagliardino GG, Gonza´lez L, Lucero S, et al. Type 2 diabetes patients educated by other patients perform at least as well as patients trained by professionals. Diabetes Metab Res Rev. 2013;29:152-60.					X																															
Gagliardino JJ, Lapertosa S, Pfirter G, Villagra M, Caporale JE, Gonzalez CD, et al; PRODIACOR. Clinical, metabolic and psychological outcomes and treatment costs of a prospective randomized trial based on different educational strategies to improve diabetes care (PRODIACOR). Diabet Med .2013;30:1102-11.					X																															
Giannopoulou I, Fernhall B, Carhart R, Weinstock RS, Baynard T, Figueroa A, et al. Effects of diet and/or exercise on the adipocytokine and inflammatory cytokine levels of postmenopausal women with type 2 diabetes. Metabolism. 2005;54:866-75					X																															
Glasgow RE, Kurz D, King D, Dickman JM, Faber AJ, Halterman E, et al. Twelve-month outcomes of an Internet-based diabetes selfmanagement support program. Patient Educ Couns. 2012;87:81-92.					X																															
Glasgow RE, Nutting PA, Toobert DJ, King DK, Strycker LA, Jex M, et al. Effects of a brief computer-assisted diabetes selfmanagement intervention on dietary, biological and quality-of-life outcomes. Chronic Illn. 2006;2:27-38.					X				X				X																	X						
Glasgow RE, Strycker LA, King DK, Toobert DJ, Rahm AK, Jex M, et al. Robustness of a computer-assisted diabetes self-management intervention across patient characteristics, healthcare settings, and intervention staff. Am J Manag Care. 2006;12:137-45.					X																	X														
Goudswaard AN, Stolk RP, Zuithoff NP, de Valk HW, Rutten GE. Long-term effects of self-management education for patients with Type 2 diabetes taking maximal oral hypoglycaemic therapy: a randomized trial in primary care. Diabet Med. 2004;21:491-6.					X														X			X		X				X								
Hawkins SY. Improving glycemic control in older adults using a videophone motivational diabetes self-management intervention.Res Theory Nurs Pract. 2010;24:217-32					X																															
Hendricks LE, Hendricks RT. The effect of diabetes selfmanagement education with frequent follow-up on the health outcomes of African American men. Diabetes Educ. 2000;26:995-1002.					X																															
Hermanns N, Kulzer B, Maier B, Mahr M, Haak T. The effect of an education programme (MEDIAS 2 ICT) involving intensive insulin treatment for people with type 2 diabetes. Patient Educ Couns. 2012;86:226-32.					X																															
Hill-Briggs F, Lazo M, Peyrot M, Doswell A, Chang YT, Hill MN, et al. Effect of problem-solving-based diabetes self-management training on diabetes control in a low income patient sample. J Gen Intern Med. 2011;26:972-8.					X																															
Holmen H, Torbjørnsen A, Wahl AK, Jenum AK, Småstuen MC, Arsand E, et al. A mobile health					X																															

intervention for self-management and lifestyle change for persons with type 2 diabetes, part 2: one year results from the Norwegian randomized controlled trial RENEWING HEALTH. JMIR Mhealth Uhealth. 2014;2:e57.																																				
Holtrop JS, Hickner J, Dosh S, Noel M, Ettenhofer TL. "Sticking to it—diabetes mellitus": a pilot study of an innovative behavior change program for women with type 2 diabetes. Am J Health Educ. 2002; 33:161-6.					x																															
Huisman S, de Gucht V, Maes S, Schroevers M, Chatrou M, Haak H. Self-regulation and weight reduction in patients with type 2 diabetes: a pilot intervention study. Patient Educ Couns. 2009;75:84-90.					x																															
Izquierdo RE, Knudson PE, Meyer S, Kearns J, Ploutz-Snyder R, Weinstock RS. A comparison of diabetes education administered through telemedicine versus in person. Diabetes Care. 2003;26: 1002-7.					x																															
Johnson ST, Bell GJ, McCargar LJ, Welsh RS, Bell RC. Improved cardiovascular health following a progressive walking and dietary intervention for type 2 diabetes. Diabetes Obes Metab. 2009;11: 836-43.					x																															
Jones H, Edwards L, Vallis TM, Ruggiero L, Rossi SR, Rossi JS, et al; Diabetes Stages of Change (DiSC) Study. Changes in diabetes self-care behaviors make a difference in glycemic control: the Diabetes Stages of Change (DiSC) study. Diabetes Care. 2003;26:732-7					x																															
Kim SH, Lee SH, Ahn KY, Lee DH, Suh YJ, Cho SG, et al. Effect of lifestyle modification on serum chemerin concentration and its association with insulin sensitivity in overweight and obese adults with type 2 diabetes. Clin Endocrinol (Oxf). 2014;80:825-33.					x																															
Kim SH, Lee SJ, Kang ES, Kang S, Hur KY, Lee HJ, et al. Effects of lifestyle modification on metabolic parameters and carotid intimamedia thickness in patients with type 2 diabetes mellitus. Metabolism. 2006;55:1053-9					x																															
Koo BK, Han KA, Ahn HJ, Jung JY, Kim HC, Min KW. The effects of total energy expenditure from all levels of physical activity vs. physical activity energy expenditure from moderate-to-vigorous activity on visceral fat and insulin sensitivity in obese Type 2 diabetic women. Diabet Med. 2010;27:1088-92					x																															
Kulzer B, Hermanns N, Reinecker H, Haak T. Effects of self management training in Type 2 diabetes: a randomized, prospective trial. Diabet Med. 2007;24:415-23.					x																															
Lorig K, Ritter PL, Laurent DD, Plant K, Green M, Jernigan VB, et al. Online diabetes self-management program: a randomized study. Diabetes Care. 2010;33:1275-81.					x				x				x	x																						
Lorig K, Ritter PL, Villa FJ, Armas J. Community-based peer-led diabetes self-management: a randomized trial. Diabetes Educ. 2009; 35:641-51.					x																															

Lynch EB, Liebman R, Ventrelle J, Avery EF, Richardson D. A self-management intervention for African Americans with comorbid diabetes and hypertension: a pilot randomized controlled trial. Prev Chronic Dis. 2014;11:E90.					X																															
Mandel SE, Davis BA, Secic M. Effects of music therapy and music-assisted relaxation and imagery on health-related outcomes in diabetes education: a feasibility study. Diabetes Educ. 2013;39:568-81.					X																															
Mayer-Davis EJ, D'Antonio AM, Smith SM, Kirkner G, Levin Martin S, Parra-Medina D, et al. Pounds off with empowerment (POWER): a clinical trial of weight management strategies for black and white adults with diabetes who live in medically underserved rural communities. Am J Public Health. 2004;94:1736-42.					X	X				X		X					X					X														
McGowan P. The efficacy of diabetes patient education and selfmanagement education in type 2 diabetes. Can J Diabetes. 2011;35: 46-53					X																															
Miller CK, Kristeller JL, Headings A, Nagaraja H. Comparison of a mindful eating intervention to a diabetes self-management intervention among adults with type 2 diabetes: a randomized controlled trial. Health Educ Behav. 2014;41:145-54.					X																															
Moncrieft AE. Randomized controlled trial of a behavioral weight loss intervention for primary prevention of renal decline in type 2 diabetics. Dissertation Abstracts International: Section B: The Sciences and Engineering. 2014;75:1-B E.					X																															
Moriyama M, Nakano M, Kuroe Y, Nin K, Niitani M, Nakaya T. Efficacy of a self-management education program for people with type 2 diabetes: results of a 12 month trial. Jpn J Nurs Sci. 2009;6:51-63.					X																															
Muchmore DB, Springer J, Miller M. Self-monitoring of blood glucose in overweight type 2 diabetic patients. Acta Diabetol. 1994; 31:215-9					X																															
Murrock CJ, Higgins PA, Killion C. Dance and peer support to improve diabetes outcomes in African American women. Diabetes Educ. 2009;35:995-1003.					X																															
Nishita C, Cardazone G, Uehara DL, Tom T. Empowered diabetes management: life coaching and pharmacist counseling for employed adults with diabetes. Health Educ Behav. 2013;40:581-91					X																															
Palmas W, Findley SE, Mejia M, Batista M, Teresi J, Kong J, et al. Results of the northern Manhattan diabetes community outreach project: a randomized trial studying a community health worker intervention to improve diabetes care in Hispanic adults. Diabetes Care. 2014;37:963-9.					X																															

Plotnikoff RC, Pickering MA, Glenn N, Doze SL, ReinboldMatthews ML, McLeod LJ, et al. The effects of a supplemental, theory-based physical activity counseling intervention for adults with type 2 diabetes. J Phys Act Health. 2011;8:944-54					X																															
Prezio EA, Cheng D, Balasubramanian BA, Shuval K, Kendzor DE, Culica D. Community Diabetes Education (CoDE) for uninsured Mexican Americans: a randomized controlled trial of a culturally tailored diabetes education and management program led by a community health worker. Diabetes Res Clin Pract. 2013;100:19-28					X																															
Reaney M, Zorzo EG, Golay A, Hermanns N, Cleall S, Petzinger U, et al. Impact of Conversation Map education tools versus regular care on diabetes-related knowledge of people with type 2 diabetes: a randomized, controlled study. Diabetes Spectr. 2013;26:236-45.					X																															
Rickheim PL, Weaver TW, Flader JL, Kendall DM. Assessment of group versus individual diabetes education: a randomized study. Diabetes Care. 2002;25:269-74.					X																			X								X				
Ridgeway NA, Harvill DR, Harvill LM, Falin TM, Forester GM, Gose OD. Improved control of type 2 diabetes mellitus: a practical education/behavior modification program in a primary care clinic. South Med J. 1999;92:667-72.					X																		X											X	X	X
Rock CL, Flatt SW, Pakiz B, Taylor KS, Leone AF, Brelje K, et al. Weight loss, glycemic control, and cardiovascular disease risk factors in response to differential diet composition in a weight loss program in type 2 diabetes: a randomized controlled trial. Diabetes Care. 2014;37:1573-80.					X																															
Rosal MC, Heyden R, Mejilla R, Capelson R, Chalmers KA, Rizzo DePaoli M, et al. A virtual world versus face-to-face intervention format to promote diabetes self-management among African American women: a pilot randomized clinical trial. JMIR Res Protoc. 2014;3: e54.					X																															
Rosal MC, Ockene IS, Restrepo A, White MJ, Borg A, Olendzki B, et al. Randomized trial of a literacy-sensitive, culturally tailored diabetes self-management intervention for low-income latinos: latinos en control. Diabetes Care. 2011;34:838-44.					X		X			X																										
Ruggiero L, Moadsiri A, Butler P, Oros SM, Berbaum ML, Whitman S, et al. Supporting diabetes self-care in underserved populations: a randomized pilot study using medical assistant coaches. Diabetes Educ. 2010;36:127-31.					X					X																										
Ruggiero L, Riley BB, Hernandez R, Quinn LT, Gerber BS, Castillo A, et al. Medical assistant coaching to support diabetes self-care among low-income racial/ethnic minority populations: randomized controlled trial. West J Nurs Res. 2014;36:1052-73					X																															
Sacco WP, Malone JI, Morrison AD, Friedman A, Wells K. Effect of a brief, regular telephone intervention by					X																															

paraprofessionals for type 2 diabetes. J Behav Med. 2009;32:349-59.																																				
Sarkadi A, Rosenqvist U. Experience-based group education in Type 2 diabetes: a randomised controlled trial. Patient Educ Couns. 2004;53:291-8					X												X					X						X								
Shibayama T, Kobayashi K, Takano A, Kadowaki T, Kazuma K. Effectiveness of lifestyle counseling by certified expert nurse of Japan for non-insulin-treated diabetic outpatients: a 1-year randomized controlled trial. Diabetes Res Clin Pract. 2007;76:265-8.					X																	X		X												
Sigurdardottir AK, Benediktsson R, Jonsdottir H. Instruments to tailor care of people with type 2 diabetes. J Adv Nurs. 2009;65:2118-30.					X																															
Siminerio L, Ruppert KM, Gabbay RA. Who can provide diabetes self-management support in primary care? Findings from a randomized controlled trial. Diabetes Educ. 2013;39:705-13.					X																															
Sinclair KA, Makahi EK, Shea-Solatorio C, Yoshimura SR,<br>Townsend CK, Kaholokula JK. Outcomes from a diabetes self-management intervention for Native Hawaiians and Pacific People: Partners in Care. Ann Behav Med. 2013;45:24-32.					X																															
Smith DE, Heckemeyer CM, Kratt PP, Mason DA. Motivational interviewing to improve adherence to a behavioral weight-control program for older obese women with NIDDM. A pilot study. Diabetes Care. 1997;20:52-4.					X							X											X											X		
Sorkin DH, Mavandadi S, Rook KS, Biegler KA, Kilgore D, Dow E, et al. Dyadic collaboration in shared health behavior change: the effects of a randomized trial to test a lifestyle intervention for highrisk Latinas. Health Psychol. 2014;33:566-75.					X																															
Sperl-Hillen J, Beaton S, Fernandes O, Von Worley A, VazquezBenitez G, Hanson A, et al. Are benefits from diabetes self-management education sustained? Am J Manag Care. 2013;19:104-12					X																															
Steed L, Lankester J, Barnard M, Earle K, Hurel S, Newman S. Evaluation of the UCL diabetes self-management programme (UCLDSMP): a randomized controlled trial. J Health Psychol. 2005;10: 261-76					X																	X														
Sung K, Bae S. Effects of a regular walking exercise program on behavioral and biochemical aspects in elderly people with type II diabetes. Nurs Health Sci. 2012;14:438-45.					X																															
Tang TS, Funnell M, Sinco B, Piatt G, Palmisano G, Spencer MS, et al. Comparative effectiveness of peer leaders and community health workers in diabetes self-management support: results of a randomized controlled trial. Diabetes Care. 2014;37:1525-34					X																															
Thoolen B, De Ridder D, Bensing J, Maas C, Griffin S, Gorter K, et al. Effectiveness of a self-management intervention in patients with screen-detected type 2 diabetes. Diabetes Care. 2007;30:2832-7					X																															

Toobert DJ, Glasgow RE, Strycker LA, Barrera M Jr, Radcliffe JL, Wander RC, et al. Biologic and quality-of-life outcomes from the Mediterranean Lifestyle Program: a randomized clinical trial. Diabetes Care. 2003;26:2288-93					x												x					x														
Toobert DJ, Strycker LA, King DK, Barrera M Jr, Osuna D, Glasgow RE. Long-term outcomes from a multiple-risk-factor diabetes trial for Latinas: Viva Bien!. Transl Behav Med. 2011;1:416-426.					x					x																										
Trief P, Sandberg JG, Ploutz-Snyder R, Brittain R, Cibula D,<br>Scales K, et al. Promoting couples collaboration in type 2 diabetes: the diabetes support project pilot data. Fam Syst Health. 2011;29:253-61.					x																															
Tucker CM, Lopez MT, Campbell K, Marsiske M, Daly K, Nghiem K, et al. The effects of a culturally sensitive, empowermentfocused, community-based health promotion program on health outcomes of adults with type 2 diabetes. J Health Care Poor Underserved. 2014;25:292-307.					x																															
Utz SW, Williams IC, Jones R, Hinton I, Alexander G, Yan G,<br>et al. Culturally tailored intervention for rural African Americans with type 2 diabetes. Diabetes Educ. 2008;34:854-65					x																															
Vadstrup ES, Frølich A, Perrild H, Borg E, Røder M. Effect of a group-based rehabilitation programme on glycaemic control and cardiovascular risk factors in type 2 diabetes patients: the Copenhagen Type 2 Diabetes Rehabilitation Project. Patient Educ Couns. 2011;84:185-90.					x																															
Varney JE, Weiland TJ, Inder WJ, Jelinek GA. Effect of hospitalbased telephone coaching on glycaemic control and adherence to management guidelines in type 2 diabetes, a randomised controlled trial. Intern Med J. 2014;44:890-7.					x																															
Vazquez IM, Millen B, Bissett L, Levenson SM, Chipkin SR.<br>Buena Alimentacion, Buena Salud: a preventive nutrition intervention in Caribbean Latinos with type 2 diabetes. Am J Health Promot. 1998;13:116-9.					x					x																										
Walker EA, Shmukler C, Ullman R, Blanco E, ScollanKoliopoulus M, Cohen HW. Results of a successful telephonic intervention to improve diabetes control in urban adults: a randomized trial. Diabetes Care. 2011;34:2-7.					x					x																										
Weinger K, Beverly EA, Lee Y, Sitnokov L, Ganda OP, Caballero AE. The effect of a structured behavioral intervention on poorly controlled diabetes: a randomized controlled trial. Arch Intern Med. 2011;171:1990-9.					x																															
Welch G, Zagarins SE, Feinberg RG, Garb JL. Motivational interviewing delivered by diabetes educators: does it improve blood glucose control among poorly controlled type 2 diabetes patients? Diabetes Res					x																															

Clin Pract. 2011;91:54-60																																				
Welschen LM, van Oppen P, Bot SD, Kostense PJ, Dekker JM, Nijpels G. Effects of a cognitive behavioural treatment in patients with type 2 diabetes when added to managed care; a randomised controlled trial. J Behav Med. 2013;36:556-66.					X																															
West DS, DiLillo V, Bursac Z, Gore SA, Greene PG. Motivational interviewing improves weight loss in women with type 2 diabetes. Diabetes Care. 2007;30:1081-7.					X	X						X										X														
Wierenga ME. Life-style modification for weight control to improve diabetes health status. Patient Educ Couns. 1994;23:33-40.					X																															X
Wing RR, Bolin P, Brancati FL, Bray GA, Clark JM, Coday M, et al; Look AHEAD Research Group. Cardiovascular effects of intensive lifestyle intervention in type 2 diabetes. N Engl J Med. 2013;369:145-54.					X	X																														
Wolever RQ, Dreusicke M, Fikkan J, Hawkins TV, Yeung S, Wakefield J, et al. Integrative health coaching for patients with type 2 diabetes: a randomized clinical trial. Diabetes Educ. 2010;36:629-39.					X																															
Wolf AM, Conaway MR, Crowther JQ, Hazen KY, L Nadler J, Oneida B, et al; Improving Control with Activity and Nutrition (ICAN) Study. Translating lifestyle intervention to practice in obese patients with type 2 diabetes: Improving Control with Activity and Nutrition (ICAN) study. Diabetes Care. 2004;27:1570-6.					X	X														X		X														
Yoo JS, Lee SJ, Lee HC, Kim MJ. The effect of a comprehensive lifestyle modification program on glycemic control and body composition in patients with type 2 diabetes. Asian Nurs Res (Korean Soc Nurs Sci). 2007;1:106-15.					X																															
Yuan C, Lai CW, Chan LW, Chow M, Law HK, Ying M. The effect of diabetes self-management education on body weight, glycemic control, and other metabolic markers in patients with type 2 diabetes mellitus. J Diabetes Res. 2014;2014:789761.					X																															
Dixon JB, O'Brien PE, Playfair J et al. Adjustable gastric banding and conventional therapy for type 2 diabetes: a randomized controlled trial. JAMA 2008; 299: 316–323.						X																														
Aas AM, Bergstad I, Thorsbyt PM, Johannesen O, Solberg M, Birkelandt KI. An intensified lifestyle intervention programme may be superior to insulin treatment in poorly controlled Type 2 diabetic patients on oral hypoglycaemic agents: results of a feasibility study. Diabet Med 2005; 22: 316–322.						X																														
Luley C, Blaik A, Reschke K, Klose S, Westphal S. Weight loss in obese patients with type 2 diabetes: effects of telemonitoring plus a diet combination-the Active Body Control (ABC) Program. Diabetes Res Clin						X																														

Pract 2011; 91: 286–292.																																				
Redmon JB, Raatz SK, Reck KP et al. One-year outcome of a combination of weight loss therapies for subjects with type 2 diabetes: a randomized trial. Diabetes Care 2003; 26: 2505–2511.						X																														
Eakin EG, Reeves MM, Winkler E et al. Six-month outcomes from living well with diabetes: a randomized trial of a telephone-delivered weight loss and physical activity intervention to improve glycemic control. Ann Behav Med 2013; 46: 193–203.						X																														
Snel M, Gastaldelli A, Ouwens DM et al. Effects of adding exercise to a 16-week very low-calorie diet in obese, insulin-dependent type 2 diabetes mellitus patients. J Clin Endocrinol Metab 2012; 97: 2512–2520.						X																														
Palmer AJ, Roze S, Valentine WJ, Spinas GA, Shaw JE, et al. (2004) Intensive lifestyle changes or metformin in patients with impaired glucose tolerance: modelling the long-term health economic implications of the diabetes prevention program in Australia, France, Germany, Switzerland, and the United Kingdom. Clin Ther 26:304–321								X																												
Lindgren P, Lindström J, Tuomilehto J, Uusitupa M, Peltonen M, et al. (2007) Lifestyle intervention to prevent diabetes in men and women with impaired glucose tolerance is cost-effective. Int J Technol Assess Health Care 23: 177-183.								X																												
Tuomilehto J, Lindström J, Eriksson JG, Valle TT, Hämäläinen H, et al. (2001) Finnish Diabetes Prevention Study Group. Prevention of type 2 diabetes mellitus by changes in lifestyle among subjects with impaired glucose tolerance. N Engl J Med 344:1343-1350								X																												
Lindström J, Eriksson JG, Valle TT, Aunola S, Cepaitis Z, et al. (2003) Prevention of diabetes mellitus in subjects with impaired glucose tolerance in the Finnish Diabetes Prevention Study: results from a randomized clinical trial. J Am Soc Nephrol. 14:S108-S113								X																												
Palmer AJ, Tucker DM (2012) Cost and clinical implications of diabetes prevention in an Australian setting: a long-term modeling analysis. Prim Care Diabetes 6: 109-121.								X																												
Diabetes Prevention Program Research Group, Knowler WC, Fowler SE, Hamman RF, Christophi CA, et al. (2009) 10-year follow-up of diabetes incidence and weight loss in the Diabetes Prevention Program Outcomes Study. Lancet 374:1677-1686.								X																												
Herman WH, Hoerger TJ, Brandle M, Hicks K, Sorensen S, et al. (2005) The cost-effectiveness of lifestyle modifcation or metformin in preventing type 2 diabetes in adults with impaired glucose tolerance. Ann Intern Med 142: 323-								X																												

332.																																				
CDC Diabetes Cost-effectiveness Group (2002) Cost-effectiveness of intensive glycemic control, intensifed hypertension control, and serum cholesterol level reduction for type 2 diabetes. JAMA 287: 2542-2551								x																												
Diabetes Prevention Program Research Group1 (2012) The 10-year costeffectiveness of lifestyle intervention or metformin for diabetes prevention: an intent-to-treat analysis of the DPP/DPPOS. Diabetes Care 35: 723-730								x																												
Herman WH, Edelstein SL, Ratner RE, Montez MG, Ackermann RT, et al. (2013) Diabetes Prevention Program Research Group. Effectiveness and costeffectiveness of diabetes prevention among adherent participants. Am J Manag Care 19:194-202								x																												
Diabetes Prevention Program Research Group (2003) Within-trial costeffectiveness of lifestyle intervention or metformin for the primary prevention of type 2 diabetes. Diabetes Care 26: 2518-2523								x																												
Eddy DM1, Schlessinger L (2003) Archimedes: a trial-validated model of diabetes. Diabetes Care 26: 3093-3101.								x																												
Mason JM, Freemantle N, Gibson JM, New JP (2005) SPLINT trial. Specialist nurse-led clinics to improve control of hypertension and hyperlipidemia in diabetes: economic analysis of the SPLINT trial. Diabetes Care 28:40–46								x																												
New JP, Mason JM, Freemantle N, Teasdale S, Wong LM, et al. (2003) Specialist nurse-led intervention to treat and control hypertension and hyperlipidemia in diabetes (SPLINT): a randomized controlled trial. Diabetes Care 26: 2250-2255.								x																												
Gillett M, Dallosso HM, Dixon S, Brennan A, Carey ME, et al. (2010) Delivering the diabetes education and self-management for ongoing and newly diagnosed (DESMOND) programme for people with newly diagnosed type 2 diabetes: cost effectiveness analysis. BMJ 341:c4093								x																												
Davies MJ, Heller S, Skinner TC, Campbell MJ, Carey ME, et al. (2008) Diabetes Education and Self-Management for Ongoing and Newly Diagnosed Collaborative. Effectiveness of the diabetes education and self-management for ongoing and newly diagnosed (DESMOND) programme for people with newly diagnosed type 2 diabetes: cluster randomised controlled trial. BMJ 336:491-495								x																												
Dijkstra RF, Niessen LW, Braspenning JC, Adang E, Grol RT (2006) Patientcentred and professional-directed implementation strategies for diabetes guidelines: a cluster-randomized trial-based cost-effectiveness analysis. Diabet Med 23:164-170.								x																												
Dijkstra RF, Braspenning JC, Huijsmans Z, Akkermans RP, van Ballegooie E, et al. (2005) Introduction of								x																												

diabetes passports involving both patients and professionals to improve hospital outpatient diabetes care. Diabetes Res Clin Pract 68: 126-134.																																				
Quinn CC, Clough SS, Minor JM, Lender D, Okafor MC, Gruber-Baldini A. WellDoc mobile diabetes management randomized controlled trial: change in clinical and behavioral outcomes and patient and physician satisfaction. Diabetes Technol Ther 2008;10:160–168									x				x																							
Smith L, Weinert C. Telecommunication support for rural women with diabetes. Diabetes Educ 2000;26:645–655									x				x																							
Glasgow RE, Nutting PA, King DK, et al. Randomized effectiveness trial of a computer-assisted intervention to improve diabetes care. Diabetes Care 2005;28:33–39									x				x																							
Christian JG, Bessesen DH, Byers TE, Christian KK, Goldstein MG, Bock BC. Clinic-based support to help overweight patients with type 2 diabetes increase physical activity and lose weight. Arch Intern Med 2008;168:141–146									x				x																							
Glasgow RE, La Chance PA, Toobert DJ, Brown J, Hampson SE, Riddle MC. Long-term effects and costs of brief behavioural dietary intervention for patients with diabetes delivered from the medical office. Patient Educ Couns 1997;32:175–184									x				x																					x		
Leu MG, Norris TE, Hummel J, Isaac M, Brogan MW. A randomized, controlled trial of an automated wireless messaging system for diabetes. Diabetes Technol Ther 2005;7:710–718; discussion 719–720									x				x																							
Lim S, Kang SM, Shin H, et al. Improved glycemic control without hypoglycemia in elderly diabetic patients using the ubiquitous healthcare service, a new medical information system. Diabetes Care 2011;34:308–313									x				x																							
Quinn CC, Shardell MD, Terrin ML, Barr EA, Ballew SH, Gruber-Baldini AL. Cluster-randomized trial of a mobile phone personalized behavioral intervention for blood glucose control. Diabetes Care 2011;34:1934–1942									x				x																							
Wise PH, Dowlatshahi DC, Farrant S, Fromson S, Meadows KA. Effect of computer-based learning on diabetes knowledge and control. Diabetes Care 1986;9:504–508									x				x																					x	x	x
Yoo HJ, Park MS, Kim TN, et al. A Ubiquitous Chronic Disease Care system using cellular phones and the internet. Diabet Med 2009;26:628–635pmid.									x		x		x																							
Glasgow RE, Kurz D, King D, et al. Outcomes of minimal and moderate support versions of an internet-based diabetes self-management support program. J Gen Intern Med 2010;25:1315–1322									x				x	x																						
Glasgow RE, Boles SM, McKay HG, Feil EG, Barrera M									x				x	x						x																

Jr. The D-Net diabetes self-management program: long-term implementation, outcomes, and generalization results. Prev Med 2003;36:410–419																																				
Zhou YD, Gu W. [Computer assisted nutrition therapy for patients with type 2 diabetes]. Zhejiang Da Xue Xue Bao Yi Xue Ban 2003;32:244–248									X																											
Lo R, Lo B, Wells E, Chard M, Hathaway J. The development and evaluation of a computer-aided diabetes education program. Aust J Adv Nurs 1996;13:19–27.									X				X																							X
Anderson-Loftin W, Barnett S, Sullivan P, Bunn PS, Anderson-Loftin W, Barnett S, Sullivan P, Bunn PS, Tavakoli A: Culturally competent dietary education for southern rural African Americans with diabetes. Diabetes Educ. 2002, 28 (2): 245-257.										X																										
Banister NA, Jastrow ST, Hodges V, Loop R, Gillham MB: Diabetes self-management training program in a community clinic improves patient outcomes at modest cost. J Am Diet Assoc. 2004, 104 (5): 807-810.										X																										
Basch CE, Walker EA, Howard CJ, Shamoon H, Zybert P: The effect of health education on the rate of ophthalmic examinations among African Americans with diabetes mellitus. Am J Public Health. 1999, 89 (12): 1878-1882.										X																										
Borges WJ, Ostwald SK: Improving foot self-care behaviors with Pies Sanos. West J Nurs Res. 2008, 30 (3): 325-341.										X																										
Brown SA, Hanis CL: A community-based, culturally sensitive education and group-support intervention for Mexican Americans with NIDDM: a pilot study of efficacy. Diabetes Educ. 1995, 21 (3): 203-210.										X																										
Culica D, Walton JW, Prezio EA: CoDE: Community Diabetes Education for uninsured Mexican Americans. Proc (Bayl Univ Med Cent). 2007, 20 (2): 111-117.										X																										
Davis RM, Hitch AD, Salaam MM, Herman WH, Zimmer-Galler IE, Mayer-Davis EJ: TeleHealth Improves Diabetes Self-Management in an Underserved Community: Diabetes TeleCare. Diabetes Care. 2010, 33 (8): 1712-1717.										X																										
Ebbesson SO, Ebbesson LO, Swenson M, Kennish JM, Robbins DC: A successful diabetes prevention study in Eskimos: the Alaska Siberia project. Int J Circumpolar Health. 2005, 64 (4): 409-424.										X																										
Elshaw EB, Young EA, Saunders MJ, McGurn WC, Lopez LC: Utilizing a 24-hour dietary recall and culturally specific diabetes education in Mexican Americans with diabetes. Diabetes Educ. 1994, 20 (3): 228-235.										X		X																								X
Long JA, Jahnle EC, Richardson DM, Loewenstein G, Volpp KG: Peer mentoring and financial incentives to improve glucose control in african american veterans: a randomized trial. Ann Intern Med. 2012, 156 (6): 416-424.										X																										

Mauldon M, Melkus GD, Cagganello M, Mauldon M, Melkus GDE, Cagganello M: Tomando Control: a culturally appropriate diabetes education program for Spanish-speaking individuals with type 2 diabetes mellitus–evaluation of a pilot project. Diabetes Educ. 2006, 32 (5): 751-760.										X																										
Melkus GD, Spollett G, Jefferson V, Chyun D, Tuohy B, Robinson T, Kaisen A: A culturally competent intervention of education and care for black women with type 2 diabetes. Appl Nurs Res. 2004, 17 (1): 10-20.										X																										
Rimmer JH, Silverman K, Braunschweig C, Quinn L, Liu Y, Feasibility of a health promotion intervention for a group of predominantly African American women with type 2 diabetes. Diabetes Educ. 2002, 28 (4): 571-580.										X																										
Schillinger D, Handley M, Wang F, Hammer H, Schillinger D, Handley M, Wang F, Hammer H: Effects of self-management support on structure, process, and outcomes among vulnerable patients with diabetes: a three-arm practical clinical trial. Diabetes Care. 2009, 32 (4): 559-566.										X																										
Two FJ, Kieffer EC, Palmisano G, Anderson M, Sinco B, Janz N, Heisler M, Spencer M, Guzman R, Thompson J, Wisdom K, James SA: Racial and Ethnic Approaches to Community Health (REACH) Detroit partnership: improving diabetes-related outcomes among African American and Latino adults. Am J Public Health. 2005, 95 (9): 1552-1560.										X																										
C.T. Liu, Y.T. Yeh, T.I. Lee, Y.C. Li Observations on online services for diabetes management Diab. Care, 28 (2005), pp. 2807–2808											X																									
H.S. Kim A randomized controlled trial of a nurse short-message service by cellular phone for people with diabetes Int. J. Nurs. Stud., 44 (2007), pp. 687–692											X																									
Z. Faridi, L. Liberti, K. Shuval, V. Northrup, A. Ali, D.L. Katz Evaluating the impact of mobile telephone technology on type 2 diabetic patients' self-management: the NICHE pilot study J. Evaluation Clin. Pract., 14 (2008), pp. 465–469											X																									
K.H. Yoon, H.S. Kim A short message service by cellular phone in type 2 diabetic patients for 12 months Diab. Res. Clin. Pract., 79 (2008), pp. 256–261											X																									
C.S. Kim, S.Y. Park, J.G. Kang, S.J. Lee, S.H. Ihm, M.G. Choi, H.J. Yoo Insulin dose titration system in diabetes patients using a short messaging service automatically produced by a knowledge matrix Diab. Technol. Therapeut., 12 (2010), pp. 663–669											X																									

A.S. Shetty, S. Chamukuttan, A. Nanditha, R.K. Raj, A. Ramachandran<br>Reinforcement of adherence to prescription recommendations in Asian Indian diabetes patients using short message service (SMS) – a pilot study<br>J. Assoc. Phys. India, 59 (2011), pp. 711–714											X																									
W.I. Hussein, K. Hasan, A.A. Jaradat<br>Effectiveness of mobile phone short message service on diabetes mellitus management; the SMS-DM study<br>Diab. Res. Clin. Pract., 94 (2011), pp. e24–e26											X																									
M. Goodarzi, I. Ebrahimzadeh, A. Rabi, B. Saedipoor, M.A. Jafarabadi<br>Impact of distance education via mobile phone text messaging on knowledge, attitude, practice and self efficacy of patients with type 2 diabetes mellitus in Iran<br>J. Diab. Metabolic Disorders, 11 (2012), p. 10											X																									
S. Arora, A.L. Peters, E. Burner, C.N. Lam, M. Menchine<br>Trial to Examine Text Message-based mHealth in emergency department patients with diabetes (TExT-MED): a randomized controlled trial<br>Ann. Emerg. Med. (2013) (Epub ahead of print)											X																									
R.M. Anderson, M.M. Funnell, M.S. Arnold, P.A. Barr, G.J. Edwards, J.T. Fitzgerald<br>Assessing the cultural relevance of an education program for urban African Americans with diabetes<br>Diabetes Educ, 26 (2000), pp. 280–289												X																								
T.L. Gary, L.R. Bone, M.N. Hill, D.M. Levine, M. McGuire, C. Saudek, et al.<br>Randomized controlled trial of the effects of nurse case manager and community health worker interventions on risk factors for diabetes-related complications in urban African Americans<br>Prev Med, 37 (2003), pp. 23–32												X										X														
L.A. Jaber, H. Halapy, M. Fernet, S. Tummalapalli, H. Diwakaran<br>Evaluation of a pharmaceutical care model on diabetes management<br>Ann Pharmacother, 30 (1996), pp. 238–243												X							X										X				X			
W.L. McNabb, M.T. Quinn, L. Rosing<br>Weight loss program for inner-city black women with non-insulin-dependent diabetes mellitus: pathways<br>J Am Diet Assoc, 93 (1993), pp. 75–77												X																								
Zhou Y, Wei GU. Computer-assisted nutrition therapy for patients with type 2 diabetes. Journal of Zhejiang University (Medical Sciences) 2003;32(3):244-8.													X																							
van Bastelaar KM, Pouwer F, Cuijpers P, Riper H, Snoek FJ. Web-based depression treatment for type 1 and type 2 diabetic patients: a randomized, controlled trial. Diabetes Care 2011 Feb;34(2):320-325														X																						
Glasgow RE, Kurz D, King D, Dickman JM, Faber AJ,														X																						

Halterman E, et al. Twelve-month outcomes of an Internet-based diabetes self-management support program. Patient Educ Couns 2012 Apr;87(1):81-92																																				
Bond GE, Burr RL, Wolf FM, Feldt K. The effects of a web-based intervention on psychosocial well-being among adults aged 60 and older with diabetes: a randomized trial. Diabetes Educ 2010;36(3):446-456.														X						X																
Bond GE, Burr R, Wolf FM, Price M, McCurry SM, Teri L. The effects of a web-based intervention on the physical outcomes associated with diabetes among adults age 60 and older: a randomized trial. Diabetes Technol Ther 2007 Feb;9(1):52-59														X						X																
Bond GE, Burr R, Wolf FM, Price M, McCurry SM, Teri L. Preliminary findings of the effects of comorbidities on a web-based intervention on self-reported blood sugar readings among adults age 60 and older with diabetes. Telemed J E Health 2006 Dec;12(6):707-710.														X																						
Barrera M, Glasgow RE, McKay HG, Boles SM, Feil EG. Do Internet-based support interventions change perceptions of social support?: An experimental trial of approaches for supporting diabetes self-management. Am J Community Psychol 2002 Oct;30(5):637-654.														X																						
McKay HG, Glasgow RE, Feil EG, Boles SM, Barrera MJ. Internet-based diabetes self-management and support: Initial outcomes from the Diabetes Network project. Rehabilitation Psychology 2002;47(1):31-48.														X						X																
Lorig KR, Ritter PL, Laurent DD, Plant K. Internet-based chronic disease self-management: a randomized trial. Med Care 2006 Nov;44(11):964-971														X																						
Kim CJ, Kang DH. Utility of a Web-based intervention for individuals with type 2 diabetes: the impact on physical activity levels and glycemic control. Comput Inform Nurs 2006;24(6):337-345.														X						X																
Wangberg SC. An Internet-based diabetes self-care intervention tailored to self-efficacy. Health Educ Res 2008 Feb;23(1):170-179.														X																						
Barth R, Campbell L V, Allen S, Jupp J J, Chisholm D J. Intensive education improves knowledge, compHance, and foot problems in type 2 diabetes. Diabetic Medicine 1991; 8(2):111-117															X																			X		X
Malone J M, Snyder M, Anderson G, Bernhard V M, Holloway G A (Jnr), Bunt T J. Prevention of amputation by diabetic education. American Journal of Surgeiy 1989; 158(6): 520-523; discussion 523-524.															X																			X		X
Sun P C, Jao S E, Lin H D, Chan R C, Chou C L, Wei S H. Improving preventive foot care for diabetic patients participating in group education. Journal of American Podiatric Medicine Association 2009; 99: 295-300.															X																					
Borges W J, Ostwald S K. Improving foot selfcare behaviors with Pies Sanos. Journal of the American Podiatric Medical Association 2008; 100(2): 101-104.															X																					
Corbett CEA Randomized pilot study of improving foot care in home health patients with diabetes. The Diabetes Educator 2003; 29: 273.															X																					

Lincoln N B, Radford K A, Game F L, Jeffcoate W J. Education for secondary prevention of foot ulcers in people with diabetes: a randomised controlled trial. Diabetologia 2008; 5 1 :1954-1961.															X																					
Litzelman D K, Slemenda S W Langefeld C D, Hays L M, Welch M A, et a!. Reduction of lower extremity clinical abnormalities in patients with non-insulin-dependent diabetes mellitus. A randomized, controlled trial. Annals of Internal Medicine 1993; 119(1): 36-41.															X																					
Viswanathan V, Madhavan S, Rajasekar S, Chamukuttan S, Ambady R. Amputation prevention initiative in South India Positive impact of foot care education. Diabetes Care 2005; 28:1019-1021.															X																					
Skelly AH, Carlson JR, Leeman J, Holditch-Davis D, Soward A. Symptoms-focused management for African American women with type 2 diabetes: a pilot study. Appl Nurs Res. 2005; 18 (4): 213–220.																X					X					X										
Cabrera-Pivaral CE, Gonzalez-Perez G, Vega-Lopez MG, Rias-Merino ED: Impact of participatory education on body mass index and blood glucose in obese type-2 diabetics. Cadernos de Saude Publica. 2004, 8: 275-281.																	X																			
Clancy DE, Huang P, Okonofua E, Yeager D, Magruder KM: Group visits: promoting adherence to diabetes guidelines. Soc Gen Intern Med. 2007, 16: 620-624.																	X	X																		
Cooper HC, Booth K, Gill G: Patients' perspectives on diabetes health care education. Theory Pract. 2003, 18: 191-206.																	X										X									
Heller SR, Clarke P, Daly H, Davis I, McCulloch DK, Allison SP, et al: Group education for obese patients with type 2 diabetes: greater success at less cost. Diabet Med. 1988, 5: 552-556.																	X										X				X		X	X	X
Holtrop JS, Hickner J, Dosh S, Noel M, Ettenhofer TL: "Sticking to it – diabetes mellitus": a pilot study of an innovative behavior change program for women with type 2 diabetes. Am J Heal Educ. 2002, 33: 161-166.																	X														X				
Hornsten A, Lundman B, Stenlund H, Sandstrom H: Metabolic improvement after intervention focusing on personal understanding in type 2 diabetes. Diabetes Res Clin Pract. 2004, 68: 65-74.																	X																			
Lozano ML, Armale MJ, Tena DI, Sanchez NC: The education of type-2 diabetics: why not in groups?. Aten Primaria. 1999, 23: 485-492.																	X														X				
McKibbin CL, Patterson TL, Norman G, Patrick K, Jin H, Roesch S, et al: A lifestyle intervention for older schizophrenia patients with diabetes mellitus: a randomized controlled trial. Schizophr Res. 2006, 8: 36-44.																	X																			
Pennings-van der Eerden LM, Ripken T, van Heijst MP, Schrijvers AP: Evaluation of the impact of a patient education programme for NIDDM patients on knowledge, self-care behaviour, metabolic control and lipid profiles. Gedran en gezondheid. 1991, 19: 246-260.																	X																			

Toobert DJ, Glasgow RE, Strycker LA, Barrera M, Ritzwoller DP, Weidner G: Long-term effects of the Mediterranean lifestyle program: a randomized clinical trial for postmenopausal women with type 2 diabetes. Int J Behav Nutr Phys Act. 2007, 82: 1.																	X																			
Trento M, Passera P, Tomalino M, Pagnozzi F, Pomero F, Vaccari P, et al: Therapeutic group education in the follow-up of patients with non-insulin treated, non-insulin dependent diabetes mellitus. Diabetes Nutr Metab Clin Exp. 1998, 11: 212-216.																	X															X			X	X
Trento M, Passera P, Tomalino M, Bajardi M, Pomero F, Allione A, et al: Group visits improve metabolic control in type 2 diabetes: a 2-year follow-up. Diabetes Care. 2001, 24: 995-1000. 10.2337/diacare.24.6.995.																	X	X									X				X				
Trento M, Passera P, Borgo E, Tomalino M, Bajardi M, Cavallo F, et al: A 5-year randomized controlled study of learning, problem solving ability, and quality of life modifications in people with type 2 diabetes managed by group care. Diabetes Care. 2004, 112: 670-675.																	X	X									X								
Wattana C, Srisuphan W, Pothiban L, Upchurch SL: Effects of a diabetes self-management program on glycemic control, coronary heart disease risk, and quality of life among Thai patients with type 2 diabetes. Nurs Heal Sci. 2007, 9: 135-141.																	X					X														
Zapotoczky H, Semlitsch B, Herzog G, Bahadori B, Siebenhofer A, Pieber TR, et al: A controlled study of weight reduction in type 2 diabetics treated by two reinforcers. Int J Behav Med. 2001, 8: 42-49.																	X															X				
Deakin TA, Cade JE, Williams DDR, Greenwood DC: Empowered patients:better diabetes control, greater freedom to eat, no weight gain!. Diabetologia. 2003, 46 (suppl 2): A90.																	X										X				X				
Clancy D, Cope D, Magruder K, Peng H, Wolfman T. Evaluating Concordance to American Diabetes Association Standards of Care for Type 2 Diabetes Through Group Visits in an Uninsured or Inadequately Insured Patient Population. Diabetes Care. 2003; 26(7): 2032-2036.																		X																		
Kirsh S, Watts S, Pascuzzi K, O'Day M, Davidson D, Aron D, et al. Shared medical appointments based on the chronic care model: a quality improvement project to address the challenges of patients with diabetes with high cardiovascular risk. Qual Saf Health Care. 2007; 16(5): 349-353.																		X																		
Edelman D, Fredrickson S, Melnyk S, Coffman C, Jeffreys A, Weinberger M, et al. Medical clinics versus usual care for patients with both diabetes and hypertension: a randomized trial. Ann Intern Med. 2010; 152(11): 689-696.																		X																		
Taveira T, Friedmann P, Cohen L, Dooley A, Khatana S, Wu W, et al. Pharmacist-led group medical appointment model in type 2 diabetes. Diabetes Educ. 2010; 36(1): 109-117.																		X																		
Trento M, Basile M, Borgo E, Grassi G, Scuntero P,																		X																		

Porta M, et al. A randomised controlled clinical trial of nurse-, dietitian- and pedagogist-led Group Care for the management of Type 2 diabetes. J Endocrinol Invest. 2008; 31(11): 1038-1042.																																				
Pieber T, Holler A, Siebenhofer A, Brunner G, Semlitsch B, Krejs G, et al. Evaluation of a structured teaching and treatment programme for type 2 diabetes in general practice in a rural area of Austria. Diabet Med. 1995; 12(4): 349-354.																	X				X															
Trento M, Passera, P, Tomalino M, Pagnozzi F, Pomero F, Vaccari P, Bajardi M, Molinatti GM, Porta M. Theraputic group education in the follow-up of patients with non-insulin treated, non-insulin dependent diabetes mellitus. Diabetes Nutr Metab. 1998; 2(3):212-216.																	X																			
Trento M, Gamba S, Gentile L, Grassi G, Miselli V, Porta M, et al. Rethink Organization to iMprove Education and Outcomes (ROMEO): a multicenter randomized trial of lifestyle intervention by group care to manage type 2 diabetes. Diabetes Care. 2010; 33(4): 745-747.																	X																			
Clancy D, Brown S, Magruder K, Huang P. Group visits in medically and economically disadvantaged patients with type 2 diabetes and their relationships to clinical outcomes. Top Health Inf Manage. 2003; 24(1): 8-14.																	X												X							
Bray P, Thompson D, Wynn J, Cummings D, Whetstone L. Confronting disparities in diabetes care: the clinical effectiveness of redesigning care management for minority patients in rural primary care practices. J Rural Health. 2005; 21(4): 317-321.																	X																			
Culhane-Pera K, Peterson K, Crain A, Center B, Lee M, Xiong T, et al. Group visits for Hmong adults with type 2 diabetes mellitus: a pre-post analysis. J Health Care Poor Underserved. 2005; 16(2): 315-327.																	X																			
Mullan RJ, Montori VM, Shah ND, Christianson TJH, Bryant SC, Guyatt GH, et al. The diabetes mellitus medication choice decision aid: a randomized trial. Arch Intern Med. 2009;169(17):15608																		X																		
Fornos JA, Andres NF, Andres JC, Guerra MM, Egea B. A pharmacotherapy follow-up program in patients with type-2 diabetes in community pharmacies in Spain. Pharm World Sci. 2006;28(2):65-72.																		X																		
Al Mazroui NR, Kamal MM, Ghabash NM, Yacout TA, Kole PL, McElnay JC. Influence of pharmaceutical care on health outcomes in patients with Type 2 diabetes mellitus. Br J Clin Pharmacol. 2009;67(5):547-57.																		X																		
Farmer AJ, Wade AN, French DP, Simon J, Yudkin P, Gray A, et al. Blood glucose selfmonitoring in type 2 diabetes: a randomised controlled trial. Health Technol Assess. 2009;13(15):iii																		X																		
Kulzer B, Hermanns N, Reinecker H, Haak T. Effects of self-management training in Type 2 diabetes: a randomized, prospective trial. Diabet Med. 2007;24(4):415-23.																		X																		
H.-S. Kwon, J.-H. Cho, H.-S. Kim, B.-R. Song, S.-H. Ko, J.-M. Lee, et al.																			X																	

Establishment of blood glucose monitoring system using the Internet<br>Diabetes Care, 27 (2) (2004), pp. 478–483																																				
J.-H. Cho, S.-A. Chang, H.-S. Kwon, Y.-H. Choi, S.-H. Ko, S.-D. Moon, et al.<br>Long-term effect of the Internet-based glucose monitoring system on HbA1c reduction and glucose stability<br>Diabetes Care, 29 (12) (2006), pp. 2625–2631																				X																
G.T. McMahon, H.E. Gomes, S. Hickson Hohne, T.M.-J. Hu, B.A. Levine, P.R. Conlin<br>Web-based care management in patients with poorly controlled diabetes<br>Diabetes Care, 28 (7) (2005), pp. 1624–1629																				X																
G.E. Bond, R. Burr, F.M. Wolf, M. Price, S.M. McCurry, L. Teri<br>Preliminary findings of the fffects of comorbidities on a web-based intervention on self-reported blood sugar readings among adults age 60 and older with diabetes<br>Telemed. J. e-Health, 12 (6) (2006), pp. 707–710																				X																
H.-S. Kim, H.-Y. Son<br>Technological intervention for obese patients with type 2 diabetes<br>Appl. Nurs. Res., 21 (2) (2008), pp. 84–89																				X																
S.-I. Kim, H.-S. Kim<br>Effectiveness of mobile and internet intervention in patients with obese type 2 diabetes<br>Int. J. Med. Inf., 77 (6) (2008), pp. 399–404																				X																
J.D. Ralston, I.B. Hirsch, J. Hoath, M. Mullen, A. Cheadle, H.I. Goldberg<br>Web-based collaborative care for type 2 diabetes<br>Diabetes Care, 32 (2) (2009), pp. 234–239																				X																
J.-H. Noh, Y.-J. Cho, H.-W. Nam, J.-H. Kim, D.-J. Kim, H.-S. Yoo, et al.<br>Web-based comprehensive information system for self-management of diabetes mellitus<br>Diabetes Technol. Ther., 12 (5) (2010), pp. 333–337																				X																
E.G. Fell, R.E. Glasgow, S. Boles, H.G. McKay<br>Who participates in Internet-based self-management programs? A study among novice computer users in a primary care setting<br>Diabetes Educ., 26 (5) (2000), pp. 806–811																				X																
H.G. McKay, D. King, E.G. Eakin, J.R. Seeley, R.E. Glasgow<br>The Diabetes Network Internet-based physical activity intervention: a randomized pilot study<br>Diabetes Care, 24 (8) (2001), pp. 1328–1334																				X			X													
C. Richardson, K. Mehari, L. McIntyre, A. Janney, L. Fortlage, A. Sen, et al.																				X																

A randomized trial comparing structured and lifestyle goals in an internet-mediated walking program for people with type 2 diabetes<br>Int. J. Behav. Nutr. Phys. Act., 4 (1) (2007), p. 59																																				
T. Liebreich, R. Plotnikoff, K. Courneya, N. Boule<br>Diabetes NetPLAY: a physical activity website and linked email counselling randomized intervention for individuals with type 2 diabetes<br>Int. J. Behav. Nutr. Phys. Act., 6 (1) (2009), p. 18																				X																
A. Holbrook, L. Thabane, K. Keshavjee, L. Dolovich, B. Bernstein, D. Chan, et al.<br>Individualized electronic decision support and reminders to improve diabetes care in the community: COMPETE II randomized trial<br>CMAJ, 181 (1–2) (2009), pp. 37–44																				X																
Keyserling TC, Samuel-Hodge CD, Ammerman AS, Ainsworth BE, Henriquez-Roldan CF, Elasy TA et al. A randomised trial of an intervention to improve self-care behaviours of African-American women with type 2 diabetes. Diabetes Care 2002; 25: 1576–1583.																					X									X						
K. Samaras, S. Ashwell, A.M. Mackintosh, A.C. Fleury, L.V. Campbell, D.J. Chisholm<br>Will older sedentary people with non-insulin-dependent diabetes mellitus start exercising? A health promotion model<br>Diabetes Res Clin Pract, 37 (1997), pp. 121–128																						X								X						
C.K. Miller, L. Edwards, G. Kissling, L. Sanville<br>Nutrition education improves metabolic outcomes among older adults with diabetes mellitus: results from a randomized controlled trial<br>Prev Med, 34 (2002), pp. 252–259																						X														
P. Gaede, M. Beck, P. Vedel, O. Pedersen<br>Limited impact of lifestyle education in patients with type 2 diabetes mellitus and microalbuminuria: results from a randomized intervention study<br>Diabetic Med, 18 (2001), pp. 104–108																						X														
C. Di Loreto, C. Fanelli, P. Lucidi, G. Murdolo, C.A. De, N. Parlanti, F. Santeusanio, P. Brunetti, P. De Feo<br>Validation of a counseling strategy to promote the adoption and the maintenance of physical activity by type 2 diabetic subjects<br>Diabetes Care, 26 (2003), pp. 404–408																						X														
R.A. Gabbay, I. Lendel, T.M. Saleem, G. Shaeffer, A.M. Adelman, D.T. Mauger, M. Collins, R.C. Polomano<br>Nurse case management improves blood pressure, emotional distress and diabetes complication screening<br>Diabetes Res Clin Pract, 71 (2006), pp. 28–35																						X														
S.H. Ko, K.H. Song, S.R. Kim, J.M. Lee, J.S. Kim, J.H. Shin<br>Long-term effects of a structured intensive diabetes education programme (SIDEP) in patients with type 2																						X						X								

diabetes mellitus—a 4-year follow-up study<br>Diabetic Med, 24 (2007), pp. 55–62																																				
P. Kronsbein, V. Jorgens, I. Muhlhauser, V. Scholz, A. Venhaus, M. Berger<br>Evaluation of a structured treatment and teaching programme on non-insulin-dependent diabetes<br>Lancet, 2 (1988), pp. 1407–1411																					X						X				X				
A.L. Estey, M.H. Tan, K. Mann<br>Follow-up intervention: its effect on compliance behavior to a diabetes regimen<br>Diabetes Educ, 16 (1990), pp. 291–295																					X												X		
M.J. Franz, A. Monk, B. Barry, K. McClain, T. Weaver, N. Cooper<br>Effectiveness of medical nutrition therapy provided by dieticians in the management of non-insulin-dependent diabetes mellitus: a randomized, controlled clinical trial<br>J Am Diet Assoc, 95 (1995), pp. 1009–1017																					X														
M.I. Uusitupa<br>Early lifestyle intervention in patients with non-insulin-dependent diabetes mellitus and impaired glucose tolerance<br>Ann Med, 28 (1996), pp. 445–449																					X								X						X
B.P. Krier, R.D. Parker, D. Grayson, G. Byrd<br>Effect of diabetes education on glucose control<br>J La State Med Soc, 151 (1999), pp. 86–92																					X							X				X			
H. Sone, A. Katagiri, S. Ishibashi, R. Abe, Y. Saito, T. Murase, R. Abe, Y. Ohashi, Y. Akanuma, N. Yamada, Japan Diabetes Complication Study Group<br>Effects of lifestyle modifications on patients with type 2 diabetes: the Japan Diabetes Complications Study (JDCS) study design, baseline analysis and three year-interim report<br>Horm Metab Res, 34 (2002), pp. 509–515																					X														
C.B. Taylor, N.H. Miller, K.R. Reilly, G. Greenwald, D. Cunning, A. Deeter, L. Abascal<br>Evaluation of nurse-care management system to improve outcomes in patients with complicated diabetes<br>Diabetes Care, 26 (2003), pp. 1058–1063																					X														
J.A. Oh, H.S. Kim, K.H. Yoon, E.S. Choi<br>A telephone-delivered intervention to improve glycemic control in type 2 diabetic patients<br>Yonsei Med J, 44 (2003), pp. 1–8																					X														
R. Whittemore, G.D. Melkus, A. Sullivan, M. Grey<br>A nurse-coaching intervention for women with type 2 diabetes<br>Diabetes Educ, 30 (2004), pp. 795–804																					X	X	X												
M. Maislos, D. Weisman<br>Multidisciplinary approach to patients with poorly																					X														

controlled type 2 diabetes mellitus: a prospective, randomized study<br>Acta Diabetol, 41 (2004), pp. 44–48																																				
R. Rachiman, I. Slavacheski, M. Berla, R. Frommer-Shapira, M. Ravid<br>Treatment of high-risk patients with diabetes: motivation and teaching intervention: a randomized, prospective 8-year follow-up study<br>J Am Soc Nephrol, 16 (2005), pp. S22–S26																						X														
R.J. Young, J. Taylor, T. Friede, S. Hollis, J.M. Mason, P. Lee, E. Burns, A.F. Long, T. Gambling, J.P. New, J.M. Gibson<br>Pro-active call center treatment support (PACCTS) to improve glucose control in type 2 diabetes: a randomized controlled trial<br>Diabetes Care, 28 (2005), pp. 278–282																						X														
M.S. Song, H.S. Kim<br>Effect of the diabetes outpatient intensive management programme on glycaemic control for type 2 diabetic patients<br>J Clin Nurs, 16 (2007), pp. 1367–1373																						X														
Z.W. Ma, M.X. Li, C.S. Wang<br>Effects of comprehensive psycho-intervention on life quality and carbohydrate metabolism in patients with type 2 diabetes mellitus<br>Zhongguo Linchuang Kangfu, 10 (2006), pp. 15–17																							X													
L. Zhao, Y.J. Sun, Q.G. Liu, M.M. Huan, J. Wu, L. Zhou, H.J. Shen, C. Jiang<br>Intervention effects of music relaxation therapy on the quality of life in patients with diabetic retinopathy<br>Zhongguo Linchuang Kangfu, 9 (2005), pp. 50–53																							X													
Z. Zeng, L. Ma, L. Tang, G. Luo, H. Zhou, L. Zhou<br>Comprehensive intervention on diabetes mellitus<br>Chin Ment Health J, 17 (2003), pp. 253–255																							X													
A. Kirk, N. Mutrie, P. MacIntyre, M. Fisher<br>Effects of a 12-month activity counselling intervention on glycaemic control and on the status of cardiovascular risk factors in people with type 2 diabetes<br>Diabetologia, 47 (2004), pp. 821–832																							X													
F.L. Huang, J.H. Ye, F. Huang<br>Effect of mental intervention on type 2 diabetics: randomized controlled study<br>Zhongguo Linchuang Kangfu, 8 (2004), pp. 5795–5798																							X													
R.A. McGinnis, A. McGrady, S.A. Cox, K.A. Grower-Dowling<br>Biofeedback-assisted relaxation in type 2 diabetes<br>Diabetes Care, 28 (2005), pp. 2145–2149																							X													
M. Siebolds, O. Gaedeke, U. Schwedes<br>Self-monitoring of blood glucose-psychological aspects																							X													

relevant to changes in hba1c in type 2 diabetic patients treated with diet or diet plus oral antidiabetic medication<br>Patient Educ Couns, 62 (2006), pp. 104–110																																				
R. Jackson, K. Asimakopoulou, A. Scammell<br>Assessment of the transtheoretical model as used by dietitians in promoting physical activity in people with type 2 diabetes<br>J Hum Nutr Diet, 20 (2007), pp. 27–36																							X													
P.J. Lustman, L.S. Griffith, K.E. Freedland, S.S. Kissel, R.E. Clouse<br>Cognitive behavior therapy for depression in type 2 diabetes mellitus: a randomized, controlled trial<br>Ann Intern Med, 129 (1998), pp. 613–621																							X													
C.A. Heitzmann, R.M. Kaplan, D.K. Wilson, J. Sandler<br>Sex differences in weight loss among adults with type ii diabetes mellitus<br>J Behav Med, 10 (1987), pp. 197–211																							X													X
L.V. Campbell, R. Barth, J.K. Gosper, J.J. Jupp, L.A. Simons, D.J. Chisholm<br>Impact of intensive educational approach to dietary change in NIDDM<br>Diabetes Care, 13 (1990), pp. 841–847																							X							X						X
J.D. Lane, C.C. McCaskill, S.L. Ross, M.N. Feinglos, R.S. Surwit<br>Relaxation training for NIDDM: predicting who may benefit<br>Diabetes Care, 16 (1993), pp. 1087–1094																							X											X		
E.M. Campbell, S. Redman, P.S. Moffitt, R.W. Sanson-Fisher<br>The relative effectiveness of educational and behavioral instruction programs for patients with NIDDM: a randomized trial<br>Diabetes Educator, 22 (1996), pp. 379–386																							X	X				X						X	X	X
J. Kenardy, M. Mensch, K. Bowen, B. Green, J. Walton<br>Group therapy for binge eating in type 2 diabetes: a randomized trial<br>Diabet Med, 19 (2002), pp. 234–239																							X													
S.W. Rabkin, E. Boyko, A. Wilson, D.A. Streja<br>A randomized clinical trial comparing behavior modification and individual counseling in the nutritional therapy of non-insulin-dependent diabetes mellitus: comparison of the effect on blood sugar, body weight, and serum lipids<br>Diabetes Care, 6 (1983), pp. 50–56																							X													
R.R. Wing, L.H. Epstein, M.P. Nowalk, R. Koeske, S. Hagg<br>Behavior change, weight loss, and physiological improvements in type ii diabetic patients<br>J Consult Clin Psych, 53 (1985), pp. 111–122																							X											X		X

S. Hartwell, R. Kaplan, J. Wallace<br>Comparison of behavioral interventions for control of type ii diabetes mellitus<br>Behav Ther, 17 (1986), pp. 447–461																							X							X						X
N. White, J. Carnahan, C.A. Nugent, T. Iwaoka, M.A. Dodson<br>Management of obese patients with diabetes mellitus: comparison of advice education with group management<br>Diabetes Care, 9 (1986), pp. 490–496																							X						X				X	X	X	X
F. Rodriguez, M. Alvarez<br>Modificacion de conducta y mejora en el cumplimiento del tratamiento en diabeticos tipo ii<br>Rev Esp Ter Comportamiento, 5 (1987), pp. 223–247																							X													
R.R. Wing, M.D. Marcus, L.H. Epstein, A. Jawad<br>A “Family-based” approach to the treatment of obese type ii diabetic patients<br>J Consult Clin Psych, 59 (1991), pp. 156–162																							X											X		
G.A. D’Eramo-Melkus, J. Wylie-Rosett, J.A. Hagan<br>Metabolic impact of education in NIDDM<br>Diabetes Care, 15 (1992), pp. 864–869																							X											X	X	X
S. Boehm, E.A. Schlenk, E. Raleigh, D. Ronis<br>Behavioral analysis and behavioral strategies to improve self-management of type ii diabetes<br>Clin Nurs Res, 2 (1993), pp. 327–344																							X											X		X
J.E. Aikens, T.A. Kiolbasa, R. Sobel<br>Psychological predictors of glycemic change with relaxation training in non-insulin-dependent diabetes mellitus<br>Psychother Psychosom, 66 (1997), pp. 302–306																							X											X		
J. Henry, P. Wilson, D. Bruce, D. Chisholm, P. Rawling<br>Cognitive-behavioural stress management for patients with non-insulin dependent diabetes mellitus<br>Psychol Health Med, 2 (1997), pp. 109–118																							X											X		
S.L. Jablon, B.D. Naliboff, S.L. Gilmore, M.J. Rosenthal<br>Effects of relaxation training on glucose tolerance and diabetic control in type ii diabetes<br>Appl Psychophys Biof, 22 (1997), pp. 155–169																							X													
X. Huang, L. Song, T. Li<br>The effect of social support on type ii diabetes with depression<br>Chin J Clin Psychol, 9 (2001), pp. 187–189																							X													
X. Zhu, Y. Gong, S. Yao<br>The effect of biofeedback assisted relaxation training on cytokines in patients with type ii diabetes<br>Chin J Clin Psychol, 9 (2001), pp. 170–172																							X													
R.S. Surwit, M.A. van Tilburg, N. Zucker, C.C. McCaskill, P. Parekh, M.N. Feinglos, C.L. Edwards, P. Williams,																							X													

J.D. Lane<br>Stress management improves long-term glycemic control in type 2 diabetes<br>Diabetes Care, 25 (2002), pp. 30–34																																				
T. Tsujiuchi, H. Kumano, K. Yoshiuchi, D. He, Y. Tsujiuchi, T. Kuboki, H. Suematsu, K. Hirao<br>The effect of qi-gong relaxation exercise on the control of type 2 diabetes mellitus: a randomized controlled trial<br>Diabetes Care, 25 (2002), pp. 241–242																							X													
Hiss RG, Lou Gillard M, Armbruster BA, McClure LA. Comprehensive evaluation of community-based diabetic patients: Effect of feedback to patients and their physicians: A randomized controlled trial. Diabetes Care 2001;24(4):690-4.																								X												
Dalmau Llorca MR, Garcia Bernal G, Aguilar Martin C, Palau Galindo A. [Group versus individual education for type-2 diabetes patients]. Atencion Primaria 2003;32(1):36-41.																								X												
Deakin T, Cade JE, Williams DDR, Greenwood DC. EXpert patient education versus routine treatment: process evaluation. Diabetologia 2002;45(Suppl 2): 317.																												X								
Brown SA, Hanis CL. Culturally competent diabetes education for Mexican Americans: the Starr County Study. Diabetes Educ 1999;25:226–36																												X								X
Cooper H, Booth K, Gill G. A randomised controlled study of education for people with type 2 diabetes. Liverpool: University of Liverpool; 2002.																												X								
Cooper H, Booth K, Gill G. Using combined research methods for exploring diabetes patient education. Patient Educ Couns 2003;51:45–52.																												X								
Raz I, Soskolne V, Stein P. Influence of small-group education sessions on glucose homeostasis in NIDDM. Diabetes Care 1988;11:67–71.																												X						X	X	X
Domenech MI, Assad D, Mazzei ME, Kronsbein P, Gagliardino JJ. Evaluation of the effectiveness of an ambulatory teaching/treatment programme for noninsulin dependent (type 2) diabetic patients. Acta Diabetol 1995;32:143–7.																												X			X	X				
Keyserling TC, Ammerman AS, Samuel-Hodge CD, Ingram AF, Skelly AH, et al. A diabetes management program for African American woment with type 2 diabetes. The diabetes educator 2000;26(5):796-805.																										X				X						
Vyas A, Haidery AZ, Wiles PG, Gill S, Roberts C, Cruikshank JK. A pilot randomised trial in primary care to investigate and improve knowledge, awareness and self-management among South Asians with diabetes in Manchester. Diabet Med 2003; 20: 1022–1026.																											X									
Anon (reporting study by Kerr L et al .). Tailored approach overcomes cultural barriers. Pharm J 2004; 273: 14.																											X									

Samanta A, Campbell JE, Spalding DL, Panja KK, Neogi SK, Burden AC. Dietary habits of Asian diabetics in a general practice clinic. Hum Nutr Appl Nutr 1987; 41A: 160–163.																											X									
Wallace P, Awan A, Talbot J. Health Advice for Asian women with diabetes. Prof Nurse 1996; 11: 794–796.																											X									
Simmons D. Diabetes self help facilitated by local diabetes research: The Coventry Asian Diabetes Support Group. Diabet Med 1992; 9: 866–869.																											X									
A. Mease, W.L. Whitlock, A. Brown, K. Moore, H. Pavliscsak, A. Dingbaum, et al. Telemedicine improved diabetic management Mil. Med., 165 (8) (2000), pp. 579–584																													X				X			
J.D. Piette, M. Weinberger, F.B. Kraemer, S.J. McPhee Impact of automated calls with nurse follow-up on diabetes treatment outcomes in a department of veterans affairs Health Care System Diab. Care, 24 (2) (2001), pp. 202–208																													X				X			
R. Rachmani, Z. Levi, I. Slavachevski, M. Avin, M. Ravid Teaching patients to monitor their risk factors retards the progression of vascular complications in high-risk patients with Type 2 diabetes mellitus. A randomized prospective study Diab. Med., 19 (2002), pp. 385–392																													X				X			
E.A. Coast-Senior, B.A. Kroner, C.L. Kelley, L.E. Trilli Management of patients with type 2 diabetes by pharmacists in primary care clinics The Ann. Pharmacother., 32 (1998), pp. 636–641																													X				X			
Y.-D. Jiang, L.-M. Chuang, H.-P. Wu, S.-J. Shiau, C.-H. Wang, Y.-J. Lee, et al. Assessment of the function and effect of diabetes education programs in Taiwan Diab. Res. Clin. Pract., 46 (1999), pp. 177–182																													X				X			
de Bont AJ, Baker IA, St Leger AS, Sweetnam PM, Wragg KG, Stephens SM, Hayes TM. A randomised controlled trial of the effect of low fat diet advice on dietary response in insulin independent diabetic women. Diabetologia 1981;21(6):529-33.																														X					X	
Gallagher A, Henderson W, Abraira C. Dietary patterns and metabolic control in diabetic diets: a prospective study of 51 outpatient men on unmeasured and exchange diets. Journal of the American College of Nutrition 1987;6(6):525-32																														X						
Gallagher AM, Abraira C, Henderson WG. A four-year prospective trial of unmeasured diet in lean diabetic adults. Diabetes Care 1984;7(6):557-65.																														X						
Glasgow RE, La Chance PA, Toobert DJ, Brown J, Hampson SE, Riddle MC. Long term effects and costs of brief behavioural dietary intervention for patients with diabetes delivered from the medical office. Patient																														X						

Education and Counseling 1997;32(3):175-184.																																				
Glasgow RE, Toobert DJ, Hampson SE. Effects of a Brief Office-Based Intervention to Facilitate Diabetes Dietary Self-Management. Diabetes Care 1996;19(8):835-42.																													X					X	X
Hockaday TDR, Hockaday JM, Mann JI, Turner RC. Prospective comparison of modified-fat-high-carbohydrate with standard low-carbohydrate dietary advice in the treatment of diabetes: a one year follow-up study. British Journal of Nutrition 1978;39:357-62.																													X						
Hockaday TDR, Pandher KS, Bron A, et al. Progression of established retinopathy is unrelated to glycosylated hemoglobin in non-insulin-dependent diabetes. Transplantation Proceedings 1986;18(6):1574-1575.																													X						
Howard-Williams J, Hillson RM, Bron A, Awdry P, Mann JI, Hockaday TDR. Retinopathy is associated with higher glycaemia in maturity-onset type diabetes. Diabetologia 1984;27:198-202.																													X						
Howard-Williams J, Patel P, Jelfs R, Carter RD, Awdry P, Bron A, et al. Polyunsaturated fatty acids and diabetic retinopathy. British Journal of Ophthalmology 1985;69(1):15-8.																													X						
Lopez-Espinoza I, Howard-Williams J, Mann JI, Carter RD, Hockaday TD. Fatty acid composition of platelet phospholipids in non-insulin-dependent diabetics randomized for dietary advice. British Journal of Nutrition 1984;52(1):41-7.																													X						
Mann JI, Hockaday TD, Hockaday JM, Turner RC. A prospective study of modified-fat and low-carbohydrate dietary advice in the treatment of maturity-onset diabetes [proceedings]. Proceedings of the Nutrition Society 1976;35(2):72A-73A.																													X						
Kaplan RM, Atkins CJ, Wilson DK. The cost-utility of diet and exercise interventions in non-insulin-dependent diabetes mellitus. Health Promotion 1987;2(4):331-340.																													X						
Kaplan RM, Hartwell SL, Wilson DK, Wallace JP. Effects of diet and exercise interventions on control and quality of life in non-insulin-dependent diabetes mellitus. Journal of General Internal Medicine 1987;2(4):220-8.																													X				X		X
Ligtenberg PC, Hoekstra JB, Bol E, Zonderland ML, Erkelens DW. Effects of physical training on metabolic control in elderly type 2 diabetes mellitus patients. Clinical Science 1997;93(2):127-35.																													X				X		
Milne RM, Mann JI, Chisholm AW, Williams SM. Long-term comparison of three dietary prescriptions in the treatment of NIDDM. Diabetes Care 1994;17(1):74-80.																													X						
Pascale RW, Wing RR, Butler BA, Mullen M, Bononi P. Effects of a behavioral weight loss program stressing calorie restriction versus calorie plus fat restriction in obese individuals with NIDDM or a family history of diabetes. Diabetes Care 1995;18(9):1241-8.																													X						
Tsihlias EB, Gibbs AL, McBurney MI, Wolever TMS. Comparison of high- and low-glycemic-index breakfast cereals with monounsaturated fat in the long-term																													X						

dietary management of type 2 diabetes. American Journal of Clinical Nutrition 2000;72(2):439-49																																				
Wolever TM, Schrade KB, Vogt JA, Tsihlias EB, McBurney MI. Do colonic short-chain fatty acids contribute to the long-term adaptation of blood lipids in subjects with type 2 diabetes consuming a high-fiber diet?. American Journal of Clinical Nutrition 2002;75(6):1023-30.																														X						
Laitinen J, Uusitupa M, Ahola I, Laakso M, Siitonen O. Metabolic and dietary variables associated with glycaemic control in patients with recently diagnosed Type II diabetes mellitus. Diabetes, Nutrition & Metabolism - Clinical & Experimental 1994;7(2):77-87.																														X						
Laitinen J, Uusitupa M, Ahola I, Siitonen O. Metabolic and dietary determinants of serum lipids in obese patients with recently diagnosed non-insulin-dependent diabetes. Annals of Medicine 1994;26(2):119-24.																														X						
Vanninen E, Laitinen J, Uusitupa M. Physical activity and fibrinogen concentration in newly diagnosed NIDDM. Diabetes Care 1994;17(9):1031-8.																														X						
Vanninen E, Uusitupa M, Lansimies E, Siitonen O, Laitinen J. Effect of metabolic control on autonomic function in obese patients with newly diagnosed type 2 diabetes. Diabetic Medicine 1993;10(1):66-73.																														X						
Vanninen E, Uusitupa M, Siitonen O, Laitinen J, Lansimies E. Habitual physical activity, aerobic capacity and metaboic control in patients with newly-diagnosed Type 2 (non-insulin-dependent) diabetes mellitus: Effect of 1-year diet and exercise intervention. Diabetologia 1992;35(4):340-346.																														X				X		
Wing RR, Epstein LH, Paternostro-Bayles M, Kriska A, Nowalk MP, Gooding W. Exercise in a behavioural weight control programme for obese patients with Type 2 (non-insulin-dependent) diabetes. Diabetologia 1988;31(12):902-9.																														X						
Marcus MD, Wing RR, Guare J, Blair EH, Jawad A. Lifetime prevalence of major depression and its effect on treatment outcome in obese type II diabetic patients. Diabetes Care 1992;15(2):253-5.																														X						
Wing RR, Marcus MD, Salata R, Epstein LH, Miaskiewicz S, Blair EH. Effects of a very-low-calorie diet on long-term glycemic control in obese type 2 diabetic subjects. see comments. Archives of Internal Medicine 1991;151(7):1334-40.																														X						
Wing RR, Blair E, Marcus M, Epstein LH, Harvey J. Year-long weight loss treatment for obese patients with type II diabetes: does including an intermittent very-low-calorie diet improve outcome?. American Journal of Medicine 1994;97(4):354-62.																														X						
Kronsbein P, Miihlhauser I,Venhaus A,Jorgens V, ScholzV. Berger M (1988).Evaluation of a structured treatment and teaching programme on noninsulin-dependent diabetes. Lancet 2: 1407-11.																															X					
Pieber TR, Brunner GA, Schnedl WJ, Schattenberg S,																																X				

Kaufmenn P, Krejs GJ. Evaluation of a structured outpatient group education program for intensive insulin therapy. Diabetes Care 1995;18:625-30.																																				
Canga N, De Irala J, Vara E, Duaso MJ, Ferrer A, Martinez-Gonzalez MA. Intervention study for smoking cessation in diabetic patients. Diabetes Care 2000;23(10):1455-60.																																X				
Hopper SV, Miller JP, Birge C, Swift J. A randomized study of the impact of home health aides on diabetic control and utilization patterns. American Journal Public Health 1984;74(6):600-2.																																X	X			
Matsuyama JR, Mason BJ, Jue SG. Pharmacists' interventions using an electronic medication-event monitoring device's adherence data versus pill counts. The Annals of Pharmacotherpay 1993;27:851-5.																																X				
Pullar T, Birtwell AJ, Wiles PG, Hay A, Feely MP. Use of a pharmocologic indicator to compare compliance with tablets prescribed to be taken once, twice, or three times daily. Clinical pharmacology and therapeutics 1988;44:540-5																																X				
Rosenkranz S. Polaroid-Fundusfotografie erhöht die Patientencompliance beim Screening auf diabetische Retinopathie. Diabetes und Stoffwechsel 1006;5:69-75																																X				
Simmons D, Upjohn M, Gamble GD. Can medication packaging improve glycemic contol and blood pressure in type 2 diabetes?. Diabetes Care 2000;23(2):153-6																																X				
Skaer TL, Sclar DA, Markowski DJ, Won JKH. Effect of value-added utilities on prescription refill compliance and Medicaid health care expenditures - a study of patients with non-insulin-dependent diabe. 1993;18:295-9.																																X				
Smith DM, Norton JA, Weinberger M, McDonald CJ, Katz BP. Increasing prescribed office visits. A controlled trial in patients with diabetes mellitus. Medical Care 1986;24(3):189-99.																																X				
Diehl AK, Sugarek NJ, Bauer RL. Medication compliance in non-insulin-dependent diabetes: a randomized comparison of chlorpropamide and insulin. Diabetes Care 1985;8(3):219-23																																X				
Davidson MB, Karlan VJ, Hair TL. Effect of a pharmacist-managed diabetes care program in a free medical clinic. American Journal of Medical Quality 2000;15(4):137-42.																																X				
Bradshaw C, McColl E, Eccles M, Bryce C, Sampson R. Can we improve the education for type 2 diabetes patients in general practice?. Practical Diabetes International 1999;16(8):241-5																																X				
Clarke J, Crawford A, Nash DB. Evaluation of a comprehensive diabetes disease management program: progress in the struggle for sustained behavior change. Disease management 2002;5(2):77-86.																																X				
Paes AHP, Bakker A, Soe-Agnie CJ. Impact of dosage frequency on patient compliance. Diabetes Care 1997;20(10):1512-7.																																X				
Allen BT, DeLong ER, Feussner JR. Impact of glucose																																	X			

self-monitoring on non-insulin-treated patients with type 11 diabetes mellitus. Randomized controlled trial comparing blood and urine testing. Diabetes Care. 1990;13:1044-1050.																																				
Arseneau DL, Mason AC, Wood OB, Schwab E, Green D. A comparison of learning activity packages and classroom instruction for diet management of patients with non-insulin-dependent diabetes mellitus. Diabetes Educ. 1994;20:509-514.																																		X		X
Aubert RE, Herman WH, Waters J, et al. Nurse case management to improve glycemic control in diabetic patients in a health maintenance organization: a randomized controlled trial. Ann Intern Med. 1998;129:605-612																																		X		
Bloomgarden ZT, Karmally W, Metzger MJ, et al. Randomized, controlled trial of diabetic patient education: improved knowledge without improved metabolic status. Diabetes Care. 1987;10:263-272.																																		X	X	X
Campbell LV, Barth R, Gosper JK, Jupp JJ, Simons LA, Chisholm DJ. Impact of an intensive educational approach to dietary change in NIDDM. Diabetes Care. 1990;13:841-847.																																		X		
Falkenberg MG, Elwing BE, Goransson AM, Hellstrand BE, Riis UM. Problem oriented participatory education in the guidance of adults with non-insulintreated type 11 diabetes mellitus. Scand J Primary Health Care. 1986;4:157-164.																																		X	X	
Franz MJ, Monk A, Barry B, et al. Effectiveness of medical nutrition therapy provided by dietitians in the management of non-insulindependent diabetes mellitus: a randomized, controlled clinical trial. J Am Diet Assoc. 1995;95:1009-1017.																																		X	X	X
Genev NM, McGill M, Hoskins PL, et al. Continuing diabetes education by telephone. Diabetic Med. 1990;7:920-921.																																		X		
Glasgow RE, Toobert DJ, Mitchell DL, Donnelly JE, Calder D. Nutrition education and social learning interventions for type 11 diabetes. Diabetes Care. 1989;12:150-152.																																		X		X
Glasgow RE, Toobert DJ, Hampson SE, Brown JE, Lewinsohn PM, Donnelly J. Improving self-care among older patients with type 11 diabetes: the "Sixty Something..." Study. Patient Educ Counsel. 1992;19:61-74.																																		X	X	X
Greenfield S, Kaplan SH, Ware JE Jr, Yano EM, Frank HJ. Patients' participation in medical care: effects on blood sugar control and quality of life in diabetes. J Gen Intern Med. 1988;3:448-457.																																		X		
Hassell J, Medved E. Group/audiovisual instruction for patients with diabetes. Learning achievements and time economics. J Am Diet Assoc. 1975;66:465-470.																																		X		
Hayes TM, Harries J. Randomised controlled trial of routine hospital clinic care versus routine general practice care for type 11 diabetics. Br Med J (Clin Res																																		X		

ed.). 1984;289:728-730																																				
Hurwitz B, Goodman C, Yudkin J. Prompting the clinical care of non-insulin dependent (type 11) diabetic patients in an inner city area: one model of community care. Br Med J. 1993;306:624-630.																																	X		
Jones PM. Use of a course on self-control behavior techniques to increase adherence to prescribed frequency for self-monitoring of blood glucose. Diabetes Educ. 1990;16:296-303.																																	X		X
Koproski J, Pretto Z, Poretsky L. Effects of an intervention by a diabetes team in hospitalized patients with diabetes. Diabetes Care. 1997;20:1553-1555.																																	X		
Kumana CR, Ma JT, Kung A, Kou M, Lauder 1. An assessment of drug information sheets for diabetic patients: only active involvement by patients is helpful. Diabetes Res Clin Pract. 1988;5:225-231.																																	X		X
Miller CK, Jensen GL, Achterberg CL. Evaluation of a food label nutrition intervention for women with type 2 diabetes mellitus. J Am Diet Assoc. 1999;99:323-328																																	X		
Morgan BS, Littell DH. A closer look at teaching and contingency contracting with type 11 diabetes. Patient Educ Counsel. 1988;12:145-158.																																	X		
Morrish NJ, Cohen DL, Hicks B, Keen H. A controlled study of the effect of computer-aided analysis of home blood glucose monitoring on blood glucose control. Diabetic Med. 1989;6:591-594																																	X		
Mulrow C, Bailey S, Sonksen PH, Slavin B. Evaluation of an audiovisual diabetes education program: negative results of a randomized trial of patients with non-insulin-dependent diabetes mellitus. J Gen Intern Med. 1987;2:215-219.																																	X	X	X
Rabkin SW, Boyko E, Wilson A, Streja DA. A randomized clinical trial comparing behavior modification and individual counseling in the nutritional therapy of non-insulin-dependent diabetes mellitus: comparison of the effect on blood sugar, body weight, and serum lipids. Diabetes Care. 1983;6:50-56.																																	X		X
Rettig BA, Shrauger DG, Recker RR, Gallagher TF, Wiltse H. A randomized study of the effects of a home diabetes education program. Diabetes Care. 1986;9:173-178.																																	X		X
Rost KM, Flavin KS, Cole K, McGill JB. Change in metabolic control and functional status after hospitalization. Impact of patient activation intervention in diabetic patients. Diabetes Care. 1991;14:881-889.																																	X		
Sadur CN, Moline N, Costa M, et al. Diabetes management in a health maintenance organization. Efficacy of care management using cluster visits. Diabetes Care. 1999;22:2011-2017																																	X		
Sawicki PT, Didjurgeit U, Muhlhauser 1, Berger M. Behaviour therapy versus doctor's anti-smoking advice in diabetic patients. J Intern Med. 1993;234:407-409																																	X		
Scott RS, Beaven DW, Stafford JM. The effectiveness of diabetes education for non-insulin-dependent diabetic																																	X	X	X

persons. Diabetes Educ. 1984:36-39.																																			
Smith DM, Weinberger M, Katz BP A controlled trial to increase office visits and reduce hospitalizations of diabetic patients. J Gen Intern Med. 1987;2:232-238.																																	X		
Tu K, McDaniel G, Gay JT. Diabetes self-care knowledge, behaviors, and metabolic control of older adults-the effect of a posteducational follow-up program. Diabetes Educ. 1993; 19:25-30																																	X	X	X
Vinicor F, Cohen SJ, Mazzuca SA, et al. DIA-BEDS: a randomized trial of the effects of physician and/or patient education on diabetes patient outcomes. J Chron Dis. 1987;40:345-356.																																	X		X
Ward WK, Haas LB, Beard JC. A randomized, controlled comparison of instruction by a diabetes educator versus self-instruction in self-monitoring of blood glucose. Diabetes Care. 1985;8:284-286.																																	X		X
Weinberger M, Kirkman MS, Samsa GE et al. A nurse-coordinated intervention for primary care patients with non-insulindependent diabetes mellitus. J Gen Intern Med. 1995;10:59-66																																	X		
Wheeler LA, Wheeler ML, Ours P, Swider C. Evaluation of computer-based diet education in persons with diabetes mellitus and limited educational background. Diabetes Care. 1985;8:537-544.																																	X		X
Wing RR, Epstein LH, Nowalk MP, Scott N, Koeske R, Hagg S. Does self-monitoring of blood glucose levels improve dietary compliance for obese patients with type 11 diabetes? Am J Med. 1986;81:830-836																																	X	X	X
Wing RR, Epstein LH, Paternostro-Bayles M, Kriska A, Nowalk MP, Gooding W. Exericise in a behavioural weight control programme for obese patients with type 2 (non-insulin-dependent) diabetes. Diabetologia. 1988;31:902-909.																																	X		
Wood ER. Evaluation of a hospital-based education program for patients with diabetes. J Am Diet Assoc. 1989;89:354-358																																	X		X
de Weerdt I, Visser A, Kok G, de Weerdt O, van der Veen E: Randomized controlled multicentre evaluation of an education programme for insulin-treated diabetic patients: effects on metabolic control, quality of life, and costs of therapy. Diabet Med 8:338–345, 1991																																		X	X
Estey A, Tan M, Mann K: Follow-up intervention: its effect on compliance behavior to diabetes regimen. Diabetes Educ 16:291–295, 1990																																		X	X
Kaplan R, Wilson D, Hartwell S, Merino K, Wallace J: Prospective evaluation of HDL cholesterol changes after diet and physical conditioning programs for patients with type II diabetes mellitus. Diabetes Care 8:343–348,																																		X	X
Korhonen T, Uusitupa M, Aro A, Kumpulainen T, Siitonen O, Voutilainen E, Pyorala K: Efficacy of dietary instructions in newly diagnosed non-insulin-dependent diabetic patients. Acta Med Scand 222:323–331, 1987																																		X	X

Mazzuca SA, Moorman NH, Wheeler ML, Norton JA, Fineberg NS, Vinicor F, Cohen SJ, Clark CM Jr: The diabetes education study: a controlled trial of the effects of diabetes patient education. Diabetes Care 9:1–10,																																			X	X
McCulloch D, Mitchell R, Ambler J, Tattersall R: Influence of imaginative teaching of diet on compliance and metabolic control in insulin dependent diabetes. Br Med J 287:1858–1861, 1983																																			X	
Perry T, Mann J, Lewis-Barned N, Duncan A, Waldron M, Thompson C: Lifestyle intervention in people with insulin-dependent diabetes mellitus (IDDM). Eur J Clin Nutr 51:757–763, 1997																																			X	X
Turnin M-C, Beddok R, Clottes J, Martini P, Abadie R, Buisson J-C, Soule-Cupuy C, Bonneu M, Camare R, Anton J-P, Chrisment C, Farreny H, Bayard F, Tauber J-P: Telematic expert system Diabeto: new tool for diet self-monitoring for diabetic patients. Diabetes Care 15:204–212, 1992																																			X	X
Wing R, Epstein L, Nowalk M, Scott N: Self-regulation in the treatment of type II diabetes. Behav Ther 19:11–23, 1988																																			X	X
Fernando D: Knowledge about diabetes and metabolic control in diabetic patients. Ceylon Med J 38:18-21, 1993																																				X
Falkenberg M, Elwing B, Goransson A, Hellstrand B, Riis U: Problem-oriented participatory education in the guidance of adults with non-insulin-treated type-II diabetes mellitus. Scand J Prim Health Care 4:157-164, 1986																																				X
McCulloch D, Mitchell R, Ambler J, Tattersall R: Influence of imaginative teaching of diet on compliance and metabolic control in insulin-dependent diabetes. BMJ 287:1858-1861, 1983																																				X
Korhonen T, Huttunen J, Aro A, Hentinen M, Ihalainen O, Majander H, Siitonen O, Uusitupa M, Pyorala K: A controlled trial on the effects of patient education in the treatment of insulin-dependent diabetes. Diabetes Care 6:256- 261, 1983																																				X
Kendall P. Jansen G: Educating patients with diabetes: comparison of nutrient-based and exchange group methods. J Am Diet Assoc 90:238-243, 1990																																				X
de Weerdt I, Visser A, Kok G, van der Veen E: Randomized controlled evaluation of an education programme for insulin treated patients with diabetes: effects on psychosocial variables. Patient Educ Counsel 14:191-215, 1989																																				X
Brown S, Duchin S, Villagomez E: Diabetes education in a Mexican-American population: pilot testing of a research-based videotape. Diabetes Educator 18:47-51, 1992																																				X
Kim J-Y, Phillips T: The effectiveness of two forms of																																				X

corrective feedback in diabetes education. J Comput Based Instruct 18:14-18, 1991																																				
Colagiuri R, Colagiuri S, Naidu V: Can patients set their own educational priorities? Diabetes Res Clin Pract 30:131-136, 1995																																				X
Small R, Hill Hopper J: Evaluation of videotape teaching of self-monitoring of blood glucose by elderly diabetic patients. Consultant Pharmacist 7:24-27, 1992																																				X
Van Veldhuizen-Scott M, Widmer L, Stacey S, Popovich N: Developing and implementing a pharmaceutical care model in an ambulatory care setting for patients with diabetes. Diabetes Educator 21:117-123, 1995																																				X
Genev N, McGill M, Hoskins P, Constantino M, Plehwe W, Yue D, Turtle J: Continuting diabetes education by telephone. Diabet Med 7:920-921, 1990																																				X
Litzelman D, Slemenda C, Langefeld C, Hays L, Welch M, Bild D, Ford E, Vinicor F: Reduction of lower extremity clinical abnormalities in patients with noninsulin-dependent diabetes mellitus: a randomized, controlled trial. Ann Intern Med 119:36-41, 1993																																				X
Kruger S, Guthrie D: Foot-care: knowledge retention and self-care practices. Diabetes Educator 18:487-490, 1992																																				X
Hanefeld M, Fischer S, Schmechel H, Rothe G, Schulze J, Dude H, Schwanebeck U, Julius U: Diabetes intervention study: multi-intervention trial in newly diagnosed NIDDM. Diabetes Care 14:308-317, 1991																																				X
de Bont A, Baker I, St. Leger A, Sweetnam P, Wragg K, Stephens S, Hayes T: A randomised controlled trial of the effect of low-fat diet advice on dietary response in insulin-independent diabetic women. Diabetologia 21:529-533, 1981																																				X
Glasgow R, La Chance P-A, Toobert D, Brown J, Hampson S, Riddle M: Long term effects and costs of brief behavioral dietary intervention for patients with diabetes delivered from the medical office. Patient Educ Couns 32:175-184, 1997																																				X
Mengham L, Morris B, Palmer C, White A: Is intensive dietetic intervention effective for overweight patients with diabetes mellitus? A randomised controlled study in a general practice. Practical Diabetes International 16:5-8, 1999																																				X
Frost G, Wilding J, Beecham J: Dietary advice based on the glycaemic index improves dietary profile and metabolic control in type 2 diabetic patients. Diabet Med 11:397-401, 1994																																				X
Pratt C, Wilson W, Leklem J, Kingsley L: Peer support and nutrition education for older adults with diabetes. J Nutr Elderly 6:31-43, 1987																																				X
Hitchcock Noel P, Larme A, Meyer J, Marsh G, Correa A, Pugh J: Patient choice in diabetes education curriculum: nutritional versus standard content for type 2 diabetes. Diabetes Care 21: 896-901, 1998																																				X
Werdier D, Jesdinsky H, Helmich P: A randomized, controlled study on the effect of diabetes counseling in																																				X

the offices of 12 general practitioners. Rev Epidemiol Sante Publique 32:225-229, 1984																																				
Gilden J, Hendryx M, Clar S, Casia C, Singh S: Diabetes support groups improve health care of older diabetic patients. J Am Geriatr Soc 40:147-150, 1992																																				X
Laitinen J, Ahola I, Sarkkinen E, Winberg R, Harmaakorph-Livonen P, Uusitupa M: Impact of intensified dietary therapy on energy and nutrient intakes and fatty acid composition of serum lipids in patients with recently diagnosed non-insulin-dependent mellitus. J Am Diet Assoc 93:276-283, 1993																																				X
Rainwater N, Ayllon T, Frederiksen L, Moore E, Bonar J: Teaching self-management skills to increase diet compliance in diabetics. In Adherence, Compliance, and Generalization in Behavioral Medicine. Stuart R, Ed. New York, Brummer/Mazel, 1982, p. 304 -328																																				X
Wilson W, Pratt C: The impact of diabetes education and peer support upon weight and glycemic control of elderly persons with non-insulin dependent diabetes mellitus (NIDDM). Am J Public Health 77:634-635, 1987																																				X
Mazzuca K, Farris N, Mendenball J, Stoupa R: Demonstrating the added value of community health nursing for clients with insulin-dependent diabetes. J Community Health Nurs 14:211-224, 1997																																				X
Franz M, Splett F, Monk A, Barry B, McClam K, Weaker T, Upham P, Bergenstal R, Mazze R: Cost-effectiveness of medical nutrition therapy provided by dietitians for persons with non-insulin-dependent diabetes mellitus. J Am Diet Assoc 95:1018-1024, 1995																																				X
Vanninen E, Uusitupa M, Siitonen O, Laitinen J, Lansimies E: Habitual physical activity, aerobic capacity and metabolic control in patients with newly-diagnosed type 2 (non-insulin-dependent) diabetes mellitus: effect of 1-year diet and exercise intervention. Diabetologia 35:340-346, 1992																																				X
Wing R, Nowalk M, Epstein L, Koeske R: Calorie-counting compared to exchange system diets in the treatment of over-weight patients with type II diabetes. Addict Behav 11:163-168, 1986																																				X
Basch C, Walker E, Howard C, Shamoon H, Zybert P: The effect of health education on the rate of opthalmic examinations among African Americans with diabetes mellitus. Am J Public Health 89: 1878-1882, 1999																																				X
Julius U, Gross P, Hanefeld M, DIS-Group: Work absenteeism in type 2 diabetes mellitus: results of the prospective diabetes intervention study. Diabete Metab 19:202-206, 1993																																				X
Lockington T, Farrant S, Meadown K, Dowlatshahi D, Wise P: Knowledge profile and control in diabetic patients. Diabet Med 5:381-386, 1988																																				X
	53	28	17	16	132	13	33	17	20	39	10	13	16	13	8	12	26	15	7	20	10	47	35	9	NP	11	11	20	8	36	3	14	21	62	31	84

1= Choi, Davidson, Walker, Lee, & Palermo, 2016; 2=Creamer, Attridge, Ramsden, Cannings-John, & Hawthorne, 2016; 3=Huang et al., 2016; 4=Chen et al., 2015; 5=Pillay et al., 2015; 6= Terranova,

Brakenridge, Lawler, Eakin, & Reeves, 2015; 7= Attridge, Creamer, Ramsden, Cannings-John, & Hawthorne, 2014; 8= Odnoletkova et al., 2014; 9 = Pal et al., 2014; 10 = Ricci-Cabello et al., 2014; 11= Saffari, Ghanizadeh, & Koenig, 2014; 12=Gucciardi, Chan, Manuel, & Sidani, 2013; 13=Pal et al., 2013; 14=Vugt, Wit, Cleijne, & Snoek, 2013; 15= Amaeshi, 2012 ; 16= Nam, Janson, Stotts, Chesla, & Kroon, 2012 ; 17= Steinsbekk, Rygg, Lisulo, Rise, & Fretheim, 2012; 18= Burke et al., 2011; 19= Kai Lun Gan, Brammer, & Creedy, 2011; 20=Ramadas, Quek, Chan, & Oldenburg, 2011; 21=Hawthorne, Robles, Cannings-John, & Edwards, 2010; 22= Minet, Moller, Vach, Wagner, & Henriksen, 2010; 23=Alam, Sturt, Lall, & Winkley, 2009; 24= Duke, Colagiuri, & Colagiuri, 2009; 25= Fan & Sidani, 2009; 26=Hawthorne, Robles, Cannings-John, & Edwards, 2008 ; 27=Khunti, Camosso-Stefinovic, Carey, Davies, & Stone, 2008; 28=Loveman, Frampton, & Clegg, 2008 ; 29=Wens et al., 2008; 30 = Nield et al., 2007; 31=Zabaleta & Forbes, 2007; 32=Deakin, McShane, Cade, & Williams, 2005; 33=Vermeire et al., 2005; 34=Gary, Genkinger, Guallar, Peyrot, & Brancati, 2003; 35=Norris, Lau, Smith, Schmid, & Engelgau, 2002; 36=Norris, Engelgau, & Narayan, 2001. T2DM = type 2 diabetes mellitus; NP = not applicable.

% overlap 177/532= 33.3%

CA= Covered Area =

(53+28+17+16+132+13+33+17+20+39+10+13+16+13+8+12+26+15+7+20+10+47+35+9+11+11+20+8+36+3+14+21+62+31+84)/(35x532) = 910/18620=4.8% (one review did not report the included studies list)

CCA= Corrected Covered Area = (910-532)/ (18620-532) = 378/18088= 2.1% (slight)
