# Supplementary material for: Health education for patients with acute coronary syndrome and type 2 diabetes mellitus: an umbrella review of systematic reviews and meta-analyses
Source: BMJ Open. 2017 Oct 16;7(10):e016857. doi: 10.1136/bmjopen-2017-016857 (PMC5652525; doi:10.1136/bmjopen-2017-016857)
Supplement: Author's manuscript [file bmjopen-2017-016857.draft_revisions.pdf]

BMJ Open

BMJ Open is committed to open peer review. As part of this commitment we make the peer review history of every article we publish publicly available.

When an article is published we post the peer reviewers' comments and the authors' responses online. We also post the versions of the paper that were used during peer review. These are the versions that the peer review comments apply to.

The versions of the paper that follow are the versions that were submitted during the peer review process. They are not the versions of record or the final published versions. They should not be cited or distributed as the published version of this manuscript.

BMJ Open is an open access journal and the full, final, typeset and author-corrected version of record of the manuscript is available on our site with no access controls, subscription charges or pay-per-view fees (http://bmjopen.bmj.com).

If you have any questions on BMJ Open's open peer review process please email editorial.bmjopen@bmj.com

Health Education for Patients with Acute Coronary Syndrome and Type 2 Diabetes Mellitus: An Umbrella Review of Systematic Reviews and Meta-Analyses

Journal:	*BMJ Open*
Manuscript ID	bmjopen-2017-016857
Article Type:	Research
Date Submitted by the Author:	16-Mar-2017
Complete List of Authors:	Liu, Xian-liang<br>Shi, Yan<br>Willis, Karen<br>Wu, Jo<br>Johnson, Maree; Australian Catholic University, Faculty of Health Sciences
**Primary Subject Heading**:	Evidence based practice
Secondary Subject Heading:	Cardiovascular medicine, Diabetes and endocrinology
Keywords:	Health Education, Acute Coronary Syndrome, Type 2 Diabetes Mellitus, Umbrella Review

SCHOLARONE™
Manuscripts

Title page

Health Education for Patients with Acute Coronary Syndrome and Type 2 Diabetes Mellitus: An Umbrella Review of Systematic Reviews and Meta-Analyses

Xian-Liang Liu, RN, MScN

Ph.D. Candidate, School of Nursing, Midwifery and Paramedicine, Australian Catholic University, Australia; Ph.D. Candidate, Tenth People's Hospital of Tongji University, China; Lecturer, School of Nursing, Jinggangshan University, China.

Address: 1100 Nudgee Road, Banyo, Brisbane, QLD, Australia

Email: liu.xianliang@myacu.edu.au; 1988_liuxianliang@tongji.edu.cn

Yan Shi, RN, Ph.D., Professor, Executive Director of Nursing Department

Tenth People's Hospital and Tongji University, China

Address: 301, YanChang Road, Shanghai, China

E-mail: shlzk2002@163.com

Karen Willis, Ph.D., Professor, Associate Dean (Learning and Teaching)

Faculty of Health Sciences, Australian Catholic University, Australia

Address: 250 Victoria Parade, Melbourne, Australia

Email: Karen.Willis@acu.edu.au

Chiung-Jung (Jo) Wu, RN, DrHlthSc, FACN

Associate Professor, School of Nursing, Midwifery and Paramedicine, University of the Sunshine Coast;

Visiting Fellow, School of Nursing, Queensland University of Technology;

Honorary Research Fellow, Royal Brisbane and Women's Hospital;

Honorary Research Fellow, Mater Medical Research Institute-University of Queensland (MMRI-

For peer review only

UQ), Brisbane, Australia;

Postal Address: ML 57, PO Box 1149, Hervey Bay Q 4655, Australia.

Email: cwu@usc.edu.au; c3.wu@qut.edu.au

Maree Johnson, RN, Ph.D., Professor, Associate Dean (Research)

Faculty of Health Sciences, Australian Catholic University, Australia

Address: Tenison Woods House (8 -20 Napier Street), North Sydney, Australia

Email: Maree.Johnson@acu.edu.au

Affiliated with the Ingham Institute of Applied Medical Research, Liverpool. Sydney.

Corresponding author:

Xian-Liang Liu, RN, MScN,

Ph.D. Candidate, School of Nursing, Midwifery and Paramedicine, Australian Catholic University, Australia; Ph.D. Candidate, Tenth People's Hospital of Tongji University, China; Lecturer, School of Nursing, Jinggangshan University, China.

Address: 1100 Nudgee Road, Banyo, Brisbane, QLD, Australia

Email: liu.xianliang@myacu.edu.au; 1988_liuxianliang@tongji.edu.cn

Tel: +61 497440830; +86 15921496130

For peer review only

Abstract

Objectives: This umbrella review aimed to identify the current evidence on health education-related interventions for patients with acute coronary syndrome or type 2 diabetes mellitus; identify the educational content, delivery methods, intensity, duration and setting required. The purpose was to provide recommendations for educational interventions for high risk patients with both acute coronary syndrome and type 2 diabetes mellitus.

Design: Umbrella review of Systematic Reviews and Meta-Analyses.

Setting: Inpatient and post discharge settings.

Participants: Patients with ACS and T2DM.

Data sources: CINAHL, Cochrane Library, Joanna Briggs Institute, Journals@Ovid, EMBase, Medline, PubMed, and Web of Science databases from January 2000 through May 2016.

Outcomes measures: Clinical outcomes (such as HbA1C), behavioral outcomes (such as smoking), psychosocial outcomes (such as anxiety) and medical service use.

Results: Fifty-one eligible reviews (15 for ACS and 36 for T2DM) consisting of 1324 relevant studies involving 288,057 patients (15 papers did not provide the total sample); 30 (58.8%) reviews were rated as high quality. Nurses only and multidisciplinary teams were the most frequent professionals to provide education, and most educational interventions were delivered post-discharge. Face-to-face sessions were the most common delivery formats, and many education sessions were also delivered by telephone or via web contact. The frequency of educational sessions was weekly or monthly, and an average of 3.7 topics was covered per education session. Psychoeducational interventions were generally effective at reducing smoking and admissions for patients with ACS. Culturally appropriate health education, self-management educational interventions, group medical visits and psychoeducational interventions were

For peer review only

generally effective for T2DM patients.

Conclusions: Results indicate that there is a body of current evidence about the efficacy of health education, its content, and delivery methods for patients with ACS or T2DM. These results provide recommendations about the content for, and approach to, health education intervention for these high risk patients.

KEY WORDS: Health Education; Acute Coronary Syndrome; Type 2 Diabetes Mellitus; Umbrella Review

Strengths and limitations of this review:

- This umbrella review is the first synthesis of systematic reviews or meta-analyses to consider health education-related interventions for patients with ACS or T2DM.
- These results provide recommendations about the content of a health education intervention for patients with ACS and T2DM.
- The diversity of the educational interventions seen in the reviews included in this umbrella review may reflect the uncertainty about the optimal strategy for providing health education to patients
- This umbrella review found no reviews focused on patients with ACS and T2DM the intended target group; instead, all of the systematic reviews and meta-analyses focused on only one of these two diseases.

For peer review only

For peer review only - http://bmjopen.bmj.com/site/about/guidelines.xhtml

Introduction

Acute coronary syndrome (ACS) is the leading cause of death worldwide. The risk of high mortality rates relating to ACS is markedly increased after an initial cardiac ischemic event.[1] Globally, 7.2 million (13%) deaths are caused by coronary artery disease (CAD),[2] and it is estimated that more than 780,000 persons will experience ACS each year in the United States.[3] Moreover, about 20–25% of patients with ACS reportedly also have diabetes mellitus (DM); predominantly type 2 diabetes mellitus [T2DM]).[4 5] Patients with ACS and DM have an increased risk of adverse outcomes such as death, recurrent myocardial infarction (MI), readmission, or heart failure during follow up.[6] Longer median delay times from symptom onset to hospital presentation, have been reported among patients with ACS and DM than patients with ACS alone.[7]

DM is now considered to confer a risk equivalent to that of CAD for patients for future MI and cardiovascular mortality.[8] Mortality was significantly higher among patients with ACS and DM than among patients with ACS only following either ST segment elevation myocardial infarction (STEMI) (8.5% [ACS and DM] versus 5.4% [ACS]) or unstable angina/non-STEMI (NSTEMI) (2.1% [ACS and DM] vs. 1.1% [ACS]).[9] ACS and T2DM are often associated with high-risk factors such as low levels of physical exercise, obesity, smoking, and unhealthy diet.[10] Some of these and other risk factors, specifically glycemia, high blood pressure (BP), lipidemia, and obesity, are frequently addressed by health education interventions.[10]

Health education interventions are comprehensive programs that healthcare providers deliver to patients aimed at improving patients' clinical outcomes through the increase and maintenance of health behaviours.[11] Along with education about, for example, medication adherence, these programs seek to increase behaviors such as physical exercise and a healthy diet thus reducing

For peer review only - http://bmjopen.bmj.com/site/about/guidelines.xhtml

patient morbidity or mortality.[11] Although there are numerous systematic reviews of educational interventions relating to ACS, or T2DM, an umbrella review providing direction on educational interventions for high risk patients with both ACS and T2DM is not available, indicating a need to gather the current evidence and develop an optimal protocol for health education programs for patients with ACS and T2DM. This umbrella review will examine the best available evidence on health education-related interventions for patients with ACS or T2DM. We will synthesize these findings to provide direction for health education-related interventions for high risk patients with both ACS and T2DM.

An umbrella review is a new method to summarize and synthesize the evidence from multiple systematic reviews/meta-analyses into one accessible publication.[12] Our aim is to systematically gather, evaluate and organize the current evidence relating the health education interventions for patients with ACS or T2DM, and proffer recommendations for the scope of educational content and delivery methods that would be suitable for patients with ACS and T2DM.

Methods

Data Sources

This umbrella review performed a literature search to identify systematic reviews and meta-analyses examining health education-related interventions for patients with ACS or T2DM. The search strategies are described in Appendix 1. This umbrella review searched eight databases for articles published from January 2000 to May 2016: CINAHL, Cochrane Library, Joanna Briggs Institute, Journals@Ovid, EMBase, Medline, PubMed, and Web of Science. The search was limited to English language only. The following broad MeSH terms were used: *acute coronary syndrome; angina, unstable; angina pectoris; coronary artery disease; coronary artery bypass; myocardial infarction; diabetes mellitus, type 2; counseling; health education; patient education*

For peer review only

as topic; and *meta-analysis [publication type];* and *meta-analysis* as a topic.

Inclusion Criteria

Participants

All participants were diagnosed with ACS or T2DM using valid, established diagnostic criteria. The diagnostic standards included those described by the American College of Cardiology/American Heart Association,[3] National Heart Foundation of Australia & Cardiac Society of Australia and New Zealand,[13] World Health Organization,[14] or other associations.

Intervention Types

For this umbrella review, health education-related interventions refer to any planned activities or programs that include behavior modification, counseling, and teaching interventions. Results considered for this review included changes in clinical outcomes (including blood pressure levels, body weight, diabetes complications, HbA1C, lipid levels, mortality rate and physical activity levels), behavioral outcomes (such as diet, knowledge, self-management skills, self-efficacy , and smoking), and psychosocial outcomes (such as anxiety, depression, quality of life, and stress) and medical service use (such as medication use, health care utilization, and cost-effectiveness) for patients with ACS or T2DM. These activities or programs included any educational interventions delivered to patients with ACS or T2DM. The interventions are delivered in any format, including face-to-face, telephone and group-based or one-on-one, and the settings include community, hospital and home. The interventions were delivered by community healthcare workers, doctors, diabetes educators, lay people, nurses, or rehabilitation therapists.

Study Types

Only systematic reviews and meta-analyses were included in this review.

For peer review only - http://bmjopen.bmj.com/site/about/guidelines.xhtml

Eligibility Assessment

The title and abstract of all of the retrieved articles were assessed independently by two reviewers (XL-L, YS) based on the inclusion criteria. All duplicate articles were identified within EndNote version X7[15] and subsequently excluded. If the information from the titles and abstract was not clear, the full articles were retrieved. The decision to include an article was based on an appraisal of the full text of all retrieved articles. Any disagreements during this process were settled by discussion and, if necessary, consensus was sought with a third reviewer. We developed an assessment form in which specific reasons for exclusion were detailed.

Assessment of Methodological Quality

The methodological quality and risk of bias were assessed for each of the included publications using the Assessment of Multiple Systematic Reviews (AMSTAR) ,[16] independently by the same two reviewers (see Table 1). The AMSTAR is an 11-item tool, with each item provided a score of 1 (specific criterion is met) or 0 (specific criterion is not met, unclear, or not applicable).[16 17] An overall score for the review methodological quality is then calculated as the sum of the individual item scores: high quality, 8–11; medium quality, 4–7; or low quality, 0–3.[17] If the required data were not available in the article, the original authors were contacted for more information.

Data Extraction

Data were independently extracted by two reviewers using a predefined data extraction form. For missing or unclear information, the primary authors were contacted for clarification.

Statistical Presentation of Results from Reviews

All of the results were extracted for each included systematic review or meta-analysis, and the

For peer review only - http://bmjopen.bmj.com/site/about/guidelines.xhtml

overall effect estimates are presented in a tabular form. The number of systematic reviews or meta-analyses that reported the outcome, total sample (from included publications), and information of health education interventions is also presented in the Tables (Table 2, 3).[18] A final 'summary of evidence' was developed to present the intervention, included study synthesis, and indication of the findings from the included papers (Table 4).[18]

Synthesizing the Results and Rating the Evidence for Effectiveness

The statements of evidence were based on a rating scheme to gather and rate the evidence across the included publications.[19] The statements of evidence were based on the following rating scheme: *sufficient evidence*, sufficient data to support decisions about the effect of the health education-related interventions.[19] A rating of *sufficient evidence* in this review is obtained when systematic reviews or meta-analyses with a large number of included articles or participants produce a statistically significant result between the health education group and the control group.[19] *Some evidence*, is a less conclusive finding about the effects of the health education-related interventions[19] with statistically significant findings found in only a few included reviews or studies. *Insufficient evidence*, refers to not enough evidence to make decisions about the effects of the health education-related interventions, such as non-significant results between the health education group and the control group in the included systematic reviews or meta-analyses.[19] *Insufficient evidence to determine*, refers to not enough pooled data to be able to determine whether of the health education-related interventions are effective or not based on the included reviews.[19]

Results

Characteristics of Included Trials

The selection process and number of studies at each step was illustrated as presented in Figure 1.

For peer review only

The database search yielded 692 publications, with removal of 197 duplicates and 372 articles that did not meet the inclusion criteria, 124 full-text articles were retrieved after applying the methodological quality rating (AMSTAR scale), and three studies [20-22] were removed due to low scores ≤3 on the AMSTAR scale. Fifty-one systematic reviews or meta-analyses [23-73] conducted between 2001 and 2016 and published in English were included (Figure 1; Tables 1 –3); 15 relating to ACS and 36 relating to T2DM. None of the articles included patients with both ACS and T2DM. The umbrella review involved a total of 277,493 patients, including 225,034 patients with coronary heart disease or ACS (one article did not report the total sample) and 52,459 patients with T2DM (16 papers did not report the total sample). The average sample size was 8,161 (range, 536–68,556) participants. Of the included systematic reviews or meta-analyses, eleven were published in *The Cochrane Library*. Nine of the articles described meta-analyses, 29 articles described systematic reviews, and the remaining 13 articles were described as systematic reviews and meta-analyses or meta-regressions or narrative reviews.

For peer review only

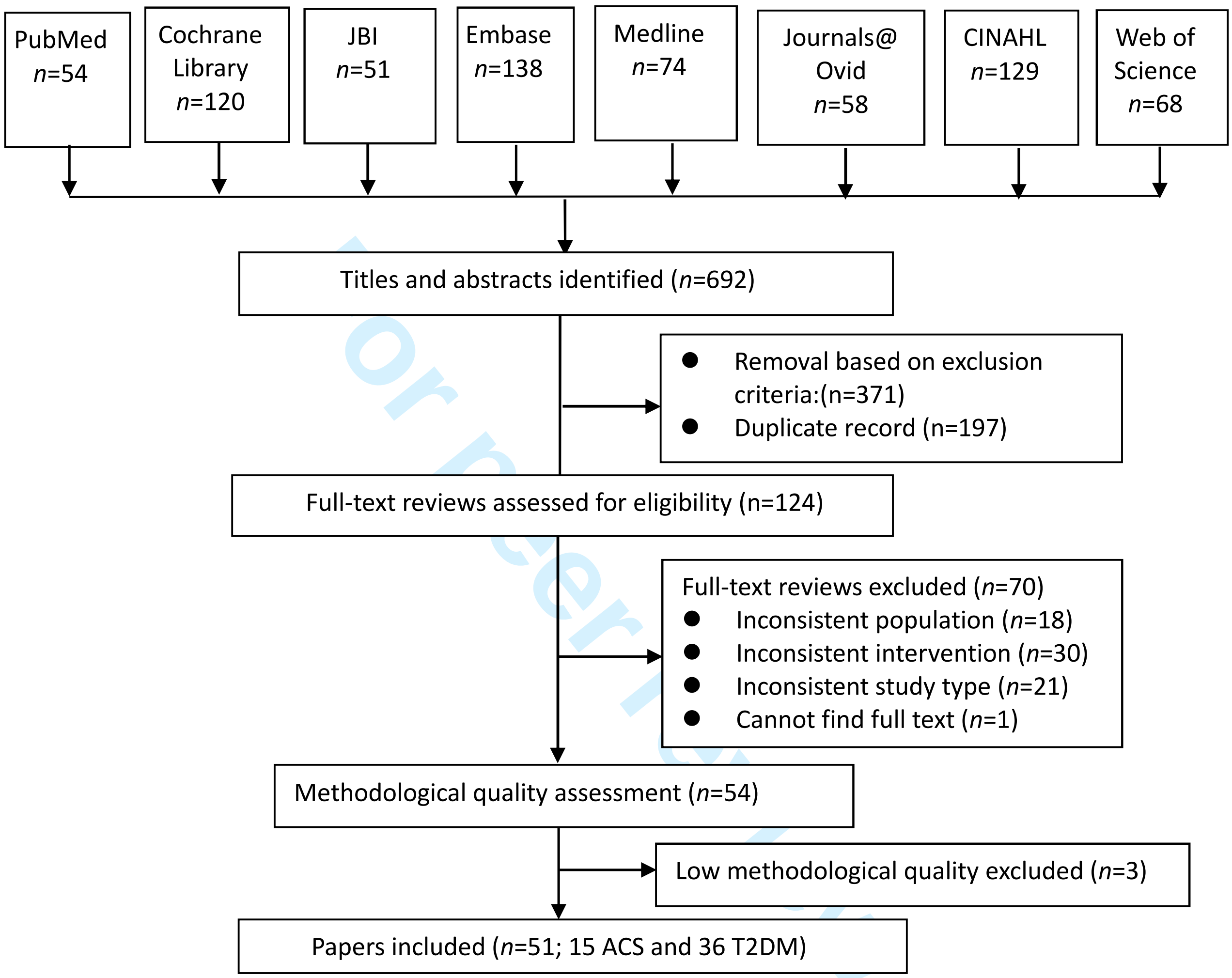

Fig. 1. Flow chart of the systematic reviews and meta-analyses selection process

Electronic database searches were conducted for all systematic reviews or meta-analyses, with an average of 6 databases searched (range, 2–16). The dates searched ranged widely, from inception of the database through December 2014. Most of the included reviews were randomized controlled trials (RCTs), and an average of 25.6 (range, 7–132) studies was included per systematic review or meta-analyses. Of the total 1,308 studies that were included in all of the reviews or meta-analyses, 406 included patients with ACS, and 902 included patients with

For peer review only - http://bmjopen.bmj.com/site/about/guidelines.xhtml

T2DM. The included reviews assessed the risk of bias using the Cochrane risk of bias tool (22 publications), JADA quality score (seven publications), Joanna Briggs quality assessment tool (two publications), PEDro scale (one publication), RCT Critical Appraisal Skills Programme (one publication), and the SIGN-50 checklist (one publication).

Methodological Quality of Included Systematic Reviews and Meta-Analyses

The methodological quality of the included publications is presented in Table 1. Thirty (58.8%) publications were classified as high quality (scores 8–11), and 21 (41.2%) publications were classified as medium quality (scores 4–7). Twenty five (49%) reviews specifically provided an *a priori* design, while the use of such a design was unclear for 26 (51%) publications. The inclusion of other forms of literature (such as grey literature) was described in 18 (35%) reviews. Only 14 out of 51 (27%) reviews included a table of included and excluded studies. Only 2 (4%) reviews did not provide a characteristics table of the included papers. The scientific quality of the included papers was evaluated and documented in 47 (92%) reviews. The scientific quality of the included studies was used appropriately to formulate conclusions in 47 (92%) reviews. The methods to combine the results of the included studies were appropriate in 43 (86%) reviews. Publication bias was assessed in only 19 (37%) reviews. Finally, conflicts of interest were reported in 47 (92%) reviews.

For peer review only

Table 1. Methodological quality assessment of included systematic reviews and meta-analyses

	Systematic Review/ Meta-analysis	Item 1	Item 2	Item 3	Item 4	Item 5	Item 6	Item 7	Item 8	Item 9	Item 10	Item 11	Total score
**Systematic reviews and meta-analysis involved ACS patients**													
1	Barth, Jacob, Daha, & Critchley, 2015 [73]	Yes	Yes	Yes	Yes	Yes	Yes	Yes	Yes	Yes	Yes	Yes	11
2	Devi et al., 2015 [37]	Yes	Yes	Yes	Yes	Yes	Yes	Yes	Yes	Yes	NA	Yes	10
3	Ghisi, Abdallah, Grace, Thomas, & Oh, 2014 [43]	CA	Yes	Yes	Yes	No	Yes	Yes	Yes	Yes	No	No	7
4	Kotb, Hsieh, & Wells, 2014 [52]	CA	Yes	Yes	Yes	No	Yes	Yes	Yes	Yes	No	Yes	8
5	Brown, Clark, Dalal, Welch, & Taylor, 2013 [30]	Yes	No	Yes	CA	No	Yes	Yes	Yes	Yes	NA	Yes	7
6	Dickens et al., 2013 [38]	CA	Yes	Yes	CA	No	Yes	Yes	Yes	Yes	Yes	Yes	8
7	Aldcroft, Taylor, Blackstock, & O'Halloran P, 2011 [24]	CA	No	Yes	CA	NO	Yes	Yes	Yes	Yes	No	Yes	6
8	Brown, Clark, Dalal, Welch, & Taylor, 2011 [72]	Yes	Yes	Yes	Yes	Yes	Yes	Yes	Yes	Yes	NA	Yes	10
9	Huttunen-Lenz, Song, & Poland, 2010 [49]	CA	No	Yes	CA	No	Yes	Yes	Yes	Yes	No	No	5
10	Goulding, Furze, & Birks, 2010 [44]	Yes	Yes	Yes	CA	No	Yes	Yes	Yes	Yes	No	Yes	8
11	Auer, Gaume, Rodondi, Cornuz, & Ghali, 2008 [27]	CA	Yes	Yes	CA	No	No	Yes	No	Yes	Yes	No	5
12	J. Barth, Critchley, & Bengel, 2008 [29]	Yes	Yes	Yes	No	Yes	Yes	Yes	Yes	Yes	Yes	Yes	10
13	Fernandez et al., 2007 [41]	Yes	Yes	Yes	Yes	Yes	Yes	Yes	Yes	Yes	No	No	8
14	J. Barth, Critchley, & Bengel, 2006 [28]	CA	Yes	Yes	CA	No	Yes	CA	Yes	Yes	Yes	Yes	7

For peer review only

15	Clark, Hartling, Vandermeer, & McAlister, 2005 [34]	CA	Yes	Yes	CA	No	Yes	Yes	Yes	Yes	Yes	Yes	8
**Systematic reviews and meta-analysis involved T2DM patients**													
16	Choi, Davidson, Walker, Lee, & Palermo, 2016 [33]	CA	Yes	Yes	No	No	Yes	Yes	Yes	Yes	Yes	Yes	8
17	Creamer, Attridge, Ramsden, Cannings-John, & Hawthorne, 2016 [35]	Yes	Yes	Yes	CA	No	Yes	Yes	Yes	Yes	No	Yes	8
18	Huang et al., 2016 [48]	CA	CA	Yes	CA	No	Yes	Yes	Yes	Yes	Yes	Yes	7
19	Chen et al., 2015 [32]	CA	CA	Yes	CA	No	Yes	Yes	Yes	Yes	Yes	Yes	7
20	Pillay et al., 2015 [62]	Yes	No	Yes	Yes	No	Yes	Yes	Yes	Yes	Yes	Yes	9
21	Terranova, Brakenridge, Lawler, Eakin, & Reeves, 2015 [67]	CA	CA	Yes	No	Yes	Yes	Yes	Yes	Yes	Yes	Yes	8
22	Attridge, Creamer, Ramsden, Cannings-John, & Hawthorne, 2014 [26]	Yes	Yes	Yes	Yes	Yes	Yes	Yes	Yes	Yes	No	Yes	10
23	Odnoletkova et al., 2014 [59]	Yes	CA	Yes	CA	No	No	Yes	Yes	Yes	Yes	No	6
24	Pal et al., 2014 [60]	CA	Yes	Yes	Yes	No	Yes	Yes	Yes	Yes	No	Yes	8
25	Ricci-Cabello et al., 2014 [64]	Yes	CA	Yes	Yes	No	Yes	Yes	Yes	Yes	Yes	Yes	9
26	Saffari, Ghanizadeh, & Koenig, 2014 [65]	CA	Yes	Yes	CA	No	Yes	Yes	Yes	Yes	Yes	Yes	8
27	Gucciardi, Chan, Manuel, & Sidani, 2013 [45]	CA	Yes	Yes	No	No	Yes	Yes	Yes	Yes	No	Yes	7
28	Pal et al., 2013 [61]	Yes	Yes	Yes	Yes	Yes	Yes	Yes	Yes	Yes	No	Yes	10
29	Vugt, Wit, Cleijne, & Snoek, 2013 [69]	CA	Yes	Yes	CA	No	Yes	Yes	Yes	NA	No	Yes	6
30	Amaeshi, 2012 [25]	CA	CA	Yes	No	No	Yes	Yes	Yes	NA	No	No	4
31	Nam, Janson, Stotts, Chesla, &	CA	CA	Yes	Yes	No	Yes	Yes	Yes	Yes	Yes	Yes	8

For peer review only

	Kroon, 2012 [55]												
32	Steinsbekk, Rygg, Lisulo, Rise, & Fretheim, 2012 [66]	CA	Yes	Yes	CA	No	Yes	Yes	Yes	Yes	No	Yes	7
33	Burke et al., 2011 [31]	Yes	Yes	Yes	Yes	Yes	Yes	Yes	Yes	Yes	NA	Yes	10
34	Kai Lun Gan, Brammer, & Creedy, 2011 [50]	Yes	Yes	Yes	CA	No	Yes	Yes	Yes	Yes	No	Yes	8
35	Ramadas, Quek, Chan, & Oldenburg, 2011 [63]	CA	CA	Yes	No	No	Yes	Yes	Yes	NA	No	Yes	5
36	Hawthorne, Robles, Cannings-John, & Edwards, 2010 [47]	Yes	Yes	Yes	CA	No	Yes	Yes	Yes	Yes	CA	Yes	8
37	Minet, Moller, Vach, Wagner, & Henriksen, 2010 [54]	Yes	Yes	Yes	No	No	Yes	Yes	Yes	Yes	Yes	Yes	9
38	Alam, Sturt, Lall, & Winkley, 2009 [23]	Yes	Yes	No	CA	No	Yes	Yes	Yes	Yes	Yes	Yes	8
39	Duke, Colagiuri, & Colagiuri, 2009 [39]	Yes	CA	Yes	No	Yes	Yes	Yes	Yes	Yes	No	Yes	8
40	Fan & Sidani, 2009 [40]	Yes	No	Yes	CA	No	Yes	No	No	Yes	No	Yes	5
41	Hawthorne, Robles, Cannings-John, & Edwards, 2008 [46]	Yes	Yes	Yes	Yes	Yes	Yes	Yes	Yes	Yes	Yes	Yes	11
41	Khunti, Camosso-Stefinovic, Carey, Davies, & Stone, 2008 [51]	CA	Yes	Yes	Yes	No	Yes	No	No	No	No	Yes	5
42	Loveman, Frampton, & Clegg, 2008 [53]	Yes	CA	Yes	Yes	No	Yes	Yes	Yes	Yes	No	Yes	8
43	Wens et al., 2008 [70]	CA	Yes	Yes	CA	No	Yes	Yes	Yes	Yes	NA	Yes	7
45	Nield et al., 2007 [56]	Yes	Yes	Yes	CA	Yes	Yes	Yes	Yes	Yes	No	Yes	9
46	Zabaleta & Forbes, 2007 [71]	CA	CA	Yes	CA	Yes	Yes	Yes	Yes	NA	No	No	5
47	Deakin, McShane, Cade, & Williams, 2005 [36]	Yes	Yes	Yes	Yes	Yes	Yes	Yes	Yes	Yes	Yes	Yes	11

For peer review only

**48**	Vermeire et al., 2005 [68]	Yes	Yes	Yes	CA	Yes	Yes	Yes	Yes	Yes	No	Yes	9
**49**	Gary, Genkinger, Guallar, Peyrot, & Brancati, 2003 [42]	CA	Yes	No	Yes	No	Yes	Yes	No	Yes	No	Yes	6
**50**	Norris, Lau, Smith, Schmid, & Engelgau, 2002 [58]	CA	No	Yes	No	No	Yes	Yes	Yes	CA	No	No	4
**51**	Norris, Engelgau, & Narayan, 2001 [57]	CA	Yes	Yes	CA	No	Yes	Yes	Yes	NA	No	No	5

Item 1:"Was an 'a priori' design provided?"; **Item 2**: "Was there duplicate study selection and data extraction?"; **Item 3**: "Was a comprehensive literature search performed?"; **Item 4**: "Was the status of publication (ie. grey literature) used as an inclusion criterion?"; **Item 5**: "Was a list of studies (included and excluded) provided?"; **Item 6**: "Were the characteristics of the included studies provided?"; **Item 7**: "Was the scientific quality of the included studies assessed and documented?"; **Item 8**: "Was the scientific quality of the included studies used appropriately in formulating conclusions?"; **Item 9**: "Were the methods used to combine the findings of studies appropriate?"; **Item 10**: "Was the likelihood of publication bias assessed?"; **Item 11**: "Was the conflict of interest stated?"

CA= Cannot Answer; NA= Not Application;

Source: *Shea, B. J., Grimshaw, J. M., Wells, G. A., Boers, M., Andersson, N., Hamel, C., ... & Bouter, L. M. (2007). Development of AMSTAR: a measurement tool to assess the methodological quality of systematic reviews. BMC Medical Research Methodology, 7(1), 1.*

Characteristics of Health Educational Interventions

The description of the health educational interventions followed the Workgroup for Intervention Development and Evaluation Research (WIDER) reporting guidelines for behavior change interventions.[74] The characteristics of the recipients, setting, delivery methods, intensity, duration, and educational content of health educational interventions for patients with ACS or T2DM are summarized in Tables 2 and 3. The delivery strategies for health education included face-to-face, internet-based, phone-based, videotape, written educational materials, or mixed. The format included one-on-one (individualized), group, or both. Face-to-face sessions were the most common delivery formats, and many education sessions were also delivered by telephone/web contact or individualized counseling. The number of sessions, total contact hours,

For peer review only

and durations varied, and there was limited information about the intensity of health education for patients provided. The frequency of educational sessions was weekly or monthly, and an average of 3.7 topics was covered per education session. Nurses and multidisciplinary teams were the most frequent educators, and most education programs were delivered post-discharge.

ACS

The educational content for patients with ACS covered cardiovascular risk factors in 8 reviews (53.33%), psychosocial issues in 8 reviews (53.33%), smoking cessation in 6 reviews (40.00%), exercise in 5 reviews (33.33%), behavioral change in 5 reviews (33.33%), diet in 4 reviews (26.67%), self-management in 3 reviews (20.00%), and medication in 1 review (6.67%). Two reviews only included smoking cessation and cardiovascular risk factors. The most common educational providers were nurses and a multidisciplinary team. Six studies [24 29 41 44 49 73] (6/15, 40%) described the theoretical approach that underpinned the education intervention.

T2DM

The educational content for patients with T2DM included diet in 23 reviews (63.89%), behavioral change in 21 reviews (58.33%), self-management in 20 reviews (55.56%), exercise in 17 reviews (47.22%), glycemic control in 16 reviews (44.45%), medication in 13 reviews (36.11%), psychosocial issues in 9 reviews (25.00%), smoking cessation in 2 reviews (5.56%), cardiovascular risk factors in 2 reviews (5.56%), and DM risks in one review (2.78%). The most common providers were dieticians, nurses, and a multidisciplinary team. The number of sessions, total contact hours, and durations varied. Thirteen reviews [23 26 36 42 45-47 53 57 60 63 66 69] (13/36, 36.11%) described the theoretical approach that underpinned the education intervention.

Effect of Interventions

The outcomes of the included systematic reviews and meta-analyses were summarized in Table

For peer review only - http://bmjopen.bmj.com/site/about/guidelines.xhtml

4.

Patients with Acute Coronary Syndrome

Three major types of health education-related interventions were used for patients with ACS: general health education (only included general health information), psychoeducational interventions, and secondary prevention educational interventions (including strategies to promote a healthy lifestyle, manage medications, and reduce cardiovascular complications) as well as internet-based interventions.

General Health Education

The findings are based on our synthesis of the findings from 6 systematic reviews.[30 41 43 44 52 72] Overall, there were mixed effects of general health education on behavioral change or clinical outcomes in patients with ACS. There was *some evidence* of a positive effect of general health education on knowledge, behavior, psychosocial indicators, beliefs, and risk factor modification, but no effects for key clinical outcomes, such as cholesterol level, hospitalization, mortality, MI, and revascularization. The results for health-related quality of life, healthcare utilization, and costs were mixed; several reviews reported a significant change, and other reviews reported no significant change for these outcomes. Only one review focused on telephone-based health education. There is *some evidence* that telephone-based health education during cardiac rehabilitation might improve all-cause hospitalization, anxiety, depression, smoking cessation, and systolic BP but there is no evidence for improvements in all-cause mortality and reductions in low-density lipoprotein cholesterol.[52]

Psychoeducational Interventions

Strategies for psychoeducational interventions have a specific focus on smoking cessation, and

For peer review only - http://bmjopen.bmj.com/site/about/guidelines.xhtml

depression. The findings are based on synthesis of results from six publications.[24 28 29 38 49 73] There is *sufficient evidence* that psychoeducational programs are effective at decreasing smoking, achieving smoking abstinence, and reducing depression. One review reported no effect on smoking cessation [24] or total mortality [49].

Secondary Prevention Educational Interventions

The following statements are based on our synthesis of results from three papers.[27 34 37] There is *some evidence* that secondary prevention educational interventions reduce MI readmission rates and improve quality of life, but the intervention was ineffective in reducing revascularization, cholesterol levels, and improving smoking cessation rates. The results are mixed for mortality and re-infarction rates; two reviews [27 34] found positive effects on mortality, while one review [37] did not.

Patients with Type 2 Diabetes Mellitus

Ten types of health education-related interventions were used for patients with T2DM: culturally appropriate health education (tailored to the religious beliefs, culture, literacy, and linguistics of the geographical area), dietary advice, foot health education, group medical visits (a group education component taught by health professionals), general health education (only included general health information), improving adherence to medical treatment recommendations (e.g., promoting oral hypoglycemic adherence), lifestyle interventions (specific focus on dietary changes and increased physical activity, or stress management), psychoeducational interventions and self-management educational interventions (based on self-management skills), and therapeutic education (collaborative process needed to modify behavior and more effectively manage risk factors).

For peer review only - http://bmjopen.bmj.com/site/about/guidelines.xhtml

Culturally Appropriate Health Education

Findings are based on our synthesis of results from 8 publications.[26 35 45-47 51 55 64] Overall, there was *some evidence* of the effects of culturally appropriate health education on clinical outcomes for T2DM. There was *sufficien*t evidence that culturally appropriate health education improves HbA1c control and knowledge scores. There is *some* evidence that physical activity and clinical outcomes (blood glucose, HbA1c, BP) were improved. There was no data relating to adverse events during the intervention and follow-up (such as hypoglycemic events and mortality), and there was insufficient evidence about improvements in quality of life.

General Health Education

The statements are based on our synthesis of results from five papers.[33 39 53 65 71] Overall, there were mixed effects of general health education programs on clinical outcomes for T2DM, including HbA1C, cholesterol level, and triglyceride level. There was *some evidence* of the effectiveness of general health education on glycemic control, weight reduction, and some diabetic control outcomes (HbA1C, diabetes complications). There were no data supporting the effectiveness of general health education on reduced health service utilization, diabetic complications, improved knowledge, psychosocial outcomes, or smoking habits.

Lifestyle Interventions

The following statements are based on our synthesis of results from 6 reviews.[32 42 48 62 63 67] Overall, there were mixed effects of the lifestyle interventions on cholesterol level, HbA1C level, and weight control. There is *some evidence* that lifestyle interventions or behavioral programs are effective for blood glucose and BP management, but they appear ineffective for HbA1c control.

For peer review only

Improving Adherence to Medical Treatment Recommendations

The statements are based on our synthesis of results from three publications.[50 68 70] There is *some evidence* of the effectiveness of improving adherence to medical treatment recommendations for HbA1C control and oral hypoglycemics.

Self-Management Educational Interventions

The statements are based on our synthesis of results from 9 reviews.[36 40 54 57 58 60 61 66 69] Overall, there was *sufficient evidence* of the effects of self-management education interventions on HbA1c control, knowledge, lifestyle outcomes, and main psychosocial outcomes. However, there was *insufficient* evidence of the benefits of this education intervention on depression, quality of life, and weight control.

Other Health Education-Related Interventions

Other health education-related interventions for patients with T2DM included therapeutic education, foot health education, group medical visits, psychoeducational interventions, and dietary advice. Statements for all of these interventions are based on our synthesis of results from one review.

There is *some evidence* that foot health education is effective in reducing the incidence of lower extremity amputation. [25] There is *some evidence* that group medical visits are effective for improving HbA1c control and systolic BP management [31]. There is also *some evidence* that psychoeducational programs are effective for improving HbA1c control and psychological status.[23]

Finally, there is *insufficient evidence* that dietary advice improves glycemic and weight control or reduces diabetic micro- and macrovascular diseases.[56] There is also *insufficient evidence* for the

For peer review only

cost-effectiveness of therapeutic education for patients with T2DM.[59]

Discussion

This umbrella review identified 51 systematic reviews or meta-analyses (15 for ACS and 36 for T2DM) that assessed the outcomes of various aspects (such as the duration, contact hours, educational content, delivery mode) of the delivery of health education-related interventions relevant to high risk patients with ACS and T2DM. Health education has become an integral part of the management for people with ACS and T2DM. The most appropriate focus of the education provided to ACS patients with T2DM remains largely undefined in the literature. For example, it remains unknown if the focus should be primarily on cardiovascular risk factors, blood glucose monitoring, or all educational components for patients with both conditions.[66 72] In addition, should cardiovascular risk factors be the focus during the acute inpatient stay with other educational needs such as the smoking cessation occurring within the primary care or outpatient settings.[24 72 73]

It remains challenging to determine the specific strategy or format that is the most effective delivery mode for patients with ACS or T2DM. There is very limited evidence to guide clinicians on the duration, contact hours, educational content, delivery mode, total length, and setting of health education programs for cardiac patients.[43] For patients with DM, one study reported that more successful programs were longer than 6 months (longer duration), consisted of greater than 10 contact sessions (high intensity), and were one-on-one sessions with individualized assessment.[75]

Health Education Interventions for Patients with ACS

Use of theoretical orientation to develop educational intervention

For peer review only

Use of theory when designing behavioral change interventions may also influence effectiveness.[75] Health education using a cognitive behavioral strategy is most consistently effective in changing maladaptive illness beliefs,[44] and studies using more than 2 behavioral change strategies reported significant differences between the intervention and control groups.[24] In one review, a significant change in smoking cessation was not observed in subgroup analyses between studies that did or did not report using a theory in intervention planning;[49] however, the authors did not suggest that using a theory in program planning should be disregarded but reported that examining actual theories or mechanisms underlying health education programs is required.[49] Owing to the considerable overlap between different theories and the detailed description of the theoretical approach in only approximately 40% of the included papers, it is difficult to determine the most effective theoretical approach, but many models can be used with success, such as the health belief model (HBM), social cognitive theory (SCT) and transtheoretical model (TTM).[49 60 69 73]

Educational content

Most reviews reported that the educational content of the interventions was comprehensive. The most common topics, of the average 3.7 topics per education session, were behavioral change, cardiovascular risk factors management, exercise, psychosocial issues, and smoking cessation. An underlying principle of health education for patients with ACS is that knowledge is necessary, but not enough to develop health behaviors and change risk factors.[24 43] Age, cognitive factors, environmental factors, and social and economic background are also important considerations.[43] While interventions using a behavioral program, telephone-based content, or self-care are effective for smoking cessation, there was insufficient evidence to support that any type of educational program was more efficacious than the others.[73] Psychoeducation, which is

For peer review only - http://bmjopen.bmj.com/site/about/guidelines.xhtml

defined as multimodal, educationally based, self-management interventions,[24] led to enhanced physical activity levels within 6–12 months when added to cardiac rehabilitation (CR) and was more effective than an exercise program or health education alone.[24 49] Moreover, psychoeducational interventions were more effective for patients with ACS than other types of health education.[24 49]

Teaching strategies and outcomes

Most reviews reported that the education was provided using multiple teaching methods and in multiple settings. Nurses and multidisciplinary teams were the most frequent people providing education, and most education programs were delivered post-discharge. Although face-to-face sessions were the most common delivery format, many education sessions were also delivered by telephone or through individualized counseling. Telephone-based health education appeared to be effective for reducing hospitalizations, systolic BP, smoking rates, depression, and anxiety.[52] The educational interventions for patients with ACS focused primarily on clinical outcomes (hospitalization and mortality), modifiable risk factors (BP, low-density lipoprotein levels, and smoking cessation), and other psychological outcomes (anxiety and depression).

Delivery, timing and follow up

Most educational sessions were delivered weekly. Few reviews provided information regarding the duration of education interventions; when the duration was reported, it varied from 4 weeks to 48 months. These findings suggest that there is a significant gap in the evidence in relation to the duration, contact hours, educational content, optimal delivery mode, total length, and setting of health education programs for cardiac patients.[43] For patients with ACS, one systematic review that included 7 studies with a total of 536 participants reported that studies with education lasting at least 6 months resulted in the most significant changes in the primary outcomes (such

For peer review only - http://bmjopen.bmj.com/site/about/guidelines.xhtml

as behavioral change, smoking cessation) [24] and that at least 12 months of follow-up is needed to evaluate the impact of telephone-based education.[52] Another review reported that the intensity of education programs is important for efficacy regarding smoking cessation: interventions with a very low intensity and brief interventions, do not have a significant effect,[73] and programs for smoking cessation among patients with coronary heart disease should last >1 month.[73] Most of the reviews were provided for patients with ACS in inpatient settings and then within post discharge settings, five reviews [24 29 38 41 52] did not explicitly state the settings in which the health education related interventions were provided.

Health Educational Interventions for Patients with T2DM

Use of theoretical orientation to develop educational intervention

Although the theoretical approach underpinning the health education program was not always described, there is evidence that health education interventions based on a theoretical model are likely to be effective.[36] Vugt et al suggested that self-care education programs should be based on theories and that theory-based self-care interventions are more effective than non-theory-based programs.[69 76] Theories could help to specify the key target health behaviors and behavioral change techniques required to generate the desired outcomes.[69] The decision regarding the theory should be based on the aim of the program and factor for intervention.[63] Only one review reported that a theoretical approach underpinning the health education program is not necessary for better outcomes.[66]

Educational content

The educational content for patients with T2DM focused more on behavioral change, diet, exercise, glycemic control, medication, and self-management. Health education that was self-management was more effective for patients with T2DM.[33 40] In addition, based on the current

For peer review only

evidence, the educational content should be culturally sensitive, especially for patients with T2DM; [26 35 47] culturally appropriate diabetes health education may have a greater impact on glycemic control and reduce diabetic complications.[63] The educational interventions for patients with T2DM focused primarily on HbA1C, lipid levels, quality of life, and body weight. HBM and SCT were the most common theories used in the included reviews.

Teaching strategies and outcomes

Mixed health education programs generally included group sessions combined with educator-facilitated individual sessions, covering basic knowledge and problem-solving skills. These programs produced greater benefits and larger effect sizes for blood glucose control and knowledge level in patients with T2DM.[40] In contrast, individual education programs have been reported as more effective in achieving outcomes than group-based education. This may be because education programs might be more efficient at addressing personal needs, with greater participant engagement.[64] Although face-to-face sessions were the most common delivery format, many education sessions were also delivered by telephone or individualized counseling. Face-to-face health education programs were most effective for enhancing blood glucose control and knowledge levels, while mixed delivery models (face-to-face, phone contact, online or web-based, or video) produced a moderate effect for knowledge levels.[40] Another review reported that face-to-face health education programs generated a greater benefit for metabolic control than those delivered using electronic communication technology.[64]

Community workers, dieticians, multidisciplinary teams, and nurses were the most frequent educators, and most of the education programs were delivered post-discharge. Some reviews indicated that health education programs delivered by a group of different educators, with some degree of education reinforcement at additional points of contact, may provide the best results.[53]

For peer review only

[66] However, based on two studies that reported HbA1C at 12 months, it was showed that the outcomes in studies with only a diabetes nurse as the educator also tended to do better than the outcomes in studies with a multidisciplinary team , while the biggest effect was seen when a dietician was the only educator.[66] Health education programs delivered by one person may focus more on the patient's ability than the educational content or quality of the health education programs.[66] However, no clear conclusion can be drawn whether having one educator delivering the intervention is best due to few information.[53]

Delivery, timing and follow up

Education sessions were delivered weekly or monthly. Longer health education programs for T2DM (>6 months) produced larger effects for all primary outcomes (such as HbA1C).[40] Health education lasting >3 months resulted in the largest effect size compared with health education of a shorter duration (<3 months).[26] For HbA1C, the effect size at 6 months seemed to be significantly greater than at 3 and 12 months; in other words, the effect size peaked at 6 months.[55] In general, health education of a greater intensity (longer duration and more sessions) was more effective for blood glucose control and knowledge level among patients with T2DM.[40 65] Compared with health education programs covering only one topic, programs that included multiple or mixed educational topics yielded consistently greater benefits in blood glucose control and knowledge levels.[40] In addition, health education programs combined with specific behavioral change strategies (such as self-care strategies) seemed more effective than other programs.[40] Health education related interventions were mainly delivered in hospital settings, primary care settings, diabetes centers or community based settings; although six reviews [25 32 48 51 60 67] did not explicitly state the delivery settings.

Health Education Interventions for Patients with ACS and T2DM

For peer review only - http://bmjopen.bmj.com/site/about/guidelines.xhtml

These results from included systematic reviews and meta-analyses help to provide recommendations about the content of a health education intervention for patients with ACS and T2DM, requiring further evaluation. Future development of educational programs for patients with ACS and T2DM by health care professionals should consider the needs of people with these diseases.[30 33 35 72] Based on the results and findings from this umbrella review, recommendations are made in Table 5.

Table 5 Recommendations of health education programs for ACS and T2DM patients

		ACS patients	T2DM patients	Both ACS and T2DM
**Theoretical approach**		TTM	HBM; SCT	HBM; SCT and TTM;
**Educational content**		Behavioral change (such as smoking cessation), cardiovascular risk factors, exercise, and psychosocial issues.	Behavioral change, diet, exercise, glycemic control, medication, and self-management.	Behavioral change (such as smoking cessation), cardiovascular risk factors, diet, exercise, glycemic control, medication, and psychosocial issues. Self- management strategies.
**Healthcare professionals to deliver**		Nurse or multidisciplinary team	Multidisciplinary team; dietitian or nurse	Nurse or multidisciplinary team
**Teaching approaches**	Strategies	Face-to-face; telephone or mixed	Face to face, written materials; telephone or mixed	Face to face, written materials; telephone contact or mixed
	Format	Individual (One by one) or mixed	Individual (One by one) or mixed	Individual (One by one) or mixed
**Delivery timing**	Contact hours	More than 30 min per time per week	More than 30 min per time per week	More than 30 min per time per week
	Duration	At least 6 months	About 6 months	At least 6 months
**Duration of follow up**		At least 12 months	At least 12 months	At least 12 months
**Settings**		Inpatient and post-discharge settings	Hospital settings and primary care settings	Inpatient and post-discharge settings

Note: ACS = acute coronary syndrome; T2DM = type 2 diabetes mellitus; TTM= Transtheoretical Model; SCT=Social Cognitive Theory; HBM =Health Belief Model.

For peer review only

Overall Completeness and Applicability of Evidence

This overview potentially provides an estimate with the lowest level of bias for the impact of health education-related interventions for patients with ACS or T2DM and could be regarded as an all-inclusive summary of the current evidence base for health education for these patients.

While this umbrella review identified evidence for each of the types of health education, there was only a small number of reviews within some categories (such as psychoeducational intervention [23] and dietary advice [56]), and these studies were not very informative. This umbrella review also found no reviews that systematically analyzed varying doses of health education; therefore, could not examine the dose-response effects. There was insufficient information about the evaluated doses (total contact hours and duration of education) to enable comparison of the benefits of differences in the magnitude of the doses across the different research. This umbrella review found no reviews focused on patients with ACS and T2DM; instead, all of the systematic reviews and meta-analyses focused on only one of these diseases.

Quality of the Evidence

The methodological quality of the included systematic reviews and meta-analyses varied. All of the included reviews or meta-analyses were of moderate to high methodological quality, as assessed using AMSTAR. However, only 30 (58.8%) systematic reviews or meta-analyses were rated as high quality and only three (5.9%) systematic reviews or meta-analyses [36 46 73] adequately met all 11 AMSTAR criteria. This indicates that some of the reviews included in this umbrella review may have limitations in their design, conduct, and/or reporting that could have influenced the findings when considered both individually and collectively.[25 58]

The quality of the primary studies in the included systematic reviews or meta-analyses also

For peer review only

varied. The main sources of bias were inadequate reporting of allocation concealment and randomization processes, as well as lack of outcome blinding.[26 35 72 73] This bias in the methodological quality led to lower quality assessments, which varied by results within each included review. Other reasons for lower methodological quality included heterogeneity in, or inconsistency of, the effect and imprecise findings. Heterogeneity between studies in this umbrella review was described in terms of the intervention, participant characteristics and length of follow-up. Heterogeneity was an important factor indicating the complexity of the health education interventions.[49] The variability in the approaches, tools or scales used to measure outcomes between the included studies are likely to introduce some heterogeneity.[23] The heterogeneity of the educational interventions seen in the reviews included in this umbrella review may reflect the uncertainty about the optimal strategy for providing health education to patients.[30]

This umbrella review is the first synthesis of systematic reviews or meta-analyses to take a broad perspective on health education-related interventions for patients with ACS or T2DM. Given that health education is complex, the biggest challenge for systematic reviews or meta-analyses of health education is accounting for the potential clinical heterogeneity in health education-related interventions (content and delivery approaches) and the population of patients who receive health education. To facilitate comparisons across systematic reviews of health education and the efficient future update of this umbrella review, future reviews or meta-analyses need high-quality research and to standardize their design and reporting, including the reporting of included study characteristics, assessment criteria for risk of bias, outcomes, and methods to synthesize evidence synthesis.

Conclusions

For peer review only - http://bmjopen.bmj.com/site/about/guidelines.xhtml

For clinicians providing educational interventions to individuals with ACS and T2DM, the results from this review provide a contemporaneous perspective on current evidence on effectiveness of health education (its content, and delivery methods) for this high risk patient group. The current evidence compiled by this umbrella review supports current international clinical guidelines, that theoretically-based education interventions lasting 6 months, delivered in multiple modes (face-to-face, phone contact, online or web-based, or video), and with individualized education delivered weekly are more likely to generate positive outcomes. This review also supports health education- related interventions provided by health professionals, including nurses only and multidisciplinary teams, and the delivery content included specific clinical factors for ACS and T2DM (BP, glycemic control, and medication), modifiable risk factors (unhealthy diet, inactivity, and smoking), and other psychological factors (anxiety and depression). These health education interventions could be delivered post-discharge, such as rehabilitation centers, primary care centers, and the community and should be at least 6 months in duration. The effectiveness of these programs was based on HbA1C levels, knowledge, psychosocial outcomes, readmission rates, and smoking status rather than clear evidence of reduced mortality and MI.

In addition, psychoeducational interventions were more effective for patients with ACS, and health education that was culturally appropriate or taught self-management was more effective for patients with T2DM. We also found that longer durations and high-intensity health education provided in an individualized format were more helpful for patients with ACS or T2DM.

The fact that none of the included reviews included patients with both ACS and T2DM indicates a clear need for further rigorous experimental studies with patients with both diseases. Future research that includes these aspects of education are likely determine the effectiveness of

For peer review only - http://bmjopen.bmj.com/site/about/guidelines.xhtml

educational interventions focusing on cardiovascular and DM risk factors and complications within patients with ACS and T2DM.

Acknowledgements: I would like to thank the authors of the original articles who provided additional unpublished data.

Author contributions: All authors contributed to study conception, design, data analysis, drafting of manuscripts, and final manuscript approval. Data extraction processes were finalized by all authors, although they were predominantly managed by Mr. Xian-Liang Liu and Professor Shi.

Conflicts of interest: None declared.

Founding statement: This research received no specific grant from any funding agency in the public, commercial or not-for-profit sectors.

Data sharing statement: No additional data are available.

For peer review only

References

1. Scarborough P, Wickramasinghe K, Bhatnagar P, et al. Trends in coronary heart disease, 1961-2011. British Heart Foundation, London 2011.
2. Finegold JA, Asaria P, Francis DP. Mortality from ischaemic heart disease by country, region, and age: statistics from World Health Organisation and United Nations. International journal of cardiology 2013;**168**(2):934-45.
3. Amsterdam EA, Wenger NK, Brindis RG, et al. 2014 AHA/ACC guideline for the management of patients with non–ST-elevation acute coronary syndromes: a report of the American College of Cardiology/American Heart Association Task Force on Practice Guidelines. Journal of the American College of Cardiology 2014;**64**(24):e139-e228.
4. Kahn M, Wheatcroft S. Acute coronary syndromes in diabetes. Heart Disease and Diabetes 2012.
5. Hasin T, Hochadel M, Gitt AK, et al. Comparison of treatment and outcome of acute coronary syndrome in patients with versus patients without diabetes mellitus. The American journal of cardiology 2009;**103**(6):772-78.
6. Franklin K, Goldberg RJ, Spencer F, et al. Implications of diabetes in patients with acute coronary syndromes: the Global Registry of Acute Coronary Events. Archives of Internal Medicine 2004;**164**(13):1457-63.
7. Ting HH, Chen AY, Roe MT, et al. Delay From Symptom Onset to Hospital Presentation for Patients With Non–ST-Segment Elevation Myocardial Infarction. Archives of internal medicine 2010;**170**(20):1834-41.
8. Saely CH, Aczel S, Koch L, et al. Diabetes as a coronary artery disease risk equivalent: before a change of paradigm? European Journal of Cardiovascular Prevention & Rehabilitation

For peer review only

2010;**17**(1):94-99.

9. Donahoe SM, Stewart GC, McCabe CH, et al. Diabetes and mortality following acute coronary syndromes. JAMA 2007;**298**(7):765-75.

10. Lakerveld J, Bot SD, Chinapaw MJ, et al. Motivational interviewing and problem solving treatment to reduce type 2 diabetes and cardiovascular disease risk in real life: a randomized controlled trial. International Journal of Behavioral Nutrition and Physical Activity 2013;**10**(1):1.

11. Glanz K, Rimer BK, Viswanath K. *Health behavior and health education: theory, research, and practice*: John Wiley & Sons, 2008.

12. Becker LA OA. Chapter 22: overviews of reviews.In: Higgins JPT, Green S (editors). Cochrane Handbook for Systematic Reviews of Interventions Version 5.1.0 [updated March 2011]. The Cochrane Collaboration 2011.

13. Chew DP, Scott IA, Cullen L, et al. National Heart Foundation of Australia and Cardiac Society of Australia and New Zealand: Australian clinical guidelines for the management of acute coronary syndromes 2016. Heart, Lung and Circulation 2016;**25**(9):895-951.

14. Alberti KGMM, Zimmet Pf. Definition, diagnosis and classification of diabetes mellitus and its complications. Part 1: diagnosis and classification of diabetes mellitus. Provisional report of a WHO consultation. Diabetic medicine 1998;**15**(7):539-53.

15. Thomson Reuters. EndNote version X7 Secondary EndNote version X7 2016. http://endnote.com/product-details/x7.

16. Shea BJ, Grimshaw JM, Wells GA, et al. Development of AMSTAR: a measurement tool to assess the methodological quality of systematic reviews. BMC medical research methodology 2007;**7**(1):1.

For peer review only

17. Sharif MO, Janjua-Sharif F, Ali H, et al. Systematic reviews explained: AMSTAR-how to tell the good from the bad and the ugly. Oral Health Dent Manag 2013;**12**(1):9-16.

18. Aromataris E, Fernandez R, Godfrey CM, et al. Summarizing systematic reviews: methodological development, conduct and reporting of an umbrella review approach. International Journal of Evidence-Based Healthcare 2015;**13**(3):132-40.

19. Ryan RE, Weir M, Leslie B, et al. Evidence on Optimal Prescribing and Medicines Use for Decision Makers: Scope and Application of the Rx for Change Database. Journal of Pharmacy Practice and Research 2011;**41**(4):295-99.

20. Sapkota S, Brien JAE, Greenfield JR, et al. A systematic review of interventions addressing adherence to anti-diabetic medications in patients with type 2 diabetes - Components of interventions. PLoS ONE 2015;**10 (6) (no pagination)**(e0128581).

21. Walker RJ, Smalls BL, Bonilha HS, et al. Behavioral interventions to improve glycemic control in African Americans with type 2 diabetes: a systematic review (Provisional abstract). Ethnicity and Disease 2013; 23(4). http://onlinelibrary.wiley.com/o/cochrane/cldare/articles/DARE-12014004933/frame.html.

22. Heinrich E, Schaper NC, de Vries NK. Self-management interventions for type 2 diabetes: a systematic review. European Diabetes Nursing 2010;**7**(2):71-76 6p.

23. Alam R, Sturt J, Lall R, et al. An updated meta-analysis to assess the effectiveness of psychological interventions delivered by psychological specialists and generalist clinicians on glycaemic control and on psychological status. Patient Educ Couns 2009;**75**(1):25-36.

24. Aldcroft SA, Taylor NF, Blackstock FC, et al. Psychoeducational Rehabilitation for Health

For peer review only

Behavior Change in Coronary Artery Disease: A Systematic review of controlled trials. Journal of Cardiopulmonary Rehabilitation & Prevention 2011;**31**(5):273-81 9p.

25. Amaeshi IJ. Exploring the impact of structured foot health education on the rate of lower extremity amputation in adults with type 2 diabetes. A systematic review. Podiatry Now 2012;**15**(6):20-27 8p.

26. Attridge M, Creamer J, Ramsden M, et al. Culturally appropriate health education for people in ethnic minority groups with type 2 diabetes mellitus. Cochrane Database of Systematic Reviews 2014;**9**:CD006424.

27. Auer R, Gaume J, Rodondi N, et al. Efficacy of in-hospital multidimensional interventions of secondary prevention after acute coronary syndrome a systematic review and meta-analysis. Circulation 2008;**117**(24):3109-17.

28. Barth J, Critchley J, Bengel J. Efficacy of psychosocial interventions for smoking cessation in patients with coronary heart disease: A systematic review and meta-analysis. Annals of Behavioral Medicine 2006;**32**(1):10-20.

29. Barth J, Critchley JA, Bengel J. Psychosocial interventions for smoking cessation in patients with coronary heart disease. The Cochrane Library 2008.

30. Brown JP, Clark AM, Dalal H, et al. Effect of patient education in the management of coronary heart disease: A systematic review and meta-analysis of randomized controlled trials. European Journal of Preventive Cardiology 2013;**20**(4):701-14.

31. Burke E, Robert, Ferrara A, et al. The effectiveness of group medical visits on diabetes mellitus type 2 (dm2) specific outcomes in adults: a systematic review, 2011.

32. Chen L, Pei JH, Kuang J, et al. Effect of lifestyle intervention in patients with type 2 diabetes: A meta-analysis. Metabolism-Clinical and Experimental 2015;**64**(2):338-47.

For peer review only - http://bmjopen.bmj.com/site/about/guidelines.xhtml

33. Choi TST, Davidson ZE, Walker KZ, et al. Diabetes education for Chinese adults with type 2 diabetes: A systematic review and meta-analysis of the effect on glycemic control. Diabetes Research and Clinical Practice 2016;**116**:218-29.

34. Clark AM, Hartling L, Vandermeer B, et al. Meta-analysis: secondary prevention programs for patients with coronary artery disease (Structured abstract). Annals of Internal Medicine 2005; 143(9). http://onlinelibrary.wiley.com/o/cochrane/cldare/articles/DARE-12005008551/frame.html.

35. Creamer J, Attridge M, Ramsden M, et al. Culturally appropriate health education for Type 2 diabetes in ethnic minority groups: An updated Cochrane Review of randomized controlled trials. Diabetic Medicine 2016;**33**(2):169-83.

36. Deakin T, McShane CE, Cade JE, et al. Group based training for self-management strategies in people with type 2 diabetes mellitus. Cochrane Database of Systematic Reviews 2005(2):CD003417.

37. Devi R, Singh Sally J, Powell J, et al. Internet-based interventions for the secondary prevention of coronary heart disease. Cochrane Database of Systematic Reviews 2015; (12). http://onlinelibrary.wiley.com/doi/10.1002/14651858.CD009386.pub2/abstract.

38. Dickens C, Cherrington A, Adeyemi I, et al. Characteristics of psychological interventions that improve depression in people with coronary heart disease: A systematic review and meta-regression. Psychosomatic Medicine 2013;**75**(2):211-21.

39. Duke SA, Colagiuri S, Colagiuri R. Individual patient education for people with type 2 diabetes mellitus. Cochrane Database of Systematic Reviews 2009(1):CD005268.

40. Fan L, Sidani S. Effectiveness of diabetes self-management education intervention elements: a meta-analysis. Canadian Journal of Diabetes 2009;**33**(1):18-26 9p.

For peer review only

41. Fernandez R, Griffiths R, Everett B, et al. Effectiveness of brief structured interventions on risk factor modification for patients with coronary heart disease: a systematic review, 2007.

42. Gary TL, Genkinger JM, Guallar E, et al. Meta-analysis of randomized educational and behavioral interventions in type 2 diabetes (Structured abstract). Diabetes Educator 2003; 29(3). http://onlinelibrary.wiley.com/o/cochrane/cldare/articles/DARE-12003001309/frame.html.

43. Ghisi GLD, Abdallah F, Grace SL, et al. A systematic review of patient education in cardiac patients: Do they increase knowledge and promote health behavior change? Patient Education and Counseling 2014;**95**(2):160-74.

44. Goulding L, Furze G, Birks Y. Randomized controlled trials of interventions to change maladaptive illness beliefs in people with coronary heart disease: systematic review. Journal of Advanced Nursing 2010;**66**(5):946-61 16p.

45. Gucciardi E, Chan VWS, Manuel L, et al. A systematic literature review of diabetes self-management education features to improve diabetes education in women of Black African/Caribbean and Hispanic/Latin American ethnicity. Patient Education and Counseling 2013;**92**(2):235-45.

46. Hawthorne K, Robles Y, Cannings-John R, et al. Culturally appropriate health education for type 2 diabetes mellitus in ethnic minority groups. Cochrane Database of Systematic Reviews 2008(3):CD006424.

47. Hawthorne K, Robles Y, Cannings-John R, et al. Culturally appropriate health education for type 2 diabetes in ethnic minority groups: a systematic and narrative review of randomized controlled trials. Diabetic Medicine 2010; 27(6).

For peer review only

http://onlinelibrary.wiley.com/o/cochrane/cldare/articles/DARE-12010004372/frame.html.

48. Huang XL, Pan JH, Chen D, et al. Efficacy of lifestyle interventions in patients with type 2 diabetes: A systematic review and meta-analysis. European Journal of Internal Medicine 2016;**27**:37-47.

49. Huttunen-Lenz M, Song F, Poland F. Are psychoeducational smoking cessation interventions for coronary heart disease patients effective? Meta-analysis of interventions. British journal of health psychology 2010;**15**(Pt 4):749-77.

50. Kai Lun Gan J, Brammer JD, Creedy DK. Effectiveness of educational interventions to promote oral hypoglycaemic adherence in adults with Type 2 diabetes: a systematic review. JBI Library of Systematic Reviews 2011;**9**(9):269-312 44p.

51. Khunti K, Camosso-Stefinovic J, Carey M, et al. Educational interventions for migrant South Asians with type 2 diabetes: a systematic review (Structured abstract). Diabetic Medicine 2008; 25(8). http://onlinelibrary.wiley.com/o/cochrane/cldare/articles/DARE-12009100341/frame.html.

52. Kotb A, Hsieh S, Wells GA. The effect of telephone support interventions on coronary artery disease (CAD) patient outcomes during cardiac rehabilitation: a systematic review and meta-analysis. PLoS One 2014;**9**(5):e96581.

53. Loveman E, Frampton GK, Clegg AJ. The clinical effectiveness of diabetes education models for type 2 diabetes: a systematic review (Structured abstract). Health Technology Assessment 2008; 12(9). http://onlinelibrary.wiley.com/o/cochrane/cldare/articles/DARE-12008104815/frame.html.

54. Minet L, Moller S, Vach W, et al. Mediating the effect of self-care management intervention

For peer review only

in type 2 diabetes: a meta-analysis of 47 randomised controlled trials (Structured abstract). Patient Education and Counseling 2010; 80(1). http://onlinelibrary.wiley.com/o/cochrane/cldare/articles/DARE-12010005378/frame.html.

55. Nam S, Janson SL, Stotts NA, et al. Effect of culturally tailored diabetes education in ethnic minorities with type 2 diabetes: a meta-analysis (Provisional abstract). Journal of Cardiovascular Nursing 2012; 27(6). http://onlinelibrary.wiley.com/o/cochrane/cldare/articles/DARE-12012056817/frame.html.

56. Nield L, Moore H, Hooper L, et al. Dietary advice for treatment of type 2 diabetes mellitus in adults. Cochrane Database of Systematic Reviews 2007; (3). http://onlinelibrary.wiley.com/doi/10.1002/14651858.CD004097.pub4/abstract.

57. Norris SL, Engelgau MM, Narayan KM. Effectiveness of self management training in type 2 diabetes: a systematic review of randomized controlled trials (Structured abstract). Diabetes Care 2001; 24(3). http://onlinelibrary.wiley.com/o/cochrane/cldare/articles/DARE-12001000796/frame.html.

58. Norris SL, Lau J, Smith SJ, et al. Self-management education for adults with type 2 diabetes: a meta-analysis of the effect on glycemic control (Structured abstract). Diabetes Care 2002; 25(7). http://onlinelibrary.wiley.com/o/cochrane/cldare/articles/DARE-12002005529/frame.html.

59. Odnoletkova I, Goderis G, Pil L, et al. Cost-effectiveness of therapeutic education to prevent the development and progression of type 2 diabetes: Systematic review. Journal of

For peer review only

Diabetes & Metabolism 2014;**2014**.

60. Pal K, Eastwood SV, Michie S, et al. Computer-based interventions to improve self-management in adults with type 2 diabetes: a systematic review and meta-analysis (Structured abstract). Database of Abstracts of Reviews of Effects 2014; (2). http://onlinelibrary.wiley.com/o/cochrane/cldare/articles/DARE-12014034836/frame.html.

61. Pal K, Eastwood SV, Michie S, et al. Computer-based diabetes self-management interventions for adults with type 2 diabetes mellitus. Cochrane Database of Systematic Reviews 2013;**3**:CD008776.

62. Pillay J, Armstrong MJ, Butalia S, et al. Behavioral Programs for Type 2 Diabetes Mellitus: A Systematic Review and Network Meta-analysis. Annals of Internal Medicine 2015;**163**(11):848-60.

63. Ramadas A, Quek KF, Chan CK, et al. Web-based interventions for the management of type 2 diabetes mellitus: a systematic review of recent evidence. International Journal of Medical Informatics 2011;**80**(6):389-405.

64. Ricci-Cabello I, Ruiz-Perez I, Rojas-Garcia A, et al. Characteristics and effectiveness of diabetes self-management educational programs targeted to racial/ethnic minority groups: a systematic review, meta-analysis and meta-regression. BMC Endocr Disord 2014;**14**:60.

65. Saffari M, Ghanizadeh G, Koenig HG. Health education via mobile text messaging for glycemic control in adults with type 2 diabetes: a systematic review and meta-analysis (Provisional abstract). Database of Abstracts of Reviews of Effects 2014; (2). http://onlinelibrary.wiley.com/o/cochrane/cldare/articles/DARE-

For peer review only

12014031070/frame.html.

66. Steinsbekk A, Rygg LO, Lisulo M, et al. Group based diabetes self-management education compared to routine treatment for people with type 2 diabetes mellitus. A systematic review with meta-analysis (Structured abstract). BMC Health Services Research 2012; 12(2). http://onlinelibrary.wiley.com/o/cochrane/cldare/articles/DARE-12013000409/frame.html.

67. Terranova CO, Brakenridge CL, Lawler SP, et al. Effectiveness of lifestyle-based weight loss interventions for adults with type 2 diabetes: a systematic review and meta-analysis. Diabetes, Obesity & Metabolism 2015;**17**(4):371-8.

68. Vermeire E, Wens J, Van Royen P, et al. Interventions for improving adherence to treatment recommendations in people with type 2 diabetes mellitus. Cochrane Database of Systematic Reviews 2005(2):CD003638.

69. Vugt M, Wit M, Cleijne WH, et al. Use of behavioral change techniques in web-based self-management programs for type 2 diabetes patients: systematic review (Provisional abstract). Journal of Medical Internet Research 2013; 15(12). http://onlinelibrary.wiley.com/o/cochrane/cldare/articles/DARE-12014000476/frame.html.

70. Wens J, Vermeire E, Hearnshaw H, et al. Educational interventions aiming at improving adherence to treatment recommendations in type 2 diabetes: A sub-analysis of a systematic review of randomised controlled trials. Diabetes Res Clin Pract 2008;**79**(3):377-88.

71. Zabaleta AM, Forbes A. Structured group-based education for type 2 diabetes in primary care (Provisional abstract). British Journal of Community Nursing 2007; 12(4).

For peer review only

http://onlinelibrary.wiley.com/o/cochrane/cldare/articles/DARE-12007009143/frame.html.

72. Brown JP, Clark AM, Dalal H, et al. Patient education in the management of coronary heart disease. Cochrane Database of Systematic Reviews 2011(12):CD008895.

73. Barth J, Jacob T, Daha I, et al. Psychosocial interventions for smoking cessation in patients with coronary heart disease. Cochrane Database of Systematic Reviews 2015; (7). http://onlinelibrary.wiley.com/doi/10.1002/14651858.CD006886.pub2/abstract.

74. Albrecht L, Archibald M, Arseneau D, et al. Development of a checklist to assess the quality of reporting of knowledge translation interventions using the Workgroup for Intervention Development and Evaluation Research (WIDER) recommendations. Implementation Science 2013;**8**(1):52.

75. Glazier RH, Bajcar J, Kennie NR, et al. A systematic review of interventions to improve diabetes care in socially disadvantaged populations. Diabetes care 2006;**29**(7):1675-88.

76. Funnell MM, Brown TL, Childs BP, et al. National standards for diabetes self-management education. Diabetes care 2009;**32**(Supplement 1):S87-S94.

For peer review only

Table 2. Characteristics and interventions of included systematic reviews and meta-analysis involved ACS patients

(First author, Year); Journal	Primary objectives (To assess effect of interventions on….)	Studies details	Intervention						Outcomes (Primary outcomes were bold.) "–" : No change "↑" : Increase "↓" : decrease	Synthesis methods
			Educational Content		Provider	Number of session(s), delivery mode, time, setting				
(Devi et al., 2015)[37]; The Cochrane Library	Lifestyle changes and medicines management	**Number of studies**:11 completed trials (12 publications);<br>**Types of studies:** RCTs;<br>**Total sample:** 1,392 participants.	All Internet based interventions.	√ BEHA (-)<br>√ CVR (-)<br>√ DIET (-)<br>√ EXERCISE (-)<br>☐ MED<br>√ PSY(-)<br>√ SMOKING (-)<br>☐ SELF	Dietitians; exercise specialists; nurse practitioners; physiotherapist rehabilitation specialists, or did not describe.	**Number of session:** Weekly or monthly or unclear;<br>**Total contact hours:** Unclear.<br>**Duration:** From six weeks to one year.	**Strategies:** Internet- and mobile phone-based intervention, such as email access, private-messaging function on the website, one-to-one chat facility, a synchronised group chat, an online discussion forum, or telephone consultations; or video files;<br>**Format:** one-on-one chat sessions; "ask an expert" group chat sessions;<br>**Theoretical approach:** Unclear.	Inpatient settings, post discharge, other	**– Clinical outcomes;**<br>**– Cardiovascular risk factors;**<br>– Lifestyle changes;<br>– Compliance with medication;<br>– Healthcare utilization and costs;<br>↓ Adverse intervention effects.	Meta-analysis used Review Manager software.
(Barth, Jacob, Daha, & Critchley, 2015)[73]; The Cochrane Library	Smoking cessation	**Number of studies**:40 RCTs;<br>**Types of studies:** RCTs;<br>**Total sample:** 7,928 participants.	Psychosocial smoking cessation interventions	☐ BEHA<br>☐ CVR<br>☐ DIET<br>☐ EXERCISE<br>☐ MED<br>☐ PSY<br>√ SMOKING<br>☐ SELF	Cardiologist; general practitioner physician or study nurse.	**Number of session**: Weekly or 2-3 times per week;<br>**Total contact hours:** Unclear.<br>**Duration:** From 8 weeks to one year.	**Strategies:** Face to face, telephone contact, written educational materials, videotape, booklet or unclear;<br>**Format:** One by one counseling; telephone call; group meetings or unclear;<br>**Theoretical approach:** TTM, SCT.	Inpatient settings, post discharge, other	**↑ Abstinence by self-report or validated.**	Meta-analysis used Review Manager software.
(Kotb, Hsieh, & Wells, 2014)[52]; PloS one	Patients' outcomes.	**Number of studies**: 26 studies;<br>**Types of studies:** RCTs;<br>**Total sample:** 4,081	Telephone-delivered post-discharge interventions.	☐ BEHA<br>√ CVR<br>☐ DIET<br>☐ EXERCISE<br>☐ MED	Dietitians; exercise specialist; health educators; nurses and pharmacists.	**Number of session:** 3-6 sessions/telephone calls and was greater than 6 calls in five	**Strategies:** Telephone calls;<br>**Format:** Unclear, did not describe the format;<br>**Theoretical approach:** Unclear.	Unclear, did not describe the setting.	**↓ All-cause hospitalization;**<br>**– All-cause mortality;**<br>↓ Depression;	Meta-analysis used Review Manager software.

For peer review only

		participants.		☐PSY<br>☐SMOKING<br>☐SELF		studies; or unclear;<br>**Total contact hours:** 40 mins to 180 mins or unclear;<br>**Duration:** 1.5 - 6 months or unclear.			− Anxiety;<br>↑ Smoking cessation,<br>↓ Systolic blood pressure;<br>− LDL-c.	
(Ghisi, Abdallah, Grace, Thomas, & Oh, 2014) [43]; Patient Education and Counseling	knowledge, health behavior change, medication adherence, psychosocial well-being.	**Number of studies**: 42 articles;<br>**Types of studies:** Thirty were experimental: 23 RCTs and 7 quasi-experimental; and 11 observational and 1 used a mixed-methods design.<br>**Total sample:** 16,079 participants.	Any educational interventions.	√ BEHA (+)<br>√ CVR (++)<br>√ DIET (+++)<br>√ EXERCISE (++)<br>√ MED (++)<br>√ PSY(++)<br>√ SMOKING (+)<br>☐ SELF	Nurses (35.7%), a multidisciplinary team (31%), dietitians (14.3%), and a cardiologist (2.4%).	**Number of session:** 1-24 or unclear.<br>**Total contact hours:** 5–10 min to 3 h as well as a full day of education.<br>**Duration:** 1-24 month; from daily education to every 6 months.	**Strategies:** Did not describe the strategies;<br>**Format:** Group (88.1%) education was delivered by lectures (40.5%), group discussions (40.5%), and question and answer periods (7.1%). Individual education (88.1%), including individual counseling (50%), follow-up telephone contacts (31%) and home visits (7.1%);<br>**Theoretical approach:** Unclear.	Inpatient settings	**− Knowledge,**<br>**− Behavior;**<br>**− Psychosocial indicators.**	Narrative synthesis
(Brown, Clark, Dalal, Welch, & Taylor, 2013) [30]; European Journal of Preventive Cardiology	Mortality, morbidity, HRQoL and healthcare costs.	**Number of studies:** Twenty-four papers reporting on 13 RCTs;<br>**Types of studies:** RCTs;<br>**Total sample:** 68,556 participants.	Patient education.	☐BEHA<br>√ CVR<br>☐DIET<br>☐EXERCISE<br>☐MED<br>☐PSY<br>☐SMOKING<br>☐SELF	Nurses or other healthcare professionals.	**Number of session and Duration:** From a total of 2 visits to a 4-week residential stay reinforced with 11 months of nurse led follow-up.<br>**Total contact hours:** Unclear.	**Strategies:** Face-to-face education sessions, telephone contact and interactive use of the Internet;<br>**Format:** Group-based sessions, individualized education and four utilized a mixture of both sessions;<br>**Theoretical approach:** Unclear.	Inpatient settings, other	**− Mortality,**<br>**− Non-fatal MI,**<br>**− Revascularizations,**<br>**− Hospitalizations,**<br>**− HRQoL,**<br>− Withdrawals/drop outs;<br>− Healthcare utilization and costs.	Meta-analysis used Review Manager software.
(Dickens et al., 2013) [38]; Psychosomatic Medicine	Depression and depressive symptoms.	**Number of studies:** Sixty-two independent studies.<br>**Types of studies:** RCTs;<br>**Total sample:** 17,397.	Psychological interventions.	√ BEHA (-)<br>☐CVR<br>☐DIET<br>☐EXERCISE<br>☐MED<br>√ PSY (-)<br>☐SMOKING	A single health professional or by a unidisciplinary team	**Number of session:** 14.4 (range, 1-156);<br>**Total contact hours:** Varying from 10 to 240 minutes.<br>**Duration:** Unclear.	**Strategies:** Face-to-face sessions, telephone contact or unclear;<br>**Format:** Group or unclear;<br>**Theoretical approach:** Unclear.	Unclear, did not describe.	**↓ Depression;**<br>− Adverse cardiac outcomes;<br>− Ongoing cardiac symptoms.	Univariate analyses using Comprehensive Meta-Analysis, multivariate

For peer review only

				√ SELF (-)						meta regression using SPSS 15.0
(Aldcroft, Taylor, Blackstock, & O'Halloran P, 2011) [14]; Journal of Cardiopulmonary Rehabilitation & Prevention	Health behavior change.	**Number of studies:** 7 trials. **Types of studies:** Six randomized controlled trials and a quasi-experimental trial. **Total sample:** 536 participants.	All psychoeducational or behavioral intervention.	☐ BEHA<br>√ CVR (-)<br>☐ DIET<br>☐ EXERCISE<br>☐ MED<br>√ PSY (-)<br>☐ SMOKING<br>☐ SELF	Appropriately trained health care workers.	**Number of session:** Unclear; **Total contact hours:** Unclear; **Duration:** 2-12 months.	**Strategies:** Did not describe the strategies; **Format:** Group setting, combination of group and one-on-one education and one-on-one format only; **Theoretical approach:** TTM, interactionist role theory, Bandura's self-efficacy theory, Gordon's relapse prevention model and a cognitive behavioral approach.	Unclear, did not describe.	↓ **Smoking rates; medication use;**<br>− **Supplemental oxygen use;**<br>↑ **Physical activity;**<br>↑ Nutritional habits.	Meta-analysis and narrative presentation
(Brown, Clark, Dalal, Welch, & Taylor, 2011) [72]; The Cochrane Library	Mortality, morbidity, HRQofL and healthcare costs.	**Number of studies:** 24 papers reporting on 13 studies. **Types of studies:** RCTs; **Total sample:** 68,556 participants.	Patient education.	√ BEHA (-)<br>√ CVR (-)<br>☐ DIET<br>√ EXERCISE (-)<br>☐ MED<br>☐ PSY<br>☐ SMOKING<br>☐ SELF	Nurse or did not describe.	**Number of session and Duration:** two 2 visits to 4 week residential 11 months of nurse led folluw up **Total contact hours:** Unclear.	**Strategies:** face-to-face sessions, telephone contact and interactive use of the Internet; **Format:** Four studies involved group sessions, five involved individualized education and three utilized both session types, with one study comparing the two approaches; **Theoretical approach:** did not describe.	Post discharge, other	− **Total mortality;**<br>− **Cardiovascular**<br>− **mortality;**<br>− **Non-cardiovascular mortality;**<br>− **Total cardiovascular (CV) events;**<br>− **Fatal and/or non-fatal MI;**<br>− Other fatal and/or non-fatal CV events	Meta-analysis used Review Manager software.
(Goulding, Furze, & Birks, 2010) [44]; Journal of Advanced Nursing	Change maladaptive illness.	**Number of studies: 13 studies; Types of studies:** RCTs; **Total sample:** Unclear.	Interventions to change maladaptive illness beliefs	√ BEHA (-)<br>☐ CVR<br>☐ DIET<br>☐ EXERCISE<br>☐ MED<br>√ PSY (-)<br>☐ SMOKING	Cardiologist, nurse, psychologist or did not describe.	**Number of session:** Unclear; **Total contact hours:** Unclear; **Duration:** 4 days to two weeks or unclear.	**Strategies:** Face-to-face sessions, telephone contact and written self-administered; **Format:** Unclear; **Theoretical approach:** Common Sense Model, Leventhal's framework.	Inpatient settings, post discharge, other	− **Beliefs (or other illness cognition);**<br>− QoL;<br>− Behaviour;<br>− Anxiety or depression;<br>− Psychological	A descriptive data synthesis.

For peer review only - http://bmjopen.bmj.com/site/about/guidelines.xhtml

				☐ SELF					wellbeing;<br>– Modifiable risk factors; protective factors.	
(Huttunen-Lenz, Song, & Poland, 2010) [49]; British Journal of Health Psychology	Smoking cessation.	**Number of studies:** A total of 14 studies were included.<br>**Types of studies:** RCTs;<br>**Total sample:** 1,792 participants.	Psychoeducational cardiac rehabilitation intervention.	☐ BEHA<br>☐ CVR<br>☐ DIET<br>☐ EXERCISE<br>☐ MED<br>☐ PSY<br>√ SMOKING (-)<br>☐ SELF	Cardiologist, nurse psychologist, or did not describe.	**Number of session:** 4-20 or unclear.<br>**Total contact hours:** 10-720 mins or unclear.<br>**Duration:** 4-29 weeks or unclear.	**Strategies:** Face-to-face counselling, self-help materials; home visit, booklet, video and telephone contact.<br>**Format:** individual or unclear.<br>**Theoretical approach:** Social learning theory; ASE model; TTM; Behavioural multicomponent approach.	Inpatient settings, post discharge, other	↑ **Prevalent smoking cessation,**<br>↑ **Continuous smoking cessation,**<br>– Mortality.	Subgroup meta-analysis was used software.
(Auer, Gaume, Rodondi, Cornuz, & Ghali, 2008) [27]; Circulation	Multiple cardiovascular risk factors and all-cause mortality.	**Number of studies:** 27 articles reporting 26 studies.<br>**Types of studies:** 16 clinical controlled trials and 10 before-after studies.<br>**Total sample:** 2,467 patients in CCTs and 38, 581 patients in before-after studies.	In-Hospital Multidimensional Interventions of Secondary Prevention	☐ BEHA<br>☐ CVR<br>√ DIET (-)<br>√ EXERCISE (-)<br>☐ MED<br>√ PSY (-)<br>√ SMOKING (-)<br>☐ SELF	Cardiac nurses; physician, or did not describe.	**Number of session:**1-5 or unclear;<br>**Total contact hours:** 30-240mins or unclear;<br>**Duration:** 4 weeks – 12 months.	**Strategies:** Written material; audiotapes; presentations; face-to-face;<br>**Format:** Group or unclear;<br>**Theoretical approach:** Unclear.	Inpatient settings.	↓ **All-cause mortality;**<br>↓ **Readmission rates;**<br>**– Reinfarction rates.**	Stata version 9.1.
(Barth, Critchley, & Bengel, 2008) [29]; The Cochrane Library	Smoking cessation.	**Number of studies:** Forty trials;<br>**Types of studies:** RCTs;<br>**Total sample:** 7,682 patients.	Psychosocial intervention	√ BEHA (+++)<br>√ CVR (++)<br>☐ DIET<br>☐ EXERCISE<br>☐ MED<br>√ PSY (+)<br>√ SMOKING (+++)	Cardiologist, nurse, physician or study nurse.	**Number of session:** 1-5 or unclear;<br>**Total contact hours:** 15 mins - 9 hours.<br>**Duration:** within 4 weeks or did not report on the duration.	**Strategies:** Face to face; information booklets, audio- or videotapes.<br>**Format:** Group sessions or individual counselling;<br>**Theoretical approach:** TTM.	Inpatient settings	↑ **Abstinence by self-report or validated.**	Meta-analysis used Review Manager software.

For peer review only

				√ SELF(+++)						
(Fernandez et al., 2007) [41]; International Journal of Evidence-Based Healthcare	Risk factor modification .	**Number of studies:** Seventeen trials;<br>**Types of studies:** randomised, quasi-RCTs and clustered trials;<br>**Total sample:** 4,725 participants.	Brief structured intervention.	√ BEHA (-)<br>√ CVR (-)<br>☐ DIET<br>☐ EXERCISE<br>☐ MED<br>☐ PSY<br>☐ SMOKING<br>√ SELF (-)	Case manager; dieticians; health educator; nurses; psychologist; and research assistants.	**Number of session:** supportive counselling ranged from 1 to 7 calls for the duration of the study;<br>**Total contact hours:** varied from 10 to 30 mins;<br>**Duration:** Unclear.	**Strategies:** Written, visual, audio, telephone contact;<br>**Format:** did not describe;<br>**Theoretical approach:** theoretical behaviour change principles.	Unclear, did not describe.	↓ **Smoking;**<br>− **Cholesterol level;**<br>− **Physical activity;**<br>↑ **Dietary habits;**<br>↓ **Blood sugar levels;**<br>− **BP levels;**<br>↓ **BMI;**<br>− Incidence of admission.	Cochrane statistical package Review Manager.
(J. Barth, Critchley, & Bengel, 2006) [28]; Annals of Behavioral Medicine	Smoking cessation.	**Number of studies:** Nineteen trials;<br>**Types of studies:** RCTs;<br>**Total sample:** 2,548 patients.	Psychosocial interventions.	√ BEHA (+++)<br>√ CVR (++)<br>☐ DIET<br>☐ EXERCISE<br>☐ MED<br>☐ PSY<br>☐ SMOKING<br>√ SELF (+++)	Unclear, did not describe.	**Number of session:** Unclear;<br>**Total contact hours:** Unclear;<br>**Duration:** Unclear.	**Strategies:** Face to face, telephone contact or unclear;<br>**Format:** Unclear;<br>**Theoretical approach:** Unclear.	Unclear, did not describe.	↑ **Abstinence;**<br>↓ **Smoking status**.	Data analyses were carried out in Review Manager 4.2.
(Clark, Hartling, Vandermeer, & McAlister, 2005) [34]; Annals of Internal Medicine	Mortality, MI	**Number of studies:** 63 randomized trials;<br>**Types of studies:** RCTs;<br>**Total sample:** 21,295 patients.	Secondary prevention programs	☐ BEHA<br>☐ CVR<br>√ DIET (-)<br>√ EXERCISE (-)<br>☐ MED<br>√ PSY (-)<br>☐ SMOKING<br>☐ SELF	Nurse, multidisciplinary team, or did not describe.	**Number of session:** 1-12 or unclear;<br>**Total contact hours:** did not describe.<br>**Duration:** 0.75-48 months.	**Strategies:** Face to face, telephone contact and home visit;<br>**Format:** Group and individual or unclear;<br>**Theoretical approach:** Unclear.	Inpatient settings, post discharge, other	↓ **Mortality,**<br>↓ **MI,**<br>− Hospitalization rates.	Performed analyses by using Review Manager 4.2 and Qualitative Data Synthesis

NOTE:

For peer review only

SMOKING= Smoking cessation; CVR= Cardiovascular risk factors; PSY =Psychosocial issues (depression, anxiety); DIET =Diet; EXERCISE= Exercise; MED= Medication; BEHA = Behavioral charge (including lifestyle modification); SELF = Self-management (including problems solving); GC= glycemic control; DR =Diabetes risks; CHD= Coronary heart disease; CAD = Coronary Artery Disease; CHW=Community Health Worker; HbA1c = Glycatedhemoglobin; BP =Blood Pressure; LDL = Low-density Lipoprotein Cholesterol; SMS = Short Message Service; BCTs = Behavioral Change Techniques; LEA = Lower Extremity Amputation; PRIDE = Problem Identification, Researching one's routine, Identifying a management goal, Developing a plan to reach it, Expressing one's reactions and Establishing rewards for making progress; ASE = Attitude-social influence-efficacy; CVRF= Cardiovascular Risk Factors; SMS = Short Message System; PA = Physical Activity; EDU = Patient Education; GP= General Practice; RCTs =Randomized controlled trials; CCTS = controlled clinical trials; HRQoL = Health Related Quality of Life; QoL = Quality of Life; MI = Myocardial Infarction; CAD = Coronary Artery Disease; CABG= Coronary Artery Bypass Graft Surgery; BMI = Body Mass Index; HbA1c = Glycatedhemoglobin; BP =Blood Pressure; SBP = Systolic Blood Pressure, DBP = Diastolic Blood Pressure, HDL-c= High-density Lipoprotein Cholesterol; LDL-c = Low-density Lipoprotein Cholesterol; TTM= Transtheoretical Model; SCT=Social Cognitive Theory; HBM =Health Belief Model; SAT=Social Action Theory;

In the educational content: “+”: minor focus; “++”:moderate focus; “+++” major focus; “-“ =unclear what the intensity of the education was for any topic;

In the outcomes: arrow up (“ ↑ ”) for improvement, arrow down (“ ↓ ”) for reduction; a dash (“−”) for no change or inconclusive evidence. Primary outcomes were bold.

For peer review only

Table 3. Characteristics and interventions of included systematic reviews and meta-analysis involved T2DM patients

(First author, Year); Journal	Primary objectives (To assess effect of interventions on….)	Studies details	Intervention						Outcomes (Primary outcomes were bold.) "−" : No change "↑" : Increase "↓" : decrease	Synthesis methods
			Educational Content		Provider	Number of session(s), delivery mode, time, setting				
(Choi, Davidson, Walker, Lee, & Palermo, 2016) [33]; Diabetes Research and Clinical Practice	Glycemic effect	**Number of studies:** 53 studies(five in English, 48 in Chinese); **Types of studies:** RCTs; **Total sample:** Unclear.	Diabetes education intervention.	☐ BEHA<br>√ DIET (-)<br>☐ DR<br>☐ EXERCISE<br>☐ GC<br>☐ MED<br>☐ PSY<br>☐ SMOKING<br>√ SELF (-)	Unclear, did not describe.	**Number of session:** Unclear; **Total contact hours:** Unclear; **Duration:** 30–150 min or unclear.	**Strategies:** Face to face, written materials; telephone contact and home visit; **Format:** Unclear; **Theoretical approach:** Unclear.	Inpatient settings, post discharge, other	↓ **HbA1c (glycated haemoglobin).**	STATAversion 12 and Review Manager version 5.3
(Creamer, Attridge, Ramsden, Cannings-John, & Hawthorne, 2016) [35]; Diabetic Medicine	successful outcomes and to suggest directions for future research.	**Number of studies:** **33;** **Types of studies:** RCTs; **Total sample:** 7,453 participants.	Culturally appropriate health education.	√ BEHA (-)<br>√ DIET (-)<br>√ DR (-)<br>√ EXERCISE<br>☐ GC<br>☐ MED<br>☐ PSY<br>☐ SMOKING<br>√ SELF (-)	CHWs, clinical pharmacists dieticians, Nurses, podiatrists, physiotherapists, and psychologists.	**Number of session:** one to ten or unclear; **Total contact hours:** unclear; **Duration:** from a single session to 24 months.	**Strategies:** Face-to-face; Phone contact; **Format:** group sessions (10 studies), individual sessions (13) or a combination of both; **Theoretical approach:** Unclear.	Inpatient settings, post discharge, other	↓ **HbA1c,**<br>− **HRQoL,**<br>− **Adverse events,**<br>− BP,<br>− BMI,<br>− Lipid levels,<br>− diabetes complications,<br>− Economic analyses,<br>− Mortality and diabetes knowledge,<br>− Empowerment,<br>− Self-efficacy and satisfaction.	Meta-analysis using the Review Manager statistical program.

For peer review only

(Huang et al., 2016)[48]; European Journal of Internal Medicine	Clinical markers of cardiovascular disease	**Number of studies:** 17 studies;<br>**Types of studies:** RCTs;<br>**Total sample:** Unclear.	Lifestyle interventions.	☐ BEHA<br>√ DIET (-)<br>√ CVR (-)<br>√ EXERCISE (-)<br>☐ GC<br>☐ MED<br>☐ PSY<br>☐ SMOKING<br>√ SELF (-)	Nurse, Pharmacist or unclear.	**Number of session:** Unclear;<br>**Total contact hours:** Unclear;<br>**Duration:** 6 month – 8 years.	**Strategies:** Unclear;<br>**Format:** Individual; Group and Mixed.<br>**Theoretical approach:** Unclear;	Unclear, did not describe.	**Cardiovascular risk factors** (such as,<br>− BMI,<br>↓ HbA1c,<br>− BP,<br>↓ The level of cholesterol.)	Review Manager 5.1.
(Chen et al., 2015)[32]; Metabolism-Clinical and Experimental	Clinical markers.	**Number of studies:** 16 studies;<br>**Types of studies:** RCTs;<br>**Total sample:** per study ranged from 23 to 2575;	Lifestyle intervention.	√ BEHA (-)<br>☐ DIET<br>√ CVR (-)<br>☐ EXERCISE<br>☐ GC<br>√ MED (-)<br>☐ PSY<br>☐ SMOKING<br>√ SELF (-)	Unclear, did not describe.	**Number of session:** Monthly;<br>**Total contact hours:** Unclear;<br>**Duration:** < 6 months -8 years.	**Strategies:** Unclear;<br>**Format:** Individual; Group and Mixed;<br>**Theoretical approach:** Unclear.	Unclear, did not describe.	**Cardiovascular risk factors** including<br>↓ BMI,<br>↓ HbA1c,<br>↓ SBP, DBP,<br>− HDL-c, and LDL-c.	All analyses were performed using Comprehensive Meta-Analysis statistical software.
(Terranova, Brakenridge, Lawler, Eakin, & Reeves, 2015)[67]; Diabetes, Obesity and Metabolism	Weight loss.	**Number of studies:** 10 individual studies (from 13 papers);<br>**Types of studies:** RCTs;<br>**Total sample:** ranging from 27 to 5145 participants.	Lifestyle-based-only intervention.	√ BEHA (-)<br>√ DIET (-)<br>☐ DR (-)<br>√ EXERCISE (-)<br>☐ GC<br>☐ MED<br>☐ PSY<br>☐ SMOKING<br>√ SELF (-)	Dietician; diabetes educator; general physician; multidisciplinary team; or nutritionist; nurse.	**Number of session:** 1-42;<br>**Total contact hours:** Unclear;<br>**Duration:** ranged from 16 weeks to 9 years.	**Strategies and Format:** Face-to-face individual or group-based sessions, or a combination of those. One study delivered the intervention via the telephone<br>**Theoretical approach:** Unclear.	Unclear, did not describe.	↓ **Weight change;**<br>− **HbA1c.**	Meta-analyses - Review Manager and meta-regression analysis - stata version
(Pillay et al., 2015)[62]; Annals of	HbAlc level.	**Number of studies:** 132;<br>**Types of studies:**	Behavioral Programs.	√ BEHA (-)<br>√ DIET (-)<br>☐ DR	Trained individuals.	**Number of session:** Unclear;<br>**Total contact hours:**	**Strategies:** Unclear;<br>**Format:** Unclear;<br>**Theoretical approach:** Unclear.	Inpatient settings, post discharge,	− **HbA1c;**<br>↓ **BMI.**	The analysis was conducted

For peer review only

internal medicine		RCTs;<br>**Total sample:**<br>Unclear.		√ EXERCISE (-)<br>√ GC (-)<br>√ MED (-)<br>☐ PSY<br>☐ SMOKING<br>√ SELF (-)		range, 7 to 40.5 hours;<br>**Duration:** 4 or more weeks.		other		by using a Bayesian network model.
(Pal et al., 2014) [60];<br>Diabetes Care	Health status, cardiovascular risk factors, and QoL.	**Number of studies:**<br>20 papers describing 16 studies;<br>**Types of studies:**<br>RCTs;<br>**Total sample:** 3,578 participants.	Computer-based self-management interventions.	☐ BEHA<br>☐ DIET<br>☐ DR<br>☐ EXERCISE<br>☐ GC<br>☐ MED<br>☐ PSY<br>☐ SMOKING<br>√ SELF	Unclear, did not describe.	**Number of session:** 1-8 ;<br>**Total contact hours:** 10 mins - 6 hours;<br>**Duration:** 8 weeks – 12 months;	**Strategies:** Online/web-based; Phone contact.<br>**Format:** Individual; Group and Mixed.<br>**Theoretical approach:** TTM, social ecological theory, SCT, and self-determination theory.	Unclear, did not describe.	**− HRQoL,**<br>**↓ HbA1c,**<br>**− Death;**<br>↓ Cognitions, behaviors,<br>− Social support,<br>↓ Cardiovascular risk factors,<br>− Complications,<br>− Emotional outcomes,<br>− Hypoglycemia,<br>− Adverse effects,<br>− CE and economic data.	Meta-analysis using Review Manager software or narrative presentatio.
(Ricci-Cabello et al., 2014) [64];<br>BMC endocrine disorders	Knowledge, behaviors and clinical outcomes.	**Number of studies:**<br>Thirty-seven studies;<br>**Types of studies:**<br>Almost two thirds of the studies were RCTs, 27% studies were Quasi-experimental design.<br>**Total sample:**<br>Unclear.	DSM educational programs.	☐ BEHA<br>√ DIET(+++)<br>☐ DR<br>√ EXERCISE (+++)<br>√ GC(+++)<br>√ MED(++)<br>√ PSY(++)<br>☐ SMOKING<br>☐ SELF	Dietitian; Nurse; Psychologist; Physician; Research team or staff;	**Number of session:**<br>13.1;<br>**Total contact hours:**<br>0.25-180 hours;<br>**Duration:** 0.25-48 months.	**Strategies:** Face-to-face; Telecommunication; Both.<br>**Format:** One on one; Group and Mixed.<br>**Theoretical approach:** Unclear.	Post discharge, other	**− Diabetes knowledge;**<br>**− Self-management;**<br>**− Behaviors;**<br>**− Clinical outcomes;**<br>↓ Glycated hemoglobin;<br>− Cost-effectiveness analysis.	Meta-analyses and bivariate meta-regression were conducted with Stata, version 12.0.

For peer review only

(Saffari, Ghanizadeh, & Koenig, 2014) [65]; Primary Care Diabetes	Glycemic control.	**Number of studies:** 10; **Types of studies:** RCTs; **Total sample:** 960 patients.	An educational intervention using SMS.	√ BEHA (-) ☐ DIET ☐ DR ☐ EXERCISE √ GC (-) √ MED (-) ☐ PSY ☐ SMOKING ☐ SELF	Unclear, did not describe.	**Number of session:** Weekly; or 2 messages daily or unclear; **Total contact hours:** Unclear. **Duration:** 3 months – 1 year	**Strategies:** SMS: sending and receiving data. Receive data through text-messaging by patients only. Used a website along with SMS; **Format:** Unclear; **Theoretical approach:** Unclear.	Inpatient settings, post discharge, other	↑ **Glycemic control**.	Comprehensive Meta-analysis Software Version 2.0.
(Odnoletkova et al., 2014) [5[illegible]]; Journal of Diabetes & Metabolism	Cost-effectivenes s (CE).	**Number of studies:** Seventeen studies; **Types of studies:** RCTs; **Total sample:** Unclear.	Therapeutic education.	√ BEHA (-) ☐ DIET ☐ DR ☐ EXERCISE ☐ GC ☐ MED ☐ PSY ☐ SMOKING √ SELF (-)	General physician; nutritionists or unclear.	**Number of session:** ~ 16; **Total contact hours:** Unclear; **Duration:** Unclear.	**Strategies:** Face-to-face or unclear; **Format:** individual and group lessons; **Theoretical approach:** Unclear.	Inhospital or unclear.	− **CE.**	Incremental Cost-Effectivenes s Ratio.
(Attridge, Creamer, Ramsden, Cannings-John, & Hawthorne, 2014) [26]; The Cochrane Library	HbAlc level, Knowledge and clinical outcomes.	**Number of studies:** 33 trials; **Types of studies:** RCTs and quasi-RCTs; **Total sample:** 7453 participants.	'Culturally appropriate' health education.	√ BEHA (-) √ DIET (-) ☐ DR √ EXERCISE (-) √ GC (-) ☐ MED ☐ PSY √ SMOKING (-) ☐ SELF	CHWs; dieticians; exercise physiologists; lay workers; nurses; podiatrists; and psychologists;.	**Number of session:** one session to 24 months; **Total contact hours:** Unclear; **Duration:** The median duration of interventions was six months.	**Strategies:** **Format:** Group intervention method, one-to-one sessions and a mixture of the two methods. Or a purely interactive patient-centred method. **Theoretical approach:** empowerment theories; behaviour change theories, TTM of behaviour change and SCT.	Inpatient settings, post discharge, other	↓ **HbA1c;** − **HRQoL;** − **Adverse events;** − Mortality; − Complications; −Satisfaction; ↑ Empowerment; ↑ Self-efficacy; − Attitude; knowledge;	Meta-analyses used Review Manager software.

For peer review only

									−BP;<br>−BMI;<br>↓ Lipid levels;<br>−Health economics.	
(Vugt, Wit, Cleijne, & Snoek, 2013) [69]; Journal of Medical Internet Research	Health outcomes.	**Number of studies:** 13 studies;<br>**Types of studies:** RCTs;<br>**Total sample:** 3813 patients.	BCTs are being used in online self-management interventions.	√ BEHA (-)<br>☐ DIET<br>☐ DR<br>☐ EXERCISE<br>☐ GC<br>☐ MED<br>☐ PSY<br>☐ SMOKING<br>√ SELF (-)	Health care professional.	**Number of session:** 6 weekly sessions or unclear;<br>**Total contact hours:** Unclear;<br>**Duration:** Unclear.	**Strategies:** Online/web-based;<br>**Format:** Unclear;<br>**Theoretical approach:** self-efficacy theory, social support theory, TTM, SCT, social-ecological model, and cognitive behavioral therapy.	Post discharge	**− Health behavior change,**<br>**− Psychological well-being;**<br>− Clinical parameters.	Unclear.
(Gucciardi, Chan, Manuel, & Sidani, 2013) [46]; Patient Education and Counseling	HbAlc level,physical activity and diet outcomes.	**Number of studies:** Thirteen studies;<br>**Types of studies:** RCTs and comparative studies;<br>**Total sample:** Unclear.	DSME interventions.	☐ BEHA<br>√ DIET (+++);<br>☐ DR<br>√ EXERCISE (+++);<br>☐ GC<br>√ MED (+);<br>√ PSY (+)<br>☐ SMOKING<br>√ SELF (++).	Dietitians (n = 7/13); Multidisciplinary Team (n = 7/13); Nurse (n = 5/13); Community Peer Worker (n = 3/13).	**Number of session:** Low Intensity: <10 education sessions (n = 7); High Intensity: ≥ 10 education sessions (n = 6);<br>**Total contact hours:** Unclear;<br>**Duration:** < 6 months (n = 7/13); ⩾6months (n = 6/13).	**Strategies:** Face-to-face(n = 13/13); Written Literature: (e.g., handbook) (n = 4/13);Telephone(n = 4/13);Audio-Visual(n = 1/13);<br>**Format:** One-on-one: (n = 11/13);Group (n = 9/13);<br>**Theoretical approach:** SAT; Empowerment Behavior Change Model; modification theories; Pharmaceutical Care Model; Behavior Change Theory; PATHWAYS Program; Symptom-Focused Management Model; Motivational interviewing.	Inpatient settings, post discharge	**− HbA1c levels,**<br>− Anthropometrics,<br>− Physical activity;<br>− Diet outcomes.	Arecently described method.
(Pal et al., 2013) [61]; The Cochrane Library	Health status and HRQoL.	**Number of studies:** 16 studies;<br>**Types of studies:** RCTs;<br>**Total sample:** 3,578	Computer-based diabetes self-management intervention.	☐ BEHA<br>√ DIET (-)<br>☐ DR<br>√ EXERCISE (-)<br>√ GC (-)	Nurse or other healthcare professionals.	**Number of session:** Unclear;<br>**Total contact hours:** Unclear;<br>**Duration:** One session –	**Strategies:** Online/web-based; phone contact<br>**Format:** Unclear;<br>**Theoretical approach:** Unclear.	Inpatient settings, post discharge, other	**− HRQoL;**<br>**− Death from any cause;**<br>**↓ HbA1c;**	Formal meta-analyses and narrative

For peer review only

		participants.		√ MED (-)<br>√ PSY (-)<br>☐ SMOKING<br>☐ SELF		1[illegible] months.			− Cognitions;<br>− Behaviours;<br>−Social support;<br>−Biological markers;<br>− Complications.	synthesis.
(Nam, Janson, Stotts, Chesla, & Kroon, 2012)[55]; Journal of Cardiovascular Nursing	Glycemic control.	**Number of studies:** 12 RCTs;<br>**Types of studies:** RCTs;<br>**Total sample:** 1,495 participants.	Diabetes educational interventions (no drug intervention).	☐ BEHA<br>√ DIET (-)<br>☐ DR<br>√ EXERCISE (-)<br>√ GC (-)<br>√ MED (-)<br>√ PSY (-)<br>☐ SMOKING<br>√ SELF (-)	Nurses (36%), dieticians (36%), diabetes educators (5%), other professionals (9%) and nonprofessional staff (14%).	**Number of session:** 1 month or less; 1 to 3 months; and 12 months;<br>**Total contact hours:** most studies did not describe, or from 1 session to more than 30 hours;<br>**Duration:** from 1 session to 12 months, frequency: 1 session to 25 weekly or biweekly education.	**Strategies:** Teaching or counseling; home-based support; and visual aids.<br>**Format:** group education or a combination of group education and individual counseling; or only individual counseling;<br>**Theoretical approach:** Unclear.	Inpatient settings, post discharge, other	↓ **HbA1c level.**	Meta-analysis.
(Steinsbekk, Rygg, Lisulo, Rise, & Fretheim, 2012)[66]; BMC Health Services Research	Clinical, lifestyle and psychosocial outcomes.	**Number of studies:** 21 studies (26 publications).<br>**Types of studies:** RCTs;<br>**Total sample:** 2,833 participants.	Group-based education.	Did not describe the content of the intervention.	Community workers; dietician; lay health advisors nurse and nutritionist.	**Number of session and Total contact hours:** 30 hours over 2.5 months, 52 hours over one year,and 36 hours or 96 hours over 6 months.<br>**Duration:** Six months to two years.	**Strategies:** Face to face;<br>**Format:** 5 to 8 participants group to 40 patients group.<br>**Theoretical approach:** empowerment model and the discovery learning theory, the SCT and the social ecological theory, the self-efficacy and self-management theories and operant reinforcement theory.	Inpatient settings, post discharge, other	↓ **HbA1c,**<br>↑ **Lifestyle outcomes,**<br>↑ **Diabetes knowledge,**<br>↑ Self-management skills,<br>↑ Psychosocial outcomes,<br>↓ Mortality rate,<br>↓ BMI,<br>↓ Blood pressure;<br>↓ Lipid profile.	Meta-analysis using Review manager v5.
(Amaeshi,	Increasing	**Number of studies:**	Foot health	Food care.	Podiatrist,	**Number of session:**	**Strategies:** Face to face;	Unclear, did	↓ **LEA;**	Narrative

2012) [25]; Podiatry Now	good foot health practices that will ultimately reduce LEA.	8 studies;<br>**Types of studies:** RCT or clinical controlled trial (CCT);<br>**Total sample:** Unclear.	education:		psychotogist or unclear.	Unclear;<br>**Total contact hours:** between 15min and 14 hours;<br>**Duration:** 3-30 months.	**Format:** In three of the studies, educational interventions were delivered to the participants in groups, while the other five provided individualized (one-to-one) foot-care education to the participants;<br>**Theoretical approach:** Unclear.	not describe.	↑ Self-care.	synthesis.
Kai Lun Gan, Brammer, & Creedy, 2011) [50]; JBI Library of Systematic Reviews	Oral hypoglycaemic adherence.	**Number of studies:** Seven studies;<br>**Types of studies:** RCTs;<br>**Total sample:** Unclear.	Educational interventions.	√ BEHA (-)<br>√ DIET (-)<br>☐ DR<br>√ EXERCISE (-)<br>√ GC (-)<br>√ MED (-)<br>√ PSY (-)<br>☐ SMOKING<br>√ SELF (-)	Nurses; pharmacists; other skilled healthcare professionals.	**Number of session:** 1-12 or unclear;<br>**Total contact hours:** 2.5 hours or unclear;<br>**Duration:** 4-12 months.	**Strategies:** Face to face;<br>**Format:** Group and individual;<br>**Theoretical approach:** Unclear.	Inpatient settings, post discharge, other	↓ **HbA1c,**<br>**− Medication adherence;**<br>↓ Blood glucose;<br>− Tablet count;<br>− Medication containers;<br>− Diabetes complications;<br>− Health service utilization.	Narrative summary form.
Burke et al., 2011) [31]; JBI Database of Systematic Reviews and Implementation Reports	HbAlc level,BP.	**Number of studies:** 11 RCTs and 4 quasi-experimental trials;<br>**Types of studies:** RCTs and quasi-experimental trials;<br>**Total sample:** 2,240 patients.	Group medical visits.	√ BEHA (-)<br>√ DIET (-)<br>☐ DR<br>☐ EXERCISE<br>√ GC (-)<br>√ MED (-)<br>☐ PSY<br>☐ SMOKING<br>√ SELF (-)	Dndocrinologists; DM nurse; family physician; nutritionist and rehab therapist.	**Number of session:** 1-4 or unclear;<br>**Total contact hours:** 2-4 hours or unclear;<br>**Duration:** One session to 2 years.	**Strategies:** Face to face;<br>**Format:** Group and individual;<br>**Theoretical approach:** Unclear.	Inpatient settings, post discharge, other	↓ **HbA1c;**<br>**− Systolic and diastolic BP;**<br>− LDL measurements.	Meta-analysis.
Ramadas, Quek, Chan, & Oldenburg, 2011) [63];	HbAlc level.	**Number of studies:** 13 different studies;<br>**Types of studies:** RCTs and quasi-experimental studies;	Web-based behavioural interventions.	√ BEHA (-)<br>√ DIET (-)<br>☐ DR<br>☐ EXERCISE	Dietician; endocrinologist; physicians; researchers or research staff	**Number of session:** Unclear;<br>**Total contact hours:** Unclear;<br>**Duration:** ranged	**Strategies:** E-mail and SMS technologies that were commonly used together with the websites to reinforce the intervention, and Website, print material.	Inpatient settings, post discharge, other	**− Self-monitoring blood sugar,**<br>− Weight-loss,<br>− Dietary behaviour,	Not statistically combined and re-analyzed;

For peer review only

International Journal of Medical Informatics		**Total sample:**<br>Unclear.		√ GC (-)<br>√ MED (-)<br>☐ PSY<br>☐ SMOKING<br>√ SELF (-)	members and study nurse.	between 12 and 52 weeks, with an average of 27.2 ± 18.3 weeks.	**Format:** Unclear;<br>**Theoretical approach:** Wagner's Chronic Care Model; Self-efficacy theory/Social support theory; TTM; HBM; SCT.		− Physical activity.	
(Minet, Moller, Vach, Wagner, & Henriksen, 2010) [54];<br>Patient Education and Counseling	Glycaemic control.	**Number of studies:** Forty-seven studies;<br>**Types of studies:** RCTs;<br>**Total sample:**<br>Unclear.	Self-care management interventions.	√ BEHA (-)<br>☐ DIET<br>☐ DR<br>☐ EXERCISE<br>☐ GC<br>☐ MED<br>☐ PSY<br>☐ SMOKING<br>√ SELF (-)	Case nurse manager; group facilitator; nurse educator; multidisciplinary team; physiologist; physician; peer counsellor; researcher; and pharmacist.	**Number of session:** 3-26;<br>**Total contact hours:** Unclear;<br>**Duration:** 4 weeks to 4 years.	**Strategies:** Face to face; home visit; phone calls;<br>**Format:** Group and individual;<br>**Theoretical approach:** Unclear.	Inpatient settings, post discharge, other	↓ **HbA1c.**	Meta-analyses and meta-regression used Stata's meta command.
(Hawthorne, Robles, Cannings-John, & Edwards, 2010) [47];<br>Diabetic Medicine	Effects of culturally appropriate health education	**Number of studies:** 10 trials;<br>**Types of studies:** RCTs;<br>**Total sample:** 1603 patients.	Culturally appropriate health education	☐ BEHA<br>√ DIET (-)<br>☐ DR<br>√ EXERCISE (-)<br>☐ GC<br>☐ MED<br>☐ PSY<br>☐ SMOKING<br>√ SELF (-)	Exercise physiologists; dieticians; diabetes nurses; link workers, and podiatrists.	**Number of session:** Unclear;<br>**Total contact hours:** Unclear;<br>**Duration:** one session to 12 months;	**Strategies:** Face to face; visual aids, leaflets and teaching materials;<br>**Format:** group approach, one-to-one interviews and a mixed approach;<br>**Theoretical approach:** SAT, Empowerment Behaviour Change Model, SCT, Management model and the Theory of Planned Behaviour.	Inpatient settings, post discharge, other	**−QoL;**<br>↓ **HbA1c;**<br>**− BP;**<br>↑ Knowledge;<br>− BMI;<br>↓ Lipid levels,<br>− Diabetic complications,<br>− Mortality rates, hospital admissions, hypoglycaemia.	Meta - analysis using the Review Manager and narrative review.
(Pan & Sidani, 2009) [49]; Canadian Journal of	Knowledge, self-management	**Number of studies:**<br>**50 studies;**<br>**Types of studies:** RCTs;	DSME intervention:	√ BEHA (-)<br>☐ DIET<br>☐ DR<br>☐ EXERCISE	Unclear, did not describe.	**Number of session:** 10 (range 1 to 28);<br>**Total contact hours:** 17 contact hours (range 1 to	**Strategies:** Online/web-based (4%); video (2%); face-to-face (60%); phone contact (4%); Mixed (30%).<br>**Format:** One-on-one (32%); Group	Inpatient settings, post discharge, other	↑ **Diabetes knowledge,**<br>↑ **Self-management behaviours;**	Comprehensive Meta-Analysis (version

For peer review only

Diabetes	behaviours and metabolic control.	**Total sample:** **Unclear.**		☐ GC<br>☐ MED<br>√ PSY (-)<br>☐ SMOKING<br>√ SELF (-)		52); ≤ 10 (46%); 11-20 (21%); > 20 (33%);<br>**Duration:** 22 weeks (range 1 to 48); ≤ 8 weeks (26%); 9–24 weeks (37%); > 24 weeks (37%).	(40%); Mixed (28%).<br>**Theoretical approach:** Unclear.		↓ **HbA1c,**	2.0).
(Duke, Colagiuri, & Colagiuri, 2009) [39]; The Cochrane Library	Metabolic control, diabetes knowledge and psychosocial outcomes.	**Number of studies:** Nine studies;<br>**Types of studies:** RCTs;<br>**Total sample:** 1,359 participants.	Individual patient education.	√ BEHA (-)<br>☐ DIET<br>☐ DR<br>√ EXERCISE (-)<br>√ GC (-)<br>☐ MED<br>√ PSY (-)<br>☐ SMOKING<br>☐ SELF	Diabetes educators and dieticians.	**Number of session:** 1-6;<br>**Total contact hours:** 20 min – 7 hours;<br>**Duration:** 4 weeks – 1 year.	**Strategies:** Face to face; telephone;<br>**Format:** Individual;<br>**Theoretical approach:** Unclear.	Inpatient settings	− **HbA1c;**<br>− **Diabetes complications;**<br>− Health service utilisation and health care costs;<br>− Psychosocial outcomes;<br>− Diabetes knowledge; patient self-care behaviours;<br>− Physical measures; metabolic.	Meta-analysis.
(Alam, Sturt, Lall, & Winkley, 2009) [23]; Patient Education and Counseling	Glycaemic control and psychological status.	**Number of studies:** 35 trials;<br>**Types of studies:** RCTs;<br>**Total sample:** 1431 patients.	Psycho-educational Interventions.	√ BEHA (-)<br>☐ DIET<br>☐ DR<br>☐ EXERCISE<br>☐ GC<br>☐ MED<br>√ PSY (-)<br>☐ SMOKING<br>☐ SELF	Generalists; psychological specialists; or did not report the specialist	**Number of session:** 1-16;<br>**Total contact hours:** 20 min – 28 hours;<br>**Duration:** about 13.7 (±11.06) weeks.	**Strategies:** Face to face; telephone calls;<br>**Format:** group format; a single format and used a combination;<br>**Theoretical approach:** TTM; motivational interviewing;	Inpatient settings, other	↓ **HbAlc;**<br>↓ **Psychological distress.**	Meta-analysis.
(Khunti, Camosso-	Knowledge and	**Number of studies:** nine studies;	Any educational	☐ BEHA<br>√ DIET<br>☐	Unclear, did not describe.	**Number of session:** Unclear;	**Strategies:** Face to face;<br>**Format:** Group and individual;	Unclear, did not describe.	− **Knowledge;**<br>− **Psychological and**	Unclear.

For peer review only

Ste[illegible]novic, Carey, Davies, & Stone, 2008)[51]; Diabetic Medicine	biomedical outcomes.	**Types of studies:** RCTs and RCT was followed by a before-and-after study;<br>**Total sample:** 1004 patients.	intervention.	DR<br>☐ EXERCISE<br>☐ GC<br>☐ MED<br>☐ PSY<br>☐ SMOKING<br>☐ SELF		**Total contact hours:** Unclear;<br>**Duration:** 3- 12 months.	**Theoretical approach:** Unclear.		**biomedical outcome measures.**	
(Loveman, Frampton, & Clegg, 2008)[53]; Health Technology Assessment	Clinical effectivenes s.	**Number of studies:** Twenty-one published trials;<br>**Types of studies:** RCTs and CCTs;<br>**Total sample:** Unclear.	Educational interventions.	√ BEHA (++)<br>√ DIET (+++)<br>☐ DR<br>√ EXERCISE (+++)<br>√ GC (+++)<br>☐ MED<br>☐ PSY<br>☐ SMOKING<br>√ SELF (+++)	Community workers; diabetes research technician; diabetes nurse, dieticians; educationalist; medical students; nurses; pharmacists; physician, or physician assistant.	**Number of session:** Two to four intensive education of 1.5 to 2 hours followed up with additional education at, 3 and 6 months;<br>**Total contact hours and Duration:** about 150 mins over a 6-month or 61 to 52 hours over one year.	**Strategies:** Face to face;<br>**Format:** Group and individual;<br>**Theoretical approach:** cognitive-behavioural strategies; pedagogical principle.	Inpatient settings, post discharge, other	**− Diabetic control outcomes**;<br>− Diabetic end-points;<br>− QoL and cognitive measures.	Narrative review
(Wens et al., 2008)[70]; Diabetes Research and Clinical Practice	Improving adherence to medical treatment recommend ations.	**Number of studies:** 8 studies;<br>**Types of studies:** RCTs and controlling before and after studies.<br>**Total sample:** 772 patients.	Interventions aimed at improving adherence to medical treatment.	√ BEHA (-)<br>√ DIET (-)<br>☐ DR<br>√ EXERCISE (-)<br>√ GC (-)<br>√ MED (-)<br>☐ PSY<br>☐ SMOKING<br>√ SELF (-)	Diabetes educator; nurse or did not describe.	**Number of session:** Unclear;<br>**Total contact hours:** Unclear;<br>**Duration:** ～9 months or unclear.	**Strategies:** Face-to-face; telephone;<br>**Format:** face-to-face; group based and telemedicine;<br>**Theoretical approach:** Unclear.	Inpatient settings, post discharge, other	**− Adherence;**<br>− HbA1c;<br>− Blood glucose.	Cochrane Review Manager software.

For peer review only

(Hawthorne, Robles, Cannings-John, & Edwards, 2008) [46]; The Cochrane Library	HbAlc level, Knowledge and clinical outcomes.	**Number of studies:** A total of 11 trials; **Types of studies:** RCTs; **Total sample:** 1,603 patients.	Culturally appropriate (or adapted) health education.	√ BEHA (-)<br>√ DIET (-)<br>☐ DR<br>√ EXERCISE (-)<br>√ GC (-)<br>☐ MED<br>☐ PSY<br>√ SMOKING (-)<br>☐ SELF	Dieticians, diabetes nurses, exercise physiologists; link workers; podiatrists; psychologist and and non-professional link worker.	**Number of session:** Unclear; **Total contact hours:** Unclear; **Duration:** one session to 12 months.	**Strategies:** Face-to-face; booklet; **Format:** Group intervention method; one to one interviews; mixture of the two methods; purely interactive patient-centred method; semi-structured didactic format and combination of the 2 approaches. **Theoretical approach:** SAT; Empowerment Behaviour Change Model; Behaviour Change Theory; SCT, Management Model and the Theory of Planned Behaviour.	Inpatient settings, post discharge, other	**↓ HbA1c**<br>**↑ Knowledge scores**<br>− Other outcome measures	Narrative presentation and meta-analysis.
(Nield et al., 2007) [56]; The Cochrane Library	Metablic control.	**Number of studies:** 36 articles (18 trials); **Types of studies:** RCTs; **Total sample:** 1,467 participants.	Dietary advice.	☐ BEHA<br>√ DIET<br>☐ DR<br>☐ EXERCISE<br>☐ GC<br>☐ MED<br>☐ PSY<br>☐ SMOKING<br>☐ SELF	exercise physiologist; dietitian; group facilitator; nutritionist; nurse educator; and physician.	**Number of session:** 1-12; **Total contact hours:** 20 min-22 hours; **Duration:** 11 weeks – 6months or unclear.	**Strategies:** Face-to-face; **Format:** Group and individual; **Theoretical approach:** Unclear.	Inpatient settings, post discharge, other	**− Weight;**<br>**− Diabetic complications;**<br>**− HbA1c;**<br>− QoL;<br>− Medication use;<br>− Cardiovascular disease risk;	Meta-analysis.
(Zabaleta & Forbes, 2007) [71]; British Journal of Community Nursing	Clinical effectivenes s.	**Number of studies:** 21 studies; **Types of studies:** Controlled trials; **Total sample:** Unclear.	Structured group diabetes education.	√ BEHA (-)<br>√ DIET (-)<br>☐ DR<br>√ EXERCISE (-)<br>√ GC (-)<br>☐ MED<br>√ PSY (-)<br>☐ SMOKING<br>☐ SELF	Diabetes nurse educator; physician's assistant; and physicians;	**Number of session:** 4-6 or unclear; **Total contact hours:** 6-12 hours or unclear; **Duration:** 1-6 months or unclear.	**Strategies:** Face to face; **Format:** Group; **Theoretical approach:** Unclear.	Post discharge.	**−HbA1c.**	A tabulative synthesis.

For peer review only

(Deakin, McShane, Cade, & Williams, 2005) [36]; The Cochrane Library	Clinical, lifestyle and psychosocial outcomes.	**Number of studies:** 14 publications, reporting 11 studies;<br>**Types of studies:** RCTs, and CCTs;<br>**Total sample:** 1,532 participants.	Group-based educational programmes.	Did not describe the content of the intervention.	Health professionals, lay health advisors.	**Number of session:** Unclear;<br>**Total contact hours:** from 6 to 52 hours;<br>**Duration:** 3 hours per year for 2 years and 3 or 4 hours per year for 4 years.	**Strategies:** Unclear;<br>**Format:** Group;<br>**Theoretical approach:** the Diabetes Treatment and Teaching Programme (DTTP); empowerment model; adult learning model, public health model, HBM and TTM.	Inpatient settings, post discharge	↓ **Metabolic control;**<br>↑ **Diabetes knowledge;** ↑ QoL; ↑ Empowerment/self-efficacy.	Summarized statistically
(Vermeire et al., 2005) [68]; The Cochrane Library	Improving adherence to treatment recommendations.	**Number of studies:** 21 articles;<br>**Types of studies:** RCTs; cross-over study; controlled trial; controlled before and after studies;<br>**Total sample:** 4,135 patients.	Interventions that were aimed at improving the adherence to treatment recommendations.	☐ BEHA<br>☐ DIET<br>☐ DR<br>☐ EXERCISE<br>√ GC (-)<br>√ MED (-)<br>☐ PSY<br>☐ SMOKING<br>☐ SELF	Nurse, pharmacist and other healthcare professionals.	**Number of session:** Unclear;<br>**Total contact hours:** Unclear;<br>**Duration:** Unclear.	**Strategies:** Face to face; telephone; home visit; video; mailed educational materials;<br>**Format:** Unclear.<br>**Theoretical approach:** Unclear.	Inpatient settings, post discharge	**Direct indicators,** such as<br>↓ Blood glucose level;<br>− Indirect indicators, such as pill counts;<br>−Health outcomes.	A descriptive review and sub-group meta-analysis.
(Gary, Genkinger, Guallar, Peyrot, & Brancati, 2003) [42]; Diabetes Educator	Body weight and glycaemic control.	**Number of studies:** Sixty-three RCTs;<br>**Types of studies:** RCTs;<br>**Total sample:** 2,720 patients.	Educational and behavioural component interventions.	☐ BEHA<br>√ DIET (-)<br>☐ DR<br>√ EXERCISE (-)<br>√ GC (-)<br>√ MED (-)<br>☐ PSY<br>☐ SMOKING<br>☐ SELF	Nurse (39%); dietitian (26%); physician (17%); other or not specified (23%); other professional (13%); psychologist (9%); Exercise psychologist (9%); and health educator (4%).	**Number of session:** Unclear;<br>**Total contact hours:** Unclear.<br>**Duration:** 1 month to 19.2 months.	**Strategies:** Unclear;<br>**Format:** Unclear;<br>**Theoretical approach:** SAT, contracting model and patient empowerment.	Inpatient settings, post discharge	− **Glycaemic control;**<br>− **Weight.**	Sufficient data were combined using meta-analysis.
(Norris, Lau, Smith,	Total GHb.	**Number of studies:** 31 studies	Self-management	√ BEHA (-)<br>√ DIET (-)	Dietitian; lay health care	**Number of session:** 6 (1–36);	**Strategies:** Online/web-based; video ; face-to-face; phone contact;	Inpatient settings, post	↓ **Total GHb,**	Meta-analysis and

For peer review only

Schmid, & Engelgau, 2002)[58]; Diabetes Care		**Types of studies:** RCTs.<br>**Total sample:** 4,263 patients.	education.	☐DR<br>☐EXERCISE<br>☐GC<br>☐MED<br>☐PSY<br>☐SMOKING<br>√ SELF (-)	worker; nurse; physician with team; self (e.g. computer assisted instruction); and team (nurse, dietitian, etc.).	**Total contact hours:** 9.2 (1–28) hours;<br>**Duration:** 6 (1.0–27) months.	**Format: Group;** individual and mixed;<br>**Theoretical approach:** Unclear	discharge, other		meta-regression.
(Norris, Engelgau, & Narayan, 2001)[57]; Diabetes Care	Clinical outcomes,Knowledge, Metablic control.	**Number of studies:** 72 studies (84 papers);<br>**Types of studies:** RCTs;<br>**Total sample:** Unclear.	Self-management training interventions.	√ BEHA (-)<br>√ DIET (-)<br>☐DR<br>☐EXERCISE<br>☐GC<br>☐MED<br>☐PSY<br>☐SMOKING<br>√ SELF (-)	CHWs; nurse; or other healthcare professionals.	**Number of session:** 1-16;<br>**Total contact hours:** ~22 hours;<br>**Duration:** ~26 months.	**Strategies:** Online/web-based; video (2%); face-to-face; phone contact;<br>**Format:** Group; individual and mixed;<br>**Theoretical approach:** SAT; Fishbein and Ajzen HBM.	Inpatient settings, post discharge, other	↑ **Knowledge;**<br>↑ **Lifestyle behaviors;**<br>**−Psychological and QoL outcomes;**<br>↑ **Glycemic control**;<br>− Cardiovascular disease risk factors.	Outcomes are summarized in a qualitative fashion.

NOTE:

SMOKING= Smoking cessation; CVR= Cardiovascular risk factors; PSY =Psychosocial issues (depression, anxiety); DIET =Diet; EXERCISE= Exercise; MED= Medication; BEHA = Behavioral charge (including lifestyle modification); SELF = Self-management (including problems solving); GC= glycemic control; DR =Diabetes risks; CHD= Coronary heart disease; CAD = Coronary Artery Disease; CHW=Community Health Worker; HbA1c = Glycatedhemoglobin; BP =Blood Pressure; LDL = Low-density Lipoprotein Cholesterol; SMS = Short Message Service; BCTs = Behavioral Change Techniques; LEA = Lower Extremity Amputation; PRIDE = Problem Identification, Researching one's routine, Identifying a management goal, Developing a plan to reach it, Expressing one's reactions and Establishing rewards for making progress; ASE = Attitude-social influence-efficacy; CVRF= Cardiovascular Risk Factors; SMS = Short Message System; PA = Physical Activity; EDU = Patient Education; GP= General Practice; RCTs =Randomized controlled trials; CCTS = controlled clinical trials; HRQoL = Health Related Quality of Life; QoL = Quality of Life; MI = Myocardial Infarction; CAD = Coronary Artery Disease; CABG= Coronary Artery Bypass Graft Surgery; T2DM = Type 2 Diabetes Mellitus; BMI = Body Mass Index; HbA1c = Glycatedhemoglobin; BP =Blood Pressure; SBP = Systolic Blood Pressure, DBP = Diastolic Blood Pressure, HDL-c= High-density Lipoprotein Cholesterol; LDL-c = Low-density Lipoprotein Cholesterol; DSME = Diabetes Self-management Education; DSM = Diabetes Self-management; TTM= Transtheoretical Model; SCT=Social Cognitive Theory; HBM =Health Belief Model; SAT=Social Action Theory;

In the educational content: “+”: minor focus; “++”:moderate focus; “+++” major focus; “-“ =unclear what the intensity of the education was for any topic;

In the outcomes: arrow up (“ ↑ ”) for improvement, arrow down (“ ↓ ”) for reduction; a dash (“−”) for no change or inconclusive evidence.

For peer review only

Table 4. Summary of evidence from quantitative research syntheses

Intervention	Number of systematic reviews/meta-analysis, Total participants	First author, Year	Primary Results/findings		Rating the evidence of effectiveness
**Acute Coronary Syndrome Patients**					
General health education	Six/161,997patients (Goulding et al., 2010 did not given the total sample size)	Ghisi et al., 2014 [43]	Knowledge	91% studies (+)	Some evidence
			Behavior	77%/84%/65% studies (+)	
			Psychosocial indicators	43% studies (+)	
		Brown et al., , 2013 [30]	Mortality		
			MI		
			Revascularizations		
			Hospitalizations		
			HRQoL		
			Withdrawals/drop outs		
			Healthcare utilization and costs		
		Brown et al., 2011 [72]	Total mortality		
			MI		
			CABG		
			Hospitalisations		
			HRQoL	63.6% studies (+)	
			Healthcare costs	40% studies (+)	
			Withdrawal/drop out		
		Goulding et al., 2010 [44]	Beliefs	30.08% studies (+)	
			Secondary outcomes		
			Smoking		
			Cholesterol level		

For peer review only - http://bmjopen.bmj.com/site/about/guidelines.xhtml

		Fernandez et al., 2007 [41]	Multiple risk factor modification		
		Kotb, Hsieh, & Wells, 2014 [52]	All-cause hospitalization		
			All-cause mortality		
			Smoking cessation		
			Depression		
			Systolic blood pressure		
			Low-density lipoprotein		
			Anxiety		
Psychoeducational interventions	Six/ 37,883 patients	Barth et al., 2015 [73]	Abstinence by self-report or validated		Sufficient evidence
		Dickens et al., 2013 [38]	Depression		
		Aldcroft et al., 2011 [24]	Smoking cessation		
			Physical activity		
		Huttunen-Lenz et al.,2010 [49]	Prevalent smoking cessation		
			Continuous smoking cessation		
			Total mortality		
		Barth et al., 2008 [29]	Abstinence by self-report or validated		
			Smoking status		
		Barth et al., 2006 [28]	Abstinence		
			Smoking status		
Secondary prevention educational interventions	Three/25,154 patients	Devi et al., 2015 [37]	Mortality		
			Revascularisation		
			Total Cholesterol		
			HDL cholesterol		
			Triglycerides		
			HRQOL		
			All-cause mortality		

For peer review only

(including Internet based secondary prevention)		Auer et al., 2008 [27]	Readmission rates		Some evidence
			Reinfarction rates		
			Smoking cessation rates		
		Clark et al., 2005 [34]	Mortality		
			MI		
			Quality of life	Most of the included studies (+)	
**Type 2 Diabetes Mellitus Patients**					
General health education	Five/2,319 patients (Choi et al., 2016; Loveman et al., 2008; Zabaleta et al., 2007 did not given the total sample size)	Choi et al., 2016 [33]	HbA1c		Some evidence
		Saffari et al., 2014 [65]	Glycemic control		
		Duke et al., 2009 [39]	HbA1c		
			BP		
			Knowledge, psychosocial outcomes and smoking habits	No Data	
			Diabetes complications or health service utilization and cost analysis	No Data	
		Loveman et al., 2008 [53]	Diabetic control outcomes	46.15% studies (+)	
			Weight	66.67% studies (+)	
			Cholesterol or triglycerides	40.00% studies (+)	
		Zabaleta et al., 2007 [71]	HbA1c	4.8% studies (+)	
		Creamer et al., 2016 [35]	HbA1c		
			HRQoL		
			AEs	No AEs	
		Ricci-Cabello et al., 2014 [64]	HbA1c		
			Diabetes knowledge	73.3% studies (+)	
			Behaviors	75% studies (+)	
			Clinical outcomes	Fasting blood glucose, HbA1c	

For peer review only

				and BP improved in 71%, 59%, and 57% of the studies.	
Culturally appropriate health education	Eight/ 20,622 patients (Ricci-Cabello et al., 2014 and Gucciardi et al., 2013 did not given the total sample size)	Attridge et al., 2014 [26]	HbA1c		Some evidence
			Knowledge scores		
			Clinical outcomes		
			Other outcome measures	Showed neutral effects.	
		Gucciardi et al., 2013 [45]	HbA1c levels	Three of ten studies (+)	
			Anthropometrics	Three of eleven studies (+)	
			Physical activity	One of five studies (+)	
			Diet outcomes	Two of six studies (+)	
		Nam et al., 2012 [55]	HbA1c level		
		Hawthorne et al., 2010 [47]	HbA1c		
			Knowledge scores		
		Khunti et al., 2008 [51]	Knowledge levels	Only one study reporting a significant improvement	
			Biomedical outcomes	Only one study reporting a significant improvement.	
		Hawthorne et al., 2008 [46]	HbA1c		
			Knowledge scores		
			Other outcome measures		
		Huang et al., 2016 [48]	HbA1c		
			BMI		
			LDL-c and HDL-c		
		Chen et al., 2015 [32]	HbA1c		
			BMI		
			SBP		
			DBP		
			HDL-c		

For peer review only - http://bmjopen.bmj.com/site/about/guidelines.xhtml

			HbA1c level		
Lifestyle interventions + behavioral program	Six/10,440 patients (Huang et al., 2016; Pillay et al., 2015 and Ramadas et al., 2011 did not given the total sample size)	Terranova et al., 2015 [67]	Weight		Some evidence
		Pillay et al., 2015 [62]	HbA1c levels		
			BMI		
		Ramadas et al., 2011 [63]	HbA1c	46.2% studies (+)	
		Gary et al., 2003 [42]	Fast blood sugar		
			Glychemoglobin		
			HbA1		
			HbA1c		
			Weight		
Self-management educational interventions	Nine/19,597 patients (Minet et al., 2010;Fan et al., 2009 and Norris et al., 2001 did not given the total sample size)	Pal et al., 2014 [60]	Cardiovascular Risk Factors		
			Cognitive Outcomes		
			Behavioral Outcomes	Only one study reporting a significant improvement.	
			AEs	No AEs	
		Vugt et al., 2013 [69]	Health behaviors	Seven of 13 studies (+)	
			Clinical outcomes measures	Nine studies (+)	
			Psychological outcomes	Nine studies (+)	
		Pal et al., 2013 [61]	HbA1c		
			Depression		
			Quality of life		
			Weight		
		Steinsbekk, et al., 2012 [66]	HbA1c		
			Main lifestyle outcomes		
			Main psychosocial outcomes		
		Minet et al., 2010 [54]	Glycaemic control		
			Diabetes knowledge		
			Overall self-management		

For peer review only

			behaviours		
		Fan et al., 2009 [40]	Overall metabolic outcomes		Sufficient evidence
			Overall weighted mean effect sizes		
		Deakin et al., 2005 [36]	Metabolic control (HbA1c)		
			Fasting blood glucose levels		
			Weight		
			Diabetes knowledge		
			SBP		
			Diabetes medication		
		Norris et al.,, 2002 [58]	Total GHb		
		Norris et al., 2001 [57]	Knowledge		
			Self-monitoring of blood glucose		
			Self-reported dietary habits		
			Glycemic control		
Therapeutic education	One/Total sample: Unclear.	Odnoletkova et al., 2014 [59]	Cost-effectiveness	Overall high in studies on prediabetes and varied in studies on T2DM.	Insufficient evidence
Foot health education	One/ Total sample: Unclear.	Amaeshi, 2012 [25]	Diabetes complications		Some evidence
			Incidence of LEA		
Group medical visit	One/2,240 patients.	Burke et al., 2011 [31]	HbA1c		Some evidence
			BP and DBP		
			SBP		
			Cholesterol - LDL		
Psychoeducational intervention.	One/1.431 patients.	Alam et al., 2009 [23]	HbA1c		Some evidence
			Psychological status		
Interventions aimed at improving adherence to	Three/ 4,907 patients (Kai Lun Gan et al., 2011 did not	Kai Lun Gan et al., 2011 [50]	Oral hypoglycaemic adherence	Five of seven studies (+)	

For peer review only - http://bmjopen.bmj.com/site/about/guidelines.xhtml

medical treatment recommendations	given the total sample size)	Wens et al., 2008 [70]	Adherence	General conclusions could not be drawn.	Some evidence
		Vermeire et al., 2005 [68]	HbA1c		
Dietary advice	One/1,467 patients	Nield et al., 2007 [56]	Glycaemic control (addition of exercise to dietary advice)		Insufficient evidence to determine
			Weight	Limited Data	
			Diabetic micro- and macrovascular diseases	Limited Data	

NOTE:

RCTs =Randomized controlled trials; HRQoL = Health Related Quality of Life; MI = Myocardial Infarction; CABG= Coronary Artery Bypass Graft Surgery; T2DM = Type 2 Diabetes Mellitus; BMI = Body Mass Index; HbA1c = Glycatedhemoglobin; BP = Blood Pressure; SBP = Systolic Blood Pressure, DBP = Diastolic Blood Pressure, HDL-c= High-density Lipoprotein Cholesterol; LDL-c = Low-density Lipoprotein Cholesterol; LEA = Lower Extremity Amputation; Adverse Events = AEs.

（+）：Intervention Group is Significantly Better than Control Group; For example: “91% studies (+)” means 91% studies reported a significant better compared with control group;

For peer review only

Appendix 1. Searching Strategies

Items	Searching Strategy	Results
	**PubMed**	
#1	(((((((((Acute Coronary Syndrome[MeSH Terms]) OR Myocardial Infarction[MeSH Terms]) OR Angina, Unstable[MeSH Terms]) OR Coronary Artery Disease[MeSH Terms]) OR Coronary Artery Bypass[MeSH Terms]) OR Angina Pectoris[MeSH Terms]) OR "Myocardial Infarct*"[Title/Abstract]) OR "Unstable Angina*"[Title/Abstract]) OR "coronary heart diseas*"[Title/Abstract]) OR "Coronary Artery Disease*"[Title/Abstract]) OR "Unstable Angina Pectori*"[Title/Abstract] Filters: Publication date from 2000/01/01 to 2016/04/31; English	145310
#2	(((((Diabetes Mellitus, Type 2[MeSH Terms]) OR "Type 2 Diabetes Mellitus"[Title/Abstract]) OR "type 2 diabete*"[Title/Abstract]) OR "type ii diabet*"[Title/Abstract]) OR T2DM[Title/Abstract]) OR Adult-Onset Diabetes Mellitus[Title/Abstract] Filters: Publication date from 2000/01/01 to 2016/04/31; English	80218
#3	#1 OR #2	222103
#4	(((((((Health Education[MeSH Terms]) OR Patient Education as Topic[MeSH Terms]) OR Counseling[MeSH Terms]) OR Social Support[MeSH Terms]) OR "Community Health Education*"[Title/Abstract]) OR "Educational Activit*"[Title/Abstract]) OR "Education* intervention*"[Title/Abstract]) OR "Education* program*"[Title/Abstract] Filters: Publication date from 2000/01/01 to 2016/04/31; English	122323
#5	(((Meta-Analysis [Publication Type]) OR Meta-Analysis as Topic[MeSH Terms]) OR "Systematic Revie*"[Title/Abstract]) OR "Meta-analys*"[Title/Abstract] Filters: Publication date from 2000/01/01 to 2016/04/31; English	165265
#6	#3 AND #4 AND #5 Filters: Publication date from 2000/01/01 to 2016/04/31; English	**54**
	**Cochrane Library**	
#1	MeSH descriptor: [Acute Coronary Syndrome] explode all trees	1070
#2	MeSH descriptor: [Myocardial Infarction] explode all trees	9450
#3	MeSH descriptor: [Angina, Unstable] explode all trees	1015
#4	MeSH descriptor: [Coronary Artery Disease] explode all trees	3469
#5	MeSH descriptor: [Coronary Artery Bypass] explode all trees	5321
#6	MeSH descriptor: [Angina Pectoris] explode all trees	4240

#7	"acute coronary syndrome":ti,ab,kw or Unstable Angina*:ti,ab,kw or Myocardial Infarct*:kw or coronary heart diseas*:ti,ab,kw or Coronary Artery Disease*:ti,ab,kw (Word variations have been searched)	29379
#8	#1 or #2 or #3 or #4 or #5 or #6 or #7	34062
#9	MeSH descriptor: [Diabetes Mellitus, Type 2] explode all trees	10474
#10	Type 2 Diabetes Mellitus:ti,ab,kw or type ii diabet*:ti,ab,kw or noninsulin dependent:ti,ab,kw or type 2 diabet*:ti,ab,kw or Adult-Onset Diabetes Mellitus:ti,ab,kw (Word variations have been searched)	20935
#11	#9 or #10	20937
#12	#8 or #11	53777
#13	MeSH descriptor: [Health Education] explode all trees	11291
#14	MeSH descriptor: [Patient Education as Topic] explode all trees	7507
#15	MeSH descriptor: [Counseling] explode all trees	4026
#16	MeSH descriptor: [Rehabilitation Nursing] explode all trees	55
#17	Education*:ti,ab,kw or Community Health Education:ti,ab,kw or Self-Management*:ti,ab,kw or Cardiac Rehabilitation:ti,ab,kw (Word variations have been searched)	40078
#18	#13 or #14 or #15 or #16 or #17	42943
#19	#12 and #18 Publication Year from 2000 to 2016, in Cochrane Reviews (Reviews only) and Other Reviews	120
#20	Cochrane reviews + other reviews	**49 + 71=120**
	**Joanna Briggs Institute, Embase, MEDLINE**	
#1	Acute Coronary Syndrome.m_titl.	16727
#2	Myocardial Infarction.m_titl.	151398
#3	Unstable Angina.m_titl.	6933
#4	Coronary Artery Disease.m_titl.	54621
#5	Coronary Artery Bypass.m_titl.	30449
#6	Angina Pectoris.m_titl.	17997
#7	Coronary Heart Disease.m_titl.	35397
#8	#1 or #2 or #3 or #4 or #5 or #6	305035
#9	Type 2 Diabetes Mellitus.mp. [mp=ti, ab, ot, nm, hw, kf, px, rx, ui, an, tx, sh, ct, sa, tn, dm, mf, dv, kw]	64926

#10	"type 2 diabet*".m_titl.	102596
#11	"type ii diabet*".m_titl.	6139
#12	Adult-Onset Diabetes Mellitus.mp. [mp=ti, ab, ot, nm, hw, kf, px, rx, ui, an, tx, sh, ct, sa, tn, dm, mf, dv, kw]	307
#13	#9 or #10 or #11 or #12	136982
#14	#8 or #13	439243
#15	Health Education.mp. [mp=ti, ab, ot, nm, hw, kf, px, rx, ui, an, tx, sh, ct, sa, tn, dm, mf, dv, kw]	177285
#16	Patient Education.mp. [mp=ti, ab, ot, nm, hw, kf, px, rx, ui, an, tx, sh, ct, sa, tn, dm, mf, dv, kw]	185631
#17	Counseling.m_titl.	23527
#18	Social Support.m_titl.	11535
#19	"Education*".m_titl.	247901
#20	#15 or #16 or #17 or #18 or #19	577203
#21	#14 and #20	6480
#22	Systematic Review.mp. [mp=ti, ab, ot, nm, hw, kf, px, rx, ui, an, tx, sh, ct, sa, tn, dm, mf, dv, kw]	212014
#23	Meta-Analysis.mp. [mp=ti, ab, ot, nm, hw, kf, px, rx, ui, an, tx, sh, ct, sa, tn, dm, mf, dv, kw]	262009
#24	#22 or #23	381113
#25	#21 and #24	288
#26	limit 25 to English and yr="2000 - 2016"	**263**
	**Cumulative Index to Nursing and Allied Health Literature (CINAHL)**	
#1	TI Acute Coronary Syndrome OR AB Acute Coronary Syndrome	4826
#2	TI Myocardial Infarction OR AB Myocardial Infarction	24578
#3	TI Unstable Angina OR AB Unstable Angina	1353
#4	TI Coronary Artery Disease OR AB Coronary Artery Disease	12930
#5	TI Coronary Heart Disease OR AB Coronary Heart Disease	12321
#6	#1 OR #2 OR #3 OR #4 OR #5	46436
#7	TI Type 2 Diabetes Mellitus OR AB Type 2 Diabetes Mellitus	8952
#8	TI type 2 diabet* OR AB type 2 diabet*	33085
#9	TI Adult-Onset Diabetes Mellitus OR AB Adult-Onset Diabetes Mellitus	23
#10	#7 OR #8 OR #9	33097

#11	#6 OR #10	77923
#12	TI Health Education OR AB Health Education	53415
#13	TI Patient Education OR AB Patient Education	37789
#14	TI Counseling OR AB Counseling	17673
#15	TI Social Support OR AB Social Support	30093
#16	TI Education* OR AB Education*	188291
#17	#12 OR #13 OR #14 OR #15 OR #16	227269
#18	#11 AND #17	4707
#19	TI Systematic Review OR AB Systematic Review	48830
#20	TI Meta-Analysis OR AB Meta-Analysis	28776
#21	#19 OR #20	64611
#22	S18 AND S21 Limiters - Published Date: 20000101-20160431; English Language	**129**
	**Web of Science**	
#1	TS=("Acute Coronary Syndrome*") OR TS=("Myocardial Infarct*") OR TS=("Unstable Angina") OR TS=("Coronary Artery Disease*") OR TS=("Coronary heart disease*")<br>Indexes=SCI-EXPANDED, SSCI, A&HCI, CPCI-S, CPCI-SSH, BKCI-S, BKCI-SSH, ESCI, CCR-EXPANDED, IC Timespan=All years	404699
#2	TS=("Type 2 Diabetes Mellitus") OR TS=("type 2 diabete*") OR TS=("Noninsulin-Dependent Diabete*") OR TS=("Adult-Onset Diabetes Mellitus")<br>Indexes=SCI-EXPANDED, SSCI, A&HCI, CPCI-S, CPCI-SSH, BKCI-S, BKCI-SSH, ESCI, CCR-EXPANDED, IC Timespan=All years	103548
#3	#2 OR #1<br>Indexes=SCI-EXPANDED, SSCI, A&HCI, CPCI-S, CPCI-SSH, BKCI-S, BKCI-SSH, ESCI, CCR-EXPANDED, IC Timespan=All years	495081
#4	TS=("Health Education") OR TS=("Patient Education")<br>Indexes=SCI-EXPANDED, SSCI, A&HCI, CPCI-S, CPCI-SSH, BKCI-S, BKCI-SSH, ESCI, CCR-EXPANDED, IC Timespan=All years	29830
#5	#4 AND #3	1290

	Indexes=SCI-EXPANDED, SSCI, A&HCI, CPCI-S, CPCI-SSH, BKCI-S, BKCI-SSH, ESCI, CCR-EXPANDED, IC Timespan=All years	
#6	TS=("Systematic Review*") OR TS=("Meta-analysis*") Indexes=SCI-EXPANDED, SSCI, A&HCI, CPCI-S, CPCI-SSH, BKCI-S, BKCI-SSH, ESCI, CCR-EXPANDED, IC Timespan=All years	149663
#7	(#6 AND #5) AND LANGUAGE: (English) Indexes=SCI-EXPANDED, SSCI, A&HCI, CPCI-S, CPCI-SSH, BKCI-S, BKCI-SSH, ESCI, CCR-EXPANDED, IC Timespan=2000-2016	**68**
	**Journals@Ovid**	
#1	Acute Coronary Syndrome.m_titl.	3021
#2	Myocardial Infarction.m_titl.	24269
#3	Unstable Angina.m_titl.	1038
#4	Coronary Artery Disease.m_titl.	10106
#5	Coronary Artery Bypass.m_titl.	6546
#6	Angina Pectoris.m_titl.	1480
#7	Coronary Heart Disease.m_titl.	6487
#8	#1 or #2 or #3 or #4 or #5 or #6	51548
#9	Type 2 Diabetes Mellitus. m_titl.	5097
#10	"type 2 diabet*".m_titl.	26434
#11	"type ii diabet*".m_titl.	980
#12	Adult-Onset Diabetes Mellitus. m_titl.	391
#13	#9 or #10 or #11 or #12	27795
#14	#8 or #13	78792
#15	Health Education. m_titl.	1135
#16	Patient Education. m_titl.	1556
#17	Counseling.m_titl.	5532
#18	Social Support.m_titl.	2667
#19	"Education*".m_titl.	50950

#20	#15 or #16 or #17 or #18 or #19	58969
#21	#14 and #20	510
#22	Systematic Review.mp. [mp=ti, ab, ot, nm, hw, kf, px, rx, ui, an, tx, sh, ct, sa, tn, dm, mf, dv, kw]	117790
#23	Meta-Analysis.mp. [mp=ti, ab, ot, nm, hw, kf, px, rx, ui, an, tx, sh, ct, sa, tn, dm, mf, dv, kw]	215069
#24	#22 or #23	282984
#25	#21 and #24	61
#26	limit 26 to yr="2000 - 2016"	**58**

For peer review only

PRISMA 2009 Checklist

Section/topic	#	Checklist item	Reported on page #
**TITLE**			
Title	1	Identify the report as a systematic review, meta-analysis, or both.	1
**ABSTRACT**			
Structured summary	2	Provide a structured summary including, as applicable: background; objectives; data sources; study eligibility criteria, participants, and interventions; study appraisal and synthesis methods; results; limitations; conclusions and implications of key findings; systematic review registration number.	3-4
**INTRODUCTION**			
Rationale	3	Describe the rationale for the review in the context of what is already known.	5-6
Objectives	4	Provide an explicit statement of questions being addressed with reference to participants, interventions, comparisons, outcomes, and study design (PICOS).	6
**METHODS**			
Protocol and registration	5	Indicate if a review protocol exists, if and where it can be accessed (e.g., Web address), and, if available, provide registration information including registration number.	NA
Eligibility criteria	6	Specify study characteristics (e.g., PICOS, length of follow-up) and report characteristics (e.g., years considered, language, publication status) used as criteria for eligibility, giving rationale.	7
Information sources	7	Describe all information sources (e.g., databases with dates of coverage, contact with study authors to identify additional studies) in the search and date last searched.	6-7
Search	8	Present full electronic search strategy for at least one database, including any limits used, such that it could be repeated.	6-7
Study selection	9	State the process for selecting studies (i.e., screening, eligibility, included in systematic review, and, if applicable, included in the meta-analysis).	8
Data collection process	10	Describe method of data extraction from reports (e.g., piloted forms, independently, in duplicate) and any processes for obtaining and confirming data from investigators.	8-9
Data items	11	List and define all variables for which data were sought (e.g., PICOS, funding sources) and any assumptions and simplifications made.	8-9
Risk of bias in individual studies	12	Describe methods used for assessing risk of bias of individual studies (including specification of whether this was done at the study or outcome level), and how this information is to be used in any data synthesis.	8
Summary measures	13	State the principal summary measures (e.g., risk ratio, difference in means).	8-9
Synthesis of results	14	Describe the methods of handling data and combining results of studies, if done, including measures of consistency (e.g., I^2) for each meta-analysis.	9

For peer review only - http://bmjopen.bmj.com/site/about/guidelines.xhtml

PRISMA 2009 Checklist

Section/topic	#	Checklist item	Reported on page #
Risk of bias across studies	15	Specify any assessment of risk of bias that may affect the cumulative evidence (e.g., publication bias, selective reporting within studies).	8
Additional analyses	16	Describe methods of additional analyses (e.g., sensitivity or subgroup analyses, meta-regression), if done, indicating which were pre-specified.	8-10
**RESULTS**			
Study selection	17	Give numbers of studies screened, assessed for eligibility, and included in the review, with reasons for exclusions at each stage, ideally with a flow diagram.	9-11
Study characteristics	18	For each study, present characteristics for which data were extracted (e.g., study size, PICOS, follow-up period) and provide the citations.	9-12
Risk of bias within studies	19	Present data on risk of bias of each study and, if available, any outcome level assessment (see item 12).	12-13
Results of individual studies	20	For all outcomes considered (benefits or harms), present, for each study: (a) simple summary data for each intervention group (b) effect estimates and confidence intervals, ideally with a forest plot.	16-21
Synthesis of results	21	Present results of each meta-analysis done, including confidence intervals and measures of consistency.	16-21
Risk of bias across studies	22	Present results of any assessment of risk of bias across studies (see Item 15).	13-16
Additional analysis	23	Give results of additional analyses, if done (e.g., sensitivity or subgroup analyses, meta-regression [see Item 16]).	NA
**DISCUSSION**			
Summary of evidence	24	Summarize the main findings including the strength of evidence for each main outcome; consider their relevance to key groups (e.g., healthcare providers, users, and policy makers).	22,29-30
Limitations	25	Discuss limitations at study and outcome level (e.g., risk of bias), and at review-level (e.g., incomplete retrieval of identified research, reporting bias).	4,29-30
Conclusions	26	Provide a general interpretation of the results in the context of other evidence, and implications for future research.	31-32
**FUNDING**			
Funding	27	Describe sources of funding for the systematic review and other support (e.g., supply of data); role of funders for the systematic review.	32

From: Moher D, Liberati A, Tetzlaff J, Altman DG, The PRISMA Group (2009). Preferred Reporting Items for Systematic Reviews and Meta-Analyses: The PRISMA Statement. PLoS Med 6(7): e1000097. doi:10.1371/journal.pmed1000097

For more information, visit: **www.prisma-statement.org**.

For peer review only - http://bmjopen.bmj.com/site/about/guidelines.xhtml

BMJ Open

Health Education for Patients with Acute Coronary Syndrome and Type 2 Diabetes Mellitus: An Umbrella Review of Systematic Reviews and Meta-Analyses

Journal:	*BMJ Open*
Manuscript ID	bmjopen-2017-016857.R1
Article Type:	Research
Date Submitted by the Author:	22-Jun-2017
Complete List of Authors:	Liu, Xian-liang<br>Shi, Yan<br>Willis, Karen<br>Wu, Jo<br>Johnson, Maree; Australian Catholic University, Faculty of Health Sciences
**Primary Subject Heading**:	Evidence based practice
Secondary Subject Heading:	Cardiovascular medicine, Diabetes and endocrinology
Keywords:	Health Education, Acute Coronary Syndrome, Type 2 Diabetes Mellitus, Umbrella Review

SCHOLARONE™
Manuscripts

For peer review only

For peer review only - http://bmjopen.bmj.com/site/about/guidelines.xhtml

Title page

Health Education for Patients with Acute Coronary Syndrome and Type 2 Diabetes Mellitus: An Umbrella Review of Systematic Reviews and Meta-Analyses

Xian-Liang Liu, RN, MScN

Ph.D. Candidate, Tenth People's Hospital of Tongji University, China; Ph.D. Candidate, School of Nursing, Midwifery and Paramedicine, Australian Catholic University, Australia; Lecturer, School of Nursing, Jinggangshan University, China.

Address: 1100 Nudgee Road, Banyo, Brisbane, QLD, Australia

Email: liu.xianliang@myacu.edu.au; 1988_liuxianliang@tongji.edu.cn

Yan Shi, RN, Ph.D., Professor, Executive Director of Nursing Department

Tenth People's Hospital and Tongji University, China

Address: 301, YanChang Road, Shanghai, China

E-mail: shlzk2002@163.com

Karen Willis, Ph.D., Professor, Associate Dean (Learning and Teaching)

Faculty of Health Sciences, Australian Catholic University, Australia

Address: 250 Victoria Parade, Melbourne, Australia

Email: Karen.Willis@acu.edu.au

Chiung-Jung (Jo) Wu, RN, DrHlthSc, FACN

Associate Professor, School of Nursing, Midwifery and Paramedicine, University of the Sunshine Coast;

Visiting Fellow, School of Nursing, Queensland University of Technology;

Honorary Research Fellow, Royal Brisbane and Women's Hospital;

Honorary Research Fellow, Mater Medical Research Institute-University of Queensland (MMRI-

For peer review only - http://bmjopen.bmj.com/site/about/guidelines.xhtml

UQ), Brisbane, Australia;

Postal Address: ML 57, PO Box 1149, Hervey Bay Q 4655, Australia.

Email: cwu@usc.edu.au; c3.wu@qut.edu.au

Maree Johnson, RN, Ph.D., Professor, Associate Dean (Research)

Faculty of Health Sciences, Australian Catholic University, Australia

Address: Tenison Woods House (8 -20 Napier Street), North Sydney, Australia

Email: Maree.Johnson@acu.edu.au

Affiliated with the Ingham Institute of Applied Medical Research, Liverpool. Sydney.

Corresponding author:

Xian-Liang Liu, RN, MScN,

Ph.D. Candidate, School of Nursing, Midwifery and Paramedicine, Australian Catholic University, Australia; Ph.D. Candidate, Tenth People's Hospital of Tongji University, China; Lecturer, School of Nursing, Jinggangshan University, China.

Address: 1100 Nudgee Road, Banyo, Brisbane, QLD, Australia

Email: liu.xianliang@myacu.edu.au; 1988_liuxianliang@tongji.edu.cn

Tel: +61 497440830; +86 15921496130

For peer review only

Abstract

Objectives: This umbrella review aimed to identify the current evidence on health education-related interventions for patients with acute coronary syndrome or type 2 diabetes mellitus; identify the educational content, delivery methods, intensity, duration and setting required. The purpose was to provide recommendations for educational interventions for high risk patients with both acute coronary syndrome and type 2 diabetes mellitus.

Design: Umbrella review of Systematic Reviews and Meta-Analyses.

Setting: Inpatient and post discharge settings.

Participants: Patients with ACS and T2DM.

Data sources: CINAHL, Cochrane Library, Joanna Briggs Institute, Journals@Ovid, EMBase, Medline, PubMed, and Web of Science databases from January 2000 through May 2016.

Outcomes measures: Clinical outcomes (such as HbA1C), behavioral outcomes (such as smoking), psychosocial outcomes (such as anxiety) and medical service use.

Results: Fifty-one eligible reviews (15 for ACS and 36 for T2DM) consisting of 1324 relevant studies involving 288,057 patients (15 papers did not provide the total sample); 30 (58.8%) reviews were rated as high quality. Nurses only and multidisciplinary teams were the most frequent professionals to provide education, and most educational interventions were delivered post-discharge. Face-to-face sessions were the most common delivery formats, and many education sessions were also delivered by telephone or via web contact. The frequency of educational sessions was weekly or monthly, and an average of 3.7 topics was covered per education session. Psychoeducational interventions were generally effective at reducing smoking and admissions for patients with ACS. Culturally appropriate health education, self-management educational interventions, group medical visits and psychoeducational interventions were

For peer review only

generally effective for T2DM patients.

Conclusions: Results indicate that there is a body of current evidence about the efficacy of health education, its content, and delivery methods for patients with ACS or T2DM. These results provide recommendations about the content for, and approach to, health education intervention for these high risk patients.

KEY WORDS: Health Education; Acute Coronary Syndrome; Type 2 Diabetes Mellitus; Umbrella Review

Strengths and limitations of this review:

- This umbrella review is the first synthesis of systematic reviews or meta-analyses to consider health education-related interventions for patients with ACS or T2DM.
- These results provide recommendations about the content of a health education intervention for patients with ACS and T2DM.
- The diversity of the educational interventions seen in the reviews included in this umbrella review may reflect the uncertainty about the optimal strategy for providing health education to patients.
- This umbrella review found no reviews focused on patients with ACS and T2DM the intended target group; instead, all of the systematic reviews and meta-analyses focused on only one of these two diseases.

For peer review only

Introduction

Acute coronary syndrome (ACS) is the leading cause of death worldwide. The risk of high mortality rates relating to ACS is markedly increased after an initial cardiac ischemic event.[1] Globally, 7.2 million (13%) deaths are caused by coronary artery disease (CAD),[2] and it is estimated that more than 780,000 persons will experience ACS each year in the United States. [3] Moreover, about 20–25% of patients with ACS reportedly also have diabetes mellitus (DM); predominantly type 2 diabetes mellitus [T2DM]). [4 5] Patients with ACS and DM have an increased risk of adverse outcomes such as death, recurrent myocardial infarction (MI), readmission, or heart failure during follow up. [6] Longer median delay times from symptom onset to hospital presentation, have been reported among patients with ACS and DM than patients with ACS alone. [7]

DM is now considered to confer a risk equivalent to that of CAD for patients for future MI and cardiovascular mortality. [8] Mortality was significantly higher among patients with ACS and DM than among patients with ACS only following either ST segment elevation myocardial infarction (STEMI) (8.5% [ACS and DM] versus 5.4% [ACS]) or unstable angina/non-STEMI (NSTEMI) (2.1% [ACS and DM] vs. 1.1% [ACS]).[9] ACS and T2DM are often associated with high-risk factors such as low levels of physical exercise, obesity, smoking, and unhealthy diet. [10] Some of these and other risk factors, specifically glycemia, high blood pressure (BP), lipidemia, and obesity, are frequently addressed by health education interventions. [10]

Health education interventions are comprehensive programs that healthcare providers deliver to patients aimed at improving patients' clinical outcomes through the increase and maintenance of health behaviours.[11] Along with education about, for example, medication adherence, these programs seek to increase behaviors such as physical exercise and a healthy diet thus reducing

For peer review only - http://bmjopen.bmj.com/site/about/guidelines.xhtml

patient morbidity or mortality. [11] Most diabetes education is provided through programs within outpatient services or physicians' practices. [12] Many recent education programs have been designed to meet national or international education standards [13-15] with diabetes education being individualized to consider patients' existing needs and health conditions. [16] Patients with T2DM have reported feelings of hopelessness and fatigue with low levels of self-efficacy, after experiencing an acute coronary episode. [17]

Although there are numerous systematic reviews of educational interventions relating to ACS, or T2DM, an umbrella review providing direction on educational interventions for high risk patients with both ACS and T2DM is not available, indicating a need to gather the current evidence and develop an optimal protocol for health education programs for patients with ACS and T2DM. This umbrella review will examine the best available evidence on health education-related interventions for patients with ACS or T2DM. We will synthesize these findings to provide direction for health education-related interventions for high risk patients with both ACS and T2DM.

An umbrella review is a new method to summarize and synthesize the evidence from multiple systematic reviews/meta-analyses into one accessible publication. [18] Our aim is to systematically gather, evaluate and organize the current evidence relating the health education interventions for patients with ACS or T2DM, and proffer recommendations for the scope of educational content and delivery methods that would be suitable for patients with ACS and T2DM.

Methods

Data Sources

For peer review only - http://bmjopen.bmj.com/site/about/guidelines.xhtml

This umbrella review performed a literature search to identify systematic reviews and meta-analyses examining health education-related interventions for patients with ACS or T2DM. The search strategies are described in Supplementary Annex 1. This umbrella review searched eight databases for articles published from January 2000 to May 2016: CINAHL, Cochrane Library, Joanna Briggs Institute, Journals@Ovid, EMBase, Medline, PubMed, and Web of Science. The search was limited to English language only. The following broad MeSH terms were used: *acute coronary syndrome; angina, unstable; angina pectoris; coronary artery disease; coronary artery bypass; myocardial infarction; diabetes mellitus, type 2; counseling; health education; patient education as topic; meta-analysis [publication type];* and *meta-analysis* as a topic.

Inclusion Criteria

Participants

All participants were diagnosed with ACS or T2DM using valid, established diagnostic criteria. The diagnostic standards included those described by the American College of Cardiology or American Heart Association, [3] National Heart Foundation of Australia & Cardiac Society of Australia and New Zealand, [19] World Health Organization, [20] or other associations.

Intervention Types

For this umbrella review, health education-related interventions refer to any planned activities or programs that include behavior modification, counseling, and teaching interventions. Results considered for this review included changes in clinical outcomes (including blood pressure levels, body weight, diabetes complications, HbA1C, lipid levels, mortality rate and physical activity levels), behavioral outcomes (such as diet, knowledge, self-management skills, self-efficacy, and smoking), and psychosocial outcomes (such as anxiety, depression, quality of life, and stress) and medical service use (such as medication use, health care utilization, and cost-

For peer review only - http://bmjopen.bmj.com/site/about/guidelines.xhtml

effectiveness) for patients with ACS or T2DM. These activities or programs included any educational interventions delivered to patients with ACS or T2DM. The interventions are delivered in any format, including face-to-face, telephone and group-based or one-on-one, and the settings include community, hospital and home. The interventions were delivered by nurses (including diabetes nurse educators), physicians, community health care workers, dietitians, lay people, rehabilitation therapists, or multidisciplinary teams.

Study Types

Only systematic reviews and meta-analyses were included in this review.

Eligibility Assessment

The title and abstract of all of the retrieved articles were assessed independently by two reviewers (XL-L, YS) based on the inclusion criteria. All duplicate articles were identified within EndNote version X7 [21] and subsequently excluded. If the information from the titles and abstract was not clear, the full articles were retrieved. The decision to include an article was based on an appraisal of the full text of all retrieved articles. Any disagreements during this process were settled by discussion and, if necessary, consensus was sought with a third reviewer. We developed an assessment form in which specific reasons for exclusion were detailed.

Assessment of Methodological Quality

The methodological quality and risk of bias were assessed for each of the included publications using the Assessment of Multiple Systematic Reviews (AMSTAR),[22] independently by the same two reviewers (see Table 1). The AMSTAR is an 11-item tool, with each item provided a score of 1 (specific criterion is met) or 0 (specific criterion is not met, unclear, or not applicable). [22 23] An overall score for the review methodological quality is then calculated as the sum of the individual item scores: high quality, 8-11; medium quality, 4-7; or low quality, 0-3. [23] If the

For peer review only - http://bmjopen.bmj.com/site/about/guidelines.xhtml

required data were not available in the article, the original authors were contacted for more information. The low quality reviews (AMSTAR scale: 0-3) were excluded in this umbrella review.

Data Extraction

Data were independently extracted by two reviewers using a predefined data extraction form. For missing or unclear information, the primary authors were contacted for clarification.

Statistical Presentation of Results from Reviews

All of the results were extracted for each included systematic review or meta-analysis, and the overall effect estimates are presented in a tabular form. The number of systematic reviews or meta-analyses that reported the outcome, total sample (from included publications), and information of health education interventions is also presented in the Tables (Table 2, 3). [24] A final 'summary of evidence' was developed to present the intervention, included study synthesis, and indication of the findings from the included papers (Table 4). [24] This umbrella review calculated the Corrected Covered Area (CCA) (Appendix 2, 3). The CCA statistic is a measure of overlap of trials (the repeated inclusion of the same trial in subsequent systematic reviews included in an umbrella systematic review). A detailed description of the calculation is provided by the authors who note slight CCA as 0-5%, moderate CCA as 6-10%, high CCA as 11-15% and very high CCA is more than 15%. [25] The lower the CCA the lower the likelihood of overlap of trials included in the umbrella review.

Synthesizing the Results and Rating the Evidence for Effectiveness

The statements of evidence were based on a rating scheme to gather and rate the evidence across the included publications.[26] The statements of evidence were based on the following rating

For peer review only - http://bmjopen.bmj.com/site/about/guidelines.xhtml

scheme: *sufficient evidence*, sufficient data to support decisions about the effect of the health education-related interventions. [26] A rating of *sufficient evidence* in this review is obtained when systematic reviews or meta-analyses with a large number of included articles or participants produce a statistically significant result between the health education group and the control group. [26] *Some evidence*, is a less conclusive finding about the effects of the health education-related interventions[26] with statistically significant findings found in only a few included reviews or studies. *Insufficient evidence*, refers to not enough evidence to make decisions about the effects of the health education-related interventions, such as non-significant results between the health education group and the control group in the included systematic reviews or meta-analyses. [26] *Insufficient evidence to determine*, refers to not enough pooled data to be able to determine whether of the health education-related interventions are effective or not based on the included reviews. [26]

Results

Characteristics of Included reviews

The selection process and number of studies at each step was illustrated as presented in Figure 1. The database search yielded 692 publications, with removal of 197 duplicates and 371 articles that did not meet the inclusion criteria, 124 full-text articles were retrieved after applying the methodological quality rating (AMSTAR scale), and three studies [27-29] were removed due to low scores ≤3 on the AMSTAR scale. Fifty-one systematic reviews or meta-analyses [30-80] conducted between 2001 and 2016 and published in English were included (Figure 1; Tables 1 –3); 15 relating to ACS. The overlap of the trials included in the 15 reviews and meta-analyses related to ACS was slight (CCA = 2.6%). For the 36 systematic reviews relating to T2DM, the overlap of trials within these 35 reviews and meta-analyses (one review [47] did not report the included

For peer review only - http://bmjopen.bmj.com/site/about/guidelines.xhtml

studies) was slight (CCA = 2.1%). None of the articles included patients with both ACS and T2DM. The umbrella review involved a total of 277,493 patients, including 225,034 patients with coronary heart disease or ACS (one article did not report the total sample) and 52,459 patients with T2DM (16 papers did not report the total sample). The average sample size of included articles was 8,161 (range, 536–68,556) participants, however, 63 studies related to ACS and 177 studies related to T2DM, were included in more than one systematic review or meta-analysis (see Appendix 2 and 3 and CCA statistics). The sample of these studies would therefore be included more than once. Of the included systematic reviews or meta-analyses, eleven were published in *The Cochrane Library*. Nine of the articles described meta-analyses, 29 articles described systematic reviews, and the remaining 13 articles were described as systematic reviews and meta-analyses or meta-regressions or narrative reviews.

Electronic database searches were conducted for all systematic reviews or meta-analyses, with an average of 6 databases searched (range, 2–16). The dates searched ranged widely, from inception of the database through December 2014. Most of the included reviews were randomized controlled trials (RCTs), and an average of 25.6 (range, 7–132) studies was included per systematic review or meta-analyses. Of the total, 818 unique (non-repeated) studies were included in all of the reviews or meta-analyses, 286 included patients with ACS, and 532 included patients with T2DM (Appendix 2, 3). The included reviews assessed the risk of bias using the Cochrane risk of bias tool (22 publications), JADA quality score (seven publications), Joanna Briggs quality assessment tool (two publications), PEDro scale (one publication), RCT Critical Appraisal Skills Programme (one publication), and the SIGN-50 checklist (one publication).

Methodological Quality of Included Systematic Reviews and Meta-Analyses

For peer review only - http://bmjopen.bmj.com/site/about/guidelines.xhtml

The methodological quality of the included publications is presented in Table 1. Thirty (58.8%) publications were classified as high quality (scores 8–11), and 21 (41.2%) publications were classified as medium quality (scores 4–7). Twenty five (49%) reviews specifically provided an *a priori* design, while the use of such a design was unclear for 26 (51%) publications. The inclusion of other forms of literature (such as grey literature) was described in 18 (35%) reviews. Only 14 out of 51 (27%) reviews included a table of included and excluded studies. Only 2 (4%) reviews did not provide a characteristics table of the included papers. The scientific quality of the included papers was evaluated and documented in 47 (92%) reviews. The scientific quality of the included studies was used appropriately to formulate conclusions in 47 (92%) reviews. The methods to combine the results of the included studies were appropriate in 43 (86%) reviews. Publication bias was assessed in only 19 (37%) reviews. Finally, conflicts of interest were reported in 47 (92%) reviews.

Characteristics of Health Educational Interventions

The description of the health educational interventions followed the Workgroup for Intervention Development and Evaluation Research (WIDER) reporting guidelines for behavior change interventions. [81] The characteristics of the recipients, setting, delivery methods, intensity, duration, and educational content of health educational interventions for patients with ACS or T2DM are summarized in Tables 2 and 3. The delivery strategies for health education included face-to-face, internet-based, phone-based, videotape, written educational materials, or mixed. The format included one-on-one (individualized), group, or both. Face-to-face sessions were the most common delivery formats, and many education sessions were also delivered by telephone/web contact or individualized counseling. The number of sessions, total contact hours, and durations varied, and there was limited information about the intensity of health education

For peer review only

for patients provided. The frequency of educational sessions was weekly or monthly, and an average of 3.7 topics was covered per education session. Nurses and multidisciplinary teams were the most frequent educators, and most education programs were delivered post-discharge.

Acute Coronary Syndrome

The educational content for patients with ACS covered cardiovascular risk factors in 8 reviews (53.33%), psychosocial issues in 8 reviews (53.33%), smoking cessation in 6 reviews (40.00%), exercise in 5 reviews (33.33%), behavioral change in 5 reviews (33.33%), diet in 4 reviews (26.67%), self-management in 3 reviews (20.00%), and medication in 1 review (6.67%). Two reviews only included smoking cessation and cardiovascular risk factors. The most common educational providers were nurses and a multidisciplinary team. Six studies [31 36 48 51 56 69] (6/15, 40%) described the theoretical approach that underpinned the education intervention.

Type 2 Diabetes Mellitus

The educational content for patients with T2DM included diet in 23 reviews (63.89%), behavioral change in 21 reviews (58.33%), self-management in 20 reviews (55.56%), exercise in 17 reviews (47.22%), glycemic control in 16 reviews (44.45%), medication in 13 reviews (36.11%), psychosocial issues in 9 reviews (25.00%), smoking cessation in 2 reviews (5.56%), cardiovascular risk factors in 2 reviews (5.56%), and DM risks in one review (2.78%). The most common providers were dieticians, nurses, and a multidisciplinary team. The number of sessions, total contact hours, and durations varied. Thirteen reviews [30 33 43 49 52-54 60 64 67 75-77] (13/36, 36.11%) described the theoretical approach that underpinned the education intervention.

Effect of Interventions

The outcomes of the included systematic reviews and meta-analyses were summarized in Table

For peer review only - http://bmjopen.bmj.com/site/about/guidelines.xhtml

4.

Patients with Acute Coronary Syndrome

Three major types of health education-related interventions were used for patients with ACS: general health education (only included general health information), psychoeducational interventions, and secondary prevention educational interventions (including strategies to promote a healthy lifestyle, manage medications, and reduce cardiovascular complications) as well as internet-based interventions.

General Health Education

The findings are based on our synthesis of the findings from 6 systematic reviews. [37 48 50 51 59 70] Overall, there were mixed effects of general health education on behavioral change or clinical outcomes in patients with ACS. There was *some evidence* of a positive effect of general health education on knowledge, behavior, psychosocial indicators, beliefs, and risk factor modification, but no effects for key clinical outcomes, such as cholesterol level, hospitalization, mortality, MI, and revascularization. The results for health-related quality of life, healthcare utilization, and costs were mixed; several reviews reported a significant change, and other reviews reported no significant change for these outcomes. Only one review focused on telephone-based health education. There is *some evidence* that telephone-based health education during cardiac rehabilitation might improve all-cause hospitalization, anxiety, depression, smoking cessation, and systolic BP but there is no evidence for improvements in all-cause mortality and reductions in low-density lipoprotein cholesterol. [59]

Psychoeducational Interventions

Strategies for psychoeducational interventions have a specific focus on smoking cessation, and

For peer review only - http://bmjopen.bmj.com/site/about/guidelines.xhtml

depression. The findings are based on synthesis of results from six publications. [31 35 36 45 56 69] There is *sufficient evidence* that psychoeducational programs are effective at decreasing smoking, achieving smoking abstinence, and reducing depression. One review reported no effect on smoking cessation [31] or total mortality [56].

Secondary Prevention Educational Interventions

The following statements are based on our synthesis of results from three papers. [34 41 44] There is *some evidence* that secondary prevention educational interventions reduce MI readmission rates and improve quality of life, but the intervention was ineffective in reducing revascularization, cholesterol levels, and improving smoking cessation rates. The results are mixed for mortality and re-infarction rates; two reviews [34 41] found positive effects on mortality, while one review [44] did not.

Patients with Type 2 Diabetes Mellitus

Ten types of health education-related interventions were used for patients with T2DM: culturally appropriate health education (tailored to the religious beliefs, culture, literacy, and linguistics of the geographical area), dietary advice, foot health education, group medical visits (a group education component taught by health professionals), general health education (only included general health information), improving compliance with taking medications (e.g., promoting oral hypoglycemic adherence), lifestyle interventions (specific focus on dietary changes and increased physical activity, or stress management), psychoeducational interventions and self-management educational interventions (activities that promote or maintain the behaviors to manage T2DM often based on the National Standards for Diabetes Self-Management Education [13]), and therapeutic education (collaborative process needed to modify behavior and more effectively manage risk factors).

For peer review only - http://bmjopen.bmj.com/site/about/guidelines.xhtml

Culturally Appropriate Health Education

Findings are based on our synthesis of results from 8 publications. [33 42 52-54 58 62 72] Overall, there was *some evidence* of the effects of culturally appropriate health education on clinical outcomes for T2DM. There was *sufficien*t evidence that culturally appropriate health education improves HbA1c control and knowledge scores. There is *some* evidence that physical activity and clinical outcomes (blood glucose, HbA1c, BP) were improved. There were no data relating to adverse events during the intervention and follow-up (such as hypoglycemic events and mortality), and there was insufficient evidence about improvements in quality of life.

General Health Education

The statements are based on our synthesis of results from five papers. [40 46 60 74 79] Overall, there were mixed effects of general health education programs on clinical outcomes for T2DM, including HbA1C, cholesterol level, and triglyceride level. There was *some evidence* of the effectiveness of general health education on glycemic control, weight reduction, and some diabetes control outcomes (HbA1C, diabetes complications). There were no data supporting the effectiveness of general health education on reduced health service utilization, diabetes complications, improved knowledge, psychosocial outcomes, or smoking habits.

Lifestyle Interventions

The following statements are based on our synthesis of results from 6 reviews. [39 49 55 71 72 77] Overall, there were mixed effects of the lifestyle interventions on cholesterol level, HbA1C level, and weight control. There is *some evidence* that lifestyle interventions or behavioral programs are effective for blood glucose and BP management, but they were ineffective for reductions in HbA1c scores [71 72].

For peer review only - http://bmjopen.bmj.com/site/about/guidelines.xhtml

Improving Adherence to Medication Regimes

The statements are based on our synthesis of results from three publications. [57 78 80] There is *some evidence* of the effectiveness of improving adherence to taking medications for HbA1C control including oral hypoglycemic agents.

Self-Management Educational Interventions

The statements are based on our synthesis of results from 9 reviews. [43 47 61 64 65 67 68 75 76] Overall, there was *sufficient evidence* of the effects of self-management education interventions on HbA1c control, knowledge, lifestyle outcomes, and main psychosocial outcomes. However, there was *insufficient* evidence of the benefits of this education intervention on depression, quality of life, and weight control.

Other Health Education-Related Interventions

Other health education-related interventions for patients with T2DM included therapeutic education, foot health education, group medical visits, psychoeducational interventions, and dietary advice. Statements for all of these interventions are based on our synthesis of results from one review.

There is *some evidence* that foot health education is effective in reducing the incidence of lower extremity amputation. [32] There is *some evidence* that group medical visits are effective for improving HbA1c control and systolic BP management [38]. There is also *some evidence* that psychoeducational programs are effective for improving HbA1c control and psychological status. [30]

Finally, there is *insufficient evidence* that dietary advice improves glycemic and weight control or reduces micro- and macrovascular diseases. [63] There is also *insufficient evidence* for the cost-

For peer review only - http://bmjopen.bmj.com/site/about/guidelines.xhtml

effectiveness of therapeutic education for patients with T2DM. [66]

Discussion

This umbrella review identified 51 systematic reviews or meta-analyses (15 for ACS and 36 for T2DM) that assessed the outcomes of various aspects (such as the duration, contact hours, educational content, delivery mode) of the delivery of health education-related interventions relevant to high risk patients with ACS and T2DM. Health education has become an integral part of the management for people with ACS and T2DM. The most appropriate focus of the education provided to ACS patients with T2DM remains largely undefined in the literature. For example, it remains unknown if the focus should be primarily on cardiovascular risk factors, blood glucose monitoring, or all educational components for patients with both conditions. [70 76] In addition, should cardiovascular risk factors be the focus during the acute inpatient stay with other educational needs such as the smoking cessation occurring within the primary care or outpatient settings. [31 69 70]

It remains challenging to determine the specific strategy or format that is the most effective delivery mode for patients with ACS or T2DM. There is very limited evidence to guide clinicians on the duration, contact hours, educational content, delivery mode, total length, and setting of health education programs for cardiac patients. [50] For patients with DM, one study reported that more successful programs were longer than 6 months (longer duration), consisted of greater than 10 contact sessions (high intensity), and were one-on-one sessions with individualized assessment. [82]

Use of Theoretical Orientation to Develop Educational Intervention

For patients with Acute Coronary Syndrome

For peer review only - http://bmjopen.bmj.com/site/about/guidelines.xhtml

Use of theory when designing behavioral change interventions may also influence effectiveness.[75] Health education using a cognitive behavioral strategy is most consistently effective in changing maladaptive illness beliefs, [51] and studies using more than 2 behavioral change strategies reported significant differences between the intervention and control groups. [31] In one review, a significant change in smoking cessation was not observed in subgroup analyses between studies that did or did not report using a theory in intervention planning; [56] however, the authors did not suggest that using a theory in program planning should be disregarded but reported that examining actual theories or mechanisms underlying health education programs is required. [56] Owing to the considerable overlap between different theories and the detailed description of the theoretical approach in only approximately 40% of the included papers, it is difficult to determine the most effective theoretical approach, but many models can be used with success, such as the health belief model (HBM), social cognitive theory (SCT) and transtheoretical model (TTM). [56 67 69 75] Three reviews [31 41 44] noted that some included studies used behavioural strategies such as goal setting. These strategies were found to be beneficial for patients with coronary heart disease.

For patients with Type 2 Diabetes Mellitus

Although the theoretical approach underpinning the health education program was not always described, 13 of the 36 reviews (36.11%) related to T2DM reported the theoretical approach used in their included studies. The most common theories were SCT (including self-efficacy), empowerment theories (eg., Empowerment Behavior Change Model, Self-determination and Autonomy Motivation Theory, Middle-range Theory of Community Empowerment) and TTM. There is evidence that health education interventions based on a theoretical model are likely to be effective. [43] Vugt et al suggested that self-care education programs should be based on

For peer review only - http://bmjopen.bmj.com/site/about/guidelines.xhtml

theories and that theory-based self-care interventions are more effective than non-theory-based programs. [75 83] Theories could help to specify the key target health behaviors and behavioral change techniques required to generate the desired outcomes. [75] The decision regarding the theory should be based on the aim of the program and factor for intervention. [77] Only one review reported that a theoretical approach underpinning the health education program is not necessary for better outcomes. [76] Fourteen reviews [30 33 40 46 52 57 60 63 64 67 68 73 75 77] reported that goal setting was conducted in the included studies. Goal setting by patients, health professionals or mutually-agreed goals were linked to improved patient outcomes.

Educational Content

For Patients with Acute Coronary Syndrome

Most reviews reported that the educational content of the interventions was comprehensive. The most common topics, of the average 3.7 topics per education session, were behavioral change, cardiovascular risk factors management, exercise, psychosocial issues, and smoking cessation. An underlying principle of health education for patients with ACS is that knowledge is necessary, but not enough to develop health behaviors and change risk factors. [31 50] Age, cognitive factors, environmental factors, and social and economic background are also important considerations. [50] While interventions using a behavioral program, telephone-based content, or self-care are effective for smoking cessation, there was insufficient evidence to support that any type of educational program was more efficacious than the others. [69] Psychoeducation, which is defined as multimodal, educationally based, self-management interventions,[31] led to enhanced physical activity levels within 6–12 months when added to cardiac rehabilitation (CR) and was more effective than an exercise program or health education alone. [31 56] Moreover, psychoeducational interventions were more effective for patients with ACS than other types of

For peer review only - http://bmjopen.bmj.com/site/about/guidelines.xhtml

health education. [31 56]

For Patients with Type 2 Diabetes Mellitus

The educational content for patients with T2DM focused more on behavioral change, diet, exercise, glycemic control, medication, and self-management. Health education that was self-management was more effective for patients with T2DM. [40 47] In addition, based on the current evidence, the educational content should be culturally sensitive, especially for patients with T2DM; [33 42 54] culturally appropriate diabetes health education may have a greater impact on glycemic control and reduce diabetes complications. [77] The educational interventions for patients with T2DM focused primarily on HbA1C, lipid levels, quality of life, and body weight. HBM and SCT were the most common theories used in the included reviews.

Teaching Strategies and Outcomes

For Patients with Acute Coronary Syndrome

Most reviews reported that the education was provided using multiple teaching methods and in multiple settings. Nurses and multidisciplinary teams were the most frequent people providing education, and most education programs were delivered post-discharge. Although face-to-face sessions were the most common delivery format, many education sessions were also delivered by telephone or through individualized counseling. Telephone-based health education appeared to be effective for reducing hospitalizations, systolic BP, smoking rates, depression, and anxiety. [59] The educational interventions for patients with ACS focused primarily on clinical outcomes (hospitalization and mortality), modifiable risk factors (BP, low-density lipoprotein levels, and smoking cessation), and other psychological outcomes (anxiety and depression).

For Patients with Type 2 Diabetes Mellitus

For peer review only - http://bmjopen.bmj.com/site/about/guidelines.xhtml

Mixed health education programs generally included group sessions combined with educator-facilitated individual sessions, covering basic knowledge and problem-solving skills. These programs produced greater benefits and larger effect sizes for blood glucose control and knowledge level in patients with T2DM. [47] In contrast, individual education programs have been reported as more effective in achieving outcomes than group-based education. This may be because education programs might be more efficient at addressing personal needs, with greater participant engagement. [73] However, one systematic review reported that individual and group patient education, demonstrated similar outcomes among T2DM patients. [46]

Although face-to-face sessions were the most common delivery format, many education sessions were also delivered by telephone or individualized counseling. Face-to-face health education programs were most effective for enhancing blood glucose control and knowledge levels, while mixed delivery models (face-to-face, phone contact, online or web-based, or video) produced a moderate effect for knowledge levels. [47] Another review reported that face-to-face health education programs generated a greater benefit for metabolic control than those delivered using electronic communication technology. [73]

Nurses (including diabetes nurses educators), community workers, dieticians and multidisciplinary teams were the most frequent educators, and most of the education programs were delivered post-discharge. Some reviews indicated that health education programs delivered by a group of different educators, with some degree of education reinforcement at additional points of contact, may provide the best results. [60 76] However, based on two studies that reported HbA1C at 12 months, it is indicated that the outcomes in studies with only a diabetes nurse as the educator also tended to do better than the outcomes in studies with a multidisciplinary team , while the biggest effect was seen when a dietician was the only educator. [76] Health education

For peer review only - http://bmjopen.bmj.com/site/about/guidelines.xhtml

programs delivered by one person may focus more on the patient's ability than the educational content or quality of the health education programs. [76] However, no clear conclusion can be drawn whether having one educator delivering the intervention is best due to few information. [60]

Delivery, Timing and Follow up

For Patients with Acute Coronary Syndrome

Most educational sessions were delivered weekly. Few reviews provided information regarding the duration of education interventions; when the duration was reported, it varied from 4 weeks to 48 months. These findings suggest that there is a significant gap in the evidence in relation to the duration, contact hours, educational content, optimal delivery mode, total length, and setting of health education programs for cardiac patients. [50] For patients with ACS, one systematic review that included 7 studies with a total of 536 participants reported that studies with education lasting at least 6 months resulted in the most significant changes in the primary outcomes (such as behavioral change, smoking cessation) [31] and that at least 12 months of follow-up is needed to evaluate the impact of telephone-based education. [59] Another review reported that the intensity of education programs is important for efficacy regarding smoking cessation: interventions with a very low intensity and brief interventions, do not have a significant effect, [69] and programs for smoking cessation among patients with coronary heart disease should last >1 month. [69] Most of the reviews were provided for patients with ACS in inpatient settings and then within post discharge settings, five reviews [31 36 45 48 59] did not explicitly state the settings in which the health education related interventions were provided.

For Patients with Type 2 Diabetes Mellitus

Education sessions were delivered weekly or monthly. Longer health education programs for T2DM (>6 months) produced larger effects for all primary outcomes (such as HbA1C). [47]

For peer review only - http://bmjopen.bmj.com/site/about/guidelines.xhtml

Health education lasting >3 months resulted in the largest effect size compared with health education of a shorter duration (<3 months). [33] For HbA1C, the effect size at 6 months seemed to be significantly greater than at 3 and 12 months; in other words, the effect size peaked at 6 months. [62] In general, health education of a greater intensity (longer duration and more sessions) was more effective for blood glucose control and knowledge level among patients with T2DM.[47 74] Compared with health education programs covering only one topic, programs that included multiple or mixed educational topics yielded consistently greater benefits in blood glucose control and knowledge levels. [47] In addition, health education programs combined with specific behavioral change strategies (such as self-care strategies) seemed more effective than other programs. [47] Health education related interventions were mainly delivered in hospital settings, primary care settings, diabetes centers or community based settings; although six reviews [32 39 55 58 67 72] did not explicitly state the delivery settings.

Recommendations about Health Education Interventions for Patients with ACS and T2DM

These results from included systematic reviews and meta-analyses help to provide recommendations about the content of a health education intervention for patients with ACS and T2DM, requiring further evaluation. Future development of educational programs for patients with ACS and T2DM by health care professionals should consider the needs of people with these diseases. [37 40 42 70] Based on the results and findings from this umbrella review, recommendations are made in Table 5. The acute life-threatening nature of ACS requires that increased emphasis should be placed on cardiovascular risk factors in any combined education program. Both ACS and T2DM have common lifestyle factors such as inactivity and high fat diet requiring modifications.

Overall Completeness and Applicability of Evidence

This overview potentially provides an estimate with the lowest level of bias for the impact of health education-related interventions for patients with ACS or T2DM and could be regarded as an all-inclusive summary of the current evidence base for health education for these patients. While this umbrella review identified evidence for each of the types of health education, there was only a small number of reviews within some categories (such as psychoeducational intervention [30] and dietary advice [63]), and these studies were not very informative. This umbrella review also found no reviews that systematically analyzed varying doses of health education; therefore, could not examine the dose-response effects. There was insufficient information about the evaluated doses (total contact hours and duration of education) to enable comparison of the benefits of differences in the magnitude of the doses across the different research. This umbrella review found no reviews focused on patients with ACS and T2DM; instead, all of the systematic reviews and meta-analyses focused on only one of these diseases.

Quality of the Evidence

The methodological quality of the included systematic reviews and meta-analyses varied. All of the included reviews or meta-analyses were of moderate to high methodological quality, as assessed using AMSTAR. However, only 30 (58.8%) systematic reviews or meta-analyses were rated as high quality and only three (5.9%) systematic reviews or meta-analyses [43 53 69] adequately met all 11 AMSTAR criteria. This indicates that some of the reviews included in this umbrella review may have limitations in their design, conduct, and/or reporting that could have influenced the findings when considered both individually and collectively. [32 65]

The quality of the primary studies in the included systematic reviews or meta-analyses also varied. The main sources of bias were inadequate reporting of allocation concealment and randomization processes, as well as lack of outcome blinding. [33 42 69 70] This bias in the

For peer review only - http://bmjopen.bmj.com/site/about/guidelines.xhtml

methodological quality led to lower quality assessments, which varied by results within each included review. Other reasons for lower methodological quality included heterogeneity in, or inconsistency of, the effect and imprecise findings. Heterogeneity between studies in this umbrella review was described in terms of the intervention, participant characteristics and length of follow-up. Heterogeneity was an important factor indicating the complexity of the health education interventions. [56] The variability in the approaches, tools or scales used to measure outcomes between the included studies are likely to introduce some heterogeneity. [30] The heterogeneity of the educational interventions seen in the reviews included in this umbrella review may reflect the uncertainty about the optimal strategy for providing health education to patients. [37] In addition, 240 studies were included more than once in the included reviews and meta-analyses. However, the overall overlap of studies among reviews and meta-analyses related ACS and T2DM was slight, CCA of 2.6% and 2.1%, respectively. [25]

This umbrella review is the first synthesis of systematic reviews or meta-analyses to take a broad perspective on health education-related interventions for patients with ACS or T2DM. Given that health education is complex, the biggest challenge for systematic reviews or meta-analyses of health education is accounting for the potential clinical heterogeneity in health education-related interventions (content and delivery approaches) and the population of patients who receive health education. To facilitate comparisons across systematic reviews of health education and the efficient future update of this umbrella review, future reviews or meta-analyses need high-quality research and to standardize their design and reporting, including the reporting of included study characteristics, assessment criteria for risk of bias, outcomes, and methods to synthesize evidence synthesis.

Conclusions

For peer review only - http://bmjopen.bmj.com/site/about/guidelines.xhtml

For clinicians providing educational interventions to individuals with ACS and T2DM, the results from this review provide a contemporaneous perspective on current evidence on the effectiveness of health education (its content, and delivery methods) for this high risk patient group. The current evidence compiled by this umbrella review supports current international clinical guidelines, that theoretically-based education interventions lasting 6 months, delivered in multiple modes (face-to-face, phone contact, online or web-based, or video), and with individualized education delivered weekly, are more likely to generate positive outcomes. This review also supports health education- related interventions provided by health professionals, including nurses and multidisciplinary teams, delivering content including specific clinical factors for ACS and T2DM (BP, glycemic control, and medication), modifiable risk factors (unhealthy diet, inactivity, and smoking), and other psychological factors (anxiety and depression). These health education interventions could be delivered post-discharge, such as rehabilitation centers, primary care centers, and the community and should be at least 6 months in duration. The effectiveness of these programs was based on HbA1C levels, knowledge, psychosocial outcomes, readmission rates, and smoking status rather than clear evidence of reduced mortality, MI, or short and long-term complications. In addition, psychoeducational interventions were more effective for patients with ACS, and health education that was culturally appropriate or taught self-management was more effective for patients with T2DM. We also found that longer durations and high-intensity health education provided in an individualized format were more helpful for patients with ACS or T2DM.

The fact that none of the included reviews included patients with both ACS and T2DM indicates a clear need for further rigorous experimental studies with patients with both diseases. Future research that includes these aspects of education are likely determine the effectiveness of

For peer review only - http://bmjopen.bmj.com/site/about/guidelines.xhtml

educational interventions focusing on cardiovascular and DM risk factors and complications within patients with ACS and T2DM.

Acknowledgements: I would like to thank the authors of the original articles who provided additional unpublished data.

Author contributions:

Study conception and design: XL-L, MJ, KW, C-JW, YS. Data collection: XL-L, YS. Data analysis: XL-L, YS, MJ, KW, C-JW. Manuscript drafts: XL-L, MJ, C-JW, KW, YS.

Conflicts of interest: None declared.

Founding statement: This research received no specific grant from any funding agency in the public, commercial or not-for-profit sectors. The lead author is a recipient of an Australian Catholic University Faculty of Health Sciences Tongji University Cotutelle Ph.D. Scholarship.

Data sharing statement: No additional data are available.

For peer review only

References

[1]. Scarborough P, Wickramasinghe K, Bhatnagar P, et al. Trends in coronary heart disease, 1961-2011. *British Heart Foundation*, London (2011).

[2]. Finegold JA, Asaria P, Francis DP. Mortality from ischaemic heart disease by country, region, and age: statistics from World Health Organisation and United Nations. *Int J Cardiol* 2013;168(2):934-45.

[3]. Amsterdam EA, Wenger NK, Brindis RG, et al. 2014 AHA/ACC guideline for the management of patients with non–ST-elevation acute coronary syndromes: a report of the American College of Cardiology/American Heart Association Task Force on Practice Guidelines. *J Am Coll Cardiol* 2014;64(24):e139-e228.

[4]. Kahn M, Wheatcroft S. Acute coronary syndromes in diabetes. In: Fisher M (ed.). Heart Disease and Diabetes. Oxford University Press, Oxford, 2008.

[5]. Hasin T, Hochadel M, Gitt AK, et al. Comparison of treatment and outcome of acute coronary syndrome in patients with versus patients without diabetes mellitus. *Am J Cardiol* 2009;103(6):772-78.

[6]. Franklin K, Goldberg RJ, Spencer F, et al. Implications of diabetes in patients with acute coronary syndromes: the Global Registry of Acute Coronary Events. *Arch Intern Med* 2004;164(13):1457-63.

[7]. Ting HH, Chen AY, Roe MT, et al. Delay From Symptom Onset to Hospital Presentation for Patients With Non–ST-Segment Elevation Myocardial Infarction. *Arch Intern Med* 2010;170(20):1834-41.

[8]. Saely CH, Aczel S, Koch L, et al. Diabetes as a coronary artery disease risk equivalent: before a change of paradigm? *Eur J Cardiovasc Prev Rehabil* 2010;17(1):94-99.

[9]. Donahoe SM, Stewart GC, McCabe CH, et al. Diabetes and mortality following acute coronary syndromes. *JAMA* 2007;298(7):765-75.

[10]. Lakerveld J, Bot SD, Chinapaw MJ, et al. Motivational interviewing and problem solving treatment to reduce type 2 diabetes and cardiovascular disease risk in real life: a randomized controlled trial. *Int J Behav Nutr Phys Act* 2013;10(1):1.

[11]. Glanz K, Rimer BK, Viswanath K. Health behavior and health education: theory, research, and practice: John Wiley & Sons 2008.

[12]. Powers MA, Bardsley J, Cypress M, et al. Diabetes self-management education and support in type 2 diabetes: a joint position statement of the American Diabetes Association, the American Association of Diabetes Educators, and the Academy of Nutrition and Dietetics. *Diabetes Educ* 2017;43(1):40-53.

For peer review only - http://bmjopen.bmj.com/site/about/guidelines.xhtml

[13]. Haas L, Maryniuk M, Beck J, et al. National standards for diabetes self-management education and support. *Diabetes Educ* 2012;38(5):619-29.

[14]. Fisher EB, Boothroyd RI, Coufal MM, et al. Peer support for self-management of diabetes improved outcomes in international settings. *Health Aff* 2012;31(1):130-39.

[15]. Tang PC, Overhage JM, Chan AS, et al. Online disease management of diabetes: engaging and motivating patients online with enhanced resources-diabetes (EMPOWER-D), a randomized controlled trial. *J Am Med Inform Assoc* 2013;20(3):526-34.

[16]. Association AD. Standards of medical care in diabetes—2014. *Diabetes Care* 2014;37 (Supplement 1):S14-S80.

[17]. Chang AM, McDowell J. Perspectives of patients with type 2 diabetes following a critical cardiac event–an interpretative approach. *J Clin Nurs* 2008;17(5a):16-24.

[18]. Becker LA OA. Chapter 22: overviews of reviews.In: Higgins JPT, Green S (editors). Cochrane Handbook for Systematic Reviews of Interventions Version 5.1.0 [updated March 2011]. *The Cochrane Collaboration* 2011

[19]. Chew DP, Scott IA, Cullen L, et al. National Heart Foundation of Australia and Cardiac Society of Australia and New Zealand: Australian clinical guidelines for the management of acute coronary syndromes 2016. *Heart Lung Circ* 2016;25(9):895-951.

[20]. Alberti KGMM, Zimmet Pf. Definition, diagnosis and classification of diabetes mellitus and its complications. Part 1: diagnosis and classification of diabetes mellitus. Provisional report of a WHO consultation. *Diabet Med* 1998;15(7):539-53.

[21]. Thomson Reuters. EndNote version X7 2016 [Available from: http://endnote.com/product-details/x7.

[22]. Shea BJ, Grimshaw JM, Wells GA, et al. Development of AMSTAR: a measurement tool to assess the methodological quality of systematic reviews. *BMC Med Res Methodol* 2007;7(1):1.

[23]. Sharif MO, Janjua-Sharif F, Ali H, et al. Systematic reviews explained: AMSTAR-how to tell the good from the bad and the ugly. *Oral Health Dent Manag* 2013;12(1):9-16.

[24]. Aromataris E, Fernandez R, Godfrey CM, et al. Summarizing systematic reviews: methodological development, conduct and reporting of an umbrella review approach. *Int J Evid Based Healthc* 2015;13(3):132-40.

[25]. Pieper D, Antoine S-L, Mathes T, et al. Systematic review finds overlapping reviews were not mentioned in every other overview. *J Clin Epidemiol* 2014;67(4):368-75.

[26]. Ryan RE, Weir M, Leslie B, et al. Evidence on Optimal Prescribing and Medicines Use for Decision Makers: Scope and Application of the Rx for Change Database. *J Pharm Pract* 2011;41(4):295-99.

For peer review only

[27]. Sapkota S, Brien JAE, Greenfield JR, et al. A systematic review of interventions addressing adherence to anti-diabetic medications in patients with type 2 diabetes. *PLoS ONE* 2015;10 (6): e0128581.

[28]. Walker RJ, Smalls BL, Bonilha HS, et al. Behavioral interventions to improve glycemic control in African Americans with type 2 diabetes: a systematic review. *Ethn Dis* 2013; 23(4):401-08.

[29]. Heinrich E, Schaper NC, de Vries NK. Self-management interventions for type 2 diabetes: a systematic review. *Eur Diabetes Nursing* 2010;7(2):71-76.

[30]. Alam R, Sturt J, Lall R, et al. An updated meta-analysis to assess the effectiveness of psychological interventions delivered by psychological specialists and generalist clinicians on glycaemic control and on psychological status. *Patient Educ Couns* 2009;75(1):25-36.

[31]. Aldcroft SA, Taylor NF, Blackstock FC, et al. Psychoeducational Rehabilitation for Health Behavior Change in Coronary Artery Disease: A systematic review of controlled trials. *J Cardiopulm Rehabil Prev* 2011;31(5):273-81.

[32]. Amaeshi IJ. Exploring the impact of structured foot health education on the rate of lower extremity amputation in adults with type 2 diabetes. A systematic review. *Podiatry Now* 2012;15(6):20-27.

[33]. Attridge M, Creamer J, Ramsden M, et al. Culturally appropriate health education for people in ethnic minority groups with type 2 diabetes mellitus. *Cochrane Database Syst Rev* 2014; 9:CD006424.

[34]. Auer R, Gaume J, Rodondi N, et al. Efficacy of in-hospital multidimensional interventions of secondary prevention after acute coronary syndrome a systematic review and meta-analysis. *Circulation* 2008;117(24):3109-17.

[35]. Barth J, Critchley J, Bengel J. Efficacy of psychosocial interventions for smoking cessation in patients with coronary heart disease: A systematic review and meta-analysis. *Ann Behav Med* 2006;32(1):10-20.

[36]. Barth J, Critchley JA, Bengel J. Psychosocial interventions for smoking cessation in patients with coronary heart disease. *Cochrane Libr* 2008

[37]. Brown JP, Clark AM, Dalal H, et al. Effect of patient education in the management of coronary heart disease: A systematic review and meta-analysis of randomized controlled trials. *Eur J Prev Cardiol* 2013;20(4):701-14.

[38]. Burke E, Robert, Ferrara A, et al. The effectiveness of group medical visits on diabetes mellitus type 2 (dm2) specific outcomes in adults: a systematic review, *JBI Libr Syst Rev*. 2011;9(23):833-885.

[39]. Chen L, Pei JH, Kuang J, et al. Effect of lifestyle intervention in patients with type 2

For peer review only

For peer review only - http://bmjopen.bmj.com/site/about/guidelines.xhtml

diabetes: A meta-analysis. *Metab. Clin. Exp.* 2015;64(2):338-47.

[40]. Choi TST, Davidson ZE, Walker KZ, et al. Diabetes education for Chinese adults with type 2 diabetes: A systematic review and meta-analysis of the effect on glycemic control. *Diabetes Res Clin Pract* 2016;116:218-29.

[41]. Clark AM, Hartling L, Vandermeer B, et al. Meta-analysis: secondary prevention programs for patients with coronary artery disease. *Ann Intern Med* 2005 Nov 1;143(9):659-72.

[42]. Creamer J, Attridge M, Ramsden M, et al. Culturally appropriate health education for Type 2 diabetes in ethnic minority groups: An updated Cochrane Review of randomized controlled trials. *Diabet Med* 2016;33(2):169-83.

[43]. Deakin T, McShane CE, Cade JE, et al. Group based training for self-management strategies in people with type 2 diabetes mellitus. *Cochrane Database Syst Rev* 2005;(2):CD003417.

[44]. Devi R, Singh Sally J, Powell J, et al. Internet-based interventions for the secondary prevention of coronary heart disease. *Cochrane Database Syst Rev* 2015, 22;(12):CD009386.

[45]. Dickens C, Cherrington A, Adeyemi I, et al. Characteristics of psychological interventions that improve depression in people with coronary heart disease: A systematic review and meta-regression. *Psychosom Med* 2013;75(2):211-21.

[46]. Duke SA, Colagiuri S, Colagiuri R. Individual patient education for people with type 2 diabetes mellitus. *Cochrane Database Syst Rev,* 2009;(1):CD005268.

[47]. Fan L, Sidani S. Effectiveness of diabetes self-management education intervention elements: a meta-analysis. *Can J Diabetes* 2009;33(1):18-26.

[48]. Fernandez R, Griffiths R, Everett B, et al. Effectiveness of brief structured interventions on risk factor modification for patients with coronary heart disease: a systematic review, Int *J Evid Based Healthc* 2007;5(4):370-405.

[49]. Gary TL, Genkinger JM, Guallar E, et al. Meta-analysis of randomized educational and behavioral interventions in type 2 diabetes. *Diabetes Educ 2003;29(3):488-501.*

[50]. Ghisi GLD, Abdallah F, Grace SL, et al. A systematic review of patient education in cardiac patients: Do they increase knowledge and promote health behavior change? *Patient Educ Couns* 2014;95(2):160-74.

[51]. Goulding L, Furze G, Birks Y. Randomized controlled trials of interventions to change maladaptive illness beliefs in people with coronary heart disease: systematic review. *J Adv Nurs* 2010;66(5):946-61.

[52]. Gucciardi E, Chan VWS, Manuel L, et al. A systematic literature review of diabetes

For peer review only - http://bmjopen.bmj.com/site/about/guidelines.xhtml

self-management education features to improve diabetes education in women of Black African/Caribbean and Hispanic/Latin American ethnicity. *Patient Educ Couns* 2013;92(2):235-45.

[53]. Hawthorne K, Robles Y, Cannings-John R, et al. Culturally appropriate health education for type 2 diabetes mellitus in ethnic minority groups. *Cochrane Database Syst Rev* 2008;(3):CD006424.

[54]. Hawthorne K, Robles Y, Cannings-John R, et al. Culturally appropriate health education for type 2 diabetes in ethnic minority groups: a systematic and narrative review of randomized controlled trials. *Diabet Med* 2010;27(6):613-23

[55]. Huang XL, Pan JH, Chen D, et al. Efficacy of lifestyle interventions in patients with type 2 diabetes: A systematic review and meta-analysis. *Eur J Intern Med* 2016;27:37-47.

[56]. Huttunen-Lenz M, Song F, Poland F. Are psychoeducational smoking cessation interventions for coronary heart disease patients effective? Meta-analysis of interventions. *Br J Health Psychol* 2010;15(Pt 4):749-77.

[57]. Kai Lun Gan J, Brammer JD, Creedy DK. Effectiveness of educational interventions to promote oral hypoglycaemic adherence in adults with Type 2 diabetes: a systematic review. *JBI Libr Syst Rev* 2011;9(9):269-312.

[58]. Khunti K, Camosso-Stefinovic J, Carey M, et al. Educational interventions for migrant South Asians with type 2 diabetes: a systematic review. *Diabet Med* 2008;25(8):985-92.

[59]. Kotb A, Hsieh S, Wells GA. The effect of telephone support interventions on coronary artery disease (CAD) patient outcomes during cardiac rehabilitation: a systematic review and meta-analysis. *PLoS One* 2014;9(5):e96581.

[60]. Loveman E, Frampton GK, Clegg AJ. The clinical effectiveness of diabetes education models for type 2 diabetes: a systematic review. *Health Technol Assess* 2008;12(9):1-116,

[61]. Minet L, Moller S, Vach W, et al. Mediating the effect of self-care management intervention in type 2 diabetes: a meta-analysis of 47 randomised controlled trials. *Patient Educ Couns* 2010;80(1):29-41.

[62]. Nam S, Janson SL, Stotts NA, et al. Effect of culturally tailored diabetes education in ethnic minorities with type 2 diabetes: a meta-analysis. *J Cardiovasc Nurs* 2012;27(6):505-18.

[63]. Nield L, Moore H, Hooper L, et al. Dietary advice for treatment of type 2 diabetes mellitus in adults. *Cochrane Database Syst Rev 2007;(3):CD004097.*

[64]. Norris SL, Engelgau MM, Narayan KM. Effectiveness of self management training in type 2 diabetes: a systematic review of randomized controlled trials. *Diabetes Care* 24(3):561-87.

For peer review only - http://bmjopen.bmj.com/site/about/guidelines.xhtml

[65]. Norris SL, Lau J, Smith SJ, et al. Self-management education for adults with type 2 diabetes: a meta-analysis of the effect on glycemic control. *Diabetes Care 2002;25(7):1159-71.*

[66]. Odnoletkova I, Goderis G, Pil L, et al. Cost-effectiveness of therapeutic education to prevent the development and progression of type 2 diabetes: Systematic review. *J Diabetes Metab 2014;5: 438*

[67]. Pal K, Eastwood SV, Michie S, et al. Computer-based interventions to improve self-management in adults with type 2 diabetes: a systematic review and meta-analysis. *Diabetes Care 2014;37(6):1759-66*

[68]. Pal K, Eastwood SV, Michie S, et al. Computer-based diabetes self-management interventions for adults with type 2 diabetes mellitus. *Cochrane Database Syst Rev* 2013;(3):CD008776

[69]. Barth J, Jacob T, Daha I, et al. Psychosocial interventions for smoking cessation in patients with coronary heart disease. *Cochrane Database Syst Rev* 2015;(7):CD006886.

[70]. Brown JP, Clark AM, Dalal H, et al. Patient education in the management of coronary heart disease. *Cochrane Database Syst Rev* 2011;(12):CD008895

[71]. Pillay J, Armstrong MJ, Butalia S, et al. Behavioral Programs for Type 2 Diabetes Mellitus: A Systematic Review and Network Meta-analysis. *Ann Intern Med* 2015;163(11):848-60.

[72]. Terranova CO, Brakenridge CL, Lawler SP, et al. Effectiveness of lifestyle-based weight loss interventions for adults with type 2 diabetes: a systematic review and meta-analysis. *Diabetes Obes Metab*2015;17(4):371-8.

[73]. Ricci-Cabello I, Ruiz-Pérez I, Rojas-García A, et al. Characteristics and effectiveness of diabetes self-management educational programs targeted to racial/ethnic minority groups: a systematic review, meta-analysis and meta-regression. *BMC Endocr Disord* 2014;14(1):60.

[74]. Saffari M, Ghanizadeh G, Koenig HG. Health education via mobile text messaging for glycemic control in adults with type 2 diabetes: a systematic review and meta-analysis. *Prim Care Diabetes 2014;8(4):275-85.*

[75]. Vugt M, Wit M, Cleijne WH, et al. Use of behavioral change techniques in web-based self-management programs for type 2 diabetes patients: systematic review. *J Med Internet Res* 2013; 15(12):e279.

[76]. Steinsbekk A, Rygg LO, Lisulo M, et al. Group based diabetes self-management education compared to routine treatment for people with type 2 diabetes mellitus. A systematic review with meta-analysis. *BMC Health Serv Res 2012;12:213.*

[77]. Ramadas A, Quek KF, Chan CK, et al. Web-based interventions for the management of

For peer review only - http://bmjopen.bmj.com/site/about/guidelines.xhtml

type 2 diabetes mellitus: a systematic review of recent evidence. *Int J Med Inform 2011*;80(6):389-405.

[78]. Wens J, Vermeire E, Hearnshaw H, et al. Educational interventions aiming at improving adherence to treatment recommendations in type 2 diabetes: A sub-analysis of a systematic review of randomised controlled trials. *Diabetes Res Clin Pract* 2008;79(3):377-88.

[79]. Zabaleta AM, Forbes A. Structured group-based education for type 2 diabetes in primary care. *Br J Community Nurs 2007;12(4):158-62.*

[80]. Vermeire E, Wens J, Van Royen P, et al. Interventions for improving adherence to treatment recommendations in people with type 2 diabetes mellitus. *Cochrane Database Syst Rev 2005;(2):CD003638.*

[81]. Albrecht L, Archibald M, Arseneau D, et al. Development of a checklist to assess the quality of reporting of knowledge translation interventions using the Workgroup for Intervention Development and Evaluation Research (WIDER) recommendations. *Implement Sci 2013;8:52.*

[82]. Glazier RH, Bajcar J, Kennie NR, et al. A systematic review of interventions to improve diabetes care in socially disadvantaged populations. *Diabetes Care* 2006;29(7):1675-88.

[83]. Powers MA, Bardsley J, Cypress M, et al. Diabetes Self-management Education and Support in Type 2 Diabetes: A Joint Position Statement of the American Diabetes Association, the American Association of Diabetes Educators, and the Academy of Nutrition and Dietetics. *Diabetes Care* 2015;38(7):1372-82

For peer review only

Table 1. Methodological quality assessment of included systematic reviews and meta-analyses

	Systematic Review/ Meta-analysis	Item 1	Item 2	Item 3	Item 4	Item 5	Item 6	Item 7	Item 8	Item 9	Item 10	Item 11	Total score
**Systematic reviews and meta-analysis involved ACS patients**													
1	Barth, Jacob, Daha, & Critchley, 2015 [69]	Yes	Yes	Yes	Yes	Yes	Yes	Yes	Yes	Yes	Yes	Yes	11
2	Devi et al., 2015 [44]	Yes	Yes	Yes	Yes	Yes	Yes	Yes	Yes	Yes	NA	Yes	10
3	Ghisi, Abdallah, Grace, Thomas, & Oh, 2014 [50]	CA	Yes	Yes	Yes	No	Yes	Yes	Yes	Yes	No	No	7
4	Kotb, Hsieh, & Wells, 2014 [59]	CA	Yes	Yes	Yes	No	Yes	Yes	Yes	Yes	No	Yes	8
5	Brown, Clark, Dalal, Welch, & Taylor, 2013 [37]	Yes	No	Yes	CA	No	Yes	Yes	Yes	Yes	NA	Yes	7
6	Dickens et al., 2013 [45]	CA	Yes	Yes	CA	No	Yes	Yes	Yes	Yes	Yes	Yes	8
7	Aldcroft, Taylor, Blackstock, & O'Halloran P, 2011 [31]	CA	No	Yes	CA	NO	Yes	Yes	Yes	Yes	No	Yes	6
8	Brown, Clark, Dalal, Welch, & Taylor, 2011 [70]	Yes	Yes	Yes	Yes	Yes	Yes	Yes	Yes	Yes	NA	Yes	10
9	Huttunen-Lenz, Song, & Poland, 2010 [56]	CA	No	Yes	CA	No	Yes	Yes	Yes	Yes	No	No	5
10	Goulding, Furze, & Birks, 2010 [51]	Yes	Yes	Yes	CA	No	Yes	Yes	Yes	Yes	No	Yes	8
11	Auer, Gaume, Rodondi, Cornuz, & Ghali, 2008 [34]	CA	Yes	Yes	CA	No	No	Yes	No	Yes	Yes	No	5
12	J. Barth, Critchley, & Bengel, 2008 [36]	Yes	Yes	Yes	No	Yes	Yes	Yes	Yes	Yes	Yes	Yes	10
13	Fernandez et al., 2007 [48]	Yes	Yes	Yes	Yes	Yes	Yes	Yes	Yes	Yes	No	No	8
14	J. Barth, Critchley, & Bengel, 2006 [35]	CA	Yes	Yes	CA	No	Yes	CA	Yes	Yes	Yes	Yes	7

For peer review only - http://bmjopen.bmj.com/site/about/guidelines.xhtml

15	Clark, Hartling, Vandermeer, & McAlister, 2005 [41]	CA	Yes	Yes	CA	No	Yes	Yes	Yes	Yes	Yes	Yes	8
**Systematic reviews and meta-analysis involved T2DM patients**													
16	Choi, Davidson, Walker, Lee, & Palermo, 2016 [40]	CA	Yes	Yes	No	No	Yes	Yes	Yes	Yes	Yes	Yes	8
17	Creamer, Attridge, Ramsden, Cannings-John, & Hawthorne, 2016 [42]	Yes	Yes	Yes	CA	No	Yes	Yes	Yes	Yes	No	Yes	8
18	Huang et al., 2016 [55]	CA	CA	Yes	CA	No	Yes	Yes	Yes	Yes	Yes	Yes	7
19	Chen et al., 2015 [39]	CA	CA	Yes	CA	No	Yes	Yes	Yes	Yes	Yes	Yes	7
20	Pillay et al., 2015 [71]	Yes	No	Yes	Yes	No	Yes	Yes	Yes	Yes	Yes	Yes	9
21	Terranova, Brakenridge, Lawler, Eakin, & Reeves, 2015 [72]	CA	CA	Yes	No	Yes	Yes	Yes	Yes	Yes	Yes	Yes	8
22	Attridge, Creamer, Ramsden, Cannings-John, & Hawthorne, 2014 [33]	Yes	Yes	Yes	Yes	Yes	Yes	Yes	Yes	Yes	No	Yes	10
23	Odnoletkova et al., 2014 [66]	Yes	CA	Yes	CA	No	No	Yes	Yes	Yes	Yes	No	6
24	Pal et al., 2014 [67]	CA	Yes	Yes	Yes	No	Yes	Yes	Yes	Yes	No	Yes	8
25	Ricci-Cabello et al., 2014 [73]	Yes	CA	Yes	Yes	No	Yes	Yes	Yes	Yes	Yes	Yes	9
26	Saffari, Ghanizadeh, & Koenig, 2014 [74]	CA	Yes	Yes	CA	No	Yes	Yes	Yes	Yes	Yes	Yes	8
27	Gucciardi, Chan, Manuel, & Sidani, 2013 [52]	CA	Yes	Yes	No	No	Yes	Yes	Yes	Yes	No	Yes	7
28	Pal et al., 2013 [68]	Yes	Yes	Yes	Yes	Yes	Yes	Yes	Yes	Yes	No	Yes	10
29	Vugt, Wit, Cleijne, & Snoek, 2013 [75]	CA	Yes	Yes	CA	No	Yes	Yes	Yes	NA	No	Yes	6
30	Amaeshi, 2012 [32]	CA	CA	Yes	No	No	Yes	Yes	Yes	NA	No	No	4
31	Nam, Janson, Stotts, Chesla, &	CA	CA	Yes	Yes	No	Yes	Yes	Yes	Yes	Yes	Yes	8

For peer review only - http://bmjopen.bmj.com/site/about/guidelines.xhtml

	Kroon, 2012 [62]												
32	Steinsbekk, Rygg, Lisulo, Rise, & Fretheim, 2012 [76]	CA	Yes	Yes	CA	No	Yes	Yes	Yes	Yes	No	Yes	7
33	Burke et al., 2011 [38]	Yes	Yes	Yes	Yes	Yes	Yes	Yes	Yes	Yes	NA	Yes	10
34	Kai Lun Gan, Brammer, & Creedy, 2011 [57]	Yes	Yes	Yes	CA	No	Yes	Yes	Yes	Yes	No	Yes	8
35	Ramadas, Quek, Chan, & Oldenburg, 2011 [77]	CA	CA	Yes	No	No	Yes	Yes	Yes	NA	No	Yes	5
36	Hawthorne, Robles, Cannings-John, & Edwards, 2010 [54]	Yes	Yes	Yes	CA	No	Yes	Yes	Yes	Yes	CA	Yes	8
37	Minet, Moller, Vach, Wagner, & Henriksen, 2010 [61]	Yes	Yes	Yes	No	No	Yes	Yes	Yes	Yes	Yes	Yes	9
38	Alam, Sturt, Lall, & Winkley, 2009 [30]	Yes	Yes	No	CA	No	Yes	Yes	Yes	Yes	Yes	Yes	8
39	Duke, Colagiuri, & Colagiuri, 2009 [46]	Yes	CA	Yes	No	Yes	Yes	Yes	Yes	Yes	No	Yes	8
40	Fan & Sidani, 2009 [47]	Yes	No	Yes	CA	No	Yes	No	No	Yes	No	Yes	5
41	Hawthorne, Robles, Cannings-John, & Edwards, 2008 [53]	Yes	Yes	Yes	Yes	Yes	Yes	Yes	Yes	Yes	Yes	Yes	11
42	Khunti, Camosso-Stefinovic, Carey, Davies, & Stone, 2008 [58]	CA	Yes	Yes	Yes	No	Yes	No	No	No	No	Yes	5
43	Loveman, Frampton, & Clegg, 2008 [60]	Yes	CA	Yes	Yes	No	Yes	Yes	Yes	Yes	No	Yes	8
44	Wens et al., 2008 [78]	CA	Yes	Yes	CA	No	Yes	Yes	Yes	Yes	NA	Yes	7
45	Nield et al., 2007 [63]	Yes	Yes	Yes	CA	Yes	Yes	Yes	Yes	Yes	No	Yes	9
46	Zabaleta & Forbes, 2007 [79]	CA	CA	Yes	CA	Yes	Yes	Yes	Yes	NA	No	No	5
47	Deakin, McShane, Cade, &	Yes	Yes	Yes	Yes	Yes	Yes	Yes	Yes	Yes	Yes	Yes	11

For peer review only

	Williams, 2005 [43]											
**48**	Vermeire et al., 2005 [80]	Yes	Yes	Yes	CA	Yes	Yes	Yes	Yes	Yes	No	Yes	9
**49**	Gary, Genkinger, Guallar, Peyrot, & Brancati, 2003 [49]	CA	Yes	No	Yes	No	Yes	Yes	No	Yes	No	Yes	6
**50**	Norris, Lau, Smith, Schmid, & Engelgau, 2002 [65]	CA	No	Yes	No	No	Yes	Yes	Yes	CA	No	No	4
**51**	Norris, Engelgau, & Narayan, 2001 [64]	CA	Yes	Yes	CA	No	Yes	Yes	Yes	NA	No	No	5

Item 1:"Was an 'a priori' design provided?"; **Item 2**: "Was there duplicate study selection and data extraction?"; **Item 3**: "Was a comprehensive literature search performed?"; **Item 4**: "Was the status of publication (ie. grey literature) used as an inclusion criterion?"; **Item 5**: "Was a list of studies (included and excluded) provided?"; **Item 6**: "Were the characteristics of the included studies provided?"; **Item 7**: "Was the scientific quality of the included studies assessed and documented?"; **Item 8**: "Was the scientific quality of the included studies used appropriately in formulating conclusions?"; **Item 9**: "Were the methods used to combine the findings of studies appropriate?"; **Item 10**: "Was the likelihood of publication bias assessed?"; **Item 11**: "Was the conflict of interest stated?"

CA= Cannot Answer; NA= Not Application;

Source: *Shea, B. J., Grimshaw, J. M., Wells, G. A., Boers, M., Andersson, N., Hamel, C., ... & Bouter, L. M. (2007). Development of AMSTAR: a measurement tool to assess the methodological quality of systematic reviews. BMC Medical Research Methodology, 7(1), 1.*

For peer review only - http://bmjopen.bmj.com/site/about/guidelines.xhtml

Table 2. Characteristics and interventions of included systematic reviews and meta-analysis involved ACS patients

(First author, Year); Journal	Primary objectives (To assess effect of intervention s on….)	Studies details	Intervention						Outcomes (Primary outcomes were bold.)<br>"–" : No change<br>"↑" : Increase<br>"↓" : decrease	Synthesis methods
			Educational Content		Provider	Number of session(s), delivery mode, time, setting				
(Devi et al., 2015) [44]; The Cochrane Library	Lifestyle changes and medicines manageme nt	**Number of studies**:11 completed trials (12 publications);<br>**Types of studies:** RCTs;<br>**Total sample:** 1,392 participants.	All Internet based interventions.	√ BEHA (-)<br>√ CVR (-)<br>√ DIET (-)<br>√ EXERCISE (-)<br>☐ MED<br>√ PSY(-)<br>√ SMOKING (-)<br>☐ SELF	Dietitians; exercise specialists; nurse practitioners; physiotherapist rehabilitation specialists, or did not describe.	**Number of session:** Weekly or monthly or unclear;<br>**Total contact hours:** Unclear.<br>**Duration:** From six weeks to one year.	**Strategies:** Internet- and mobile phone-based intervention, such as email access, private-messaging function on the website, one-to-one chat facility, a synchronised group chat, an online discussion forum, or telephone consultations; or video files;<br>**Format:** one-on-one chat sessions; "ask an expert" group chat sessions;<br>**Theoretical approach:** Unclear.	Inpatient settings, post discharge, other	**– Clinical outcomes;**<br>**– Cardiovascular risk factors;**<br>– Lifestyle changes;<br>– Compliance with medication;<br>– Healthcare utilization and costs;<br>↓ Adverse intervention effects.	Meta-analysis used Review Manager software.
(Barth, Jacob, Daha, & Critchley, 2015) [69]; The Cochrane Library	Smoking cessation	**Number of studies**:40 RCTs;<br>**Types of studies:** RCTs;<br>**Total sample:** 7,928 participants.	Psychosocial smoking cessation interventions	☐ BEHA<br>☐ CVR<br>☐ DIET<br>☐ EXERCISE<br>☐ MED<br>☐ PSY<br>√ SMOKING<br>☐ SELF	Cardiologist; general practitioner physician or study nurse.	**Number of session**: Weekly or 2-3 times per week;<br>**Total contact hours:** Unclear.<br>**Duration:** From 8 weeks to one year.	**Strategies:** Face to face, telephone contact, written educational materials, videotape, booklet or unclear;<br>**Format:** One by one counseling; telephone call; group meetings or unclear;<br>**Theoretical approach:** TTM, SCT.	Inpatient settings, post discharge, other	**↑ Abstinence by self-report or validated.**	Meta-analysis used Review Manager software.
(Kotb, Hsieh, & Wells, 2014) [59]; PloS one	Patients' outcomes.	**Number of studies**: 26 studies;<br>**Types of studies:** RCTs;<br>**Total sample:** 4,081	Telephone-delivered post-discharge interventions.	☐ BEHA<br>√ CVR<br>☐ DIET<br>☐ EXERCISE<br>☐ MED	Dietitians; exercise specialist; health educators; nurses and pharmacists.	**Number of session:** 3-6 sessions/telephone calls and was greater than 6 calls in five	**Strategies:** Telephone calls;<br>**Format:** Unclear, did not describe the format;<br>**Theoretical approach:** Unclear.	Unclear, did not describe the setting.	**↓ All-cause hospitalization;**<br>**– All-cause mortality;**<br>↓ Depression;	Meta-analysis used Review Manager software.

For peer review only

For peer review only - http://bmjopen.bmj.com/site/about/guidelines.xhtml

		participants.		☐ PSY<br>☐ SMOKING<br>☐ SELF		studies; or unclear;<br>**Total contact hours:** 40 mins to 180 mins or unclear;<br>**Duration:** 1.5 - 6 months or unclear.			– Anxiety;<br>↑ Smoking cessation,<br>↓ Systolic blood pressure;<br>– LDL-c.	
(Ghisi, Abdallah, Grace, Thomas, & Oh, 2014) [50]; Patient Education and Counseling	knowledge, health behavior change, medication adherence, psychosocial well-being.	**Number of studies**: 42 articles;<br>**Types of studies:** Thirty were experimental: 23 RCTs and 7 quasi-experimental; and 11 observational and 1 used a mixed-methods design.<br>**Total sample:** 16,079 participants.	Any educational interventions.	√ BEHA (+)<br>√ CVR (++)<br>√ DIET (+++)<br>√ EXERCISE (++)<br>√ MED (++)<br>√ PSY(++)<br>√ SMOKING (+)<br>☐ SELF	Nurses (35.7%), a multidisciplinary team (31%), dietitians (14.3%), and a cardiologist (2.4%).	**Number of session:** 1-24 or unclear.<br>**Total contact hours:** 5–10 min to 3 h as well as a full day of education.<br>**Duration:** 1-24 month; from daily education to every 6 months.	**Strategies:** Did not describe the strategies;<br>**Format:** Group (88.1%) education was delivered by lectures (40.5%), group discussions (40.5%), and question and answer periods (7.1%). Individual education (88.1%), including individual counseling (50%), follow-up telephone contacts (31%) and home visits (7.1%);<br>**Theoretical approach:** Unclear.	Inpatient settings	**– Knowledge,**<br>**– Behavior;**<br>**– Psychosocial indicators.**	Narrative synthesis
(Brown, Clark, Dalal, Welch, & Taylor, 2013) [37]; European Journal of Preventive Cardiology	Mortality, morbidity, HRQoL and healthcare costs.	**Number of studies:** Twenty-four papers reporting on 13 RCTs;<br>**Types of studies:** RCTs;<br>**Total sample:** 68,556 participants.	Patient education.	☐ BEHA<br>√ CVR<br>☐ DIET<br>☐ EXERCISE<br>☐ MED<br>☐ PSY<br>☐ SMOKING<br>☐ SELF	Nurses or other healthcare professionals.	**Number of session and Duration:** From a total of 2 visits to a 4-week residential stay reinforced with 11 months of nurse led follow-up.<br>**Total contact hours:** Unclear.	**Strategies:** Face-to-face education sessions, telephone contact and interactive use of the Internet;<br>**Format:** Group-based sessions, individualized education and four utilized a mixture of both sessions;<br>**Theoretical approach:** Unclear.	Inpatient settings, other	**– Mortality,**<br>**– Non-fatal MI,**<br>**– Revascularizations,**<br>**– Hospitalizations,**<br>**– HRQoL,**<br>– Withdrawals/drop outs;<br>– Healthcare utilization and costs.	Meta-analysis used Review Manager software.
(Dickens et al., 2013) [45]; Psychosomatic Medicine	Depression and depressive symptoms.	**Number of studies:** Sixty-two independent studies.<br>**Types of studies:** RCTs;<br>**Total sample:** 17,397.	Psychological interventions.	√ BEHA (-)<br>☐ CVR<br>☐ DIET<br>☐ EXERCISE<br>☐ MED<br>√ PSY (-)<br>☐ SMOKING	A single health professional or by a unidisciplinary team	**Number of session:** 14.4 (range, 1-156);<br>**Total contact hours:** Varying from 10 to 240 minutes.<br>**Duration:** Unclear.	**Strategies:** Face-to-face sessions, telephone contact or unclear;<br>**Format:** Group or unclear;<br>**Theoretical approach:** Unclear.	Unclear, did not describe.	↓ **Depression;**<br>– Adverse cardiac outcomes;<br>– Ongoing cardiac symptoms.	Univariate analyses using Comprehensive Meta-Analysis, multivariate

For peer review only

				√ SELF (-)						meta-regression using SPSS 15.0
(Aldcroft, Taylor, Blackstock, & O'Halloran P, 2011) [20]; Journal of Cardiopulmonary Rehabilitation & Prevention	Health behavior change.	**Number of studies:** 7 trials. **Types of studies:** Six randomized controlled trials and a quasi-experimental trial. **Total sample:** 536 participants.	All psychoeducational or behavioral intervention.	☐ BEHA<br>√ CVR (-)<br>☐ DIET<br>☐ EXERCISE<br>☐ MED<br>√ PSY (-)<br>☐ SMOKING<br>☐ SELF	Appropriately trained health care workers.	**Number of session:** Unclear; **Total contact hours:** Unclear; **Duration:** 2-12 months.	**Strategies:** Did not describe the strategies; **Format:** Group setting, combination of group and one-on-one education and one-on-one format only; **Theoretical approach:** TTM, interactionist role theory, Bandura's self-efficacy theory, Gordon's relapse prevention model and a cognitive behavioral approach.	Unclear, did not describe.	↓ **Smoking rates; medication use;**<br>− **Supplemental oxygen use;**<br>↑ **Physical activity;**<br>↑ Nutritional habits.	Meta-analysis and narrative presentation
(Brown, Clark, Dalal, Welch, & Taylor, 2011) [70]; The Cochrane Library	Mortality, morbidity, HRQofL and healthcare costs.	**Number of studies:** 24 papers reporting on 13 studies. **Types of studies:** RCTs; **Total sample:** 68,556 participants.	Patient education.	√ BEHA (-)<br>√ CVR (-)<br>☐ DIET<br>√ EXERCISE (-)<br>√ MED<br>☐ PSY<br>☐ SMOKING<br>☐ SELF	Nurse or did not describe.	**Number of session and Duration:** two 2 visits to 4 week residential 11 months of nurse led folluw up **Total contact hours:** Unclear.	**Strategies:** face-to-face sessions, telephone contact and interactive use of the Internet; **Format:** Four studies involved group sessions, five involved individualized education and three utilized both session types, with one study comparing the two approaches; **Theoretical approach:** did not describe.	Post discharge, other	− **Total mortality;**<br>− **Cardiovascular**<br>− **mortality;**<br>− **Non-cardiovascular mortality;**<br>− **Total cardiovascular (CV) events;**<br>− **Fatal and/or non-fatal MI;**<br>− Other fatal and/or non-fatal CV events	Meta-analysis used Review Manager software.
(Goulding, Furze, & Birks, 2010) [51]; Journal of Advanced Nursing	Change maladaptive illness.	**Number of studies:** **13 studies;** **Types of studies:** RCTs; **Total sample:** Unclear.	Interventions to change maladaptive illness beliefs	√ BEHA (-)<br>☐ CVR<br>☐ DIET<br>☐ EXERCISE<br>☐ MED<br>√ PSY (-)<br>☐ SMOKING	Cardiologist, nurse, psychologist or did not describe.	**Number of session:** Unclear; **Total contact hours:** Unclear; **Duration:** 4 days to two weeks or unclear.	**Strategies:** Face-to-face sessions, telephone contact and written self-administered; **Format:** Unclear; **Theoretical approach:** Common Sense Model, Leventhal's framework.	Inpatient settings, post discharge, other	− **Beliefs (or other illness cognition);**<br>− QoL;<br>− Behaviour;<br>− Anxiety or depression;<br>− Psychological	A descriptive data synthesis.

For peer review only

				☐ SELF					wellbeing;<br>− Modifiable risk factors; protective factors.	
(Huttunen-Lenz, Song, & Poland, 2010) [56]; British Journal of Health Psychology	Smoking cessation.	**Number of studies:** A total of 14 studies were included.<br>**Types of studies:** RCTs;<br>**Total sample:** 1,792 participants.	Psychoeducational cardiac rehabilitation intervention.	☐ BEHA<br>☐ CVR<br>☐ DIET<br>☐ EXERCISE<br>☐ MED<br>☐ PSY<br>√ SMOKING (-)<br>☐ SELF	Cardiologist, nurse psychologist, or did not describe.	**Number of session:** 4-20 or unclear.<br>**Total contact hours:** 10-720 mins or unclear.<br>**Duration:** 4-29 weeks or unclear.	**Strategies:** Face-to-face counselling, self-help materials; home visit, booklet, video and telephone contact.<br>**Format:** individual or unclear.<br>**Theoretical approach:** Social learning theory; ASE model; TTM; Behavioural multicomponent approach.	Inpatient settings, post discharge, other	↑ **Prevalent smoking cessation,**<br>↑ **Continuous smoking cessation,**<br>− Mortality.	Subgroup meta-analysis was used software.
(Auer, Gaume, Rodondi, Cornuz, & Ghali, 2008) [34]; Circulation	Multiple cardiovascular risk factors and all-cause mortality.	**Number of studies:** 27 articles reporting 26 studies.<br>**Types of studies:** 16 clinical controlled trials and 10 before-after studies.<br>**Total sample:** 2,467 patients in CCTs and 38, 581 patients in before-after studies.	In-Hospital Multidimensional Interventions of Secondary Prevention	☐ BEHA<br>☐ CVR<br>√ DIET (-)<br>√ EXERCISE (-)<br>√ MED<br>√ PSY (-)<br>√ SMOKING (-)<br>☐ SELF	Cardiac nurses; physician, or did not describe.	**Number of session:**1-5 or unclear;<br>**Total contact hours:** 30-240mins or unclear;<br>**Duration:** 4 weeks – 12 months.	**Strategies:** Written material; audiotapes; presentations; face-to-face;<br>**Format:** Group or unclear;<br>**Theoretical approach:** Unclear.	Inpatient settings.	↓ **All-cause mortality;**<br>↓ **Readmission rates;**<br>− **Reinfarction rates.**	Stata version 9.1.
(Barth, Critchley, & Bengel, 2008) [36] ; The Cochrane Library	Smoking cessation.	**Number of studies:** Forty trials;<br>**Types of studies:** RCTs;<br>**Total sample:** 7,682 patients.	Psychosocial intervention	√ BEHA (+++)<br>√ CVR (++)<br>☐ DIET<br>☐ EXERCISE<br>☐ MED<br>√ PSY (+)<br>√ SMOKING (+++)	Cardiologist, nurse, physician or study nurse.	**Number of session:** 1-5 or unclear;<br>**Total contact hours:** 15 mins - 9 hours.<br>**Duration:** within 4 weeks or did not report on the duration.	**Strategies:** Face to face; information booklets, audio- or videotapes.<br>**Format:** Group sessions or individual counselling;<br>**Theoretical approach:** TTM.	Inpatient settings	↑ **Abstinence by self-report or validated.**	Meta-analysis used Review Manager software.

For peer review only

				√ SELF(+++)						
(Fernandez et al., 2007) [48]; International Journal of Evidence-Based Healthcare	Risk factor modification.	**Number of studies:** Seventeen trials;<br>**Types of studies:** randomised, quasi-RCTs and clustered trials;<br>**Total sample:** 4,725 participants.	Brief structured intervention.	√ BEHA (-)<br>√ CVR (-)<br>☐ DIET<br>☐ EXERCISE<br>☐ MED<br>☐ PSY<br>☐ SMOKING<br>√ SELF (-)	Case manager; dieticians; health educator; nurses; psychologist; and research assistants.	**Number of session:** supportive counselling ranged from 1 to 7 calls for the duration of the study;<br>**Total contact hours:** varied from 10 to 30 mins;<br>**Duration:** Unclear.	**Strategies:** Written, visual, audio, telephone contact;<br>**Format:** did not describe;<br>**Theoretical approach:** theoretical behaviour change principles.	Unclear, did not describe.	↓ **Smoking;**<br>**− Cholesterol level;**<br>**− Physical activity;**<br>↑ **Dietary habits;**<br>↓ **Blood sugar levels;**<br>**− BP levels;**<br>↓ **BMI;**<br>− Incidence of admission.	Cochrane statistical package Review Manager.
(J. Barth, Critchley, & Bengel, 2006) [35]; Annals of Behavioral Medicine	Smoking cessation.	**Number of studies:** Nineteen trials;<br>**Types of studies:** RCTs;<br>**Total sample:** 2,548 patients.	Psychosocial interventions.	√ BEHA (+++)<br>√ CVR (++)<br>☐ DIET<br>☐ EXERCISE<br>☐ MED<br>☐ PSY<br>☐ SMOKING<br>√ SELF (+++)	Unclear, did not describe.	**Number of session:** Unclear;<br>**Total contact hours:** Unclear;<br>**Duration:** Unclear.	**Strategies:** Face to face, telephone contact or unclear;<br>**Format:** Unclear;<br>**Theoretical approach:** Unclear.	Unclear, did not describe.	↑ **Abstinence;**<br>↓ **Smoking status**.	Data analyses were carried out in Review Manager 4.2.
(Clark, Hartling, Vandermeer, & McAlister, 2005) [41]; Annals of Internal Medicine	Mortality, MI	**Number of studies:** 63 randomized trials;<br>**Types of studies:** RCTs;<br>**Total sample:** 21,295 patients.	Secondary prevention programs	☐ BEHA<br>☐ CVR<br>√ DIET (-)<br>√ EXERCISE (-)<br>☐ MED<br>√ PSY (-)<br>☐ SMOKING<br>☐ SELF	Nurse, multidisciplinary team, or did not describe.	**Number of session:** 1-12 or unclear;<br>**Total contact hours:** did not describe.<br>**Duration:** 0.75-48 months.	**Strategies:** Face to face, telephone contact and home visit;<br>**Format:** Group and individual or unclear;<br>**Theoretical approach:** Unclear.	Inpatient settings, post discharge, other	↓ **Mortality,**<br>↓ **MI,**<br>− Hospitalization rates.	Performed analyses by using Review Manager 4.2 and Qualitative Data Synthesis

NOTE:

For peer review only

SMOKING= Smoking cessation; CVR= Cardiovascular risk factors; PSY =Psychosocial issues (depression, anxiety); DIET =Diet; EXERCISE= Exercise; MED= Medication; BEHA = Behavioral charge (including lifestyle modification); SELF = Self-management (including problems solving); GC= glycemic control; DR =Diabetes risks; CHD= Coronary heart disease; CAD = Coronary Artery Disease; CHW=Community Health Worker; HbA1c = Glycatedhemoglobin; BP =Blood Pressure; LDL = Low-density Lipoprotein Cholesterol; SMS = Short Message Service; BCTs = Behavioral Change Techniques; LEA = Lower Extremity Amputation; PRIDE = Problem Identification, Researching one's routine, Identifying a management goal, Developing a plan to reach it, Expressing one's reactions and Establishing rewards for making progress; ASE = Attitude-social influence-efficacy; CVRF= Cardiovascular Risk Factors; SMS = Short Message System; PA = Physical Activity; EDU = Patient Education; GP= General Practice; RCTs =Randomized controlled trials; CCTS = controlled clinical trials; HRQoL = Health Related Quality of Life; QoL = Quality of Life; MI = Myocardial Infarction; CAD = Coronary Artery Disease; CABG= Coronary Artery Bypass Graft Surgery; BMI = Body Mass Index; HbA1c = Glycatedhemoglobin; BP =Blood Pressure; SBP = Systolic Blood Pressure, DBP = Diastolic Blood Pressure, HDL-c= High-density Lipoprotein Cholesterol; LDL-c = Low-density Lipoprotein Cholesterol; TTM= Transtheoretical Model; SCT=Social Cognitive Theory; HBM =Health Belief Model; SAT=Social Action Theory;

In the educational content: "+": minor focus; "++":moderate focus; "+++" major focus; "-" =unclear what the intensity of the education was for any topic;

In the outcomes: arrow up (" ↑ ") for improvement, arrow down (" ↓ ") for reduction; a dash ("−") for no change or inconclusive evidence. Primary outcomes were bold.

For peer review only

Table 3. Characteristics and interventions of included systematic reviews and meta-analysis involved T2DM patients

(First author, Year); Journal	Primary objectives (To assess effect of interventions on….)	Studies details	Intervention						Outcomes (Primary outcomes were bold.) "−" : No change "$\uparrow$" : Increase "$\downarrow$" : decrease	Synthesis methods
			Educational Content		Provider	Number of session(s), delivery mode, time, setting				
(Choi, Davidson, Walker, Lee, & Palermo, 2016) [40]; Diabetes Research and Clinical Practice	Glycemic effect	**Number of studies:** 53 studies(five in English, 48 in Chinese);<br>**Types of studies:** RCTs;<br>**Total sample:** Unclear.	Diabetes education intervention.	☐ BEHA<br>√ DIET (-)<br>☐ DR<br>☐ EXERCISE<br>☐ GC<br>☐ MED<br>☐ PSY<br>☐ SMOKING<br>√ SELF (-)	Unclear, did not describe.	**Number of session:** Unclear;<br>**Total contact hours:** Unclear;<br>**Duration:** 30–150 min or unclear.	**Strategies:** Face to face, written materials; telephone contact and home visit;<br>**Format:** Unclear;<br>**Theoretical approach:** Unclear.	Inpatient settings, post discharge, other	$\downarrow$ **HbA1c (glycated haemoglobin).**	STATAversion 12 and Review Manager version 5.3
(Creamer, Attridge, Ramsden, Cannings-John, & Hawthorne, 2016) [42]; Diabetic Medicine	successful outcomes and to suggest directions for future research.	**Number of studies: 33;**<br>**Types of studies:** RCTs;<br>**Total sample:** 7,453 participants.	Culturally appropriate health education.	√ BEHA (-)<br>√ DIET (-)<br>√ DR (-)<br>√ EXERCISE<br>☐ GC<br>☐ MED<br>☐ PSY<br>☐ SMOKING<br>√ SELF (-)	CHWs, clinical pharmacists dieticians, Nurses, podiatrists, physiotherapists, and psychologists.	**Number of session:** one to ten or unclear;<br>**Total contact hours:** unclear;<br>**Duration:** from a single session to 24 months.	**Strategies:** Face-to-face; Phone contact;<br>**Format:** group sessions (10 studies), individual sessions (13) or a combination of both;<br>**Theoretical approach:** Unclear.	Inpatient settings, post discharge, other	$\downarrow$ **HbA1c,**<br>**− HRQoL,**<br>**− Adverse events,**<br>− BP,<br>− BMI,<br>− Lipid levels,<br>− diabetes complications,<br>− Economic analyses,<br>− Mortality and diabetes knowledge,<br>− Empowerment,<br>− Self-efficacy and satisfaction.	Meta-analysis using the Review Manager statistical program.

For peer review only

(Huang et al., 2016) [55]; European Journal of Internal Medicine	Clinical markers of cardiovascular disease	**Number of studies:** 17 studies;<br>**Types of studies:** RCTs;<br>**Total sample:** Unclear.	Lifestyle interventions.	☐ BEHA<br>√ DIET (-)<br>√ CVR (-)<br>√ EXERCISE (-)<br>☐ GC<br>☐ MED<br>☐ PSY<br>☐ SMOKING<br>√ SELF (-)	Nurse, Pharmacist or unclear.	**Number of session:** Unclear;<br>**Total contact hours:** Unclear;<br>**Duration:** 6 month – 8 years.	**Strategies:** Unclear;<br>**Format:** Individual; Group and Mixed.<br>**Theoretical approach:** Unclear;	Unclear, did not describe.	**Cardiovascular risk factors** (such as,<br>− BMI,<br>↓ HbA1c,<br>− BP,<br>↓ The level of cholesterol.)	Review Manager 5.1.
(Chen et al., 2015) [39]; Metabolism-Clinical and Experimental	Clinical markers.	**Number of studies:** 16 studies;<br>**Types of studies:** RCTs;<br>**Total sample:** per study ranged from 23 to 2575;	Lifestyle intervention.	√ BEHA (-)<br>☐ DIET<br>√ CVR (-)<br>☐ EXERCISE<br>☐ GC<br>√ MED (-)<br>☐ PSY<br>☐ SMOKING<br>√ SELF (-)	Unclear, did not describe.	**Number of session:** Monthly;<br>**Total contact hours:** Unclear;<br>**Duration:** < 6 months -8 years.	**Strategies:** Unclear;<br>**Format:** Individual; Group and Mixed;<br>**Theoretical approach:** Unclear.	Unclear, did not describe.	**Cardiovascular risk factors** including<br>↓ BMI,<br>↓ HbA1c,<br>↓ SBP, DBP,<br>− HDL-c, and LDL-c.	All analyses were performed using Comprehensive Meta-Analysis statistical software.
(Terranova, Brakenridge, Lawler, Eakin, & Reeves, 2015) [72]; Diabetes, Obesity and Metabolism	Weight loss.	**Number of studies:** 10 individual studies (from 13 papers);<br>**Types of studies:** RCTs;<br>**Total sample:** ranging from 27 to 5145 participants.	Lifestyle-based-only intervention.	√ BEHA (-)<br>√ DIET (-)<br>☐ DR (-)<br>√ EXERCISE (-)<br>☐ GC<br>☐ MED<br>☐ PSY<br>☐ SMOKING<br>√ SELF (-)	Dietician; diabetes educator; general physician; multidisciplinary team; or nutritionist; nurse.	**Number of session:** 1-42;<br>**Total contact hours:** Unclear;<br>**Duration:** ranged from 16 weeks to 9 years.	**Strategies and Format:** Face-to-face individual or group-based sessions, or a combination of those. One study delivered the intervention via the telephone<br>**Theoretical approach:** Unclear.	Unclear, did not describe.	↓ **Weight change;**<br>− **HbA1c.**	Meta-analyses - Review Manager and meta-regression analysis - stata version
(Pillay et al., 2015) [71]; Annals of	HbAlc level.	**Number of studies:** 132;<br>**Types of studies:**	Behavioral Programs.	√ BEHA (-)<br>√ DIET (-)<br>☐ DR	Trained individuals.	**Number of session:** Unclear;<br>**Total contact hours:**	**Strategies:** Unclear;<br>**Format:** Unclear;<br>**Theoretical approach:** Unclear.	Inpatient settings, post discharge,	− **HbA1c;**<br>↓ **BMI.**	The analysis was conducted

For peer review only

internal medicine		RCTs; **Total sample:** Unclear.		√ EXERCISE (-)<br>√ GC (-)<br>√ MED (-)<br>☐ PSY<br>☐ SMOKING<br>√ SELF (-)		range, [illegible] to 40.5 hours; **Duration:** 4 or more weeks.		other		by using a Bayesian network model.
(Pal et al., 2014) [67]; Diabetes Care	Health status, cardiovascular risk factors, and QoL.	**Number of studies:** 20 papers describing 16 studies; **Types of studies:** RCTs; **Total sample:** 3,578 participants.	Computer-based self-management interventions.	☐ BEHA<br>☐ DIET<br>☐ DR<br>☐ EXERCISE<br>☐ GC<br>☐ MED<br>☐ PSY<br>☐ SMOKING<br>√ SELF	Unclear, did not describe.	**Number of session:** 1-8 ; **Total contact hours:** 10 mins - 6 hours; **Duration:** 8 weeks – 12 months;	**Strategies:** Online/web-based; Phone contact. **Format:** Individual; Group and Mixed. **Theoretical approach:** TTM, social ecological theory, SCT, and self-determination theory.	Unclear, did not describe.	**− HRQoL,**<br>**↓ HbA1c,**<br>**− Death;**<br>↓ Cognitions, behaviors,<br>− Social support,<br>↓ Cardiovascular risk factors,<br>− Complications,<br>− Emotional outcomes,<br>− Hypoglycemia,<br>− Adverse effects,<br>− CE and ecfonomic data.	Meta-analysis using Review Manager software or narrative presentatio.
(Ricci-Cabello et al., 2014) [73]; BMC endocrine disorders	Knowledge, behaviors and clinical outcomes.	**Number of studies:** Thirty-seven studies; **Types of studies:** Almost two thirds of the studies were RCTs, 27% studies were Quasi-experimental design. **Total sample:** Unclear.	DSM educational programs.	☐ BEHA<br>√ DIET(+++)<br>☐ DR<br>√ EXERCISE (+++)<br>√ GC(+++)<br>√ MED(++)<br>√ PSY(++)<br>☐ SMOKING<br>☐ SELF	Dietitian; Nurse; Psychologist; Physician; Research team or staff;	**Number of session:** 13.1; **Total contact hours:** 0.25-180 hours; **Duration:** 0.25-48 months.	**Strategies:** Face-to-face; Telecommunication; Both. **Format:** One on one; Group and Mixed. **Theoretical approach:** Unclear.	Post discharge, other	**− Diabetes knowledge;**<br>**− Self-management;**<br>**− Behaviors;**<br>**− Clinical outcomes;**<br>↓ Glycated hemoglobin;<br>− Cost-effectiveness analysis.	Meta-analyses and bivariate meta-regression were conducted with Stata, version 12.0.

For peer review only

(Saffari, Ghanizadeh, & Koenig, 2014) [74]; Primary Care Diabetes	Glycemic control.	**Number of studies:** 10;<br>**Types of studies:** RCTs;<br>**Total sample:** 960 patients.	An educational intervention using SMS.	√ BEHA (-)<br>☐ DIET<br>☐ DR<br>☐ EXERCISE<br>√ GC (-)<br>√ MED (-)<br>☐ PSY<br>☐ SMOKING<br>☐ SELF	Unclear, did not describe.	**Number of session:** Weekly; or 2 messages daily or unclear;<br>**Total contact hours:** Unclear.<br>**Duration:** 3 months – 1 year	**Strategies:** SMS: sending and receiving data. Receive data through text-messaging by patients only. Used a website along with SMS;<br>**Format:** Unclear;<br>**Theoretical approach:** Unclear.	Inpatient settings, post discharge, other	↑ **Glycemic control**.	Comprehensive Meta-analysis Software Version 2.0.
(Odnoletkova et al., 2014) [66]; Journal of Diabetes & Metabolism	Cost-effectiveness (CE).	**Number of studies:** Seventeen studies;<br>**Types of studies:** RCTs;<br>**Total sample:** Unclear.	Therapeutic education.	√ BEHA (-)<br>☐ DIET<br>☐ DR<br>☐ EXERCISE<br>☐ GC<br>☐ MED<br>☐ PSY<br>☐ SMOKING<br>√ SELF (-)	General physician; nutritionists or unclear.	**Number of session:** ~ 16;<br>**Total contact hours:** Unclear;<br>**Duration:** Unclear.	**Strategies:** Face-to-face or unclear;<br>**Format:** individual and group lessons;<br>**Theoretical approach:** Unclear.	Inhospital or unclear.	− **CE.**	Incremental Cost-Effectiveness Ratio.
(Attridge, Creamer, Ramsden, Cannings-John, & Hawthorne, 2014) [33]; The Cochrane Library	HbAlc level, Knowledge and clinical outcomes.	**Number of studies:** 33 trials;<br>**Types of studies:** RCTs and quasi-RCTs;<br>**Total sample:** 7453 participants.	'Culturally appropriate' health education.	√ BEHA (-)<br>√ DIET (-)<br>☐ DR<br>√ EXERCISE (-)<br>√ GC (-)<br>☐ MED<br>☐ PSY<br>√ SMOKING (-)<br>☐ SELF	CHWs; dieticians; exercise physiologists; lay workers; nurses; podiatrists; and psychologists;.	**Number of session:** one session to 24 months;<br>**Total contact hours:** Unclear;<br>**Duration:** The median duration of interventions was six months.	**Strategies:**<br>**Format:** Group intervention method, one-to-one sessions and a mixture of the two methods. Or a purely interactive patient-centred method.<br>**Theoretical approach:** empowerment theories; behaviour change theories, TTM of behaviour change and SCT.	Inpatient settings, post discharge, other	↓ **HbA1c;**<br>− **HRQoL;**<br>− **Adverse events;**<br>− Mortality;<br>− Complications;<br>−Satisfaction; ↑ Empowerment;<br>↑ Self-efficacy;<br>− Attitude; knowledge;	Meta-analyses used Review Manager software.

For peer review only

									−BP;<br>−BMI;<br>↓ Lipid levels;<br>−Health economics.	
(Vugt, Wit, Cleijne, & Snoek, 2013)[75]; Journal of Medical Internet Research	Health outcomes.	**Number of studies:** 13 studies;<br>**Types of studies:** RCTs;<br>**Total sample:** 3813 patients.	BCTs are being used in online self-management interventions.	√ BEHA (-)<br>☐ DIET<br>☐ DR<br>☐ EXERCISE<br>☐ GC<br>☐ MED<br>☐ PSY<br>☐ SMOKING<br>√ SELF (-)	Health care professional.	**Number of session:** 6 weekly sessions or unclear;<br>**Total contact hours:** Unclear;<br>**Duration:** Unclear.	**Strategies:** Online/web-based;<br>**Format:** Unclear;<br>**Theoretical approach:** self-efficacy theory, social support theory, TTM, SCT, social-ecological model, and cognitive behavioral therapy.	Post discharge	**− Health behavior change,**<br>**− Psychological well-being;**<br>− Clinical parameters.	Unclear.
(Gucciardi, Chan, Manuel, & Sidani, 2013)[52]; Patient Education and Counseling	HbAlc level,physical activity and diet outcomes.	**Number of studies:** Thirteen studies;<br>**Types of studies:** RCTs and comparative studies;<br>**Total sample:** Unclear.	DSME interventions.	☐ BEHA<br>√ DIET (+++);<br>☐ DR<br>√ EXERCISE (+++);<br>☐ GC<br>√ MED (+);<br>√ PSY (+)<br>☐ SMOKING<br>√ SELF (++).	Dietitians (n = 7/13); Multidisciplinary Team (n = 7/13); Nurse (n = 5/13); Community Peer Worker (n = 3/13).	**Number of session:** Low Intensity: <10 education sessions (n = 7); High Intensity: ≥ 10 education sessions (n = 6);<br>**Total contact hours:** Unclear;<br>**Duration:** < 6 months (n = 7/13); ⩾6months (n = 6/13).	**Strategies:** Face-to-face(n = 13/13); Written Literature: (e.g., handbook) (n = 4/13);Telephone(n = 4/13);Audio-Visual(n = 1/13);<br>**Format:** One-on-one: (n = 11/13);Group (n = 9/13);<br>**Theoretical approach:** SAT; Empowerment Behavior Change Model; modification theories; Pharmaceutical Care Model; Behavior Change Theory; PATHWAYS Program; Symptom-Focused Management Model; Motivational interviewing.	Inpatient settings, post discharge	**− HbA1c levels,**<br>− Anthropometrics,<br>− Physical activity;<br>− Diet outcomes.	Arecently described method.
(Pal et al., 2013) [68]; The Cochrane Library	Health status and HRQoL.	**Number of studies:** 16 studies;<br>**Types of studies:** RCTs;<br>**Total sample:** 3,578	Computer-based diabetes self-management intervention.	☐ BEHA<br>√ DIET (-)<br>☐ DR<br>√ EXERCISE (-)<br>√ GC (-)	Nurse or other healthcare professionals.	**Number of session:** Unclear;<br>**Total contact hours:** Unclear;<br>**Duration:** One session –	**Strategies:** Online/web-based; phone contact<br>**Format:** Unclear;<br>**Theoretical approach:** Unclear.	Inpatient settings, post discharge, other	**− HRQoL;**<br>**− Death from any cause;**<br>**↓ HbA1c;**	Formal meta-analyses and narrative

For peer review only

For peer review only

		participants.		√ MED (-)<br>√ PSY (-)<br>☐ SMOKING<br>☐ SELF		1[illegible] months.			− Cognitions;<br>− Behaviours;<br>−Social support;<br>−Biological markers;<br>− Complications.	synthesis.
(Nam, Janson, Stotts, Chesla, & Kroon, 2012) [62]; Journal of Cardiovascular Nursing	Glycemic control.	**Number of studies:** 12 RCTs;<br>**Types of studies:** RCTs;<br>**Total sample:** 1,495 participants.	Diabetes educational interventions (no drug intervention).	☐ BEHA<br>√ DIET (-)<br>☐ DR<br>√ EXERCISE (-)<br>√ GC (-)<br>√ MED (-)<br>√ PSY (-)<br>☐ SMOKING<br>√ SELF (-)	Nurses (36%), dieticians (36%), diabetes educators (5%), other professionals (9%) and nonprofessional staff (14%).	**Number of session:** 1 month or less; 1 to 3 months; and 12 months;<br>**Total contact hours:** most studies did not describe, or from 1 session to more than 30 hours;<br>**Duration:** from 1 session to 12 months, frequency: 1 session to 25 weekly or biweekly education.	**Strategies:** Teaching or counseling; home-based support; and visual aids.<br>**Format:** group education or a combination of group education and individual counseling; or only individual counseling;<br>**Theoretical approach:** Unclear.	Inpatient settings, post discharge, other	↓ **HbA1c level.**	Meta-analysis.
(Steinsbekk, Rygg, Lisulo, Rise, & Fretheim, 2012) [76]; BMC Health Services Research	Clinical, lifestyle and psychosocial outcomes.	**Number of studies:** 21 studies (26 publications).<br>**Types of studies:** RCTs;<br>**Total sample:** 2,833 participants.	Group-based education.	Did not describe the content of the intervention.	Community workers; dietician; lay health advisors nurse and nutritionist.	**Number of session and Total contact hours:** 30 hours over 2.5 months, 52 hours over one year,and 36 hours or 96 hours over 6 months.<br>**Duration:** Six months to two years.	**Strategies:** Face to face;<br>**Format:** 5 to 8 participants group to 40 patients group.<br>**Theoretical approach:** empowerment model and the discovery learning theory, the SCT and the social ecological theory, the self-efficacy and self-management theories and operant reinforcement theory.	Inpatient settings, post discharge, other	↓ **HbA1c,**<br>↑ **Lifestyle outcomes,**<br>↑ **Diabetes knowledge,**<br>↑ Self-management skills,<br>↑ Psychosocial outcomes,<br>↓ Mortality rate,<br>↓ BMI,<br>↓ Blood pressure;<br>↓ Lipid profile.	Meta-analysis using Review manager v5.
(Amaeshi,	Increasing	**Number of studies:**	Foot health	Food care.	Podiatrist,	**Number of session:**	**Strategies:** Face to face;	Unclear, did	↓ **LEA;**	Narrative

2012) [32]; Podiatry Now	good foot health practices that will ultimately reduce LEA.	8 studies; **Types of studies:** RCT or clinical controlled trial (CCT); **Total sample:** Unclear.	education:		psychotogist or unclear.	Unclear; **Total contact hours:** between 15min and 14 hours; **Duration:** 3-30 months.	**Format:** In three of the studies, educational interventions were delivered to the participants in groups, while the other five provided individualized (one-to-one) foot-care education to the participants; **Theoretical approach:** Unclear.	not describe.	↑ Self-care.	synthesis.
Kai Lun Gan, Brammer, & Creedy, 2011) [57]; JBI Library of Systematic Reviews	Oral hypoglycaemic adherence.	**Number of studies:** Seven studies; **Types of studies:** RCTs; **Total sample:** Unclear.	Educational interventions.	√ BEHA (-)<br>√ DIET (-)<br>☐ DR<br>√ EXERCISE (-)<br>√ GC (-)<br>√ MED (-)<br>√ PSY (-)<br>☐ SMOKING<br>√ SELF (-)	Nurses; pharmacists; other skilled healthcare professionals.	**Number of session:** 1-12 or unclear; **Total contact hours:** 2.5 hours or unclear; **Duration:** 4-12 months.	**Strategies:** Face to face; **Format:** Group and individual; **Theoretical approach:** Unclear.	Inpatient settings, post discharge, other	↓ **HbA1c,**<br>− **Medication adherence;**<br>↓ Blood glucose;<br>− Tablet count;<br>− Medication containers;<br>− Diabetes complications;<br>− Health service utilization.	Narrative summary form.
Burke et al., 2011) [38]; JBI Database of Systematic Reviews and Implementation Reports	HbAlc level,BP.	**Number of studies:** 11 RCTs and 4 quasi-experimental trials; **Types of studies:** RCTs and quasi-experimental trials; **Total sample:** 2,240 patients.	Group medical visits.	√ BEHA (-)<br>√ DIET (-)<br>☐ DR<br>☐ EXERCISE<br>√ GC (-)<br>√ MED (-)<br>☐ PSY<br>☐ SMOKING<br>√ SELF (-)	Dndocrinologists; DM nurse; family physician; nutritionist and rehab therapist.	**Number of session:** 1-4 or unclear; **Total contact hours:** 2-4 hours or unclear; **Duration:** One session to 2 years.	**Strategies:** Face to face; **Format:** Group and individual; **Theoretical approach:** Unclear.	Inpatient settings, post discharge, other	↓ **HbA1c;**<br>− **Systolic and diastolic BP;**<br>− LDL measurements.	Meta-analysis.
(Ramadas, Quek, Chan, & Oldenburg, 2011) [77];	HbAlc level.	**Number of studies:** 13 different studies; **Types of studies:** RCTs and quasi-experimental studies;	Web-based behavioural interventions.	√ BEHA (-)<br>√ DIET (-)<br>☐ DR<br>☐ EXERCISE	Dietician; endocrinologist; physicians; researchers or research staff	**Number of session:** Unclear; **Total contact hours:** Unclear; **Duration:** ranged	**Strategies:** E-mail and SMS technologies that were commonly used together with the websites to reinforce the intervention, and Website, print material.	Inpatient settings, post discharge, other	− **Self-monitoring blood sugar,**<br>− Weight-loss,<br>− Dietary behaviour,	Not statistically combined and re-analyzed;

International Journal of Medical Informatics		**Total sample:** Unclear.		√ GC (-)<br>√ MED (-)<br>☐ PSY<br>☐ SMOKING<br>√ SELF (-)	members and study nurse.	between 12 and 52 weeks, with an average of 27.2 ± 18.3 weeks.	**Format:** Unclear;<br>**Theoretical approach:** Wagner's Chronic Care Model; Self-efficacy theory/Social support theory; TTM; HBM; SCT.		− Physical activity.	
(Minet, Moller, Vach, Wagner, & Henriksen, 2010) [61]; Patient Education and Counseling	Glycaemic control.	**Number of studies:** Forty-seven studies;<br>**Types of studies:** RCTs;<br>**Total sample:** Unclear.	Self-care management interventions.	√ BEHA (-)<br>☐ DIET<br>☐ DR<br>☐ EXERCISE<br>☐ GC<br>☐ MED<br>☐ PSY<br>☐ SMOKING<br>√ SELF (-)	Case nurse manager; group facilitator; nurse educator; multidisciplinary team; physiologist; physician; peer counsellor; researcher; and pharmacist.	**Number of session:** 3-26;<br>**Total contact hours:** Unclear;<br>**Duration:** 4 weeks to 4 years.	**Strategies:** Face to face; home visit; phone calls;<br>**Format:** Group and individual;<br>**Theoretical approach:** Unclear.	Inpatient settings, post discharge, other	↓ **HbA1c.**	Meta-analyses and meta-regression used Stata's meta command.
(Hawthorne, Robles, Cannings-John, & Edwards, 2010) [54]; Diabetic Medicine	Effects of culturally appropriate health education	**Number of studies:** 10 trials;<br>**Types of studies:** RCTs;<br>**Total sample:** 1603 patients.	Culturally appropriate health education	☐ BEHA<br>√ DIET (-)<br>☐ DR<br>√ EXERCISE (-)<br>☐ GC<br>☐ MED<br>☐ PSY<br>☐ SMOKING<br>√ SELF (-)	Exercise physiologists; dieticians; diabetes nurses; link workers, and podiatrists.	**Number of session:** Unclear;<br>**Total contact hours:** Unclear;<br>**Duration:** one session to 12 months;	**Strategies:** Face to face; visual aids, leaflets and teaching materials;<br>**Format:** group approach, one-to-one interviews and a mixed approach;<br>**Theoretical approach:** SAT, Empowerment Behaviour Change Model, SCT, Management model and the Theory of Planned Behaviour.	Inpatient settings, post discharge, other	**−QoL;**<br>↓ **HbA1c;**<br>**− BP;**<br>↑ Knowledge;<br>− BMI;<br>↓ Lipid levels,<br>− Diabetic complications,<br>− Mortality rates, hospital admissions, hypoglycaemia.	Meta - analysis using the Review Manager and narrative review.
(Fan & Sidani, 2009) [47]; Canadian	Knowledge, self-manageme nt	**Number of studies:** **50 studies;**<br>**Types of studies:** RCTs;	DSME intervention:	√ BEHA (-)<br>☐ DIET<br>☐ DR<br>☐ EXERCISE	Unclear, did not describe.	**Number of session:** 10 (range 1 to 28);<br>**Total contact hours:** 17 contact hours (range 1 to	**Strategies:** Online/web-based (4%); video (2%); face-to-face (60%); phone contact (4%); Mixed (30%).<br>**Format:** One-on-one (32%); Group	Inpatient settings, post discharge, other	↑ **Diabetes knowledge,**<br>↑ **Self-management behaviours;**	Comprehensive Meta-Analysis (version

For peer review only

Journal of Diabetes	behaviours and metabolic control.	**Total sample:** Unclear.		☐ GC<br>☐ MED<br>√ PSY (-)<br>☐ SMOKING<br>√ SELF (-)		52); ≤ 10 (46%); 11-20 (21%); > 20 (33%);<br>**Duration:** 22 weeks (range 1 to 48); ≤ 8 weeks (26%); 9–24 weeks (37%); > 24 weeks (37%).	(40%); Mixed (28%).<br>**Theoretical approach:** Unclear.		↓ **HbA1c,**	2.0).
(Duke, Colagiuri, & Colagiuri, 2009) [46]; The Cochrane Library	Metabolic control, diabetes knowledge and psychosocial outcomes.	**Number of studies:** Nine studies;<br>**Types of studies:** RCTs;<br>**Total sample:** 1,359 participants.	Individual patient education.	√ BEHA (-)<br>☐ DIET<br>☐ DR<br>√ EXERCISE (-)<br>√ GC (-)<br>☐ MED<br>√ PSY (-)<br>☐ SMOKING<br>☐ SELF	Diabetes educators and dieticians.	**Number of session:** 1-6;<br>**Total contact hours:** 20 min – 7 hours;<br>**Duration:** 4 weeks – 1 year.	**Strategies:** Face to face; telephone;<br>**Format:** Individual;<br>**Theoretical approach:** Unclear.	Inpatient settings	− **HbA1c;**<br>− **Diabetes complications;**<br>− Health service utilisation and health care costs;<br>− Psychosocial outcomes;<br>− Diabetes knowledge; patient self-care behaviours;<br>− Physical measures; metabolic.	Meta-analysis.
(Alam, Sturt, Lall, & Winkley, 2009) [30]; Patient Education and Counseling	Glycaemic control and psychological status.	**Number of studies:** 35 trials;<br>**Types of studies:** RCTs;<br>**Total sample:** 1431 patients.	Psycho-educational Interventions.	√ BEHA (-)<br>☐ DIET<br>☐ DR<br>☐ EXERCISE<br>☐ GC<br>☐ MED<br>√ PSY (-)<br>☐ SMOKING<br>☐ SELF	Generalists; psychological specialists; or did not report the specialist	**Number of session:** 1-16;<br>**Total contact hours:** 20 min – 28 hours;<br>**Duration:** about 13.7 (±11.06) weeks.	**Strategies:** Face to face; telephone calls;<br>**Format:** group format; a single format and used a combination;<br>**Theoretical approach:** TTM; motivational interviewing;	Inpatient settings, other	↓ **HbAlc;**<br>↓ **Psychological distress.**	Meta-analysis.
(Khunti, Camosso-	Knowledge and	**Number of studies:** nine studies;	Any educational	☐ BEHA<br>√ DIET<br>☐	Unclear, did not describe.	**Number of session:** Unclear;	**Strategies:** Face to face;<br>**Format:** Group and individual;	Unclear, did not describe.	− **Knowledge;**<br>− **Psychological and**	Unclear.

Ste[illegible]ovic,<br>Carey,<br>Davies, &<br>Stone, 2008)<br>[58]; Diabetic<br>Medicine	biomedical<br>outcomes.	**Types of studies:**<br>RCTs and RCT was<br>followed by a before-<br>and-after study;<br>**Total sample:** 1004<br>patients.	intervention.	DR<br>☐ EXERCISE<br>☐ GC<br>☐ MED<br>☐ PSY<br>☐ SMOKING<br>☐ SELF		**Total contact hours:**<br>Unclear;<br>**Duration:** 3- 12 months.	**Theoretical approach:** Unclear.		**biomedical outcome**<br>**measures.**	
(Loveman,<br>Frampton, &<br>Clegg, 2008)<br>[60]; Health<br>Technology<br>Assessment	Clinical<br>effectivenes<br>s.	**Number of studies:**<br>Twenty-one published<br>trials;<br>**Types of studies:**<br>RCTs and CCTs;<br>**Total sample:**<br>Unclear.	Educational<br>interventions.	√ BEHA (++)<br>√ DIET (+++)<br>☐ DR<br>√ EXERCISE<br>(+++)<br>√ GC (+++)<br>☐ MED<br>☐ PSY<br>☐ SMOKING<br>√ SELF (+++)	Community<br>workers;<br>diabetes<br>research<br>technician;<br>diabetes nurse,<br>dieticians;<br>educationalist;<br>medical<br>students;<br>nurses;<br>pharmacists;<br>physician, or<br>physician<br>assistant.	**Number of session:** Two<br>to four intensive<br>education of 1.5 to 2<br>hours followed up with<br>additional education at, 3<br>and 6 months;<br>**Total contact hours and**<br>**Duration:** about 150 mins<br>over a 6-month or 61 to<br>52 hours over one year.	**Strategies:** Face to face;<br>**Format:** Group and individual;<br>**Theoretical approach:** cognitive-<br>behavioural strategies; pedagogical<br>principle.	Inpatient<br>settings, post<br>discharge,<br>other	**− Diabetic control**<br>**outcomes**;<br>− Diabetic end-points;<br>− QoL and cognitive<br>measures.	Narrative<br>review
(Wens et al.,<br>2008) [78];<br>Diabetes<br>Research<br>and Clinical<br>Practice	Improving<br>adherence<br>to medical<br>treatment<br>recommend<br>ations.	**Number of studies:**<br>8 studies;<br>**Types of studies:**<br>RCTs and controlling<br>before and after<br>studies.<br>**Total sample:** 772<br>patients.	Interventions<br>aimed at<br>improving<br>adherence to<br>medical<br>treatment.	√ BEHA (-)<br>√ DIET (-)<br>☐ DR<br>√ EXERCISE (-)<br>√ GC (-)<br>√ MED (-)<br>☐ PSY<br>☐ SMOKING<br>√ SELF (-)	Diabetes<br>educator; nurse<br>or did not<br>describe.	**Number of session:**<br>Unclear;<br>**Total contact hours:**<br>Unclear;<br>**Duration:** ～9 months or<br>unclear.	**Strategies:** Face-to-face; telephone;<br>**Format:** face-to-face; group based<br>and telemedicine;<br>**Theoretical approach:** Unclear.	Inpatient<br>settings, post<br>discharge,<br>other	**− Adherence;**<br>− HbA1c;<br>− Blood glucose.	Cochrane<br>Review<br>Manager<br>software.

For peer review only

(Hawthorne, Robles, Cannings-John, & Edwards, 2008) [53]; The Cochrane Library	HbAlc level, Knowledge and clinical outcomes.	**Number of studies:** A total of 11 trials;<br>**Types of studies:** RCTs;<br>**Total sample:** 1,603 patients.	Culturally appropriate (or adapted) health education.	√ BEHA (-)<br>√ DIET (-)<br>☐ DR<br>√ EXERCISE (-)<br>√ GC (-)<br>☐ MED<br>☐ PSY<br>√ SMOKING (-)<br>☐ SELF	Dieticians, diabetes nurses, exercise physiologists; link workers; podiatrists; psychologist and and non-professional link worker.	**Number of session:** Unclear;<br>**Total contact hours:** Unclear;<br>**Duration:** one session to 12 months.	**Strategies:** Face-to-face; booklet;<br>**Format:** Group intervention method; one to one interviews; mixture of the two methods; purely interactive patient-centred method; semi-structured didactic format and combination of the 2 approaches.<br>**Theoretical approach:** SAT; Empowerment Behaviour Change Model; Behaviour Change Theory; SCT, Management Model and the Theory of Planned Behaviour.	Inpatient settings, post discharge, other	↓ **HbA1c**<br>↑ **Knowledge scores**<br>− Other outcome measures	Narrative presentation and meta-analysis.
(Nield et al., 2007) [63]; The Cochrane Library	Metablic control.	**Number of studies:** 36 articles (18 trials);<br>**Types of studies:** RCTs;<br>**Total sample:** 1,467 participants.	Dietary advice.	☐ BEHA<br>√ DIET<br>☐ DR<br>☐ EXERCISE<br>☐ GC<br>☐ MED<br>☐ PSY<br>☐ SMOKING<br>☐ SELF	exercise physiologist; dietitian; group facilitator; nutritionist; nurse educator; and physician.	**Number of session:** 1-12;<br>**Total contact hours:** 20 min-22 hours;<br>**Duration:** 11 weeks – 6months or unclear.	**Strategies:** Face-to-face;<br>**Format:** Group and individual;<br>**Theoretical approach:** Unclear.	Inpatient settings, post discharge, other	− **Weight;**<br>− **Diabetic complications;**<br>− **HbA1c;**<br>− QoL;<br>− Medication use;<br>− Cardiovascular disease risk;	Meta-analysis.
(Zabaleta & Forbes, 2007) [79]; British Journal of Community Nursing	Clinical effectivenes s.	**Number of studies:** 21 studies;<br>**Types of studies:** Controlled trials;<br>**Total sample:** Unclear.	Structured group diabetes education.	√ BEHA (-)<br>√ DIET (-)<br>☐ DR<br>√ EXERCISE (-)<br>√ GC (-)<br>☐ MED<br>√ PSY (-)<br>☐ SMOKING<br>☐ SELF	Diabetes nurse educator; physician's assistant; and physicians;	**Number of session:** 4-6 or unclear;<br>**Total contact hours:** 6-12 hours or unclear;<br>**Duration:** 1-6 months or unclear.	**Strategies:** Face to face;<br>**Format:** Group;<br>**Theoretical approach:** Unclear.	Post discharge.	−**HbA1c.**	A tabulative synthesis.

For peer review only

(Deakin, McShane, Cade, & Williams, 2005) [43]; The Cochrane Library	Clinical, lifestyle and psychosocial outcomes.	**Number of studies:** 14 publications, reporting 11 studies;<br>**Types of studies:** RCTs, and CCTs;<br>**Total sample:** 1,532 participants.	Group-based educational programmes.	Did not describe the content of the intervention.	Health professionals, lay health advisors.	**Number of session:** Unclear;<br>**Total contact hours:** from 6 to 52 hours;<br>**Duration:** 3 hours per year for 2 years and 3 or 4 hours per year for 4 years.	**Strategies:** Unclear;<br>**Format:** Group;<br>**Theoretical approach:** the Diabetes Treatment and Teaching Programme (DTTP); empowerment model; adult learning model, public health model, HBM and TTM.	Inpatient settings, post discharge	**↓ Metabolic control;**<br>**↑ Diabetes knowledge;**<br>↑ QoL;<br>↑ Empowerment/self-efficacy.	Summarized statistically
(Vermeire et al., 2005) [80]; The Cochrane Library	Improving adherence to treatment recommendations.	**Number of studies:** 21 articles;<br>**Types of studies:** RCTs; cross-over study; controlled trial; controlled before and after studies;<br>**Total sample:** 4,135 patients.	Interventions that were aimed at improving the adherence to treatment recommendations.	☐ BEHA<br>☐ DIET<br>☐ DR<br>☐ EXERCISE<br>√ GC (-)<br>√ MED (-)<br>☐ PSY<br>☐ SMOKING<br>☐ SELF	Nurse, pharmacist and other healthcare professionals.	**Number of session:** Unclear;<br>**Total contact hours:** Unclear;<br>**Duration:** Unclear.	**Strategies:** Face to face; telephone; home visit; video; mailed educational materials;<br>**Format:** Unclear.<br>**Theoretical approach:** Unclear.	Inpatient settings, post discharge	**Direct indicators,** such as<br>↓ Blood glucose level;<br>− Indirect indicators, such as pill counts;<br>−Health outcomes.	A descriptive review and sub-group meta-analysis.
(Gary, Genkinger, Guallar, Peyrot, & Brancati, 2003) [49]; Diabetes Educator	Body weight and glycaemic control.	**Number of studies:** Sixty-three RCTs;<br>**Types of studies:** RCTs;<br>**Total sample:** 2,720 patients.	Educational and behavioural component interventions.	☐ BEHA<br>√ DIET (-)<br>☐ DR<br>√ EXERCISE (-)<br>√ GC (-)<br>√ MED (-)<br>☐ PSY<br>☐ SMOKING<br>☐ SELF	Nurse (39%); dietitian (26%); physician (17%); other or not specified (23%); other professional (13%); psychologist (9%); Exercise psychologist (9%); and health educator (4%).	**Number of session:** Unclear;<br>**Total contact hours:** Unclear.<br>**Duration:** 1 month to 19.2 months.	**Strategies:** Unclear;<br>**Format:** Unclear;<br>**Theoretical approach:** SAT, contracting model and patient empowerment.	Inpatient settings, post discharge	**− Glycaemic control;**<br>**− Weight.**	Sufficient data were combined using meta-analysis.
(Norris, Lau, Smith,	Total GHb.	**Number of studies:** 31 studies	Self-management	√ BEHA (-)<br>√ DIET (-)	Dietitian; lay health care	**Number of session:** 6 (1–36);	**Strategies:** Online/web-based; video ; face-to-face; phone contact;	Inpatient settings, post	**↓ Total GHb,**	Meta-analysis and

For peer review only

Schmid, & Engelgau, 2002) [65]; Diabetes Care		**Types of studies:** RCTs. **Total sample:** 4,263 patients.	education.	☐DR ☐EXERCISE ☐GC ☐MED ☐PSY ☐SMOKING √ SELF (-)	worker; nurse; physician with team; self (e.g. computer assisted instruction); and team (nurse, dietitian, etc.).	**Total contact hours:** 9.2 (1–28) hours; **Duration:** 6 (1.0–27) months.	**Format: Group;** individual and mixed; **Theoretical approach:** Unclear	discharge, other		meta-regression.
(Norris, Engelgau, & Narayan, 2001) [64]; Diabetes Care	Clinical outcomes,Knowledge, Metablic control.	**Number of studies:** 72 studies (84 papers); **Types of studies:** RCTs; **Total sample:** Unclear.	Self-management training interventions.	√ BEHA (-) √ DIET (-) ☐DR ☐EXERCISE ☐GC ☐MED ☐PSY ☐SMOKING √ SELF (-)	CHWs; nurse; or other healthcare professionals.	**Number of session:** 1-16; **Total contact hours:** ～22 hours; **Duration:** ～26 months.	**Strategies:** Online/web-based; video (2%); face-to-face; phone contact; **Format:** Group; individual and mixed; **Theoretical approach:** SAT; Fishbein and Ajzen HBM.	Inpatient settings, post discharge, other	↑ **Knowledge;** ↑ **Lifestyle behaviors;** **−Psychological and QoL outcomes;** ↑ **Glycemic control;** − Cardiovascular disease risk factors.	Outcomes are summarized in a qualitative fashion.

NOTE:

SMOKING= Smoking cessation; CVR= Cardiovascular risk factors; PSY =Psychosocial issues (depression, anxiety); DIET =Diet; EXERCISE= Exercise; MED= Medication; BEHA = Behavioral charge (including lifestyle modification); SELF = Self-management (including problems solving); GC= glycemic control; DR =Diabetes risks; CHD= Coronary heart disease; CAD = Coronary Artery Disease; CHW=Community Health Worker; HbA1c = Glycatedhemoglobin; BP =Blood Pressure; LDL = Low-density Lipoprotein Cholesterol; SMS = Short Message Service; BCTs = Behavioral Change Techniques; LEA = Lower Extremity Amputation; PRIDE = Problem Identification, Researching one's routine, Identifying a management goal, Developing a plan to reach it, Expressing one's reactions and Establishing rewards for making progress; ASE = Attitude-social influence-efficacy; CVRF= Cardiovascular Risk Factors; SMS = Short Message System; PA = Physical Activity; EDU = Patient Education; GP= General Practice; RCTs =Randomized controlled trials; CCTS = controlled clinical trials; HRQoL = Health Related Quality of Life; QoL = Quality of Life; MI = Myocardial Infarction; CAD = Coronary Artery Disease; CABG= Coronary Artery Bypass Graft Surgery; T2DM = Type 2 Diabetes Mellitus; BMI = Body Mass Index; HbA1c = Glycatedhemoglobin; BP =Blood Pressure; SBP = Systolic Blood Pressure, DBP = Diastolic Blood Pressure, HDL-c= High-density Lipoprotein Cholesterol; LDL-c = Low-density Lipoprotein Cholesterol; DSME = Diabetes Self-management Education; DSM = Diabetes Self-management; TTM= Transtheoretical Model; SCT=Social Cognitive Theory; HBM =Health Belief Model; SAT=Social Action Theory;

In the educational content: "+": minor focus; "++":moderate focus; "+++" major focus; "-" =unclear what the intensity of the education was for any topic;

In the outcomes: arrow up (" ↑ ") for improvement, arrow down (" ↓ ") for reduction; a dash ("−") for no change or inconclusive evidence.

For peer review only

Table 4. Summary of evidence from quantitative research syntheses

Intervention	Number of systematic reviews/meta-analysis, Total participants	First author, Year	Primary Results/findings		Rating the evidence of effectiveness
**Acute Coronary Syndrome Patients**					
General health education	Six/161,997patients (Goulding et al., 2010 did not given the total sample size)	Ghisi et al., 2014 [50]	Knowledge	91% studies (+)	Some evidence
			Behavior	77%/84%/65% studies (+)	
			Psychosocial indicators	43% studies (+)	
		Brown et al., , 2013 [37]	Mortality		
			MI		
			Revascularizations		
			Hospitalizations		
			HRQoL		
			Withdrawals/drop outs		
			Healthcare utilization and costs		
		Brown et al., 2011 [70]	Total mortality		
			MI		
			CABG		
			Hospitalisations		
			HRQoL	63.6% studies (+)	
			Healthcare costs	40% studies (+)	
			Withdrawal/drop out		
		Goulding et al., 2010 [51]	Beliefs	30.08% studies (+)	
			Secondary outcomes		
			Smoking		
			Cholesterol level		

For peer review only

		Fernandez et al., 2007 [48]	Multiple risk factor modification		
		Kotb, Hsieh, & Wells, 2014 [59]	All-cause hospitalization		
			All-cause mortality		
			Smoking cessation		
			Depression		
			Systolic blood pressure		
			Low-density lipoprotein		
			Anxiety		
Psychoeducational interventions	Six/ 37,883 patients	Barth et al., 2015 [69]	Abstinence by self-report or validated		Sufficient evidence
		Dickens et al., 2013 [45]	Depression		
		Aldcroft et al., 2011 [31]	Smoking cessation		
			Physical activity		
		Huttunen-Lenz et al.,2010 [56]	Prevalent smoking cessation		
			Continuous smoking cessation		
			Total mortality		
		Barth et al., 2008 [36]	Abstinence by self-report or validated		
			Smoking status		
		Barth et al., 2006 [35]	Abstinence		
			Smoking status		
Secondary prevention educational interventions	Three/25,154 patients	Devi et al., 2015 [44]	Mortality		
			Revascularisation		
			Total Cholesterol		
			HDL cholesterol		
			Triglycerides		
			HRQOL		
			All-cause mortality		

(including Internet based secondary prevention)		Auer et al., 2008 [34]	Readmission rates		Some evidence
			Reinfarction rates		
			Smoking cessation rates		
		Clark et al., 2005 [41]	Mortality		
			MI		
			Quality of life	Most of the included studies (+)	
**Type 2 Diabetes Mellitus Patients**					
General health education	Five/2,319 patients (Choi et al., 2016; Loveman et al., 2008; Zabaleta et al., 2007 did not given the total sample size)	Choi et al., 2016 [40]	HbA1c		Some evidence
		Saffari et al., 2014 [74]	Glycemic control		
		Duke et al., 2009 [46]	HbA1c		
			BP		
			Knowledge, psychosocial outcomes and smoking habits	No Data	
			Diabetes complications or health service utilization and cost analysis	No Data	
		Loveman et al., 2008 [60]	Diabetic control outcomes	46.15% studies (+)	
			Weight	66.67% studies (+)	
			Cholesterol or triglycerides	40.00% studies (+)	
		Zabaleta et al., 2007 [79]	HbA1c	4.8% studies (+)	
		Creamer et al., 2016 [42]	HbA1c		
			HRQoL		
			AEs	No AEs	
		Ricci-Cabello et al., 2014 [73]	HbA1c		
			Diabetes knowledge	73.3% studies (+)	
			Behaviors	75% studies (+)	
			Clinical outcomes	Fasting blood glucose, HbA1c	

For peer review only

				and BP improved in 71%, 59%, and 57% of the studies.	
Culturally appropriate health education	Eight/ 20,622 patients (Ricci-Cabello et al., 2014 and Gucciardi et al., 2013 did not given the total sample size)	Attridge et al., 2014 [33]	HbA1c		Some evidence
			Knowledge scores		
			Clinical outcomes		
			Other outcome measures	Showed neutral effects.	
		Gucciardi et al., 2013 [52]	HbA1c levels	Three of ten studies (+)	
			Anthropometrics	Three of eleven studies (+)	
			Physical activity	One of five studies (+)	
			Diet outcomes	Two of six studies (+)	
		Nam et al., 2012 [62]	HbA1c level		
		Hawthorne et al., 2010 [54]	HbA1c		
			Knowledge scores		
		Khunti et al., 2008 [58]	Knowledge levels	Only one study reporting a significant improvement	
			Biomedical outcomes	Only one study reporting a significant improvement.	
		Hawthorne et al., 2008 [53]	HbA1c		
			Knowledge scores		
			Other outcome measures		
		Huang et al., 2016 [55]	HbA1c		
			BMI		
			LDL-c and HDL-c		
		Chen et al., 2015 [39]	HbA1c		
			BMI		
			SBP		
			DBP		
			HDL-c		

For peer review only

			HbA1c level		
Lifestyle interventions + behavioral program	Six/10,440 patients (Huang et al., 2016; Pillay et al., 2015 and Ramadas et al., 2011 did not given the total sample size)	Terranova et al., 2015 [72]	Weight		Some evidence
		Pillay et al., 2015 [71]	HbA1c levels		
			BMI		
		Ramadas et al., 2011 [77]	HbA1c	46.2% studies (+)	
		Gary et al., 2003 [49]	Fast blood sugar		
			Glychemoglobin		
			HbA1		
			HbA1c		
			Weight		
Self-management educational interventions	Nine/19,597 patients (Minet et al., 2010;Fan et al., 2009 and Norris et al., 2001 did not given the total sample size)	Pal et al., 2014 [67]	Cardiovascular Risk Factors		
			Cognitive Outcomes		
			Behavioral Outcomes	Only one study reporting a significant improvement.	
			AEs	No AEs	
		Vugt et al., 2013 [75]	Health behaviors	Seven of 13 studies (+)	
			Clinical outcomes measures	Nine studies (+)	
			Psychological outcomes	Nine studies (+)	
		Pal et al., 2013 [68]	HbA1c		
			Depression		
			Quality of life		
			Weight		
		Steinsbekk, et al., 2012 [76]	HbA1c		
			Main lifestyle outcomes		
			Main psychosocial outcomes		
		Minet et al., 2010 [61]	Glycaemic control		
			Diabetes knowledge		
			Overall self-management		

For peer review only

			behaviours		
		Fan et al., 2009 [47]	Overall metabolic outcomes		Sufficient evidence
			Overall weighted mean effect sizes		
		Deakin et al., 2005 [43]	Metabolic control (HbA1c)		
			Fasting blood glucose levels		
			Weight		
			Diabetes knowledge		
			SBP		
			Diabetes medication		
		Norris et al.,, 2002 [65]	Total GHb		
		Norris et al., 2001 [64]	Knowledge		
			Self-monitoring of blood glucose		
			Self-reported dietary habits		
			Glycemic control		
Therapeutic education	One/Total sample: Unclear.	Odnoletkova et al., 2014 [66]	Cost-effectiveness	Overall high in studies on prediabetes and varied in studies on T2DM.	Insufficient evidence
Foot health education	One/ Total sample: Unclear.	Amaeshi, 2012 [32]	Diabetes complications		Some evidence
			Incidence of LEA		
Group medical visit	One/2,240 patients.	Burke et al., 2011 [38]	HbA1c		
			BP and DBP		
			SBP		
			Cholesterol - LDL		Some evidence
Psychoeducational intervention.	One/1.431 patients.	Alam et al., 2009 [30]	HbA1c		Some evidence
			Psychological status		
Interventions aimed at improving adherence to	Three/ 4,907 patients (Kai Lun Gan et al., 2011 did not	Kai Lun Gan et al., 2011 [57]	Oral hypoglycaemic adherence	Five of seven studies (+)	

For peer review only - http://bmjopen.bmj.com/site/about/guidelines.xhtml

medical treatment recommendations	given the total sample size)	Wens et al., 2008 [78]	Adherence	General conclusions could not be drawn.	Some evidence
		Vermeire et al., 2005 [80]	HbA1c		
Dietary advice	One/1,467 patients	Nield et al., 2007 [63]	Glycaemic control (addition of exercise to dietary advice)		Insufficient evidence to determine
			Weight	Limited Data	
			Diabetic micro- and macrovascular diseases	Limited Data	

NOTE:

RCTs =Randomized controlled trials; HRQoL = Health Related Quality of Life; MI = Myocardial Infarction; CABG= Coronary Artery Bypass Graft Surgery; T2DM = Type 2 Diabetes Mellitus; BMI = Body Mass Index; HbA1c = Glycatedhemoglobin; BP = Blood Pressure; SBP = Systolic Blood Pressure, DBP = Diastolic Blood Pressure, HDL-c= High-density Lipoprotein Cholesterol; LDL-c = Low-density Lipoprotein Cholesterol; LEA = Lower Extremity Amputation; Adverse Events = AEs.

（+）：Intervention Group is Significantly Better than Control Group; For example: “91% studies (+)” means 91% studies reported a significant better compared with control group;

For peer review only

Table 5 Recommendations of health education programs for ACS and T2DM patients

		ACS patients	T2DM patients	Both ACS and T2DM
**Theoretical approach**		SCT, empowerment theories.	HBM; SCT.	HBM; SCT and empowerment theories.
**Behavioral strategies**		Goal setting	Goal setting	Goal setting
**Educational content**		Behavioral change (such as smoking cessation), cardiovascular risk factors, exercise, medication, and psychosocial issues.	Behavioral change, diet, exercise, glycemic control, medication, and self-management.	Behavioral change (such as smoking cessation), cardiovascular risk factors, diet, exercise, glycemic control, medication, psychosocial issues, and self-management.
**Healthcare professionals to deliver**		Nurse or multidisciplinary team	Multidisciplinary team; dietitian or nurse	Nurse or multidisciplinary team
**Teaching approaches**	Strategies	Face-to-face; telephone or mixed	Face to face, written materials; telephone or mixed	Face to face, written materials; telephone contact or mixed
	Format	Individual (One by one) or mixed	Individual (One by one) or mixed	Individual (One by one) or mixed
**Delivery timing**	Contact hours	More than 30 min per time per week	More than 30 min per time per week	More than 30 min per time per week
	Duration	At least 6 months	About 6 months	At least 6 months
**Duration of follow up**		At least 12 months	At least 12 months	At least 12 months
**Settings**		Inpatient and post-discharge settings	Hospital settings and primary care settings	Inpatient and post-discharge settings

Note: ACS = acute coronary syndrome; T2DM = type 2 diabetes mellitus; SCT=Social Cognitive Theory; HBM =Health Belief Model.

For peer review only

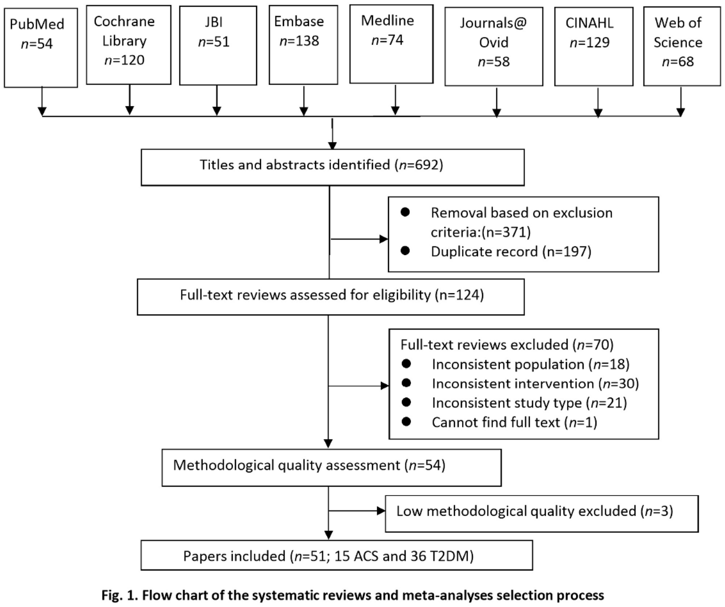

Fig. 1. Flow chart of the systematic reviews and meta-analyses selection process

215x279mm (300 x 300 DPI)

Supplementary Annex 1. Searching Strategies

Items	Searching Strategy	Results
	**PubMed**	
#1	(((((((((Acute Coronary Syndrome[MeSH Terms]) OR Myocardial Infarction[MeSH Terms]) OR Angina, Unstable[MeSH Terms]) OR Coronary Artery Disease[MeSH Terms]) OR Coronary Artery Bypass[MeSH Terms]) OR Angina Pectoris[MeSH Terms]) OR “Myocardial Infarct*”[Title/Abstract]) OR “Unstable Angina*”[Title/Abstract]) OR “coronary heart diseas*”[Title/Abstract]) OR “Coronary Artery Disease*”[Title/Abstract]) OR "Unstable Angina Pectori*"[Title/Abstract] Filters: Publication date from 2000/01/01 to 2016/04/31; English	145310
#2	(((((Diabetes Mellitus, Type 2[MeSH Terms]) OR "Type 2 Diabetes Mellitus"[Title/Abstract]) OR "type 2 diabete*"[Title/Abstract]) OR "type ii diabet*"[Title/Abstract]) OR T2DM[Title/Abstract]) OR Adult-Onset Diabetes Mellitus[Title/Abstract] Filters: Publication date from 2000/01/01 to 2016/04/31; English	80218
#3	#1 OR #2	222103
#4	(((((((Health Education[MeSH Terms]) OR Patient Education as Topic[MeSH Terms]) OR Counseling[MeSH Terms]) OR Social Support[MeSH Terms]) OR "Community Health Education*"[Title/Abstract]) OR "Educational Activit*"[Title/Abstract]) OR "Education* intervention*"[Title/Abstract]) OR "Education* program*"[Title/Abstract] Filters: Publication date from 2000/01/01 to 2016/04/31; English	122323
#5	(((Meta-Analysis [Publication Type]) OR Meta-Analysis as Topic[MeSH Terms]) OR "Systematic Revie*"[Title/Abstract]) OR "Meta-analys*"[Title/Abstract] Filters: Publication date from 2000/01/01 to 2016/04/31; English	165265
#6	#3 AND #4 AND #5 Filters: Publication date from 2000/01/01 to 2016/04/31; English	**54**
	**Cochrane Library**	
#1	MeSH descriptor: [Acute Coronary Syndrome] explode all trees	1070
#2	MeSH descriptor: [Myocardial Infarction] explode all trees	9450
#3	MeSH descriptor: [Angina, Unstable] explode all trees	1015
#4	MeSH descriptor: [Coronary Artery Disease] explode all trees	3469

For peer review only - http://bmjopen.bmj.com/site/about/guidelines.xhtml

#5	MeSH descriptor: [Coronary Artery Bypass] explode all trees	5321
#6	MeSH descriptor: [Angina Pectoris] explode all trees	4240
#7	"acute coronary syndrome":ti,ab,kw or Unstable Angina*:ti,ab,kw or Myocardial Infarct*:kw or coronary heart diseas*:ti,ab,kw or Coronary Artery Disease*:ti,ab,kw (Word variations have been searched)	29379
#8	#1 or #2 or #3 or #4 or #5 or #6 or #7	34062
#9	MeSH descriptor: [Diabetes Mellitus, Type 2] explode all trees	10474
#10	Type 2 Diabetes Mellitus:ti,ab,kw or type ii diabet*:ti,ab,kw or noninsulin dependent:ti,ab,kw or type 2 diabet*:ti,ab,kw or Adult-Onset Diabetes Mellitus:ti,ab,kw (Word variations have been searched)	20935
#11	#9 or #10	20937
#12	#8 or #11	53777
#13	MeSH descriptor: [Health Education] explode all trees	11291
#14	MeSH descriptor: [Patient Education as Topic] explode all trees	7507
#15	MeSH descriptor: [Counseling] explode all trees	4026
#16	MeSH descriptor: [Rehabilitation Nursing] explode all trees	55
#17	Education*:ti,ab,kw or Community Health Education:ti,ab,kw or Self-Management*:ti,ab,kw or Cardiac Rehabilitation:ti,ab,kw (Word variations have been searched)	40078
#18	#13 or #14 or #15 or #16 or #17	42943
#19	#12 and #18 Publication Year from 2000 to 2016, in Cochrane Reviews (Reviews only) and Other Reviews	120
#20	Cochrane reviews + other reviews	**49 + 71=120**
	**Joanna Briggs Institute,_Embase,_MEDLINE**	
#1	Acute Coronary Syndrome.m_titl.	16727
#2	Myocardial Infarction.m_titl.	151398

For peer review only - http://bmjopen.bmj.com/site/about/guidelines.xhtml

#3	Unstable Angina.m_titl.	6933
#4	Coronary Artery Disease.m_titl.	54621
#5	Coronary Artery Bypass.m_titl.	30449
#6	Angina Pectoris.m_titl.	17997
#7	Coronary Heart Disease.m_titl.	35397
#8	#1 or #2 or #3 or #4 or #5 or #6	305035
#9	Type 2 Diabetes Mellitus.mp. [mp=ti, ab, ot, nm, hw, kf, px, rx, ui, an, tx, sh, ct, sa, tn, dm, mf, dv, kw]	64926
#10	"type 2 diabet*".m_titl.	102596
#11	"type ii diabet*".m_titl.	6139
#12	Adult-Onset Diabetes Mellitus.mp. [mp=ti, ab, ot, nm, hw, kf, px, rx, ui, an, tx, sh, ct, sa, tn, dm, mf, dv, kw]	307
#13	#9 or #10 or #11 or #12	136982
#14	#8 or #13	439243
#15	Health Education.mp. [mp=ti, ab, ot, nm, hw, kf, px, rx, ui, an, tx, sh, ct, sa, tn, dm, mf, dv, kw]	177285
#16	Patient Education.mp. [mp=ti, ab, ot, nm, hw, kf, px, rx, ui, an, tx, sh, ct, sa, tn, dm, mf, dv, kw]	185631
#17	Counseling.m_titl.	23527
#18	Social Support.m_titl.	11535
#19	"Education*".m_titl.	247901
#20	#15 or #16 or #17 or #18 or #19	577203
#21	#14 and #20	6480
#22	Systematic Review.mp. [mp=ti, ab, ot, nm, hw, kf, px, rx, ui, an, tx, sh, ct, sa, tn, dm, mf, dv, kw]	212014
#23	Meta-Analysis.mp. [mp=ti, ab, ot, nm, hw, kf, px, rx, ui, an, tx, sh, ct, sa, tn, dm, mf, dv, kw]	262009
#24	#22 or #23	381113

For peer review only - http://bmjopen.bmj.com/site/about/guidelines.xhtml

#25	#21 and #24	288
#26	limit 25 to English and yr="2000 - 2016"	**263**
	**Cumulative Index to Nursing and Allied Health Literature (CINAHL)**	
#1	TI Acute Coronary Syndrome OR AB Acute Coronary Syndrome	4826
#2	TI Myocardial Infarction OR AB Myocardial Infarction	24578
#3	TI Unstable Angina OR AB Unstable Angina	1353
#4	TI Coronary Artery Disease OR AB Coronary Artery Disease	12930
#5	TI Coronary Heart Disease OR AB Coronary Heart Disease	12321
#6	#1 OR #2 OR #3 OR #4 OR #5	46436
#7	TI Type 2 Diabetes Mellitus OR AB Type 2 Diabetes Mellitus	8952
#8	TI type 2 diabet* OR AB type 2 diabet*	33085
#9	TI Adult-Onset Diabetes Mellitus OR AB Adult-Onset Diabetes Mellitus	23
#10	#7 OR #8 OR #9	33097
#11	#6 OR #10	77923
#12	TI Health Education OR AB Health Education	53415
#13	TI Patient Education OR AB Patient Education	37789
#14	TI Counseling OR AB Counseling	17673
#15	TI Social Support OR AB Social Support	30093
#16	TI Education* OR AB Education*	188291
#17	#12 OR #13 OR #14 OR #15 OR #16	227269
#18	#11 AND #17	4707
#19	TI Systematic Review OR AB Systematic Review	48830

For peer review only - http://bmjopen.bmj.com/site/about/guidelines.xhtml

#20	TI Meta-Analysis OR AB Meta-Analysis	28776
#21	#19 OR #20	64611
#22	S18 AND S21 Limiters - Published Date: 20000101-20160431; English Language	**129**
	**Web of Science**	
#1	TS=("Acute Coronary Syndrome*") OR TS=("Myocardial Infarct*") OR TS=("Unstable Angina") OR TS=("Coronary Artery Disease*") OR TS=("Coronary heart disease*") Indexes=SCI-EXPANDED, SSCI, A&HCI, CPCI-S, CPCI-SSH, BKCI-S, BKCI-SSH, ESCI, CCR-EXPANDED, IC Timespan=All years	404699
#2	TS=("Type 2 Diabetes Mellitus") OR TS=("type 2 diabete*") OR TS=("Noninsulin-Dependent Diabete*") OR TS= ("Adult-Onset Diabetes Mellitus") Indexes=SCI-EXPANDED, SSCI, A&HCI, CPCI-S, CPCI-SSH, BKCI-S, BKCI-SSH, ESCI, CCR-EXPANDED, IC Timespan=All years	103548
#3	#2 OR #1 Indexes=SCI-EXPANDED, SSCI, A&HCI, CPCI-S, CPCI-SSH, BKCI-S, BKCI-SSH, ESCI, CCR-EXPANDED, IC Timespan=All years	495081
#4	TS=("Health Education") OR TS=("Patient Education") Indexes=SCI-EXPANDED, SSCI, A&HCI, CPCI-S, CPCI-SSH, BKCI-S, BKCI-SSH, ESCI, CCR-EXPANDED, IC Timespan=All years	29830
#5	#4 AND #3 Indexes=SCI-EXPANDED, SSCI, A&HCI, CPCI-S, CPCI-SSH, BKCI-S, BKCI-SSH, ESCI, CCR-EXPANDED, IC Timespan=All years	1290
#6	TS=("Systematic Review*") OR TS=("Meta-analysis*") Indexes=SCI-EXPANDED, SSCI, A&HCI, CPCI-S, CPCI-SSH, BKCI-S, BKCI-SSH, ESCI, CCR-EXPANDED, IC Timespan=All years	149663
#7	(#6 AND #5) AND LANGUAGE: (English) Indexes=SCI-EXPANDED, SSCI, A&HCI, CPCI-S, CPCI-SSH, BKCI-S, BKCI-SSH, ESCI, CCR-EXPANDED, IC Timespan=2000-2016	**68**
	**Journals@Ovid**	
#1	Acute Coronary Syndrome.m_titl.	3021
#2	Myocardial Infarction.m_titl.	24269
#3	Unstable Angina.m_titl.	1038
#4	Coronary Artery Disease.m_titl.	10106
#5	Coronary Artery Bypass.m_titl.	6546
#6	Angina Pectoris.m_titl.	1480

For peer review only - http://bmjopen.bmj.com/site/about/guidelines.xhtml

#7	Coronary Heart Disease.m_titl.	6487
#8	#1 or #2 or #3 or #4 or #5 or #6	51548
#9	Type 2 Diabetes Mellitus. m_titl.	5097
#10	"type 2 diabet*".m_titl.	26434
#11	"type ii diabet*".m_titl.	980
#12	Adult-Onset Diabetes Mellitus. m_titl.	391
#13	#9 or #10 or #11 or #12	27795
#14	#8 or #13	78792
#15	Health Education. m_titl.	1135
#16	Patient Education. m_titl.	1556
#17	Counseling.m_titl.	5532
#18	Social Support.m_titl.	2667
#19	"Education*".m_titl.	50950
#20	#15 or #16 or #17 or #18 or #19	58969
#21	#14 and #20	510
#22	Systematic Review.mp. [mp=ti, ab, ot, nm, hw, kf, px, rx, ui, an, tx, sh, ct, sa, tn, dm, mf, dv, kw]	117790
#23	Meta-Analysis.mp. [mp=ti, ab, ot, nm, hw, kf, px, rx, ui, an, tx, sh, ct, sa, tn, dm, mf, dv, kw]	215069
#24	#22 or #23	282984
#25	#21 and #24	61
#26	limit 26 to yr="2000 - 2016"	**58**

Supplementary Annex 2.

Citation Matrix: Non-repeated trials (rows) included in the 15 systematic review and/or meta-analyses (column)--ACS.

Author-year (Trial reference)	Barth, Jacob, Daha, & Critchley, 2015	Devi et al., 2015	Ghisi, Abdallah, Grace, Thomas, & Oh, 2014	Kotb, Hsieh, & Wells, 2014	Brown, Clark, Dalal, Welch, & Taylor, 2013	Dickens et al., 2013	Aldcroft, Taylor, Blackstock, & O'Halloran P, 2011	Brown, Clark, Dalal, Welch, & Taylor, 2011	Huttunen-Lenz, Song, & Poland, 2010	Goulding, Furze, & Birks, 2010	Auer, Gaume, Rodondi, Cornuz, & Ghali, 2008	J. Barth, Critchley, & Bengel, 2008	Fernandez et al., 2007	J. Barth, Critchley, & Bengel, 2006	Clark, Hartling, Vandermeer, & McAlister, 2005
Allen JK. Coronary risk factor modification in women after coronary artery bypass surgery. Nursing Research 1996;45(5):260-5.	X											X			
Burt A, Thornley R, Illingworth D, White P, Shaw TR, Turner R. Stopping smoking after myocardial infarction. Lancet 1974;1(7852):304-6.	X											X		X	
Carlsson R, Lindberg G, Westin L, Israelsson B. Influence of coronary nursing managment follow up on lifestyle after acute myocardial infarction. Heart 1997;77(3):256-9.	X											X			
Ockene J, Kristeller JL, Goldberg R, Ockene I, Merriam P, Barrett S, et al. Smoking cessation and severity of disease: the Coronary Artery Smoking Intervention Study. Health Psychology 1992;11(2):119-26.	X											X		X	
DeBusk R, Miller NH, Superko R, Dennis CA, Thomas RJ, et al. A case-management system for coronary risk factor modification after acute myocardial infarction. Annals of Internal Medicine 1994;120(9):721-9.	X										X	X			X
Dornelas EA, Sampson RA, Gray J F, Waters D, Thompson PD. A randomized controlled trial of smoking cessation counseling after myocardial infarction. Preventive Medicine 2000;30(4):261-8.	X								X			X	X	X	
Hajek P, Taylor TZ, Mills P. Brief intervention during hospital admission to help patients to give up smoking after myocardial infarction and bypass surgery: randomised controlled trial. BMJ 2002;324:87-9.	X								X			X	X	X	
Heller RF, Knapp JC, Valenti LA, Dobson AJ. Secondary prevention after acute myocardial infarction. American Journal of Cardiology 1993;72(11):759-62.	X											X	X	X	
Moreno Ortigosa A, Ochoa Gomez FJ, Ramalle-Gomara E, Saralegui Reta I, Fernandez Esteban MV, Quintana Diaz M. Efficacy of an smoking cessationg intervention for patients with myocardial infarction [Eficacia de una intervencion para dejar de fumar en pacientes con infarto de miocardio]. Medicina Clinica 2000;114:209-10.	X											X		X	
Quist-Paulsen P, Gallefoss F. Randomised controlled trial of smoking cessation intervention after admission for coronary heart disease. BMJ 2003;327(7426):1254-7.	X								X			X		X	
Reid R, Pipe A, Higginson LK, D'Angelo MS, Cooke D, Dafoe W. Stepped care approach to smoking cessation in patients hospitalized for coronary artery disease. Journal	X											X		X	

For peer review only

of Cardiopulmonary Rehabilitation 2003;23(3):176-82.															
Rigotti NA, McKool KM, Shiffmann S. Predictors of smoking cessation after coronary artery bypass graft surgery. Annals of Internal Medicine 1994;120(4):287-93.	X								X			X		X	
Sivarajan ES, Newton KM, Almes MJ, Kempf TM, Mansfield LW, Bruce RA. Limited effects of outpatient teaching and counseling after myocardial infarction: a controlled study. Heart & Lung 1983;12(1):65-73.	X													X	
Taylor CB, Houston-Miller N, Killen JD, DeBusk RF. Smoking cessation after acute myocardial infarction: effects of a nurse-managed intervention. Annals of Internal Medicine 1990;113(2):118-23.	X								X				X	X	
Van Elderen T, Maes S, Seegers G. Effects of a group health education program for coronary heart patients after the hospitalization phase. Tijdschrift voor Psychologie & Gezondheid 1991;19(3):129-44.	X											X			
van Elderen-van Kemenade T, Maes S, van den Broek Y. Effects of a health education programme with telephone follow-up during cardiac rehabilitation. British Journal of Clinical Psychology 1994;33(Pt 3):367-78.	X		X	X							X	X		X	
Antypas K, Wangberg SC. An Internet- and mobile-based tailored intervention to enhance maintenance of physical activity after cardiac rehabilitation: short-term results of a randomized controlled trial. Journal of Medical Internet Research 2014;16(3):e77.		X													
Devi R, Powell J, Singh S. A web-based program improves physical activity outcomes in a primary care angina population: randomized controlled trial. Journal of Medical Internet Research 2014;16(9):e186.		X													
Frederix I, Van Driessche N, Hansen D, Berger J, Bonne K, Alders T, et al. Increasing the medium-term clinical benefits of hospital-based cardiac rehabilitation by physical activity telemonitoring in coronary artery disease patients. European Journal of Preventive Cardiology 2015;22(2):150-8.		X													
Lear SA, Singer J, Banner-Lukaris D, Horvat D, Park JE, Bates J, et al. Randomized trial of a virtual cardiac rehabilitation program delivered at a distance via the internet. Circulation: Cardiovascular Quality and Outcomes 2014;7(6):952-9.		X													
Lindsay S, Bellaby P, Smith S, Baker R. Enabling healthy choices: is ICT the highway to health improvement?. Health (London, England: 1997) 2008;12(3):313-31.		X													
Maddison R, Pfaeffli L, Whittaker R, Stewart R, Kerr A, Jiang Y, et al. A mobile phone intervention increases physical activity in people with cardiovascular disease: Results from the HEART randomized controlled trial. European Journal of Preventive Cardiology 2014 May 9 [Epub ahead of print].		X													
Reid RD, Morrin LI, Beaton LJ, Papadakis S, Kocourek J, McDonnell L, et al. Randomized trial of an internet-based computer-tailored expert system for physical activity in patients with heart disease. European Journal of Preventive Cardiology 2012;19(6):1357-64.		X													

For peer review only

Southard BH, Southard DR, Nuckolls J. Clinical trial of an Internet-based case management system for secondary prevention of heart disease. Journal of Cardiopulmonary Rehabilitation and Prevention 2003;23(5):341-8.		X			X	X		X					X		
Varnfield M, Karunanithi M, Lee CK, Honeyman E, Arnold D, Ding H, et al. Smartphone-based home care model improved use of cardiac rehabilitation in postmyocardial infarction patients: results from a randomised controlled trial. Heart 2014;100(22):1770-9.		X													
Vernooij JW, Kaasjager HA, van der Graaf Y, Wierdsma J, Grandjean HM, Hovens MM, et al. Internet based vascular risk factor management for patients with clinically manifest vascular disease: randomised controlled trial. BMJ (Clinical research ed.) 2012;344:e3750.		X													
Zutz A, Ignaszewski A, Bates J, Lear SA. Utilization of the Internet to deliver cardiac rehabilitation at a distance: a pilot study. Telemedicine Journal and e-Health 2007;13(3):323-30.		X													
Dale L, Whittaker R, Jiang Y, Stewart R, Rolleston A, Maddison R. Improving coronary heart disease self-management using mobile technologies (Text4Heart): a randomised controlled trial protocol. Trials 2014;15:71.		X													
ISRCTN29243064. Effectiveness of a comprehensive telerehabilitation program for the heart. http://www.controlled-trials.com/ISRCTN29243064 (accessed January 2015).		X													
NCT02228603. How to enhance physical activity after cardiac rehabilitation? A randomised controlled study comparing two follow-up training exercise programs. https://clinicaltrials.gov/show/NCT02228603 (accessed January 2015).		X													
NCT02350192. Effectiveness of an e-health educational intervention for cardiovascular disease adults in improving total exercise and outcomes. https://clinicaltrials.gov/ct2/show/NCT02350192 (accessed January 2015).		X													
Redfern J, Usherwood T, Harris MF, Rodgers A, Hayman N, Panaretto K, et al. A randomised controlled trial of a consumer-focused e-health strategy for cardiovascular risk management in primary care: the Consumer Navigation of Electronic Cardiovascular Tools (CONNECT) study protocol. BMJ Open 2014; Vol. 4, issue 2. [DOI: 10.1136/bmjopen-2013-004523]		X													
Reinwand D, Kuhlmann T, Wienert J, Vries HD, Lippke S. Designing a theory- and evidence-based tailored eHealth rehabilitation aftercare program in Germany and the Netherlands: study protocol. BMC Public Health 2013;13:1081.		X													
Shah B, Adams M, Peterson E, Powers B, Oddone E, Royal K, et al. Secondary Prevention Risk Interventions Via Telemedicine and Tailored Patient Education (SPRITE): A randomized trial to improve postmyocardial infarction management. Circulation: Cardiovascular Quality and Outcomes 2011;4(2):235-42.		X													

For peer review only - http://bmjopen.bmj.com/site/about/guidelines.xhtml

T. Buckley, S. McKinley, R. Gallagher, K. Dracup, D.K. Moser, L.M. Aitken<br>The effect of education and counselling on knowledge, attitudes and beliefs about responses to acute myocardial infarction symptoms<br>Eur J Cardiovasc Nurs, 6 (2007), pp. 105–111			x							x					
J.J. Carlson, G.J. Norman, D.L. Feltz, B.A. Franklin, J.A. Johnson, S.K. Locke<br>Self-efficacy, psychosocial factors, and exercise behavior in traditional versus modified cardiac rehabilitation<br>J Cardpulm Rehabil, 21 (2001), pp. 363–373			x												
S.S. Chan, D.Y. Leung, D.C. Wong, C.P. Lau, V.T. Wong, T.H. Lam<br>A randomized controlled trial of stage-matched intervention for smoking cessation in cardiac-outpatients<br>Addiction, 107 (2012), pp. 829–837			x												
K.M. Cordasco, S.M. Asch, D.S. Bell, J.J. Guterman, S. Gross-Schulman, L. Ramer, et al.<br>A low-literacy medication education tool for safety-net hospital patients<br>Am J Prev Med, 37 (2009), pp. S209–S216			x												
M.H. Eckman, R. Wise, A.C. Leonard, E. Dixon, C. Burrows, F. Khan, et al.<br>Impact of health literacy on outcomes and effectiveness of an educational intervention in patients with chronic diseases<br>Patient Educ Couns, 87 (2012), pp. 143–151			x												
P. Giannuzzi, P.L. Temporelli, R. Marchioli, A.P. Maggioni, G. Balestroni, V. Ceci, et al.<br>Global secondary prevention strategies to limit event recurrence after myocardial infarction: results of the GOSPEL study, a multicenter, randomized controlled trial from the Italian Cardiac Rehabilitation Network<br>Arch Intern Med, 168 (2008), pp. 2194–2204			x												
K.B. Heidal, N.M. Lewis, S.M. Evans, L. Boeckner<br>Nutrition education intervention increases total n − 3 fatty acid intakes in heart patients living in the Midwest<br>Nutr Res, 27 (2007), pp. 33–37			x												
L. Horlick, R. Cameron, W. Firor, U. Bhalerao, R. Baltzan<br>The effects of education and group discussion in the post myocardial infarction patient<br>J Psychosom Res, 28 (1984), pp. 485–492			x												
H.S. Jeong, J.S. Chae, J.S. Moon, Y.S. Yoo<br>An individualized teaching program for atherosclerotic risk factor reduction in patients with myocardial infarction<br>Yonsei Med J, 43 (2002), pp. 93–100			x												
X. Jiang, J.W. Sit, T.K. Wong<br>A nurse-led cardiac rehabilitation programme improves health behaviours and cardiac physiological risk			x												

For peer review only

parameters: evidence from Chengdu, China<br>J Clin Nurs, 16 (2007), pp. 1886–1897															
M. Kummel, T. Vahlberg, A. Ojanlatva, R. Karki, T. Mattila, S.L. Kivela<br>Effects of an intervention on health behaviors of older coronary artery bypass (CAB) patients<br>Arch Gerontol Geriatr, 46 (2008), pp. 227–244			x												
W.S. Leslie, C.R. Hankey, D. Matthews, J.E.P. Currall, M.E.J. Lean<br>A transferable programme of nutritional counselling for rehabilitation following myocardial infarction: a randomised controlled study<br>Eur J Clin Nutr, 58 (2004), pp. 778–786			x												
E. Lidell, B. Fridlund<br>Long-term effects of a comprehensive rehabilitation programme after myocardial infarction<br>Scand J Caring Sci, 10 (1996), pp. 67–74			x							x					
S. Lindsay, S. Smith, P. Bellaby, R. Baker<br>The health impact of an online heart disease support group: a comparison of moderated versus unmoderated support<br>Health Educ Res, 24 (2009), pp. 646–654			x												
R.A. Mayou, D.R. Thompson, A. Clements, C.H. Davies, S.J. Goodwin, K. Normington, et al.<br>Guideline-based early rehabilitation after myocardial infarction: a pragmatic randomised controlled trial<br>J Psychosom Res, 52 (2002), pp. 89–95			x								x				
S. McKinley, K. Dracup, D.K. Moser, B. Riegel, L.V. Doering, H. Meischke, et al.<br>The effect of a short one-on-one nursing intervention on knowledge, attitudes and beliefs related to response to acute coronary syndrome in people with coronary heart disease: a randomized controlled trial<br>Int J Nurs Stud, 46 (2009), pp. 1037–1046			x												
S.M. Moore, M.A. Dolansky<br>Randomized trial of a home recovery intervention following coronary artery bypass surgery<br>Res Nurs Health, 24 (2001), pp. 93–104			x			x									
B. Oldenburg, A. Martin, J. Greenwood, L. Bernstein, R. Allan<br>A controlled trial of a behavioral and educational intervention following coronary artery bypass surgery<br>J Cardpulm Rehabil, 15 (1995), pp. 39–46			x												
J.E. Otterstad<br>Influence on lifestyle measures and five-year coronary risk by a comprehensive lifestyle intervention programme in patients with coronary heart disease<br>Eur J Cardiovasc Prev Rehabil, 10 (2003), pp. 429–437			x												x
P.M. Smith, E. Burgess			x						x						

For peer review only

Smoking cessation initiated during hospital stay for patients with coronary artery disease: a randomized controlled trial<br>CMAJ, 180 (2009), pp. 1297–1303															
T.M. Van Elderen, S. Maes, G. Seegers, H. Kragten, L. Relik-Can Wely<br>Effects of a post-hospitalization group health education programme for patients with coronary heart disease<br>Psychol Health, 9 (1994), pp. 317–330			X											X	
J. Wolkanin-Bartnik, H. Pogorzelska, A. Bartnik<br>Patient education and quality of home-based rehabilitation in patients older than 60 years after acute myocardial infarction<br>J Cardiopulm Rehabil Prev, 31 (2011), pp. 249–253			X												
C. Froger-Bompas, B. Laviolle, P. Guillo, C. Letellier, K. Ligier, J.C. Daubert, et al.<br>Sustained positive impact of a coronary rehabilitation programme on adherence to dietary recommendations<br>Arch Cardiovasc Dis, 102 (2009), pp. 97–104			X												
B.J. Linde, N.M. Janz<br>Effect of a teaching program on knowledge and compliance of cardiac patients<br>Nurs Res, 28 (1979), pp. 282–286			X												
J. Oliveira, F. Ribeiro, H. Gomes<br>Effects of a home-based cardiac rehabilitation program on the physical activity levels of patients with coronary artery disease<br>J Cardiopulm Rehabil Prev, 28 (2008), pp. 392–396			X												
R. Song, H. Lee<br>Managing health habits for myocardial infarction (MI) patients<br>Int J Nurs Stud, 38 (2001), pp. 375–380			X												
B.L. Verges, B. Patois-Verges, M. Cohen, J.M. Casillas<br>Comprehensive cardiac rehabilitation improves the control of dyslipidemia in secondary prevention<br>J Cardpulm Rehabil, 18 (1998), pp. 408–415			X												
T. Yoshida, M. Kohzuki, K. Yoshida, M. Hiwatari, M. Kamimoto, C. Yamamoto, et al.<br>Physical and psychological improvements after phase II cardiac rehabilitation in patients with myocardial infarction<br>Nurs Health Sci, 1 (1999), pp. 163–170			X												
G.F. Fletcher<br>Rehabilitation after coronary angioplasty—is it effective<br>Arch Phys Med Rehabil, 67 (1986), pp. 517–519			X												
C.J. Frame, C.G. Green, D.G. Herr, M.L. Taylor<br>A 2-year stage of change evaluation of dietary fat and fruit and vegetable intake behaviors of cardiac rehabilitation patients			X												

For peer review only

Am J Health Promot, 17 (2003), pp. 361–368															
S. Pelletier, S. Kundrat, C.M. Hasler<br>Effects of a functional foods nutrition education program with cardiac rehabilitation patients<br>J Cardpulm Rehabil, 23 (2003), pp. 334–340			x												
L. Jensen<br>Self-administered cardiac medication program evaluation<br>Can J Cardiovasc Nurs, 13 (2003), pp. 35–44			x												
J. Lisspers, C. Hofman-Bang, R. Nordlander, L. Ryden, O. Sundin, A. Ohman, et al.<br>Multifactorial evaluation of a program for lifestyle behavior change in rehabilitation and secondary prevention of coronary artery disease<br>Scand Cardiovasc J, 33 (1999), pp. 9–16			x												
A. Palomaki, S. Miilunpalo, P. Holm, E. Makinen, K. Malminiem<br>Effects of preventive group education on the resistance of LDL against oxidation and risk factors for coronary heart disease in bypass surgery patients<br>Ann Med, 34 (2002), pp. 272–283			x												
M.T. Timlin, K.V. Shores, M. Reicks<br>Behavior change outcomes in an outpatient cardiac rehabilitation program<br>J Am Diet Assoc, 102 (2002), pp. 664–671			x												
C. Bellman, K. Hambraeus, J. Lindbäck, B. Lindahl<br>Achievement of secondary preventive goals after acute myocardial infarction: a comparison between participants and nonparticipants in a routine patient education program in Sweden<br>J Cardiovasc Nurs, 24 (2009), pp. 362–368			x												
K. Dracup, A.I. Meleis, S. Clark, A. Clyburn, L. Shields, M. Staley<br>Group counseling in cardiac rehabilitation: effect on patient compliance<br>Patient Educ Couns, 6 (1984), pp. 169–177			x				x								
Z. Irmak, H. Fesci<br>Effects of nurse-managed secondary prevention program on lifestyle and risk factors of patients who had experienced myocardial infarction<br>Appl Nurs Res, 23 (2010), pp. 147–152			x												
J. Marshall, S. Penckofer, J. Llewellyn<br>Structured postoperative teaching and knowledge and compliance of patients who had coronary artery bypass surgery<br>Heart Lung, 15 (1986), pp. 76–82			x												
T. Van Elderen, E. Dusseldorp<br>Lifestyle effects of group health education for patients with coronary heart disease			x												

For peer review only

Psychol Health, 16 (2001), pp. 327–341															
P. Davidson, M. Digiacomo, R. Zecchin, M. Clarke, G. Paul, K. Lamb, et al. A cardiac rehabilitation program to improve psychosocial outcomes of women with heart disease J Womens Health, 17 (2008), pp. 123–134			X												
Arthur HM, Smith KM, Kodis J, McKelvie R (2002) A controlled trial of hospital versus home-based exercise in cardiac patients. Medicine and Science in Sports and Exercise. 34(10): 15441550				X											
Bambauer KZ, Aupont O, Stone PH, Locke SE, Mullan MG, et al. (2005) "The effect of a telephone counseling intervention on self-rated health of cardiac patients.". Psychosomatic Medicine 67(4): 539–545.				X											
Bazargani RH, Besharat MA, Ehsan HB, Nejatian M, Hosseini K (2011) The efficacy of Chronic Disease Self Management Programs and Tele-health on psychosocial adjustment by increasing self-efficacy in patients with CABG. Procedia - Social and Behavioral Sciences 30: 817–821.				X											
Beckie T (1989) A supportive-educative telephone program: Impact on knowledge and anxiety after coronary artery bypass graft surgery. Heart and Lung: Journal of Critical Care 18(1): 46–55.				X											
Gallagher R, McKinley S, Dracup K (2003) Effects of a telephone counseling intervention on psychosocial adjustment in women following a cardiac event. Heart and Lung: Journal of Acute and Critical Care 32(2): 79–87.				X		X									
Hanssen TA, Nordrehaug JE, Eide GE, Rokne B (2007) Improving outcomes after myocardial infarction: A randomized controlled trial evaluating effects of a telephone follow-up intervention. European Journal of Cardiovascular Prevention and Rehabilitation 14 (3): 429–437.				X				X							
Hanssen TA, Nordrehaug JE, Eide GE, Hanestad BR (2009) Does a telephone follow-up intervention for patients discharged with acute myocardial infarction have long-term effects on health-related quality of life? A randomised controlled trial. Journal of Clinical Nursing 18(9): 1334–1345.				X											
Hartford K, Wong C, Zakaria D (2002) Randomized controlled trial of a telephone intervention by nurses to provide information and support to patients and their partners after elective coronary artery bypass graft surgery: Effects of anxiety. Heart and Lung: Journal of Acute and Critical Care, 31(3), 199–206.				X											
Holmes-Rovner M, Stommel M, Corser WD, Olomu A, Holtrop JS, et al. (2008) Does outpatient telephone coaching add to hospital quality improvement following				X		X									

For peer review only

hospitalization for acute coronary syndrome? Journal of General Internal Medicine 23(9): 1464–70.															
Ma Y, Ockene IS, Rosal MC, Merriam PA, Ockene JK, et al. (2010) Randomized trial of a pharmacist-delivered intervention for improving lipid-lowering medication adherence among patients with coronary heart disease. Cholesterol 2010: 1–11.				x											
McLaughlin TJ, Aupont O, Bambauer K, Stone P, Mullan MG, et al. (2005) Improving psychologic adjustment to chronic illness in cardiac patients: The role of depression and anxiety. Journal of General Internal Medicine 20(12): 1084–1090.				x											
Mittag O, China C, Hoberg E, Juers E, Kolenda K, et al. (2006) Outcomes of cardiac rehabilitation with versus without a follow-up intervention rendered by telephone (Luebeck follow-up trial): overall and gender-specific effects. International journal of rehabilitation research Internationale Zeitschrift fur Rehabilitationsforschung. Revue internationale de recherches de readaptation 29(4): 295–302.				x		x									
Neubeck L, Redfern J, Briffa T, Ascanio R, Freedman SB, et al. (2009) Cardiovascular risk benefits of the CHOICE (Choice of Health Options In prevention of Cardiovascular Events) program are maintained for four years: Randomised controlled trial. European Heart Journal Conference: European Society of Cardiology, ESC Congress 2009 Barcelona Spain. Conference Start: 20090829 Conference End: 20090902. Conference Publication: (var.pagings) 30: 76.				x											
Neubeck L, Freedman SB, Briffa T, Bauman A, Redfern J (2011) Four-year follow-up of the choice of health options in prevention of cardiovascular events randomized controlled trial. European Journal of Cardiovascular Prevention and Rehabilitation.18(2)278286				x											
Redfern J, Briffa T, Ellis E, Freedman SB (2008) Patient-centered modular secondary prevention following acute coronary syndrome: A randomized controlled trial. Journal of Cardiopulmonary Rehabilitation and Prevention.28(2)107117				x											
Redfern J, Briffa T, Ellis E, Freedman SB (2009) Choice of secondary prevention improves risk factors after acute coronary syndrome: 1-Year follow-up of the CHOICE (Choice of Health Options in prevention of Cardiovascular Events) randomised controlled trial. Heart 95 (6): 468–475.				x											
Redfern J, Menzies M, Briffa T, Freedman SB (2010) Impact of medical consultation frequency on modifiable risk factors and medications at 12 months after acute coronary syndrome in the CHOICE randomised controlled trial. International Journal of Cardiology 145 (3): 481–486.				x											
Reid RD, Pipe AL, Quinlan B, Oda J (2007) Interactive				x					x						

For peer review only

voice response telephony to promote smoking cessation in patients with heart disease: A pilot study. Patient Education and Counseling 66 (3): 319–326.															
Smith KM, Arthur HM, McKelvie RS, Kodis J (2004) Differences in sustainability of exercise and health-related quality of life outcomes following home or hospital-based cardiac rehabilitation. European Journal of Cardiovascular Prevention and Rehabilitation 11(4): 313–319.				X											
Smith KM (2007) Sustainability of exercise capacity and quality of life after home or hospital based exercise training in low-risk patients following coronary artery bypass graft surgery: a six-year follow-up of a randomized controlled trial, McMaster University (Canada): 176 p–176 p.				X											
Smith KM, McKelvie RS, Thorpe KE, Arthur HM (2011) Six-year follow-up of a randomised controlled trial examining hospital versus home-based exercise training after coronary artery bypass graft surgery. Heart 97(14): 1169–1174.				X											
Stevens B (1985) The effectiveness of patient education follow-up by telephone on knowledge of post-myocardial infarction patients. (M.Nus., University of Alberta (Canada)). ProQuest Dissertations and Theses (303461144).				X											
Tranmer JE, Parry MJE (2004) Enhancing postoperative recovery of cardiac surgery patients - A randomized clinical trial of an advanced practice nursing intervention. Western Journal of Nursing Research 26(5): 515–532.				X											
Vale MJ, Jelinek MV, Best JD, Dart AM, Grigg LE, et al. (2003) Coaching patients on achieving cardiovascular health (COACH): A multicenter randomized trial in patients with coronary heart disease. Archives of Internal Medicine–. 163(22)27752783				X		X							X		X
Vale MJ, Jelinek MV, Best JD, Santamaria JD (2002) Coaching patients with coronary heart disease to achieve the target cholesterol: A method to bridge the gap between evidence-based medicine and the "real world" - randomized controlled trial. Journal of Clinical Epidemiology. 55(3)245252				X											X
Clark NM, Janz NK, Becker MH, . Impact of self-management education on the functional health status of older adults with heart disease. The Gerontologist 1992; 32: 438–443.					X			X							
Clark NM, Janz NK, Dodge JA, . Self-Management of heart disease by older adults. Res Aging 1997; 19: 362–382.					X										
Clark NM, Janz NK, Dodge JA, . Changes in functional health status of older women with heart disease: evaluation of a program based on self-regulation. J					X			X							

For peer review only

Gerontol B Psychol Sci Soc Sci 2000; 55B: S117–S126.															
Wheeler JR. Can a disease self-management programme reduce health care costs: the case of older women with heart disease. Med Care 2003; 41: 706–715					X										
Clark NM, Janz NK, Dodge JA, . Heart disease management by women: does intervention format matter? Health Educ Behav 2009; 36: 394–409					X			X							
Cupples ME, McKnight A, O'Neill C, . The effect of personal health education on the quality of life of patients with angina in general practice. Health Educ J 1996; 55: 75–83					X										
Cupples ME, McKnight A. Five year follow up of patients at high cardiovascular risk who took part in randomised controlled trial of health promotion. Brit Med J 1999; 319: 687–688.					X			X							X
Cupples ME, McKnight A. Randomised controlled trial of health promotion in general practice for patients at high cardiovascular risk. Brit Med J 1994; 309: 993–996					X										X
O'Neill C, Normand C, Cupples M, . Cost effectiveness of personal health education in primary care for people with angina in the greater Belfast area of Northern Ireland. J Epidemiol Community Health 1996; 50: 538–540.					X										
Esposito D, Brown R, Chen A, . Impacts of a disease management program for dually eligible beneficiaries. Health Care Finan Rev 2008; 30: 27–45.					X			X							
Hanssen TA, Nordrehaug JE, Eide BE, . Improving outcomes after myocardial infarction: a randomized controlled trial evaluating effects of a telephone follow-up intervention. Eur J Cardiovasc Prevent Rehab 2007; 14(3): 429–437.					X										
Hanssen TA, Nordrehaug JE, Eide GE, . Can telephone follow-up after discharge improve life style factors after a myocardial infarction? A randomized controlled trial. 7th Annual Cardiovascular Nursing Spring Meeting of the European Society of Cardiology Council on Cardiovascular Nursing and Allied Professions: changing practice to improve care Manchester, UK 23–24 March 2007. Eur J Cardiovasc Nurs 2007; 6: S43–S44.					X										
Hanssen TA, Nordrehaug JE, Eide GE, . Does a telephone follow-up intervention for patients discharges with acute myocardial infarction have long-term effects on health-related quality of life? A randomised controlled trial. J Clin Nurs 2009; 18: 1334–1345					X										
Hanssen TA, Nordrehaug JE, Eide GE, . Evaluating the effect of a combined reactive and proactive telephone follow-up intervention after acute myocardial infarction. A randomized controlled trial. Eur J Cardiovasc Nurs 2006;					X										

For peer review only

5: S46–S46.															
Lie I, Arnesen H, Sandvik L, . Health-related quality of life after coronary artery bypass grafting. The impact of a randomised controlled home-based intervention program. Qual Life Res 2009; 18: 201–207.					X			X							
Hofman-Bang C, Lisspers J, Nordlander R, . Two-year results of a controlled study of residential rehabilitation for patients treated with percutaneous transluminal coronary angioplasty. A randomized study of a multifactorial programme. Eur Heart J 1999; 20: 1465–1474.					X	X		X							
Lisspers J, Sundin O, Hofman-Bang C, . Behavioural effects of a comprehensive, mutifactorial program for lifestyle change after percutaneous transluminal coronary angioplasty: a prospective, randomized, controlled study. J Psychosom Res 1999; 46: 143–154.					X										
Lisspers J, Sundin O, Ohman A, . Long-term effects of lifestyle behavior change in coronary artery disease: effects on recurrent coronary events after percutaneous coronary intervention. Health Psychol 2005; 24: 41–48.					X										
P.RE.COR. Group. Comparison of a rehabilitation programme, a counselling programme and usual care after an acute myocardial infarction: results of a long-term randomized trial. Eur Heart J 1991; 12: 612–616.					X			X							X
Brown R, Peikes D, Chen A, . 15-Site randomized trial of coordinated care in medicare FFS. Health Care Finan Rev 2008; 30: 5–25					X			X							
Peikes D, Chen A, Schore J, . Effects of care coordination on hospitalization, quality of care, and health care expenditures among medicare beneficiaries 15 randomized trials. J Amer Med Assoc 2009; 301: 603–618.					X										
Pogosova GV, Kalinina AM, Spivak EI, . Efficacy of an educational preventive technology in patients with stable angina in ambulatory conditions. Kardiologiia 2008; 48(7): 4–9.					X			X							
Tingstrom PR, Kamwendo K, Bergdahl B. Effects of a problem-based learning rehabilitation programme on quality of life in patients with coronary artery disease. Eur J Cardiovasc Nurs 2005; 4: 324–330					X			X							
Appels A, B„r F, van der PG, et al. Effects of treating exhaustion in angioplasty patients on new coronary events: results of the randomized Exhaustion Intervention Trial (EXIT). Psychosomatic Medicine. 2005;67(2):217-223.						X									
Ashton RC, Whitworth GC, Seldomridge JA, et al. The effects of self-hypnosis on quality of life following coronary artery bypass surgery: preliminary results of a prospective, randomized trial. Journal of Alternative and						X									

For peer review only

Complementary Medicine 1995;1(3):285-290.															
Barr Taylor CB, Houston-Miller N, Ahn DK, Haskell W, DeBusk RF. The effects of exercise training programs on psychosocial improvement in uncomplicated postmyocardial infarction patients. Journal of Psychosomatic Research. 1986;30(5):581-587.						x									
Berkman LF, Blumenthal J, Burg M, et al. Effects of treating depression and low perceived social support on clinical events after myocardial infarction: the Enhancing Recovery in Coronary Heart Disease Patients (ENRICHD) Randomized Trial. JAMA : the journal of the American Medical Association. 2003;289(23):3106-3116.						x									
Blumenthal JA, Sherwood A, Babyak MA, et al. Effects of exercise and stress management training on markers of cardiovascular risk in patients with ischemic heart disease: a randomized controlled trial. JAMA : the journal of the American Medical Association. 2005;293(13):1626-1634.						x									
Brown MA, Munford AM, Munford PR. Behavior therapy of psychological distress in patients after myocardial infarction or coronary bypass. Journal of Cardiopulmonary Rehabilitation. 1993;13(3):201-210.						x									
Burgess AW, Lerner DJ, D'Agostino RB, Vokonas PS, Hartman CR, Gaccione P. A randomized control trial of cardiac rehabilitation. Social Science & Medicine. 1987;24(4):359-370.						x									
Claesson M, Birgander LS, Lindahl B, et al. Women's hearts-stress management for women with ischemic heart disease: explanatory analyses of a randomized controlled trial. Journal of Cardiopulmonary Rehabilitation. 2005;25(2):93-102.						x									
de Klerk JE, du Plessis WF, Steyn HS, Botha M. Hypnotherapeutic ego strengthening with male South African coronary artery bypass patients. The American Journal of Clinical Hypnosis. 2004;47(2):79-92.						x									
Dugmore LD, Tipson RJ, Phillips MH, et al. Changes in cardiorespiratory fitness, psychological wellbeing, quality of life, and vocational status following a 12 month cardiac exercise rehabilitation programme. Heart. 1999;81(4):359-366						x									x
Engblom E, Korpilahti K, H„m„l„inen H, R"nnemaa T, Puukka P. Quality of life and return to work 5 years after coronary artery bypass surgery. Long-term results of cardiac rehabilitation. Journal of Cardiopulmonary Rehabilitation. 1997;17(1):29-36.						x									x
Frasure-Smith N, Lesperance F, Prince RH, et al. Randomised trial of home-based psychosocial nursing intervention for patients recovering from myocardial infarction. Lancet. 1997;350(9076):473-479.						x									x
Freedland KE, Skala JA, Carney RM, et al. Treatment of depression after coronary artery bypass surgery: A randomized controlled trial. Archives of General Psychiatry. 2009;66(4):387-396.						x									

For peer review only

Fridlund B, H”gstedt B, Lidell E, Larsson PA. Recovery after myocardial infarction. Effects of a caring rehabilitation programme. Scandinavian Journal of Caring Sciences. 1991;5(1):23-32.						X									X
Frizelle DJ, Lewin RJ, Kaye G, et al. Cognitive-behavioural rehabilitation programme for patients with an implanted cardioverter defibrillator: a pilot study. British Journal of Health Psychology. 2004;9(3):381-392.						X									
Furze G, Dumville JC, Miles JNV, Irvine K, Thompson DR, Lewin RJP. "Prehabilitation" prior to CABG surgery improves physical functioning and depression. International Journal of Cardiology. 2009;132:51-58.						X									
Goodman H, Parsons A, Davison J, et al. A randomised controlled trial to evaluate a nurse-led programme of support and lifestyle management for patients awaiting cardiac surgery 'Fit for surgery: Fit for life' study. European Journal of Cardiovascular Nursing. 2008;7(3):189-195.						X									
Hughes AR, Mutrie N, Macintyre PD. Effect of an exercise consultation on maintenance of physical activity after completion of phase III exercise-based cardiac rehabilitation. European Journal of Cardiovascular Prevention and Rehabilitation 2007;14(1):114-121.						X									
Johnston M, Foulkes J, Johnston DW, Pollard B, Gudmundsdottir H. Impact on patients and partners of inpatient and extended cardiac counseling and rehabilitation: a controlled trial. Psychosomatic Medicine. 1999;61(2):225-233.						X					X				X
Jones DA, West RR. Psychological rehabilitation after myocardial infarction: multicentre randomised controlled trial. BMJ (Clinical research ed.). 1996;313(7071):1517-1521.						X									X
Karlsson MR, Edstr”m-Ploss C, Held C, Henriksson P, Billing E, Wall,n NH. Effects of expanded cardiac rehabilitation on psychosocial status in coronary artery disease with focus on type D characteristics. Journal of Behavioral Medicine. 2007;30(3):253-261.						X									
Koertge J, Janszky I, Sundin O, et al. Effects of a stress management program on vital exhaustion and depression in women with coronary heart disease: a randomized controlled intervention study. Journal of Internal Medicine. 2008;263(3):281-293.						X									
Leserman J, Stuart EM, Mamish ME, Benson H. The efficacy of the relaxation response in preparing for cardiac surgery. Behavioral Medicine. 1989;15(3):111-117.						X									
Lesperance F, Frasure-Smith N, Koszycki D, et al. Effects of citalopram and interpersonal psychotherapy on depression in patients with coronary artery disease: the Canadian Cardiac Randomized Evaluation of Antidepressant and Psychotherapy Efficacy (CREATE) trial. JAMA : the journal of the American Medical Association. 2007;297(4):367-379.						X									
Lewin B, Robertson IH, Cay EL, Irving JB, Campbell M.						X									

Effects of self-help post-myocardial-infarction rehabilitation on psychological adjustment and use of health services. Lancet. 1992;339(8800):1036-1040.															
Lewin RJ, Coulton S, Frizelle DJ, Kaye G, Cox H. A brief cognitive behavioural preimplantation and rehabilitation programme for patients receiving an implantable cardioverter-defibrillator improves physical health and reduces psychological morbidity and unplanned readmissions. Heart. 2009;95:63-69.						X									
Lewin RJ, Furze G, Robinson J, et al. A randomised controlled trial of a self-management plan for patients with newly diagnosed angina. The British Journal of General Practice 2002;52(476):194-201.						X				X					
Lewin RJ, Thompson DR, Elton RA. Trial of the effects of an advice and relaxation tape given within the first 24 h of admission to hospital with acute myocardial infarction. International Journal of Cardiology. 2002;82(2):107-114.						X					X		X		
Lie I, Arnesen H, Sandvik L, Hamilton G, Bunch EH. Effects of a home-based intervention program on anxiety and depression 6 months after coronary artery bypass grafting: a randomized controlled trial. Journal of Psychosomatic Research. 2007;62(4):411-418.						X									
Linden B. Evaluation of a home-based rehabilitation programme for patients recovering from acute myocardial infarction. Intensive & Critical Care Nursing 1995;11(1):10-19.						X					X				
McHugh F, Lindsay GM, Hanlon P, et al. Nurse led shared care for patients on the waiting list for coronary artery bypass surgery: a randomised controlled trial. Heart 2001;86(3):317-323.						X									X
McLaughlin TJ, Aupont O, Bambauer KZ, et al. Improving psychologic adjustment to chronic illness in cardiac patients. The role of depression and anxiety. Journal of General Internal Medicine 2005;20(12):1084-1090.						X									
Michalsen A, Grossman P, Lehmann N, et al. Psychological and quality-of-life outcomes from a comprehensive stress reduction and lifestyle program in patients with coronary artery disease: results of a randomized trial. Psychotherapy and Psychosomatics. 2005;74(6):344-352.						X									
Murchie P, Campbell NC, Ritchie LD, Deans HG, Thain J. Effects of secondary prevention clinics on health status in patients with coronary heart disease: 4 year follow-up of a randomized trial in primary care. Family Practice. 2004;21(5):567-574.						X									X
Newton M, Mutrie N, McArthur JD. The effects of exercise in a coronary rehabilitation programme. Scottish Medical Journal. 1991;36(2):38-41.						X									
Oldridge N, Guyatt G, Jones N, et al. Effects on quality of life with comprehensive rehabilitation after acute myocardial infarction. The American Journal of Cardiology. 1991;67(13):1084-1089.						X									X
Paul-Labrador M, Polk D, Dwyer JH, et al. Effects of a randomized controlled trial of transcendental meditation						X									

For peer review only - http://bmjopen.bmj.com/site/about/guidelines.xhtml

on components of the metabolic syndrome in subjects with coronary heart disease. Archives of Internal Medicine. 2006;166(11):1218-1224.															
Postlethwaite R, Stirling G, Peck CL. Stress inoculation for acute pain: a clinical trial. Journal of Behavioral Medicine. 1986;9(2):219-227.						X									
Scholz U, Knoll N, Sniehotta FF, Schwarzer R. Physical activity and depressive symptoms in cardiac rehabilitation: long-term effects of a self-management intervention. Social Science & Medicine 2006;62(12):3109-3120.						X									
Schrader G, Cheok F, Hordacre AL, Marker J, Wade V. Effect of psychiatry liaison with general practitioners on depression severity in recently hospitalised cardiac patients: a randomised controlled trial. The Medical Journal of Australia. 2005;182(6):272-276.						X									
Sebregts EH, Falger PR, Appels A, Kester AD, B„r FW. Psychological effects of a short behavior modification program in patients with acute myocardial infarction or coronary artery bypass grafting. A randomized controlled trial. Journal of Psychosomatic Research. 2005;58(5):417-424.						X									
Seki E, Watanabe Y, Sunayama S, et al. Effects of phase III cardiac rehabilitation programs on health-related quality of life in elderly patients with coronary artery disease: Juntendo Cardiac Rehabilitation Program (J-CARP). Circulation Journal 2003;67(1):73-77.						X									X
Shuldham CM, Fleming S, Goodman H. The impact of pre-operative education on recovery following coronary artery bypass surgery. A randomized controlled clinical trial. European Heart Journal. 2002;23(8):666-674.						X									
Sneed NV, Finch NJ, Michel Y. The effect of psychosocial nursing intervention on the mood state of patients with implantable cardioverter defibrillators and their caregivers. Progress In Cardiovascular Nursing. 1997;12(2):4-14.						X									
Sorlie T, Busund R, Sexton J, Sexton H, Sorlie D. Video information combined with individualized information sessions: Effects upon emotional well-being following coronary artery bypass surgery-A randomized trial. Patient Education & Counseling. 2007;65(2):180-188.						X									
Stern MJ, Gorman PA, Kaslow L. The group counseling v exercise therapy study. A controlled intervention with subjects following myocardial infarction. Archives of Internal Medicine. 1983;143(9):1719-1725.						X									X
Thompson DR, Meddis R. A prospective evaluation of in hospital counselling for first time myocardial infarction men. Journal of Psychosomatic Research. 1990;34(3):237-248.						X									
Trzcieniecka-Green A, Steptoe A. The effects of stress management on the quality of life of patients following acute myocardial infarction or coronary bypass surgery. European Heart Journal. 1996;17(11):1663-1670.						X									
Unden AL, Schenck-Gustafsson K, Axelsson PO, Karlsson I, Orth-Gom,r K, Ydrefors AM. Positive effects of						X									

For peer review only

increased nurse support for male patients after acute myocardial infarction. Quality of Life Research 1993;2(2):121-127.															
Barth J. Depression as a Risk Factor for Mortality in Patients With Coronary Heart Disease: A Meta-analysis. 2004.						X									
Merswolken M. Treating anxiety in patients with coronary heart disease: a randomized controlled trial. 2011.						X									
utwalli HA. Randomized controlled evaluation shows the effectiveness of a home-based cardiac rehabilitation program. 2012.						X									
Oranta O. The outcomes of interpersonal counselling on depressive symptoms and distress after myocardial infarction. 2010.						X									
Poortaghi S. General health improves with home-based cardiac rehabilitation program. 2011.						X									
Utriyaprasit K. Recovery after coronary artery bypass surgery: effect of an audiotape information programme. 2010.						X									
Izawa KP, Watanabe S, Omiya K, et al.. Effect of the self-monitoring approach on exercise maintenance during cardiac rehabilitation: a randomized controlled trial. Am J Phys Med Rehabil. 2005;84:313–321.a							X								
Yates BC, Anderson T, Hertzog M, Ott C, Williams J. Effectiveness of follow-up booster sessions in improving physical status after cardiac rehabilitation: health, behavioral and clinical outcomes. App Nurs Res. 2005;18:59–62							X								
Hofman-Bang C, Lisspers J, Nordiander R, et al.. Two-year results of a controlled study of residential rehabilitation for patients treated with percutaneous transluminal coronary angioplasty: a randomized study of a multifactorial program. Eur Heart J. 1999;20:1465–1474.							X								
Hsin-Hsin L, Yun-Fang T, Pyng-Jing L, Pei-Kwei T. Effects of a therapeutic lifestyle-change programme on cardiac risk factors after coronary artery bypass graft. J Clin Nurs. 2010;19:60–69.							X								
Lewin B, Robertson IH, Cay EL, Irving JB, Campbell M. Effects of self-help post myocardial infarction rehabilitation on psychological adjustment and use of health services. Lancet. 1992;339:1036–1040.							X								
Lewin RJP, Furze G, Robinson J, Griffith K, Wiseman S, Boyle R. A randomized controlled trial of a self-management plan for patients with newly diagnosed angina. Br J Gen Pract. 2001;52:194–201.							X								
Bolman, C. Vries, de, H., Breukelen, van, G. Evaluation of nurse managed minimal contact smoking cessation intervention for cardiac patients Health Education Research 17 99–116 2002b.									X						

For peer review only

Burt, A., Thornley, P., Illingworth, D., White, P., Shaw, T. R., Turner, R. Stopping smoking after myocardial infarction Lancet 1 304–306 1974.									X						
Feeney, G., McPherson, A., Connor, J., McAlister, A., Young, M., Garrahy, P. Randomized controlled trial of two cigarette quit programmes in coronary care patients after acute myocardial infarction Internal Medicine Journal 31 470–475 2001.									X				X	X	
Johnson, J. L., Budz, B., Mackay, M., Miller, M. Evaluation of a nurse-delivered smoking cessation intervention for hospitalized patients with cardiac disease Heart and Lung 28 55–64 1999.									X						
Mohiuddin, S. M., Mooss, A. N., Hunter, C. B., Grollmes, T. L., Cloutier, D. A., Hilleman, D. E. Intensive smoking cessation intervention reduces mortality in high-risk smokers with cardiovascular disease Chest 131 446–452 2007.									X						
Ockene, J., Kristeller, J. L., Goldberg, R., Ockene, I., Merriam, P., Barrett, S. Smoking cessation and severity of disease: The coronary artery smoking cessation intervention study Health Psychology 11 119–126 1992.									X						
Reid, R., Pipe, A., Higginson, L., Johnson, K., D'Angelo, M. S., Cooke, D., Dafoe, W. Stepped care approach to smoking cessation in patients hospitalized for coronary artery disease Journal of Cardiopulmonary Rehabilitation 23 176–182 2003.									X				X		
Barnason S. & Zimmerman L. (1995) A comparison of patient teaching outcomes among postoperative coronary artery bypass graft (CABG) patients. Progress in Cardiovascular Nursing 10(4), 11–20.										X					
Bengtsson K. (1983) Rehabilitation after myocardial infarction. A controlled study. Scandinavian Journal of Rehabilitation Medicine 15(1), 1–9.										X					X
Bolman C., Brug J., Bär F., Martinali J. & Van Den Borne B. (2005) Long-term efficacy of a checklist to improve patient education in cardiology. Patient Education and Counseling 56, 240–248.										X					
Cupples S.A. (1991) Effects of timing and reinforcement of preoperative education on knowledge and recovery of patients having coronary artery bypass graft surgery. Heart and Lung 20(6), 654–660.										X					
Furze G., Dumville J.C., Miles J.N.V., Irvine K., Thompson D.R. & Lewin R.J.P. (2009) "Prehabilitation" prior to CABG surgery improves physical functioning and depression. International Journal of Cardiology 132, 51–58.										X					
Martinali J., Bolman C., Brug J., Van Den Borne B. & Bar F. (2001) A checklist to improve patient education in a cardiology outpatient setting. Patient Education and										X					

For peer review only - http://bmjopen.bmj.com/site/about/guidelines.xhtml

For peer review only

Counseling 42, 231–238.															
Petrie K.J., Cameron L.D., Ellis C.J., Buick D. & Weinman J. (2002) Changing illness perceptions after myocardial infarction: an early intervention randomized controlled trial. Psychosomatic Medicine 64, 580–586.										X					
Pozen M.W., Stechmiller J.A., Harris W., Smith S., Fried D. & Voigt G.C. (1977) A nurse rehabilitator's impact on patients with myocardial infarction. Medical Care 15(10), 830–837.										X	X				
Tullman D.F., Haugh K.H., Dracup K.A. & Bourguignon C. (2007) A randomized controlled trial to reduce delay in older adults seeking help for symptoms of acute myocardial infarction. Research in Nursing & Health 30, 485–497.										X					
Eagle KA, Montoye CK, Riba AL, DeFranco AC, Parrish R, Skorcz S, Baker PL, Faul J, Jani SM, Chen B. Guideline-based standardized care is associated with substantially lower mortality in medicare patients with acute myocardial infarction: the American College of Cardiology's Guidelines Applied in Practice (GAP) projects in Michigan. J Am Coll Cardiol. 2005;46:1242–1248.											X				
Fonarow GC, Gawlinski A, Moughrabi S, Tillisch JH. Improved treatment of coronary heart disease by implementation of a Cardiac Hospitalization Atherosclerosis Management Program (CHAMP). Am J Cardiol. 2001;87:819–822.											X				
Oldenburg B, Perkins RJ, Andrews G. Controlled trial of psychological intervention in myocardial infarction. J Consult Clin Psychol. 1985;53:852–859											X				
Hedback B, Perk J. 5-year results of a comprehensive rehabilitation programme after myocardial infarction. Eur Heart J. 1987;8:234–242											X				
Maeland JG, Havik OE. The effects of an in-hospital educational programme for myocardial infarction patients. Scand J Rehabil Med. 1987;19:57–65.											X				
Fletcher V. An individualized teaching programme following primary uncomplicated myocardial infarction. J Adv Nurs. 1987;12:195–200											X				
Thompson DR. A randomized controlled trial of in-hospital nursing support for first time myocardial infarction patients and their partners: effects on anxiety and depression. J Adv Nurs. 1989;14:291–297.											X				
Fridlund B, Hogstedt B, Lidell E, Larsson PA. Recovery after myocardial infarction: effects of a caring rehabilitation programme. Scand J Caring Sci. 1991;5:23–32.											X				

For peer review only

Nordmann A, Heilmbauer I, Walker T, Martina B, Battegay E. A case-management program of medium intensity does not improve cardiovascular risk factor control in coronary artery disease patients: the Heartcare I Trial. Am J Med. 2001;110:543–550.											X				
Jeong HS, Chae JS, Moon JS, Yoo YS. An individualized teaching program for atherosclerotic risk factor reduction in patients with myocardial infarction. Yonsei Med J. 2002;43:93–100.											X				
Echeverry D, Dike M, Jovanovic L, Wollitzer AO, Westphal S, Mudaliar S, Washington C, Davidson MB. Efforts to improve subsequent treatment of cardiovascular risk factors in older patients with diabetes hospitalized for a cardiac event. Am J Manag Care. 2005;11:758–764.											X		X		
Scalzi CC, Burke LE, Greenland S. Evaluation of an inpatient educational program for coronary patients and families. Heart Lung. 1980;9:846–853.											X				
Kushnir B, Fox K, Tomlinson I, Aber CP. The effect of a pre-discharge consultation on the resumption of work, sexual activity, and driving following acute myocardial infarction. Scand J Rehabil Med. 1976;8:155–159.											X				
Young DT, Kottke TE, McCall MM, Blume D. A prospective controlled study of in-hospital myocardial infarction rehabilitation. J Card Rehabil. 1982;2:32–40.											X				
Holmboe ES, Meehan TP, Radford MJ, Wang Y, Marciniak TA, Krumholz HM. Use of critical pathways to improve the care of patients with acute myocardial infarction. Am J Med. 1999;107:324–331.											X				
Kucenic MJ, Meyers DG. Impact of a clinical pathway on the care and costs of myocardial infarction. Angiology. 2000;51:393–404.											X				
Cheah J. Clinical pathways: an evaluation of its impact on the quality of care in an acute care general hospital in Singapore. Singapore Med J. 2000;41:335–346.											X				
Hickey A, Scott I, Denaro C, Stewart N, Bennett C, Theile T. Using clinical indicators in a quality improvement programme targeting cardiac care. Int J Qual Health Care. 2004;16:i11–i25.											X				
Scott IA, Denaro CP, Hickey AC, Bennett C, Mudge AM, Sanders DC, Thiele J, Flores JL. Optimising care of acute coronary syndromes in three Australian hospitals. Int J Qual Health Care. 2004;16:275–284.											X				
Lappe JM, Muhlestein JB, Lappe DL, Badger RS, Bair TL, Brockman R, French TK, Hofmann LC, Horne BD, Kralick-Goldberg S, Nicponski N, Orton JA, Pearson RR, Renlund DG, Rimmasch H, Roberts C, Anderson JL. Improvements in 1-year cardiovascular clinical outcomes associated with a hospital-based discharge medication											X				

For peer review only

program. Ann Intern Med. 2004;141:446–453.															
Grace C, Summerbell C. Does provision of additional dietary information affect actual or only reported compliance to a low-fat diet over 12 weeks in hyperlipidaemic individuals? Report of a pilot study. J Hum Nutr Diet 1996; 9: 303–7.													X		
Dalgård C, Thurøe A, Haastrup B, Haghfelt T, Stender S. Saturated fat intake is reduced in patients with ischemic heart disease 1 year after comprehensive counseling but not after brief counseling. J Am Diet Assoc 2001; 101: 1420–9.													X		
Levetan CS, Dawn KR, Murray JF, Popma JJ, Ratner RE, Robbins DC. Impact of computer-generated personalized goals on cholesterol lowering. Value Health 2005; 8: 639–46.													X		
Bolman CVH, van Breukelen G. Evaluation of a nurse-managed minimal-contact smoking cessation intervention for cardiac inpatients. Health Educ Res 2002; 17: 99–116.													X		
Sivarajan Froelicher ESS, Miller NH, Christoperson DJ et al. High rates of sustained smoking cessation in women hospitalized with cardiovascular disease: the Women's Initiative for Nonsmoking(WINS). Circulation 2004; 109: 587–93													X		
Feder G, Griffiths C, Eldridge S, Spence M. Effect of postal prompts to patients and general practitioners on the quality of primary care after a coronary event (POST): randomised controlled trial. Br Med J 1999; 318: 1522.													X		
Mahler HI, Kulik JA, Tarazi RY. Effects of a videotape information intervention at discharge on diet and exercise compliance after coronary bypass surgery. J Cardiopulm Rehabil 1999; 19:170–7.													X		
Allen JK: Coronary risk factor modification in woman after coronary artery by pass surgery.Nursing Research. 1996,45:260–265.															X
Carlsson R, Lindberg G, Westin L, Israelsson B: Influence of coronary nursing management follow up on lifestyle after acute myocardial infarction.Heart. 1997,77:256–259.															X
DeBusk RF, Miller N, Superko HR, et al.: Acase-management system for coronary risk factor modification after acute myocardial infarction.Annals of Internal Medicine. 1994,120:721–729.															X
Lisspers J, Sundin O, Hofman-Bang C, et al.: Behavioral effects of a comprehensive, multifactorial program for lifestyle change after percutaneous transluminal coronary angioplasty: A prospective, randomized, controlled study.Journal of Psychosomatic Research. 1999,46:143–154.															X
Mitsibounas DN, Tsouna-Hadjis ED, Sideris DA, Rotas															X

For peer review only

AR: Effects of group psychsocial intervention on coronary risk factors.Psychotherapy and Psychosomatics. 1992,58:97–102.															
Campbell NC, Thain J, Deans HG, Ritchie LD, Rawles JM, Squair JL. Secondary prevention clinics for coronary heart disease: randomised trial of effect on health. BMJ. 1998;316:1434-7.															X
Campbell NC, Ritchie LD, Thain J, Deans HG, Rawles JM, Squair JL. Secondary prevention in coronary heart disease: a randomised trial of nurse led clinics in primary care. Heart. 1998;80:447-52															X
Engblom E, Rönnemaa T, Hämäläinen H, Kallio V, Vänttinen E, Knuts LR. Coronary heart disease risk factors before and after bypass surgery: results of a controlled trial on multifactorial rehabilitation. Eur Heart J. 1992;13:232-7.															X
Taylor CB, Miller NH, Smith PM, DeBusk RF. The effect of a home-based, case-managed, multifactorial risk-reduction program on reducing psychological distress in patients with cardiovascular disease. J Cardiopulm Rehabil. 1997;17:157-62.															X
Murchie P, Campbell NC, Ritchie LD, Simpson JA, Thain J. Secondary prevention clinics for coronary heart disease: four year follow up of a randomised controlled trial in primary care. BMJ. 2003;326:84															X
Schuler G, Hambrecht R, Schlierf G, Niebauer J, Hauer K, Neumann J, et al. Regular physical exercise and low-fat diet. Effects on progression of coronary artery disease. Circulation. 1992;86:1-11.a															X
Niebauer J, Hambrecht R, Velich T, Hauer K, Marburger C, Kälberer B, et al. Attenuated progression of coronary artery disease after 6 years of multifactorial risk intervention: role of physical exercise. Circulation. 1997;96:2534-41.															X
Ornish D, Brown SE, Scherwitz LW, Billings JH, Armstrong WT, Ports TA, et al. Can lifestyle changes reverse coronary heart disease? The Lifestyle Heart Trial. Lancet. 1990;336:129-33															X
Ornish D, Scherwitz LW, Billings JH, Brown SE, Gould KL, Merritt TA, et al. Intensive lifestyle changes for reversal of coronary heart disease. JAMA. 1998;280:2001-7.															X
Naylor MD, Brooten D, Campbell R, Jacobsen BS, Mezey MD, Pauly MV, et al. Comprehensive discharge planning and home follow-up of hospitalized elders: a randomized clinical trial. JAMA. 1999;281:613-20.															X
Naylor MD, McCauley KM. The effects of a discharge planning and home follow-up intervention on elders hospitalized with common medical and surgical cardiac conditions. J Cardiovasc Nurs. 1999;14:44-54.															X
Dorossiev D. Rehabilitation and Comprehensive Secondary Prevention after Acute Myocardial Infarction. Report on a Study. EURO Reports and Studies No. 84. Copenhagen: World Health Organization; 1983.															X

For peer review only

For peer review only - http://bmjopen.bmj.com/site/about/guidelines.xhtml

Kallio V, Hämäläinen H, Hakkila J, Luurila OJ. Reduction in sudden deaths by a multifactorial intervention programme after acute myocardial infarction. Lancet. 1979;2:1091-4															X
Hämäläinen H, Luurila OJ, Kallio V, Knuts LR, Arstila M, Hakkila J. Long-term reduction in sudden deaths after a multifactorial intervention programme in patients with myocardial infarction: 10-year results of a controlled investigation. Eur Heart J. 1989;10:55-62															X
Hämäläinen H, Luurila OJ, Kallio V, Knuts LR. Reduction in sudden deaths and coronary mortality in myocardial infarction patients after rehabilitation. 15 year follow-up study. Eur Heart J. 1995;16:1839-44.															X
Sivarajan ES, Bruce RA, Lindskog BD, Almes MJ, Belanger L, Green B. Treadmill test responses to an early exercise program after myocardial infarction: a randomized study. Circulation. 1982;65:1420-8.															X
Vermeulen A, Lie KI, Durrer D. Effects of cardiac rehabilitation after myocardial infarction: changes in coronary risk factors and long-term prognosis. Am Heart J. 1983;105:798-801															X
Bell JM. A Comparison of a Multi-Disciplinary Home Based Cardiac Rehabilitation Programme with Comprehensive Conventional Rehabilitation in Post-Myocardial Infarction Patients [PhD thesis]. London: University of London; 1998.															X
Toobert DJ, Glasgow RE, Radcliffe JL. Physiologic and related behavioral outcomes from the Women's Lifestyle Heart Trial. Ann Behav Med. 2000;22:1-9.															X
Sundin O, Lisspers J, Hofman-Bang C, Nygren A, Rydén L, Ohman A. Comparing multifactorial lifestyle interventions and stress management in coronary risk reduction. Int J Behav Med. 2003;10:191-204.															X
Yu CM, Li LS, Ho HH, Lau CP. Long-term changes in exercise capacity, quality of life, body anthropometry, and lipid profiles after a cardiac rehabilitation program in obese patients with coronary heart disease. Am J Cardiol. 2003;91:321-5.															X
Marchionni N, Fattirolli F, Fumagalli S, Oldridge N, Del Lungo F, Morosi L, et al. Improved exercise tolerance and quality of life with cardiac rehabilitation of older patients after myocardial infarction: results of a randomized, controlled trial. Circulation. 2003;107:2201-6.															X
Masley S, Phillips S, Copeland JR. Group office visits change dietary habits of patients with coronary artery disease—the dietary intervention and evaluation trial (D.I.E.T.). J Fam Pract. 2001;50:235-9.															X
Ornish D, Scherwitz LW, Doody RS, Kesten D, McLanahan SM, Brown SE, et al. Effects of stress management training and dietary changes in treating ischemic heart disease. JAMA. 1983;249:54-9.															X
Fitzgerald JF, Smith DM, Martin DK, Freedman JA, Katz															X

For peer review only

BP. A case manager intervention to reduce readmissions. Arch Intern Med. 1994;154:1721-9.															
Naylor M, Brooten D, Jones R, Lavizzo-Mourey R, Mezey M, Pauly M. Comprehensive discharge planning for the hospitalized elderly. A randomized clinical trial. Ann Intern Med. 1994;120:999-1006															X
Carlsson R. Serum cholesterol, lifestyle, working capacity and quality of life in patients with coronary artery disease. Experiences from a hospital-based secondary prevention programme. Scand Cardiovasc J Suppl. 1998;50:1-20															X
Jolly K, Bradley F, Sharp S, Smith H, Thompson S, Kinmonth AL, et al. Randomised controlled trial of follow up care in general practice of patients with myocardial infarction and angina: final results of the Southampton heart integrated care project (SHIP). The SHIP Collaborative Group. BMJ. 1999;318:706-11.															X
Allison TG, Farkouh ME, Smars PA, Evans RW, Squires RW, Gabriel SE, et al. Management of coronary risk factors by registered nurses versus usual care in patients with unstable angina pectoris (a chest pain evaluation in the emergency room [CHEER] substudy). Am J Cardiol. 2000;86:133-8															X
Moher M, Yudkin P, Wright L, Turner R, Fuller A, Schofield T, et al. Cluster randomised controlled trial to compare three methods of promoting secondary prevention of coronary heart disease in primary care. BMJ. 2001;322:1338.															X
Higgins HC, Hayes RL, McKenna KT. Rehabilitation outcomes following percutaneous coronary interventions (PCI). Patient Educ Couns. 2001;43:219-30.															X
Allen JK, Blumenthal RS, Margolis S, Young DR, Miller ER 3rd, Kelly K. Nurse case management of hypercholesterolemia in patients with coronary heart disease: results of a randomized clinical trial. Am Heart J. 2002;144:678-86															X
Lear SA, Ignaszewski A, Linden W, Brozic A, Kiess M, Spinelli JJ, et al. The Extensive Lifestyle Management Intervention (ELMI) following cardiac rehabilitation trial. Eur Heart J. 2003;24:1920-7.															X
Young W, Rewa G, Goodman SG, Jaglal SB, Cash L, Lefkowitz C, et al. Evaluation of a community-based inner-city disease management program for postmyocardial infarction patients: a randomized controlled trial. CMAJ. 2003;169:905-10.															X
Lichtman JH, Amatruda J, Yaari S, Cheng S, Smith GL, Mattera JA, et al. Clinical trial of an educational intervention to achieve recommended cholesterol levels in patients with coronary artery disease. Am Heart J. 2004;147:522-8.															X
Haskell WL, Alderman EL, Fair JM, Maron DJ, Mackey SF, Superko HR, et al. Effects of intensive multiple risk factor reduction on coronary atherosclerosis and clinical cardiac events in men and women with coronary artery disease. The Stanford Coronary Risk Intervention Project															X

For peer review only

(SCRIP). Circulation. 1994;89:975-90.															
Allison TG, Squires RW, Johnson BD, Gau GT. Achieving National Cholesterol Education Program goals for low-density lipoprotein cholesterol in cardiac patients: importance of diet, exercise, weight control, and drug therapy. Mayo Clin Proc. 1999;74:466-73															X
Stagmo M, Westin L, Carlsson R, Israelsson B. Long-term effects on cholesterol levels and the utilization of lipid-lowering drugs of a hospital-based programme for secondary prevention of coronary artery disease. J Cardiovasc Risk. 2001;8:243-8.															X
Wilhelmsen L, Sanne H, Elmfeldt D, Grimby G, Tibblin G, Wedel H. A controlled trial of physical training after myocardial infarction. Effects on risk factors, nonfatal reinfarction, and death. Prev Med. 1975;4:491-508.															X
Shaw LW. Effects of a prescribed supervised exercise program on mortality and cardiovascular morbidity in patients after myocardial infarction. The National Exercise and Heart Disease Project. Am J Cardiol. 1981;48:39-46															X
Carson P, Phillips R, Lloyd M, Tucker H, Neophytou M, Buch NJ, et al. Exercise after myocardial infarction: a controlled trial. J R Coll Physicians Lond. 1982;16:147-51.															X
Ballantyne FC, Clark RS, Simpson HS, Ballantyne D. The effect of moderate physical exercise on the plasma lipoprotein subfractions of male survivors of myocardial infarction. Circulation. 1982;65:913-8.															X
Miller NH, Haskell WL, Berra K, DeBusk RF. Home versus group exercise training for increasing functional capacity after myocardial infarction. Circulation. 1984;70:645-9.															X
Roviaro S, Holmes DS, Holmsten RD. Influence of a cardiac rehabilitation program on the cardiovascular, psychological, and social functioning of cardiac patients. J Behav Med. 1984;7:61-81.															X
Erdman RA, Duivenvoorden HJ, Verhage F, Kazemier M, Hugenholtz PG. Predictability of beneficial effects in cardiac rehabilitation: a randomized clinical trial of psychosocial variables. J Cardiopulm Rehabil. 1986;6:206-13.															X
Agren B, Olin C, Castenfors J, Nilsson-Ehle P. Improvements of the lipoprotein profile after coronary bypass surgery: additional effects of an exercise training program. Eur Heart J. 1989;10:451-8.															X
Bethell HJ, Mullee MA. A controlled trial of community based coronary rehabilitation. Br Heart J. 1990;64:370-5															X
Bertie J, King A, Reed N, Marshall AJ, Ricketts C. Benefits and weaknesses of a cardiac rehabilitation programme. J R Coll Physicians Lond. 1992;26:147-51.															X
Fletcher BJ, Dunbar SB, Felner JM, Jensen BE, Almon L, Cotsonis G, et al. Exercise testing and training in physically disabled men with clinical evidence of coronary artery disease. Am J Cardiol. 1994;73:170-4															X
Holmbäck AM, Säwe U, Fagher B. Training after myocardial infarction: lack of long-term effects on physical capacity and psychological variables. Arch Phys Med															X

For peer review only

Rehabil. 1994;75:551-4.															
Wosornu D, Bedford D, Ballantyne D. A comparison of the effects of strength and aerobic exercise training on exercise capacity and lipids after coronary artery bypass surgery. Eur Heart J. 1996;17:854-63.															x
Heldal M, Sire S, Dale J. Randomised training after myocardial infarction: short and long-term effects of exercise training after myocardial infarction in patients on beta-blocker treatment. A randomized, controlled study. Scand Cardiovasc J. 2000;34:59-64.															x
Ståhle A, Lindquist I, Mattsson E. Important factors for physical activity among elderly patients one year after an acute myocardial infarction. Scand J Rehabil Med. 2000;32:111-6.															x
Belardinelli R, Paolini I, Cianci G, Piva R, Georgiou D, Purcaro A. Exercise training intervention after coronary angioplasty: the ETICA trial. J Am Coll Cardiol. 2001;37:1891-900.															x
	16	20	42	26	24	62	7	13	14	13	27	18	17	19	74

ACS = acute coronary syndrome;

% overlap 63/286= 22.0%

CA= Covered Area = (16+20+42+26+24+62+7+13+14+13+27+18+17+19+74)/ (15x286) = 392/4290=9.1%

CCA= Corrected Covered Area = (392-286)/ (4290-286) = 106/4004= 2.6% (slight)

For peer review only

Supplementary Annex 3.

Citation Matrix: Non-repeated trials (rows) included in the 36 systematic review and/or meta-analyses (column)--T2DM.

Author-year (Trial reference)	1	2	3	4	5	6	7	8	9	10	11	12	13	14	15	16	17	18	19	20	21	22	23	24	25	26	27	28	29	30	31	32	33	34	35	36
K. Cai, S. Zhang, M. Lu, L. Shao The effects of intensive education for elderly patients with diabetes mellitus. Pract Geriatr, 18 (2004), pp. 313–315	X																																			
Y. Chen, W. Xue, S. Lin, S. Huang, D. Li. ntensive diabetes education in the role of diabetes control. J Sun Yat-sen Univ (Med Sci), 3S (2004), pp. 201–203	X																																			
L. Chen, M. Zhu. Clinical investigation of the effect of type 2 diabetes health education. Strait Pharm J, 21 (2009), pp. 217–218	X																																			
Y. Fang. Coordinated health education model in diabetes education. Today Nurse, 3 (2011), pp. 148–149	X																																			
B. Li, Y. Liu, J. Tian, J. Yuan. Impact of diabetes education for type 2 diabetes on glycated haemoglobin and health cost.Jiangsu J Prev Med, 23 (2012), pp. 55–56	X																																			
B.-h. Li, F.-h. Zhang, C. Zhang, M. Yang Effect of different education models on blood glucose in diabetic patients J Qilu Nurs, 19 (2013), pp. 26–27	X																																			
M. Li, T. Li, B.-y. Shi, C.-x. Gao Effect of motivational interviewing on the quality of life in type 2 diabetes patients with poor control of blood glucose Chin J Nurs, 49 (2014), pp. 6–10	X																																			
X. Li, Q. Zhou, F. Zou, L. Wu, H. Chen, Z. Liu Effectiveness of systematic self-management education on blood sugar level of patients in the community with type 2 diabetes J Central South Univ (Med Sci), 37 (2012), pp. 355–357	X																																			
Y. Li Evaluation of the standardized management effect of diabetes in community Chin Prim Health Care, 25 (2011), pp. 33–35	X																																			
Q. Liu, X. Pan, W. Feng, J. Kong Effectiveness of health education to the patients with diabetes mellitus J Nurs Sci, 20 (2005), pp. 54–55	X																																			
Z.-p. Liu, L.-l. Han, J.-r. Deng, X.-r. Liu, X.-r. Zhang, Q.-f. Li Effect of short-term intensified education on glucose	X																																			

For peer review only

control of type 2 diabetic patients<br>Chin J Nurs Educ, 4 (2007), pp. 249–251																																				
L.-m. Luo, K.-J. Yang, Z.-J. Zhang, Y.-H. Li, C.-H. Hu, X.-L. Feng<br>Role of health education on diabetic community chronic disease management<br>China Pharm, 24 (2013), pp. 3020–3022	X																																			
Y. Lu<br>Observation on the effect of diabetes intensive education in type 2 diabetes mellitus patients<br>Med Inform, 21 (2008), pp. 690–692	X																																			
X. Mei<br>Analysis of compliance and effectiveness of community diabetes education program<br>J Nanchang Univ (Med Sci), 50 (2010), pp. 83–84	X																																			
G. Pang<br>The impact of intensive diabetes education on community patients<br>J Med Theory Pract, 21 (2008), pp. 839–840	X																																			
Y. Qi, C. Chen, L. Yan<br>Impact of cooking class on diabetic patients eating habits<br>Chin J Nurs, 41 (2006), pp. 126–127	X																																			
B. Sun, B. Ban, H.-l. Sun, S. Zhang, S. Jie<br>Effects of diabetes education and psychological intervention on comprehensive treatment of type 2 diabetes<br>Chin J Clin Psychol, 13 (2005), pp. 483–485	X																						X													
M. Sun, X. Li, F. Wang, D. Zhu<br>Study on the effect of diabetes education<br>J Pract Diagn Ther, 19 (2005), pp. 422–423	X																																			
Z. Sun, M. Lv<br>90 cases of individualised type 2 diabetes education<br>J Qilu Nurs, 14 (2008), pp. 106–107	X																																			
H. Wang, S. Hu<br>Comparison of clinical applicability of individual intervention with intensive group education for diabetic patients<br>People's Mil Surg, 53 (2009), pp. 234–235	X																																			
X. Wang, H. Zou, H. Jie, S. Shen, J. Zhang<br>Effect of individualised diabetes education and care for aged patients with diabetes<br>Chin J Clin Res, 26 (2013), pp. 90–91	X																																			
Y. Wei<br>Effectiveness of intensive education for patients with diabetes mellitus<br>Chin J Health Educ, 21 (2005), pp. 53–55	X																																			
S.-q. Wu, G.-x. Jin, Y.-f. Chen	X																																			

For peer review only

Effect observation of sample diet education on diet control of patients with diabetes<br>J Nurs (China), 13 (2006), pp. 17–19																																				
Y. Wu, K. Wang, X. Yang<br>The effect of diabetes self-management education curriculum for patients with diabetes<br>Guide China Med, 11 (2013), pp. 395–397	X																																			
X.-L. Xiao, Q.-Q. Hu, Y.-P. Liu<br>Effects of family involvement in health education for patients with type 2 diabetes<br>Chin J Nurs, 47 (2012), pp. 313–316	X																																			
B. Xie, C.-p. Ju, Z.-l. Sun, J. Han, L. Liu<br>Effect of education network for diabetes self-management on self-efficacy in patients with type 2 diabetes mellitus<br>Chin Gen Pract, 15 (2012), pp. 3610–3616	X																																			
H. Xie, C. Cheng, D. Chen<br>Intensive diabetes education for poorly controlled elderly diabetic patients in the community<br>J Pract Med Tech, 14 (2007), pp. 3958–3959	X																																			
S. Xu<br>Effect of continued outpatient health education on diabetes patients<br>Mod Clin Nurs, 6 (2007), pp. 28–30	X																																			
L. Yan, C. Zhu, X. Tan<br>“Diabetes conversation” map interactive education for diabetic patients<br>Chin J Misdiagn, 11 (2011), pp. 6383–6384	X																																			
X. Yan, N. Li, H. Liu, H. Sha, P. Li<br>Effect of “interactive groups” diabetes education model on diabetes patients in the village<br>Nurs Pract Res, 9 (2012), pp. 13–14	X																																			
S. Yao, Y. Liu, R. Ji, G. Wang<br>Effectiveness of diabetes education for patients with type 2 diabetes<br>Hebei J Tradit Chin Med, 34 (2012), pp. 1723–1724	X																																			
W. Yu, Y.-l. Bi, J. Han, et al.<br>Affect of the MUST education mode on compliance of patients with type 2 diabetes mellitus<br>J Shandong Univ (Health Sci), 49 (2011), pp. 1–4	X																																			
Y. Zeng, A. Wang, M. Gong<br>Clinical observation of communication management targeted at patients with type 2 diabetes<br>J Pract Diabetol, 5 (2009), pp. 48–49	X																																			
F. Zhang, B. Xu<br>Influence of diabetes education on patients lifestyle and metabolic indicators	X																																			

For peer review only

Mod Med J China, 12 (2010), pp. 116–117																																				
J. Zhang, Y. Zhang, X. Huang<br>“Diabetes conversation” map interactive education for type 2 diabetes patients<br>Guide China Med, 9 (2011), pp. 330–331	X																																			
L. Zhang<br>Analysis on the therapeutic effects of diabetes education<br>Tianjin J Tradit Chin Med, 26 (2009), pp. 22–23	X																																			
X.-q. Zhang, L.-f. Fan, Y.-g. Zheng<br>Effect of health education applied in promoting compliance of diet treatment of patients with diabetes mellitus<br>Mod Nurs, 12 (2006), pp. 1082–1084	X																																			
X. Zheng, H. Tang, Y. Huang<br>Strengthen health education on observed efficacy in patients with type 2 diabetes<br>Youjiang Med J, 33 (2005), pp. 349–350	X																																			
C. Zhong, J. Li, X. Yang<br>Effect of interactive diabetes education for patients with diabetes<br>Jilin Med J, 27 (2006), pp. 1490–1491	X																																			
A. Zhou, Y. Ying, X. Dai, et al.<br>Application of text message to diabetic education<br>J Nurs Sci, 27 (2012), pp. 17–19	X																																			
M.-l. Gu, X.-g. Zhu, X.-f. Xu<br>The study of comprehensive intervention for patients with type 2 diabetes in community of Shanghai<br>Chin Gen Pract, 15 (2012), pp. 107–109	X																																			
Z.-M. Huang<br>Impact of intensive diabetes education targeted at patients with type 2 diabetes<br>Shanghai J Prev Med, 21 (2009), pp. 323–324	X																																			
C. Liu, W. Sun, J. Wang, J. Liu<br>Effect of community management of type 2 diabetic patients<br>Chin J Prev Control Chronic Dis, 20 (2012), pp. 624–625	X																																			
J. Sun, Y. Wang, X. Chen, et al.<br>An integrated intervention program to control diabetes in overweight Chinese women and men with type 2 diabetes<br>Asia Pac J Clin Nutr, 17 (2008), pp. 514–524	X																																			
X. Wang, X. Zhou, H. Zhen, J. Sun, C. Cui<br>Impact of stratified intensive education for patients with diabetes<br>J Med Theory Pract, 18 (2005), pp. 336–337	X																																			
X. Wang, X. Zhou, H. Zhen, J. Sun, C. Cui	X																																			

For peer review only

Impact of stratified intensive education for patients with diabetes<br>J Med Theory Pract, 18 (2005), pp. 336–337																																				
B. Yang, C. Ma, X. Feng, et al.<br>Effect of face-to-face education on metabolic improvement in Tibetan diabetic patients<br>J Clin Rehabil Tissue Eng Res, 11 (2007), pp. 10467–10470	X																																			
G.T. Ko, J.K. Li, E.C. Kan, M.K. Lo<br>Effects of a structured health education programme by a diabetic education nurse on cardiovascular risk factors in Chinese type 2 diabetic patients: a 1-year prospective randomized study<br>Diabet Med, 21 (2004), pp. 1274–1279	X		X	X																		X		X												
A. Lee, C.F. Siu, K.T. Leung, L.C. Lau, C.C. Chan, K.W. Kwok<br>General practice and social service partnership for better clinical outcomes, patient self efficacy and lifestyle behaviours of diabetic care: randomised control trial of a chronic care model Postgrad Med J, 87 (2011), pp. 688–693	X				X																															
S.M. Chen, D. Creedy, H.S. Lin, J. Wollin<br>Effects of motivational interviewing intervention on self-management, psychological and glycemic outcomes in type 2 diabetes: a randomized controlled trial<br>Int J Nurs Stud, 49 (2012), pp. 637–644	X																																			
M.C. Huang, C.C. Hsu, H.S. Wang, S.J. Shin<br>Prospective randomized controlled trial to evaluate effectiveness of registered dietitian-led diabetes management on glycemic and diet control in a primary care setting in Taiwan<br>Diabetes Care, 33 (2010), pp. 233–239	X																																			
M.C. Huang, C.C. Hsu, H.S. Wang, S.J. Shin<br>Prospective randomized controlled trial to evaluate effectiveness of registered dietitian-led diabetes management on glycemic and diet control in a primary care setting in Taiwan<br>Diabetes Care, 33 (2010), pp. 233–239	X																																			
M. Jian, L. Liu, H.-c. Chiang<br>Building a certificate-based secure and quick response telemedical health-care system for diabetic patients<br>J Intern Med Taiwan, 17 (2006), pp. 61–72	X																																			
T.I. Lee, Y.T. Yeh, C.T. Liu, P.L. Chen<br>Development and evaluation of a patient-oriented education system for diabetes management<br>Int J Med Inform, 76 (2007), pp. 655–663	X																			X																
Agurs-Collins TD, Kumanyika SK, Ten Have TR, Adams-Campbell LL. A randomized controlled trial of weight reduction and exercise for diabetes management in		X			X		X			X		X				X					X	X				X				X				X	X	X

older African-American subjects. Diabetes Care 1997; 20: 1503–1511.																																				
Anderson RM, Funnell MM, Nwankwo R, Gillard ML, Oh M, Fitzgerald JT. Evaluating a problem-based empowerment program for African Americans with diabetes: results of a randomized controlled trial. Ethn Dis 2005; 15: 671–678.		X			X		X			X						X					X	X				X										
Babamoto KS, Sey KA, Camilleri AJ, Karlan VJ, Catalasan J, Morisky DE. Improving diabetes care and health measures among hispanics using community health workers: results from a randomized controlled trial. Health Educ Behav 2009; 36: 113–126.		X					X			X																										
Baradaran HR, Knill-Jones RP, Wallia S, Rodgers A. A controlled trial of the effectiveness of a diabetes education programme in a multi-ethnic community in Glasgow [ISRCTN28317455]. BMC Public Health 2006; 18: 134.		X					X			X							X				X					X	X									
Bellary S, O'Hare JP, Raymond NT, Gumber A, Mughal S, Szczepura A et al. Enhanced diabetes care to patients of south Asian ethnic origin (the United Kingdom Asian Diabetes Study): a cluster randomised controlled trial. Lancet 2008; 371: 1769–1776.		X					X														X															
Brown SA, Garcia AA, Kouzekanani K, Hanis CL. Culturally competent diabetes self-management education for Mexican Americans: the Starr County border health initiative. Diabetes Care 2002; 25: 259–268.		X			X		X			X						X	X				X	X				X		X				X				
Carter EL, Nunlee-Bland G, Callender C. A patient-centric, provider-assisted diabetes telehealth self-management intervention for urban minorities. Perspect Health Inf Manag: Winter 2011.		X					X																													
Crowley MJ, Powers BJ, Olsen MK, Grubber JM, Koropchak C, Rose CM et al. The Cholesterol, Hypertension, And Glucose Education (CHANGE) study: results from a randomized controlled trial in African Americans with diabetes. Am Heart J 2013; 166: 179–186.		X					X																													
D'Eramo Melkus G, Chyun D, Vorderstrasse A, Newlin K, Jefferson V, Langerman S. The effect of a diabetes education, coping skills training, and care intervention on physiological and psychosocial outcomes in black women with type 2 diabetes. Biol Res Nurs 2010; 12:7–19.		X			X		X																													
DePue JD, Dunsiger S, Seiden AD, Blume J, Rosen RK, Goldstein MG. Nurse-community health worker team improves diabetes care in American Samoa: results of a randomized controlled trial. Diabetes Care 2013; 36: 1947–1953.		X					X																													
Gary TL, Batts-Turner M, Yeh HC, Hill-Briggs F, Bone LR, Wang NY et al. The effects of a nurse case manager		X					X																													

For peer review only

and a community health worker team on diabetic control, emergency department visits, and hospitalizations among urban African Americans with type 2 diabetes mellitus: a randomized controlled trial. Arch Intern Med 2009; 169: 1788–1794.																																				
Gucciardi E, Demelo M, Lee RN, Grace SL. Assessment of two culturally competent diabetes education methods: individual versus individual plus group education in Canadian Portuguese adults with type 2 diabetes. Ethn Health 2007; 12: 163–187.		X					X									X										X										
Hawthorne K, Tomlinson S. One-to-one teaching with pictures–flashcard health education for British Asians with diabetes. Br J Gen Pract 1997; 47: 301–304.		X					X			X						X					X			X		X	X							X		X
Kattelmann KK, Conti K, Ren C. The medicine wheel nutrition intervention: a diabetes education study with the Cheyenne River Sioux Tribe. J Am Diet Assoc 2009; 109: 1532–1539.		X					X																													
Keyserling TC, Samuel-Hodge CD, Ammerman AS, Ainsworth BE, Henríquez-Roldán CF, Elasy TA et al. A randomized trial of an intervention to improve self-care behaviors of African-American women with type 2 diabetes: impact on physical activity. Diabetes Care 2002; 25: 1576–1583.		X			X		X			X		X										X														
Khan MA, Shah S, Grudzien A, Onyejekwe N, Banskota P, Karim S et al. A diabetes education multimedia program in the waiting room setting. Diabetes Therapy 2011; 2: 178–188.		X					X																													
Kim MT, Han HR, Song HJ, Lee JE, Kim J, Ryu JP et al. A community-based, culturally tailored behavioral intervention for Korean Americans with type 2 diabetes. Diabetes Educator 2009; 35: 986–944.		X			X		X									X																				
Lorig K, Ritter PL, Villa F, Piette JD. Spanish diabetes self-management with and without automated telephone reinforcement: two randomized trials. Diabetes Care 2008; 31: 408–414.		X			X		X																													
Lujan J, Ostwald SK, Ortiz M. Promotora diabetes intervention for Mexican Americans. Diabetes Educator 2007; 33: 660–670.		X			X		X			X							X																			
Middelkoop BJ, Geelhoed-Duijvestijn PH, van der Wal G. Effectiveness of culture-specific diabetes care for Surinam South Asian patients in the Hague: a randomized controlled trial/controlled before-and-after study. Diabetes Care 2001; 24: 1997–1998		X					X			X						X					X					X	X									
O'Hare JP, Raymond NT, Mughal S, Dodd L, Hanif W, Ahmad Y et al. Evaluation of delivery of enhanced diabetes care to patients of South Asian ethnicity: the United Kingdom Asian Diabetes Study (UKADS).		X					X									X					X					X	X									

Diabetic Med 2004; 221: 1357–1365.																																				
Osborn CY, Amico KR, Cruz N, O'Connell AA, Perez-Escamilla R, Kalichman SC et al. A brief culturally tailored intervention for Puerto Ricans with type 2 diabetes. Health Educ Behav 2010; 37: 849–862.		X					X																													
Philis-Tsimikas A, Fortmann A, Lleva-Ocana L, Walker C, Gallo LC. Peer-led diabetes education programs in high-risk Mexican Americans improve glycemic control compared with standard approaches: a Project Dulce promotora randomized trial. Diabetes Care 2011; 34: 1926–1931		X			X		X			X																										
Rosal MC, Olendzki B, Reed GW, Gumieniak O, Scavron J, Ockene I. Diabetes self-management among low-income Spanish-speaking patients: a pilot study. Ann Behav Med 2005; 29: 225–235.		X			X		X			X						X	X				X	X				X										
Rothschild SK, Martin MA, Swider SM, Tumialán Lynas CM, Janssen I, Avery EF et al. Mexican American trial of community health workers: a randomized controlled trial of a community health worker intervention for Mexican Americans with type 2 diabetes mellitus. Am J Public Health 2014; 104: 1540–1548.		X			X		X																													
Samuel-Hodge CD, Keyserling TC, Park S, Johnston LF, Gizlice Z, Bangdiwala SI. A randomized trial of a church-based diabetes self-management program for African Americans with type 2 diabetes. Diabetes Educator 2009; 35: 439–453.		X			X		X																													
Sixta CS, Ostwald S. Texas-Mexico border intervention by promotores for patients with type 2 diabetes. Diabetes Educ 2008; 34: 299–309.		X			X		X																													
Skelly AH, Carlson JR, Leeman J, Holditch-Davis D, Soward AC. Symptom-focused management for African American women with Type 2 diabetes: a pilot study. Appl Nurs Res 2005; 18: 213–220.		X			X		X					X										X														
Skelly AH, Carlson J, Leeman J, Soward A, Burns D. Controlled trial of nursing interventions to improve health outcomes of older African American women with type 2 diabetes. Nurs Res 2009; 58: 410–418.		X			X		X			X																										
Spencer MS, Rosland AM, Kieffer EC, Sinco BR, Valerio M, Palmisano G et al. Effectiveness of a community health worker intervention among African American and Latino adults with type 2 diabetes: a randomized controlled trial. Am J Public Health 2011; 101: 2253–2260.		X			X		X																													
Toobert DJ, Strycker LA, Barrera M Jr, Osuna D, King DK, Glasgow RE. Outcomes from a multiple risk factor diabetes self-management trial for Latinas: ¡Viva Bien!. Ann Behav Med 2011; 41: 310–323.		X																																		

Vincent D, Pasvogel A, Barrera L. A feasibility study of a culturally tailored diabetes intervention for Mexican Americans. Biol Res Nurs 2007; 9: 130–141.		X			X					X						X																				
A.R.G. Look, R.R. Wing Long-term effects of a lifestyle intervention on weight and cardiovascular risk factors in individuals with type 2 diabetes mellitus: four-year results of the Look AHEAD trial. Arch Intern Med, 170 (2010), pp. 1566–1575			X	X																																
D.A. Dobrosielski, B.B. Gibbs, P. Ouyang, S. Bonekamp, J.M. Clark, N.Y. Wang, et al. Effect of exercise on blood pressure in type 2 diabetes: a randomized controlled trial J Gen Intern Med, 27 (2012), pp. 1453–1459			X	X																																
W. Wisse, M. Boer Rookhuizen, M.D. de Kruif, J. van Rossum, I. Jordans, H. ten Cate, et al. Prescription of physical activity is not sufficient to change sedentary behavior and improve glycemic control in type 2 diabetes patients Diabetes Res Clin Pract, 88 (2010), pp. e10–e13			X	X																																
S. Balducci, S. Zanuso, A. Nicolucci, P. De Feo, S. Cavallo, P. Cardelli, et al. Effect of an intensive exercise intervention strategy on modifiable cardiovascular risk factors in subjects with type 2 diabetes mellitus: a randomized controlled trial: the Italian Diabetes and Exercise Study (IDES) Arch Intern Med, 170 (2010), pp. 1794–1803			X	X																																
A. Kirk, N. Mutrie, P. MacIntyre, M. Fisher Effects of a 12-month physical activity counselling intervention on glycaemic control and on the status of cardiovascular risk factors in people with type 2 diabetes Diabetologia, 47 (2004), pp. 821–832			X	X																		X														
K.J. Coppell, M. Kataoka, S.M. Williams, A.W. Chisholm, S.M. Vorgers, J.I. Mann Nutritional intervention in patients with type 2 diabetes who are hyperglycaemic despite optimised drug treatment—Lifestyle Over and Above Drugs in Diabetes (LOADD) study: randomised controlled trial BMJ, 341 (2010), p. c3337			X	X																																
M. Uusitupa, J. Laitinen, O. Siitonen, E. Vanninen, K. Pyorala The maintenance of improved metabolic control after intensified diet therapy in recent type 2 diabetes Diabetes Res Clin Pract, 19 (1993), pp. 227–238			X	X																										X					X	X
M. Ali, F. Schifano, P. Robinson, G. Phillips, L. Doherty, P. Melnick, et al. Impact of community pharmacy diabetes monitoring and education programme on diabetes management: a randomized controlled study Diabet Med, 29 (9) (2012), pp. e326–e333			X	X																																

For peer review only

H. Mohamed, B. Al-Lenjawi, P. Amuna, F. Zotor, H. Elmahdi Culturally sensitive patient-centred educational programme for self-management of type 2 diabetes: a randomized controlled trial Prim Care Diabetes, 7 (2013), pp. 199–206			X																																	
C.W. Chan, S.C. Siu, C.K. Wong, V.W. Lee A pharmacist care program: positive impact on cardiac risk in patients with type 2 diabetes J Cardiovasc Pharmacol Ther, 17 (2012), pp. 57–64			X	X																																
M.A. Sevick, M. Korytkowski, R.A. Stone, B. Piraino, D. Ren, S. Sereika, et al. Biophysiologic outcomes of the Enhancing Adherence in Type 2 Diabetes (ENHANCE) trial J Acad Nutr Diet, 112 (2012), pp. 1147–1157			X	X	X																															
W. Crasto, J. Jarvis, K. Khunti, T.C. Skinner, L.J. Gray, J. Brela, et al. Multifactorial intervention in individuals with type 2 diabetes and microalbuminuria: the Microalbuminuria Education and Medication Optimisation (MEMO) study Diabetes Res Clin Pract, 93 (2011), pp. 328–336			X	X																																
M.A. Salinero-Fort, E. Carrillo-de Santa Pau, F.J. Arrieta-Blanco, J.C. Abanades-Herranz, C. Martin-Madrazo, B. Rodes-Soldevila, et al. Effectiveness of PRECEDE model for health education on changes and level of control of HbA1c, blood pressure, lipids, and body mass index in patients with type 2 diabetes mellitus BMC Public Health, 11 (2011), p. 267			X		X																															
H. Sone, S. Tanaka, S. Iimuro, S. Tanaka, K. Oida, Y. Yamasaki, et al. Long-term lifestyle intervention lowers the incidence of stroke in Japanese patients with type 2 diabetes: a nationwide multicentre randomised controlled trial (the Japan Diabetes Complications Study) Diabetologia, 53 (2010), pp. 419–428			X	X																																
S.L. Krein, M.L. Klamerus, S. Vijan, J.L. Lee, J.T. Fitzgerald, A. Pawlow, et al. Case management for patients with poorly controlled diabetes: a randomized trial Am J Med, 116 (2004), pp. 732–739			X	X																		X														
M. Trento, P. Passera, M. Bajardi, M. Tomalino, G. Grassi, E. Borgo, et al. Lifestyle intervention by group care prevents deterioration of type II diabetes: a 4-year randomized controlled clinical trial. Diabetologia, 45 (2002), pp. 1231–1239			X	X													X	X				X						X				X				
AdachiMYamaokaKWatanabeMNishikawaMKobayashiI HidaEet alEffects of lifestyle education program for type 2 diabetes patients in clinics: a cluster randomized controlled trial.BMC Public Health201313467.					X																															

For peer review only

AdolfssonETWalker-EngströmMLSmideBWikbladKPatient education in type 2 diabetes: a randomized controlled 1-year follow-up study.Diabetes Res Clin Pract20077634150					X												X					X														
AmoakoESkellyAHRossenEKOutcomes of an intervention to reduce uncertainty among African American women with diabetes.West J Nurs Res20083092842					X					X																										
AndersonDRChristison-LagayJVillagraVLiuHDziuraJManaging the space between visits: a randomized trial of disease management for diabetes in a community health center.J Gen Intern Med201025111622					X																															
AndersonRMFunnellMMAikensJEKreinSLFitzgeraldJTNwankwoRet alEvaluating the efficacy of an empowerment-based self-management consultant intervention: results of a two-year randomized controlled trial.Ther Patient Educ20091311					X																															
AndersonRMFunnellMMButlerPMArnoldMSFitzgeraldJTFesteCCPatient empowerment. Results of a randomized controlled trial.Diabetes Care1995189439					X																														X		X
Anderson-LoftinWBarnett SBunnP SullivanPHusseyJTavakoli A Soul food light: culturally competent diabetes education.Diabetes Educ20053155563					X					X		X				X																				
BaksiAKAl-MrayatMHoganDWhittingstallEWilsonPWexJ Peer advisers compared with specialist health professionals in delivering a training programme on self-management to people with diabetes: a randomized controlled trial.Diabet Med200825107682					X																															
BarrattRFrostGMillwardDJTrubyHA randomised controlled trial investigating the effect of an intensive lifestyle intervention v.standard care in adults with type 2 diabetes immediately after initiating insulin therapy. Br J Nutr200899102531					X																															
Beverly EA, Fitzgerald SM, Brooks KM, Hultgren BA, Ganda OP,Munshi M, et al. Impact of reinforcement of diabetes self-care on poorly controlled diabetes: a randomized controlled trial. Diabetes Educ. 2013;39:504-14. [PMID: 23640303] doi:10.1177/0145721713486837					X																															
Bond GE, Burr R, Wolf FM, Price M, McCurry SM, Teri L. The effects of a web-based intervention on the physical outcomes associated with diabetes among adults age 60 and older: a randomized trial. Diabetes Technol Ther. 2007;9:52-9					X																															
Bozzetto L, Annuzzi G, Costabile G, Costagliola L, Giorgini M, Alderisio A, et al. A CHO/fibre diet reduces and a MUFA diet increases postprandial lipaemia in type 2 diabetes: no supplementary effects of low-volume physical training. Acta Diabetol. 2014;51:385-93					X																															
Bradshaw BG, Richardson GE, Kumpfer K, Carlson J,					X																															

For peer review only

Stanchfield J, Overall J, et al. Determining the efficacy of a resiliency training approach in adults with type 2 diabetes. Diabetes Educ. 2007;33: 650-9																																				
Brown SA, Blozis SA, Kouzekanani K, Garcia AA, Winchell M, Hanis CL. Dosage effects of diabetes self-management education for Mexican Americans: the Starr County Border Health Initiative. Diabetes Care. 2005;28:527-32					X					X																			X								
Brown SA, Garcı´a AA, Winter M, Silva L, Brown A, Hanis CL. Integrating education, group support, and case management for diabetic Hispanics. Ethn Dis. 2011;21:20-6					X																															
Castejo AM, Caldero JL, Perez A, Millar C, McLaughlinMiddlekauff J, Sangasubana N, et al. A community-based pilot study of a diabetes pharmacist intervention in Latinos: impact on weight and hemoglobin A1c. J Health Care Poor Underserved. 2013;24:48-60.					X																															
Chan JC, Sui Y, Oldenburg B, Zhang Y, Chung HH, Goggins W, et al; JADE and PEARL Project Team. Effects of telephone-based peer support in patients with type 2 diabetes mellitus receiving integrated care: a randomized clinical trial. JAMA Intern Med. 2014;174: 972-81.					X																															
Chan LS. Chronic disease self-management in Hong Kong Chinese older adults living in the community. Dissertation Abstracts International: Section B: The Sciences and Engineering. 2012;74:4-B E.					X																															
Cheong SH, McCargar LJ, Paty BW, Tudor-Locke C, Bell RC. The First Step First Bite Program: guidance to increase physical activity and daily intake of low-glycemic index foods. J Am Diet Assoc. 2009; 109:1411-6.					X																															
Chlebowy DO, El-Mallakh P, Myers J, Kubiak N, Cloud R, Wall MP. Motivational interviewing to improve diabetes outcomes in African Americans adults with diabetes. West J Nurs Res. 2015;37:566-80.					X																															
Clark M, Hampson SE, Avery L, Simpson R. Effects of a tailored lifestyle self-management intervention in patients with type 2 diabetes. Br J Health Psychol. 2004;9:365-79.					X																		X													
Cooper H, Booth K, Gill G. A trial of empowerment-based education in type 2 diabetes—global rather than glycaemic benefits. Diabetes Res Clin Pract. 2008;82:165-71.					X																															
Corkery E, Palmer C, Foley ME, Schechter CB, Frisher L, Roman SH. Effect of a bicultural community health worker on completion of diabetes education in a Hispanic population. Diabetes Care. 1997; 20:254-7.					X							X																						X		
Cramer JS, Sibley RF, Bartlett DP, Kahn LS, Loffredo L. An adaptation of the diabetes prevention program for use with high-risk, minority patients with type 2 diabetes.					X																	X														

For peer review only

Diabetes Educ. 2007;33:503-8																																				
Dasgupta K, Grover SA, Da Costa D, Lowensteyn I, Yale JF, Rahme E. Impact of modified glucose target and exercise interventions on vascular risk factors. Diabetes Res Clin Pract. 2006;72:53-60.					X																															
Davis RM, Hitch AD, Salaam MM, Herman WH, Zimmer-Galler IE, Mayer-Davis EJ. Tele Health improves diabetes self-management in an under served community: diabetes Tele Care. Diabetes Care. 2010;33:1712-7.					X																															
Deakin TA, Cade JE, Williams R, Greenwood DC. Structured patient education: the diabetes X-PERT Programme makes a difference. Diabet Med. 2006;23:944-54.					X												X											X								
Dunstan DW, Daly RM, Owen N, Jolley D, Vulikh E, Shaw J, et al. Home-based resistance training is not sufficient to maintain improved glycemic control following supervised training in older individuals with type 2 diabetes. Diabetes Care. 2005;28:3-9.					X																															
Dunstan DW, Mori TA, Puddey IB, Beilin LJ, Burke V, Morton AR, et al. The independent and combined effects of aerobic exercise and dietary fish intake on serum lipids and glycemic control in NIDDM. A randomized controlled study. Diabetes Care. 1997;20: 913-21.					X																															
Eakin EG, Winkler EA, Dunstan DW, Healy GN, Owen N, Marshall AM, et al. Living well with diabetes: 24-month outcomes from a randomized trial of telephone-delivered weight loss and physical activity intervention to improve glycemic control. Diabetes Care. 2014; 37:2177-85.					X																															
Edelman D, Dolor RJ, Coffman CJ, Pereira KC, Granger BB, Lindquist JH, et al. Nurse-led behavioral management of diabetes and hypertension in community practices: a randomized trial. J Gen Intern Med. 2015;30:626-33.					X																															
Fisher L, Hessler D, Glasgow RE, Arean PA, Masharani U, Naranjo D, et al. REDEEM: a pragmatic trial to reduce diabetes distress. Diabetes Care. 2013;36:2551-8.					X																															
Foster GD, Borradaile KE, Vander Veur SS, Leh Shantz K, Dilks RJ, Goldbacher EM, et al. The effects of a commercially available weight loss program among obese patients with type 2 diabetes: a randomized study. Postgrad Med. 2009;121:113-8.					X																															
Foster GD, Wadden TA, Lagrotte CA, Vander Veur SS, Hesson LA, Homko CJ, et al. A randomized comparison of a commercially available portion-controlled weight-loss intervention with a diabetes self-management education program. Nutr Diabetes. 2013;3:e63					X																															
Frosch DL, Uy V, Ochoa S, Mangione CM. Evaluation of a behavior support intervention for patients with poorly controlled diabetes. Arch Intern Med. 2011;171:2011-7.					X																															

For peer review only

Gagliardino JJ, Arrechea V, Assad D, Gagliardino GG, Gonza´lez L, Lucero S, et al. Type 2 diabetes patients educated by other patients perform at least as well as patients trained by professionals. Diabetes Metab Res Rev. 2013;29:152-60.					X																															
Gagliardino JJ, Lapertosa S, Pfirter G, Villagra M, Caporale JE, Gonzalez CD, et al; PRODIACOR. Clinical, metabolic and psychological outcomes and treatment costs of a prospective randomized trial based on different educational strategies to improve diabetes care (PRODIACOR). Diabet Med .2013;30:1102-11.					X																															
Giannopoulou I, Fernhall B, Carhart R, Weinstock RS, Baynard T, Figueroa A, et al. Effects of diet and/or exercise on the adipocytokine and inflammatory cytokine levels of postmenopausal women with type 2 diabetes. Metabolism. 2005;54:866-75					X																															
Glasgow RE, Kurz D, King D, Dickman JM, Faber AJ, Halterman E, et al. Twelve-month outcomes of an Internet-based diabetes selfmanagement support program. Patient Educ Couns. 2012;87:81-92.					X																															
Glasgow RE, Nutting PA, Toobert DJ, King DK, Strycker LA, Jex M, et al. Effects of a brief computer-assisted diabetes selfmanagement intervention on dietary, biological and quality-of-life outcomes. Chronic Illn. 2006;2:27-38.					X				X				X																	X						
Glasgow RE, Strycker LA, King DK, Toobert DJ, Rahm AK, Jex M, et al. Robustness of a computer-assisted diabetes self-management intervention across patient characteristics, healthcare settings, and intervention staff. Am J Manag Care. 2006;12:137-45.					X																	X														
Goudswaard AN, Stolk RP, Zuithoff NP, de Valk HW, Rutten GE. Long-term effects of self-management education for patients with Type 2 diabetes taking maximal oral hypoglycaemic therapy: a randomized trial in primary care. Diabet Med. 2004;21:491-6.					X													X			X		X				X									
Hawkins SY. Improving glycemic control in older adults using a videophone motivational diabetes self-management intervention.Res Theory Nurs Pract. 2010;24:217-32					X																															
Hendricks LE, Hendricks RT. The effect of diabetes selfmanagement education with frequent follow-up on the health outcomes of African American men. Diabetes Educ. 2000;26:995-1002.					X																															
Hermanns N, Kulzer B, Maier B, Mahr M, Haak T. The effect of an education programme (MEDIAS 2 ICT) involving intensive insulin treatment for people with type 2 diabetes. Patient Educ Couns. 2012;86:226-32.					X																															
Hill-Briggs F, Lazo M, Peyrot M, Doswell A, Chang YT, Hill MN, et al. Effect of problem-solving-based diabetes self-management training on diabetes control in a low income patient sample. J Gen Intern Med. 2011;26:972-8.					X																															
Holmen H, Torbjørnsen A, Wahl AK, Jenum AK, Småstuen MC, Arsand E, et al. A mobile health					X																															

For peer review only - http://bmjopen.bmj.com/site/about/guidelines.xhtml

intervention for self-management and lifestyle change for persons with type 2 diabetes, part 2: one year results from the Norwegian randomized controlled trial RENEWING HEALTH. JMIR Mhealth Uhealth. 2014;2:e57.																																				
Holtrop JS, Hickner J, Dosh S, Noel M, Ettenhofer TL. "Sticking to it—diabetes mellitus": a pilot study of an innovative behavior change program for women with type 2 diabetes. Am J Health Educ. 2002; 33:161-6.					x																															
Huisman S, de Gucht V, Maes S, Schroevers M, Chatrou M, Haak H. Self-regulation and weight reduction in patients with type 2 diabetes: a pilot intervention study. Patient Educ Couns. 2009;75:84-90.					x																															
Izquierdo RE, Knudson PE, Meyer S, Kearns J, Ploutz-Snyder R, Weinstock RS. A comparison of diabetes education administered through telemedicine versus in person. Diabetes Care. 2003;26: 1002-7.					x																															
Johnson ST, Bell GJ, McCargar LJ, Welsh RS, Bell RC. Improved cardiovascular health following a progressive walking and dietary intervention for type 2 diabetes. Diabetes Obes Metab. 2009;11: 836-43.					x																															
Jones H, Edwards L, Vallis TM, Ruggiero L, Rossi SR, Rossi JS, et al; Diabetes Stages of Change (DiSC) Study. Changes in diabetes self-care behaviors make a difference in glycemic control: the Diabetes Stages of Change (DiSC) study. Diabetes Care. 2003;26:732-7					x																															
Kim SH, Lee SH, Ahn KY, Lee DH, Suh YJ, Cho SG, et al. Effect of lifestyle modification on serum chemerin concentration and its association with insulin sensitivity in overweight and obese adults with type 2 diabetes. Clin Endocrinol (Oxf). 2014;80:825-33.					x																															
Kim SH, Lee SJ, Kang ES, Kang S, Hur KY, Lee HJ, et al. Effects of lifestyle modification on metabolic parameters and carotid intimamedia thickness in patients with type 2 diabetes mellitus. Metabolism. 2006;55:1053-9					x																															
Koo BK, Han KA, Ahn HJ, Jung JY, Kim HC, Min KW. The effects of total energy expenditure from all levels of physical activity vs. physical activity energy expenditure from moderate-to-vigorous activity on visceral fat and insulin sensitivity in obese Type 2 diabetic women. Diabet Med. 2010;27:1088-92					x																															
Kulzer B, Hermanns N, Reinecker H, Haak T. Effects of self management training in Type 2 diabetes: a randomized, prospective trial. Diabet Med. 2007;24:415-23.					x																															
Lorig K, Ritter PL, Laurent DD, Plant K, Green M, Jernigan VB, et al. Online diabetes self-management program: a randomized study. Diabetes Care. 2010;33:1275-81.					x				x				x	x																						
Lorig K, Ritter PL, Villa FJ, Armas J. Community-based peer-led diabetes self-management: a randomized trial. Diabetes Educ. 2009; 35:641-51.					x																															

For peer review only

Lynch EB, Liebman R, Ventrelle J, Avery EF, Richardson D. A self-management intervention for African Americans with comorbid diabetes and hypertension: a pilot randomized controlled trial. Prev Chronic Dis. 2014;11:E90.					X																															
Mandel SE, Davis BA, Secic M. Effects of music therapy and music-assisted relaxation and imagery on health-related outcomes in diabetes education: a feasibility study. Diabetes Educ. 2013;39:568-81.					X																															
Mayer-Davis EJ, D'Antonio AM, Smith SM, Kirkner G, Levin Martin S, Parra-Medina D, et al. Pounds off with empowerment (POWER): a clinical trial of weight management strategies for black and white adults with diabetes who live in medically underserved rural communities. Am J Public Health. 2004;94:1736-42.					X	X				X		X					X					X														
McGowan P. The efficacy of diabetes patient education and selfmanagement education in type 2 diabetes. Can J Diabetes. 2011;35: 46-53					X																															
Miller CK, Kristeller JL, Headings A, Nagaraja H. Comparison of a mindful eating intervention to a diabetes self-management intervention among adults with type 2 diabetes: a randomized controlled trial. Health Educ Behav. 2014;41:145-54.					X																															
Moncrieft AE. Randomized controlled trial of a behavioral weight loss intervention for primary prevention of renal decline in type 2 diabetics. Dissertation Abstracts International: Section B: The Sciences and Engineering. 2014;75:1-B E.					X																															
Moriyama M, Nakano M, Kuroe Y, Nin K, Niitani M, Nakaya T. Efficacy of a self-management education program for people with type 2 diabetes: results of a 12 month trial. Jpn J Nurs Sci. 2009;6:51-63.					X																															
Muchmore DB, Springer J, Miller M. Self-monitoring of blood glucose in overweight type 2 diabetic patients. Acta Diabetol. 1994; 31:215-9					X																															
Murrock CJ, Higgins PA, Killion C. Dance and peer support to improve diabetes outcomes in African American women. Diabetes Educ. 2009;35:995-1003.					X																															
Nishita C, Cardazone G, Uehara DL, Tom T. Empowered diabetes management: life coaching and pharmacist counseling for employed adults with diabetes. Health Educ Behav. 2013;40:581-91					X																															
Palmas W, Findley SE, Mejia M, Batista M, Teresi J, Kong J, et al. Results of the northern Manhattan diabetes community outreach project: a randomized trial studying a community health worker intervention to improve diabetes care in Hispanic adults. Diabetes Care. 2014;37:963-9.					X																															

For peer review only

Plotnikoff RC, Pickering MA, Glenn N, Doze SL, ReinboldMatthews ML, McLeod LJ, et al. The effects of a supplemental, theory-based physical activity counseling intervention for adults with type 2 diabetes. J Phys Act Health. 2011;8:944-54					X																															
Prezio EA, Cheng D, Balasubramanian BA, Shuval K, Kendzor DE, Culica D. Community Diabetes Education (CoDE) for uninsured Mexican Americans: a randomized controlled trial of a culturally tailored diabetes education and management program led by a community health worker. Diabetes Res Clin Pract. 2013;100:19-28					X																															
Reaney M, Zorzo EG, Golay A, Hermanns N, Cleall S, Petzinger U, et al. Impact of Conversation Map education tools versus regular care on diabetes-related knowledge of people with type 2 diabetes: a randomized, controlled study. Diabetes Spectr. 2013;26:236-45.					X																															
Rickheim PL, Weaver TW, Flader JL, Kendall DM. Assessment of group versus individual diabetes education: a randomized study. Diabetes Care. 2002;25:269-74.					X																			X								X				
Ridgeway NA, Harvill DR, Harvill LM, Falin TM, Forester GM, Gose OD. Improved control of type 2 diabetes mellitus: a practical education/behavior modification program in a primary care clinic. South Med J. 1999;92:667-72.					X																		X											X	X	X
Rock CL, Flatt SW, Pakiz B, Taylor KS, Leone AF, Brelje K, et al. Weight loss, glycemic control, and cardiovascular disease risk factors in response to differential diet composition in a weight loss program in type 2 diabetes: a randomized controlled trial. Diabetes Care. 2014;37:1573-80.					X																															
Rosal MC, Heyden R, Mejilla R, Capelson R, Chalmers KA, Rizzo DePaoli M, et al. A virtual world versus face-to-face intervention format to promote diabetes self-management among African American women: a pilot randomized clinical trial. JMIR Res Protoc. 2014;3: e54.					X																															
Rosal MC, Ockene IS, Restrepo A, White MJ, Borg A, Olendzki B, et al. Randomized trial of a literacy-sensitive, culturally tailored diabetes self-management intervention for low-income latinos: latinos en control. Diabetes Care. 2011;34:838-44.					X		X			X																										
Ruggiero L, Moadsiri A, Butler P, Oros SM, Berbaum ML, Whitman S, et al. Supporting diabetes self-care in underserved populations: a randomized pilot study using medical assistant coaches. Diabetes Educ. 2010;36:127-31.					X					X																										
Ruggiero L, Riley BB, Hernandez R, Quinn LT, Gerber BS, Castillo A, et al. Medical assistant coaching to support diabetes self-care among low-income racial/ethnic minority populations: randomized controlled trial. West J Nurs Res. 2014;36:1052-73					X																															
Sacco WP, Malone JI, Morrison AD, Friedman A, Wells K. Effect of a brief, regular telephone intervention by					X																															

paraprofessionals for type 2 diabetes. J Behav Med. 2009;32:349-59.																																				
Sarkadi A, Rosenqvist U. Experience-based group education in Type 2 diabetes: a randomised controlled trial. Patient Educ Couns. 2004;53:291-8					X												X					X						X								
Shibayama T, Kobayashi K, Takano A, Kadowaki T, Kazuma K. Effectiveness of lifestyle counseling by certified expert nurse of Japan for non-insulin-treated diabetic outpatients: a 1-year randomized controlled trial. Diabetes Res Clin Pract. 2007;76:265-8.					X																	X		X												
Sigurdardottir AK, Benediktsson R, Jonsdottir H. Instruments to tailor care of people with type 2 diabetes. J Adv Nurs. 2009;65:2118-30.					X																															
Siminerio L, Ruppert KM, Gabbay RA. Who can provide diabetes self-management support in primary care? Findings from a randomized controlled trial. Diabetes Educ. 2013;39:705-13.					X																															
Sinclair KA, Makahi EK, Shea-Solatorio C, Yoshimura SR, Townsend CK, Kaholokula JK. Outcomes from a diabetes self-management intervention for Native Hawaiians and Pacific People: Partners in Care. Ann Behav Med. 2013;45:24-32.					X																															
Smith DE, Heckemeyer CM, Kratt PP, Mason DA. Motivational interviewing to improve adherence to a behavioral weight-control program for older obese women with NIDDM. A pilot study. Diabetes Care. 1997;20:52-4.					X						X											X											X		
Sorkin DH, Mavandadi S, Rook KS, Biegler KA, Kilgore D, Dow E, et al. Dyadic collaboration in shared health behavior change: the effects of a randomized trial to test a lifestyle intervention for highrisk Latinas. Health Psychol. 2014;33:566-75.					X																															
Sperl-Hillen J, Beaton S, Fernandes O, Von Worley A, VazquezBenitez G, Hanson A, et al. Are benefits from diabetes self-management education sustained? Am J Manag Care. 2013;19:104-12					X																															
Steed L, Lankester J, Barnard M, Earle K, Hurel S, Newman S. Evaluation of the UCL diabetes self-management programme (UCLDSMP): a randomized controlled trial. J Health Psychol. 2005;10: 261-76					X																	X														
Sung K, Bae S. Effects of a regular walking exercise program on behavioral and biochemical aspects in elderly people with type II diabetes. Nurs Health Sci. 2012;14:438-45.					X																															
Tang TS, Funnell M, Sinco B, Piatt G, Palmisano G, Spencer MS, et al. Comparative effectiveness of peer leaders and community health workers in diabetes self-management support: results of a randomized controlled trial. Diabetes Care. 2014;37:1525-34					X																															
Thoolen B, De Ridder D, Bensing J, Maas C, Griffin S, Gorter K, et al. Effectiveness of a self-management intervention in patients with screen-detected type 2 diabetes. Diabetes Care. 2007;30:2832-7					X																															

For peer review only

Toobert DJ, Glasgow RE, Strycker LA, Barrera M Jr, Radcliffe JL, Wander RC, et al. Biologic and quality-of-life outcomes from the Mediterranean Lifestyle Program: a randomized clinical trial. Diabetes Care. 2003;26:2288-93					X												X					X														
Toobert DJ, Strycker LA, King DK, Barrera M Jr, Osuna D, Glasgow RE. Long-term outcomes from a multiple-risk-factor diabetes trial for Latinas: Viva Bien!. Transl Behav Med. 2011;1:416-426.					X					X																										
Trief P, Sandberg JG, Ploutz-Snyder R, Brittain R, Cibula D, Scales K, et al. Promoting couples collaboration in type 2 diabetes: the diabetes support project pilot data. Fam Syst Health. 2011;29:253-61.					X																															
Tucker CM, Lopez MT, Campbell K, Marsiske M, Daly K, Nghiem K, et al. The effects of a culturally sensitive, empowermentfocused, community-based health promotion program on health outcomes of adults with type 2 diabetes. J Health Care Poor Underserved. 2014;25:292-307.					X																															
Utz SW, Williams IC, Jones R, Hinton I, Alexander G, Yan G, et al. Culturally tailored intervention for rural African Americans with type 2 diabetes. Diabetes Educ. 2008;34:854-65					X																															
Vadstrup ES, Frølich A, Perrild H, Borg E, Røder M. Effect of a group-based rehabilitation programme on glycaemic control and cardiovascular risk factors in type 2 diabetes patients: the Copenhagen Type 2 Diabetes Rehabilitation Project. Patient Educ Couns. 2011;84:185-90.					X																															
Varney JE, Weiland TJ, Inder WJ, Jelinek GA. Effect of hospitalbased telephone coaching on glycaemic control and adherence to management guidelines in type 2 diabetes, a randomised controlled trial. Intern Med J. 2014;44:890-7.					X																															
Vazquez IM, Millen B, Bissett L, Levenson SM, Chipkin SR. Buena Alimentacion, Buena Salud: a preventive nutrition intervention in Caribbean Latinos with type 2 diabetes. Am J Health Promot. 1998;13:116-9.					X					X																										
Walker EA, Shmukler C, Ullman R, Blanco E, ScollanKoliopoulus M, Cohen HW. Results of a successful telephonic intervention to improve diabetes control in urban adults: a randomized trial. Diabetes Care. 2011;34:2-7.					X					X																										
Weinger K, Beverly EA, Lee Y, Sitnokov L, Ganda OP, Caballero AE. The effect of a structured behavioral intervention on poorly controlled diabetes: a randomized controlled trial. Arch Intern Med. 2011;171:1990-9.					X																															
Welch G, Zagarins SE, Feinberg RG, Garb JL. Motivational interviewing delivered by diabetes educators: does it improve blood glucose control among poorly controlled type 2 diabetes patients? Diabetes Res					X																															

For peer review only - http://bmjopen.bmj.com/site/about/guidelines.xhtml

Clin Pract. 2011;91:54-60																																				
Welschen LM, van Oppen P, Bot SD, Kostense PJ, Dekker JM, Nijpels G. Effects of a cognitive behavioural treatment in patients with type 2 diabetes when added to managed care; a randomised controlled trial. J Behav Med. 2013;36:556-66.					x																															
West DS, DiLillo V, Bursac Z, Gore SA, Greene PG. Motivational interviewing improves weight loss in women with type 2 diabetes. Diabetes Care. 2007;30:1081-7.					x	x						x										x														
Wierenga ME. Life-style modification for weight control to improve diabetes health status. Patient Educ Couns. 1994;23:33-40.					x																															x
Wing RR, Bolin P, Brancati FL, Bray GA, Clark JM, Coday M, et al; Look AHEAD Research Group. Cardiovascular effects of intensive lifestyle intervention in type 2 diabetes. N Engl J Med. 2013;369:145-54.					x	x																														
Wolever RQ, Dreusicke M, Fikkan J, Hawkins TV, Yeung S, Wakefield J, et al. Integrative health coaching for patients with type 2 diabetes: a randomized clinical trial. Diabetes Educ. 2010;36:629-39.					x																															
Wolf AM, Conaway MR, Crowther JQ, Hazen KY, L Nadler J, Oneida B, et al; Improving Control with Activity and Nutrition (ICAN) Study. Translating lifestyle intervention to practice in obese patients with type 2 diabetes: Improving Control with Activity and Nutrition (ICAN) study. Diabetes Care. 2004;27:1570-6.					x	x														x		x														
Yoo JS, Lee SJ, Lee HC, Kim MJ. The effect of a comprehensive lifestyle modification program on glycemic control and body composition in patients with type 2 diabetes. Asian Nurs Res (Korean Soc Nurs Sci). 2007;1:106-15.					x																															
Yuan C, Lai CW, Chan LW, Chow M, Law HK, Ying M. The effect of diabetes self-management education on body weight, glycemic control, and other metabolic markers in patients with type 2 diabetes mellitus. J Diabetes Res. 2014;2014:789761.					x																															
Dixon JB, O'Brien PE, Playfair J et al. Adjustable gastric banding and conventional therapy for type 2 diabetes: a randomized controlled trial. JAMA 2008; 299: 316–323.						x																														
Aas AM, Bergstad I, Thorsbyt PM, Johannesen O, Solberg M, Birkelandt KI. An intensified lifestyle intervention programme may be superior to insulin treatment in poorly controlled Type 2 diabetic patients on oral hypoglycaemic agents: results of a feasibility study. Diabet Med 2005; 22: 316–322.						x																														
Luley C, Blaik A, Reschke K, Klose S, Westphal S. Weight loss in obese patients with type 2 diabetes: effects of telemonitoring plus a diet combination-the Active Body Control (ABC) Program. Diabetes Res Clin						x																														

For peer review only - http://bmjopen.bmj.com/site/about/guidelines.xhtml

Pract 2011; 91: 286–292.																																				
Redmon JB, Raatz SK, Reck KP et al. One-year outcome of a combination of weight loss therapies for subjects with type 2 diabetes: a randomized trial. Diabetes Care 2003; 26: 2505–2511.						x																														
Eakin EG, Reeves MM, Winkler E et al. Six-month outcomes from living well with diabetes: a randomized trial of a telephone-delivered weight loss and physical activity intervention to improve glycemic control. Ann Behav Med 2013; 46: 193–203.						x																														
Snel M, Gastaldelli A, Ouwens DM et al. Effects of adding exercise to a 16-week very low-calorie diet in obese, insulin-dependent type 2 diabetes mellitus patients. J Clin Endocrinol Metab 2012; 97: 2512–2520.						x																														
Palmer AJ, Roze S, Valentine WJ, Spinas GA, Shaw JE, et al. (2004) Intensive lifestyle changes or metformin in patients with impaired glucose tolerance: modelling the long-term health economic implications of the diabetes prevention program in Australia, France, Germany, Switzerland, and the United Kingdom. Clin Ther 26:304–321								x																												
Lindgren P, Lindström J, Tuomilehto J, Uusitupa M, Peltonen M, et al. (2007) Lifestyle intervention to prevent diabetes in men and women with impaired glucose tolerance is cost-effective. Int J Technol Assess Health Care 23: 177-183.								x																												
Tuomilehto J, Lindström J, Eriksson JG, Valle TT, Hämäläinen H, et al. (2001) Finnish Diabetes Prevention Study Group. Prevention of type 2 diabetes mellitus by changes in lifestyle among subjects with impaired glucose tolerance. N Engl J Med 344:1343-1350								x																												
Lindström J, Eriksson JG, Valle TT, Aunola S, Cepaitis Z, et al. (2003) Prevention of diabetes mellitus in subjects with impaired glucose tolerance in the Finnish Diabetes Prevention Study: results from a randomized clinical trial. J Am Soc Nephrol. 14:S108-S113								x																												
Palmer AJ, Tucker DM (2012) Cost and clinical implications of diabetes prevention in an Australian setting: a long-term modeling analysis. Prim Care Diabetes 6: 109-121.								x																												
Diabetes Prevention Program Research Group, Knowler WC, Fowler SE, Hamman RF, Christophi CA, et al. (2009) 10-year follow-up of diabetes incidence and weight loss in the Diabetes Prevention Program Outcomes Study. Lancet 374:1677-1686.								x																												
Herman WH, Hoerger TJ, Brandle M, Hicks K, Sorensen S, et al. (2005) The cost-effectiveness of lifestyle modifcation or metformin in preventing type 2 diabetes in adults with impaired glucose tolerance. Ann Intern Med 142: 323-								x																												

332.																																				
CDC Diabetes Cost-effectiveness Group (2002) Cost-effectiveness of intensive glycemic control, intensifed hypertension control, and serum cholesterol level reduction for type 2 diabetes. JAMA 287: 2542-2551								X																												
Diabetes Prevention Program Research Group1 (2012) The 10-year costeffectiveness of lifestyle intervention or metformin for diabetes prevention: an intent-to-treat analysis of the DPP/DPPOS. Diabetes Care 35: 723-730								X																												
Herman WH, Edelstein SL, Ratner RE, Montez MG, Ackermann RT, et al. (2013) Diabetes Prevention Program Research Group. Effectiveness and costeffectiveness of diabetes prevention among adherent participants. Am J Manag Care 19:194-202								X																												
Diabetes Prevention Program Research Group (2003) Within-trial costeffectiveness of lifestyle intervention or metformin for the primary prevention of type 2 diabetes. Diabetes Care 26: 2518-2523								X																												
Eddy DM1, Schlessinger L (2003) Archimedes: a trial-validated model of diabetes. Diabetes Care 26: 3093-3101.								X																												
Mason JM, Freemantle N, Gibson JM, New JP (2005) SPLINT trial. Specialist nurse-led clinics to improve control of hypertension and hyperlipidemia in diabetes: economic analysis of the SPLINT trial. Diabetes Care 28:40–46								X																												
New JP, Mason JM, Freemantle N, Teasdale S, Wong LM, et al. (2003) Specialist nurse-led intervention to treat and control hypertension and hyperlipidemia in diabetes (SPLINT): a randomized controlled trial. Diabetes Care 26: 2250-2255.								X																												
Gillett M, Dallosso HM, Dixon S, Brennan A, Carey ME, et al. (2010) Delivering the diabetes education and self-management for ongoing and newly diagnosed (DESMOND) programme for people with newly diagnosed type 2 diabetes: cost effectiveness analysis. BMJ 341:c4093								X																												
Davies MJ, Heller S, Skinner TC, Campbell MJ, Carey ME, et al. (2008) Diabetes Education and Self-Management for Ongoing and Newly Diagnosed Collaborative. Effectiveness of the diabetes education and self-management for ongoing and newly diagnosed (DESMOND) programme for people with newly diagnosed type 2 diabetes: cluster randomised controlled trial. BMJ 336:491-495								X																												
Dijkstra RF, Niessen LW, Braspenning JC, Adang E, Grol RT (2006) Patientcentred and professional-directed implementation strategies for diabetes guidelines: a cluster-randomized trial-based cost-effectiveness analysis. Diabet Med 23:164-170.								X																												
Dijkstra RF, Braspenning JC, Huijsmans Z, Akkermans RP, van Ballegooie E, et al. (2005) Introduction of								X																												

For peer review only

For peer review only - http://bmjopen.bmj.com/site/about/guidelines.xhtml

diabetes passports involving both patients and professionals to improve hospital outpatient diabetes care. Diabetes Res Clin Pract 68: 126-134.																																				
Quinn CC, Clough SS, Minor JM, Lender D, Okafor MC, Gruber-Baldini A. WellDoc mobile diabetes management randomized controlled trial: change in clinical and behavioral outcomes and patient and physician satisfaction. Diabetes Technol Ther 2008;10:160–168									X				X																							
Smith L, Weinert C. Telecommunication support for rural women with diabetes. Diabetes Educ 2000;26:645–655									X				X																							
Glasgow RE, Nutting PA, King DK, et al. Randomized effectiveness trial of a computer-assisted intervention to improve diabetes care. Diabetes Care 2005;28:33–39									X				X																							
Christian JG, Bessesen DH, Byers TE, Christian KK, Goldstein MG, Bock BC. Clinic-based support to help overweight patients with type 2 diabetes increase physical activity and lose weight. Arch Intern Med 2008;168:141–146									X				X																							
Glasgow RE, La Chance PA, Toobert DJ, Brown J, Hampson SE, Riddle MC. Long-term effects and costs of brief behavioural dietary intervention for patients with diabetes delivered from the medical office. Patient Educ Couns 1997;32:175–184									X				X																					X		
Leu MG, Norris TE, Hummel J, Isaac M, Brogan MW. A randomized, controlled trial of an automated wireless messaging system for diabetes. Diabetes Technol Ther 2005;7:710–718; discussion 719–720									X				X																							
Lim S, Kang SM, Shin H, et al. Improved glycemic control without hypoglycemia in elderly diabetic patients using the ubiquitous healthcare service, a new medical information system. Diabetes Care 2011;34:308–313									X				X																							
Quinn CC, Shardell MD, Terrin ML, Barr EA, Ballew SH, Gruber-Baldini AL. Cluster-randomized trial of a mobile phone personalized behavioral intervention for blood glucose control. Diabetes Care 2011;34:1934–1942									X				X																							
Wise PH, Dowlatshahi DC, Farrant S, Fromson S, Meadows KA. Effect of computer-based learning on diabetes knowledge and control. Diabetes Care 1986;9:504–508									X				X																					X	X	X
Yoo HJ, Park MS, Kim TN, et al. A Ubiquitous Chronic Disease Care system using cellular phones and the internet. Diabet Med 2009;26:628–635pmid.									X		X		X																							
Glasgow RE, Kurz D, King D, et al. Outcomes of minimal and moderate support versions of an internet-based diabetes self-management support program. J Gen Intern Med 2010;25:1315–1322									X				X	X																						
Glasgow RE, Boles SM, McKay HG, Feil EG, Barrera M									X				X	X						X																

For peer review only - http://bmjopen.bmj.com/site/about/guidelines.xhtml

Jr. The D-Net diabetes self-management program: long-term implementation, outcomes, and generalization results. Prev Med 2003;36:410–419																																				
Zhou YD, Gu W. [Computer assisted nutrition therapy for patients with type 2 diabetes]. Zhejiang Da Xue Xue Bao Yi Xue Ban 2003;32:244–248									X																											
Lo R, Lo B, Wells E, Chard M, Hathaway J. The development and evaluation of a computer-aided diabetes education program. Aust J Adv Nurs 1996;13:19–27.									X				X																							X
Anderson-Loftin W, Barnett S, Sullivan P, Bunn PS, Anderson-Loftin W, Barnett S, Sullivan P, Bunn PS, Tavakoli A: Culturally competent dietary education for southern rural African Americans with diabetes. Diabetes Educ. 2002, 28 (2): 245-257.										X																										
Banister NA, Jastrow ST, Hodges V, Loop R, Gillham MB: Diabetes self-management training program in a community clinic improves patient outcomes at modest cost. J Am Diet Assoc. 2004, 104 (5): 807-810.										X																										
Basch CE, Walker EA, Howard CJ, Shamoon H, Zybert P: The effect of health education on the rate of ophthalmic examinations among African Americans with diabetes mellitus. Am J Public Health. 1999, 89 (12): 1878-1882.										X																										
Borges WJ, Ostwald SK: Improving foot self-care behaviors with Pies Sanos. West J Nurs Res. 2008, 30 (3): 325-341.										X																										
Brown SA, Hanis CL: A community-based, culturally sensitive education and group-support intervention for Mexican Americans with NIDDM: a pilot study of efficacy. Diabetes Educ. 1995, 21 (3): 203-210.										X																										
Culica D, Walton JW, Prezio EA: CoDE: Community Diabetes Education for uninsured Mexican Americans. Proc (Bayl Univ Med Cent). 2007, 20 (2): 111-117.										X																										
Davis RM, Hitch AD, Salaam MM, Herman WH, Zimmer-Galler IE, Mayer-Davis EJ: TeleHealth Improves Diabetes Self-Management in an Underserved Community: Diabetes TeleCare. Diabetes Care. 2010, 33 (8): 1712-1717.										X																										
Ebbesson SO, Ebbesson LO, Swenson M, Kennish JM, Robbins DC: A successful diabetes prevention study in Eskimos: the Alaska Siberia project. Int J Circumpolar Health. 2005, 64 (4): 409-424.										X																										
Elshaw EB, Young EA, Saunders MJ, McGurn WC, Lopez LC: Utilizing a 24-hour dietary recall and culturally specific diabetes education in Mexican Americans with diabetes. Diabetes Educ. 1994, 20 (3): 228-235.										X		X																								X
Long JA, Jahnle EC, Richardson DM, Loewenstein G, Volpp KG: Peer mentoring and financial incentives to improve glucose control in african american veterans: a randomized trial. Ann Intern Med. 2012, 156 (6): 416-424.										X																										

For peer review only

Mauldon M, Melkus GD, Cagganello M, Mauldon M, Melkus GDE, Cagganello M: Tomando Control: a culturally appropriate diabetes education program for Spanish-speaking individuals with type 2 diabetes mellitus–evaluation of a pilot project. Diabetes Educ. 2006, 32 (5): 751-760.										X																										
Melkus GD, Spollett G, Jefferson V, Chyun D, Tuohy B, Robinson T, Kaisen A: A culturally competent intervention of education and care for black women with type 2 diabetes. Appl Nurs Res. 2004, 17 (1): 10-20.										X																										
Rimmer JH, Silverman K, Braunschweig C, Quinn L, Liu Y, Feasibility of a health promotion intervention for a group of predominantly African American women with type 2 diabetes. Diabetes Educ. 2002, 28 (4): 571-580.										X																										
Schillinger D, Handley M, Wang F, Hammer H, Schillinger D, Handley M, Wang F, Hammer H: Effects of self-management support on structure, process, and outcomes among vulnerable patients with diabetes: a three-arm practical clinical trial. Diabetes Care. 2009, 32 (4): 559-566.										X																										
Two FJ, Kieffer EC, Palmisano G, Anderson M, Sinco B, Janz N, Heisler M, Spencer M, Guzman R, Thompson J, Wisdom K, James SA: Racial and Ethnic Approaches to Community Health (REACH) Detroit partnership: improving diabetes-related outcomes among African American and Latino adults. Am J Public Health. 2005, 95 (9): 1552-1560.										X																										
C.T. Liu, Y.T. Yeh, T.I. Lee, Y.C. Li Observations on online services for diabetes management Diab. Care, 28 (2005), pp. 2807–2808											X																									
H.S. Kim A randomized controlled trial of a nurse short-message service by cellular phone for people with diabetes Int. J. Nurs. Stud., 44 (2007), pp. 687–692											X																									
Z. Faridi, L. Liberti, K. Shuval, V. Northrup, A. Ali, D.L. Katz Evaluating the impact of mobile telephone technology on type 2 diabetic patients' self-management: the NICHE pilot study J. Evaluation Clin. Pract., 14 (2008), pp. 465–469											X																									
K.H. Yoon, H.S. Kim A short message service by cellular phone in type 2 diabetic patients for 12 months Diab. Res. Clin. Pract., 79 (2008), pp. 256–261											X																									
C.S. Kim, S.Y. Park, J.G. Kang, S.J. Lee, S.H. Ihm, M.G. Choi, H.J. Yoo Insulin dose titration system in diabetes patients using a short messaging service automatically produced by a knowledge matrix Diab. Technol. Therapeut., 12 (2010), pp. 663–669											X																									

For peer review only

A.S. Shetty, S. Chamukuttan, A. Nanditha, R.K. Raj, A. Ramachandran Reinforcement of adherence to prescription recommendations in Asian Indian diabetes patients using short message service (SMS) – a pilot study J. Assoc. Phys. India, 59 (2011), pp. 711–714											X																									
W.I. Hussein, K. Hasan, A.A. Jaradat Effectiveness of mobile phone short message service on diabetes mellitus management; the SMS-DM study Diab. Res. Clin. Pract., 94 (2011), pp. e24–e26											X																									
M. Goodarzi, I. Ebrahimzadeh, A. Rabi, B. Saedipoor, M.A. Jafarabadi Impact of distance education via mobile phone text messaging on knowledge, attitude, practice and self efficacy of patients with type 2 diabetes mellitus in Iran J. Diab. Metabolic Disorders, 11 (2012), p. 10											X																									
S. Arora, A.L. Peters, E. Burner, C.N. Lam, M. Menchine Trial to Examine Text Message-based mHealth in emergency department patients with diabetes (TExT-MED): a randomized controlled trial Ann. Emerg. Med. (2013) (Epub ahead of print)											X																									
R.M. Anderson, M.M. Funnell, M.S. Arnold, P.A. Barr, G.J. Edwards, J.T. Fitzgerald Assessing the cultural relevance of an education program for urban African Americans with diabetes Diabetes Educ, 26 (2000), pp. 280–289												X																								
T.L. Gary, L.R. Bone, M.N. Hill, D.M. Levine, M. McGuire, C. Saudek, et al. Randomized controlled trial of the effects of nurse case manager and community health worker interventions on risk factors for diabetes-related complications in urban African Americans Prev Med, 37 (2003), pp. 23–32												X										X														
L.A. Jaber, H. Halapy, M. Fernet, S. Tummalapalli, H. Diwakaran Evaluation of a pharmaceutical care model on diabetes management Ann Pharmacother, 30 (1996), pp. 238–243												X							X										X				X			
W.L. McNabb, M.T. Quinn, L. Rosing Weight loss program for inner-city black women with non-insulin-dependent diabetes mellitus: pathways J Am Diet Assoc, 93 (1993), pp. 75–77												X																								
Zhou Y, Wei GU. Computer-assisted nutrition therapy for patients with type 2 diabetes. Journal of Zhejiang University (Medical Sciences) 2003;32(3):244-8.													X																							
van Bastelaar KM, Pouwer F, Cuijpers P, Riper H, Snoek FJ. Web-based depression treatment for type 1 and type 2 diabetic patients: a randomized, controlled trial. Diabetes Care 2011 Feb;34(2):320-325														X																						
Glasgow RE, Kurz D, King D, Dickman JM, Faber AJ,														X																						

For peer review only - http://bmjopen.bmj.com/site/about/guidelines.xhtml

Halterman E, et al. Twelve-month outcomes of an Internet-based diabetes self-management support program. Patient Educ Couns 2012 Apr;87(1):81-92																																				
Bond GE, Burr RL, Wolf FM, Feldt K. The effects of a web-based intervention on psychosocial well-being among adults aged 60 and older with diabetes: a randomized trial. Diabetes Educ 2010;36(3):446-456.														X						X																
Bond GE, Burr R, Wolf FM, Price M, McCurry SM, Teri L. The effects of a web-based intervention on the physical outcomes associated with diabetes among adults age 60 and older: a randomized trial. Diabetes Technol Ther 2007 Feb;9(1):52-59														X						X																
Bond GE, Burr R, Wolf FM, Price M, McCurry SM, Teri L. Preliminary findings of the effects of comorbidities on a web-based intervention on self-reported blood sugar readings among adults age 60 and older with diabetes. Telemed J E Health 2006 Dec;12(6):707-710.														X																						
Barrera M, Glasgow RE, McKay HG, Boles SM, Feil EG. Do Internet-based support interventions change perceptions of social support?: An experimental trial of approaches for supporting diabetes self-management. Am J Community Psychol 2002 Oct;30(5):637-654.														X																						
McKay HG, Glasgow RE, Feil EG, Boles SM, Barrera MJ. Internet-based diabetes self-management and support: Initial outcomes from the Diabetes Network project. Rehabilitation Psychology 2002;47(1):31-48.														X						X																
Lorig KR, Ritter PL, Laurent DD, Plant K. Internet-based chronic disease self-management: a randomized trial. Med Care 2006 Nov;44(11):964-971														X																						
Kim CJ, Kang DH. Utility of a Web-based intervention for individuals with type 2 diabetes: the impact on physical activity levels and glycemic control. Comput Inform Nurs 2006;24(6):337-345.														X						X																
Wangberg SC. An Internet-based diabetes self-care intervention tailored to self-efficacy. Health Educ Res 2008 Feb;23(1):170-179.														X																						
Barth R, Campbell L V, Allen S, Jupp J J, Chisholm D J. Intensive education improves knowledge, compHance, and foot problems in type 2 diabetes. Diabetic Medicine 1991; 8(2):111-117															X																			X		X
Malone J M, Snyder M, Anderson G, Bernhard V M, Holloway G A (Jnr), Bunt T J. Prevention of amputation by diabetic education. American Journal of Surgeiy 1989; 158(6): 520-523; discussion 523-524.															X																			X		X
Sun P C, Jao S E, Lin H D, Chan R C, Chou C L, Wei S H. Improving preventive foot care for diabetic patients participating in group education. Journal of American Podiatric Medicine Association 2009; 99: 295-300.															X																					
Borges W J, Ostwald S K. Improving foot selfcare behaviors with Pies Sanos. Journal of the American Podiatric Medical Association 2008; 100(2): 101-104.															X																					
Corbett CEA Randomized pilot study of improving foot care in home health patients with diabetes. The Diabetes Educator 2003; 29: 273.															X																					

For peer review only

Lincoln N B, Radford K A, Game F L, Jeffcoate W J. Education for secondary prevention of foot ulcers in people with diabetes: a randomised controlled trial. Diabetologia 2008; 5 1 :1954-1961.															X																					
Litzelman D K, Slemenda S W Langefeld C D, Hays L M, Welch M A, et a!. Reduction of lower extremity clinical abnormalities in patients with non-insulin-dependent diabetes mellitus. A randomized, controlled trial. Annals of Internal Medicine 1993; 119(1): 36-41.															X																					
Viswanathan V, Madhavan S, Rajasekar S, Chamukuttan S, Ambady R. Amputation prevention initiative in South India Positive impact of foot care education. Diabetes Care 2005; 28:1019-1021.															X																					
Skelly AH, Carlson JR, Leeman J, Holditch-Davis D, Soward A. Symptoms-focused management for African American women with type 2 diabetes: a pilot study. Appl Nurs Res. 2005; 18 (4): 213–220.																X					X					X										
Cabrera-Pivaral CE, Gonzalez-Perez G, Vega-Lopez MG, Rias-Merino ED: Impact of participatory education on body mass index and blood glucose in obese type-2 diabetics. Cadernos de Saude Publica. 2004, 8: 275-281.																	X																			
Clancy DE, Huang P, Okonofua E, Yeager D, Magruder KM: Group visits: promoting adherence to diabetes guidelines. Soc Gen Intern Med. 2007, 16: 620-624.																	X	X																		
Cooper HC, Booth K, Gill G: Patients' perspectives on diabetes health care education. Theory Pract. 2003, 18: 191-206.																	X											X								
Heller SR, Clarke P, Daly H, Davis I, McCulloch DK, Allison SP, et al: Group education for obese patients with type 2 diabetes: greater success at less cost. Diabet Med. 1988, 5: 552-556.																	X											X				X		X	X	X
Holtrop JS, Hickner J, Dosh S, Noel M, Ettenhofer TL: "Sticking to it – diabetes mellitus": a pilot study of an innovative behavior change program for women with type 2 diabetes. Am J Heal Educ. 2002, 33: 161-166.																	X															X				
Hornsten A, Lundman B, Stenlund H, Sandstrom H: Metabolic improvement after intervention focusing on personal understanding in type 2 diabetes. Diabetes Res Clin Pract. 2004, 68: 65-74.																	X																			
Lozano ML, Armale MJ, Tena DI, Sanchez NC: The education of type-2 diabetics: why not in groups?. Aten Primaria. 1999, 23: 485-492.																	X															X				
McKibbin CL, Patterson TL, Norman G, Patrick K, Jin H, Roesch S, et al: A lifestyle intervention for older schizophrenia patients with diabetes mellitus: a randomized controlled trial. Schizophr Res. 2006, 8: 36-44.																	X																			
Pennings-van der Eerden LM, Ripken T, van Heijst MP, Schrijvers AP: Evaluation of the impact of a patient education programme for NIDDM patients on knowledge, self-care behaviour, metabolic control and lipid profiles. Gedran en gezondheid. 1991, 19: 246-260.																	X																			

For peer review only - http://bmjopen.bmj.com/site/about/guidelines.xhtml

Toobert DJ, Glasgow RE, Strycker LA, Barrera M, Ritzwoller DP, Weidner G: Long-term effects of the Mediterranean lifestyle program: a randomized clinical trial for postmenopausal women with type 2 diabetes. Int J Behav Nutr Phys Act. 2007, 82: 1.																	X																			
Trento M, Passera P, Tomalino M, Pagnozzi F, Pomero F, Vaccari P, et al: Therapeutic group education in the follow-up of patients with non-insulin treated, non-insulin dependent diabetes mellitus. Diabetes Nutr Metab Clin Exp. 1998, 11: 212-216.																	X															X			X	X
Trento M, Passera P, Tomalino M, Bajardi M, Pomero F, Allione A, et al: Group visits improve metabolic control in type 2 diabetes: a 2-year follow-up. Diabetes Care. 2001, 24: 995-1000. 10.2337/diacare.24.6.995.																	X	X									X				X				
Trento M, Passera P, Borgo E, Tomalino M, Bajardi M, Cavallo F, et al: A 5-year randomized controlled study of learning, problem solving ability, and quality of life modifications in people with type 2 diabetes managed by group care. Diabetes Care. 2004, 112: 670-675.																	X	X									X								
Wattana C, Srisuphan W, Pothiban L, Upchurch SL: Effects of a diabetes self-management program on glycemic control, coronary heart disease risk, and quality of life among Thai patients with type 2 diabetes. Nurs Heal Sci. 2007, 9: 135-141.																	X					X														
Zapotoczky H, Semlitsch B, Herzog G, Bahadori B, Siebenhofer A, Pieber TR, et al: A controlled study of weight reduction in type 2 diabetics treated by two reinforcers. Int J Behav Med. 2001, 8: 42-49.																	X															X				
Deakin TA, Cade JE, Williams DDR, Greenwood DC: Empowered patients:better diabetes control, greater freedom to eat, no weight gain!. Diabetologia. 2003, 46 (suppl 2): A90.																	X										X				X				
Clancy D, Cope D, Magruder K, Peng H, Wolfman T. Evaluating Concordance to American Diabetes Association Standards of Care for Type 2 Diabetes Through Group Visits in an Uninsured or Inadequately Insured Patient Population. Diabetes Care. 2003; 26(7): 2032-2036.																		X																		
Kirsh S, Watts S, Pascuzzi K, O'Day M, Davidson D, Aron D, et al. Shared medical appointments based on the chronic care model: a quality improvement project to address the challenges of patients with diabetes with high cardiovascular risk. Qual Saf Health Care. 2007; 16(5): 349-353.																		X																		
Edelman D, Fredrickson S, Melnyk S, Coffman C, Jeffreys A, Weinberger M, et al. Medical clinics versus usual care for patients with both diabetes and hypertension: a randomized trial. Ann Intern Med. 2010; 152(11): 689-696.																		X																		
Taveira T, Friedmann P, Cohen L, Dooley A, Khatana S, Wu W, et al. Pharmacist-led group medical appointment model in type 2 diabetes. Diabetes Educ. 2010; 36(1): 109-117.																		X																		
Trento M, Basile M, Borgo E, Grassi G, Scuntero P,																		X																		

Porta M, et al. A randomised controlled clinical trial of nurse-, dietitian- and pedagogist-led Group Care for the management of Type 2 diabetes. J Endocrinol Invest. 2008; 31(11): 1038-1042.																																				
Pieber T, Holler A, Siebenhofer A, Brunner G, Semlitsch B, Krejs G, et al. Evaluation of a structured teaching and treatment programme for type 2 diabetes in general practice in a rural area of Austria. Diabet Med. 1995; 12(4): 349-354.																		X				X														
Trento M, Passera, P, Tomalino M, Pagnozzi F, Pomero F, Vaccari P, Bajardi M, Molinatti GM, Porta M. Theraputic group education in the follow-up of patients with non-insulin treated, non-insulin dependent diabetes mellitus. Diabetes Nutr Metab. 1998; 2(3):212-216.																		X																		
Trento M, Gamba S, Gentile L, Grassi G, Miselli V, Porta M, et al. Rethink Organization to iMprove Education and Outcomes (ROMEO): a multicenter randomized trial of lifestyle intervention by group care to manage type 2 diabetes. Diabetes Care. 2010; 33(4): 745-747.																		X																		
Clancy D, Brown S, Magruder K, Huang P. Group visits in medically and economically disadvantaged patients with type 2 diabetes and their relationships to clinical outcomes. Top Health Inf Manage. 2003; 24(1): 8-14.																		X													X					
Bray P, Thompson D, Wynn J, Cummings D, Whetstone L. Confronting disparities in diabetes care: the clinical effectiveness of redesigning care management for minority patients in rural primary care practices. J Rural Health. 2005; 21(4): 317-321.																		X																		
Culhane-Pera K, Peterson K, Crain A, Center B, Lee M, Xiong T, et al. Group visits for Hmong adults with type 2 diabetes mellitus: a pre-post analysis. J Health Care Poor Underserved. 2005; 16(2): 315-327.																		X																		
Mullan RJ, Montori VM, Shah ND, Christianson TJH, Bryant SC, Guyatt GH, et al. The diabetes mellitus medication choice decision aid: a randomized trial. Arch Intern Med. 2009;169(17):15608																			X																	
Fornos JA, Andres NF, Andres JC, Guerra MM, Egea B. A pharmacotherapy follow-up program in patients with type-2 diabetes in community pharmacies in Spain. Pharm World Sci. 2006;28(2):65-72.																			X																	
Al Mazroui NR, Kamal MM, Ghabash NM, Yacout TA, Kole PL, McElnay JC. Influence of pharmaceutical care on health outcomes in patients with Type 2 diabetes mellitus. Br J Clin Pharmacol. 2009;67(5):547-57.																			X																	
Farmer AJ, Wade AN, French DP, Simon J, Yudkin P, Gray A, et al. Blood glucose selfmonitoring in type 2 diabetes: a randomised controlled trial. Health Technol Assess. 2009;13(15):iii																			X																	
Kulzer B, Hermanns N, Reinecker H, Haak T. Effects of self-management training in Type 2 diabetes: a randomized, prospective trial. Diabet Med. 2007;24(4):415-23.																			X																	
H.-S. Kwon, J.-H. Cho, H.-S. Kim, B.-R. Song, S.-H. Ko, J.-M. Lee, et al.																				X																

For peer review only - http://bmjopen.bmj.com/site/about/guidelines.xhtml

Establishment of blood glucose monitoring system using the Internet<br>Diabetes Care, 27 (2) (2004), pp. 478–483																																				
J.-H. Cho, S.-A. Chang, H.-S. Kwon, Y.-H. Choi, S.-H. Ko, S.-D. Moon, et al.<br>Long-term effect of the Internet-based glucose monitoring system on HbA1c reduction and glucose stability<br>Diabetes Care, 29 (12) (2006), pp. 2625–2631																			X																	
G.T. McMahon, H.E. Gomes, S. Hickson Hohne, T.M.-J. Hu, B.A. Levine, P.R. Conlin<br>Web-based care management in patients with poorly controlled diabetes<br>Diabetes Care, 28 (7) (2005), pp. 1624–1629																			X																	
G.E. Bond, R. Burr, F.M. Wolf, M. Price, S.M. McCurry, L. Teri<br>Preliminary findings of the fffects of comorbidities on a web-based intervention on self-reported blood sugar readings among adults age 60 and older with diabetes<br>Telemed. J. e-Health, 12 (6) (2006), pp. 707–710																			X																	
H.-S. Kim, H.-Y. Son<br>Technological intervention for obese patients with type 2 diabetes<br>Appl. Nurs. Res., 21 (2) (2008), pp. 84–89																			X																	
S.-I. Kim, H.-S. Kim<br>Effectiveness of mobile and internet intervention in patients with obese type 2 diabetes<br>Int. J. Med. Inf., 77 (6) (2008), pp. 399–404																			X																	
J.D. Ralston, I.B. Hirsch, J. Hoath, M. Mullen, A. Cheadle, H.I. Goldberg<br>Web-based collaborative care for type 2 diabetes<br>Diabetes Care, 32 (2) (2009), pp. 234–239																			X																	
J.-H. Noh, Y.-J. Cho, H.-W. Nam, J.-H. Kim, D.-J. Kim, H.-S. Yoo, et al.<br>Web-based comprehensive information system for self-management of diabetes mellitus<br>Diabetes Technol. Ther., 12 (5) (2010), pp. 333–337																			X																	
E.G. Fell, R.E. Glasgow, S. Boles, H.G. McKay<br>Who participates in Internet-based self-management programs? A study among novice computer users in a primary care setting<br>Diabetes Educ., 26 (5) (2000), pp. 806–811																			X																	
H.G. McKay, D. King, E.G. Eakin, J.R. Seeley, R.E. Glasgow<br>The Diabetes Network Internet-based physical activity intervention: a randomized pilot study<br>Diabetes Care, 24 (8) (2001), pp. 1328–1334																			X			X														
C. Richardson, K. Mehari, L. McIntyre, A. Janney, L. Fortlage, A. Sen, et al.																			X																	

For peer review only

A randomized trial comparing structured and lifestyle goals in an internet-mediated walking program for people with type 2 diabetes<br>Int. J. Behav. Nutr. Phys. Act., 4 (1) (2007), p. 59																																				
T. Liebreich, R. Plotnikoff, K. Courneya, N. Boule<br>Diabetes NetPLAY: a physical activity website and linked email counselling randomized intervention for individuals with type 2 diabetes<br>Int. J. Behav. Nutr. Phys. Act., 6 (1) (2009), p. 18																				X																
A. Holbrook, L. Thabane, K. Keshavjee, L. Dolovich, B. Bernstein, D. Chan, et al.<br>Individualized electronic decision support and reminders to improve diabetes care in the community: COMPETE II randomized trial<br>CMAJ, 181 (1–2) (2009), pp. 37–44																				X																
Keyserling TC, Samuel-Hodge CD, Ammerman AS, Ainsworth BE, Henriquez-Roldan CF, Elasy TA et al. A randomised trial of an intervention to improve self-care behaviours of African-American women with type 2<br>diabetes. Diabetes Care 2002; 25: 1576–1583.																					X									X						
K. Samaras, S. Ashwell, A.M. Mackintosh, A.C. Fleury, L.V. Campbell, D.J. Chisholm<br>Will older sedentary people with non-insulin-dependent diabetes mellitus start exercising? A health promotion model<br>Diabetes Res Clin Pract, 37 (1997), pp. 121–128																						X								X						
C.K. Miller, L. Edwards, G. Kissling, L. Sanville<br>Nutrition education improves metabolic outcomes among older adults with diabetes mellitus: results from a randomized controlled trial<br>Prev Med, 34 (2002), pp. 252–259																						X														
P. Gaede, M. Beck, P. Vedel, O. Pedersen<br>Limited impact of lifestyle education in patients with type 2 diabetes mellitus and microalbuminuria: results from a randomized intervention study<br>Diabetic Med, 18 (2001), pp. 104–108																						X														
C. Di Loreto, C. Fanelli, P. Lucidi, G. Murdolo, C.A. De, N. Parlanti, F. Santeusanio, P. Brunetti, P. De Feo<br>Validation of a counseling strategy to promote the adoption and the maintenance of physical activity by type 2 diabetic subjects<br>Diabetes Care, 26 (2003), pp. 404–408																						X														
R.A. Gabbay, I. Lendel, T.M. Saleem, G. Shaeffer, A.M. Adelman, D.T. Mauger, M. Collins, R.C. Polomano<br>Nurse case management improves blood pressure, emotional distress and diabetes complication screening<br>Diabetes Res Clin Pract, 71 (2006), pp. 28–35																						X														
S.H. Ko, K.H. Song, S.R. Kim, J.M. Lee, J.S. Kim, J.H. Shin<br>Long-term effects of a structured intensive diabetes education programme (SIDEP) in patients with type 2																						X						X								

For peer review only

diabetes mellitus—a 4-year follow-up study<br>Diabetic Med, 24 (2007), pp. 55–62																																				
P. Kronsbein, V. Jorgens, I. Muhlhauser, V. Scholz, A. Venhaus, M. Berger<br>Evaluation of a structured treatment and teaching programme on non-insulin-dependent diabetes<br>Lancet, 2 (1988), pp. 1407–1411																					X						X				X				
A.L. Estey, M.H. Tan, K. Mann<br>Follow-up intervention: its effect on compliance behavior to a diabetes regimen<br>Diabetes Educ, 16 (1990), pp. 291–295																					X												X		
M.J. Franz, A. Monk, B. Barry, K. McClain, T. Weaver, N. Cooper<br>Effectiveness of medical nutrition therapy provided by dieticians in the management of non-insulin-dependent diabetes mellitus: a randomized, controlled clinical trial<br>J Am Diet Assoc, 95 (1995), pp. 1009–1017																					X														
M.I. Uusitupa<br>Early lifestyle intervention in patients with non-insulin-dependent diabetes mellitus and impaired glucose tolerance<br>Ann Med, 28 (1996), pp. 445–449																					X								X						X
B.P. Krier, R.D. Parker, D. Grayson, G. Byrd<br>Effect of diabetes education on glucose control<br>J La State Med Soc, 151 (1999), pp. 86–92																					X							X				X			
H. Sone, A. Katagiri, S. Ishibashi, R. Abe, Y. Saito, T. Murase, R. Abe, Y. Ohashi, Y. Akanuma, N. Yamada, Japan Diabetes Complication Study Group<br>Effects of lifestyle modifications on patients with type 2 diabetes: the Japan Diabetes Complications Study (JDCS) study design, baseline analysis and three year-interim report<br>Horm Metab Res, 34 (2002), pp. 509–515																					X														
C.B. Taylor, N.H. Miller, K.R. Reilly, G. Greenwald, D. Cunning, A. Deeter, L. Abascal<br>Evaluation of nurse-care management system to improve outcomes in patients with complicated diabetes<br>Diabetes Care, 26 (2003), pp. 1058–1063																					X														
J.A. Oh, H.S. Kim, K.H. Yoon, E.S. Choi<br>A telephone-delivered intervention to improve glycemic control in type 2 diabetic patients<br>Yonsei Med J, 44 (2003), pp. 1–8																					X														
R. Whittemore, G.D. Melkus, A. Sullivan, M. Grey<br>A nurse-coaching intervention for women with type 2 diabetes<br>Diabetes Educ, 30 (2004), pp. 795–804																					X	X	X												
M. Maislos, D. Weisman<br>Multidisciplinary approach to patients with poorly																					X														

controlled type 2 diabetes mellitus: a prospective, randomized study<br>Acta Diabetol, 41 (2004), pp. 44–48																																				
R. Rachiman, I. Slavacheski, M. Berla, R. Frommer-Shapira, M. Ravid<br>Treatment of high-risk patients with diabetes: motivation and teaching intervention: a randomized, prospective 8-year follow-up study<br>J Am Soc Nephrol, 16 (2005), pp. S22–S26																					X														
R.J. Young, J. Taylor, T. Friede, S. Hollis, J.M. Mason, P. Lee, E. Burns, A.F. Long, T. Gambling, J.P. New, J.M. Gibson<br>Pro-active call center treatment support (PACCTS) to improve glucose control in type 2 diabetes: a randomized controlled trial<br>Diabetes Care, 28 (2005), pp. 278–282																					X														
M.S. Song, H.S. Kim<br>Effect of the diabetes outpatient intensive management programme on glycaemic control for type 2 diabetic patients<br>J Clin Nurs, 16 (2007), pp. 1367–1373																					X														
Z.W. Ma, M.X. Li, C.S. Wang<br>Effects of comprehensive psycho-intervention on life quality and carbohydrate metabolism in patients with type 2 diabetes mellitus<br>Zhongguo Linchuang Kangfu, 10 (2006), pp. 15–17																						X													
L. Zhao, Y.J. Sun, Q.G. Liu, M.M. Huan, J. Wu, L. Zhou, H.J. Shen, C. Jiang<br>Intervention effects of music relaxation therapy on the quality of life in patients with diabetic retinopathy<br>Zhongguo Linchuang Kangfu, 9 (2005), pp. 50–53																						X													
Z. Zeng, L. Ma, L. Tang, G. Luo, H. Zhou, L. Zhou<br>Comprehensive intervention on diabetes mellitus<br>Chin Ment Health J, 17 (2003), pp. 253–255																						X													
A. Kirk, N. Mutrie, P. MacIntyre, M. Fisher<br>Effects of a 12-month activity counselling intervention on glycaemic control and on the status of cardiovascular risk factors in people with type 2 diabetes<br>Diabetologia, 47 (2004), pp. 821–832																						X													
F.L. Huang, J.H. Ye, F. Huang<br>Effect of mental intervention on type 2 diabetics: randomized controlled study<br>Zhongguo Linchuang Kangfu, 8 (2004), pp. 5795–5798																						X													
R.A. McGinnis, A. McGrady, S.A. Cox, K.A. Grower-Dowling<br>Biofeedback-assisted relaxation in type 2 diabetes<br>Diabetes Care, 28 (2005), pp. 2145–2149																						X													
M. Siebolds, O. Gaedeke, U. Schwedes<br>Self-monitoring of blood glucose-psychological aspects																						X													

For peer review only

relevant to changes in hba1c in type 2 diabetic patients treated with diet or diet plus oral antidiabetic medication<br>Patient Educ Couns, 62 (2006), pp. 104–110																																				
R. Jackson, K. Asimakopoulou, A. Scammell<br>Assessment of the transtheoretical model as used by dietitians in promoting physical activity in people with type 2 diabetes<br>J Hum Nutr Diet, 20 (2007), pp. 27–36																							X													
P.J. Lustman, L.S. Griffith, K.E. Freedland, S.S. Kissel, R.E. Clouse<br>Cognitive behavior therapy for depression in type 2 diabetes mellitus: a randomized, controlled trial<br>Ann Intern Med, 129 (1998), pp. 613–621																							X													
C.A. Heitzmann, R.M. Kaplan, D.K. Wilson, J. Sandler<br>Sex differences in weight loss among adults with type ii diabetes mellitus<br>J Behav Med, 10 (1987), pp. 197–211																							X													X
L.V. Campbell, R. Barth, J.K. Gosper, J.J. Jupp, L.A. Simons, D.J. Chisholm<br>Impact of intensive educational approach to dietary change in NIDDM<br>Diabetes Care, 13 (1990), pp. 841–847																							X							X						X
J.D. Lane, C.C. McCaskill, S.L. Ross, M.N. Feinglos, R.S. Surwit<br>Relaxation training for NIDDM: predicting who may benefit<br>Diabetes Care, 16 (1993), pp. 1087–1094																							X											X		
E.M. Campbell, S. Redman, P.S. Moffitt, R.W. Sanson-Fisher<br>The relative effectiveness of educational and behavioral instruction programs for patients with NIDDM: a randomized trial<br>Diabetes Educator, 22 (1996), pp. 379–386																							X	X				X						X	X	X
J. Kenardy, M. Mensch, K. Bowen, B. Green, J. Walton<br>Group therapy for binge eating in type 2 diabetes: a randomized trial<br>Diabet Med, 19 (2002), pp. 234–239																							X													
S.W. Rabkin, E. Boyko, A. Wilson, D.A. Streja<br>A randomized clinical trial comparing behavior modification and individual counseling in the nutritional therapy of non-insulin-dependent diabetes mellitus: comparison of the effect on blood sugar, body weight, and serum lipids<br>Diabetes Care, 6 (1983), pp. 50–56																							X													
R.R. Wing, L.H. Epstein, M.P. Nowalk, R. Koeske, S. Hagg<br>Behavior change, weight loss, and physiological improvements in type ii diabetic patients<br>J Consult Clin Psych, 53 (1985), pp. 111–122																							X											X		X

For peer review only - http://bmjopen.bmj.com/site/about/guidelines.xhtml

S. Hartwell, R. Kaplan, J. Wallace<br>Comparison of behavioral interventions for control of type ii diabetes mellitus<br>Behav Ther, 17 (1986), pp. 447–461																							X							X						X
N. White, J. Carnahan, C.A. Nugent, T. Iwaoka, M.A. Dodson<br>Management of obese patients with diabetes mellitus: comparison of advice education with group management<br>Diabetes Care, 9 (1986), pp. 490–496																							X						X				X	X	X	X
F. Rodriguez, M. Alvarez<br>Modificacion de conducta y mejora en el cumplimiento del tratamiento en diabeticos tipo ii<br>Rev Esp Ter Comportamiento, 5 (1987), pp. 223–247																							X													
R.R. Wing, M.D. Marcus, L.H. Epstein, A. Jawad<br>A “Family-based” approach to the treatment of obese type ii diabetic patients<br>J Consult Clin Psych, 59 (1991), pp. 156–162																							X											X		
G.A. D’Eramo-Melkus, J. Wylie-Rosett, J.A. Hagan<br>Metabolic impact of education in NIDDM<br>Diabetes Care, 15 (1992), pp. 864–869																							X											X	X	X
S. Boehm, E.A. Schlenk, E. Raleigh, D. Ronis<br>Behavioral analysis and behavioral strategies to improve self-management of type ii diabetes<br>Clin Nurs Res, 2 (1993), pp. 327–344																							X											X		X
J.E. Aikens, T.A. Kiolbasa, R. Sobel<br>Psychological predictors of glycemic change with relaxation training in non-insulin-dependent diabetes mellitus<br>Psychother Psychosom, 66 (1997), pp. 302–306																							X											X		
J. Henry, P. Wilson, D. Bruce, D. Chisholm, P. Rawling<br>Cognitive-behavioural stress management for patients with non-insulin dependent diabetes mellitus<br>Psychol Health Med, 2 (1997), pp. 109–118																							X											X		
S.L. Jablon, B.D. Naliboff, S.L. Gilmore, M.J. Rosenthal<br>Effects of relaxation training on glucose tolerance and diabetic control in type ii diabetes<br>Appl Psychophys Biof, 22 (1997), pp. 155–169																							X													
X. Huang, L. Song, T. Li<br>The effect of social support on type ii diabetes with depression<br>Chin J Clin Psychol, 9 (2001), pp. 187–189																							X													
X. Zhu, Y. Gong, S. Yao<br>The effect of biofeedback assisted relaxation training on cytokines in patients with type ii diabetes<br>Chin J Clin Psychol, 9 (2001), pp. 170–172																							X													
R.S. Surwit, M.A. van Tilburg, N. Zucker, C.C. McCaskill, P. Parekh, M.N. Feinglos, C.L. Edwards, P. Williams,																							X													

For peer review only

J.D. Lane Stress management improves long-term glycemic control in type 2 diabetes Diabetes Care, 25 (2002), pp. 30–34																																				
T. Tsujiuchi, H. Kumano, K. Yoshiuchi, D. He, Y. Tsujiuchi, T. Kuboki, H. Suematsu, K. Hirao The effect of qi-gong relaxation exercise on the control of type 2 diabetes mellitus: a randomized controlled trial Diabetes Care, 25 (2002), pp. 241–242																							X													
Hiss RG, Lou Gillard M, Armbruster BA, McClure LA. Comprehensive evaluation of community-based diabetic patients: Effect of feedback to patients and their physicians: A randomized controlled trial. Diabetes Care 2001;24(4):690-4.																								X												
Dalmau Llorca MR, Garcia Bernal G, Aguilar Martin C, Palau Galindo A. [Group versus individual education for type-2 diabetes patients]. Atencion Primaria 2003;32(1):36-41.																								X												
Deakin T, Cade JE, Williams DDR, Greenwood DC. EXpert patient education versus routine treatment: process evaluation. Diabetologia 2002;45(Suppl 2): 317.																												X								
Brown SA, Hanis CL. Culturally competent diabetes education for Mexican Americans: the Starr County Study. Diabetes Educ 1999;25:226–36																												X								X
Cooper H, Booth K, Gill G. A randomised controlled study of education for people with type 2 diabetes. Liverpool: University of Liverpool; 2002.																												X								
Cooper H, Booth K, Gill G. Using combined research methods for exploring diabetes patient education. Patient Educ Couns 2003;51:45–52.																												X								
Raz I, Soskolne V, Stein P. Influence of small-group education sessions on glucose homeostasis in NIDDM. Diabetes Care 1988;11:67–71.																												X						X	X	X
Domenech MI, Assad D, Mazzei ME, Kronsbein P, Gagliardino JJ. Evaluation of the effectiveness of an ambulatory teaching/treatment programme for noninsulin dependent (type 2) diabetic patients. Acta Diabetol 1995;32:143–7.																												X			X	X				
Keyserling TC, Ammerman AS, Samuel-Hodge CD, Ingram AF, Skelly AH, et al. A diabetes management program for African American woment with type 2 diabetes. The diabetes educator 2000;26(5):796-805.																										X				X						
Vyas A, Haidery AZ, Wiles PG, Gill S, Roberts C, Cruikshank JK. A pilot randomised trial in primary care to investigate and improve knowledge, awareness and self-management among South Asians with diabetes in Manchester. Diabet Med 2003; 20: 1022–1026.																											X									
Anon (reporting study by Kerr L et al .). Tailored approach overcomes cultural barriers. Pharm J 2004; 273: 14.																											X									

For peer review only - http://bmjopen.bmj.com/site/about/guidelines.xhtml

Samanta A, Campbell JE, Spalding DL, Panja KK, Neogi SK, Burden AC. Dietary habits of Asian diabetics in a general practice clinic. Hum Nutr Appl Nutr 1987; 41A: 160–163.																											X									
Wallace P, Awan A, Talbot J. Health Advice for Asian women with diabetes. Prof Nurse 1996; 11: 794–796.																											X									
Simmons D. Diabetes self help facilitated by local diabetes research: The Coventry Asian Diabetes Support Group. Diabet Med 1992; 9: 866–869.																											X									
A. Mease, W.L. Whitlock, A. Brown, K. Moore, H. Pavliscsak, A. Dingbaum, et al. Telemedicine improved diabetic management Mil. Med., 165 (8) (2000), pp. 579–584																													X				X			
J.D. Piette, M. Weinberger, F.B. Kraemer, S.J. McPhee Impact of automated calls with nurse follow-up on diabetes treatment outcomes in a department of veterans affairs Health Care System Diab. Care, 24 (2) (2001), pp. 202–208																													X				X			
R. Rachmani, Z. Levi, I. Slavachevski, M. Avin, M. Ravid Teaching patients to monitor their risk factors retards the progression of vascular complications in high-risk patients with Type 2 diabetes mellitus. A randomized prospective study Diab. Med., 19 (2002), pp. 385–392																													X				X			
E.A. Coast-Senior, B.A. Kroner, C.L. Kelley, L.E. Trilli Management of patients with type 2 diabetes by pharmacists in primary care clinics The Ann. Pharmacother., 32 (1998), pp. 636–641																													X				X			
Y.-D. Jiang, L.-M. Chuang, H.-P. Wu, S.-J. Shiau, C.-H. Wang, Y.-J. Lee, et al. Assessment of the function and effect of diabetes education programs in Taiwan Diab. Res. Clin. Pract., 46 (1999), pp. 177–182																													X				X			
de Bont AJ, Baker IA, St Leger AS, Sweetnam PM, Wragg KG, Stephens SM, Hayes TM. A randomised controlled trial of the effect of low fat diet advice on dietary response in insulin independent diabetic women. Diabetologia 1981;21(6):529-33.																														X					X	
Gallagher A, Henderson W, Abraira C. Dietary patterns and metabolic control in diabetic diets: a prospective study of 51 outpatient men on unmeasured and exchange diets. Journal of the American College of Nutrition 1987;6(6):525-32																														X						
Gallagher AM, Abraira C, Henderson WG. A four-year prospective trial of unmeasured diet in lean diabetic adults. Diabetes Care 1984;7(6):557-65.																														X						
Glasgow RE, La Chance PA, Toobert DJ, Brown J, Hampson SE, Riddle MC. Long term effects and costs of brief behavioural dietary intervention for patients with diabetes delivered from the medical office. Patient																														X						

For peer review only

Education and Counseling 1997;32(3):175-184.																																				
Glasgow RE, Toobert DJ, Hampson SE. Effects of a Brief Office-Based Intervention to Facilitate Diabetes Dietary Self-Management. Diabetes Care 1996;19(8):835-42.																														X					X	X
Hockaday TDR, Hockaday JM, Mann JI, Turner RC. Prospective comparison of modified-fat-high-carbohydrate with standard low-carbohydrate dietary advice in the treatment of diabetes: a one year follow-up study. British Journal of Nutrition 1978;39:357-62.																														X						
Hockaday TDR, Pandher KS, Bron A, et al. Progression of established retinopathy is unrelated to glycosylated hemoglobin in non-insulin-dependent diabetes. Transplantation Proceedings 1986;18(6):1574-1575.																														X						
Howard-Williams J, Hillson RM, Bron A, Awdry P, Mann JI, Hockaday TDR. Retinopathy is associated with higher glycaemia in maturity-onset type diabetes. Diabetologia 1984;27:198-202.																														X						
Howard-Williams J, Patel P, Jelfs R, Carter RD, Awdry P, Bron A, et al. Polyunsaturated fatty acids and diabetic retinopathy. British Journal of Ophthalmology 1985;69(1):15-8.																														X						
Lopez-Espinoza I, Howard-Williams J, Mann JI, Carter RD, Hockaday TD. Fatty acid composition of platelet phospholipids in non-insulin-dependent diabetics randomized for dietary advice. British Journal of Nutrition 1984;52(1):41-7.																														X						
Mann JI, Hockaday TD, Hockaday JM, Turner RC. A prospective study of modified-fat and low-carbohydrate dietary advice in the treatment of maturity-onset diabetes [proceedings]. Proceedings of the Nutrition Society 1976;35(2):72A-73A.																														X						
Kaplan RM, Atkins CJ, Wilson DK. The cost-utility of diet and exercise interventions in non-insulin-dependent diabetes mellitus. Health Promotion 1987;2(4):331-340.																														X						
Kaplan RM, Hartwell SL, Wilson DK, Wallace JP. Effects of diet and exercise interventions on control and quality of life in non-insulin-dependent diabetes mellitus. Journal of General Internal Medicine 1987;2(4):220-8.																														X				X		X
Ligtenberg PC, Hoekstra JB, Bol E, Zonderland ML, Erkelens DW. Effects of physical training on metabolic control in elderly type 2 diabetes mellitus patients. Clinical Science 1997;93(2):127-35.																														X				X		
Milne RM, Mann JI, Chisholm AW, Williams SM. Long-term comparison of three dietary prescriptions in the treatment of NIDDM. Diabetes Care 1994;17(1):74-80.																														X						
Pascale RW, Wing RR, Butler BA, Mullen M, Bononi P. Effects of a behavioral weight loss program stressing calorie restriction versus calorie plus fat restriction in obese individuals with NIDDM or a family history of diabetes. Diabetes Care 1995;18(9):1241-8.																														X						
Tsihlias EB, Gibbs AL, McBurney MI, Wolever TMS. Comparison of high- and low-glycemic-index breakfast cereals with monounsaturated fat in the long-term																														X						

For peer review only - http://bmjopen.bmj.com/site/about/guidelines.xhtml

dietary management of type 2 diabetes. American Journal of Clinical Nutrition 2000;72(2):439-49																																				
Wolever TM, Schrade KB, Vogt JA, Tsihlias EB, McBurney MI. Do colonic short-chain fatty acids contribute to the long-term adaptation of blood lipids in subjects with type 2 diabetes consuming a high-fiber diet?. American Journal of Clinical Nutrition 2002;75(6):1023-30.																														x						
Laitinen J, Uusitupa M, Ahola I, Laakso M, Siitonen O. Metabolic and dietary variables associated with glycaemic control in patients with recently diagnosed Type II diabetes mellitus. Diabetes, Nutrition & Metabolism - Clinical & Experimental 1994;7(2):77-87.																														x						
Laitinen J, Uusitupa M, Ahola I, Siitonen O. Metabolic and dietary determinants of serum lipids in obese patients with recently diagnosed non-insulin-dependent diabetes. Annals of Medicine 1994;26(2):119-24.																														x						
Vanninen E, Laitinen J, Uusitupa M. Physical activity and fibrinogen concentration in newly diagnosed NIDDM. Diabetes Care 1994;17(9):1031-8.																														x						
Vanninen E, Uusitupa M, Lansimies E, Siitonen O, Laitinen J. Effect of metabolic control on autonomic function in obese patients with newly diagnosed type 2 diabetes. Diabetic Medicine 1993;10(1):66-73.																														x						
Vanninen E, Uusitupa M, Siitonen O, Laitinen J, Lansimies E. Habitual physical activity, aerobic capacity and metaboic control in patients with newly-diagnosed Type 2 (non-insulin-dependent) diabetes mellitus: Effect of 1-year diet and exercise intervention. Diabetologia 1992;35(4):340-346.																														x				x		
Wing RR, Epstein LH, Paternostro-Bayles M, Kriska A, Nowalk MP, Gooding W. Exercise in a behavioural weight control programme for obese patients with Type 2 (non-insulin-dependent) diabetes. Diabetologia 1988;31(12):902-9.																														x						
Marcus MD, Wing RR, Guare J, Blair EH, Jawad A. Lifetime prevalence of major depression and its effect on treatment outcome in obese type II diabetic patients. Diabetes Care 1992;15(2):253-5.																														x						
Wing RR, Marcus MD, Salata R, Epstein LH, Miaskiewicz S, Blair EH. Effects of a very-low-calorie diet on long-term glycemic control in obese type 2 diabetic subjects. see comments. Archives of Internal Medicine 1991;151(7):1334-40.																														x						
Wing RR, Blair E, Marcus M, Epstein LH, Harvey J. Year-long weight loss treatment for obese patients with type II diabetes: does including an intermittent very-low-calorie diet improve outcome?. American Journal of Medicine 1994;97(4):354-62.																														x						
Kronsbein P, Miihlhauser I,Venhaus A,Jorgens V, ScholzV. Berger M (1988).Evaluation of a structured treatment and teaching programme on noninsulin-dependent diabetes. Lancet 2: 1407-11.																															x					
Pieber TR, Brunner GA, Schnedl WJ, Schattenberg S,																																x				

For peer review only

Kaufmenn P, Krejs GJ. Evaluation of a structured outpatient group education program for intensive insulin therapy. Diabetes Care 1995;18:625-30.																																				
Canga N, De Irala J, Vara E, Duaso MJ, Ferrer A, Martinez-Gonzalez MA. Intervention study for smoking cessation in diabetic patients. Diabetes Care 2000;23(10):1455-60.																																	X			
Hopper SV, Miller JP, Birge C, Swift J. A randomized study of the impact of home health aides on diabetic control and utilization patterns. American Journal Public Health 1984;74(6):600-2.																																	X	X		
Matsuyama JR, Mason BJ, Jue SG. Pharmacists' interventions using an electronic medication-event monitoring device's adherence data versus pill counts. The Annals of Pharmacotherpay 1993;27:851-5.																																	X			
Pullar T, Birtwell AJ, Wiles PG, Hay A, Feely MP. Use of a pharmacologic indicator to compare compliance with tablets prescribed to be taken once, twice, or three times daily. Clinical pharmacology and therapeutics 1988;44:540-5																																	X			
Rosenkranz S. Polaroid-Fundusfotografie erhöht die Patientencompliance beim Screening auf diabetische Retinopathie. Diabetes und Stoffwechsel 1006;5:69-75																																	X			
Simmons D, Upjohn M, Gamble GD. Can medication packaging improve glycemic contol and blood pressure in type 2 diabetes?. Diabetes Care 2000;23(2):153-6																																	X			
Skaer TL, Sclar DA, Markowski DJ, Won JKH. Effect of value-added utilities on prescription refill compliance and Medicaid health care expenditures - a study of patients with non-insulin-dependent diabe. 1993;18:295-9.																																	X			
Smith DM, Norton JA, Weinberger M, McDonald CJ, Katz BP. Increasing prescribed office visits. A controlled trial in patients with diabetes mellitus. Medical Care 1986;24(3):189-99.																																	X			
Diehl AK, Sugarek NJ, Bauer RL. Medication compliance in non-insulin-dependent diabetes: a randomized comparison of chlorpropamide and insulin. Diabetes Care 1985;8(3):219-23																																	X			
Davidson MB, Karlan VJ, Hair TL. Effect of a pharmacist-managed diabetes care program in a free medical clinic. American Journal of Medical Quality 2000;15(4):137-42.																																	X			
Bradshaw C, McColl E, Eccles M, Bryce C, Sampson R. Can we improve the education for type 2 diabetes patients in general practice?. Practical Diabetes International 1999;16(8):241-5																																	X			
Clarke J, Crawford A, Nash DB. Evaluation of a comprehensive diabetes disease management program: progress in the struggle for sustained behavior change. Disease management 2002;5(2):77-86.																																	X			
Paes AHP, Bakker A, Soe-Agnie CJ. Impact of dosage frequency on patient compliance. Diabetes Care 1997;20(10):1512-7.																																	X			
Allen BT, DeLong ER, Feussner JR. Impact of glucose																																		X		

For peer review only

self-monitoring on non-insulin-treated patients with type 11 diabetes mellitus. Randomized controlled trial comparing blood and urine testing. Diabetes Care. 1990;13:1044-1050.																																				
Arseneau DL, Mason AC, Wood OB, Schwab E, Green D. A comparison of learning activity packages and classroom instruction for diet management of patients with non-insulin-dependent diabetes mellitus. Diabetes Educ. 1994;20:509-514.																																		X		X
Aubert RE, Herman WH, Waters J, et al. Nurse case management to improve glycemic control in diabetic patients in a health maintenance organization: a randomized controlled trial. Ann Intern Med. 1998;129:605-612																																		X		
Bloomgarden ZT, Karmally W, Metzger MJ, et al. Randomized, controlled trial of diabetic patient education: improved knowledge without improved metabolic status. Diabetes Care. 1987;10:263-272.																																		X	X	X
Campbell LV, Barth R, Gosper JK, Jupp JJ, Simons LA, Chisholm DJ. Impact of an intensive educational approach to dietary change in NIDDM. Diabetes Care. 1990;13:841-847.																																		X		
Falkenberg MG, Elwing BE, Goransson AM, Hellstrand BE, Riis UM. Problem oriented participatory education in the guidance of adults with non-insulintreated type 11 diabetes mellitus. Scand J Primary Health Care. 1986;4:157-164.																																		X	X	
Franz MJ, Monk A, Barry B, et al. Effectiveness of medical nutrition therapy provided by dietitians in the management of non-insulindependent diabetes mellitus: a randomized, controlled clinical trial. J Am Diet Assoc. 1995;95:1009-1017.																																		X	X	X
Genev NM, McGill M, Hoskins PL, et al. Continuing diabetes education by telephone. Diabetic Med. 1990;7:920-921.																																		X		
Glasgow RE, Toobert DJ, Mitchell DL, Donnelly JE, Calder D. Nutrition education and social learning interventions for type 11 diabetes. Diabetes Care. 1989;12:150-152.																																		X		X
Glasgow RE, Toobert DJ, Hampson SE, Brown JE, Lewinsohn PM, Donnelly J. Improving self-care among older patients with type 11 diabetes: the "Sixty Something..." Study. Patient Educ Counsel. 1992;19:61-74.																																		X	X	X
Greenfield S, Kaplan SH, Ware JE Jr, Yano EM, Frank HJ. Patients' participation in medical care: effects on blood sugar control and quality of life in diabetes. J Gen Intern Med. 1988;3:448-457.																																		X		
Hassell J, Medved E. Group/audiovisual instruction for patients with diabetes. Learning achievements and time economics. J Am Diet Assoc. 1975;66:465-470.																																		X		
Hayes TM, Harries J. Randomised controlled trial of routine hospital clinic care versus routine general practice care for type 11 diabetics. Br Med J (Clin Res																																		X		

For peer review only

ed.). 1984;289:728-730																																				
Hurwitz B, Goodman C, Yudkin J. Prompting the clinical care of non-insulin dependent (type 11) diabetic patients in an inner city area: one model of community care. Br Med J. 1993;306:624-630.																																	X		
Jones PM. Use of a course on self-control behavior techniques to increase adherence to prescribed frequency for self-monitoring of blood glucose. Diabetes Educ. 1990;16:296-303.																																	X		X
Koproski J, Pretto Z, Poretsky L. Effects of an intervention by a diabetes team in hospitalized patients with diabetes. Diabetes Care. 1997;20:1553-1555.																																	X		
Kumana CR, Ma JT, Kung A, Kou M, Lauder 1. An assessment of drug information sheets for diabetic patients: only active involvement by patients is helpful. Diabetes Res Clin Pract. 1988;5:225-231.																																	X		X
Miller CK, Jensen GL, Achterberg CL. Evaluation of a food label nutrition intervention for women with type 2 diabetes mellitus. J Am Diet Assoc. 1999;99:323-328																																	X		
Morgan BS, Littell DH. A closer look at teaching and contingency contracting with type 11 diabetes. Patient Educ Counsel. 1988;12:145-158.																																	X		
Morrish NJ, Cohen DL, Hicks B, Keen H. A controlled study of the effect of computer-aided analysis of home blood glucose monitoring on blood glucose control. Diabetic Med. 1989;6:591-594																																	X		
Mulrow C, Bailey S, Sonksen PH, Slavin B. Evaluation of an audiovisual diabetes education program: negative results of a randomized trial of patients with non-insulin-dependent diabetes mellitus. J Gen Intern Med. 1987;2:215-219.																																	X	X	X
Rabkin SW, Boyko E, Wilson A, Streja DA. A randomized clinical trial comparing behavior modification and individual counseling in the nutritional therapy of non-insulin-dependent diabetes mellitus: comparison of the effect on blood sugar, body weight, and serum lipids. Diabetes Care. 1983;6:50-56.																																	X		X
Rettig BA, Shrauger DG, Recker RR, Gallagher TF, Wiltse H. A randomized study of the effects of a home diabetes education program. Diabetes Care. 1986;9:173-178.																																	X		X
Rost KM, Flavin KS, Cole K, McGill JB. Change in metabolic control and functional status after hospitalization. Impact of patient activation intervention in diabetic patients. Diabetes Care. 1991;14:881-889.																																	X		
Sadur CN, Moline N, Costa M, et al. Diabetes management in a health maintenance organization. Efficacy of care management using cluster visits. Diabetes Care. 1999;22:2011-2017																																	X		
Sawicki PT, Didjurgeit U, Muhlhauser 1, Berger M. Behaviour therapy versus doctor's anti-smoking advice in diabetic patients. J Intern Med. 1993;234:407-409																																	X		
Scott RS, Beaven DW, Stafford JM. The effectiveness of diabetes education for non-insulin-dependent diabetic																																	X	X	X

For peer review only

persons. Diabetes Educ. 1984:36-39.																																				
Smith DM, Weinberger M, Katz BP A controlled trial to increase office visits and reduce hospitalizations of diabetic patients. J Gen Intern Med. 1987;2:232-238.																																		X		
Tu K, McDaniel G, Gay JT. Diabetes self-care knowledge, behaviors, and metabolic control of older adults-the effect of a posteducational follow-up program. Diabetes Educ. 1993; 19:25-30																																		X	X	X
Vinicor F, Cohen SJ, Mazzuca SA, et al. DIA-BEDS: a randomized trial of the effects of physician and/or patient education on diabetes patient outcomes. J Chron Dis. 1987;40:345-356.																																		X		X
Ward WK, Haas LB, Beard JC. A randomized, controlled comparison of instruction by a diabetes educator versus self-instruction in self-monitoring of blood glucose. Diabetes Care. 1985;8:284-286.																																		X		X
Weinberger M, Kirkman MS, Samsa GE et al. A nurse-coordinated intervention for primary care patients with non-insulindependent diabetes mellitus. J Gen Intern Med. 1995;10:59-66																																		X		
Wheeler LA, Wheeler ML, Ours P, Swider C. Evaluation of computer-based diet education in persons with diabetes mellitus and limited educational background. Diabetes Care. 1985;8:537-544.																																		X		X
Wing RR, Epstein LH, Nowalk MP, Scott N, Koeske R, Hagg S. Does self-monitoring of blood glucose levels improve dietary compliance for obese patients with type 11 diabetes? Am J Med. 1986;81:830-836																																		X	X	X
Wing RR, Epstein LH, Paternostro-Bayles M, Kriska A, Nowalk MP, Gooding W. Exericise in a behavioural weight control programme for obese patients with type 2 (non-insulin-dependent) diabetes. Diabetologia. 1988;31:902-909.																																		X		
Wood ER. Evaluation of a hospital-based education program for patients with diabetes. J Am Diet Assoc. 1989;89:354-358																																		X		X
de Weerdt I, Visser A, Kok G, de Weerdt O, van der Veen E: Randomized controlled multicentre evaluation of an education programme for insulin-treated diabetic patients: effects on metabolic control, quality of life, and costs of therapy. Diabet Med 8:338–345, 1991																																			X	X
Estey A, Tan M, Mann K: Follow-up intervention: its effect on compliance behavior to diabetes regimen. Diabetes Educ 16:291–295, 1990																																			X	X
Kaplan R, Wilson D, Hartwell S, Merino K, Wallace J: Prospective evaluation of HDL cholesterol changes after diet and physical conditioning programs for patients with type II diabetes mellitus. Diabetes Care 8:343–348,																																			X	X
Korhonen T, Uusitupa M, Aro A, Kumpulainen T, Siitonen O, Voutilainen E, Pyorala K: Efficacy of dietary instructions in newly diagnosed non-insulin-dependent diabetic patients. Acta Med Scand 222:323–331, 1987																																			X	X

For peer review only

For peer review only - http://bmjopen.bmj.com/site/about/guidelines.xhtml

Mazzuca SA, Moorman NH, Wheeler ML, Norton JA, Fineberg NS, Vinicor F, Cohen SJ, Clark CM Jr: The diabetes education study: a controlled trial of the effects of diabetes patient education. Diabetes Care 9:1–10,																																			x	x
McCulloch D, Mitchell R, Ambler J, Tattersall R: Influence of imaginative teaching of diet on compliance and metabolic control in insulin dependent diabetes. Br Med J 287:1858–1861, 1983																																			x	
Perry T, Mann J, Lewis-Barned N, Duncan A, Waldron M, Thompson C: Lifestyle intervention in people with insulin-dependent diabetes mellitus (IDDM). Eur J Clin Nutr 51:757–763, 1997																																			x	x
Turnin M-C, Beddok R, Clottes J, Martini P, Abadie R, Buisson J-C, Soule-Cupuy C, Bonneu M, Camare R, Anton J-P, Chrisment C, Farreny H, Bayard F, Tauber J-P: Telematic expert system Diabeto: new tool for diet self-monitoring for diabetic patients. Diabetes Care 15:204–212, 1992																																			x	x
Wing R, Epstein L, Nowalk M, Scott N: Self-regulation in the treatment of type II diabetes. Behav Ther 19:11–23, 1988																																			x	x
Fernando D: Knowledge about diabetes and metabolic control in diabetic patients. Ceylon Med J 38:18-21, 1993																																				x
Falkenberg M, Elwing B, Goransson A, Hellstrand B, Riis U: Problem-oriented participatory education in the guidance of adults with non-insulin-treated type-II diabetes mellitus. Scand J Prim Health Care 4:157-164, 1986																																				x
McCulloch D, Mitchell R, Ambler J, Tattersall R: Influence of imaginative teaching of diet on compliance and metabolic control in insulin-dependent diabetes. BMJ 287:1858-1861, 1983																																				x
Korhonen T, Huttunen J, Aro A, Hentinen M, Ihalainen O, Majander H, Siitonen O, Uusitupa M, Pyorala K: A controlled trial on the effects of patient education in the treatment of insulin-dependent diabetes. Diabetes Care 6:256- 261, 1983																																				x
Kendall P. Jansen G: Educating patients with diabetes: comparison of nutrient-based and exchange group methods. J Am Diet Assoc 90:238-243, 1990																																				x
de Weerdt I, Visser A, Kok G, van der Veen E: Randomized controlled evaluation of an education programme for insulin treated patients with diabetes: effects on psychosocial variables. Patient Educ Counsel 14:191-215, 1989																																				x
Brown S, Duchin S, Villagomez E: Diabetes education in a Mexican-American population: pilot testing of a research-based videotape. Diabetes Educator 18:47-51, 1992																																				x
Kim J-Y, Phillips T: The effectiveness of two forms of																																				x

For peer review only

corrective feedback in diabetes education. J Comput Based Instruct 18:14-18, 1991																																				
Colagiuri R, Colagiuri S, Naidu V: Can patients set their own educational priorities? Diabetes Res Clin Pract 30:131-136, 1995																																				X
Small R, Hill Hopper J: Evaluation of videotape teaching of self-monitoring of blood glucose by elderly diabetic patients. Consultant Pharmacist 7:24-27, 1992																																				X
Van Veldhuizen-Scott M, Widmer L, Stacey S, Popovich N: Developing and implementing a pharmaceutical care model in an ambulatory care setting for patients with diabetes. Diabetes Educator 21:117-123, 1995																																				X
Genev N, McGill M, Hoskins P, Constantino M, Plehwe W, Yue D, Turtle J: Continuting diabetes education by telephone. Diabet Med 7:920-921, 1990																																				X
Litzelman D, Slemenda C, Langefeld C, Hays L, Welch M, Bild D, Ford E, Vinicor F: Reduction of lower extremity clinical abnormalities in patients with noninsulin-dependent diabetes mellitus: a randomized, controlled trial. Ann Intern Med 119:36-41, 1993																																				X
Kruger S, Guthrie D: Foot-care: knowledge retention and self-care practices. Diabetes Educator 18:487-490, 1992																																				X
Hanefeld M, Fischer S, Schmechel H, Rothe G, Schulze J, Dude H, Schwanebeck U, Julius U: Diabetes intervention study: multi-intervention trial in newly diagnosed NIDDM. Diabetes Care 14:308-317, 1991																																				X
de Bont A, Baker I, St. Leger A, Sweetnam P, Wragg K, Stephens S, Hayes T: A randomised controlled trial of the effect of low-fat diet advice on dietary response in insulin-independent diabetic women. Diabetologia 21:529-533, 1981																																				X
Glasgow R, La Chance P-A, Toobert D, Brown J, Hampson S, Riddle M: Long term effects and costs of brief behavioral dietary intervention for patients with diabetes delivered from the medical office. Patient Educ Couns 32:175-184, 1997																																				X
Mengham L, Morris B, Palmer C, White A: Is intensive dietetic intervention effective for overweight patients with diabetes mellitus? A randomised controlled study in a general practice. Practical Diabetes International 16:5-8, 1999																																				X
Frost G, Wilding J, Beecham J: Dietary advice based on the glycaemic index improves dietary profile and metabolic control in type 2 diabetic patients. Diabet Med 11:397-401, 1994																																				X
Pratt C, Wilson W, Leklem J, Kingsley L: Peer support and nutrition education for older adults with diabetes. J Nutr Elderly 6:31-43, 1987																																				X
Hitchcock Noel P, Larme A, Meyer J, Marsh G, Correa A, Pugh J: Patient choice in diabetes education curriculum: nutritional versus standard content for type 2 diabetes. Diabetes Care 21: 896-901, 1998																																				X
Werdier D, Jesdinsky H, Helmich P: A randomized, controlled study on the effect of diabetes counseling in																																				X

For peer review only

the offices of 12 general practitioners. Rev Epidemiol Sante Publique 32:225-229, 1984																																				
Gilden J, Hendryx M, Clar S, Casia C, Singh S: Diabetes support groups improve health care of older diabetic patients. J Am Geriatr Soc 40:147-150, 1992																																				X
Laitinen J, Ahola I, Sarkkinen E, Winberg R, Harmaakorph-Livonen P, Uusitupa M: Impact of intensified dietary therapy on energy and nutrient intakes and fatty acid composition of serum lipids in patients with recently diagnosed non-insulin-dependent mellitus. J Am Diet Assoc 93:276-283, 1993																																				X
Rainwater N, Ayllon T, Frederiksen L, Moore E, Bonar J: Teaching self-management skills to increase diet compliance in diabetics. In Adherence, Compliance, and Generalization in Behavioral Medicine. Stuart R, Ed. New York, Brummer/Mazel, 1982, p. 304 -328																																				X
Wilson W, Pratt C: The impact of diabetes education and peer support upon weight and glycemic control of elderly persons with non-insulin dependent diabetes mellitus (NIDDM). Am J Public Health 77:634-635, 1987																																				X
Mazzuca K, Farris N, Mendenball J, Stoupa R: Demonstrating the added value of community health nursing for clients with insulin-dependent diabetes. J Community Health Nurs 14:211-224, 1997																																				X
Franz M, Splett F, Monk A, Barry B, McClam K, Weaker T, Upham P, Bergenstal R, Mazze R: Cost-effectiveness of medical nutrition therapy provided by dietitians for persons with non-insulin-dependent diabetes mellitus. J Am Diet Assoc 95:1018-1024, 1995																																				X
Vanninen E, Uusitupa M, Siitonen O, Laitinen J, Lansimies E: Habitual physical activity, aerobic capacity and metabolic control in patients with newly-diagnosed type 2 (non-insulin-dependent) diabetes mellitus: effect of 1-year diet and exercise intervention. Diabetologia 35:340-346, 1992																																				X
Wing R, Nowalk M, Epstein L, Koeske R: Calorie-counting compared to exchange system diets in the treatment of over-weight patients with type II diabetes. Addict Behav 11:163-168, 1986																																				X
Basch C, Walker E, Howard C, Shamoon H, Zybert P: The effect of health education on the rate of opthalmic examinations among African Americans with diabetes mellitus. Am J Public Health 89: 1878-1882, 1999																																				X
Julius U, Gross P, Hanefeld M, DIS-Group: Work absenteeism in type 2 diabetes mellitus: results of the prospective diabetes intervention study. Diabete Metab 19:202-206, 1993																																				X
Lockington T, Farrant S, Meadown K, Dowlatshahi D, Wise P: Knowledge profile and control in diabetic patients. Diabet Med 5:381-386, 1988																																				X
	53	28	17	16	132	13	33	17	20	39	10	13	16	13	8	12	26	15	7	20	10	47	35	9	NP	11	11	20	8	36	3	14	21	62	31	84

1= Choi, Davidson, Walker, Lee, & Palermo, 2016; 2=Creamer, Attridge, Ramsden, Cannings-John, & Hawthorne, 2016; 3=Huang et al., 2016; 4=Chen et al., 2015; 5=Pillay et al., 2015; 6= Terranova,

For peer review only - http://bmjopen.bmj.com/site/about/guidelines.xhtml

Brakenridge, Lawler, Eakin, & Reeves, 2015; 7= Attridge, Creamer, Ramsden, Cannings-John, & Hawthorne, 2014; 8= Odnoletkova et al., 2014; 9 = Pal et al., 2014; 10 = Ricci-Cabello et al., 2014; 11= Saffari, Ghanizadeh, & Koenig, 2014; 12=Gucciardi, Chan, Manuel, & Sidani, 2013; 13=Pal et al., 2013; 14=Vugt, Wit, Cleijne, & Snoek, 2013; 15= Amaeshi, 2012 ; 16= Nam, Janson, Stotts, Chesla, & Kroon, 2012 ; 17= Steinsbekk, Rygg, Lisulo, Rise, & Fretheim, 2012; 18= Burke et al., 2011; 19= Kai Lun Gan, Brammer, & Creedy, 2011; 20=Ramadas, Quek, Chan, & Oldenburg, 2011; 21=Hawthorne, Robles, Cannings-John, & Edwards, 2010; 22= Minet, Moller, Vach, Wagner, & Henriksen, 2010; 23=Alam, Sturt, Lall, & Winkley, 2009; 24= Duke, Colagiuri, & Colagiuri, 2009; 25= Fan & Sidani, 2009; 26=Hawthorne, Robles, Cannings-John, & Edwards, 2008 ; 27=Khunti, Camosso-Stefinovic, Carey, Davies, & Stone, 2008; 28=Loveman, Frampton, & Clegg, 2008 ; 29=Wens et al., 2008; 30 = Nield et al., 2007; 31=Zabaleta & Forbes, 2007; 32=Deakin, McShane, Cade, & Williams, 2005; 33=Vermeire et al., 2005; 34=Gary, Genkinger, Guallar, Peyrot, & Brancati, 2003; 35=Norris, Lau, Smith, Schmid, & Engelgau, 2002; 36=Norris, Engelgau, & Narayan, 2001. T2DM = type 2 diabetes mellitus; NP = not applicable.

% overlap 177/532= 33.3%

CA= Covered Area =

(53+28+17+16+132+13+33+17+20+39+10+13+16+13+8+12+26+15+7+20+10+47+35+9+11+11+20+8+36+3+14+21+62+31+84)/(35x532) = 910/18620=4.8% (one review did not report the included studies list)

CCA= Corrected Covered Area = (910-532)/ (18620-532) = 378/18088= 2.1% (slight)

For peer review only

Health Education for Patients with Acute Coronary Syndrome and Type 2 Diabetes Mellitus: An Umbrella Review of Systematic Reviews and Meta-Analyses

Journal:	*BMJ Open*
Manuscript ID	bmjopen-2017-016857.R2
Article Type:	Research
Date Submitted by the Author:	07-Jul-2017
Complete List of Authors:	Liu, Xian-liang<br>Shi, Yan<br>Willis, Karen<br>Wu, Jo<br>Johnson, Maree; Australian Catholic University, Faculty of Health Sciences
**Primary Subject Heading**:	Evidence based practice
Secondary Subject Heading:	Cardiovascular medicine, Diabetes and endocrinology
Keywords:	Health Education, Acute Coronary Syndrome, Type 2 Diabetes Mellitus, Umbrella Review

SCHOLARONE™ Manuscripts

For peer review only

Title page

Health Education for Patients with Acute Coronary Syndrome and Type 2 Diabetes Mellitus: An Umbrella Review of Systematic Reviews and Meta-Analyses

Xian-Liang Liu, RN, MScN

Ph.D. Candidate, Tenth People's Hospital of Tongji University, China;

Ph.D. Candidate, School of Nursing, Midwifery and Paramedicine, Australian Catholic University, Australia;

Lecturer, School of Nursing, Jinggangshan University, China.

Address: 1100 Nudgee Road, Banyo, Brisbane, QLD, Australia

Email: liu.xianliang@myacu.edu.au; 1988_liuxianliang@tongji.edu.cn

Yan Shi, RN, Ph.D., Professor, Executive Director of Nursing Department

Tenth People's Hospital and Tongji University, China

Address: 301, YanChang Road, Shanghai, China

E-mail: shlzk2002@163.com

Karen Willis, Ph.D., Professor, Associate Dean (Learning and Teaching)

Faculty of Health Sciences, Australian Catholic University, Australia

Address: 250 Victoria Parade, Melbourne, Australia

Email: Karen.Willis@acu.edu.au

Chiung-Jung (Jo) Wu, RN, DrHlthSc, FACN

Associate Professor, School of Nursing, Midwifery and Paramedicine, University of the Sunshine Coast;

Visiting Fellow, School of Nursing, Queensland University of Technology;

Honorary Research Fellow, Royal Brisbane and Women's Hospital;

For peer review only

Honorary Research Fellow, Mater Medical Research Institute-University of Queensland (MMRI-UQ), Brisbane, Australia;

Postal Address: ML 57, PO Box 1149, Hervey Bay Q 4655, Australia.

Email: cwu@usc.edu.au; c3.wu@qut.edu.au

Maree Johnson, RN, Ph.D., Professor, Associate Dean (Research)

Faculty of Health Sciences, Australian Catholic University, Australia

Address: Tenison Woods House (8 -20 Napier Street), North Sydney, Australia

Email: Maree.Johnson@acu.edu.au

Affiliated with the Ingham Institute of Applied Medical Research, Liverpool. Sydney.

Corresponding author:

Xian-Liang Liu, RN, MScN,

Ph.D. Candidate, School of Nursing, Midwifery and Paramedicine, Australian Catholic University, Australia; Ph.D. Candidate, Tenth People's Hospital of Tongji University, China; Lecturer, School of Nursing, Jinggangshan University, China.

Address: 1100 Nudgee Road, Banyo, Brisbane, QLD, Australia

Email: liu.xianliang@myacu.edu.au; 1988_liuxianliang@tongji.edu.cn

Tel: +61 497440830; +86 15921496130

For peer review only

Abstract

Objectives: This umbrella review aimed to identify the current evidence on health education-related interventions for patients with acute coronary syndrome or type 2 diabetes mellitus; identify the educational content, delivery methods, intensity, duration and setting required. The purpose was to provide recommendations for educational interventions for high risk patients with both acute coronary syndrome and type 2 diabetes mellitus.

Design: Umbrella review of Systematic Reviews and Meta-Analyses.

Setting: Inpatient and post discharge settings.

Participants: Patients with ACS and T2DM.

Data sources: CINAHL, Cochrane Library, Joanna Briggs Institute, Journals@Ovid, EMBase, Medline, PubMed, and Web of Science databases from January 2000 through May 2016.

Outcomes measures: Clinical outcomes (such as HbA1C), behavioral outcomes (such as smoking), psychosocial outcomes (such as anxiety) and medical service use.

Results: Fifty-one eligible reviews (15 for ACS and 36 for T2DM) consisting of 1324 relevant studies involving 288,057 patients (15 papers did not provide the total sample); 30 (58.8%) reviews were rated as high quality. Nurses only and multidisciplinary teams were the most frequent professionals to provide education, and most educational interventions were delivered post-discharge. Face-to-face sessions were the most common delivery formats, and many education sessions were also delivered by telephone or via web contact. The frequency of educational sessions was weekly or monthly, and an average of 3.7 topics was covered per education session. Psychoeducational interventions were generally effective at reducing smoking and admissions for patients with ACS. Culturally appropriate health education, self-management educational interventions, group medical visits and psychoeducational interventions were

For peer review only - http://bmjopen.bmj.com/site/about/guidelines.xhtml

generally effective for T2DM patients.

Conclusions: Results indicate that there is a body of current evidence about the efficacy of health education, its content, and delivery methods for patients with ACS or T2DM. These results provide recommendations about the content for, and approach to, health education intervention for these high risk patients.

KEY WORDS: Health Education; Acute Coronary Syndrome; Type 2 Diabetes Mellitus; Umbrella Review

Strengths and limitations of this review:

- This umbrella review is the first synthesis of systematic reviews or meta-analyses to consider health education-related interventions for patients with ACS or T2DM.
- These results provide recommendations about the content of a health education intervention for patients with ACS and T2DM.
- The diversity of the educational interventions seen in the reviews included in this umbrella review may reflect the uncertainty about the optimal strategy for providing health education to patients.
- This umbrella review found no reviews focused on patients with ACS and T2DM the intended target group; instead, all of the systematic reviews and meta-analyses focused on only one of these two diseases.

For peer review only - http://bmjopen.bmj.com/site/about/guidelines.xhtml

Introduction

Acute coronary syndrome (ACS) is the leading cause of death worldwide. The risk of high mortality rates relating to ACS is markedly increased after an initial cardiac ischemic event.[1] Globally, 7.2 million (13%) deaths are caused by coronary artery disease (CAD),[2] and it is estimated that more than 780,000 persons will experience ACS each year in the United States. [3] Moreover, about 20–25% of patients with ACS reportedly also have diabetes mellitus (DM); predominantly type 2 diabetes mellitus [T2DM]). [4 5] Patients with ACS and DM have an increased risk of adverse outcomes such as death, recurrent myocardial infarction (MI), readmission, or heart failure during follow up. [6] Longer median delay times from symptom onset to hospital presentation, have been reported among patients with ACS and DM than patients with ACS alone. [7]

DM is now considered to confer a risk equivalent to that of CAD for patients for future MI and cardiovascular mortality. [8] Mortality was significantly higher among patients with ACS and DM than among patients with ACS only following either ST segment elevation myocardial infarction (STEMI) (8.5% [ACS and DM] versus 5.4% [ACS]) or unstable angina/non-STEMI (NSTEMI) (2.1% [ACS and DM] vs. 1.1% [ACS]).[9] ACS and T2DM are often associated with high-risk factors such as low levels of physical exercise, obesity, smoking, and unhealthy diet. [10] Some of these and other risk factors, specifically glycemia, high blood pressure (BP), lipidemia, and obesity, are frequently addressed by health education interventions. [10]

Health education interventions are comprehensive programs that healthcare providers deliver to patients aimed at improving patients' clinical outcomes through the increase and maintenance of health behaviours.[11] Along with education about, for example, medication taking, these programs seek to increase behaviors such as physical exercise and a healthy diet thus reducing

For peer review only - http://bmjopen.bmj.com/site/about/guidelines.xhtml

patient morbidity or mortality. [11] Most diabetes education is provided through programs within outpatient services or physicians' practices. [12] Many recent education programs have been designed to meet national or international education standards [13-15] with diabetes education being individualized to consider patients' existing needs and health conditions. [16] Patients with T2DM have reported feelings of hopelessness and fatigue with low levels of self-efficacy, after experiencing an acute coronary episode. [17]

Although there are numerous systematic reviews of educational interventions relating to ACS, or T2DM, an umbrella review providing direction on educational interventions for high risk patients with both ACS and T2DM is not available, indicating a need to gather the current evidence and develop an optimal protocol for health education programs for patients with ACS and T2DM. This umbrella review will examine the best available evidence on health education-related interventions for patients with ACS or T2DM. We will synthesize these findings to provide direction for health education-related interventions for high risk patients with both ACS and T2DM.

An umbrella review is a new method to summarize and synthesize the evidence from multiple systematic reviews/meta-analyses into one accessible publication. [18] Our aim is to systematically gather, evaluate and organize the current evidence relating the health education interventions for patients with ACS or T2DM, and proffer recommendations for the scope of educational content and delivery methods that would be suitable for patients with ACS and T2DM.

Methods

Data Sources

For peer review only - http://bmjopen.bmj.com/site/about/guidelines.xhtml

This umbrella review performed a literature search to identify systematic reviews and meta-analyses examining health education-related interventions for patients with ACS or T2DM. The search strategies are described in Appendix 1. This umbrella review searched eight databases for articles published from January 2000 to May 2016: CINAHL, Cochrane Library, Joanna Briggs Institute, Journals@Ovid, EMBase, Medline, PubMed, and Web of Science. The search was limited to English language only. The following broad MeSH terms were used: *acute coronary syndrome; angina, unstable; angina pectoris; coronary artery disease; coronary artery bypass; myocardial infarction; diabetes mellitus, type 2; counseling; health education; patient education as topic; meta-analysis [publication type];* and *meta-analysis* as a topic.

Inclusion Criteria

Participants

All participants were diagnosed with ACS or T2DM using valid, established diagnostic criteria. The diagnostic standards included those described by the American College of Cardiology or American Heart Association, [3] National Heart Foundation of Australia & Cardiac Society of Australia and New Zealand, [19] World Health Organization, [20] or other associations.

Intervention Types

For this umbrella review, health education-related interventions refer to any planned activities or programs that include behavior modification, counseling, and teaching interventions. Results considered for this review included changes in clinical outcomes (including blood pressure levels, body weight, diabetes complications, HbA1C, lipid levels, mortality rate and physical activity levels), behavioral outcomes (such as diet, knowledge, self-management skills, self-efficacy, and smoking), and psychosocial outcomes (such as anxiety, depression, quality of life, and stress) and medical service use (such as medication use, health care utilization, and cost-

For peer review only - http://bmjopen.bmj.com/site/about/guidelines.xhtml

effectiveness) for patients with ACS or T2DM. These activities or programs included any educational interventions delivered to patients with ACS or T2DM. The interventions are delivered in any format, including face-to-face, telephone and group-based or one-on-one, and the settings include community, hospital and home. The interventions were delivered by nurses (including diabetes nurse educators), physicians, community health care workers, dietitians, lay people, rehabilitation therapists, or multidisciplinary teams.

Study Types

Only systematic reviews and meta-analyses were included in this review.

Eligibility Assessment

The title and abstract of all of the retrieved articles were assessed independently by two reviewers (XL-L, YS) based on the inclusion criteria. All duplicate articles were identified within EndNote version X7 [21] and subsequently excluded. If the information from the titles and abstract was not clear, the full articles were retrieved. The decision to include an article was based on an appraisal of the full text of all retrieved articles. Any disagreements during this process were settled by discussion and, if necessary, consensus was sought with a third reviewer. We developed an assessment form in which specific reasons for exclusion were detailed.

Assessment of Methodological Quality

The methodological quality and risk of bias were assessed for each of the included publications using the Assessment of Multiple Systematic Reviews (AMSTAR),[22] independently by the same two reviewers (see Table 1). The AMSTAR is an 11-item tool, with each item provided a score of 1 (specific criterion is met) or 0 (specific criterion is not met, unclear, or not applicable). [22 23] An overall score for the review methodological quality is then calculated as the sum of the individual item scores: high quality, 8-11; medium quality, 4-7; or low quality, 0-3. [23] If the

For peer review only - http://bmjopen.bmj.com/site/about/guidelines.xhtml

required data were not available in the article, the original authors were contacted for more information. The low quality reviews (AMSTAR scale: 0-3) were excluded in this umbrella review.

Data Extraction

Data were independently extracted by two reviewers using a predefined data extraction form. For missing or unclear information, the primary authors were contacted for clarification.

Statistical Presentation of Results from Reviews

All of the results were extracted for each included systematic review or meta-analysis, and the overall effect estimates are presented in a tabular form. The number of systematic reviews or meta-analyses that reported the outcome, total sample (from included publications), and information of health education interventions is also presented in the Tables (Table 2, 3). [24] A final 'summary of evidence' was developed to present the intervention, included study synthesis, and indication of the findings from the included papers (Table 4). [24] This umbrella review calculated the Corrected Covered Area (CCA) (Appendix 2, 3). The CCA statistic is a measure of overlap of trials (the repeated inclusion of the same trial in subsequent systematic reviews included in an umbrella systematic review). A detailed description of the calculation is provided by the authors who note slight CCA as 0-5%, moderate CCA as 6-10%, high CCA as 11-15% and very high CCA is more than 15%. [25] The lower the CCA the lower the likelihood of overlap of trials included in the umbrella review.

Synthesizing the Results and Rating the Evidence for Effectiveness

The statements of evidence were based on a rating scheme to gather and rate the evidence across the included publications.[26] The statements of evidence were based on the following rating

For peer review only - http://bmjopen.bmj.com/site/about/guidelines.xhtml

scheme: *sufficient evidence*, sufficient data to support decisions about the effect of the health education-related interventions. [26] A rating of *sufficient evidence* in this review is obtained when systematic reviews or meta-analyses with a large number of included articles or participants produce a statistically significant result between the health education group and the control group. [26] *Some evidence*, is a less conclusive finding about the effects of the health education-related interventions[26] with statistically significant findings found in only a few included reviews or studies. *Insufficient evidence*, refers to not enough evidence to make decisions about the effects of the health education-related interventions, such as non-significant results between the health education group and the control group in the included systematic reviews or meta-analyses. [26] *Insufficient evidence to determine*, refers to not enough pooled data to be able to determine whether of the health education-related interventions are effective or not based on the included reviews. [26]

Results

Characteristics of Included reviews

The selection process and number of studies at each step was illustrated as presented in Figure 1. The database search yielded 692 publications, with removal of 197 duplicates and 371 articles that did not meet the inclusion criteria, 124 full-text articles were retrieved after applying the methodological quality rating (AMSTAR scale), and three studies [27-29] were removed due to low scores ≤3 on the AMSTAR scale. Fifty-one systematic reviews or meta-analyses [30-80] conducted between 2001 and 2016 and published in English were included (Figure 1; Tables 1 –3); 15 relating to ACS. The overlap of the trials included in the 15 reviews and meta-analyses related to ACS was slight (CCA = 2.6%). For the 36 systematic reviews relating to T2DM, the overlap of trials within these 35 reviews and meta-analyses (one review [47] did not report the included

For peer review only - http://bmjopen.bmj.com/site/about/guidelines.xhtml

studies) was slight (CCA = 2.1%). None of the articles included patients with both ACS and T2DM. The umbrella review involved a total of 277,493 patients, including 225,034 patients with coronary heart disease or ACS (one article did not report the total sample) and 52,459 patients with T2DM (16 papers did not report the total sample). The average sample size of included articles was 8,161 (range, 536–68,556) participants, however, 63 studies related to ACS and 177 studies related to T2DM, were included in more than one systematic review or meta-analysis (see Appendix 2 and 3 and CCA statistics). The sample of these studies would therefore be included more than once. Of the included systematic reviews or meta-analyses, eleven were published in *The Cochrane Library*. Nine of the articles described meta-analyses, 29 articles described systematic reviews, and the remaining 13 articles were described as systematic reviews and meta-analyses or meta-regressions or narrative reviews.

Electronic database searches were conducted for all systematic reviews or meta-analyses, with an average of 6 databases searched (range, 2–16). The dates searched ranged widely, from inception of the database through December 2014. Most of the included reviews were randomized controlled trials (RCTs), and an average of 25.6 (range, 7–132) studies was included per systematic review or meta-analyses. Of the total, 818 unique (non-repeated) studies were included in all of the reviews or meta-analyses, 286 included patients with ACS, and 532 included patients with T2DM (Appendix 2, 3). The included reviews assessed the risk of bias using the Cochrane risk of bias tool (22 publications), JADA quality score (seven publications), Joanna Briggs quality assessment tool (two publications), PEDro scale (one publication), RCT Critical Appraisal Skills Programme (one publication), and the SIGN-50 checklist (one publication).

Methodological Quality of Included Systematic Reviews and Meta-Analyses

For peer review only - http://bmjopen.bmj.com/site/about/guidelines.xhtml

For peer review only

The methodological quality of the included publications is presented in Table 1. Thirty (58.8%) publications were classified as high quality (scores 8–11), and 21 (41.2%) publications were classified as medium quality (scores 4–7). Twenty five (49%) reviews specifically provided an *a priori* design, while the use of such a design was unclear for 26 (51%) publications. The inclusion of other forms of literature (such as grey literature) was described in 18 (35%) reviews. Only 14 out of 51 (27%) reviews included a table of included and excluded studies. Only 2 (4%) reviews did not provide a characteristics table of the included papers. The scientific quality of the included papers was evaluated and documented in 47 (92%) reviews. The scientific quality of the included studies was used appropriately to formulate conclusions in 47 (92%) reviews. The methods to combine the results of the included studies were appropriate in 43 (86%) reviews. Publication bias was assessed in only 19 (37%) reviews. Finally, conflicts of interest were reported in 47 (92%) reviews.

Characteristics of Health Educational Interventions

The description of the health educational interventions followed the Workgroup for Intervention Development and Evaluation Research (WIDER) reporting guidelines for behavior change interventions. [81] The characteristics of the recipients, setting, delivery methods, intensity, duration, and educational content of health educational interventions for patients with ACS or T2DM are summarized in Tables 2 and 3. The delivery strategies for health education included face-to-face, internet-based, phone-based, videotape, written educational materials, or mixed. The format included one-on-one (individualized), group, or both. Face-to-face sessions were the most common delivery formats, and many education sessions were also delivered by telephone/web contact or individualized counseling. The number of sessions, total contact hours, and durations varied, and there was limited information about the intensity of health education

For peer review only - http://bmjopen.bmj.com/site/about/guidelines.xhtml

for patients provided. The frequency of educational sessions was weekly or monthly, and an average of 3.7 topics was covered per education session. Nurses and multidisciplinary teams were the most frequent educators, and most education programs were delivered post-discharge.

Acute Coronary Syndrome

The educational content for patients with ACS covered cardiovascular risk factors in 8 reviews (53.33%), psychosocial issues in 8 reviews (53.33%), smoking cessation in 6 reviews (40.00%), exercise in 5 reviews (33.33%), behavioral change in 5 reviews (33.33%), diet in 4 reviews (26.67%), self-management in 3 reviews (20.00%), and medication in 1 review (6.67%). Two reviews only included smoking cessation and cardiovascular risk factors. The most common educational providers were nurses and a multidisciplinary team. Six studies [31 36 48 51 56 69] (6/15, 40%) described the theoretical approach that underpinned the education intervention.

Type 2 Diabetes Mellitus

The educational content for patients with T2DM included diet in 23 reviews (63.89%), behavioral change in 21 reviews (58.33%), self-management in 20 reviews (55.56%), exercise in 17 reviews (47.22%), glycemic regulation in 16 reviews (44.45%), medication in 13 reviews (36.11%), psychosocial issues in 9 reviews (25.00%), smoking cessation in 2 reviews (5.56%), cardiovascular risk factors in 2 reviews (5.56%), and DM risks in one review (2.78%). The most common providers were dieticians, nurses, and a multidisciplinary team. The number of sessions, total contact hours, and durations varied. Thirteen reviews [30 33 43 49 52-54 60 64 67 75-77] (13/36, 36.11%) described the theoretical approach that underpinned the education intervention.

Effect of Interventions

The outcomes of the included systematic reviews and meta-analyses were summarized in Table

For peer review only - http://bmjopen.bmj.com/site/about/guidelines.xhtml

4.

Patients with Acute Coronary Syndrome

Three major types of health education-related interventions were used for patients with ACS: general health education (only included general health information), psychoeducational interventions, and secondary prevention educational interventions (including strategies to promote a healthy lifestyle, manage medications, and reduce cardiovascular complications) as well as internet-based interventions.

General Health Education

The findings are based on our synthesis of the findings from 6 systematic reviews. [37 48 50 51 59 70] Overall, there were mixed effects of general health education on behavioral change or clinical outcomes in patients with ACS. There was *some evidence* of a positive effect of general health education on knowledge, behavior, psychosocial indicators, beliefs, and risk factor modification, but no effects for key clinical outcomes, such as cholesterol level, hospitalization, mortality, MI, and revascularization. The results for health-related quality of life, healthcare utilization, and costs were mixed; several reviews reported a significant change, and other reviews reported no significant change for these outcomes. Only one review focused on telephone-based health education. There is *some evidence* that telephone-based health education during cardiac rehabilitation might improve all-cause hospitalization, anxiety, depression, smoking cessation, and systolic BP but there is no evidence for improvements in all-cause mortality and reductions in low-density lipoprotein cholesterol. [59]

Psychoeducational Interventions

Strategies for psychoeducational interventions have a specific focus on smoking cessation, and

For peer review only - http://bmjopen.bmj.com/site/about/guidelines.xhtml

depression. The findings are based on synthesis of results from six publications. [31 35 36 45 56 69] There is *sufficient evidence* that psychoeducational programs are effective at decreasing smoking, achieving smoking abstinence, and reducing depression. One review reported no effect on smoking cessation [31] or total mortality [56].

Secondary Prevention Educational Interventions

The following statements are based on our synthesis of results from three papers. [34 41 44] There is *some evidence* that secondary prevention educational interventions reduce MI readmission rates and improve quality of life, but the intervention was ineffective in reducing revascularization, cholesterol levels, and improving smoking cessation rates. The results are mixed for mortality and re-infarction rates; two reviews [34 41] found positive effects on mortality, while one review [44] did not.

Patients with Type 2 Diabetes Mellitus

Ten types of health education-related interventions were used for patients with T2DM: culturally appropriate health education (tailored to the religious beliefs, culture, literacy, and linguistics of the geographical area), dietary advice, foot health education, group medical visits (a group education component taught by health professionals), general health education (only included general health information), improving the uptake and maintenance of medication regimes (e.g., promoting the use of oral hypoglycemic medications), lifestyle interventions (specific focus on dietary changes and increased physical activity, or stress management), psychoeducational interventions and self-management educational interventions (activities that promote or maintain the behaviors to manage T2DM often based on the National Standards for Diabetes Self-Management Education [13]), and therapeutic education (collaborative process needed to modify behavior and more effectively manage risk factors).

For peer review only - http://bmjopen.bmj.com/site/about/guidelines.xhtml

Culturally Appropriate Health Education

Findings are based on our synthesis of results from 8 publications. [33 42 52-54 58 62 72] Overall, there was *some evidence* of the effects of culturally appropriate health education on clinical outcomes for T2DM. There was *sufficien*t evidence that culturally appropriate health education improves HbA1c reduction and knowledge scores. There is *some* evidence that physical activity and clinical outcomes (blood glucose, HbA1c, BP) were improved. There were no data relating to adverse events during the intervention and follow-up (such as hypoglycemic events and mortality), and there was insufficient evidence about improvements in quality of life.

General Health Education

The statements are based on our synthesis of results from five papers. [40 46 60 74 79] Overall, there were mixed effects of general health education programs on clinical outcomes for T2DM, including HbA1C, cholesterol level, and triglyceride level. There was *some evidence* of the effectiveness of general health education on the management of glycemia, weight reduction, and some diabetes management outcomes (HbA1C, diabetes complications). There were no data supporting the effectiveness of general health education on reduced health service utilization, diabetes complications, improved knowledge, psychosocial outcomes, or smoking habits.

Lifestyle Interventions

The following statements are based on our synthesis of results from 6 reviews. [39 49 55 71 72 77] Overall, there were mixed effects of the lifestyle interventions on cholesterol level, HbA1C level, and body weight. There is *some evidence* that lifestyle interventions or behavioral programs are effective for blood glucose and BP management, but they were ineffective for reductions in HbA1c scores [71 72].

For peer review only - http://bmjopen.bmj.com/site/about/guidelines.xhtml

Uptake and maintenance of medication regimes

The statements are based on our synthesis of results from three publications. [57 78 80] There is *some evidence* of the effectiveness of increased uptake and maintenance of medication regimes for taking medications for HbA1C regulation including oral hypoglycemic agents.

Self-Management Educational Interventions

The statements are based on our synthesis of results from 9 reviews. [43 47 61 64 65 67 68 75 76] Overall, there was *sufficient evidence* of the effects of self-management education interventions on HbA1c level, knowledge, lifestyle outcomes, and main psychosocial outcomes. However, there was *insufficient* evidence of the benefits of this education intervention on depression, quality of life, and body weight.

Other Health Education-Related Interventions

Other health education-related interventions for patients with T2DM included therapeutic education, foot health education, group medical visits, psychoeducational interventions, and dietary advice. Statements for all of these interventions are based on our synthesis of results from one review.

There is *some evidence* that foot health education is effective in reducing the incidence of lower extremity amputation. [32] There is *some evidence* that group medical visits are effective for improving HbA1c and systolic BP management [38]. There is also *some evidence* that psychoeducational programs are effective for improving HbA1c regulation and psychological status. [30]

Finally, there is *insufficient evidence* that dietary advice improves glycemic and weight management or reduces micro- and macrovascular diseases. [63] There is also *insufficient*

For peer review only - http://bmjopen.bmj.com/site/about/guidelines.xhtml

evidence for the cost-effectiveness of therapeutic education for patients with T2DM. [66]

Discussion

This umbrella review identified 51 systematic reviews or meta-analyses (15 for ACS and 36 for T2DM) that assessed the outcomes of various aspects (such as the duration, contact hours, educational content, delivery mode) of the delivery of health education-related interventions relevant to high risk patients with ACS and T2DM. Health education has become an integral part of the management for people with ACS and T2DM. The most appropriate focus of the education provided to ACS patients with T2DM remains largely undefined in the literature. For example, it remains unknown if the focus should be primarily on cardiovascular risk factors, blood glucose monitoring, or all educational components for patients with both conditions. [70 76] In addition, should cardiovascular risk factors be the focus during the acute inpatient stay with other educational needs such as the smoking cessation occurring within the primary care or outpatient settings. [31 69 70]

It remains challenging to determine the specific strategy or format that is the most effective delivery mode for patients with ACS or T2DM. There is very limited evidence to guide clinicians on the duration, contact hours, educational content, delivery mode, total length, and setting of health education programs for cardiac patients. [50] For patients with DM, one study reported that more successful programs were longer than 6 months (longer duration), consisted of greater than 10 contact sessions (high intensity), and were one-on-one sessions with individualized assessment. [82]

Use of Theoretical Orientation to Develop Educational Intervention

For patients with Acute Coronary Syndrome

For peer review only - http://bmjopen.bmj.com/site/about/guidelines.xhtml

Use of theory when designing behavioral change interventions may also influence effectiveness.[75] Health education using a cognitive behavioral strategy is most consistently effective in changing maladaptive illness beliefs, [51] and studies using more than 2 behavioral change strategies reported significant differences between the intervention and control groups. [31] In one review, a significant change in smoking cessation was not observed in subgroup analyses between studies that did or did not report using a theory in intervention planning; [56] however, the authors did not suggest that using a theory in program planning should be disregarded but reported that examining actual theories or mechanisms underlying health education programs is required. [56] Owing to the considerable overlap between different theories and the detailed description of the theoretical approach in only approximately 40% of the included papers, it is difficult to determine the most effective theoretical approach, but many models can be used with success, such as the health belief model (HBM), social cognitive theory (SCT) and transtheoretical model (TTM). [56 67 69 75] Three reviews [31 41 44] noted that some included studies used behavioural strategies such as goal setting. These strategies were found to be beneficial for patients with coronary heart disease.

For patients with Type 2 Diabetes Mellitus

Although the theoretical approach underpinning the health education program was not always described, 13 of the 36 reviews (36.11%) related to T2DM reported the theoretical approach used in their included studies. The most common theories were SCT (including self-efficacy), empowerment theories (eg., Empowerment Behavior Change Model, Self-determination and Autonomy Motivation Theory, Middle-range Theory of Community Empowerment) and TTM. There is evidence that health education interventions based on a theoretical model are likely to be effective. [43] Vugt et al suggested that self-care education programs should be based on

For peer review only - http://bmjopen.bmj.com/site/about/guidelines.xhtml

theories and that theory-based self-care interventions are more effective than non-theory-based programs. [75 83] Theories could help to specify the key target health behaviors and behavioral change techniques required to generate the desired outcomes. [75] The decision regarding the theory should be based on the aim of the program and factor for intervention. [77] Only one review reported that a theoretical approach underpinning the health education program is not necessary for better outcomes. [76] Fourteen reviews [30 33 40 46 52 57 60 63 64 67 68 73 75 77] reported that goal setting was conducted in the included studies. Goal setting by patients, health professionals or mutually-agreed goals were linked to improved patient outcomes.

Educational Content

For Patients with Acute Coronary Syndrome

Most reviews reported that the educational content of the interventions was comprehensive. The most common topics, of the average 3.7 topics per education session, were behavioral change, cardiovascular risk factors management, exercise, psychosocial issues, and smoking cessation. An underlying principle of health education for patients with ACS is that knowledge is necessary, but not enough to develop health behaviors and change risk factors. [31 50] Age, cognitive factors, environmental factors, and social and economic background are also important considerations. [50] While interventions using a behavioral program, telephone-based content, or self-care are effective for smoking cessation, there was insufficient evidence to support that any type of educational program was more efficacious than the others. [69] Psychoeducation, which is defined as multimodal, educationally based, self-management interventions,[31] led to enhanced physical activity levels within 6–12 months when added to cardiac rehabilitation (CR) and was more effective than an exercise program or health education alone. [31 56] Moreover, psychoeducational interventions were more effective for patients with ACS than other types of

For peer review only - http://bmjopen.bmj.com/site/about/guidelines.xhtml

health education. [31 56]

For Patients with Type 2 Diabetes Mellitus

The educational content for patients with T2DM focused more on behavioral change, diet, exercise, glycemic regulation, medication, and self-management. Health education that was self-management was more effective for patients with T2DM. [40 47] In addition, based on the current evidence, the educational content should be culturally sensitive, especially for patients with T2DM; [33 42 54] culturally appropriate diabetes health education may have a greater impact on the management of glycemia and reduce diabetes complications. [77] The educational interventions for patients with T2DM focused primarily on HbA1C, lipid levels, quality of life, and body weight. HBM and SCT were the most common theories used in the included reviews.

Teaching Strategies and Outcomes

For Patients with Acute Coronary Syndrome

Most reviews reported that the education was provided using multiple teaching methods and in multiple settings. Nurses and multidisciplinary teams were the most frequent people providing education, and most education programs were delivered post-discharge. Although face-to-face sessions were the most common delivery format, many education sessions were also delivered by telephone or through individualized counseling. Telephone-based health education appeared to be effective for reducing hospitalizations, systolic BP, smoking rates, depression, and anxiety. [59] The educational interventions for patients with ACS focused primarily on clinical outcomes (hospitalization and mortality), modifiable risk factors (BP, low-density lipoprotein levels, and smoking cessation), and other psychological outcomes (anxiety and depression).

For Patients with Type 2 Diabetes Mellitus

For peer review only - http://bmjopen.bmj.com/site/about/guidelines.xhtml

Mixed health education programs generally included group sessions combined with educator-facilitated individual sessions, covering basic knowledge and problem-solving skills. These programs produced greater benefits and larger effect sizes for blood glucose reduction and knowledge levels in patients with T2DM. [47] In contrast, individual education programs have been reported as more effective in achieving outcomes than group-based education. This may be because education programs might be more efficient at addressing personal needs, with greater participant engagement. [73] However, one systematic review reported that individual and group patient education, demonstrated similar outcomes among T2DM patients. [46]

Although face-to-face sessions were the most common delivery format, many education sessions were also delivered by telephone or individualized counseling. Face-to-face health education programs were most effective for enhancing blood glucose regulation and knowledge levels, while mixed delivery models (face-to-face, phone contact, online or web-based, or video) produced a moderate effect for knowledge levels. [47] Another review reported that face-to-face health education programs generated a greater benefit for metabolic management than those delivered using electronic communication technology. [73]

Nurses (including diabetes nurses educators), community workers, dieticians and multidisciplinary teams were the most frequent educators, and most of the education programs were delivered post-discharge. Some reviews indicated that health education programs delivered by a group of different educators, with some degree of education reinforcement at additional points of contact, may provide the best results. [60 76] However, based on two studies that reported HbA1C at 12 months, it is indicated that the outcomes in studies with only a diabetes nurse as the educator also tended to do better than the outcomes in studies with a multidisciplinary team , while the biggest effect was seen when a dietician was the only educator. [76] Health education

For peer review only - http://bmjopen.bmj.com/site/about/guidelines.xhtml

programs delivered by one person may focus more on the patient's ability than the educational content or quality of the health education programs. [76] However, no clear conclusion can be drawn whether having one educator delivering the intervention is best due to few information. [60]

Delivery, Timing and Follow up

For Patients with Acute Coronary Syndrome

Most educational sessions were delivered weekly. Few reviews provided information regarding the duration of education interventions; when the duration was reported, it varied from 4 weeks to 48 months. These findings suggest that there is a significant gap in the evidence in relation to the duration, contact hours, educational content, optimal delivery mode, total length, and setting of health education programs for cardiac patients. [50] For patients with ACS, one systematic review that included 7 studies with a total of 536 participants reported that studies with education lasting at least 6 months resulted in the most significant changes in the primary outcomes (such as behavioral change, smoking cessation) [31] and that at least 12 months of follow-up is needed to evaluate the impact of telephone-based education. [59] Another review reported that the intensity of education programs is important for efficacy regarding smoking cessation: interventions with a very low intensity and brief interventions, do not have a significant effect, [69] and programs for smoking cessation among patients with coronary heart disease should last >1 month. [69] Most of the reviews were provided for patients with ACS in inpatient settings and then within post discharge settings, five reviews [31 36 45 48 59] did not explicitly state the settings in which the health education related interventions were provided.

For Patients with Type 2 Diabetes Mellitus

Education sessions were delivered weekly or monthly. Longer health education programs for T2DM (>6 months) produced larger effects for all primary outcomes (such as HbA1C). [47]

For peer review only - http://bmjopen.bmj.com/site/about/guidelines.xhtml

Health education lasting >3 months resulted in the largest effect size compared with health education of a shorter duration (<3 months). [33] For HbA1C, the effect size at 6 months seemed to be significantly greater than at 3 and 12 months; in other words, the effect size peaked at 6 months. [62] In general, health education of a greater intensity (longer duration and more sessions) was more effective for blood glucose reduction and knowledge levels among patients with T2DM.[47 74] Compared with health education programs covering only one topic, programs that included multiple or mixed educational topics yielded consistently greater benefits in blood glucose reduction and knowledge levels. [47] In addition, health education programs combined with specific behavioral change strategies (such as self-care strategies) seemed more effective than other programs. [47] Health education related interventions were mainly delivered in hospital settings, primary care settings, diabetes centers or community based settings; although six reviews [32 39 55 58 67 72] did not explicitly state the delivery settings.

Recommendations about Health Education Interventions for Patients with ACS and T2DM

These results from included systematic reviews and meta-analyses help to provide recommendations about the content of a health education intervention for patients with ACS and T2DM, requiring further evaluation. Future development of educational programs for patients with ACS and T2DM by health care professionals should consider the needs of people with these diseases. [37 40 42 70] Based on the results and findings from this umbrella review, recommendations are made in Table 5. The acute life-threatening nature of ACS requires that increased emphasis should be placed on cardiovascular risk factors in any combined education program. Both ACS and T2DM have common lifestyle factors such as inactivity and high fat diet requiring modifications.

Overall Completeness and Applicability of Evidence

For peer review only - http://bmjopen.bmj.com/site/about/guidelines.xhtml

This overview potentially provides an estimate with the lowest level of bias for the impact of health education-related interventions for patients with ACS or T2DM and could be regarded as an all-inclusive summary of the current evidence base for health education for these patients. While this umbrella review identified evidence for each of the types of health education, there was only a small number of reviews within some categories (such as psychoeducational intervention [30] and dietary advice [63]), and these studies were not very informative. This umbrella review also found no reviews that systematically analyzed varying doses of health education; therefore, could not examine the dose-response effects. There was insufficient information about the evaluated doses (total contact hours and duration of education) to enable comparison of the benefits of differences in the magnitude of the doses across the different research. This umbrella review found no reviews focused on patients with ACS and T2DM; instead, all of the systematic reviews and meta-analyses focused on only one of these diseases.

Quality of the Evidence

The methodological quality of the included systematic reviews and meta-analyses varied. All of the included reviews or meta-analyses were of moderate to high methodological quality, as assessed using AMSTAR. However, only 30 (58.8%) systematic reviews or meta-analyses were rated as high quality and only three (5.9%) systematic reviews or meta-analyses [43 53 69] adequately met all 11 AMSTAR criteria. This indicates that some of the reviews included in this umbrella review may have limitations in their design, conduct, and/or reporting that could have influenced the findings when considered both individually and collectively. [32 65]

The quality of the primary studies in the included systematic reviews or meta-analyses also varied. The main sources of bias were inadequate reporting of allocation concealment and randomization processes, as well as lack of outcome blinding. [33 42 69 70] This bias in the

For peer review only - http://bmjopen.bmj.com/site/about/guidelines.xhtml

methodological quality led to lower quality assessments, which varied by results within each included review. Other reasons for lower methodological quality included heterogeneity in, or inconsistency of, the effect and imprecise findings. Heterogeneity between studies in this umbrella review was described in terms of the intervention, participant characteristics and length of follow-up. Heterogeneity was an important factor indicating the complexity of the health education interventions. [56] The variability in the approaches, tools or scales used to measure outcomes between the included studies are likely to introduce some heterogeneity. [30] The heterogeneity of the educational interventions seen in the reviews included in this umbrella review may reflect the uncertainty about the optimal strategy for providing health education to patients. [37] In addition, 240 studies were included more than once in the included reviews and meta-analyses. However, the overall overlap of studies among reviews and meta-analyses related ACS and T2DM was slight, CCA of 2.6% and 2.1%, respectively. [25]

This umbrella review is the first synthesis of systematic reviews or meta-analyses to take a broad perspective on health education-related interventions for patients with ACS or T2DM. Given that health education is complex, the biggest challenge for systematic reviews or meta-analyses of health education is accounting for the potential clinical heterogeneity in health education-related interventions (content and delivery approaches) and the population of patients who receive health education. To facilitate comparisons across systematic reviews of health education and the efficient future update of this umbrella review, future reviews or meta-analyses need high-quality research and to standardize their design and reporting, including the reporting of included study characteristics, assessment criteria for risk of bias, outcomes, and methods to synthesize evidence synthesis.

Conclusions

For peer review only - http://bmjopen.bmj.com/site/about/guidelines.xhtml

For clinicians providing educational interventions to individuals with ACS and T2DM, the results from this review provide a contemporaneous perspective on current evidence on the effectiveness of health education (its content, and delivery methods) for this high risk patient group. The current evidence compiled by this umbrella review supports current international clinical guidelines, that theoretically-based education interventions lasting 6 months, delivered in multiple modes (face-to-face, phone contact, online or web-based, or video), and with individualized education delivered weekly, are more likely to generate positive outcomes. This review also supports health education- related interventions provided by health professionals, including nurses and multidisciplinary teams, delivering content including specific clinical factors for ACS and T2DM (BP, glycemic level, and medication), modifiable risk factors (unhealthy diet, inactivity, and smoking), and other psychological factors (anxiety and depression). These health education interventions could be delivered post-discharge, such as rehabilitation centers, primary care centers, and the community and should be at least 6 months in duration. The effectiveness of these programs was based on HbA1C levels, knowledge, psychosocial outcomes, readmission rates, and smoking status rather than clear evidence of reduced mortality, MI, or short and long-term complications. In addition, psychoeducational interventions were more effective for patients with ACS, and health education that was culturally appropriate or taught self-management was more effective for patients with T2DM. We also found that longer durations and high-intensity health education provided in an individualized format were more helpful for patients with ACS or T2DM.

The fact that none of the included reviews included patients with both ACS and T2DM indicates a clear need for further rigorous experimental studies with patients with both diseases. Future research that includes these aspects of education are likely determine the effectiveness of

For peer review only - http://bmjopen.bmj.com/site/about/guidelines.xhtml

educational interventions focusing on cardiovascular and DM risk factors and complications within patients with ACS and T2DM.

Acknowledgements: I would like to thank the authors of the original articles who provided additional unpublished data.

Author contributions:

Study conception and design: XL-L, MJ, KW, C-JW, YS. Data collection: XL-L, YS. Data analysis: XL-L, YS, MJ, KW, C-JW. Manuscript drafts: XL-L, MJ, C-JW, KW, YS.

Conflicts of interest: None declared.

Founding statement: This research received no specific grant from any funding agency in the public, commercial or not-for-profit sectors. The lead author is a recipient of an Australian Catholic University Faculty of Health Sciences Tongji University Cotutelle Ph.D. Scholarship.

Data sharing statement: No additional data are available.

For peer review only

References

[1]. Scarborough P, Wickramasinghe K, Bhatnagar P, et al. Trends in coronary heart disease, 1961-2011. *British Heart Foundation*, London (2011).

[2]. Finegold JA, Asaria P, Francis DP. Mortality from ischaemic heart disease by country, region, and age: statistics from World Health Organisation and United Nations. *Int J Cardiol* 2013;168(2):934-45.

[3]. Amsterdam EA, Wenger NK, Brindis RG, et al. 2014 AHA/ACC guideline for the management of patients with non–ST-elevation acute coronary syndromes: a report of the American College of Cardiology/American Heart Association Task Force on Practice Guidelines. *J Am Coll Cardiol* 2014; 64(24):e139-e228.

[4]. Kahn M, Wheatcroft S. Acute coronary syndromes in diabetes. In: Fisher M (ed.). Heart Disease and Diabetes. Oxford University Press, Oxford, 2008.

[5]. Hasin T, Hochadel M, Gitt AK, et al. Comparison of treatment and outcome of acute coronary syndrome in patients with versus patients without diabetes mellitus. *Am J Cardiol* 2009;103 (6):772-78.

[6]. Franklin K, Goldberg RJ, Spencer F, et al. Implications of diabetes in patients with acute coronary syndromes: the Global Registry of Acute Coronary Events. *Arch Intern Med* 2004;164(13):1457-63.

[7]. Ting HH, Chen AY, Roe MT, et al. Delay From Symptom Onset to Hospital Presentation for Patients With Non–ST-Segment Elevation Myocardial Infarction. *Arch Intern Med* 2010;170(20):1834-41.

[8]. Saely CH, Aczel S, Koch L, et al. Diabetes as a coronary artery disease risk equivalent: before a change of paradigm? *Eur J Cardiovasc Prev Rehabil* 2010; 17(1):94-99.

[9]. Donahoe SM, Stewart GC, McCabe CH, et al. Diabetes and mortality following acute coronary syndromes. *JAMA* 2007; 298(7):765-75.

[10]. Lakerveld J, Bot SD, Chinapaw MJ, et al. Motivational interviewing and problem solving treatment to reduce type 2 diabetes and cardiovascular disease risk in real life: a randomized controlled trial. *Int J Behav Nutr Phys Act* 2013; 10(1):1.

[11]. Glanz K, Rimer BK, Viswanath K. Health behavior and health education: theory, research, and practice: John Wiley & Sons 2008.

[12]. Powers MA, Bardsley J, Cypress M, et al. Diabetes self-management education and support in type 2 diabetes: a joint position statement of the American Diabetes Association, the American Association of Diabetes Educators, and the Academy of Nutrition and Dietetics. *Diabetes Educ* 2017; 43(1):40-53.

[13]. Haas L, Maryniuk M, Beck J, et al. National standards for diabetes self-management

For peer review only - http://bmjopen.bmj.com/site/about/guidelines.xhtml

education and support. *Diabetes Educ* 2012; 38(5):619-29.

[14]. Fisher EB, Boothroyd RI, Coufal MM, et al. Peer support for self-management of diabetes improved outcomes in international settings. *Health Aff* 2012;31(1):130-39.

[15]. Tang PC, Overhage JM, Chan AS, et al. Online disease management of diabetes: engaging and motivating patients online with enhanced resources-diabetes (EMPOWER-D), a randomized controlled trial. *J Am Med Inform Assoc* 2013;20(3):526-34.

[16]. Association AD. Standards of medical care in diabetes—2014. *Diabetes Care* 2014;37 (Supplement 1):S14-S80.

[17]. Chang AM, McDowell J. Perspectives of patients with type 2 diabetes following a critical cardiac event–an interpretative approach. *J Clin Nurs* 2008;17(5a):16-24.

[18]. Becker LA OA. Chapter 22: overviews of reviews.In: Higgins JPT, Green S (editors). Cochrane Handbook for Systematic Reviews of Interventions Version 5.1.0 [updated March 2011]. *The Cochrane Collaboration* 2011

[19]. Chew DP, Scott IA, Cullen L, et al. National Heart Foundation of Australia and Cardiac Society of Australia and New Zealand: Australian clinical guidelines for the management of acute coronary syndromes 2016. *Heart Lung Circ* 2016;25(9):895-951.

[20]. Alberti KGMM, Zimmet Pf. Definition, diagnosis and classification of diabetes mellitus and its complications. Part 1: diagnosis and classification of diabetes mellitus. Provisional report of a WHO consultation. *Diabet Med* 1998;15(7):539-53.

[21]. Thomson Reuters. EndNote version X7 2016 [Available from: http://endnote.com/product-details/x7.]

[22]. Shea BJ, Grimshaw JM, Wells GA, et al. Development of AMSTAR: a measurement tool to assess the methodological quality of systematic reviews. *BMC Med Res Methodol* 2007;7(1):1.

[23]. Sharif MO, Janjua-Sharif F, Ali H, et al. Systematic reviews explained: AMSTAR-how to tell the good from the bad and the ugly. *Oral Health Dent Manag* 2013;12(1):9-16.

[24]. Aromataris E, Fernandez R, Godfrey CM, et al. Summarizing systematic reviews: methodological development, conduct and reporting of an umbrella review approach. *Int J Evid Based Healthc* 2015; 13(3):132-40.

[25]. Pieper D, Antoine S-L, Mathes T, et al. Systematic review finds overlapping reviews were not mentioned in every other overview. *J Clin Epidemiol* 2014; 67(4):368-75.

[26]. Ryan RE, Weir M, Leslie B, et al. Evidence on Optimal Prescribing and Medicines Use for Decision Makers: Scope and Application of the Rx for Change Database. *J Pharm Pract* 2011; 41(4):295-99.

For peer review only

[27]. Sapkota S, Brien JAE, Greenfield JR, et al. A systematic review of interventions addressing adherence to anti-diabetic medications in patients with type 2 diabetes. *PLoS ONE* 2015; 10 (6): e0128581.

[28]. Walker RJ, Smalls BL, Bonilha HS, et al. Behavioral interventions to improve glycemic control in African Americans with type 2 diabetes: a systematic review. *Ethn Dis* 2013; 23(4):401-08.

[29]. Heinrich E, Schaper NC, de Vries NK. Self-management interventions for type 2 diabetes: a systematic review. *Eur Diabetes Nursing* 2010; 7(2):71-76.

[30]. Alam R, Sturt J, Lall R, et al. An updated meta-analysis to assess the effectiveness of psychological interventions delivered by psychological specialists and generalist clinicians on glycaemic control and on psychological status. *Patient Educ Couns* 2009;75(1):25-36.

[31]. Aldcroft SA, Taylor NF, Blackstock FC, et al. Psychoeducational Rehabilitation for Health Behavior Change in Coronary Artery Disease: A systematic review of controlled trials. *J Cardiopulm Rehabil Prev* 2011; 31(5):273-81.

[32]. Amaeshi IJ. Exploring the impact of structured foot health education on the rate of lower extremity amputation in adults with type 2 diabetes. A systematic review. *Podiatry Now* 2012;15(6):20-27.

[33]. Attridge M, Creamer J, Ramsden M, et al. Culturally appropriate health education for people in ethnic minority groups with type 2 diabetes mellitus. *Cochrane Database Syst Rev* 2014; 9:CD006424.

[34]. Auer R, Gaume J, Rodondi N, et al. Efficacy of in-hospital multidimensional interventions of secondary prevention after acute coronary syndrome a systematic review and meta-analysis. *Circulation* 2008;117(24):3109-17.

[35]. Barth J, Critchley J, Bengel J. Efficacy of psychosocial interventions for smoking cessation in patients with coronary heart disease: A systematic review and meta-analysis. *Ann Behav Med* 2006;32(1):10-20.

[36]. Barth J, Critchley JA, Bengel J. Psychosocial interventions for smoking cessation in patients with coronary heart disease. *Cochrane Libr* 2008

[37]. Brown JP, Clark AM, Dalal H, et al. Effect of patient education in the management of coronary heart disease: A systematic review and meta-analysis of randomized controlled trials. *Eur J Prev Cardiol* 2013;20(4):701-14.

[38]. Burke E, Robert, Ferrara A, et al. The effectiveness of group medical visits on diabetes mellitus type 2 (dm2) specific outcomes in adults: a systematic review, *JBI Libr Syst Rev*. 2011;9(23):833-885.

[39]. Chen L, Pei JH, Kuang J, et al. Effect of lifestyle intervention in patients with type 2

For peer review only - http://bmjopen.bmj.com/site/about/guidelines.xhtml

diabetes: A meta-analysis. *Metab. Clin. Exp.* 2015;64(2):338-47.

[40]. Choi TST, Davidson ZE, Walker KZ, et al. Diabetes education for Chinese adults with type 2 diabetes: A systematic review and meta-analysis of the effect on glycemic control. *Diabetes Res Clin Pract* 2016;116:218-29.

[41]. Clark AM, Hartling L, Vandermeer B, et al. Meta-analysis: secondary prevention programs for patients with coronary artery disease. *Ann Intern Med* 2005;143(9):659-72.

[42]. Creamer J, Attridge M, Ramsden M, et al. Culturally appropriate health education for Type 2 diabetes in ethnic minority groups: An updated Cochrane Review of randomized controlled trials. *Diabet Med* 2016;33(2):169-83.

[43]. Deakin T, McShane CE, Cade JE, et al. Group based training for self-management strategies in people with type 2 diabetes mellitus. *Cochrane Database Syst Rev* 2005;(2):CD003417.

[44]. Devi R, Singh Sally J, Powell J, et al. Internet-based interventions for the secondary prevention of coronary heart disease. *Cochrane Database Syst Rev* 2015, 22;(12): CD009386.

[45]. Dickens C, Cherrington A, Adeyemi I, et al. Characteristics of psychological interventions that improve depression in people with coronary heart disease: A systematic review and meta-regression. *Psychosom Med* 2013;75(2):211-21.

[46]. Duke SA, Colagiuri S, Colagiuri R. Individual patient education for people with type 2 diabetes mellitus. *Cochrane Database Syst Rev,* 2009;(1):CD005268.

[47]. Fan L, Sidani S. Effectiveness of diabetes self-management education intervention elements: a meta-analysis. *Can J Diabetes* 2009;33(1):18-26.

[48]. Fernandez R, Griffiths R, Everett B, et al. Effectiveness of brief structured interventions on risk factor modification for patients with coronary heart disease: a systematic review, Int *J Evid Based Healthc* 2007;5(4):370-405.

[49]. Gary TL, Genkinger JM, Guallar E, et al. Meta-analysis of randomized educational and behavioral interventions in type 2 diabetes. *Diabetes Educ 2003;29(3):488-501.*

[50]. Ghisi GLD, Abdallah F, Grace SL, et al. A systematic review of patient education in cardiac patients: Do they increase knowledge and promote health behavior change? *Patient Educ Couns* 2014;95(2):160-74.

[51]. Goulding L, Furze G, Birks Y. Randomized controlled trials of interventions to change maladaptive illness beliefs in people with coronary heart disease: systematic review. *J Adv Nurs* 2010;66(5):946-61.

[52]. Gucciardi E, Chan VWS, Manuel L, et al. A systematic literature review of diabetes self-management education features to improve diabetes education in women of Black

For peer review only

African/Caribbean and Hispanic/Latin American ethnicity. *Patient Educ Couns* 2013; 92(2):235-45.

[53]. Hawthorne K, Robles Y, Cannings-John R, et al. Culturally appropriate health education for type 2 diabetes mellitus in ethnic minority groups. *Cochrane Database Syst Rev* 2008;(3):CD006424.

[54]. Hawthorne K, Robles Y, Cannings-John R, et al. Culturally appropriate health education for type 2 diabetes in ethnic minority groups: a systematic and narrative review of randomized controlled trials. *Diabet Med* 2010;27(6):613-23

[55]. Huang XL, Pan JH, Chen D, et al. Efficacy of lifestyle interventions in patients with type 2 diabetes: A systematic review and meta-analysis. *Eur J Intern Med* 2016;27:37-47.

[56]. Huttunen-Lenz M, Song F, Poland F. Are psychoeducational smoking cessation interventions for coronary heart disease patients effective? Meta-analysis of interventions. *Br J Health Psychol* 2010;15(Pt 4):749-77.

[57]. Kai Lun Gan J, Brammer JD, Creedy DK. Effectiveness of educational interventions to promote oral hypoglycaemic adherence in adults with Type 2 diabetes: a systematic review. *JBI Libr Syst Rev* 2011;9(9):269-312.

[58]. Khunti K, Camosso-Stefinovic J, Carey M, et al. Educational interventions for migrant South Asians with type 2 diabetes: a systematic review. *Diabet Med* 2008;25(8):985-92.

[59]. Kotb A, Hsieh S, Wells GA. The effect of telephone support interventions on coronary artery disease (CAD) patient outcomes during cardiac rehabilitation: a systematic review and meta-analysis. *PLoS One* 2014;9(5):e96581.

[60]. Loveman E, Frampton GK, Clegg AJ. The clinical effectiveness of diabetes education models for type 2 diabetes: a systematic review. *Health Technol Assess* 2008;12(9):1-116,

[61]. Minet L, Moller S, Vach W, et al. Mediating the effect of self-care management intervention in type 2 diabetes: a meta-analysis of 47 randomised controlled trials. *Patient Educ Couns* 2010; 80(1):29-41.

[62]. Nam S, Janson SL, Stotts NA, et al. Effect of culturally tailored diabetes education in ethnic minorities with type 2 diabetes: a meta-analysis. *J Cardiovasc Nurs* 2012;27(6): 505-18.

[63]. Nield L, Moore H, Hooper L, et al. Dietary advice for treatment of type 2 diabetes mellitus in adults. *Cochrane Database Syst Rev 2007;(3):CD004097.*

[64]. Norris SL, Engelgau MM, Narayan KM. Effectiveness of self management training in type 2 diabetes: a systematic review of randomized controlled trials. *Diabetes Care* 24(3):561-87.

[65]. Norris SL, Lau J, Smith SJ, et al. Self-management education for adults with type 2

For peer review only

For peer review only - http://bmjopen.bmj.com/site/about/guidelines.xhtml

diabetes: a meta-analysis of the effect on glycemic control. *Diabetes Care 2002;25(7): 1159-71.*

[66]. Odnoletkova I, Goderis G, Pil L, et al. Cost-effectiveness of therapeutic education to prevent the development and progression of type 2 diabetes: Systematic review. *J Diabetes Metab 2014;5: 438*

[67]. Pal K, Eastwood SV, Michie S, et al. Computer-based interventions to improve self-management in adults with type 2 diabetes: a systematic review and meta-analysis. *Diabetes Care 2014;37(6):1759-66*

[68]. Pal K, Eastwood SV, Michie S, et al. Computer-based diabetes self-management interventions for adults with type 2 diabetes mellitus. *Cochrane Database Syst Rev* 2013;(3):CD008776

[69]. Barth J, Jacob T, Daha I, et al. Psychosocial interventions for smoking cessation in patients with coronary heart disease. *Cochrane Database Syst Rev* 2015;(7):CD006886.

[70]. Brown JP, Clark AM, Dalal H, et al. Patient education in the management of coronary heart disease. *Cochrane Database Syst Rev* 2011;(12):CD008895

[71]. Pillay J, Armstrong MJ, Butalia S, et al. Behavioral Programs for Type 2 Diabetes Mellitus: A Systematic Review and Network Meta-analysis. *Ann Intern Med* 2015; 163(11):848-60.

[72]. Terranova CO, Brakenridge CL, Lawler SP, et al. Effectiveness of lifestyle-based weight loss interventions for adults with type 2 diabetes: a systematic review and meta-analysis. *Diabetes Obes Metab*2015;17(4):371-8.

[73]. Ricci-Cabello I, Ruiz-Pérez I, Rojas-García A, et al. Characteristics and effectiveness of diabetes self-management educational programs targeted to racial/ethnic minority groups: a systematic review, meta-analysis and meta-regression. *BMC Endocr Disord* 2014;14(1): 60.

[74]. Saffari M, Ghanizadeh G, Koenig HG. Health education via mobile text messaging for glycemic control in adults with type 2 diabetes: a systematic review and meta-analysis. *Prim Care Diabetes 2014;8(4):275-85.*

[75]. Vugt M, Wit M, Cleijne WH, et al. Use of behavioral change techniques in web-based self-management programs for type 2 diabetes patients: systematic review. *J Med Internet Res* 2013; 15(12):e279.

[76]. Steinsbekk A, Rygg LO, Lisulo M, et al. Group based diabetes self-management education compared to routine treatment for people with type 2 diabetes mellitus. A systematic review with meta-analysis. *BMC Health Serv Res 2012;12:213.*

[77]. Ramadas A, Quek KF, Chan CK, et al. Web-based interventions for the management of type 2 diabetes mellitus: a systematic review of recent evidence. *Int J Med Inform 2011*;

For peer review only - http://bmjopen.bmj.com/site/about/guidelines.xhtml

80(6):389-405.

[78]. Wens J, Vermeire E, Hearnshaw H, et al. Educational interventions aiming at improving adherence to treatment recommendations in type 2 diabetes: A sub-analysis of a systematic review of randomised controlled trials. *Diabetes Res Clin Pract* 2008;79 (3):377-88.

[79]. Zabaleta AM, Forbes A. Structured group-based education for type 2 diabetes in primary care. *Br J Community Nurs 2007;12(4):158-62.*

[80]. Vermeire E, Wens J, Van Royen P, et al. Interventions for improving adherence to treatment recommendations in people with type 2 diabetes mellitus. *Cochrane Database Syst Rev 2005;(2):CD003638.*

[81]. Albrecht L, Archibald M, Arseneau D, et al. Development of a checklist to assess the quality of reporting of knowledge translation interventions using the Workgroup for Intervention Development and Evaluation Research (WIDER) recommendations. *Implement Sci 2013;8:52.*

[82]. Glazier RH, Bajcar J, Kennie NR, et al. A systematic review of interventions to improve diabetes care in socially disadvantaged populations. *Diabetes Care* 2006; 29(7):1675-88.

[83]. Powers MA, Bardsley J, Cypress M, et al. Diabetes Self-management Education and Support in Type 2 Diabetes: A Joint Position Statement of the American Diabetes Association, the American Association of Diabetes Educators, and the Academy of Nutrition and Dietetics. *Diabetes Care* 2015;38(7):1372-82

For peer review only

For peer review only - http://bmjopen.bmj.com/site/about/guidelines.xhtml

Table 1. Methodological quality assessment of included systematic reviews and meta-analyses

	Systematic Review/ Meta-analysis	Item 1	Item 2	Item 3	Item 4	Item 5	Item 6	Item 7	Item 8	Item 9	Item 10	Item 11	Total score
**Systematic reviews and meta-analysis involved ACS patients**													
1	Barth, Jacob, Daha, & Critchley, 2015 [69]	Yes	Yes	Yes	Yes	Yes	Yes	Yes	Yes	Yes	Yes	Yes	11
2	Devi et al., 2015 [44]	Yes	Yes	Yes	Yes	Yes	Yes	Yes	Yes	Yes	NA	Yes	10
3	Ghisi, Abdallah, Grace, Thomas, & Oh, 2014 [50]	CA	Yes	Yes	Yes	No	Yes	Yes	Yes	Yes	No	No	7
4	Kotb, Hsieh, & Wells, 2014 [59]	CA	Yes	Yes	Yes	No	Yes	Yes	Yes	Yes	No	Yes	8
5	Brown, Clark, Dalal, Welch, & Taylor, 2013 [37]	Yes	No	Yes	CA	No	Yes	Yes	Yes	Yes	NA	Yes	7
6	Dickens et al., 2013 [45]	CA	Yes	Yes	CA	No	Yes	Yes	Yes	Yes	Yes	Yes	8
7	Aldcroft, Taylor, Blackstock, & O'Halloran P, 2011 [31]	CA	No	Yes	CA	NO	Yes	Yes	Yes	Yes	No	Yes	6
8	Brown, Clark, Dalal, Welch, & Taylor, 2011 [70]	Yes	Yes	Yes	Yes	Yes	Yes	Yes	Yes	Yes	NA	Yes	10
9	Huttunen-Lenz, Song, & Poland, 2010 [56]	CA	No	Yes	CA	No	Yes	Yes	Yes	Yes	No	No	5
10	Goulding, Furze, & Birks, 2010 [51]	Yes	Yes	Yes	CA	No	Yes	Yes	Yes	Yes	No	Yes	8
11	Auer, Gaume, Rodondi, Cornuz, & Ghali, 2008 [34]	CA	Yes	Yes	CA	No	No	Yes	No	Yes	Yes	No	5
12	J. Barth, Critchley, & Bengel, 2008 [36]	Yes	Yes	Yes	No	Yes	Yes	Yes	Yes	Yes	Yes	Yes	10
13	Fernandez et al., 2007 [48]	Yes	Yes	Yes	Yes	Yes	Yes	Yes	Yes	Yes	No	No	8
14	J. Barth, Critchley, & Bengel, 2006 [35]	CA	Yes	Yes	CA	No	Yes	CA	Yes	Yes	Yes	Yes	7

For peer review only

For peer review only - http://bmjopen.bmj.com/site/about/guidelines.xhtml

15	Clark, Hartling, Vandermeer, & McAlister, 2005 [41]	CA	Yes	Yes	CA	No	Yes	Yes	Yes	Yes	Yes	Yes	8
**Systematic reviews and meta-analysis involved T2DM patients**													
16	Choi, Davidson, Walker, Lee, & Palermo, 2016 [40]	CA	Yes	Yes	No	No	Yes	Yes	Yes	Yes	Yes	Yes	8
17	Creamer, Attridge, Ramsden, Cannings-John, & Hawthorne, 2016 [42]	Yes	Yes	Yes	CA	No	Yes	Yes	Yes	Yes	No	Yes	8
18	Huang et al., 2016 [55]	CA	CA	Yes	CA	No	Yes	Yes	Yes	Yes	Yes	Yes	7
19	Chen et al., 2015 [39]	CA	CA	Yes	CA	No	Yes	Yes	Yes	Yes	Yes	Yes	7
20	Pillay et al., 2015 [71]	Yes	No	Yes	Yes	No	Yes	Yes	Yes	Yes	Yes	Yes	9
21	Terranova, Brakenridge, Lawler, Eakin, & Reeves, 2015 [72]	CA	CA	Yes	No	Yes	Yes	Yes	Yes	Yes	Yes	Yes	8
22	Attridge, Creamer, Ramsden, Cannings-John, & Hawthorne, 2014 [33]	Yes	Yes	Yes	Yes	Yes	Yes	Yes	Yes	Yes	No	Yes	10
23	Odnoletkova et al., 2014 [66]	Yes	CA	Yes	CA	No	No	Yes	Yes	Yes	Yes	No	6
24	Pal et al., 2014 [67]	CA	Yes	Yes	Yes	No	Yes	Yes	Yes	Yes	No	Yes	8
25	Ricci-Cabello et al., 2014 [73]	Yes	CA	Yes	Yes	No	Yes	Yes	Yes	Yes	Yes	Yes	9
26	Saffari, Ghanizadeh, & Koenig, 2014 [74]	CA	Yes	Yes	CA	No	Yes	Yes	Yes	Yes	Yes	Yes	8
27	Gucciardi, Chan, Manuel, & Sidani, 2013 [52]	CA	Yes	Yes	No	No	Yes	Yes	Yes	Yes	No	Yes	7
28	Pal et al., 2013 [68]	Yes	Yes	Yes	Yes	Yes	Yes	Yes	Yes	Yes	No	Yes	10
29	Vugt, Wit, Cleijne, & Snoek, 2013 [75]	CA	Yes	Yes	CA	No	Yes	Yes	Yes	NA	No	Yes	6
30	Amaeshi, 2012 [32]	CA	CA	Yes	No	No	Yes	Yes	Yes	NA	No	No	4
31	Nam, Janson, Stotts, Chesla, &	CA	CA	Yes	Yes	No	Yes	Yes	Yes	Yes	Yes	Yes	8

For peer review only - http://bmjopen.bmj.com/site/about/guidelines.xhtml

	Kroon, 2012 [62]												
**32**	Steinsbekk, Rygg, Lisulo, Rise, & Fretheim, 2012 [76]	CA	Yes	Yes	CA	No	Yes	Yes	Yes	Yes	No	Yes	7
**33**	Burke et al., 2011 [38]	Yes	Yes	Yes	Yes	Yes	Yes	Yes	Yes	Yes	NA	Yes	10
**34**	Kai Lun Gan, Brammer, & Creedy, 2011 [57]	Yes	Yes	Yes	CA	No	Yes	Yes	Yes	Yes	No	Yes	8
**35**	Ramadas, Quek, Chan, & Oldenburg, 2011 [77]	CA	CA	Yes	No	No	Yes	Yes	Yes	NA	No	Yes	5
**36**	Hawthorne, Robles, Cannings-John, & Edwards, 2010 [54]	Yes	Yes	Yes	CA	No	Yes	Yes	Yes	Yes	CA	Yes	8
**37**	Minet, Moller, Vach, Wagner, & Henriksen, 2010 [61]	Yes	Yes	Yes	No	No	Yes	Yes	Yes	Yes	Yes	Yes	9
**38**	Alam, Sturt, Lall, & Winkley, 2009 [30]	Yes	Yes	No	CA	No	Yes	Yes	Yes	Yes	Yes	Yes	8
**39**	Duke, Colagiuri, & Colagiuri, 2009 [46]	Yes	CA	Yes	No	Yes	Yes	Yes	Yes	Yes	No	Yes	8
**40**	Fan & Sidani, 2009 [47]	Yes	No	Yes	CA	No	Yes	No	No	Yes	No	Yes	5
**41**	Hawthorne, Robles, Cannings-John, & Edwards, 2008 [53]	Yes	Yes	Yes	Yes	Yes	Yes	Yes	Yes	Yes	Yes	Yes	11
**42**	Khunti, Camosso-Stefinovic, Carey, Davies, & Stone, 2008 [58]	CA	Yes	Yes	Yes	No	Yes	No	No	No	No	Yes	5
**43**	Loveman, Frampton, & Clegg, 2008 [60]	Yes	CA	Yes	Yes	No	Yes	Yes	Yes	Yes	No	Yes	8
**44**	Wens et al., 2008 [78]	CA	Yes	Yes	CA	No	Yes	Yes	Yes	Yes	NA	Yes	7
**45**	Nield et al., 2007 [63]	Yes	Yes	Yes	CA	Yes	Yes	Yes	Yes	Yes	No	Yes	9
**46**	Zabaleta & Forbes, 2007 [79]	CA	CA	Yes	CA	Yes	Yes	Yes	Yes	NA	No	No	5
**47**	Deakin, McShane, Cade, &	Yes	Yes	Yes	Yes	Yes	Yes	Yes	Yes	Yes	Yes	Yes	11

For peer review only

For peer review only - http://bmjopen.bmj.com/site/about/guidelines.xhtml

	Williams, 2005 [43]												
48	Vermeire et al., 2005 [80]	Yes	Yes	Yes	CA	Yes	Yes	Yes	Yes	Yes	No	Yes	9
49	Gary, Genkinger, Guallar, Peyrot, & Brancati, 2003 [49]	CA	Yes	No	Yes	No	Yes	Yes	No	Yes	No	Yes	6
50	Norris, Lau, Smith, Schmid, & Engelgau, 2002 [65]	CA	No	Yes	No	No	Yes	Yes	Yes	CA	No	No	4
51	Norris, Engelgau, & Narayan, 2001 [64]	CA	Yes	Yes	CA	No	Yes	Yes	Yes	NA	No	No	5

Item 1:"Was an 'a priori' design provided?"; **Item 2**: "Was there duplicate study selection and data extraction?"; **Item 3**: "Was a comprehensive literature search performed?"; **Item 4**: "Was the status of publication (ie. grey literature) used as an inclusion criterion?"; **Item 5**: "Was a list of studies (included and excluded) provided?"; **Item 6**: "Were the characteristics of the included studies provided?"; **Item 7**: "Was the scientific quality of the included studies assessed and documented?"; **Item 8**: "Was the scientific quality of the included studies used appropriately in formulating conclusions?"; **Item 9**: "Were the methods used to combine the findings of studies appropriate?"; **Item 10**: "Was the likelihood of publication bias assessed?"; **Item 11**: "Was the conflict of interest stated?"

CA= Cannot Answer; NA= Not Application;

Source: *Shea, B. J., Grimshaw, J. M., Wells, G. A., Boers, M., Andersson, N., Hamel, C., ... & Bouter, L. M. (2007). Development of AMSTAR: a measurement tool to assess the methodological quality of systematic reviews. BMC Medical Research Methodology, 7(1), 1.*

For peer review only - http://bmjopen.bmj.com/site/about/guidelines.xhtml

Table 2. Characteristics and interventions of included systematic reviews and meta-analysis involved ACS patients

(First author, Year); Journal	Primary objectives (To assess effect of intervention s on….)	Studies details	Intervention						Outcomes (Primary outcomes were bold.) "–" : No change "↑" : Increase "↓" : decrease	Synthesis methods
			Educational Content		Provider	Number of session(s), delivery mode, time, setting				
(Devi et al., 2015) [44]; The Cochrane Library	Lifestyle changes and medicines manageme nt	**Number of studies**:11 completed trials (12 publications); **Types of studies:** RCTs; **Total sample:** 1,392 participants.	All Internet based interventions.	√ BEHA (-)<br>√ CVR (-)<br>√ DIET (-)<br>√ EXERCISE (-)<br>☐ MED<br>√ PSY(-)<br>√ SMOKING (-)<br>☐ SELF	Dietitians; exercise specialists; nurse practitioners; physiotherapist rehabilitation specialists, or did not describe.	**Number of session:** Weekly or monthly or unclear; **Total contact hours:** Unclear. **Duration:** From six weeks to one year.	**Strategies:** Internet- and mobile phone-based intervention, such as email access, private-messaging function on the website, one-to-one chat facility, a synchronised group chat, an online discussion forum, or telephone consultations; or video files; **Format:** one-on-one chat sessions; "ask an expert" group chat sessions; **Theoretical approach:** Unclear.	Inpatient settings, post discharge, other	**– Clinical outcomes;**<br>**– Cardiovascular risk factors;**<br>– Lifestyle changes;<br>– Compliance with medication;<br>– Healthcare utilization and costs;<br>↓ Adverse intervention effects.	Meta-analysis used Review Manager software.
(Barth, Jacob, Daha, & Critchley, 2015) [69]; The Cochrane Library	Smoking cessation	**Number of studies**:40 RCTs; **Types of studies:** RCTs; **Total sample:** 7,928 participants.	Psychosocial smoking cessation interventions	☐ BEHA<br>☐ CVR<br>☐ DIET<br>☐ EXERCISE<br>☐ MED<br>☐ PSY<br>√ SMOKING<br>☐ SELF	Cardiologist; general practitioner physician or study nurse.	**Number of session**: Weekly or 2-3 times per week; **Total contact hours:** Unclear. **Duration:** From 8 weeks to one year.	**Strategies:** Face to face, telephone contact, written educational materials, videotape, booklet or unclear; **Format:** One by one counseling; telephone call; group meetings or unclear; **Theoretical approach:** TTM, SCT.	Inpatient settings, post discharge, other	**↑ Abstinence by self-report or validated.**	Meta-analysis used Review Manager software.
(Kotb, Hsieh, & Wells, 2014) [59]; PloS one	Patients' outcomes.	**Number of studies**: 26 studies; **Types of studies:** RCTs; **Total sample:** 4,081	Telephone-delivered post-discharge interventions.	☐ BEHA<br>√ CVR<br>☐ DIET<br>☐ EXERCISE<br>☐ MED	Dietitians; exercise specialist; health educators; nurses and pharmacists.	**Number of session:** 3-6 sessions/telephone calls and was greater than 6 calls in five	**Strategies:** Telephone calls; **Format:** Unclear, did not describe the format; **Theoretical approach:** Unclear.	Unclear, did not describe the setting.	**↓ All-cause hospitalization;**<br>**– All-cause mortality;**<br>↓ Depression;	Meta-analysis used Review Manager software.

For peer review only

		participants.		☐ PSY<br>☐ SMOKING<br>☐ SELF		studies; or unclear;<br>**Total contact hours:** 40 mins to 180 mins or unclear;<br>**Duration:** 1.5 - 6 months or unclear.			− Anxiety;<br>↑ Smoking cessation,<br>↓ Systolic blood pressure;<br>− LDL-c.	
(Ghisi, Abdallah, Grace, Thomas, & Oh, 2014) [50]; Patient Education and Counseling	knowledge, health behavior change, medication adherence, psychosocial well-being.	**Number of studies**: 42 articles;<br>**Types of studies:** Thirty were experimental: 23 RCTs and 7 quasi-experimental; and 11 observational and 1 used a mixed-methods design.<br>**Total sample:** 16,079 participants.	Any educational interventions.	√ BEHA (+)<br>√ CVR (++)<br>√ DIET (+++)<br>√ EXERCISE (++)<br>√ MED (++)<br>√ PSY(++)<br>√ SMOKING (+)<br>☐ SELF	Nurses (35.7%), a multidisciplinary team (31%), dietitians (14.3%), and a cardiologist (2.4%).	**Number of session:** 1-24 or unclear.<br>**Total contact hours:** 5–10 min to 3 h as well as a full day of education.<br>**Duration:** 1-24 month; from daily education to every 6 months.	**Strategies:** Did not describe the strategies;<br>**Format:** Group (88.1%) education was delivered by lectures (40.5%), group discussions (40.5%), and question and answer periods (7.1%). Individual education (88.1%), including individual counseling (50%), follow-up telephone contacts (31%) and home visits (7.1%);<br>**Theoretical approach:** Unclear.	Inpatient settings	**− Knowledge,**<br>**− Behavior;**<br>**− Psychosocial indicators.**	Narrative synthesis
(Brown, Clark, Dalal, Welch, & Taylor, 2013) [37]; European Journal of Preventive Cardiology	Mortality, morbidity, HRQoL and healthcare costs.	**Number of studies:** Twenty-four papers reporting on 13 RCTs;<br>**Types of studies:** RCTs;<br>**Total sample:** 68,556 participants.	Patient education.	☐ BEHA<br>√ CVR<br>☐ DIET<br>☐ EXERCISE<br>☐ MED<br>☐ PSY<br>☐ SMOKING<br>☐ SELF	Nurses or other healthcare professionals.	**Number of session and Duration:** From a total of 2 visits to a 4-week residential stay reinforced with 11 months of nurse led follow-up.<br>**Total contact hours:** Unclear.	**Strategies:** Face-to-face education sessions, telephone contact and interactive use of the Internet;<br>**Format:** Group-based sessions, individualized education and four utilized a mixture of both sessions;<br>**Theoretical approach:** Unclear.	Inpatient settings, other	**− Mortality,**<br>**− Non-fatal MI,**<br>**− Revascularizations,**<br>**− Hospitalizations,**<br>**− HRQoL,**<br>− Withdrawals/drop outs;<br>− Healthcare utilization and costs.	Meta-analysis used Review Manager software.
(Dickens et al., 2013) [45]; Psychosomatic Medicine	Depression and depressive symptoms.	**Number of studies:** Sixty-two independent studies.<br>**Types of studies:** RCTs;<br>**Total sample:** 17,397.	Psychological interventions.	√ BEHA (-)<br>☐ CVR<br>☐ DIET<br>☐ EXERCISE<br>☐ MED<br>√ PSY (-)<br>☐ SMOKING	A single health professional or by a unidisciplinary team	**Number of session:** 14.4 (range, 1-156);<br>**Total contact hours:** Varying from 10 to 240 minutes.<br>**Duration:** Unclear.	**Strategies:** Face-to-face sessions, telephone contact or unclear;<br>**Format:** Group or unclear;<br>**Theoretical approach:** Unclear.	Unclear, did not describe.	**↓ Depression;**<br>− Adverse cardiac outcomes;<br>− Ongoing cardiac symptoms.	Univariate analyses using Comprehensive Meta-Analysis, multivariate

For peer review only

				√ SELF (-)						meta-regression using SPSS 15.0
(Aldcroft, Taylor, Blackstock, & O'Halloran P, 2011) [20]; Journal of Cardiopulmonary Rehabilitation & Prevention	Health behavior change.	**Number of studies:** 7 trials. **Types of studies:** Six randomized controlled trials and a quasi-experimental trial. **Total sample:** 536 participants.	All psychoeducational or behavioral intervention.	☐ BEHA<br>√ CVR (-)<br>☐ DIET<br>☐ EXERCISE<br>☐ MED<br>√ PSY (-)<br>☐ SMOKING<br>☐ SELF	Appropriately trained health care workers.	**Number of session:** Unclear; **Total contact hours:** Unclear; **Duration:** 2-12 months.	**Strategies:** Did not describe the strategies; **Format:** Group setting, combination of group and one-on-one education and one-on-one format only; **Theoretical approach:** TTM, interactionist role theory, Bandura's self-efficacy theory, Gordon's relapse prevention model and a cognitive behavioral approach.	Unclear, did not describe.	↓ **Smoking rates; medication use;**<br>− **Supplemental oxygen use;**<br>↑ **Physical activity;**<br>↑ Nutritional habits.	Meta-analysis and narrative presentation
(Brown, Clark, Dalal, Welch, & Taylor, 2011) [70]; The Cochrane Library	Mortality, morbidity, HRQofL and healthcare costs.	**Number of studies:** 24 papers reporting on 13 studies. **Types of studies:** RCTs; **Total sample:** 68,556 participants.	Patient education.	√ BEHA (-)<br>√ CVR (-)<br>☐ DIET<br>√ EXERCISE (-)<br>√ MED<br>☐ PSY<br>☐ SMOKING<br>☐ SELF	Nurse or did not describe.	**Number of session and Duration:** two 2 visits to 4 week residential 11 months of nurse led folluw up **Total contact hours:** Unclear.	**Strategies:** face-to-face sessions, telephone contact and interactive use of the Internet; **Format:** Four studies involved group sessions, five involved individualized education and three utilized both session types, with one study comparing the two approaches; **Theoretical approach:** did not describe.	Post discharge, other	− **Total mortality;**<br>− **Cardiovascular**<br>− **mortality;**<br>− **Non-cardiovascular mortality;**<br>− **Total cardiovascular (CV) events;**<br>− **Fatal and/or non-fatal MI;**<br>− Other fatal and/or non-fatal CV events	Meta-analysis used Review Manager software.
(Goulding, Furze, & Birks, 2010) [51]; Journal of Advanced Nursing	Change maladaptive illness.	**Number of studies:** **13 studies;** **Types of studies:** RCTs; **Total sample:** Unclear.	Interventions to change maladaptive illness beliefs	√ BEHA (-)<br>☐ CVR<br>☐ DIET<br>☐ EXERCISE<br>☐ MED<br>√ PSY (-)<br>☐ SMOKING	Cardiologist, nurse, psychologist or did not describe.	**Number of session:** Unclear; **Total contact hours:** Unclear; **Duration:** 4 days to two weeks or unclear.	**Strategies:** Face-to-face sessions, telephone contact and written self-administered; **Format:** Unclear; **Theoretical approach:** Common Sense Model, Leventhal's framework.	Inpatient settings, post discharge, other	− **Beliefs (or other illness cognition);**<br>− QoL;<br>− Behaviour;<br>− Anxiety or depression;<br>− Psychological	A descriptive data synthesis.

For peer review only

				☐ SELF					wellbeing;<br>− Modifiable risk factors; protective factors.	
(Huttunen-Lenz, Song, & Poland, 2010) [56]; British Journal of Health Psychology	Smoking cessation.	**Number of studies:** A total of 14 studies were included.<br>**Types of studies:** RCTs;<br>**Total sample:** 1,792 participants.	Psychoeducational cardiac rehabilitation intervention.	☐ BEHA<br>☐ CVR<br>☐ DIET<br>☐ EXERCISE<br>☐ MED<br>☐ PSY<br>√ SMOKING (-)<br>☐ SELF	Cardiologist, nurse psychologist, or did not describe.	**Number of session:** 4-20 or unclear.<br>**Total contact hours:** 10-720 mins or unclear.<br>**Duration:** 4-29 weeks or unclear.	**Strategies:** Face-to-face counselling, self-help materials; home visit, booklet, video and telephone contact.<br>**Format:** individual or unclear.<br>**Theoretical approach:** Social learning theory; ASE model; TTM; Behavioural multicomponent approach.	Inpatient settings, post discharge, other	↑ **Prevalent smoking cessation,**<br>↑ **Continuous smoking cessation,**<br>− Mortality.	Subgroup meta-analysis was used software.
(Auer, Gaume, Rodondi, Cornuz, & Ghali, 2008) [34]; Circulation	Multiple cardiovascular risk factors and all-cause mortality.	**Number of studies:** 27 articles reporting 26 studies.<br>**Types of studies:** 16 clinical controlled trials and 10 before-after studies.<br>**Total sample:** 2,467 patients in CCTs and 38, 581 patients in before-after studies.	In-Hospital Multidimensional Interventions of Secondary Prevention	☐ BEHA<br>☐ CVR<br>√ DIET (-)<br>√ EXERCISE (-)<br>√ MED<br>√ PSY (-)<br>√ SMOKING (-)<br>☐ SELF	Cardiac nurses; physician, or did not describe.	**Number of session:**1-5 or unclear;<br>**Total contact hours:** 30-240mins or unclear;<br>**Duration:** 4 weeks – 12 months.	**Strategies:** Written material; audiotapes; presentations; face-to-face;<br>**Format:** Group or unclear;<br>**Theoretical approach:** Unclear.	Inpatient settings.	↓ **All-cause mortality;**<br>↓ **Readmission rates;**<br>− **Reinfarction rates.**	Stata version 9.1.
(Barth, Critchley, & Bengel, 2008) [36] ; The Cochrane Library	Smoking cessation.	**Number of studies:** Forty trials;<br>**Types of studies:** RCTs;<br>**Total sample:** 7,682 patients.	Psychosocial intervention	√ BEHA (+++)<br>√ CVR (++)<br>☐ DIET<br>☐ EXERCISE<br>☐ MED<br>√ PSY (+)<br>√ SMOKING (+++)	Cardiologist, nurse, physician or study nurse.	**Number of session:** 1-5 or unclear;<br>**Total contact hours:** 15 mins - 9 hours.<br>**Duration:** within 4 weeks or did not report on the duration.	**Strategies:** Face to face; information booklets, audio- or videotapes.<br>**Format:** Group sessions or individual counselling;<br>**Theoretical approach:** TTM.	Inpatient settings	↑ **Abstinence by self-report or validated.**	Meta-analysis used Review Manager software.

				√ SELF(+++)						
(Fernandez et al., 2007) [48]; International Journal of Evidence-Based Healthcare	Risk factor modification.	**Number of studies:** Seventeen trials; **Types of studies:** randomised, quasi-RCTs and clustered trials; **Total sample:** 4,725 participants.	Brief structured intervention.	√ BEHA (-)<br>√ CVR (-)<br>☐ DIET<br>☐ EXERCISE<br>☐ MED<br>☐ PSY<br>☐ SMOKING<br>√ SELF (-)	Case manager; dieticians; health educator; nurses; psychologist; and research assistants.	**Number of session:** supportive counselling ranged from 1 to 7 calls for the duration of the study; **Total contact hours:** varied from 10 to 30 mins; **Duration:** Unclear.	**Strategies:** Written, visual, audio, telephone contact; **Format:** did not describe; **Theoretical approach:** theoretical behaviour change principles.	Unclear, did not describe.	↓ **Smoking;**<br>− **Cholesterol level;**<br>− **Physical activity;**<br>↑ **Dietary habits;**<br>↓ **Blood sugar levels;**<br>− **BP levels;**<br>↓ **BMI;**<br>− Incidence of admission.	Cochrane statistical package Review Manager.
(J. Barth, Critchley, & Bengel, 2006) [35]; Annals of Behavioral Medicine	Smoking cessation.	**Number of studies:** Nineteen trials; **Types of studies:** RCTs; **Total sample:** 2,548 patients.	Psychosocial interventions.	√ BEHA (+++)<br>√ CVR (++)<br>☐ DIET<br>☐ EXERCISE<br>☐ MED<br>☐ PSY<br>☐ SMOKING<br>√ SELF (+++)	Unclear, did not describe.	**Number of session:** Unclear; **Total contact hours:** Unclear; **Duration:** Unclear.	**Strategies:** Face to face, telephone contact or unclear; **Format:** Unclear; **Theoretical approach:** Unclear.	Unclear, did not describe.	↑ **Abstinence;**<br>↓ **Smoking status**.	Data analyses were carried out in Review Manager 4.2.
(Clark, Hartling, Vandermeer, & McAlister, 2005) [41]; Annals of Internal Medicine	Mortality, MI	**Number of studies:** 63 randomized trials; **Types of studies:** RCTs; **Total sample:** 21,295 patients.	Secondary prevention programs	☐ BEHA<br>☐ CVR<br>√ DIET (-)<br>√ EXERCISE (-)<br>☐ MED<br>√ PSY (-)<br>☐ SMOKING<br>☐ SELF	Nurse, multidisciplinary team, or did not describe.	**Number of session:** 1-12 or unclear; **Total contact hours:** did not describe. **Duration:** 0.75-48 months.	**Strategies:** Face to face, telephone contact and home visit; **Format:** Group and individual or unclear; **Theoretical approach:** Unclear.	Inpatient settings, post discharge, other	↓ **Mortality,**<br>↓ **MI,**<br>− Hospitalization rates.	Performed analyses by using Review Manager 4.2 and Qualitative Data Synthesis

NOTE:

For peer review only

SMOKING= Smoking cessation; CVR= Cardiovascular risk factors; PSY =Psychosocial issues (depression, anxiety); DIET =Diet; EXERCISE= Exercise; MED= Medication; BEHA = Behavioral charge (including lifestyle modification); SELF = Self-management (including problems solving); DR =Diabetes risks; CHD= Coronary heart disease; CAD = Coronary Artery Disease; CHW=Community Health Worker; HbA1c = Glycatedhemoglobin; BP =Blood Pressure; LDL = Low-density Lipoprotein Cholesterol; SMS = Short Message Service; BCTs = Behavioral Change Techniques; LEA = Lower Extremity Amputation; PRIDE = Problem Identification, Researching one's routine, Identifying a management goal, Developing a plan to reach it, Expressing one's reactions and Establishing rewards for making progress; ASE = Attitude-social influence-efficacy; CVRF= Cardiovascular Risk Factors; SMS = Short Message System; PA = Physical Activity; EDU = Patient Education; GP= General Practice; RCTs =Randomized controlled trials; CCTS = controlled clinical trials; HRQoL = Health Related Quality of Life; QoL = Quality of Life; MI = Myocardial Infarction; CAD = Coronary Artery Disease; CABG= Coronary Artery Bypass Graft Surgery; BMI = Body Mass Index; HbA1c = Glycatedhemoglobin; BP =Blood Pressure; SBP = Systolic Blood Pressure, DBP = Diastolic Blood Pressure, HDL-c= High-density Lipoprotein Cholesterol; LDL-c = Low-density Lipoprotein Cholesterol; TTM= Transtheoretical Model; SCT=Social Cognitive Theory; HBM =Health Belief Model; SAT=Social Action Theory;

In the educational content: “+”: minor focus; “++”:moderate focus; “+++” major focus; “-“ =unclear what the intensity of the education was for any topic;

In the outcomes: arrow up (“ ↑ ”) for improvement, arrow down (“ ↓ ”) for reduction; a dash (“−”) for no change or inconclusive evidence. Primary outcomes were bold.

For peer review only

Table 3. Characteristics and interventions of included systematic reviews and meta-analysis involved T2DM patients

(First author, Year); Journal	Primary objectives (To assess effect of interventions on….)	Studies details	Intervention: Educational Content		Intervention: Provider	Intervention: Number of session(s), delivery mode, time, setting			Outcomes (Primary outcomes were bold.) "–" : No change "↑" : Increase "↓" : decrease	Synthesis methods
(Choi, Davidson, Walker, Lee, & Palermo, 2016) [40]; Diabetes Research and Clinical Practice	Glycemic effect	**Number of studies:** 53 studies(five in English, 48 in Chinese); **Types of studies:** RCTs; **Total sample:** Unclear.	Diabetes education intervention.	☐ BEHA<br>√ DIET (-)<br>☐ DR<br>☐ EXERCISE<br>☐ GC<br>☐ MED<br>☐ PSY<br>☐ SMOKING<br>√ SELF (-)	Unclear, did not describe.	**Number of session:** Unclear; **Total contact hours:** Unclear; **Duration:** 30–150 min or unclear.	**Strategies:** Face to face, written materials; telephone contact and home visit; **Format:** Unclear; **Theoretical approach:** Unclear.	Inpatient settings, post discharge, other	↓ **HbA1c (glycated haemoglobin).**	STATAversion 12 and Review Manager version 5.3
(Creamer, Attridge, Ramsden, Cannings-John, & Hawthorne, 2016) [42]; Diabetic Medicine	successful outcomes and to suggest directions for future research.	**Number of studies:** **33;** **Types of studies:** RCTs; **Total sample:** 7,453 participants.	Culturally appropriate health education.	√ BEHA (-)<br>√ DIET (-)<br>√ DR (-)<br>√ EXERCISE<br>☐ GC<br>☐ MED<br>☐ PSY<br>☐ SMOKING<br>√ SELF (-)	CHWs, clinical pharmacists dieticians, Nurses, podiatrists, physiotherapists, and psychologists.	**Number of session:** one to ten or unclear; **Total contact hours:** unclear; **Duration:** from a single session to 24 months.	**Strategies:** Face-to-face; Phone contact; **Format:** group sessions (10 studies), individual sessions (13) or a combination of both; **Theoretical approach:** Unclear.	Inpatient settings, post discharge, other	↓ **HbA1c,**<br>– **HRQoL,**<br>– **Adverse events,**<br>– BP,<br>– BMI,<br>– Lipid levels,<br>– diabetes complications,<br>– Economic analyses,<br>– Mortality and diabetes knowledge,<br>– Empowerment,<br>– Self-efficacy and satisfaction.	Meta-analysis using the Review Manager statistical program.

For peer review only

(Huang et al., 2016) [55]; European Journal of Internal Medicine	Clinical markers of cardiovascular disease	**Number of studies:** 17 studies;<br>**Types of studies:** RCTs;<br>**Total sample:** Unclear.	Lifestyle interventions.	☐ BEHA<br>√ DIET (-)<br>√ CVR (-)<br>√ EXERCISE (-)<br>☐ GC<br>☐ MED<br>☐ PSY<br>☐ SMOKING<br>√ SELF (-)	Nurse, Pharmacist or unclear.	**Number of session:** Unclear;<br>**Total contact hours:** Unclear;<br>**Duration:** 6 month – 8 years.	**Strategies:** Unclear;<br>**Format:** Individual; Group and Mixed.<br>**Theoretical approach:** Unclear;	Unclear, did not describe.	**Cardiovascular risk factors** (such as,<br>– BMI,<br>↓ HbA1c,<br>– BP,<br>↓ The level of cholesterol.)	Review Manager 5.1.
(Chen et al., 2015) [39]; Metabolism-Clinical and Experimental	Clinical markers.	**Number of studies:** 16 studies;<br>**Types of studies:** RCTs;<br>**Total sample:** per study ranged from 23 to 2575;	Lifestyle intervention.	√ BEHA (-)<br>☐ DIET<br>√ CVR (-)<br>☐ EXERCISE<br>☐ GC<br>√ MED (-)<br>☐ PSY<br>☐ SMOKING<br>√ SELF (-)	Unclear, did not describe.	**Number of session:** Monthly;<br>**Total contact hours:** Unclear;<br>**Duration:** < 6 months -8 years.	**Strategies:** Unclear;<br>**Format:** Individual; Group and Mixed;<br>**Theoretical approach:** Unclear.	Unclear, did not describe.	**Cardiovascular risk factors** including<br>↓ BMI,<br>↓ HbA1c,<br>↓ SBP, DBP,<br>– HDL-c, and LDL-c.	All analyses were performed using Comprehensive Meta-Analysis statistical software.
(Terranova, Brakenridge, Lawler, Eakin, & Reeves, 2015) [72]; Diabetes, Obesity and Metabolism	Weight loss.	**Number of studies:** 10 individual studies (from 13 papers);<br>**Types of studies:** RCTs;<br>**Total sample:** ranging from 27 to 5145 participants.	Lifestyle-based-only intervention.	√ BEHA (-)<br>√ DIET (-)<br>☐ DR (-)<br>√ EXERCISE (-)<br>☐ GC<br>☐ MED<br>☐ PSY<br>☐ SMOKING<br>√ SELF (-)	Dietician; diabetes educator; general physician; multidisciplinary team; or nutritionist; nurse.	**Number of session:** 1-42;<br>**Total contact hours:** Unclear;<br>**Duration:** ranged from 16 weeks to 9 years.	**Strategies and Format:** Face-to-face individual or group-based sessions, or a combination of those. One study delivered the intervention via the telephone<br>**Theoretical approach:** Unclear.	Unclear, did not describe.	↓ **Weight change;**<br>– **HbA1c.**	Meta-analyses - Review Manager and meta-regression analysis - stata version
(Pillay et al., 2015) [71]; Annals of	HbAlc level.	**Number of studies:** 132;<br>**Types of studies:**	Behavioral Programs.	√ BEHA (-)<br>√ DIET (-)<br>☐ DR	Trained individuals.	**Number of session:** Unclear;<br>**Total contact hours:**	**Strategies:** Unclear;<br>**Format:** Unclear;<br>**Theoretical approach:** Unclear.	Inpatient settings, post discharge,	– **HbA1c;**<br>↓ **BMI.**	The analysis was conducted

For peer review only

internal medicine		RCTs;<br>**Total sample:** Unclear.		√ EXERCISE (-)<br>√ GC (-)<br>√ MED (-)<br>☐ PSY<br>☐ SMOKING<br>√ SELF (-)		range, 7 to 40.5 hours;<br>**Duration:** 4 or more weeks.		other		by using a Bayesian network model.
(Pal et al., 2014) [67]; Diabetes Care	Health status, cardiovascular risk factors, and QoL.	**Number of studies:** 20 papers describing 16 studies;<br>**Types of studies:** RCTs;<br>**Total sample:** 3,578 participants.	Computer-based self-management interventions.	☐ BEHA<br>☐ DIET<br>☐ DR<br>☐ EXERCISE<br>☐ GC<br>☐ MED<br>☐ PSY<br>☐ SMOKING<br>√ SELF	Unclear, did not describe.	**Number of session:** 1-8 ;<br>**Total contact hours:** 10 mins - 6 hours;<br>**Duration:** 8 weeks – 12 months;	**Strategies:** Online/web-based; Phone contact.<br>**Format:** Individual; Group and Mixed.<br>**Theoretical approach:** TTM, social ecological theory, SCT, and self-determination theory.	Unclear, did not describe.	**– HRQoL,**<br>**↓ HbA1c,**<br>**– Death;**<br>↓ Cognitions, behaviors,<br>– Social support,<br>↓ Cardiovascular risk factors,<br>– Complications,<br>– Emotional outcomes,<br>– Hypoglycemia,<br>– Adverse effects,<br>– CE and ecfonomic data.	Meta-analysis using Review Manager software or narrative presentatio.
(Ricci-Cabello et al., 2014) [73]; BMC endocrine disorders	Knowledge, behaviors and clinical outcomes.	**Number of studies:** Thirty-seven studies;<br>**Types of studies:** Almost two thirds of the studies were RCTs, 27% studies were Quasi-experimental design.<br>**Total sample:** Unclear.	DSM educational programs.	☐ BEHA<br>√ DIET(+++)<br>☐ DR<br>√ EXERCISE (+++)<br>√ GC(+++)<br>√ MED(++)<br>√ PSY(++)<br>☐ SMOKING<br>☐ SELF	Dietitian; Nurse; Psychologist; Physician; Research team or staff;	**Number of session:** 13.1;<br>**Total contact hours:** 0.25-180 hours;<br>**Duration:** 0.25-48 months.	**Strategies:** Face-to-face; Telecommunication; Both.<br>**Format:** One on one; Group and Mixed.<br>**Theoretical approach:** Unclear.	Post discharge, other	**– Diabetes knowledge;**<br>**– Self-management;**<br>**– Behaviors;**<br>**– Clinical outcomes;**<br>↓ Glycated hemoglobin;<br>– Cost-effectiveness analysis.	Meta-analyses and bivariate meta-regression were conducted with Stata, version 12.0.

For peer review only

For peer review only

(Saffari, Ghanizadeh, & Koenig, 2014) [74]; Primary Care Diabetes	Glycemic control.	**Number of studies:** 10;<br>**Types of studies:** RCTs;<br>**Total sample:** 960 patients.	An educational intervention using SMS.	√ BEHA (-)<br>☐ DIET<br>☐ DR<br>☐ EXERCISE<br>√ GC (-)<br>√ MED (-)<br>☐ PSY<br>☐ SMOKING<br>☐ SELF	Unclear, did not describe.	**Number of session:** Weekly; or 2 messages daily or unclear;<br>**Total contact hours:** Unclear.<br>**Duration:** 3 months – 1 year	**Strategies:** SMS: sending and receiving data. Receive data through text-messaging by patients only. Used a website along with SMS;<br>**Format:** Unclear;<br>**Theoretical approach:** Unclear.	Inpatient settings, post discharge, other	↑ **Glycemic control**.	Comprehensive Meta-analysis Software Version 2.0.
(Odnoletkova et al., 2014) [66]; Journal of Diabetes & Metabolism	Cost-effectiveness (CE).	**Number of studies:** Seventeen studies;<br>**Types of studies:** RCTs;<br>**Total sample:** Unclear.	Therapeutic education.	√ BEHA (-)<br>☐ DIET<br>☐ DR<br>☐ EXERCISE<br>☐ GC<br>☐ MED<br>☐ PSY<br>☐ SMOKING<br>√ SELF (-)	General physician; nutritionists or unclear.	**Number of session:** ~16;<br>**Total contact hours:** Unclear;<br>**Duration:** Unclear.	**Strategies:** Face-to-face or unclear;<br>**Format:** individual and group lessons;<br>**Theoretical approach:** Unclear.	Inhospital or unclear.	− **CE.**	Incremental Cost-Effectiveness Ratio.
(Attridge, Creamer, Ramsden, Cannings-John, & Hawthorne, 2014) [33]; The Cochrane Library	HbAlc level, Knowledge and clinical outcomes.	**Number of studies:** 33 trials;<br>**Types of studies:** RCTs and quasi-RCTs;<br>**Total sample:** 7453 participants.	'Culturally appropriate' health education.	√ BEHA (-)<br>√ DIET (-)<br>☐ DR<br>√ EXERCISE (-)<br>√ GC (-)<br>☐ MED<br>☐ PSY<br>√ SMOKING (-)<br>☐ SELF	CHWs; dieticians; exercise physiologists; lay workers; nurses; podiatrists; and psychologists;.	**Number of session:** one session to 24 months;<br>**Total contact hours:** Unclear;<br>**Duration:** The median duration of interventions was six months.	**Strategies:**<br>**Format:** Group intervention method, one-to-one sessions and a mixture of the two methods. Or a purely interactive patient-centred method.<br>**Theoretical approach:** empowerment theories; behaviour change theories, TTM of behaviour change and SCT.	Inpatient settings, post discharge, other	↓ **HbA1c;**<br>− **HRQoL;**<br>− **Adverse events;**<br>− Mortality;<br>− Complications;<br>−Satisfaction; ↑ Empowerment;<br>↑ Self-efficacy;<br>− Attitude; knowledge;	Meta-analyses used Review Manager software.

									−BP;<br>−BMI;<br>↓ Lipid levels;<br>−Health economics.	
(Vugt, Wit, Cleijne, & Snoek, 2013) [75]; Journal of Medical Internet Research	Health outcomes.	**Number of studies:** 13 studies;<br>**Types of studies:** RCTs;<br>**Total sample:** 3813 patients.	BCTs are being used in online self-management interventions.	√ BEHA (-)<br>☐ DIET<br>☐ DR<br>☐ EXERCISE<br>☐ GC<br>☐ MED<br>☐ PSY<br>☐ SMOKING<br>√ SELF (-)	Health care professional.	**Number of session:** 6 weekly sessions or unclear;<br>**Total contact hours:** Unclear;<br>**Duration:** Unclear.	**Strategies:** Online/web-based;<br>**Format:** Unclear;<br>**Theoretical approach:** self-efficacy theory, social support theory, TTM, SCT, social-ecological model, and cognitive behavioral therapy.	Post discharge	**− Health behavior change,**<br>**− Psychological well-being;**<br>− Clinical parameters.	Unclear.
(Gucciardi, Chan, Manuel, & Sidani, 2013) [52]; Patient Education and Counseling	HbAlc level,physical activity and diet outcomes.	**Number of studies:** Thirteen studies;<br>**Types of studies:** RCTs and comparative studies;<br>**Total sample:** Unclear.	DSME interventions.	☐ BEHA<br>√ DIET (+++);<br>☐ DR<br>√ EXERCISE (+++);<br>☐ GC<br>√ MED (+);<br>√ PSY (+)<br>☐ SMOKING<br>√ SELF (++).	Dietitians (n = 7/13); Multidisciplinary Team (n = 7/13); Nurse (n = 5/13); Community Peer Worker (n = 3/13).	**Number of session:** Low Intensity: <10 education sessions (n = 7); High Intensity: ≥ 10 education sessions (n = 6);<br>**Total contact hours:** Unclear;<br>**Duration:** < 6 months (n = 7/13); ⩾6months (n = 6/13).	**Strategies:** Face-to-face(n = 13/13); Written Literature: (e.g., handbook) (n = 4/13);Telephone(n = 4/13);Audio-Visual(n = 1/13);<br>**Format:** One-on-one: (n = 11/13);Group (n = 9/13);<br>**Theoretical approach:** SAT; Empowerment Behavior Change Model; modification theories; Pharmaceutical Care Model; Behavior Change Theory; PATHWAYS Program; Symptom-Focused Management Model; Motivational interviewing.	Inpatient settings, post discharge	**− HbA1c levels,**<br>− Anthropometrics,<br>− Physical activity;<br>− Diet outcomes.	Arecently described method.
(Pal et al., 2013) [68]; The Cochrane Library	Health status and HRQoL.	**Number of studies:** 16 studies;<br>**Types of studies:** RCTs;<br>**Total sample:** 3,578	Computer-based diabetes self-management intervention.	☐ BEHA<br>√ DIET (-)<br>☐ DR<br>√ EXERCISE (-)<br>√ GC (-)	Nurse or other healthcare professionals.	**Number of session:** Unclear;<br>**Total contact hours:** Unclear;<br>**Duration:** One session –	**Strategies:** Online/web-based; phone contact<br>**Format:** Unclear;<br>**Theoretical approach:** Unclear.	Inpatient settings, post discharge, other	**− HRQoL;**<br>**− Death from any cause;**<br>**↓ HbA1c;**	Formal meta-analyses and narrative

For peer review only

		participants.		√ MED (-)<br>√ PSY (-)<br>☐ SMOKING<br>☐ SELF		18 months.			− Cognitions;<br>− Behaviours;<br>−Social support;<br>−Biological markers;<br>− Complications.	synthesis.
(Nam, Janson, Stotts, Chesla, & Kroon, 2012) [62]; Journal of Cardiovascular Nursing	Glycemic control.	**Number of studies:** 12 RCTs;<br>**Types of studies:** RCTs;<br>**Total sample:** 1,495 participants.	Diabetes educational interventions (no drug intervention).	☐ BEHA<br>√ DIET (-)<br>☐ DR<br>√ EXERCISE (-)<br>√ GC (-)<br>√ MED (-)<br>√ PSY (-)<br>☐ SMOKING<br>√ SELF (-)	Nurses (36%), dieticians (36%), diabetes educators (5%), other professionals (9%) and nonprofessional staff (14%).	**Number of session:** 1 month or less; 1 to 3 months; and 12 months;<br>**Total contact hours:** most studies did not describe, or from 1 session to more than 30 hours;<br>**Duration:** from 1 session to 12 months, frequency: 1 session to 25 weekly or biweekly education.	**Strategies:** Teaching or counseling; home-based support; and visual aids.<br>**Format:** group education or a combination of group education and individual counseling; or only individual counseling;<br>**Theoretical approach:** Unclear.	Inpatient settings, post discharge, other	↓ **HbA1c level.**	Meta-analysis.
(Steinsbekk, Rygg, Lisulo, Rise, & Fretheim, 2012) [76]; BMC Health Services Research	Clinical, lifestyle and psychosocial outcomes.	**Number of studies:** 21 studies (26 publications).<br>**Types of studies:** RCTs;<br>**Total sample:** 2,833 participants.	Group-based education.	Did not describe the content of the intervention.	Community workers; dietician; lay health advisors nurse and nutritionist.	**Number of session and Total contact hours:** 30 hours over 2.5 months, 52 hours over one year,and 36 hours or 96 hours over 6 months.<br>**Duration:** Six months to two years.	**Strategies:** Face to face;<br>**Format:** 5 to 8 participants group to 40 patients group.<br>**Theoretical approach:** empowerment model and the discovery learning theory, the SCT and the social ecological theory, the self-efficacy and self-management theories and operant reinforcement theory.	Inpatient settings, post discharge, other	↓ **HbA1c,**<br>↑ **Lifestyle outcomes,**<br>↑ **Diabetes knowledge,**<br>↑ Self-management skills,<br>↑ Psychosocial outcomes,<br>↓ Mortality rate,<br>↓ BMI,<br>↓ Blood pressure;<br>↓ Lipid profile.	Meta-analysis using Review manager v5.
(Amaeshi,	Increasing	**Number of studies:**	Foot health	Food care.	Podiatrist,	**Number of session:**	**Strategies:** Face to face;	Unclear, did	↓ **LEA;**	Narrative

For peer review only

2012) [32]; Podiatry Now	good foot health practices that will ultimately reduce LEA.	8 studies; **Types of studies:** RCT or clinical controlled trial (CCT); **Total sample:** Unclear.	education:		psychotogist or unclear.	Unclear; **Total contact hours:** between 15min and 14 hours; **Duration:** 3-30 months.	**Format:** In three of the studies, educational interventions were delivered to the participants in groups, while the other five provided individualized (one-to-one) foot-care education to the participants; **Theoretical approach:** Unclear.	not describe.	↑ Self-care.	synthesis.
Kai Lun Gan, Brammer, & Creedy, 2011) [57]; JBI Library of Systematic Reviews	Oral hypoglycaemic adherence.	**Number of studies:** Seven studies; **Types of studies:** RCTs; **Total sample:** Unclear.	Educational interventions.	√ BEHA (-) √ DIET (-) ☐ DR √ EXERCISE (-) √ GC (-) √ MED (-) √ PSY (-) ☐ SMOKING √ SELF (-)	Nurses; pharmacists; other skilled healthcare professionals.	**Number of session:** 1-12 or unclear; **Total contact hours:** 2.5 hours or unclear; **Duration:** 4-12 months.	**Strategies:** Face to face; **Format:** Group and individual; **Theoretical approach:** Unclear.	Inpatient settings, post discharge, other	↓ **HbA1c,** − **Medication adherence;** ↓ Blood glucose; − Tablet count; − Medication containers; − Diabetes complications; − Health service utilization.	Narrative summary form.
Burke et al., 2011) [38]; JBI Database of Systematic Reviews and Implementation Reports	HbAlc level,BP.	**Number of studies:** 11 RCTs and 4 quasi-experimental trials; **Types of studies:** RCTs and quasi-experimental trials; **Total sample:** 2,240 patients.	Group medical visits.	√ BEHA (-) √ DIET (-) ☐ DR ☐ EXERCISE √ GC (-) √ MED (-) ☐ PSY ☐ SMOKING √ SELF (-)	Dndocrinologists; DM nurse; family physician; nutritionist and rehab therapist.	**Number of session:** 1-4 or unclear; **Total contact hours:** 2-4 hours or unclear; **Duration:** One session to 2 years.	**Strategies:** Face to face; **Format:** Group and individual; **Theoretical approach:** Unclear.	Inpatient settings, post discharge, other	↓ **HbA1c;** − **Systolic and diastolic BP;** − LDL measurements.	Meta-analysis.
(Ramadas, Quek, Chan, & Oldenburg, 2011) [77];	HbAlc level.	**Number of studies:** 13 different studies; **Types of studies:** RCTs and quasi-experimental studies;	Web-based behavioural interventions.	√ BEHA (-) √ DIET (-) ☐ DR ☐ EXERCISE	Dietician; endocrinologist; physicians; researchers or research staff	**Number of session:** Unclear; **Total contact hours:** Unclear; **Duration:** ranged	**Strategies:** E-mail and SMS technologies that were commonly used together with the websites to reinforce the intervention, and Website, print material.	Inpatient settings, post discharge, other	− **Self-monitoring blood sugar,** − Weight-loss, − Dietary behaviour,	Not statistically combined and re-analyzed;

For peer review only

International Journal of Medical Informatics		**Total sample:** Unclear.		√ GC (-)<br>√ MED (-)<br>☐ PSY<br>☐ SMOKING<br>√ SELF (-)	members and study nurse.	between 12 and 52 weeks, with an average of 27.2 ± 18.3 weeks.	**Format:** Unclear;<br>**Theoretical approach:** Wagner's Chronic Care Model; Self-efficacy theory/Social support theory; TTM; HBM; SCT.		− Physical activity.	
(Minet, Moller, Vach, Wagner, & Henriksen, 2010) [61];<br>Patient Education and Counseling	Glycaemic control.	**Number of studies:** Forty-seven studies;<br>**Types of studies:** RCTs;<br>**Total sample:** Unclear.	Self-care management interventions.	√ BEHA (-)<br>☐ DIET<br>☐ DR<br>☐ EXERCISE<br>☐ GC<br>☐ MED<br>☐ PSY<br>☐ SMOKING<br>√ SELF (-)	Case nurse manager; group facilitator; nurse educator; multidisciplinary team; physiologist; physician; peer counsellor; researcher; and pharmacist.	**Number of session:** 3-26;<br>**Total contact hours:** Unclear;<br>**Duration:** 4 weeks to 4 years.	**Strategies:** Face to face; home visit; phone calls;<br>**Format:** Group and individual;<br>**Theoretical approach:** Unclear.	Inpatient settings, post discharge, other	↓ **HbA1c.**	Meta-analyses and meta-regression used Stata's meta command.
(Hawthorne, Robles, Cannings-John, & Edwards, 2010) [54];<br>Diabetic Medicine	Effects of culturally appropriate health education	**Number of studies:** 10 trials;<br>**Types of studies:** RCTs;<br>**Total sample:** 1603 patients.	Culturally appropriate health education	☐ BEHA<br>√ DIET (-)<br>☐ DR<br>√ EXERCISE (-)<br>☐ GC<br>☐ MED<br>☐ PSY<br>☐ SMOKING<br>√ SELF (-)	Exercise physiologists; dieticians; diabetes nurses; link workers, and podiatrists.	**Number of session:** Unclear;<br>**Total contact hours:** Unclear;<br>**Duration:** one session to 12 months;	**Strategies:** Face to face; visual aids, leaflets and teaching materials;<br>**Format:** group approach, one-to-one interviews and a mixed approach;<br>**Theoretical approach:** SAT, Empowerment Behaviour Change Model, SCT, Management model and the Theory of Planned Behaviour.	Inpatient settings, post discharge, other	**−QoL;**<br>↓ **HbA1c;**<br>− **BP;**<br>↑ Knowledge;<br>− BMI;<br>↓ Lipid levels,<br>− Diabetic complications,<br>− Mortality rates, hospital admissions, hypoglycaemia.	Meta - analysis using the Review Manager and narrative review.
(Fan & Sidani, 2009) [47];<br>Canadian	Knowledge, self-manageme nt	**Number of studies:**<br>**50 studies;**<br>**Types of studies:** RCTs;	DSME intervention:	√ BEHA (-)<br>☐ DIET<br>☐ DR<br>☐ EXERCISE	Unclear, did not describe.	**Number of session:** 10 (range 1 to 28);<br>**Total contact hours:** 17 contact hours (range 1 to	**Strategies:** Online/web-based (4%); video (2%); face-to-face (60%); phone contact (4%); Mixed (30%).<br>**Format:** One-on-one (32%); Group	Inpatient settings, post discharge, other	↑ **Diabetes knowledge,**<br>↑ **Self-management behaviours;**	Comprehens ive Meta-Analysis (version

For peer review only

Journal of Diabetes	behaviours and metabolic control.	**Total sample:** **Unclear.**		☐ GC<br>☐ MED<br>√ PSY (-)<br>☐ SMOKING<br>√ SELF (-)		52); ⩽ 10 (46%); 11-20 (21%); > 20 (33%);<br>**Duration:** 22 weeks (range 1 to 48); ⩽ 8 weeks (26%); 9–24 weeks (37%); > 24 weeks (37%).	(40%); Mixed (28%).<br>**Theoretical approach:** Unclear.		↓ **HbA1c,**	2.0).
(Duke, Colagiuri, & Colagiuri, 2009) [46]; The Cochrane Library	Metabolic control, diabetes knowledge and psychosocial outcomes.	**Number of studies:** Nine studies;<br>**Types of studies:** RCTs;<br>**Total sample:** 1,359 participants.	Individual patient education.	√ BEHA (-)<br>☐ DIET<br>☐ DR<br>√ EXERCISE (-)<br>√ GC (-)<br>☐ MED<br>√ PSY (-)<br>☐ SMOKING<br>☐ SELF	Diabetes educators and dieticians.	**Number of session:** 1-6;<br>**Total contact hours:** 20 min – 7 hours;<br>**Duration:** 4 weeks – 1 year.	**Strategies:** Face to face; telephone;<br>**Format:** Individual;<br>**Theoretical approach:** Unclear.	Inpatient settings	− **HbA1c;**<br>− **Diabetes complications;**<br>− Health service utilisation and health care costs;<br>− Psychosocial outcomes;<br>− Diabetes knowledge; patient self-care behaviours;<br>− Physical measures; metabolic.	Meta-analysis.
(Alam, Sturt, Lall, & Winkley, 2009) [30]; Patient Education and Counseling	Glycaemic control and psychological status.	**Number of studies:** 35 trials;<br>**Types of studies:** RCTs;<br>**Total sample:** 1431 patients.	Psycho-educational Interventions.	√ BEHA (-)<br>☐ DIET<br>☐ DR<br>☐ EXERCISE<br>☐ GC<br>☐ MED<br>√ PSY (-)<br>☐ SMOKING<br>☐ SELF	Generalists; psychological specialists; or did not report the specialist	**Number of session:** 1-16;<br>**Total contact hours:** 20 min – 28 hours;<br>**Duration:** about 13.7 (±11.06) weeks.	**Strategies:** Face to face; telephone calls;<br>**Format:** group format; a single format and used a combination;<br>**Theoretical approach:** TTM; motivational interviewing;	Inpatient settings, other	↓ **HbAlc;**<br>↓ **Psychological distress.**	Meta-analysis.
(Khunti, Camosso-	Knowledge and	**Number of studies:** nine studies;	Any educational	☐ BEHA<br>√ DIET<br>☐	Unclear, did not describe.	**Number of session:** Unclear;	**Strategies:** Face to face;<br>**Format:** Group and individual;	Unclear, did not describe.	− **Knowledge;**<br>− **Psychological and**	Unclear.

For peer review only

Ste[illegible]novic, Carey, Davies, & Stone, 2008) [58]; Diabetic Medicine	biomedical outcomes.	**Types of studies:** RCTs and RCT was followed by a before-and-after study; **Total sample:** 1004 patients.	intervention.	DR ☐ EXERCISE ☐ GC ☐ MED ☐ PSY ☐ SMOKING ☐ SELF		**Total contact hours:** Unclear; **Duration:** 3- 12 months.	**Theoretical approach:** Unclear.		**biomedical outcome measures.**	
(Loveman, Frampton, & Clegg, 2008) [60]; Health Technology Assessment	Clinical effectiveness.	**Number of studies:** Twenty-one published trials; **Types of studies:** RCTs and CCTs; **Total sample:** Unclear.	Educational interventions.	√ BEHA (++) √ DIET (+++) ☐ DR √ EXERCISE (+++) √ GC (+++) ☐ MED ☐ PSY ☐ SMOKING √ SELF (+++)	Community workers; diabetes research technician; diabetes nurse, dieticians; educationalist; medical students; nurses; pharmacists; physician, or physician assistant.	**Number of session:** Two to four intensive education of 1.5 to 2 hours followed up with additional education at, 3 and 6 months; **Total contact hours and Duration:** about 150 mins over a 6-month or 61 to 52 hours over one year.	**Strategies:** Face to face; **Format:** Group and individual; **Theoretical approach:** cognitive-behavioural strategies; pedagogical principle.	Inpatient settings, post discharge, other	**− Diabetic control outcomes**; − Diabetic end-points; − QoL and cognitive measures.	Narrative review
(Wens et al., 2008) [78]; Diabetes Research and Clinical Practice	Improving adherence to medical treatment recommendations.	**Number of studies:** 8 studies; **Types of studies:** RCTs and controlling before and after studies. **Total sample:** 772 patients.	Interventions aimed at improving adherence to medical treatment.	√ BEHA (-) √ DIET (-) ☐ DR √ EXERCISE (-) √ GC (-) √ MED (-) ☐ PSY ☐ SMOKING √ SELF (-)	Diabetes educator; nurse or did not describe.	**Number of session:** Unclear; **Total contact hours:** Unclear; **Duration:** ～9 months or unclear.	**Strategies:** Face-to-face; telephone; **Format:** face-to-face; group based and telemedicine; **Theoretical approach:** Unclear.	Inpatient settings, post discharge, other	**− Adherence;** − HbA1c; − Blood glucose.	Cochrane Review Manager software.

For peer review only - http://bmjopen.bmj.com/site/about/guidelines.xhtml

(Hawthorne, Robles, Cannings-John, & Edwards, 2008) [53]; The Cochrane Library	HbAlc level, Knowledge and clinical outcomes.	**Number of studies:** A total of 11 trials;<br>**Types of studies:** RCTs;<br>**Total sample:** 1,603 patients.	Culturally appropriate (or adapted) health education.	√ BEHA (-)<br>√ DIET (-)<br>☐ DR<br>√ EXERCISE (-)<br>√ GC (-)<br>☐ MED<br>☐ PSY<br>√ SMOKING (-)<br>☐ SELF	Dieticians, diabetes nurses, exercise physiologists; link workers; podiatrists; psychologist and and non-professional link worker.	**Number of session:** Unclear;<br>**Total contact hours:** Unclear;<br>**Duration:** one session to 12 months.	**Strategies:** Face-to-face; booklet;<br>**Format:** Group intervention method; one to one interviews; mixture of the two methods; purely interactive patient-centred method; semi-structured didactic format and combination of the 2 approaches.<br>**Theoretical approach:** SAT; Empowerment Behaviour Change Model; Behaviour Change Theory; SCT, Management Model and the Theory of Planned Behaviour.	Inpatient settings, post discharge, other	↓ **HbA1c**<br>↑ **Knowledge scores**<br>− Other outcome measures	Narrative presentation and meta-analysis.
(Nield et al., 2007) [63]; The Cochrane Library	Metablic control.	**Number of studies:** 36 articles (18 trials);<br>**Types of studies:** RCTs;<br>**Total sample:** 1,467 participants.	Dietary advice.	☐ BEHA<br>√ DIET<br>☐ DR<br>☐ EXERCISE<br>☐ GC<br>☐ MED<br>☐ PSY<br>☐ SMOKING<br>☐ SELF	exercise physiologist; dietitian; group facilitator; nutritionist; nurse educator; and physician.	**Number of session:** 1-12;<br>**Total contact hours:** 20 min-22 hours;<br>**Duration:** 11 weeks – 6months or unclear.	**Strategies:** Face-to-face;<br>**Format:** Group and individual;<br>**Theoretical approach:** Unclear.	Inpatient settings, post discharge, other	− **Weight;**<br>− **Diabetic complications;**<br>− **HbA1c;**<br>− QoL;<br>− Medication use;<br>− Cardiovascular disease risk;	Meta-analysis.
(Zabaleta & Forbes, 2007) [79]; British Journal of Community Nursing	Clinical effectivenes s.	**Number of studies:** 21 studies;<br>**Types of studies:** Controlled trials;<br>**Total sample:** Unclear.	Structured group diabetes education.	√ BEHA (-)<br>√ DIET (-)<br>☐ DR<br>√ EXERCISE (-)<br>√ GC (-)<br>☐ MED<br>√ PSY (-)<br>☐ SMOKING<br>☐ SELF	Diabetes nurse educator; physician's assistant; and physicians;	**Number of session:** 4-6 or unclear;<br>**Total contact hours:** 6-12 hours or unclear;<br>**Duration:** 1-6 months or unclear.	**Strategies:** Face to face;<br>**Format:** Group;<br>**Theoretical approach:** Unclear.	Post discharge.	−**HbA1c.**	A tabulative synthesis.

For peer review only

(Deakin, McShane, Cade, & Williams, 2005) [43]; The Cochrane Library	Clinical, lifestyle and psychosocial outcomes.	**Number of studies:** 14 publications, reporting 11 studies;<br>**Types of studies:** RCTs, and CCTs;<br>**Total sample:** 1,532 participants.	Group-based educational programmes.	Did not describe the content of the intervention.	Health professionals, lay health advisors.	**Number of session:** Unclear;<br>**Total contact hours:** from 6 to 52 hours;<br>**Duration:** 3 hours per year for 2 years and 3 or 4 hours per year for 4 years.	**Strategies:** Unclear;<br>**Format:** Group;<br>**Theoretical approach:** the Diabetes Treatment and Teaching Programme (DTTP); empowerment model; adult learning model, public health model, HBM and TTM.	Inpatient settings, post discharge	↓ **Metabolic control;**<br>↑ **Diabetes knowledge;**<br>↑ QoL;<br>↑ Empowerment/self-efficacy.	Summarized statistically
(Vermeire et al., 2005) [80]; The Cochrane Library	Improving adherence to treatment recommendations.	**Number of studies:** 21 articles;<br>**Types of studies:** RCTs; cross-over study; controlled trial; controlled before and after studies;<br>**Total sample:** 4,135 patients.	Interventions that were aimed at improving the adherence to treatment recommendations.	☐ BEHA<br>☐ DIET<br>☐ DR<br>☐ EXERCISE<br>√ GC (-)<br>√ MED (-)<br>☐ PSY<br>☐ SMOKING<br>☐ SELF	Nurse, pharmacist and other healthcare professionals.	**Number of session:** Unclear;<br>**Total contact hours:** Unclear;<br>**Duration:** Unclear.	**Strategies:** Face to face; telephone; home visit; video; mailed educational materials;<br>**Format:** Unclear.<br>**Theoretical approach:** Unclear.	Inpatient settings, post discharge	**Direct indicators,** such as<br>↓ Blood glucose level;<br>− Indirect indicators, such as pill counts;<br>−Health outcomes.	A descriptive review and sub-group meta-analysis.
(Gary, Genkinger, Guallar, Peyrot, & Brancati, 2003) [49]; Diabetes Educator	Body weight and glycaemic control.	**Number of studies:** Sixty-three RCTs;<br>**Types of studies:** RCTs;<br>**Total sample:** 2,720 patients.	Educational and behavioural component interventions.	☐ BEHA<br>√ DIET (-)<br>☐ DR<br>√ EXERCISE (-)<br>√ GC (-)<br>√ MED (-)<br>☐ PSY<br>☐ SMOKING<br>☐ SELF	Nurse (39%); dietitian (26%); physician (17%); other or not specified (23%); other professional (13%); psychologist (9%); Exercise psychologist (9%); and health educator (4%).	**Number of session:** Unclear;<br>**Total contact hours:** Unclear.<br>**Duration:** 1 month to 19.2 months.	**Strategies:** Unclear;<br>**Format:** Unclear;<br>**Theoretical approach:** SAT, contracting model and patient empowerment.	Inpatient settings, post discharge	− **Glycaemic control;**<br>− **Weight.**	Sufficient data were combined using meta-analysis.
(Norris, Lau, Smith,	Total GHb.	**Number of studies:** 31 studies	Self-management	√ BEHA (-)<br>√ DIET (-)	Dietitian; lay health care	**Number of session:** 6 (1–36);	**Strategies:** Online/web-based; video ; face-to-face; phone contact;	Inpatient settings, post	↓ **Total GHb,**	Meta-analysis and

For peer review only - http://bmjopen.bmj.com/site/about/guidelines.xhtml

Schmid, & Engelgau, 2002) [65]; Diabetes Care		**Types of studies:** RCTs. **Total sample:** 4,263 patients.	education.	☐DR ☐EXERCISE ☐GC ☐MED ☐PSY ☐SMOKING √ SELF (-)	worker; nurse; physician with team; self (e.g. computer assisted instruction); and team (nurse, dietitian, etc.).	**Total contact hours:** 9.2 (1–28) hours; **Duration:** 6 (1.0–27) months.	**Format: Group;** individual and mixed; **Theoretical approach:** Unclear	discharge, other		meta-regression.
(Norris, Engelgau, & Narayan, 2001) [64]; Diabetes Care	Clinical outcomes,Knowledge, Metablic control.	**Number of studies:** 72 studies (84 papers); **Types of studies:** RCTs; **Total sample:** Unclear.	Self-management training interventions.	√ BEHA (-) √ DIET (-) ☐DR ☐EXERCISE ☐GC ☐MED ☐PSY ☐SMOKING √ SELF (-)	CHWs; nurse; or other healthcare professionals.	**Number of session:** 1-16; **Total contact hours:** ~22 hours; **Duration:** ~26 months.	**Strategies:** Online/web-based; video (2%); face-to-face; phone contact; **Format:** Group; individual and mixed; **Theoretical approach:** SAT; Fishbein and Ajzen HBM.	Inpatient settings, post discharge, other	↑ **Knowledge;** ↑ **Lifestyle behaviors;** **−Psychological and QoL outcomes;** ↑ **Glycemic control;** − Cardiovascular disease risk factors.	Outcomes are summarized in a qualitative fashion.

NOTE:

SMOKING= Smoking cessation; CVR= Cardiovascular risk factors; PSY =Psychosocial issues (depression, anxiety); DIET =Diet; EXERCISE= Exercise; MED= Medication; BEHA = Behavioral charge (including lifestyle modification); SELF = Self-management (including problems solving); GC= glycemic regulation; DR =Diabetes risks; CHD= Coronary heart disease; CAD = Coronary Artery Disease; CHW=Community Health Worker; HbA1c = Glycatedhemoglobin; BP =Blood Pressure; LDL = Low-density Lipoprotein Cholesterol; SMS = Short Message Service; BCTs = Behavioral Change Techniques; LEA = Lower Extremity Amputation; PRIDE = Problem Identification, Researching one's routine, Identifying a management goal, Developing a plan to reach it, Expressing one's reactions and Establishing rewards for making progress; ASE = Attitude-social influence-efficacy; CVRF= Cardiovascular Risk Factors; SMS = Short Message System; PA = Physical Activity; EDU = Patient Education; GP= General Practice; RCTs =Randomized controlled trials; CCTS = controlled clinical trials; HRQoL = Health Related Quality of Life; QoL = Quality of Life; MI = Myocardial Infarction; CAD = Coronary Artery Disease; CABG= Coronary Artery Bypass Graft Surgery; T2DM = Type 2 Diabetes Mellitus; BMI = Body Mass Index; HbA1c = Glycatedhemoglobin; BP =Blood Pressure; SBP = Systolic Blood Pressure, DBP = Diastolic Blood Pressure, HDL-c= High-density Lipoprotein Cholesterol; LDL-c = Low-density Lipoprotein Cholesterol; DSME = Diabetes Self-management Education; DSM = Diabetes Self-management; TTM= Transtheoretical Model; SCT=Social Cognitive Theory; HBM =Health Belief Model; SAT=Social Action Theory;

In the educational content: "+": minor focus; "++":moderate focus; "+++" major focus; "-" =unclear what the intensity of the education was for any topic;

In the outcomes: arrow up (" ↑ ") for improvement, arrow down (" ↓ ") for reduction; a dash ("−") for no change or inconclusive evidence.

For peer review only

Table 4. Summary of evidence from quantitative research syntheses

Intervention	Number of systematic reviews/meta-analysis, Total participants	First author, Year	Primary Results/findings		Rating the evidence of effectiveness
**Acute Coronary Syndrome Patients**					
General health education	Six/161,997patients (Goulding et al., 2010 did not given the total sample size)	Ghisi et al., 2014 [50]	Knowledge	91% studies (+)	Some evidence
			Behavior	77%/84%/65% studies (+)	
			Psychosocial indicators	43% studies (+)	
		Brown et al., , 2013 [37]	Mortality		
			MI		
			Revascularizations		
			Hospitalizations		
			HRQoL		
			Withdrawals/drop outs		
			Healthcare utilization and costs		
		Brown et al., 2011 [70]	Total mortality		
			MI		
			CABG		
			Hospitalisations		
			HRQoL	63.6% studies (+)	
			Healthcare costs	40% studies (+)	
			Withdrawal/drop out		
		Goulding et al., 2010 [51]	Beliefs	30.08% studies (+)	
			Secondary outcomes		
			Smoking		
			Cholesterol level		

For peer review only

		Fernandez et al., 2007 [48]	Multiple risk factor modification		
		Kotb, Hsieh, & Wells, 2014 [59]	All-cause hospitalization		
			All-cause mortality		
			Smoking cessation		
			Depression		
			Systolic blood pressure		
			Low-density lipoprotein		
			Anxiety		
Psychoeducational interventions	Six/ 37,883 patients	Barth et al., 2015 [69]	Abstinence by self-report or validated		Sufficient evidence
		Dickens et al., 2013 [45]	Depression		
		Aldcroft et al., 2011 [31]	Smoking cessation		
			Physical activity		
		Huttunen-Lenz et al.,2010 [56]	Prevalent smoking cessation		
			Continuous smoking cessation		
			Total mortality		
		Barth et al., 2008 [36]	Abstinence by self-report or validated		
			Smoking status		
		Barth et al., 2006 [35]	Abstinence		
			Smoking status		
Secondary prevention educational interventions	Three/25,154 patients	Devi et al., 2015 [44]	Mortality		
			Revascularisation		
			Total Cholesterol		
			HDL cholesterol		
			Triglycerides		
			HRQOL		
			All-cause mortality		

For peer review only - http://bmjopen.bmj.com/site/about/guidelines.xhtml

(including Internet based secondary prevention)		Auer et al., 2008 [34]	Readmission rates		Some evidence
			Reinfarction rates		
			Smoking cessation rates		
		Clark et al., 2005 [41]	Mortality		
			MI		
			Quality of life	Most of the included studies (+)	
**Type 2 Diabetes Mellitus Patients**					
General health education	Five/2,319 patients (Choi et al., 2016; Loveman et al., 2008; Zabaleta et al., 2007 did not given the total sample size)	Choi et al., 2016 [40]	HbA1c		Some evidence
		Saffari et al., 2014 [74]	Glycemic control		
		Duke et al., 2009 [46]	HbA1c		
			BP		
			Knowledge, psychosocial outcomes and smoking habits	No Data	
			Diabetes complications or health service utilization and cost analysis	No Data	
		Loveman et al., 2008 [60]	Diabetic control outcomes	46.15% studies (+)	
			Weight	66.67% studies (+)	
			Cholesterol or triglycerides	40.00% studies (+)	
		Zabaleta et al., 2007 [79]	HbA1c	4.8% studies (+)	
		Creamer et al., 2016 [42]	HbA1c		
			HRQoL		
			AEs	No AEs	
		Ricci-Cabello et al., 2014 [73]	HbA1c		
			Diabetes knowledge	73.3% studies (+)	
			Behaviors	75% studies (+)	
			Clinical outcomes	Fasting blood glucose, HbA1c	

For peer review only

				and BP improved in 71%, 59%, and 57% of the studies.	
Culturally appropriate health education	Eight/ 20,622 patients (Ricci-Cabello et al., 2014 and Gucciardi et al., 2013 did not given the total sample size)	Attridge et al., 2014 [33]	HbA1c		Some evidence
			Knowledge scores		
			Clinical outcomes		
			Other outcome measures	Showed neutral effects.	
		Gucciardi et al., 2013 [52]	HbA1c levels	Three of ten studies (+)	
			Anthropometrics	Three of eleven studies (+)	
			Physical activity	One of five studies (+)	
			Diet outcomes	Two of six studies (+)	
		Nam et al., 2012 [62]	HbA1c level		
		Hawthorne et al., 2010 [54]	HbA1c		
			Knowledge scores		
		Khunti et al., 2008 [58]	Knowledge levels	Only one study reporting a significant improvement	
			Biomedical outcomes	Only one study reporting a significant improvement.	
		Hawthorne et al., 2008 [53]	HbA1c		
			Knowledge scores		
			Other outcome measures		
		Huang et al., 2016 [55]	HbA1c		
			BMI		
			LDL-c and HDL-c		
		Chen et al., 2015 [39]	HbA1c		
			BMI		
			SBP		
			DBP		
			HDL-c		

For peer review only - http://bmjopen.bmj.com/site/about/guidelines.xhtml

Lifestyle interventions + behavioral program	Six/10,440 patients (Huang et al., 2016; Pillay et al., 2015 and Ramadas et al., 2011 did not given the total sample size)		HbA1c level		Some evidence
		Terranova et al., 2015 [72]	Weight		
		Pillay et al., 2015 [71]	HbA1c levels		
			BMI		
		Ramadas et al., 2011 [77]	HbA1c	46.2% studies (+)	
		Gary et al., 2003 [49]	Fast blood sugar		
			Glychemoglobin		
			HbA1		
			HbA1c		
			Weight		
Self-management educational interventions	Nine/19,597 patients (Minet et al., 2010;Fan et al., 2009 and Norris et al., 2001 did not given the total sample size)	Pal et al., 2014 [67]	Cardiovascular Risk Factors		
			Cognitive Outcomes		
			Behavioral Outcomes	Only one study reporting a significant improvement.	
			AEs	No AEs	
		Vugt et al., 2013 [75]	Health behaviors	Seven of 13 studies (+)	
			Clinical outcomes measures	Nine studies (+)	
			Psychological outcomes	Nine studies (+)	
		Pal et al., 2013 [68]	HbA1c		
			Depression		
			Quality of life		
			Weight		
		Steinsbekk, et al., 2012 [76]	HbA1c		
			Main lifestyle outcomes		
			Main psychosocial outcomes		
		Minet et al., 2010 [61]	Glycaemic control		
			Diabetes knowledge		
			Overall self-management		

For peer review only

			behaviours		
		Fan et al., 2009 [47]	Overall metabolic outcomes		
			Overall weighted mean effect sizes		Sufficient evidence
		Deakin et al., 2005 [43]	Metabolic control (HbA1c)		
			Fasting blood glucose levels		
			Weight		
			Diabetes knowledge		
			SBP		
			Diabetes medication		
		Norris et al.,, 2002 [65]	Total GHb		
		Norris et al., 2001 [64]	Knowledge		
			Self-monitoring of blood glucose		
			Self-reported dietary habits		
			Glycemic control		
Therapeutic education	One/Total sample: Unclear.	Odnoletkova et al., 2014 [66]	Cost-effectiveness	Overall high in studies on prediabetes and varied in studies on T2DM.	Insufficient evidence
Foot health education	One/ Total sample: Unclear.	Amaeshi, 2012 [32]	Diabetes complications		Some evidence
			Incidence of LEA		
Group medical visit	One/2,240 patients.	Burke et al., 2011 [38]	HbA1c		
			BP and DBP		
			SBP		
			Cholesterol - LDL		Some evidence
Psychoeducational intervention.	One/1.431 patients.	Alam et al., 2009 [30]	HbA1c		Some evidence
			Psychological status		
Interventions aimed at improving adherence to	Three/ 4,907 patients (Kai Lun Gan et al., 2011 did not	Kai Lun Gan et al., 2011 [57]	Oral hypoglycaemic adherence	Five of seven studies (+)	

For peer review only - http://bmjopen.bmj.com/site/about/guidelines.xhtml

medical treatment recommendations	given the total sample size)	Wens et al., 2008 [78]	Adherence	General conclusions could not be drawn.	Some evidence
		Vermeire et al., 2005 [80]	HbA1c		
Dietary advice	One/1,467 patients	Nield et al., 2007 [63]	Glycaemic control (addition of exercise to dietary advice)		Insufficient evidence to determine
			Weight	Limited Data	
			Diabetic micro- and macrovascular diseases	Limited Data	

NOTE:

RCTs =Randomized controlled trials; HRQoL = Health Related Quality of Life; MI = Myocardial Infarction; CABG= Coronary Artery Bypass Graft Surgery; T2DM = Type 2 Diabetes Mellitus; BMI = Body Mass Index; HbA1c = Glycatedhemoglobin; BP = Blood Pressure; SBP = Systolic Blood Pressure, DBP = Diastolic Blood Pressure, HDL-c= High-density Lipoprotein Cholesterol; LDL-c = Low-density Lipoprotein Cholesterol; LEA = Lower Extremity Amputation; Adverse Events = AEs.

（+）: Intervention Group is Significantly Better than Control Group; For example: “91% studies (+)” means 91% studies reported a significant better compared with control group;

For peer review only

Table 5 Recommendations of health education programs for ACS and T2DM patients

		ACS patients	T2DM patients	Both ACS and T2DM
**Theoretical approach**		SCT, empowerment theories.	HBM; SCT.	HBM; SCT and empowerment theories.
**Behavioral strategies**		Goal setting	Goal setting	Goal setting
**Educational content**		Behavioral change (such as smoking cessation), cardiovascular risk factors, exercise, medication, and psychosocial issues.	Behavioral change, diet, exercise, glycemic control, medication, and self-management.	Behavioral change (such as smoking cessation), cardiovascular risk factors, diet, exercise, glycemic control, medication, psychosocial issues, and self-management.
**Healthcare professionals to deliver**		Nurse or multidisciplinary team	Multidisciplinary team; dietitian or nurse	Nurse or multidisciplinary team
**Teaching approaches**	Strategies	Face-to-face; telephone or mixed	Face to face, written materials; telephone or mixed	Face to face, written materials; telephone contact or mixed
	Format	Individual (One by one) or mixed	Individual (One by one) or mixed	Individual (One by one) or mixed
**Delivery timing**	Contact hours	More than 30 min per time per week	More than 30 min per time per week	More than 30 min per time per week
	Duration	At least 6 months	About 6 months	At least 6 months
**Duration of follow up**		At least 12 months	At least 12 months	At least 12 months
**Settings**		Inpatient and post-discharge settings	Hospital settings and primary care settings	Inpatient and post-discharge settings

Note: ACS = acute coronary syndrome; T2DM = type 2 diabetes mellitus; SCT=Social Cognitive Theory; HBM =Health Belief Model.

For peer review only

Fig. 1. Flow chart of the systematic reviews and meta-analyses selection process

For peer review only

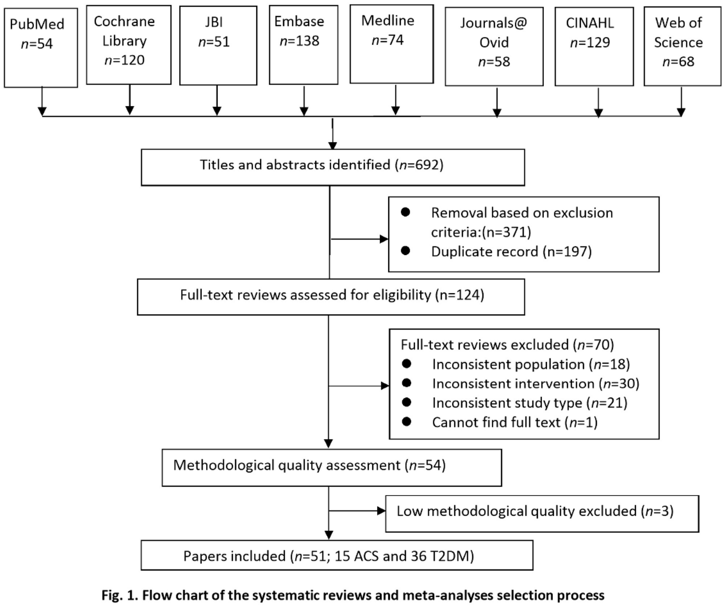

Fig. 1. Flow chart of the systematic reviews and meta-analyses selection process

215x279mm (300 x 300 DPI)

For peer review only - http://bmjopen.bmj.com/site/about/guidelines.xhtml

Supplementary Annex 1. Searching Strategies

Items	Searching Strategy	Results
	**PubMed**	
#1	(((((((((Acute Coronary Syndrome[MeSH Terms]) OR Myocardial Infarction[MeSH Terms]) OR Angina, Unstable[MeSH Terms]) OR Coronary Artery Disease[MeSH Terms]) OR Coronary Artery Bypass[MeSH Terms]) OR Angina Pectoris[MeSH Terms]) OR “Myocardial Infarct*”[Title/Abstract]) OR “Unstable Angina*”[Title/Abstract]) OR “coronary heart diseas*”[Title/Abstract]) OR “Coronary Artery Disease*”[Title/Abstract]) OR "Unstable Angina Pectori*"[Title/Abstract] Filters: Publication date from 2000/01/01 to 2016/04/31; English	145310
#2	(((((Diabetes Mellitus, Type 2[MeSH Terms]) OR "Type 2 Diabetes Mellitus"[Title/Abstract]) OR "type 2 diabete*"[Title/Abstract]) OR "type ii diabet*"[Title/Abstract]) OR T2DM[Title/Abstract]) OR Adult-Onset Diabetes Mellitus[Title/Abstract] Filters: Publication date from 2000/01/01 to 2016/04/31; English	80218
#3	#1 OR #2	222103
#4	(((((((Health Education[MeSH Terms]) OR Patient Education as Topic[MeSH Terms]) OR Counseling[MeSH Terms]) OR Social Support[MeSH Terms]) OR "Community Health Education*"[Title/Abstract]) OR "Educational Activit*"[Title/Abstract]) OR "Education* intervention*"[Title/Abstract]) OR "Education* program*"[Title/Abstract] Filters: Publication date from 2000/01/01 to 2016/04/31; English	122323
#5	(((Meta-Analysis [Publication Type]) OR Meta-Analysis as Topic[MeSH Terms]) OR "Systematic Revie*"[Title/Abstract]) OR "Meta-analys*"[Title/Abstract] Filters: Publication date from 2000/01/01 to 2016/04/31; English	165265
#6	#3 AND #4 AND #5 Filters: Publication date from 2000/01/01 to 2016/04/31; English	**54**
	**Cochrane Library**	
#1	MeSH descriptor: [Acute Coronary Syndrome] explode all trees	1070
#2	MeSH descriptor: [Myocardial Infarction] explode all trees	9450
#3	MeSH descriptor: [Angina, Unstable] explode all trees	1015
#4	MeSH descriptor: [Coronary Artery Disease] explode all trees	3469

For peer review only - http://bmjopen.bmj.com/site/about/guidelines.xhtml

#5	MeSH descriptor: [Coronary Artery Bypass] explode all trees	5321
#6	MeSH descriptor: [Angina Pectoris] explode all trees	4240
#7	"acute coronary syndrome":ti,ab,kw or Unstable Angina*:ti,ab,kw or Myocardial Infarct*:kw or coronary heart diseas*:ti,ab,kw or Coronary Artery Disease*:ti,ab,kw (Word variations have been searched)	29379
#8	#1 or #2 or #3 or #4 or #5 or #6 or #7	34062
#9	MeSH descriptor: [Diabetes Mellitus, Type 2] explode all trees	10474
#10	Type 2 Diabetes Mellitus:ti,ab,kw or type ii diabet*:ti,ab,kw or noninsulin dependent:ti,ab,kw or type 2 diabet*:ti,ab,kw or Adult-Onset Diabetes Mellitus:ti,ab,kw (Word variations have been searched)	20935
#11	#9 or #10	20937
#12	#8 or #11	53777
#13	MeSH descriptor: [Health Education] explode all trees	11291
#14	MeSH descriptor: [Patient Education as Topic] explode all trees	7507
#15	MeSH descriptor: [Counseling] explode all trees	4026
#16	MeSH descriptor: [Rehabilitation Nursing] explode all trees	55
#17	Education*:ti,ab,kw or Community Health Education:ti,ab,kw or Self-Management*:ti,ab,kw or Cardiac Rehabilitation:ti,ab,kw (Word variations have been searched)	40078
#18	#13 or #14 or #15 or #16 or #17	42943
#19	#12 and #18 Publication Year from 2000 to 2016, in Cochrane Reviews (Reviews only) and Other Reviews	120
#20	Cochrane reviews + other reviews	**49 + 71=120**
	**Joanna Briggs Institute,_Embase,_MEDLINE**	
#1	Acute Coronary Syndrome.m_titl.	16727
#2	Myocardial Infarction.m_titl.	151398

#3	Unstable Angina.m_titl.	6933
#4	Coronary Artery Disease.m_titl.	54621
#5	Coronary Artery Bypass.m_titl.	30449
#6	Angina Pectoris.m_titl.	17997
#7	Coronary Heart Disease.m_titl.	35397
#8	#1 or #2 or #3 or #4 or #5 or #6	305035
#9	Type 2 Diabetes Mellitus.mp. [mp=ti, ab, ot, nm, hw, kf, px, rx, ui, an, tx, sh, ct, sa, tn, dm, mf, dv, kw]	64926
#10	"type 2 diabet*".m_titl.	102596
#11	"type ii diabet*".m_titl.	6139
#12	Adult-Onset Diabetes Mellitus.mp. [mp=ti, ab, ot, nm, hw, kf, px, rx, ui, an, tx, sh, ct, sa, tn, dm, mf, dv, kw]	307
#13	#9 or #10 or #11 or #12	136982
#14	#8 or #13	439243
#15	Health Education.mp. [mp=ti, ab, ot, nm, hw, kf, px, rx, ui, an, tx, sh, ct, sa, tn, dm, mf, dv, kw]	177285
#16	Patient Education.mp. [mp=ti, ab, ot, nm, hw, kf, px, rx, ui, an, tx, sh, ct, sa, tn, dm, mf, dv, kw]	185631
#17	Counseling.m_titl.	23527
#18	Social Support.m_titl.	11535
#19	"Education*".m_titl.	247901
#20	#15 or #16 or #17 or #18 or #19	577203
#21	#14 and #20	6480
#22	Systematic Review.mp. [mp=ti, ab, ot, nm, hw, kf, px, rx, ui, an, tx, sh, ct, sa, tn, dm, mf, dv, kw]	212014
#23	Meta-Analysis.mp. [mp=ti, ab, ot, nm, hw, kf, px, rx, ui, an, tx, sh, ct, sa, tn, dm, mf, dv, kw]	262009
#24	#22 or #23	381113

For peer review only - http://bmjopen.bmj.com/site/about/guidelines.xhtml

#25	#21 and #24	288
#26	limit 25 to English and yr="2000 - 2016"	**263**
	**Cumulative Index to Nursing and Allied Health Literature (CINAHL)**	
#1	TI Acute Coronary Syndrome OR AB Acute Coronary Syndrome	4826
#2	TI Myocardial Infarction OR AB Myocardial Infarction	24578
#3	TI Unstable Angina OR AB Unstable Angina	1353
#4	TI Coronary Artery Disease OR AB Coronary Artery Disease	12930
#5	TI Coronary Heart Disease OR AB Coronary Heart Disease	12321
#6	#1 OR #2 OR #3 OR #4 OR #5	46436
#7	TI Type 2 Diabetes Mellitus OR AB Type 2 Diabetes Mellitus	8952
#8	TI type 2 diabet* OR AB type 2 diabet*	33085
#9	TI Adult-Onset Diabetes Mellitus OR AB Adult-Onset Diabetes Mellitus	23
#10	#7 OR #8 OR #9	33097
#11	#6 OR #10	77923
#12	TI Health Education OR AB Health Education	53415
#13	TI Patient Education OR AB Patient Education	37789
#14	TI Counseling OR AB Counseling	17673
#15	TI Social Support OR AB Social Support	30093
#16	TI Education* OR AB Education*	188291
#17	#12 OR #13 OR #14 OR #15 OR #16	227269
#18	#11 AND #17	4707
#19	TI Systematic Review OR AB Systematic Review	48830

For peer review only - http://bmjopen.bmj.com/site/about/guidelines.xhtml

#20	TI Meta-Analysis OR AB Meta-Analysis	28776
#21	#19 OR #20	64611
#22	S18 AND S21 Limiters - Published Date: 20000101-20160431; English Language	**129**
	**Web of Science**	
#1	TS=("Acute Coronary Syndrome*") OR TS=("Myocardial Infarct*") OR TS=("Unstable Angina") OR TS=("Coronary Artery Disease*") OR TS=("Coronary heart disease*")<br>Indexes=SCI-EXPANDED, SSCI, A&HCI, CPCI-S, CPCI-SSH, BKCI-S, BKCI-SSH, ESCI, CCR-EXPANDED, IC Timespan=All years	404699
#2	TS=("Type 2 Diabetes Mellitus") OR TS=("type 2 diabete*") OR TS=("Noninsulin-Dependent Diabete*") OR TS= ("Adult-Onset Diabetes Mellitus")<br>Indexes=SCI-EXPANDED, SSCI, A&HCI, CPCI-S, CPCI-SSH, BKCI-S, BKCI-SSH, ESCI, CCR-EXPANDED, IC Timespan=All years	103548
#3	#2 OR #1<br>Indexes=SCI-EXPANDED, SSCI, A&HCI, CPCI-S, CPCI-SSH, BKCI-S, BKCI-SSH, ESCI, CCR-EXPANDED, IC Timespan=All years	495081
#4	TS=("Health Education") OR TS=("Patient Education")<br>Indexes=SCI-EXPANDED, SSCI, A&HCI, CPCI-S, CPCI-SSH, BKCI-S, BKCI-SSH, ESCI, CCR-EXPANDED, IC Timespan=All years	29830
#5	#4 AND #3<br>Indexes=SCI-EXPANDED, SSCI, A&HCI, CPCI-S, CPCI-SSH, BKCI-S, BKCI-SSH, ESCI, CCR-EXPANDED, IC Timespan=All years	1290
#6	TS=("Systematic Review*") OR TS=("Meta-analysis*")<br>Indexes=SCI-EXPANDED, SSCI, A&HCI, CPCI-S, CPCI-SSH, BKCI-S, BKCI-SSH, ESCI, CCR-EXPANDED, IC Timespan=All years	149663
#7	(#6 AND #5) AND LANGUAGE: (English)<br>Indexes=SCI-EXPANDED, SSCI, A&HCI, CPCI-S, CPCI-SSH, BKCI-S, BKCI-SSH, ESCI, CCR-EXPANDED, IC Timespan=2000-2016	**68**
	**Journals@Ovid**	
#1	Acute Coronary Syndrome.m_titl.	3021
#2	Myocardial Infarction.m_titl.	24269
#3	Unstable Angina.m_titl.	1038
#4	Coronary Artery Disease.m_titl.	10106
#5	Coronary Artery Bypass.m_titl.	6546
#6	Angina Pectoris.m_titl.	1480

For peer review only - http://bmjopen.bmj.com/site/about/guidelines.xhtml

#7	Coronary Heart Disease.m_titl.	6487
#8	#1 or #2 or #3 or #4 or #5 or #6	51548
#9	Type 2 Diabetes Mellitus. m_titl.	5097
#10	"type 2 diabet*".m_titl.	26434
#11	"type ii diabet*".m_titl.	980
#12	Adult-Onset Diabetes Mellitus. m_titl.	391
#13	#9 or #10 or #11 or #12	27795
#14	#8 or #13	78792
#15	Health Education. m_titl.	1135
#16	Patient Education. m_titl.	1556
#17	Counseling.m_titl.	5532
#18	Social Support.m_titl.	2667
#19	"Education*".m_titl.	50950
#20	#15 or #16 or #17 or #18 or #19	58969
#21	#14 and #20	510
#22	Systematic Review.mp. [mp=ti, ab, ot, nm, hw, kf, px, rx, ui, an, tx, sh, ct, sa, tn, dm, mf, dv, kw]	117790
#23	Meta-Analysis.mp. [mp=ti, ab, ot, nm, hw, kf, px, rx, ui, an, tx, sh, ct, sa, tn, dm, mf, dv, kw]	215069
#24	#22 or #23	282984
#25	#21 and #24	61
#26	limit 26 to yr="2000 - 2016"	**58**

For peer review only - http://bmjopen.bmj.com/site/about/guidelines.xhtml

Supplementary Annex 2.

Citation Matrix: Non-repeated trials (rows) included in the 15 systematic review and/or meta-analyses (column)--ACS.

Author-year (Trial reference)	Barth, Jacob, Daha, & Critchley, 2015	Devi et al., 2015	Ghisi, Abdallah, Grace, Thomas, & Oh, 2014	Kotb, Hsieh, & Wells, 2014	Brown, Clark, Dalal, Welch, & Taylor, 2013	Dickens et al., 2013	Aldcroft, Taylor, Blackstock, & O'Halloran P, 2011	Brown, Clark, Dalal, Welch, & Taylor, 2011	Huttunen-Lenz, Song, & Poland, 2010	Goulding, Furze, & Birks, 2010	Auer, Gaume, Rodondi, Cornuz, & Ghali, 2008	J. Barth, Critchley, & Bengel, 2008	Fernandez et al., 2007	J. Barth, Critchley, & Bengel, 2006	Clark, Hartling, Vandermeer, & McAlister, 2005
Allen JK. Coronary risk factor modification in women after coronary artery bypass surgery. Nursing Research 1996;45(5):260-5.	X											X			
Burt A, Thornley R, Illingworth D, White P, Shaw TR, Turner R. Stopping smoking after myocardial infarction. Lancet 1974;1(7852):304-6.	X											X		X	
Carlsson R, Lindberg G, Westin L, Israelsson B. Influence of coronary nursing managment follow up on lifestyle after acute myocardial infarction. Heart 1997;77(3):256-9.	X											X			
Ockene J, Kristeller JL, Goldberg R, Ockene I, Merriam P, Barrett S, et al. Smoking cessation and severity of disease: the Coronary Artery Smoking Intervention Study. Health Psychology 1992;11(2):119-26.	X											X		X	
DeBusk R, Miller NH, Superko R, Dennis CA, Thomas RJ, et al. A case-management system for coronary risk factor modification after acute myocardial infarction. Annals of Internal Medicine 1994;120(9):721-9.	X										X	X			X
Dornelas EA, Sampson RA, Gray J F, Waters D, Thompson PD. A randomized controlled trial of smoking cessation counseling after myocardial infarction. Preventive Medicine 2000;30(4):261-8.	X								X			X	X	X	
Hajek P, Taylor TZ, Mills P. Brief intervention during hospital admission to help patients to give up smoking after myocardial infarction and bypass surgery: randomised controlled trial. BMJ 2002;324:87-9.	X								X			X	X	X	
Heller RF, Knapp JC, Valenti LA, Dobson AJ. Secondary prevention after acute myocardial infarction. American Journal of Cardiology 1993;72(11):759-62.	X											X	X	X	
Moreno Ortigosa A, Ochoa Gomez FJ, Ramalle-Gomara E, Saralegui Reta I, Fernandez Esteban MV, Quintana Diaz M. Efficacy of an smoking cessationg intervention for patients with myocardial infarction [Eficacia de una intervencion para dejar de fumar en pacientes con infarto de miocardio]. Medicina Clinica 2000;114:209-10.	X											X		X	
Quist-Paulsen P, Gallefoss F. Randomised controlled trial of smoking cessation intervention after admission for coronary heart disease. BMJ 2003;327(7426):1254-7.	X								X			X		X	
Reid R, Pipe A, Higginson LK, D'Angelo MS, Cooke D, Dafoe W. Stepped care approach to smoking cessation in patients hospitalized for coronary artery disease. Journal	X											X		X	

For peer review only

of Cardiopulmonary Rehabilitation 2003;23(3):176-82.															
Rigotti NA, McKool KM, Shiffmann S. Predictors of smoking cessation after coronary artery bypass graft surgery. Annals of Internal Medicine 1994;120(4):287-93.	X								X			X		X	
Sivarajan ES, Newton KM, Almes MJ, Kempf TM, Mansfield LW, Bruce RA. Limited effects of outpatient teaching and counseling after myocardial infarction: a controlled study. Heart & Lung 1983;12(1):65-73.	X													X	
Taylor CB, Houston-Miller N, Killen JD, DeBusk RF. Smoking cessation after acute myocardial infarction: effects of a nurse-managed intervention. Annals of Internal Medicine 1990;113(2):118-23.	X								X				X	X	
Van Elderen T, Maes S, Seegers G. Effects of a group health education program for coronary heart patients after the hospitalization phase. Tijdschrift voor Psychologie & Gezondheid 1991;19(3):129-44.	X											X			
van Elderen-van Kemenade T, Maes S, van den Broek Y. Effects of a health education programme with telephone follow-up during cardiac rehabilitation. British Journal of Clinical Psychology 1994;33(Pt 3):367-78.	X		X	X							X	X		X	
Antypas K, Wangberg SC. An Internet- and mobile-based tailored intervention to enhance maintenance of physical activity after cardiac rehabilitation: short-term results of a randomized controlled trial. Journal of Medical Internet Research 2014;16(3):e77.		X													
Devi R, Powell J, Singh S. A web-based program improves physical activity outcomes in a primary care angina population: randomized controlled trial. Journal of Medical Internet Research 2014;16(9):e186.		X													
Frederix I, Van Driessche N, Hansen D, Berger J, Bonne K, Alders T, et al. Increasing the medium-term clinical benefits of hospital-based cardiac rehabilitation by physical activity telemonitoring in coronary artery disease patients. European Journal of Preventive Cardiology 2015;22(2):150-8.		X													
Lear SA, Singer J, Banner-Lukaris D, Horvat D, Park JE, Bates J, et al. Randomized trial of a virtual cardiac rehabilitation program delivered at a distance via the internet. Circulation: Cardiovascular Quality and Outcomes 2014;7(6):952-9.		X													
Lindsay S, Bellaby P, Smith S, Baker R. Enabling healthy choices: is ICT the highway to health improvement?. Health (London, England: 1997) 2008;12(3):313-31.		X													
Maddison R, Pfaeffli L, Whittaker R, Stewart R, Kerr A, Jiang Y, et al. A mobile phone intervention increases physical activity in people with cardiovascular disease: Results from the HEART randomized controlled trial. European Journal of Preventive Cardiology 2014 May 9 [Epub ahead of print].		X													
Reid RD, Morrin LI, Beaton LJ, Papadakis S, Kocourek J, McDonnell L, et al. Randomized trial of an internet-based computer-tailored expert system for physical activity in patients with heart disease. European Journal of Preventive Cardiology 2012;19(6):1357-64.		X													

For peer review only

Southard BH, Southard DR, Nuckolls J. Clinical trial of an Internet-based case management system for secondary prevention of heart disease. Journal of Cardiopulmonary Rehabilitation and Prevention 2003;23(5):341-8.		X			X	X		X					X		
Varnfield M, Karunanithi M, Lee CK, Honeyman E, Arnold D, Ding H, et al. Smartphone-based home care model improved use of cardiac rehabilitation in postmyocardial infarction patients: results from a randomised controlled trial. Heart 2014;100(22):1770-9.		X													
Vernooij JW, Kaasjager HA, van der Graaf Y, Wierdsma J, Grandjean HM, Hovens MM, et al. Internet based vascular risk factor management for patients with clinically manifest vascular disease: randomised controlled trial. BMJ (Clinical research ed.) 2012;344:e3750.		X													
Zutz A, Ignaszewski A, Bates J, Lear SA. Utilization of the Internet to deliver cardiac rehabilitation at a distance: a pilot study. Telemedicine Journal and e-Health 2007;13(3):323-30.		X													
Dale L, Whittaker R, Jiang Y, Stewart R, Rolleston A, Maddison R. Improving coronary heart disease self-management using mobile technologies (Text4Heart): a randomised controlled trial protocol. Trials 2014;15:71.		X													
ISRCTN29243064. Effectiveness of a comprehensive telerehabilitation program for the heart. http://www.controlled-trials.com/ISRCTN29243064 (accessed January 2015).		X													
NCT02228603. How to enhance physical activity after cardiac rehabilitation? A randomised controlled study comparing two follow-up training exercise programs. https://clinicaltrials.gov/show/NCT02228603 (accessed January 2015).		X													
NCT02350192. Effectiveness of an e-health educational intervention for cardiovascular disease adults in improving total exercise and outcomes. https://clinicaltrials.gov/ct2/show/NCT02350192 (accessed January 2015).		X													
Redfern J, Usherwood T, Harris MF, Rodgers A, Hayman N, Panaretto K, et al. A randomised controlled trial of a consumer-focused e-health strategy for cardiovascular risk management in primary care: the Consumer Navigation of Electronic Cardiovascular Tools (CONNECT) study protocol. BMJ Open 2014; Vol. 4, issue 2. [DOI: 10.1136/bmjopen-2013-004523]		X													
Reinwand D, Kuhlmann T, Wienert J, Vries HD, Lippke S. Designing a theory- and evidence-based tailored eHealth rehabilitation aftercare program in Germany and the Netherlands: study protocol. BMC Public Health 2013;13:1081.		X													
Shah B, Adams M, Peterson E, Powers B, Oddone E, Royal K, et al. Secondary Prevention Risk Interventions Via Telemedicine and Tailored Patient Education (SPRITE): A randomized trial to improve postmyocardial infarction management. Circulation: Cardiovascular Quality and Outcomes 2011;4(2):235-42.		X													

For peer review only

T. Buckley, S. McKinley, R. Gallagher, K. Dracup, D.K. Moser, L.M. Aitken<br>The effect of education and counselling on knowledge, attitudes and beliefs about responses to acute myocardial infarction symptoms<br>Eur J Cardiovasc Nurs, 6 (2007), pp. 105–111			x							x					
J.J. Carlson, G.J. Norman, D.L. Feltz, B.A. Franklin, J.A. Johnson, S.K. Locke<br>Self-efficacy, psychosocial factors, and exercise behavior in traditional versus modified cardiac rehabilitation<br>J Cardpulm Rehabil, 21 (2001), pp. 363–373			x												
S.S. Chan, D.Y. Leung, D.C. Wong, C.P. Lau, V.T. Wong, T.H. Lam<br>A randomized controlled trial of stage-matched intervention for smoking cessation in cardiac-outpatients<br>Addiction, 107 (2012), pp. 829–837			x												
K.M. Cordasco, S.M. Asch, D.S. Bell, J.J. Guterman, S. Gross-Schulman, L. Ramer, et al.<br>A low-literacy medication education tool for safety-net hospital patients<br>Am J Prev Med, 37 (2009), pp. S209–S216			x												
M.H. Eckman, R. Wise, A.C. Leonard, E. Dixon, C. Burrows, F. Khan, et al.<br>Impact of health literacy on outcomes and effectiveness of an educational intervention in patients with chronic diseases<br>Patient Educ Couns, 87 (2012), pp. 143–151			x												
P. Giannuzzi, P.L. Temporelli, R. Marchioli, A.P. Maggioni, G. Balestroni, V. Ceci, et al.<br>Global secondary prevention strategies to limit event recurrence after myocardial infarction: results of the GOSPEL study, a multicenter, randomized controlled trial from the Italian Cardiac Rehabilitation Network<br>Arch Intern Med, 168 (2008), pp. 2194–2204			x												
K.B. Heidal, N.M. Lewis, S.M. Evans, L. Boeckner<br>Nutrition education intervention increases total n − 3 fatty acid intakes in heart patients living in the Midwest<br>Nutr Res, 27 (2007), pp. 33–37			x												
L. Horlick, R. Cameron, W. Firor, U. Bhalerao, R. Baltzan<br>The effects of education and group discussion in the post myocardial infarction patient<br>J Psychosom Res, 28 (1984), pp. 485–492			x												
H.S. Jeong, J.S. Chae, J.S. Moon, Y.S. Yoo<br>An individualized teaching program for atherosclerotic risk factor reduction in patients with myocardial infarction<br>Yonsei Med J, 43 (2002), pp. 93–100			x												
X. Jiang, J.W. Sit, T.K. Wong<br>A nurse-led cardiac rehabilitation programme improves health behaviours and cardiac physiological risk			x												

For peer review only

parameters: evidence from Chengdu, China<br>J Clin Nurs, 16 (2007), pp. 1886–1897															
M. Kummel, T. Vahlberg, A. Ojanlatva, R. Karki, T. Mattila, S.L. Kivela<br>Effects of an intervention on health behaviors of older coronary artery bypass (CAB) patients<br>Arch Gerontol Geriatr, 46 (2008), pp. 227–244			X												
W.S. Leslie, C.R. Hankey, D. Matthews, J.E.P. Currall, M.E.J. Lean<br>A transferable programme of nutritional counselling for rehabilitation following myocardial infarction: a randomised controlled study<br>Eur J Clin Nutr, 58 (2004), pp. 778–786			X												
E. Lidell, B. Fridlund<br>Long-term effects of a comprehensive rehabilitation programme after myocardial infarction<br>Scand J Caring Sci, 10 (1996), pp. 67–74			X							X					
S. Lindsay, S. Smith, P. Bellaby, R. Baker<br>The health impact of an online heart disease support group: a comparison of moderated versus unmoderated support<br>Health Educ Res, 24 (2009), pp. 646–654			X												
R.A. Mayou, D.R. Thompson, A. Clements, C.H. Davies, S.J. Goodwin, K. Normington, et al.<br>Guideline-based early rehabilitation after myocardial infarction: a pragmatic randomised controlled trial<br>J Psychosom Res, 52 (2002), pp. 89–95			X								X				
S. McKinley, K. Dracup, D.K. Moser, B. Riegel, L.V. Doering, H. Meischke, et al.<br>The effect of a short one-on-one nursing intervention on knowledge, attitudes and beliefs related to response to acute coronary syndrome in people with coronary heart disease: a randomized controlled trial<br>Int J Nurs Stud, 46 (2009), pp. 1037–1046			X												
S.M. Moore, M.A. Dolansky<br>Randomized trial of a home recovery intervention following coronary artery bypass surgery<br>Res Nurs Health, 24 (2001), pp. 93–104			X			X									
B. Oldenburg, A. Martin, J. Greenwood, L. Bernstein, R. Allan<br>A controlled trial of a behavioral and educational intervention following coronary artery bypass surgery<br>J Cardpulm Rehabil, 15 (1995), pp. 39–46			X												
J.E. Otterstad<br>Influence on lifestyle measures and five-year coronary risk by a comprehensive lifestyle intervention programme in patients with coronary heart disease<br>Eur J Cardiovasc Prev Rehabil, 10 (2003), pp. 429–437			X												X
P.M. Smith, E. Burgess			X						X						

For peer review only

Smoking cessation initiated during hospital stay for patients with coronary artery disease: a randomized controlled trial<br>CMAJ, 180 (2009), pp. 1297–1303															
T.M. Van Elderen, S. Maes, G. Seegers, H. Kragten, L. Relik-Can Wely<br>Effects of a post-hospitalization group health education programme for patients with coronary heart disease<br>Psychol Health, 9 (1994), pp. 317–330			X											X	
J. Wolkanin-Bartnik, H. Pogorzelska, A. Bartnik<br>Patient education and quality of home-based rehabilitation in patients older than 60 years after acute myocardial infarction<br>J Cardiopulm Rehabil Prev, 31 (2011), pp. 249–253			X												
C. Froger-Bompas, B. Laviolle, P. Guillo, C. Letellier, K. Ligier, J.C. Daubert, et al.<br>Sustained positive impact of a coronary rehabilitation programme on adherence to dietary recommendations<br>Arch Cardiovasc Dis, 102 (2009), pp. 97–104			X												
B.J. Linde, N.M. Janz<br>Effect of a teaching program on knowledge and compliance of cardiac patients<br>Nurs Res, 28 (1979), pp. 282–286			X												
J. Oliveira, F. Ribeiro, H. Gomes<br>Effects of a home-based cardiac rehabilitation program on the physical activity levels of patients with coronary artery disease<br>J Cardiopulm Rehabil Prev, 28 (2008), pp. 392–396			X												
R. Song, H. Lee<br>Managing health habits for myocardial infarction (MI) patients<br>Int J Nurs Stud, 38 (2001), pp. 375–380			X												
B.L. Verges, B. Patois-Verges, M. Cohen, J.M. Casillas<br>Comprehensive cardiac rehabilitation improves the control of dyslipidemia in secondary prevention<br>J Cardpulm Rehabil, 18 (1998), pp. 408–415			X												
T. Yoshida, M. Kohzuki, K. Yoshida, M. Hiwatari, M. Kamimoto, C. Yamamoto, et al.<br>Physical and psychological improvements after phase II cardiac rehabilitation in patients with myocardial infarction<br>Nurs Health Sci, 1 (1999), pp. 163–170			X												
G.F. Fletcher<br>Rehabilitation after coronary angioplasty—is it effective<br>Arch Phys Med Rehabil, 67 (1986), pp. 517–519			X												
C.J. Frame, C.G. Green, D.G. Herr, M.L. Taylor<br>A 2-year stage of change evaluation of dietary fat and fruit and vegetable intake behaviors of cardiac rehabilitation patients			X												

For peer review only

Am J Health Promot, 17 (2003), pp. 361–368															
S. Pelletier, S. Kundrat, C.M. Hasler<br>Effects of a functional foods nutrition education program with cardiac rehabilitation patients<br>J Cardpulm Rehabil, 23 (2003), pp. 334–340			x												
L. Jensen<br>Self-administered cardiac medication program evaluation<br>Can J Cardiovasc Nurs, 13 (2003), pp. 35–44			x												
J. Lisspers, C. Hofman-Bang, R. Nordlander, L. Ryden, O. Sundin, A. Ohman, et al.<br>Multifactorial evaluation of a program for lifestyle behavior change in rehabilitation and secondary prevention of coronary artery disease<br>Scand Cardiovasc J, 33 (1999), pp. 9–16			x												
A. Palomaki, S. Miilunpalo, P. Holm, E. Makinen, K. Malminiem<br>Effects of preventive group education on the resistance of LDL against oxidation and risk factors for coronary heart disease in bypass surgery patients<br>Ann Med, 34 (2002), pp. 272–283			x												
M.T. Timlin, K.V. Shores, M. Reicks<br>Behavior change outcomes in an outpatient cardiac rehabilitation program<br>J Am Diet Assoc, 102 (2002), pp. 664–671			x												
C. Bellman, K. Hambraeus, J. Lindbäck, B. Lindahl<br>Achievement of secondary preventive goals after acute myocardial infarction: a comparison between participants and nonparticipants in a routine patient education program in Sweden<br>J Cardiovasc Nurs, 24 (2009), pp. 362–368			x												
K. Dracup, A.I. Meleis, S. Clark, A. Clyburn, L. Shields, M. Staley<br>Group counseling in cardiac rehabilitation: effect on patient compliance<br>Patient Educ Couns, 6 (1984), pp. 169–177			x				x								
Z. Irmak, H. Fesci<br>Effects of nurse-managed secondary prevention program on lifestyle and risk factors of patients who had experienced myocardial infarction<br>Appl Nurs Res, 23 (2010), pp. 147–152			x												
J. Marshall, S. Penckofer, J. Llewellyn<br>Structured postoperative teaching and knowledge and compliance of patients who had coronary artery bypass surgery<br>Heart Lung, 15 (1986), pp. 76–82			x												
T. Van Elderen, E. Dusseldorp<br>Lifestyle effects of group health education for patients with coronary heart disease			x												

For peer review only

Psychol Health, 16 (2001), pp. 327–341															
P. Davidson, M. Digiacomo, R. Zecchin, M. Clarke, G. Paul, K. Lamb, et al. A cardiac rehabilitation program to improve psychosocial outcomes of women with heart disease J Womens Health, 17 (2008), pp. 123–134			X												
Arthur HM, Smith KM, Kodis J, McKelvie R (2002) A controlled trial of hospital versus home-based exercise in cardiac patients. Medicine and Science in Sports and Exercise. 34(10): 15441550				X											
Bambauer KZ, Aupont O, Stone PH, Locke SE, Mullan MG, et al. (2005) "The effect of a telephone counseling intervention on self-rated health of cardiac patients.". Psychosomatic Medicine 67(4): 539–545.				X											
Bazargani RH, Besharat MA, Ehsan HB, Nejatian M, Hosseini K (2011) The efficacy of Chronic Disease Self Management Programs and Tele-health on psychosocial adjustment by increasing self-efficacy in patients with CABG. Procedia - Social and Behavioral Sciences 30: 817–821.				X											
Beckie T (1989) A supportive-educative telephone program: Impact on knowledge and anxiety after coronary artery bypass graft surgery. Heart and Lung: Journal of Critical Care 18(1): 46–55.				X											
Gallagher R, McKinley S, Dracup K (2003) Effects of a telephone counseling intervention on psychosocial adjustment in women following a cardiac event. Heart and Lung: Journal of Acute and Critical Care 32(2): 79–87.				X		X									
Hanssen TA, Nordrehaug JE, Eide GE, Rokne B (2007) Improving outcomes after myocardial infarction: A randomized controlled trial evaluating effects of a telephone follow-up intervention. European Journal of Cardiovascular Prevention and Rehabilitation 14 (3): 429–437.				X				X							
Hanssen TA, Nordrehaug JE, Eide GE, Hanestad BR (2009) Does a telephone follow-up intervention for patients discharged with acute myocardial infarction have long-term effects on health-related quality of life? A randomised controlled trial. Journal of Clinical Nursing 18(9): 1334–1345.				X											
Hartford K, Wong C, Zakaria D (2002) Randomized controlled trial of a telephone intervention by nurses to provide information and support to patients and their partners after elective coronary artery bypass graft surgery: Effects of anxiety. Heart and Lung: Journal of Acute and Critical Care, 31(3), 199–206.				X											
Holmes-Rovner M, Stommel M, Corser WD, Olomu A, Holtrop JS, et al. (2008) Does outpatient telephone coaching add to hospital quality improvement following				X		X									

For peer review only

hospitalization for acute coronary syndrome? Journal of General Internal Medicine 23(9): 1464–70.															
Ma Y, Ockene IS, Rosal MC, Merriam PA, Ockene JK, et al. (2010) Randomized trial of a pharmacist-delivered intervention for improving lipid-lowering medication adherence among patients with coronary heart disease. Cholesterol 2010: 1–11.				x											
McLaughlin TJ, Aupont O, Bambauer K, Stone P, Mullan MG, et al. (2005) Improving psychologic adjustment to chronic illness in cardiac patients: The role of depression and anxiety. Journal of General Internal Medicine 20(12): 1084–1090.				x											
Mittag O, China C, Hoberg E, Juers E, Kolenda K, et al. (2006) Outcomes of cardiac rehabilitation with versus without a follow-up intervention rendered by telephone (Luebeck follow-up trial): overall and gender-specific effects. International journal of rehabilitation research Internationale Zeitschrift fur Rehabilitationsforschung. Revue internationale de recherches de readaptation 29(4): 295–302.				x		x									
Neubeck L, Redfern J, Briffa T, Ascanio R, Freedman SB, et al. (2009) Cardiovascular risk benefits of the CHOICE (Choice of Health Options In prevention of Cardiovascular Events) program are maintained for four years: Randomised controlled trial. European Heart Journal Conference: European Society of Cardiology, ESC Congress 2009 Barcelona Spain. Conference Start: 20090829 Conference End: 20090902. Conference Publication: (var.pagings) 30: 76.				x											
Neubeck L, Freedman SB, Briffa T, Bauman A, Redfern J (2011) Four-year follow-up of the choice of health options in prevention of cardiovascular events randomized controlled trial. European Journal of Cardiovascular Prevention and Rehabilitation.18(2)278286				x											
Redfern J, Briffa T, Ellis E, Freedman SB (2008) Patient-centered modular secondary prevention following acute coronary syndrome: A randomized controlled trial. Journal of Cardiopulmonary Rehabilitation and Prevention.28(2)107117				x											
Redfern J, Briffa T, Ellis E, Freedman SB (2009) Choice of secondary prevention improves risk factors after acute coronary syndrome: 1-Year follow-up of the CHOICE (Choice of Health Options in prevention of Cardiovascular Events) randomised controlled trial. Heart 95 (6): 468–475.				x											
Redfern J, Menzies M, Briffa T, Freedman SB (2010) Impact of medical consultation frequency on modifiable risk factors and medications at 12 months after acute coronary syndrome in the CHOICE randomised controlled trial. International Journal of Cardiology 145 (3): 481–486.				x											
Reid RD, Pipe AL, Quinlan B, Oda J (2007) Interactive				x					x						

For peer review only - http://bmjopen.bmj.com/site/about/guidelines.xhtml

voice response telephony to promote smoking cessation in patients with heart disease: A pilot study. Patient Education and Counseling 66 (3): 319–326.															
Smith KM, Arthur HM, McKelvie RS, Kodis J (2004) Differences in sustainability of exercise and health-related quality of life outcomes following home or hospital-based cardiac rehabilitation. European Journal of Cardiovascular Prevention and Rehabilitation 11(4): 313–319.				X											
Smith KM (2007) Sustainability of exercise capacity and quality of life after home or hospital based exercise training in low-risk patients following coronary artery bypass graft surgery: a six-year follow-up of a randomized controlled trial, McMaster University (Canada): 176 p–176 p.				X											
Smith KM, McKelvie RS, Thorpe KE, Arthur HM (2011) Six-year follow-up of a randomised controlled trial examining hospital versus home-based exercise training after coronary artery bypass graft surgery. Heart 97(14): 1169–1174.				X											
Stevens B (1985) The effectiveness of patient education follow-up by telephone on knowledge of post-myocardial infarction patients. (M.Nus., University of Alberta (Canada)). ProQuest Dissertations and Theses (303461144).				X											
Tranmer JE, Parry MJE (2004) Enhancing postoperative recovery of cardiac surgery patients - A randomized clinical trial of an advanced practice nursing intervention. Western Journal of Nursing Research 26(5): 515–532.				X											
Vale MJ, Jelinek MV, Best JD, Dart AM, Grigg LE, et al. (2003) Coaching patients on achieving cardiovascular health (COACH): A multicenter randomized trial in patients with coronary heart disease. Archives of Internal Medicine–. 163(22)27752783				X		X							X		X
Vale MJ, Jelinek MV, Best JD, Santamaria JD (2002) Coaching patients with coronary heart disease to achieve the target cholesterol: A method to bridge the gap between evidence-based medicine and the "real world" - randomized controlled trial. Journal of Clinical Epidemiology. 55(3)245252				X											X
Clark NM, Janz NK, Becker MH, . Impact of self-management education on the functional health status of older adults with heart disease. The Gerontologist 1992; 32: 438–443.					X			X							
Clark NM, Janz NK, Dodge JA, . Self-Management of heart disease by older adults. Res Aging 1997; 19: 362–382.					X										
Clark NM, Janz NK, Dodge JA, . Changes in functional health status of older women with heart disease: evaluation of a program based on self-regulation. J					X			X							

For peer review only

Gerontol B Psychol Sci Soc Sci 2000; 55B: S117–S126.															
Wheeler JR. Can a disease self-management programme reduce health care costs: the case of older women with heart disease. Med Care 2003; 41: 706–715					X										
Clark NM, Janz NK, Dodge JA, . Heart disease management by women: does intervention format matter? Health Educ Behav 2009; 36: 394–409					X			X							
Cupples ME, McKnight A, O'Neill C, . The effect of personal health education on the quality of life of patients with angina in general practice. Health Educ J 1996; 55: 75–83					X										
Cupples ME, McKnight A. Five year follow up of patients at high cardiovascular risk who took part in randomised controlled trial of health promotion. Brit Med J 1999; 319: 687–688.					X			X							X
Cupples ME, McKnight A. Randomised controlled trial of health promotion in general practice for patients at high cardiovascular risk. Brit Med J 1994; 309: 993–996					X										X
O'Neill C, Normand C, Cupples M, . Cost effectiveness of personal health education in primary care for people with angina in the greater Belfast area of Northern Ireland. J Epidemiol Community Health 1996; 50: 538–540.					X										
Esposito D, Brown R, Chen A, . Impacts of a disease management program for dually eligible beneficiaries. Health Care Finan Rev 2008; 30: 27–45.					X			X							
Hanssen TA, Nordrehaug JE, Eide BE, . Improving outcomes after myocardial infarction: a randomized controlled trial evaluating effects of a telephone follow-up intervention. Eur J Cardiovasc Prevent Rehab 2007; 14(3): 429–437.					X										
Hanssen TA, Nordrehaug JE, Eide GE, . Can telephone follow-up after discharge improve life style factors after a myocardial infarction? A randomized controlled trial. 7th Annual Cardiovascular Nursing Spring Meeting of the European Society of Cardiology Council on Cardiovascular Nursing and Allied Professions: changing practice to improve care Manchester, UK 23–24 March 2007. Eur J Cardiovasc Nurs 2007; 6: S43–S44.					X										
Hanssen TA, Nordrehaug JE, Eide GE, . Does a telephone follow-up intervention for patients discharges with acute myocardial infarction have long-term effects on health-related quality of life? A randomised controlled trial. J Clin Nurs 2009; 18: 1334–1345					X										
Hanssen TA, Nordrehaug JE, Eide GE, . Evaluating the effect of a combined reactive and proactive telephone follow-up intervention after acute myocardial infarction. A randomized controlled trial. Eur J Cardiovasc Nurs 2006;					X										

5: S46–S46.															
Lie I, Arnesen H, Sandvik L, . Health-related quality of life after coronary artery bypass grafting. The impact of a randomised controlled home-based intervention program. Qual Life Res 2009; 18: 201–207.					x			x							
Hofman-Bang C, Lisspers J, Nordlander R, . Two-year results of a controlled study of residential rehabilitation for patients treated with percutaneous transluminal coronary angioplasty. A randomized study of a multifactorial programme. Eur Heart J 1999; 20: 1465–1474.					x	x		x							
Lisspers J, Sundin O, Hofman-Bang C, . Behavioural effects of a comprehensive, mutifactorial program for lifestyle change after percutaneous transluminal coronary angioplasty: a prospective, randomized, controlled study. J Psychosom Res 1999; 46: 143–154.					x										
Lisspers J, Sundin O, Ohman A, . Long-term effects of lifestyle behavior change in coronary artery disease: effects on recurrent coronary events after percutaneous coronary intervention. Health Psychol 2005; 24: 41–48.					x										
P.RE.COR. Group. Comparison of a rehabilitation programme, a counselling programme and usual care after an acute myocardial infarction: results of a long-term randomized trial. Eur Heart J 1991; 12: 612–616.					x			x							x
Brown R, Peikes D, Chen A, . 15-Site randomized trial of coordinated care in medicare FFS. Health Care Finan Rev 2008; 30: 5–25					x			x							
Peikes D, Chen A, Schore J, . Effects of care coordination on hospitalization, quality of care, and health care expenditures among medicare beneficiaries 15 randomized trials. J Amer Med Assoc 2009; 301: 603–618.					x										
Pogosova GV, Kalinina AM, Spivak EI, . Efficacy of an educational preventive technology in patients with stable angina in ambulatory conditions. Kardiologiia 2008; 48(7): 4–9.					x			x							
Tingstrom PR, Kamwendo K, Bergdahl B. Effects of a problem-based learning rehabilitation programme on quality of life in patients with coronary artery disease. Eur J Cardiovasc Nurs 2005; 4: 324–330					x			x							
Appels A, B„r F, van der PG, et al. Effects of treating exhaustion in angioplasty patients on new coronary events: results of the randomized Exhaustion Intervention Trial (EXIT). Psychosomatic Medicine. 2005;67(2):217-223.						x									
Ashton RC, Whitworth GC, Seldomridge JA, et al. The effects of self-hypnosis on quality of life following coronary artery bypass surgery: preliminary results of a prospective, randomized trial. Journal of Alternative and						x									

For peer review only

Complementary Medicine 1995;1(3):285-290.															
Barr Taylor CB, Houston-Miller N, Ahn DK, Haskell W, DeBusk RF. The effects of exercise training programs on psychosocial improvement in uncomplicated postmyocardial infarction patients. Journal of Psychosomatic Research. 1986;30(5):581-587.						X									
Berkman LF, Blumenthal J, Burg M, et al. Effects of treating depression and low perceived social support on clinical events after myocardial infarction: the Enhancing Recovery in Coronary Heart Disease Patients (ENRICHD) Randomized Trial. JAMA : the journal of the American Medical Association. 2003;289(23):3106-3116.						X									
Blumenthal JA, Sherwood A, Babyak MA, et al. Effects of exercise and stress management training on markers of cardiovascular risk in patients with ischemic heart disease: a randomized controlled trial. JAMA : the journal of the American Medical Association. 2005;293(13):1626-1634.						X									
Brown MA, Munford AM, Munford PR. Behavior therapy of psychological distress in patients after myocardial infarction or coronary bypass. Journal of Cardiopulmonary Rehabilitation. 1993;13(3):201-210.						X									
Burgess AW, Lerner DJ, D'Agostino RB, Vokonas PS, Hartman CR, Gaccione P. A randomized control trial of cardiac rehabilitation. Social Science & Medicine. 1987;24(4):359-370.						X									
Claesson M, Birgander LS, Lindahl B, et al. Women's hearts-stress management for women with ischemic heart disease: explanatory analyses of a randomized controlled trial. Journal of Cardiopulmonary Rehabilitation. 2005;25(2):93-102.						X									
de Klerk JE, du Plessis WF, Steyn HS, Botha M. Hypnotherapeutic ego strengthening with male South African coronary artery bypass patients. The American Journal of Clinical Hypnosis. 2004;47(2):79-92.						X									
Dugmore LD, Tipson RJ, Phillips MH, et al. Changes in cardiorespiratory fitness, psychological wellbeing, quality of life, and vocational status following a 12 month cardiac exercise rehabilitation programme. Heart. 1999;81(4):359-366						X									X
Engblom E, Korpilahti K, H„m„l„inen H, R”nnemaa T, Puukka P. Quality of life and return to work 5 years after coronary artery bypass surgery. Long-term results of cardiac rehabilitation. Journal of Cardiopulmonary Rehabilitation. 1997;17(1):29-36.						X									X
Frasure-Smith N, Lesperance F, Prince RH, et al. Randomised trial of home-based psychosocial nursing intervention for patients recovering from myocardial infarction. Lancet. 1997;350(9076):473-479.						X									X
Freedland KE, Skala JA, Carney RM, et al. Treatment of depression after coronary artery bypass surgery: A randomized controlled trial. Archives of General Psychiatry. 2009;66(4):387-396.						X									

For peer review only

Fridlund B, H"gstedt B, Lidell E, Larsson PA. Recovery after myocardial infarction. Effects of a caring rehabilitation programme. Scandinavian Journal of Caring Sciences. 1991;5(1):23-32.						X									X
Frizelle DJ, Lewin RJ, Kaye G, et al. Cognitive-behavioural rehabilitation programme for patients with an implanted cardioverter defibrillator: a pilot study. British Journal of Health Psychology. 2004;9(3):381-392.						X									
Furze G, Dumville JC, Miles JNV, Irvine K, Thompson DR, Lewin RJP. "Prehabilitation" prior to CABG surgery improves physical functioning and depression. International Journal of Cardiology. 2009;132:51-58.						X									
Goodman H, Parsons A, Davison J, et al. A randomised controlled trial to evaluate a nurse-led programme of support and lifestyle management for patients awaiting cardiac surgery 'Fit for surgery: Fit for life' study. European Journal of Cardiovascular Nursing. 2008;7(3):189-195.						X									
Hughes AR, Mutrie N, Macintyre PD. Effect of an exercise consultation on maintenance of physical activity after completion of phase III exercise-based cardiac rehabilitation. European Journal of Cardiovascular Prevention and Rehabilitation 2007;14(1):114-121.						X									
Johnston M, Foulkes J, Johnston DW, Pollard B, Gudmundsdottir H. Impact on patients and partners of inpatient and extended cardiac counseling and rehabilitation: a controlled trial. Psychosomatic Medicine. 1999;61(2):225-233.						X					X				X
Jones DA, West RR. Psychological rehabilitation after myocardial infarction: multicentre randomised controlled trial. BMJ (Clinical research ed.). 1996;313(7071):1517-1521.						X									X
Karlsson MR, Edstr"m-Ploss C, Held C, Henriksson P, Billing E, Wall,n NH. Effects of expanded cardiac rehabilitation on psychosocial status in coronary artery disease with focus on type D characteristics. Journal of Behavioral Medicine. 2007;30(3):253-261.						X									
Koertge J, Janszky I, Sundin O, et al. Effects of a stress management program on vital exhaustion and depression in women with coronary heart disease: a randomized controlled intervention study. Journal of Internal Medicine. 2008;263(3):281-293.						X									
Leserman J, Stuart EM, Mamish ME, Benson H. The efficacy of the relaxation response in preparing for cardiac surgery. Behavioral Medicine. 1989;15(3):111-117.						X									
Lesperance F, Frasure-Smith N, Koszycki D, et al. Effects of citalopram and interpersonal psychotherapy on depression in patients with coronary artery disease: the Canadian Cardiac Randomized Evaluation of Antidepressant and Psychotherapy Efficacy (CREATE) trial. JAMA : the journal of the American Medical Association. 2007;297(4):367-379.						X									
Lewin B, Robertson IH, Cay EL, Irving JB, Campbell M.						X									

For peer review only

Effects of self-help post-myocardial-infarction rehabilitation on psychological adjustment and use of health services. Lancet. 1992;339(8800):1036-1040.															
Lewin RJ, Coulton S, Frizelle DJ, Kaye G, Cox H. A brief cognitive behavioural preimplantation and rehabilitation programme for patients receiving an implantable cardioverter-defibrillator improves physical health and reduces psychological morbidity and unplanned readmissions. Heart. 2009;95:63-69.						X									
Lewin RJ, Furze G, Robinson J, et al. A randomised controlled trial of a self-management plan for patients with newly diagnosed angina. The British Journal of General Practice 2002;52(476):194-201.						X				X					
Lewin RJ, Thompson DR, Elton RA. Trial of the effects of an advice and relaxation tape given within the first 24 h of admission to hospital with acute myocardial infarction. International Journal of Cardiology. 2002;82(2):107-114.						X					X		X		
Lie I, Arnesen H, Sandvik L, Hamilton G, Bunch EH. Effects of a home-based intervention program on anxiety and depression 6 months after coronary artery bypass grafting: a randomized controlled trial. Journal of Psychosomatic Research. 2007;62(4):411-418.						X									
Linden B. Evaluation of a home-based rehabilitation programme for patients recovering from acute myocardial infarction. Intensive & Critical Care Nursing 1995;11(1):10-19.						X					X				
McHugh F, Lindsay GM, Hanlon P, et al. Nurse led shared care for patients on the waiting list for coronary artery bypass surgery: a randomised controlled trial. Heart 2001;86(3):317-323.						X									X
McLaughlin TJ, Aupont O, Bambauer KZ, et al. Improving psychologic adjustment to chronic illness in cardiac patients. The role of depression and anxiety. Journal of General Internal Medicine 2005;20(12):1084-1090.						X									
Michalsen A, Grossman P, Lehmann N, et al. Psychological and quality-of-life outcomes from a comprehensive stress reduction and lifestyle program in patients with coronary artery disease: results of a randomized trial. Psychotherapy and Psychosomatics. 2005;74(6):344-352.						X									
Murchie P, Campbell NC, Ritchie LD, Deans HG, Thain J. Effects of secondary prevention clinics on health status in patients with coronary heart disease: 4 year follow-up of a randomized trial in primary care. Family Practice. 2004;21(5):567-574.						X									X
Newton M, Mutrie N, McArthur JD. The effects of exercise in a coronary rehabilitation programme. Scottish Medical Journal. 1991;36(2):38-41.						X									
Oldridge N, Guyatt G, Jones N, et al. Effects on quality of life with comprehensive rehabilitation after acute myocardial infarction. The American Journal of Cardiology. 1991;67(13):1084-1089.						X									X
Paul-Labrador M, Polk D, Dwyer JH, et al. Effects of a randomized controlled trial of transcendental meditation						X									

For peer review only

on components of the metabolic syndrome in subjects with coronary heart disease. Archives of Internal Medicine. 2006;166(11):1218-1224.															
Postlethwaite R, Stirling G, Peck CL. Stress inoculation for acute pain: a clinical trial. Journal of Behavioral Medicine. 1986;9(2):219-227.						X									
Scholz U, Knoll N, Sniehotta FF, Schwarzer R. Physical activity and depressive symptoms in cardiac rehabilitation: long-term effects of a self-management intervention. Social Science & Medicine 2006;62(12):3109-3120.						X									
Schrader G, Cheok F, Hordacre AL, Marker J, Wade V. Effect of psychiatry liaison with general practitioners on depression severity in recently hospitalised cardiac patients: a randomised controlled trial. The Medical Journal of Australia. 2005;182(6):272-276.						X									
Sebregts EH, Falger PR, Appels A, Kester AD, B„r FW. Psychological effects of a short behavior modification program in patients with acute myocardial infarction or coronary artery bypass grafting. A randomized controlled trial. Journal of Psychosomatic Research. 2005;58(5):417-424.						X									
Seki E, Watanabe Y, Sunayama S, et al. Effects of phase III cardiac rehabilitation programs on health-related quality of life in elderly patients with coronary artery disease: Juntendo Cardiac Rehabilitation Program (J-CARP). Circulation Journal 2003;67(1):73-77.						X									X
Shuldham CM, Fleming S, Goodman H. The impact of pre-operative education on recovery following coronary artery bypass surgery. A randomized controlled clinical trial. European Heart Journal. 2002;23(8):666-674.						X									
Sneed NV, Finch NJ, Michel Y. The effect of psychosocial nursing intervention on the mood state of patients with implantable cardioverter defibrillators and their caregivers. Progress In Cardiovascular Nursing. 1997;12(2):4-14.						X									
Sorlie T, Busund R, Sexton J, Sexton H, Sorlie D. Video information combined with individualized information sessions: Effects upon emotional well-being following coronary artery bypass surgery-A randomized trial. Patient Education & Counseling. 2007;65(2):180-188.						X									
Stern MJ, Gorman PA, Kaslow L. The group counseling v exercise therapy study. A controlled intervention with subjects following myocardial infarction. Archives of Internal Medicine. 1983;143(9):1719-1725.						X									X
Thompson DR, Meddis R. A prospective evaluation of in hospital counselling for first time myocardial infarction men. Journal of Psychosomatic Research. 1990;34(3):237-248.						X									
Trzcieniecka-Green A, Steptoe A. The effects of stress management on the quality of life of patients following acute myocardial infarction or coronary bypass surgery. European Heart Journal. 1996;17(11):1663-1670.						X									
Unden AL, Schenck-Gustafsson K, Axelsson PO, Karlsson I, Orth-Gom,r K, Ydrefors AM. Positive effects of						X									

For peer review only

increased nurse support for male patients after acute myocardial infarction. Quality of Life Research 1993;2(2):121-127.															
Barth J. Depression as a Risk Factor for Mortality in Patients With Coronary Heart Disease: A Meta-analysis. 2004.						X									
Merswolken M. Treating anxiety in patients with coronary heart disease: a randomized controlled trial. 2011.						X									
utwalli HA. Randomized controlled evaluation shows the effectiveness of a home-based cardiac rehabilitation program. 2012.						X									
Oranta O. The outcomes of interpersonal counselling on depressive symptoms and distress after myocardial infarction. 2010.						X									
Poortaghi S. General health improves with home-based cardiac rehabilitation program. 2011.						X									
Utriyaprasit K. Recovery after coronary artery bypass surgery: effect of an audiotape information programme. 2010.						X									
Izawa KP, Watanabe S, Omiya K, et al.. Effect of the self-monitoring approach on exercise maintenance during cardiac rehabilitation: a randomized controlled trial. Am J Phys Med Rehabil. 2005;84:313–321.a							X								
Yates BC, Anderson T, Hertzog M, Ott C, Williams J. Effectiveness of follow-up booster sessions in improving physical status after cardiac rehabilitation: health, behavioral and clinical outcomes. App Nurs Res. 2005;18:59–62							X								
Hofman-Bang C, Lisspers J, Nordiander R, et al.. Two-year results of a controlled study of residential rehabilitation for patients treated with percutaneous transluminal coronary angioplasty: a randomized study of a multifactorial program. Eur Heart J. 1999;20:1465–1474.							X								
Hsin-Hsin L, Yun-Fang T, Pyng-Jing L, Pei-Kwei T. Effects of a therapeutic lifestyle-change programme on cardiac risk factors after coronary artery bypass graft. J Clin Nurs. 2010;19:60–69.							X								
Lewin B, Robertson IH, Cay EL, Irving JB, Campbell M. Effects of self-help post myocardial infarction rehabilitation on psychological adjustment and use of health services. Lancet. 1992;339:1036–1040.							X								
Lewin RJP, Furze G, Robinson J, Griffith K, Wiseman S, Boyle R. A randomized controlled trial of a self-management plan for patients with newly diagnosed angina. Br J Gen Pract. 2001;52:194–201.							X								
Bolman, C. Vries, de, H., Breukelen, van, G. Evaluation of nurse managed minimal contact smoking cessation intervention for cardiac patients Health Education Research 17 99–116 2002b.									X						

For peer review only

Burt, A., Thornley, P., Illingworth, D., White, P., Shaw, T. R., Turner, R. Stopping smoking after myocardial infarction Lancet 1 304–306 1974.									X						
Feeney, G., McPherson, A., Connor, J., McAlister, A., Young, M., Garrahy, P. Randomized controlled trial of two cigarette quit programmes in coronary care patients after acute myocardial infarction Internal Medicine Journal 31 470–475 2001.									X				X	X	
Johnson, J. L., Budz, B., Mackay, M., Miller, M. Evaluation of a nurse-delivered smoking cessation intervention for hospitalized patients with cardiac disease Heart and Lung 28 55–64 1999.									X						
Mohiuddin, S. M., Mooss, A. N., Hunter, C. B., Grollmes, T. L., Cloutier, D. A., Hilleman, D. E. Intensive smoking cessation intervention reduces mortality in high-risk smokers with cardiovascular disease Chest 131 446–452 2007.									X						
Ockene, J., Kristeller, J. L., Goldberg, R., Ockene, I., Merriam, P., Barrett, S. Smoking cessation and severity of disease: The coronary artery smoking cessation intervention study Health Psychology 11 119–126 1992.									X						
Reid, R., Pipe, A., Higginson, L., Johnson, K., D'Angelo, M. S., Cooke, D., Dafoe, W. Stepped care approach to smoking cessation in patients hospitalized for coronary artery disease Journal of Cardiopulmonary Rehabilitation 23 176–182 2003.									X				X		
Barnason S. & Zimmerman L. (1995) A comparison of patient teaching outcomes among postoperative coronary artery bypass graft (CABG) patients. Progress in Cardiovascular Nursing 10(4), 11–20.										X					
Bengtsson K. (1983) Rehabilitation after myocardial infarction. A controlled study. Scandinavian Journal of Rehabilitation Medicine 15(1), 1–9.										X					X
Bolman C., Brug J., Bär F., Martinali J. & Van Den Borne B. (2005) Long-term efficacy of a checklist to improve patient education in cardiology. Patient Education and Counseling 56, 240–248.										X					
Cupples S.A. (1991) Effects of timing and reinforcement of preoperative education on knowledge and recovery of patients having coronary artery bypass graft surgery. Heart and Lung 20(6), 654–660.										X					
Furze G., Dumville J.C., Miles J.N.V., Irvine K., Thompson D.R. & Lewin R.J.P. (2009) “Prehabilitation” prior to CABG surgery improves physical functioning and depression. International Journal of Cardiology 132, 51–58.										X					
Martinali J., Bolman C., Brug J., Van Den Borne B. & Bar F. (2001) A checklist to improve patient education in a cardiology outpatient setting. Patient Education and										X					

For peer review only

Counseling 42, 231–238.															
Petrie K.J., Cameron L.D., Ellis C.J., Buick D. & Weinman J. (2002) Changing illness perceptions after myocardial infarction: an early intervention randomized controlled trial. Psychosomatic Medicine 64, 580–586.										X					
Pozen M.W., Stechmiller J.A., Harris W., Smith S., Fried D. & Voigt G.C. (1977) A nurse rehabilitator's impact on patients with myocardial infarction. Medical Care 15(10), 830–837.										X	X				
Tullman D.F., Haugh K.H., Dracup K.A. & Bourguignon C. (2007) A randomized controlled trial to reduce delay in older adults seeking help for symptoms of acute myocardial infarction. Research in Nursing & Health 30, 485–497.										X					
Eagle KA, Montoye CK, Riba AL, DeFranco AC, Parrish R, Skorcz S, Baker PL, Faul J, Jani SM, Chen B. Guideline-based standardized care is associated with substantially lower mortality in medicare patients with acute myocardial infarction: the American College of Cardiology's Guidelines Applied in Practice (GAP) projects in Michigan. J Am Coll Cardiol. 2005;46:1242–1248.											X				
Fonarow GC, Gawlinski A, Moughrabi S, Tillisch JH. Improved treatment of coronary heart disease by implementation of a Cardiac Hospitalization Atherosclerosis Management Program (CHAMP). Am J Cardiol. 2001;87:819–822.											X				
Oldenburg B, Perkins RJ, Andrews G. Controlled trial of psychological intervention in myocardial infarction. J Consult Clin Psychol. 1985;53:852–859											X				
Hedback B, Perk J. 5-year results of a comprehensive rehabilitation programme after myocardial infarction. Eur Heart J. 1987;8:234–242											X				
Maeland JG, Havik OE. The effects of an in-hospital educational programme for myocardial infarction patients. Scand J Rehabil Med. 1987;19:57–65.											X				
Fletcher V. An individualized teaching programme following primary uncomplicated myocardial infarction. J Adv Nurs. 1987;12:195–200											X				
Thompson DR. A randomized controlled trial of in-hospital nursing support for first time myocardial infarction patients and their partners: effects on anxiety and depression. J Adv Nurs. 1989;14:291–297.											X				
Fridlund B, Hogstedt B, Lidell E, Larsson PA. Recovery after myocardial infarction: effects of a caring rehabilitation programme. Scand J Caring Sci. 1991;5:23–32.											X				

For peer review only

Nordmann A, Heilmbauer I, Walker T, Martina B, Battegay E. A case-management program of medium intensity does not improve cardiovascular risk factor control in coronary artery disease patients: the Heartcare I Trial. Am J Med. 2001;110:543–550.											X				
Jeong HS, Chae JS, Moon JS, Yoo YS. An individualized teaching program for atherosclerotic risk factor reduction in patients with myocardial infarction. Yonsei Med J. 2002;43:93–100.											X				
Echeverry D, Dike M, Jovanovic L, Wollitzer AO, Westphal S, Mudaliar S, Washington C, Davidson MB. Efforts to improve subsequent treatment of cardiovascular risk factors in older patients with diabetes hospitalized for a cardiac event. Am J Manag Care. 2005;11:758–764.											X		X		
Scalzi CC, Burke LE, Greenland S. Evaluation of an inpatient educational program for coronary patients and families. Heart Lung. 1980;9:846–853.											X				
Kushnir B, Fox K, Tomlinson I, Aber CP. The effect of a pre-discharge consultation on the resumption of work, sexual activity, and driving following acute myocardial infarction. Scand J Rehabil Med. 1976;8:155–159.											X				
Young DT, Kottke TE, McCall MM, Blume D. A prospective controlled study of in-hospital myocardial infarction rehabilitation. J Card Rehabil. 1982;2:32–40.											X				
Holmboe ES, Meehan TP, Radford MJ, Wang Y, Marciniak TA, Krumholz HM. Use of critical pathways to improve the care of patients with acute myocardial infarction. Am J Med. 1999;107:324–331.											X				
Kucenic MJ, Meyers DG. Impact of a clinical pathway on the care and costs of myocardial infarction. Angiology. 2000;51:393–404.											X				
Cheah J. Clinical pathways: an evaluation of its impact on the quality of care in an acute care general hospital in Singapore. Singapore Med J. 2000;41:335–346.											X				
Hickey A, Scott I, Denaro C, Stewart N, Bennett C, Theile T. Using clinical indicators in a quality improvement programme targeting cardiac care. Int J Qual Health Care. 2004;16:i11–i25.											X				
Scott IA, Denaro CP, Hickey AC, Bennett C, Mudge AM, Sanders DC, Thiele J, Flores JL. Optimising care of acute coronary syndromes in three Australian hospitals. Int J Qual Health Care. 2004;16:275–284.											X				
Lappe JM, Muhlestein JB, Lappe DL, Badger RS, Bair TL, Brockman R, French TK, Hofmann LC, Horne BD, Kralick-Goldberg S, Nicponski N, Orton JA, Pearson RR, Renlund DG, Rimmasch H, Roberts C, Anderson JL. Improvements in 1-year cardiovascular clinical outcomes associated with a hospital-based discharge medication											X				

For peer review only

program. Ann Intern Med. 2004;141:446–453.															
Grace C, Summerbell C. Does provision of additional dietary information affect actual or only reported compliance to a low-fat diet over 12 weeks in hyperlipidaemic individuals? Report of a pilot study. J Hum Nutr Diet 1996; 9: 303–7.												X			
Dalgård C, Thurøe A, Haastrup B, Haghfelt T, Stender S. Saturated fat intake is reduced in patients with ischemic heart disease 1 year after comprehensive counseling but not after brief counseling. J Am Diet Assoc 2001; 101: 1420–9.												X			
Levetan CS, Dawn KR, Murray JF, Popma JJ, Ratner RE, Robbins DC. Impact of computer-generated personalized goals on cholesterol lowering. Value Health 2005; 8: 639–46.												X			
Bolman CVH, van Breukelen G. Evaluation of a nurse-managed minimal-contact smoking cessation intervention for cardiac inpatients. Health Educ Res 2002; 17: 99–116.												X			
Sivarajan Froelicher ESS, Miller NH, Christoperson DJ et al. High rates of sustained smoking cessation in women hospitalized with cardiovascular disease: the Women's Initiative for Nonsmoking(WINS). Circulation 2004; 109: 587–93												X			
Feder G, Griffiths C, Eldridge S, Spence M. Effect of postal prompts to patients and general practitioners on the quality of primary care after a coronary event (POST): randomised controlled trial. Br Med J 1999; 318: 1522.												X			
Mahler HI, Kulik JA, Tarazi RY. Effects of a videotape information intervention at discharge on diet and exercise compliance after coronary bypass surgery. J Cardiopulm Rehabil 1999; 19:170–7.												X			
Allen JK: Coronary risk factor modification in woman after coronary artery by pass surgery.Nursing Research. 1996,45:260–265.															X
Carlsson R, Lindberg G, Westin L, Israelsson B: Influence of coronary nursing management follow up on lifestyle after acute myocardial infarction.Heart. 1997,77:256–259.															X
DeBusk RF, Miller N, Superko HR, et al.: Acase-management system for coronary risk factor modification after acute myocardial infarction.Annals of Internal Medicine. 1994,120:721–729.															X
Lisspers J, Sundin O, Hofman-Bang C, et al.: Behavioral effects of a comprehensive, multifactorial program for lifestyle change after percutaneous transluminal coronary angioplasty: A prospective, randomized, controlled study.Journal of Psychosomatic Research. 1999,46:143–154.															X
Mitsibounas DN, Tsouna-Hadjis ED, Sideris DA, Rotas															X

For peer review only

AR: Effects of group psychsocial intervention on coronary risk factors.Psychotherapy and Psychosomatics. 1992,58:97–102.															
Campbell NC, Thain J, Deans HG, Ritchie LD, Rawles JM, Squair JL. Secondary prevention clinics for coronary heart disease: randomised trial of effect on health. BMJ. 1998;316:1434-7.															X
Campbell NC, Ritchie LD, Thain J, Deans HG, Rawles JM, Squair JL. Secondary prevention in coronary heart disease: a randomised trial of nurse led clinics in primary care. Heart. 1998;80:447-52															X
Engblom E, Rönnemaa T, Hämäläinen H, Kallio V, Vänttinen E, Knuts LR. Coronary heart disease risk factors before and after bypass surgery: results of a controlled trial on multifactorial rehabilitation. Eur Heart J. 1992;13:232-7.															X
Taylor CB, Miller NH, Smith PM, DeBusk RF. The effect of a home-based, case-managed, multifactorial risk-reduction program on reducing psychological distress in patients with cardiovascular disease. J Cardiopulm Rehabil. 1997;17:157-62.															X
Murchie P, Campbell NC, Ritchie LD, Simpson JA, Thain J. Secondary prevention clinics for coronary heart disease: four year follow up of a randomised controlled trial in primary care. BMJ. 2003;326:84															X
Schuler G, Hambrecht R, Schlierf G, Niebauer J, Hauer K, Neumann J, et al. Regular physical exercise and low-fat diet. Effects on progression of coronary artery disease. Circulation. 1992;86:1-11.a															X
Niebauer J, Hambrecht R, Velich T, Hauer K, Marburger C, Kälberer B, et al. Attenuated progression of coronary artery disease after 6 years of multifactorial risk intervention: role of physical exercise. Circulation. 1997;96:2534-41.															X
Ornish D, Brown SE, Scherwitz LW, Billings JH, Armstrong WT, Ports TA, et al. Can lifestyle changes reverse coronary heart disease? The Lifestyle Heart Trial. Lancet. 1990;336:129-33															X
Ornish D, Scherwitz LW, Billings JH, Brown SE, Gould KL, Merritt TA, et al. Intensive lifestyle changes for reversal of coronary heart disease. JAMA. 1998;280:2001-7.															X
Naylor MD, Brooten D, Campbell R, Jacobsen BS, Mezey MD, Pauly MV, et al. Comprehensive discharge planning and home follow-up of hospitalized elders: a randomized clinical trial. JAMA. 1999;281:613-20.															X
Naylor MD, McCauley KM. The effects of a discharge planning and home follow-up intervention on elders hospitalized with common medical and surgical cardiac conditions. J Cardiovasc Nurs. 1999;14:44-54.															X
Dorossiev D. Rehabilitation and Comprehensive Secondary Prevention after Acute Myocardial Infarction. Report on a Study. EURO Reports and Studies No. 84. Copenhagen: World Health Organization; 1983.															X

For peer review only

Kallio V, Hämäläinen H, Hakkila J, Luurila OJ. Reduction in sudden deaths by a multifactorial intervention programme after acute myocardial infarction. Lancet. 1979;2:1091-4															X
Hämäläinen H, Luurila OJ, Kallio V, Knuts LR, Arstila M, Hakkila J. Long-term reduction in sudden deaths after a multifactorial intervention programme in patients with myocardial infarction: 10-year results of a controlled investigation. Eur Heart J. 1989;10:55-62															X
Hämäläinen H, Luurila OJ, Kallio V, Knuts LR. Reduction in sudden deaths and coronary mortality in myocardial infarction patients after rehabilitation. 15 year follow-up study. Eur Heart J. 1995;16:1839-44.															X
Sivarajan ES, Bruce RA, Lindskog BD, Almes MJ, Belanger L, Green B. Treadmill test responses to an early exercise program after myocardial infarction: a randomized study. Circulation. 1982;65:1420-8.															X
Vermeulen A, Lie KI, Durrer D. Effects of cardiac rehabilitation after myocardial infarction: changes in coronary risk factors and long-term prognosis. Am Heart J. 1983;105:798-801															X
Bell JM. A Comparison of a Multi-Disciplinary Home Based Cardiac Rehabilitation Programme with Comprehensive Conventional Rehabilitation in Post-Myocardial Infarction Patients [PhD thesis]. London: University of London; 1998.															X
Toobert DJ, Glasgow RE, Radcliffe JL. Physiologic and related behavioral outcomes from the Women's Lifestyle Heart Trial. Ann Behav Med. 2000;22:1-9.															X
Sundin O, Lisspers J, Hofman-Bang C, Nygren A, Rydén L, Ohman A. Comparing multifactorial lifestyle interventions and stress management in coronary risk reduction. Int J Behav Med. 2003;10:191-204.															X
Yu CM, Li LS, Ho HH, Lau CP. Long-term changes in exercise capacity, quality of life, body anthropometry, and lipid profiles after a cardiac rehabilitation program in obese patients with coronary heart disease. Am J Cardiol. 2003;91:321-5.															X
Marchionni N, Fattirolli F, Fumagalli S, Oldridge N, Del Lungo F, Morosi L, et al. Improved exercise tolerance and quality of life with cardiac rehabilitation of older patients after myocardial infarction: results of a randomized, controlled trial. Circulation. 2003;107:2201-6.															X
Masley S, Phillips S, Copeland JR. Group office visits change dietary habits of patients with coronary artery disease—the dietary intervention and evaluation trial (D.I.E.T.). J Fam Pract. 2001;50:235-9.															X
Ornish D, Scherwitz LW, Doody RS, Kesten D, McLanahan SM, Brown SE, et al. Effects of stress management training and dietary changes in treating ischemic heart disease. JAMA. 1983;249:54-9.															X
Fitzgerald JF, Smith DM, Martin DK, Freedman JA, Katz															X

For peer review only

BP. A case manager intervention to reduce readmissions. Arch Intern Med. 1994;154:1721-9.															
Naylor M, Brooten D, Jones R, Lavizzo-Mourey R, Mezey M, Pauly M. Comprehensive discharge planning for the hospitalized elderly. A randomized clinical trial. Ann Intern Med. 1994;120:999-1006															X
Carlsson R. Serum cholesterol, lifestyle, working capacity and quality of life in patients with coronary artery disease. Experiences from a hospital-based secondary prevention programme. Scand Cardiovasc J Suppl. 1998;50:1-20															X
Jolly K, Bradley F, Sharp S, Smith H, Thompson S, Kinmonth AL, et al. Randomised controlled trial of follow up care in general practice of patients with myocardial infarction and angina: final results of the Southampton heart integrated care project (SHIP). The SHIP Collaborative Group. BMJ. 1999;318:706-11.															X
Allison TG, Farkouh ME, Smars PA, Evans RW, Squires RW, Gabriel SE, et al. Management of coronary risk factors by registered nurses versus usual care in patients with unstable angina pectoris (a chest pain evaluation in the emergency room [CHEER] substudy). Am J Cardiol. 2000;86:133-8															X
Moher M, Yudkin P, Wright L, Turner R, Fuller A, Schofield T, et al. Cluster randomised controlled trial to compare three methods of promoting secondary prevention of coronary heart disease in primary care. BMJ. 2001;322:1338.															X
Higgins HC, Hayes RL, McKenna KT. Rehabilitation outcomes following percutaneous coronary interventions (PCI). Patient Educ Couns. 2001;43:219-30.															X
Allen JK, Blumenthal RS, Margolis S, Young DR, Miller ER 3rd, Kelly K. Nurse case management of hypercholesterolemia in patients with coronary heart disease: results of a randomized clinical trial. Am Heart J. 2002;144:678-86															X
Lear SA, Ignaszewski A, Linden W, Brozic A, Kiess M, Spinelli JJ, et al. The Extensive Lifestyle Management Intervention (ELMI) following cardiac rehabilitation trial. Eur Heart J. 2003;24:1920-7.															X
Young W, Rewa G, Goodman SG, Jaglal SB, Cash L, Lefkowitz C, et al. Evaluation of a community-based inner-city disease management program for postmyocardial infarction patients: a randomized controlled trial. CMAJ. 2003;169:905-10.															X
Lichtman JH, Amatruda J, Yaari S, Cheng S, Smith GL, Mattera JA, et al. Clinical trial of an educational intervention to achieve recommended cholesterol levels in patients with coronary artery disease. Am Heart J. 2004;147:522-8.															X
Haskell WL, Alderman EL, Fair JM, Maron DJ, Mackey SF, Superko HR, et al. Effects of intensive multiple risk factor reduction on coronary atherosclerosis and clinical cardiac events in men and women with coronary artery disease. The Stanford Coronary Risk Intervention Project															X

For peer review only

(SCRIP). Circulation. 1994;89:975-90.															
Allison TG, Squires RW, Johnson BD, Gau GT. Achieving National Cholesterol Education Program goals for low-density lipoprotein cholesterol in cardiac patients: importance of diet, exercise, weight control, and drug therapy. Mayo Clin Proc. 1999;74:466-73															X
Stagmo M, Westin L, Carlsson R, Israelsson B. Long-term effects on cholesterol levels and the utilization of lipid-lowering drugs of a hospital-based programme for secondary prevention of coronary artery disease. J Cardiovasc Risk. 2001;8:243-8.															X
Wilhelmsen L, Sanne H, Elmfeldt D, Grimby G, Tibblin G, Wedel H. A controlled trial of physical training after myocardial infarction. Effects on risk factors, nonfatal reinfarction, and death. Prev Med. 1975;4:491-508.															X
Shaw LW. Effects of a prescribed supervised exercise program on mortality and cardiovascular morbidity in patients after myocardial infarction. The National Exercise and Heart Disease Project. Am J Cardiol. 1981;48:39-46															X
Carson P, Phillips R, Lloyd M, Tucker H, Neophytou M, Buch NJ, et al. Exercise after myocardial infarction: a controlled trial. J R Coll Physicians Lond. 1982;16:147-51.															X
Ballantyne FC, Clark RS, Simpson HS, Ballantyne D. The effect of moderate physical exercise on the plasma lipoprotein subfractions of male survivors of myocardial infarction. Circulation. 1982;65:913-8.															X
Miller NH, Haskell WL, Berra K, DeBusk RF. Home versus group exercise training for increasing functional capacity after myocardial infarction. Circulation. 1984;70:645-9.															X
Roviaro S, Holmes DS, Holmsten RD. Influence of a cardiac rehabilitation program on the cardiovascular, psychological, and social functioning of cardiac patients. J Behav Med. 1984;7:61-81.															X
Erdman RA, Duivenvoorden HJ, Verhage F, Kazemier M, Hugenholtz PG. Predictability of beneficial effects in cardiac rehabilitation: a randomized clinical trial of psychosocial variables. J Cardiopulm Rehabil. 1986;6:206-13.															X
Agren B, Olin C, Castenfors J, Nilsson-Ehle P. Improvements of the lipoprotein profile after coronary bypass surgery: additional effects of an exercise training program. Eur Heart J. 1989;10:451-8.															X
Bethell HJ, Mullee MA. A controlled trial of community based coronary rehabilitation. Br Heart J. 1990;64:370-5															X
Bertie J, King A, Reed N, Marshall AJ, Ricketts C. Benefits and weaknesses of a cardiac rehabilitation programme. J R Coll Physicians Lond. 1992;26:147-51.															X
Fletcher BJ, Dunbar SB, Felner JM, Jensen BE, Almon L, Cotsonis G, et al. Exercise testing and training in physically disabled men with clinical evidence of coronary artery disease. Am J Cardiol. 1994;73:170-4															X
Holmbäck AM, Säwe U, Fagher B. Training after myocardial infarction: lack of long-term effects on physical capacity and psychological variables. Arch Phys Med															X

For peer review only

Rehabil. 1994;75:551-4.															
Wosornu D, Bedford D, Ballantyne D. A comparison of the effects of strength and aerobic exercise training on exercise capacity and lipids after coronary artery bypass surgery. Eur Heart J. 1996;17:854-63.															x
Heldal M, Sire S, Dale J. Randomised training after myocardial infarction: short and long-term effects of exercise training after myocardial infarction in patients on beta-blocker treatment. A randomized, controlled study. Scand Cardiovasc J. 2000;34:59-64.															x
Ståhle A, Lindquist I, Mattsson E. Important factors for physical activity among elderly patients one year after an acute myocardial infarction. Scand J Rehabil Med. 2000;32:111-6.															x
Belardinelli R, Paolini I, Cianci G, Piva R, Georgiou D, Purcaro A. Exercise training intervention after coronary angioplasty: the ETICA trial. J Am Coll Cardiol. 2001;37:1891-900.															x
	16	20	42	26	24	62	7	13	14	13	27	18	17	19	74

ACS = acute coronary syndrome;

% overlap 63/286= 22.0%

CA= Covered Area = (16+20+42+26+24+62+7+13+14+13+27+18+17+19+74)/ (15x286) = 392/4290=9.1%

CCA= Corrected Covered Area = (392-286)/ (4290-286) = 106/4004= 2.6% (slight)

For peer review only

Supplementary Annex 3.

Citation Matrix: Non-repeated trials (rows) included in the 36 systematic review and/or meta-analyses (column)--T2DM.

Author-year (Trial reference)	1	2	3	4	5	6	7	8	9	10	11	12	13	14	15	16	17	18	19	20	21	22	23	24	25	26	27	28	29	30	31	32	33	34	35	36
K. Cai, S. Zhang, M. Lu, L. Shao The effects of intensive education for elderly patients with diabetes mellitus. Pract Geriatr, 18 (2004), pp. 313–315	X																																			
Y. Chen, W. Xue, S. Lin, S. Huang, D. Li. ntensive diabetes education in the role of diabetes control. J Sun Yat-sen Univ (Med Sci), 3S (2004), pp. 201–203	X																																			
L. Chen, M. Zhu. Clinical investigation of the effect of type 2 diabetes health education. Strait Pharm J, 21 (2009), pp. 217–218	X																																			
Y. Fang. Coordinated health education model in diabetes education. Today Nurse, 3 (2011), pp. 148–149	X																																			
B. Li, Y. Liu, J. Tian, J. Yuan. Impact of diabetes education for type 2 diabetes on glycated haemoglobin and health cost.Jiangsu J Prev Med, 23 (2012), pp. 55–56	X																																			
B.-h. Li, F.-h. Zhang, C. Zhang, M. Yang Effect of different education models on blood glucose in diabetic patients J Qilu Nurs, 19 (2013), pp. 26–27	X																																			
M. Li, T. Li, B.-y. Shi, C.-x. Gao Effect of motivational interviewing on the quality of life in type 2 diabetes patients with poor control of blood glucose Chin J Nurs, 49 (2014), pp. 6–10	X																																			
X. Li, Q. Zhou, F. Zou, L. Wu, H. Chen, Z. Liu Effectiveness of systematic self-management education on blood sugar level of patients in the community with type 2 diabetes J Central South Univ (Med Sci), 37 (2012), pp. 355–357	X																																			
Y. Li Evaluation of the standardized management effect of diabetes in community Chin Prim Health Care, 25 (2011), pp. 33–35	X																																			
Q. Liu, X. Pan, W. Feng, J. Kong Effectiveness of health education to the patients with diabetes mellitus J Nurs Sci, 20 (2005), pp. 54–55	X																																			
Z.-p. Liu, L.-l. Han, J.-r. Deng, X.-r. Liu, X.-r. Zhang, Q.-f. Li Effect of short-term intensified education on glucose	X																																			

For peer review only

control of type 2 diabetic patients<br>Chin J Nurs Educ, 4 (2007), pp. 249–251																																				
L.-m. Luo, K.-J. Yang, Z.-J. Zhang, Y.-H. Li, C.-H. Hu, X.-L. Feng<br>Role of health education on diabetic community chronic disease management<br>China Pharm, 24 (2013), pp. 3020–3022	X																																			
Y. Lu<br>Observation on the effect of diabetes intensive education in type 2 diabetes mellitus patients<br>Med Inform, 21 (2008), pp. 690–692	X																																			
X. Mei<br>Analysis of compliance and effectiveness of community diabetes education program<br>J Nanchang Univ (Med Sci), 50 (2010), pp. 83–84	X																																			
G. Pang<br>The impact of intensive diabetes education on community patients<br>J Med Theory Pract, 21 (2008), pp. 839–840	X																																			
Y. Qi, C. Chen, L. Yan<br>Impact of cooking class on diabetic patients eating habits<br>Chin J Nurs, 41 (2006), pp. 126–127	X																																			
B. Sun, B. Ban, H.-l. Sun, S. Zhang, S. Jie<br>Effects of diabetes education and psychological intervention on comprehensive treatment of type 2 diabetes<br>Chin J Clin Psychol, 13 (2005), pp. 483–485	X																						X													
M. Sun, X. Li, F. Wang, D. Zhu<br>Study on the effect of diabetes education<br>J Pract Diagn Ther, 19 (2005), pp. 422–423	X																																			
Z. Sun, M. Lv<br>90 cases of individualised type 2 diabetes education<br>J Qilu Nurs, 14 (2008), pp. 106–107	X																																			
H. Wang, S. Hu<br>Comparison of clinical applicability of individual intervention with intensive group education for diabetic patients<br>People's Mil Surg, 53 (2009), pp. 234–235	X																																			
X. Wang, H. Zou, H. Jie, S. Shen, J. Zhang<br>Effect of individualised diabetes education and care for aged patients with diabetes<br>Chin J Clin Res, 26 (2013), pp. 90–91	X																																			
Y. Wei<br>Effectiveness of intensive education for patients with diabetes mellitus<br>Chin J Health Educ, 21 (2005), pp. 53–55	X																																			
S.-q. Wu, G.-x. Jin, Y.-f. Chen	X																																			

For peer review only

Effect observation of sample diet education on diet control of patients with diabetes<br>J Nurs (China), 13 (2006), pp. 17–19																																				
Y. Wu, K. Wang, X. Yang<br>The effect of diabetes self-management education curriculum for patients with diabetes<br>Guide China Med, 11 (2013), pp. 395–397	X																																			
X.-L. Xiao, Q.-Q. Hu, Y.-P. Liu<br>Effects of family involvement in health education for patients with type 2 diabetes<br>Chin J Nurs, 47 (2012), pp. 313–316	X																																			
B. Xie, C.-p. Ju, Z.-l. Sun, J. Han, L. Liu<br>Effect of education network for diabetes self-management on self-efficacy in patients with type 2 diabetes mellitus<br>Chin Gen Pract, 15 (2012), pp. 3610–3616	X																																			
H. Xie, C. Cheng, D. Chen<br>Intensive diabetes education for poorly controlled elderly diabetic patients in the community<br>J Pract Med Tech, 14 (2007), pp. 3958–3959	X																																			
S. Xu<br>Effect of continued outpatient health education on diabetes patients<br>Mod Clin Nurs, 6 (2007), pp. 28–30	X																																			
L. Yan, C. Zhu, X. Tan<br>"Diabetes conversation" map interactive education for diabetic patients<br>Chin J Misdiagn, 11 (2011), pp. 6383–6384	X																																			
X. Yan, N. Li, H. Liu, H. Sha, P. Li<br>Effect of "interactive groups" diabetes education model on diabetes patients in the village<br>Nurs Pract Res, 9 (2012), pp. 13–14	X																																			
S. Yao, Y. Liu, R. Ji, G. Wang<br>Effectiveness of diabetes education for patients with type 2 diabetes<br>Hebei J Tradit Chin Med, 34 (2012), pp. 1723–1724	X																																			
W. Yu, Y.-l. Bi, J. Han, et al.<br>Affect of the MUST education mode on compliance of patients with type 2 diabetes mellitus<br>J Shandong Univ (Health Sci), 49 (2011), pp. 1–4	X																																			
Y. Zeng, A. Wang, M. Gong<br>Clinical observation of communication management targeted at patients with type 2 diabetes<br>J Pract Diabetol, 5 (2009), pp. 48–49	X																																			
F. Zhang, B. Xu<br>Influence of diabetes education on patients lifestyle and metabolic indicators	X																																			

For peer review only

Mod Med J China, 12 (2010), pp. 116–117																																				
J. Zhang, Y. Zhang, X. Huang<br>"Diabetes conversation" map interactive education for type 2 diabetes patients<br>Guide China Med, 9 (2011), pp. 330–331	X																																			
L. Zhang<br>Analysis on the therapeutic effects of diabetes education<br>Tianjin J Tradit Chin Med, 26 (2009), pp. 22–23	X																																			
X.-q. Zhang, L.-f. Fan, Y.-g. Zheng<br>Effect of health education applied in promoting compliance of diet treatment of patients with diabetes mellitus<br>Mod Nurs, 12 (2006), pp. 1082–1084	X																																			
X. Zheng, H. Tang, Y. Huang<br>Strengthen health education on observed efficacy in patients with type 2 diabetes<br>Youjiang Med J, 33 (2005), pp. 349–350	X																																			
C. Zhong, J. Li, X. Yang<br>Effect of interactive diabetes education for patients with diabetes<br>Jilin Med J, 27 (2006), pp. 1490–1491	X																																			
A. Zhou, Y. Ying, X. Dai, et al.<br>Application of text message to diabetic education<br>J Nurs Sci, 27 (2012), pp. 17–19	X																																			
M.-l. Gu, X.-g. Zhu, X.-f. Xu<br>The study of comprehensive intervention for patients with type 2 diabetes in community of Shanghai<br>Chin Gen Pract, 15 (2012), pp. 107–109	X																																			
Z.-M. Huang<br>Impact of intensive diabetes education targeted at patients with type 2 diabetes<br>Shanghai J Prev Med, 21 (2009), pp. 323–324	X																																			
C. Liu, W. Sun, J. Wang, J. Liu<br>Effect of community management of type 2 diabetic patients<br>Chin J Prev Control Chronic Dis, 20 (2012), pp. 624–625	X																																			
J. Sun, Y. Wang, X. Chen, et al.<br>An integrated intervention program to control diabetes in overweight Chinese women and men with type 2 diabetes<br>Asia Pac J Clin Nutr, 17 (2008), pp. 514–524	X																																			
X. Wang, X. Zhou, H. Zhen, J. Sun, C. Cui<br>Impact of stratified intensive education for patients with diabetes<br>J Med Theory Pract, 18 (2005), pp. 336–337	X																																			
X. Wang, X. Zhou, H. Zhen, J. Sun, C. Cui	X																																			

For peer review only

Impact of stratified intensive education for patients with diabetes<br>J Med Theory Pract, 18 (2005), pp. 336–337																																				
B. Yang, C. Ma, X. Feng, et al.<br>Effect of face-to-face education on metabolic improvement in Tibetan diabetic patients<br>J Clin Rehabil Tissue Eng Res, 11 (2007), pp. 10467–10470	X																																			
G.T. Ko, J.K. Li, E.C. Kan, M.K. Lo<br>Effects of a structured health education programme by a diabetic education nurse on cardiovascular risk factors in Chinese type 2 diabetic patients: a 1-year prospective randomized study<br>Diabet Med, 21 (2004), pp. 1274–1279	X		X	X																		X		X												
A. Lee, C.F. Siu, K.T. Leung, L.C. Lau, C.C. Chan, K.W. Kwok<br>General practice and social service partnership for better clinical outcomes, patient self efficacy and lifestyle behaviours of diabetic care: randomised control trial of a chronic care model Postgrad Med J, 87 (2011), pp. 688–693	X				X																															
S.M. Chen, D. Creedy, H.S. Lin, J. Wollin<br>Effects of motivational interviewing intervention on self-management, psychological and glycemic outcomes in type 2 diabetes: a randomized controlled trial<br>Int J Nurs Stud, 49 (2012), pp. 637–644	X																																			
M.C. Huang, C.C. Hsu, H.S. Wang, S.J. Shin<br>Prospective randomized controlled trial to evaluate effectiveness of registered dietitian-led diabetes management on glycemic and diet control in a primary care setting in Taiwan<br>Diabetes Care, 33 (2010), pp. 233–239	X																																			
M.C. Huang, C.C. Hsu, H.S. Wang, S.J. Shin<br>Prospective randomized controlled trial to evaluate effectiveness of registered dietitian-led diabetes management on glycemic and diet control in a primary care setting in Taiwan<br>Diabetes Care, 33 (2010), pp. 233–239	X																																			
M. Jian, L. Liu, H.-c. Chiang<br>Building a certificate-based secure and quick response telemedical health-care system for diabetic patients<br>J Intern Med Taiwan, 17 (2006), pp. 61–72	X																																			
T.I. Lee, Y.T. Yeh, C.T. Liu, P.L. Chen<br>Development and evaluation of a patient-oriented education system for diabetes management<br>Int J Med Inform, 76 (2007), pp. 655–663	X																			X																
Agurs-Collins TD, Kumanyika SK, Ten Have TR, Adams-Campbell LL. A randomized controlled trial of weight reduction and exercise for diabetes management in		X			X		X			X		X				X					X	X				X				X				X	X	X

For peer review only

older African-American subjects. Diabetes Care 1997; 20: 1503–1511.																																				
Anderson RM, Funnell MM, Nwankwo R, Gillard ML, Oh M, Fitzgerald JT. Evaluating a problem-based empowerment program for African Americans with diabetes: results of a randomized controlled trial. Ethn Dis 2005; 15: 671–678.		x			x		x			x						x					x	x				x										
Babamoto KS, Sey KA, Camilleri AJ, Karlan VJ, Catalasan J, Morisky DE. Improving diabetes care and health measures among hispanics using community health workers: results from a randomized controlled trial. Health Educ Behav 2009; 36: 113–126.		x					x			x																										
Baradaran HR, Knill-Jones RP, Wallia S, Rodgers A. A controlled trial of the effectiveness of a diabetes education programme in a multi-ethnic community in Glasgow [ISRCTN28317455]. BMC Public Health 2006; 18: 134.		x					x			x							x				x					x	x									
Bellary S, O'Hare JP, Raymond NT, Gumber A, Mughal S, Szczepura A et al. Enhanced diabetes care to patients of south Asian ethnic origin (the United Kingdom Asian Diabetes Study): a cluster randomised controlled trial. Lancet 2008; 371: 1769–1776.		x					x														x															
Brown SA, Garcia AA, Kouzekanani K, Hanis CL. Culturally competent diabetes self-management education for Mexican Americans: the Starr County border health initiative. Diabetes Care 2002; 25: 259–268.		x			x		x			x						x	x				x	x				x		x				x				
Carter EL, Nunlee-Bland G, Callender C. A patient-centric, provider-assisted diabetes telehealth self-management intervention for urban minorities. Perspect Health Inf Manag: Winter 2011.		x					x																													
Crowley MJ, Powers BJ, Olsen MK, Grubber JM, Koropchak C, Rose CM et al. The Cholesterol, Hypertension, And Glucose Education (CHANGE) study: results from a randomized controlled trial in African Americans with diabetes. Am Heart J 2013; 166: 179–186.		x					x																													
D'Eramo Melkus G, Chyun D, Vorderstrasse A, Newlin K, Jefferson V, Langerman S. The effect of a diabetes education, coping skills training, and care intervention on physiological and psychosocial outcomes in black women with type 2 diabetes. Biol Res Nurs 2010; 12:7–19.		x			x		x																													
DePue JD, Dunsiger S, Seiden AD, Blume J, Rosen RK, Goldstein MG. Nurse-community health worker team improves diabetes care in American Samoa: results of a randomized controlled trial. Diabetes Care 2013; 36: 1947–1953.		x					x																													
Gary TL, Batts-Turner M, Yeh HC, Hill-Briggs F, Bone LR, Wang NY et al. The effects of a nurse case manager		x					x																													

For peer review only

and a community health worker team on diabetic control, emergency department visits, and hospitalizations among urban African Americans with type 2 diabetes mellitus: a randomized controlled trial. Arch Intern Med 2009; 169: 1788–1794.																																				
Gucciardi E, Demelo M, Lee RN, Grace SL. Assessment of two culturally competent diabetes education methods: individual versus individual plus group education in Canadian Portuguese adults with type 2 diabetes. Ethn Health 2007; 12: 163–187.		X					X									X										X										
Hawthorne K, Tomlinson S. One-to-one teaching with pictures–flashcard health education for British Asians with diabetes. Br J Gen Pract 1997; 47: 301–304.		X					X			X						X					X			X		X	X							X		X
Kattelmann KK, Conti K, Ren C. The medicine wheel nutrition intervention: a diabetes education study with the Cheyenne River Sioux Tribe. J Am Diet Assoc 2009; 109: 1532–1539.		X					X																													
Keyserling TC, Samuel-Hodge CD, Ammerman AS, Ainsworth BE, Henríquez-Roldán CF, Elasy TA et al. A randomized trial of an intervention to improve self-care behaviors of African-American women with type 2 diabetes: impact on physical activity. Diabetes Care 2002; 25: 1576–1583.		X			X		X			X		X										X														
Khan MA, Shah S, Grudzien A, Onyejekwe N, Banskota P, Karim S et al. A diabetes education multimedia program in the waiting room setting. Diabetes Therapy 2011; 2: 178–188.		X					X																													
Kim MT, Han HR, Song HJ, Lee JE, Kim J, Ryu JP et al. A community-based, culturally tailored behavioral intervention for Korean Americans with type 2 diabetes. Diabetes Educator 2009; 35: 986–944.		X			X		X									X																				
Lorig K, Ritter PL, Villa F, Piette JD. Spanish diabetes self-management with and without automated telephone reinforcement: two randomized trials. Diabetes Care 2008; 31: 408–414.		X			X		X																													
Lujan J, Ostwald SK, Ortiz M. Promotora diabetes intervention for Mexican Americans. Diabetes Educator 2007; 33: 660–670.		X			X		X			X							X																			
Middelkoop BJ, Geelhoed-Duijvestijn PH, van der Wal G. Effectiveness of culture-specific diabetes care for Surinam South Asian patients in the Hague: a randomized controlled trial/controlled before-and-after study. Diabetes Care 2001; 24: 1997–1998		X					X			X						X					X					X	X									
O'Hare JP, Raymond NT, Mughal S, Dodd L, Hanif W, Ahmad Y et al. Evaluation of delivery of enhanced diabetes care to patients of South Asian ethnicity: the United Kingdom Asian Diabetes Study (UKADS).		X					X									X					X					X	X									

Diabetic Med 2004; 221: 1357–1365.																																				
Osborn CY, Amico KR, Cruz N, O'Connell AA, Perez-Escamilla R, Kalichman SC et al. A brief culturally tailored intervention for Puerto Ricans with type 2 diabetes. Health Educ Behav 2010; 37: 849–862.		X					X																													
Philis-Tsimikas A, Fortmann A, Lleva-Ocana L, Walker C, Gallo LC. Peer-led diabetes education programs in high-risk Mexican Americans improve glycemic control compared with standard approaches: a Project Dulce promotora randomized trial. Diabetes Care 2011; 34: 1926–1931		X			X		X			X																										
Rosal MC, Olendzki B, Reed GW, Gumieniak O, Scavron J, Ockene I. Diabetes self-management among low-income Spanish-speaking patients: a pilot study. Ann Behav Med 2005; 29: 225–235.		X			X		X			X						X	X				X	X				X										
Rothschild SK, Martin MA, Swider SM, Tumialán Lynas CM, Janssen I, Avery EF et al. Mexican American trial of community health workers: a randomized controlled trial of a community health worker intervention for Mexican Americans with type 2 diabetes mellitus. Am J Public Health 2014; 104: 1540–1548.		X			X		X																													
Samuel-Hodge CD, Keyserling TC, Park S, Johnston LF, Gizlice Z, Bangdiwala SI. A randomized trial of a church-based diabetes self-management program for African Americans with type 2 diabetes. Diabetes Educator 2009; 35: 439–453.		X			X		X																													
Sixta CS, Ostwald S. Texas-Mexico border intervention by promotores for patients with type 2 diabetes. Diabetes Educ 2008; 34: 299–309.		X			X		X																													
Skelly AH, Carlson JR, Leeman J, Holditch-Davis D, Soward AC. Symptom-focused management for African American women with Type 2 diabetes: a pilot study. Appl Nurs Res 2005; 18: 213–220.		X			X		X					X										X														
Skelly AH, Carlson J, Leeman J, Soward A, Burns D. Controlled trial of nursing interventions to improve health outcomes of older African American women with type 2 diabetes. Nurs Res 2009; 58: 410–418.		X			X		X			X																										
Spencer MS, Rosland AM, Kieffer EC, Sinco BR, Valerio M, Palmisano G et al. Effectiveness of a community health worker intervention among African American and Latino adults with type 2 diabetes: a randomized controlled trial. Am J Public Health 2011; 101: 2253–2260.		X			X		X																													
Toobert DJ, Strycker LA, Barrera M Jr, Osuna D, King DK, Glasgow RE. Outcomes from a multiple risk factor diabetes self-management trial for Latinas: ¡Viva Bien!. Ann Behav Med 2011; 41: 310–323.		X																																		

For peer review only

Vincent D, Pasvogel A, Barrera L. A feasibility study of a culturally tailored diabetes intervention for Mexican Americans. Biol Res Nurs 2007; 9: 130–141.		x			x					x						x																				
A.R.G. Look, R.R. Wing<br>Long-term effects of a lifestyle intervention on weight and cardiovascular risk factors in individuals with type 2 diabetes mellitus: four-year results of the Look AHEAD trial. Arch Intern Med, 170 (2010), pp. 1566–1575			x	x																																
D.A. Dobrosielski, B.B. Gibbs, P. Ouyang, S. Bonekamp, J.M. Clark, N.Y. Wang, et al.<br>Effect of exercise on blood pressure in type 2 diabetes: a randomized controlled trial<br>J Gen Intern Med, 27 (2012), pp. 1453–1459			x	x																																
W. Wisse, M. Boer Rookhuizen, M.D. de Kruif, J. van Rossum, I. Jordans, H. ten Cate, et al.<br>Prescription of physical activity is not sufficient to change sedentary behavior and improve glycemic control in type 2 diabetes patients<br>Diabetes Res Clin Pract, 88 (2010), pp. e10–e13			x	x																																
S. Balducci, S. Zanuso, A. Nicolucci, P. De Feo, S. Cavallo, P. Cardelli, et al.<br>Effect of an intensive exercise intervention strategy on modifiable cardiovascular risk factors in subjects with type 2 diabetes mellitus: a randomized controlled trial: the Italian Diabetes and Exercise Study (IDES)<br>Arch Intern Med, 170 (2010), pp. 1794–1803			x	x																																
A. Kirk, N. Mutrie, P. MacIntyre, M. Fisher<br>Effects of a 12-month physical activity counselling intervention on glycaemic control and on the status of cardiovascular risk factors in people with type 2 diabetes<br>Diabetologia, 47 (2004), pp. 821–832			x	x																		x														
K.J. Coppell, M. Kataoka, S.M. Williams, A.W. Chisholm, S.M. Vorgers, J.I. Mann<br>Nutritional intervention in patients with type 2 diabetes who are hyperglycaemic despite optimised drug treatment—Lifestyle Over and Above Drugs in Diabetes (LOADD) study: randomised controlled trial<br>BMJ, 341 (2010), p. c3337			x	x																																
M. Uusitupa, J. Laitinen, O. Siitonen, E. Vanninen, K. Pyorala<br>The maintenance of improved metabolic control after intensified diet therapy in recent type 2 diabetes<br>Diabetes Res Clin Pract, 19 (1993), pp. 227–238			x	x																										x					x	x
M. Ali, F. Schifano, P. Robinson, G. Phillips, L. Doherty, P. Melnick, et al.<br>Impact of community pharmacy diabetes monitoring and education programme on diabetes management: a randomized controlled study<br>Diabet Med, 29 (9) (2012), pp. e326–e333			x	x																																

For peer review only

H. Mohamed, B. Al-Lenjawi, P. Amuna, F. Zotor, H. Elmahdi Culturally sensitive patient-centred educational programme for self-management of type 2 diabetes: a randomized controlled trial Prim Care Diabetes, 7 (2013), pp. 199–206			x																																	
C.W. Chan, S.C. Siu, C.K. Wong, V.W. Lee A pharmacist care program: positive impact on cardiac risk in patients with type 2 diabetes J Cardiovasc Pharmacol Ther, 17 (2012), pp. 57–64			x	x																																
M.A. Sevick, M. Korytkowski, R.A. Stone, B. Piraino, D. Ren, S. Sereika, et al. Biophysiologic outcomes of the Enhancing Adherence in Type 2 Diabetes (ENHANCE) trial J Acad Nutr Diet, 112 (2012), pp. 1147–1157			x	x	x																															
W. Crasto, J. Jarvis, K. Khunti, T.C. Skinner, L.J. Gray, J. Brela, et al. Multifactorial intervention in individuals with type 2 diabetes and microalbuminuria: the Microalbuminuria Education and Medication Optimisation (MEMO) study Diabetes Res Clin Pract, 93 (2011), pp. 328–336			x	x																																
M.A. Salinero-Fort, E. Carrillo-de Santa Pau, F.J. Arrieta-Blanco, J.C. Abanades-Herranz, C. Martin-Madrazo, B. Rodes-Soldevila, et al. Effectiveness of PRECEDE model for health education on changes and level of control of HbA1c, blood pressure, lipids, and body mass index in patients with type 2 diabetes mellitus BMC Public Health, 11 (2011), p. 267			x		x																															
H. Sone, S. Tanaka, S. Iimuro, S. Tanaka, K. Oida, Y. Yamasaki, et al. Long-term lifestyle intervention lowers the incidence of stroke in Japanese patients with type 2 diabetes: a nationwide multicentre randomised controlled trial (the Japan Diabetes Complications Study) Diabetologia, 53 (2010), pp. 419–428			x	x																																
S.L. Krein, M.L. Klamerus, S. Vijan, J.L. Lee, J.T. Fitzgerald, A. Pawlow, et al. Case management for patients with poorly controlled diabetes: a randomized trial Am J Med, 116 (2004), pp. 732–739			x	x																		x														
M. Trento, P. Passera, M. Bajardi, M. Tomalino, G. Grassi, E. Borgo, et al. Lifestyle intervention by group care prevents deterioration of type II diabetes: a 4-year randomized controlled clinical trial. Diabetologia, 45 (2002), pp. 1231–1239			x	x													x	x				x						x				x				
AdachiMYamaokaKWatanabeMNishikawaMKobayashiI HidaEet alEffects of lifestyle education program for type 2 diabetes patients in clinics: a cluster randomized controlled trial.BMC Public Health201313467.					x																															

For peer review only - http://bmjopen.bmj.com/site/about/guidelines.xhtml

AdolfssonETWalker-EngströmMLSmideBWikbladKPatient education in type 2 diabetes: a randomized controlled 1-year follow-up study.Diabetes Res Clin Pract20077634150					X												X					X														
AmoakoESkellyAHRossenEKOutcomes of an intervention to reduce uncertainty among African American women with diabetes.West J Nurs Res20083092842					X					X																										
AndersonDRChristison-LagayJVillagraVLiuHDziuraJManaging the space between visits: a randomized trial of disease management for diabetes in a community health center.J Gen Intern Med201025111622					X																															
AndersonRMFunnellMMAikensJEKreinSLFitzgeraldJTNwankwoRet alEvaluating the efficacy of an empowerment-based self-management consultant intervention: results of a two-year randomized controlled trial.Ther Patient Educ20091311					X																															
AndersonRMFunnellMMButlerPMArnoldMSFitzgeraldJTFesteCCPatient empowerment. Results of a randomized controlled trial.Diabetes Care1995189439					X																													X		X
Anderson-LoftinWBarnett SBunnP SullivanPHusseyJTavakoli A Soul food light: culturally competent diabetes education.Diabetes Educ20053155563					X					X		X				X																				
BaksiAKAl-MrayatMHoganDWhittingstallEWilsonPWexJ Peer advisers compared with specialist health professionals in delivering a training programme on self-management to people with diabetes: a randomized controlled trial.Diabet Med200825107682					X																															
BarrattRFrostGMillwardDJTrubyHA randomised controlled trial investigating the effect of an intensive lifestyle intervention v.standard care in adults with type 2 diabetes immediately after initiating insulin therapy. Br J Nutr200899102531					X																															
Beverly EA, Fitzgerald SM, Brooks KM, Hultgren BA, Ganda OP,Munshi M, et al. Impact of reinforcement of diabetes self-care on poorly controlled diabetes: a randomized controlled trial. Diabetes Educ. 2013;39:504-14. [PMID: 23640303] doi:10.1177/0145721713486837					X																															
Bond GE, Burr R, Wolf FM, Price M, McCurry SM, Teri L. The effects of a web-based intervention on the physical outcomes associated with diabetes among adults age 60 and older: a randomized trial. Diabetes Technol Ther. 2007;9:52-9					X																															
Bozzetto L, Annuzzi G, Costabile G, Costagliola L, Giorgini M, Alderisio A, et al. A CHO/fibre diet reduces and a MUFA diet increases postprandial lipaemia in type 2 diabetes: no supplementary effects of low-volume physical training. Acta Diabetol. 2014;51:385-93					X																															
Bradshaw BG, Richardson GE, Kumpfer K, Carlson J,					X																															

For peer review only

Stanchfield J, Overall J, et al. Determining the efficacy of a resiliency training approach in adults with type 2 diabetes. Diabetes Educ. 2007;33: 650-9																																				
Brown SA, Blozis SA, Kouzekanani K, Garcia AA, Winchell M, Hanis CL. Dosage effects of diabetes self-management education for Mexican Americans: the Starr County Border Health Initiative. Diabetes Care. 2005;28:527-32					X					X																		X								
Brown SA, Garcı´a AA, Winter M, Silva L, Brown A, Hanis CL. Integrating education, group support, and case management for diabetic Hispanics. Ethn Dis. 2011;21:20-6					X																															
Castejo AM, Caldero JL, Perez A, Millar C, McLaughlinMiddlekauff J, Sangasubana N, et al. A community-based pilot study of a diabetes pharmacist intervention in Latinos: impact on weight and hemoglobin A1c. J Health Care Poor Underserved. 2013;24:48-60.					X																															
Chan JC, Sui Y, Oldenburg B, Zhang Y, Chung HH, Goggins W, et al; JADE and PEARL Project Team. Effects of telephone-based peer support in patients with type 2 diabetes mellitus receiving integrated care: a randomized clinical trial. JAMA Intern Med. 2014;174: 972-81.					X																															
Chan LS. Chronic disease self-management in Hong Kong Chinese older adults living in the community. Dissertation Abstracts International: Section B: The Sciences and Engineering. 2012;74:4-B E.					X																															
Cheong SH, McCargar LJ, Paty BW, Tudor-Locke C, Bell RC. The First Step First Bite Program: guidance to increase physical activity and daily intake of low-glycemic index foods. J Am Diet Assoc. 2009; 109:1411-6.					X																															
Chlebowy DO, El-Mallakh P, Myers J, Kubiak N, Cloud R, Wall MP. Motivational interviewing to improve diabetes outcomes in African Americans adults with diabetes. West J Nurs Res. 2015;37:566-80.					X																															
Clark M, Hampson SE, Avery L, Simpson R. Effects of a tailored lifestyle self-management intervention in patients with type 2 diabetes. Br J Health Psychol. 2004;9:365-79.					X																		X													
Cooper H, Booth K, Gill G. A trial of empowerment-based education in type 2 diabetes—global rather than glycaemic benefits. Diabetes Res Clin Pract. 2008;82:165-71.					X																															
Corkery E, Palmer C, Foley ME, Schechter CB, Frisher L, Roman SH. Effect of a bicultural community health worker on completion of diabetes education in a Hispanic population. Diabetes Care. 1997; 20:254-7.					X							X																						X		
Cramer JS, Sibley RF, Bartlett DP, Kahn LS, Loffredo L. An adaptation of the diabetes prevention program for use with high-risk, minority patients with type 2 diabetes.					X																	X														

For peer review only

Diabetes Educ. 2007;33:503-8																																				
Dasgupta K, Grover SA, Da Costa D, Lowensteyn I, Yale JF, Rahme E. Impact of modified glucose target and exercise interventions on vascular risk factors. Diabetes Res Clin Pract. 2006;72:53-60.					X																															
Davis RM, Hitch AD, Salaam MM, Herman WH, Zimmer-Galler IE, Mayer-Davis EJ. Tele Health improves diabetes self-management in an under served community: diabetes Tele Care. Diabetes Care. 2010;33:1712-7.					X																															
Deakin TA, Cade JE, Williams R, Greenwood DC. Structured patient education: the diabetes X-PERT Programme makes a difference. Diabet Med. 2006;23:944-54.					X												X											X								
Dunstan DW, Daly RM, Owen N, Jolley D, Vulikh E, Shaw J, et al. Home-based resistance training is not sufficient to maintain improved glycemic control following supervised training in older individuals with type 2 diabetes. Diabetes Care. 2005;28:3-9.					X																															
Dunstan DW, Mori TA, Puddey IB, Beilin LJ, Burke V, Morton AR, et al. The independent and combined effects of aerobic exercise and dietary fish intake on serum lipids and glycemic control in NIDDM. A randomized controlled study. Diabetes Care. 1997;20: 913-21.					X																															
Eakin EG, Winkler EA, Dunstan DW, Healy GN, Owen N, Marshall AM, et al. Living well with diabetes: 24-month outcomes from a randomized trial of telephone-delivered weight loss and physical activity intervention to improve glycemic control. Diabetes Care. 2014; 37:2177-85.					X																															
Edelman D, Dolor RJ, Coffman CJ, Pereira KC, Granger BB, Lindquist JH, et al. Nurse-led behavioral management of diabetes and hypertension in community practices: a randomized trial. J Gen Intern Med. 2015;30:626-33.					X																															
Fisher L, Hessler D, Glasgow RE, Arean PA, Masharani U, Naranjo D, et al. REDEEM: a pragmatic trial to reduce diabetes distress. Diabetes Care. 2013;36:2551-8.					X																															
Foster GD, Borradaile KE, Vander Veur SS, Leh Shantz K, Dilks RJ, Goldbacher EM, et al. The effects of a commercially available weight loss program among obese patients with type 2 diabetes: a randomized study. Postgrad Med. 2009;121:113-8.					X																															
Foster GD, Wadden TA, Lagrotte CA, Vander Veur SS, Hesson LA, Homko CJ, et al. A randomized comparison of a commercially available portion-controlled weight-loss intervention with a diabetes self-management education program. Nutr Diabetes. 2013;3:e63					X																															
Frosch DL, Uy V, Ochoa S, Mangione CM. Evaluation of a behavior support intervention for patients with poorly controlled diabetes. Arch Intern Med. 2011;171:2011-7.					X																															

For peer review only - http://bmjopen.bmj.com/site/about/guidelines.xhtml

Gagliardino JJ, Arrechea V, Assad D, Gagliardino GG, Gonza´lez L, Lucero S, et al. Type 2 diabetes patients educated by other patients perform at least as well as patients trained by professionals. Diabetes Metab Res Rev. 2013;29:152-60.					X																															
Gagliardino JJ, Lapertosa S, Pfirter G, Villagra M, Caporale JE, Gonzalez CD, et al; PRODIACOR. Clinical, metabolic and psychological outcomes and treatment costs of a prospective randomized trial based on different educational strategies to improve diabetes care (PRODIACOR). Diabet Med .2013;30:1102-11.					X																															
Giannopoulou I, Fernhall B, Carhart R, Weinstock RS, Baynard T, Figueroa A, et al. Effects of diet and/or exercise on the adipocytokine and inflammatory cytokine levels of postmenopausal women with type 2 diabetes. Metabolism. 2005;54:866-75					X																															
Glasgow RE, Kurz D, King D, Dickman JM, Faber AJ, Halterman E, et al. Twelve-month outcomes of an Internet-based diabetes selfmanagement support program. Patient Educ Couns. 2012;87:81-92.					X																															
Glasgow RE, Nutting PA, Toobert DJ, King DK, Strycker LA, Jex M, et al. Effects of a brief computer-assisted diabetes selfmanagement intervention on dietary, biological and quality-of-life outcomes. Chronic Illn. 2006;2:27-38.					X				X				X																	X						
Glasgow RE, Strycker LA, King DK, Toobert DJ, Rahm AK, Jex M, et al. Robustness of a computer-assisted diabetes self-management intervention across patient characteristics, healthcare settings, and intervention staff. Am J Manag Care. 2006;12:137-45.					X																	X														
Goudswaard AN, Stolk RP, Zuithoff NP, de Valk HW, Rutten GE. Long-term effects of self-management education for patients with Type 2 diabetes taking maximal oral hypoglycaemic therapy: a randomized trial in primary care. Diabet Med. 2004;21:491-6.					X														X			X		X				X								
Hawkins SY. Improving glycemic control in older adults using a videophone motivational diabetes self-management intervention.Res Theory Nurs Pract. 2010;24:217-32					X																															
Hendricks LE, Hendricks RT. The effect of diabetes selfmanagement education with frequent follow-up on the health outcomes of African American men. Diabetes Educ. 2000;26:995-1002.					X																															
Hermanns N, Kulzer B, Maier B, Mahr M, Haak T. The effect of an education programme (MEDIAS 2 ICT) involving intensive insulin treatment for people with type 2 diabetes. Patient Educ Couns. 2012;86:226-32.					X																															
Hill-Briggs F, Lazo M, Peyrot M, Doswell A, Chang YT, Hill MN, et al. Effect of problem-solving-based diabetes self-management training on diabetes control in a low income patient sample. J Gen Intern Med. 2011;26:972-8.					X																															
Holmen H, Torbjørnsen A, Wahl AK, Jenum AK, Småstuen MC, Arsand E, et al. A mobile health					X																															

For peer review only

intervention for self-management and lifestyle change for persons with type 2 diabetes, part 2: one year results from the Norwegian randomized controlled trial RENEWING HEALTH. JMIR Mhealth Uhealth. 2014;2:e57.																																				
Holtrop JS, Hickner J, Dosh S, Noel M, Ettenhofer TL. "Sticking to it—diabetes mellitus": a pilot study of an innovative behavior change program for women with type 2 diabetes. Am J Health Educ. 2002; 33:161-6.					X																															
Huisman S, de Gucht V, Maes S, Schroevers M, Chatrou M, Haak H. Self-regulation and weight reduction in patients with type 2 diabetes: a pilot intervention study. Patient Educ Couns. 2009;75:84-90.					X																															
Izquierdo RE, Knudson PE, Meyer S, Kearns J, Ploutz-Snyder R, Weinstock RS. A comparison of diabetes education administered through telemedicine versus in person. Diabetes Care. 2003;26: 1002-7.					X																															
Johnson ST, Bell GJ, McCargar LJ, Welsh RS, Bell RC. Improved cardiovascular health following a progressive walking and dietary intervention for type 2 diabetes. Diabetes Obes Metab. 2009;11: 836-43.					X																															
Jones H, Edwards L, Vallis TM, Ruggiero L, Rossi SR, Rossi JS, et al; Diabetes Stages of Change (DiSC) Study. Changes in diabetes self-care behaviors make a difference in glycemic control: the Diabetes Stages of Change (DiSC) study. Diabetes Care. 2003;26:732-7					X																															
Kim SH, Lee SH, Ahn KY, Lee DH, Suh YJ, Cho SG, et al. Effect of lifestyle modification on serum chemerin concentration and its association with insulin sensitivity in overweight and obese adults with type 2 diabetes. Clin Endocrinol (Oxf). 2014;80:825-33.					X																															
Kim SH, Lee SJ, Kang ES, Kang S, Hur KY, Lee HJ, et al. Effects of lifestyle modification on metabolic parameters and carotid intimamedia thickness in patients with type 2 diabetes mellitus. Metabolism. 2006;55:1053-9					X																															
Koo BK, Han KA, Ahn HJ, Jung JY, Kim HC, Min KW. The effects of total energy expenditure from all levels of physical activity vs. physical activity energy expenditure from moderate-to-vigorous activity on visceral fat and insulin sensitivity in obese Type 2 diabetic women. Diabet Med. 2010;27:1088-92					X																															
Kulzer B, Hermanns N, Reinecker H, Haak T. Effects of self management training in Type 2 diabetes: a randomized, prospective trial. Diabet Med. 2007;24:415-23.					X																															
Lorig K, Ritter PL, Laurent DD, Plant K, Green M, Jernigan VB, et al. Online diabetes self-management program: a randomized study. Diabetes Care. 2010;33:1275-81.					X				X				X	X																						
Lorig K, Ritter PL, Villa FJ, Armas J. Community-based peer-led diabetes self-management: a randomized trial. Diabetes Educ. 2009; 35:641-51.					X																															

Lynch EB, Liebman R, Ventrelle J, Avery EF, Richardson D. A self-management intervention for African Americans with comorbid diabetes and hypertension: a pilot randomized controlled trial. Prev Chronic Dis. 2014;11:E90.					X																															
Mandel SE, Davis BA, Secic M. Effects of music therapy and music-assisted relaxation and imagery on health-related outcomes in diabetes education: a feasibility study. Diabetes Educ. 2013;39:568-81.					X																															
Mayer-Davis EJ, D'Antonio AM, Smith SM, Kirkner G, Levin Martin S, Parra-Medina D, et al. Pounds off with empowerment (POWER): a clinical trial of weight management strategies for black and white adults with diabetes who live in medically underserved rural communities. Am J Public Health. 2004;94:1736-42.					X	X				X		X					X					X														
McGowan P. The efficacy of diabetes patient education and selfmanagement education in type 2 diabetes. Can J Diabetes. 2011;35: 46-53					X																															
Miller CK, Kristeller JL, Headings A, Nagaraja H. Comparison of a mindful eating intervention to a diabetes self-management intervention among adults with type 2 diabetes: a randomized controlled trial. Health Educ Behav. 2014;41:145-54.					X																															
Moncrieft AE. Randomized controlled trial of a behavioral weight loss intervention for primary prevention of renal decline in type 2 diabetics. Dissertation Abstracts International: Section B: The Sciences and Engineering. 2014;75:1-B E.					X																															
Moriyama M, Nakano M, Kuroe Y, Nin K, Niitani M, Nakaya T. Efficacy of a self-management education program for people with type 2 diabetes: results of a 12 month trial. Jpn J Nurs Sci. 2009;6:51-63.					X																															
Muchmore DB, Springer J, Miller M. Self-monitoring of blood glucose in overweight type 2 diabetic patients. Acta Diabetol. 1994; 31:215-9					X																															
Murrock CJ, Higgins PA, Killion C. Dance and peer support to improve diabetes outcomes in African American women. Diabetes Educ. 2009;35:995-1003.					X																															
Nishita C, Cardazone G, Uehara DL, Tom T. Empowered diabetes management: life coaching and pharmacist counseling for employed adults with diabetes. Health Educ Behav. 2013;40:581-91					X																															
Palmas W, Findley SE, Mejia M, Batista M, Teresi J, Kong J, et al. Results of the northern Manhattan diabetes community outreach project: a randomized trial studying a community health worker intervention to improve diabetes care in Hispanic adults. Diabetes Care. 2014;37:963-9.					X																															

For peer review only

Plotnikoff RC, Pickering MA, Glenn N, Doze SL, ReinboldMatthews ML, McLeod LJ, et al. The effects of a supplemental, theory-based physical activity counseling intervention for adults with type 2 diabetes. J Phys Act Health. 2011;8:944-54					x																															
Prezio EA, Cheng D, Balasubramanian BA, Shuval K, Kendzor DE, Culica D. Community Diabetes Education (CoDE) for uninsured Mexican Americans: a randomized controlled trial of a culturally tailored diabetes education and management program led by a community health worker. Diabetes Res Clin Pract. 2013;100:19-28					x																															
Reaney M, Zorzo EG, Golay A, Hermanns N, Cleall S, Petzinger U, et al. Impact of Conversation Map education tools versus regular care on diabetes-related knowledge of people with type 2 diabetes: a randomized, controlled study. Diabetes Spectr. 2013;26:236-45.					x																															
Rickheim PL, Weaver TW, Flader JL, Kendall DM. Assessment of group versus individual diabetes education: a randomized study. Diabetes Care. 2002;25:269-74.					x																			x								x				
Ridgeway NA, Harvill DR, Harvill LM, Falin TM, Forester GM,<br>Gose OD. Improved control of type 2 diabetes mellitus: a practical education/behavior modification program in a primary care clinic. South Med J. 1999;92:667-72.					x																		x											x	x	x
Rock CL, Flatt SW, Pakiz B, Taylor KS, Leone AF, Brelje K, et al. Weight loss, glycemic control, and cardiovascular disease risk factors in response to differential diet composition in a weight loss program in type 2 diabetes: a randomized controlled trial. Diabetes Care. 2014;37:1573-80.					x																															
Rosal MC, Heyden R, Mejilla R, Capelson R, Chalmers KA, Rizzo DePaoli M, et al. A virtual world versus face-to-face intervention format to promote diabetes self-management among African American women: a pilot randomized clinical trial. JMIR Res Protoc. 2014;3: e54.					x																															
Rosal MC, Ockene IS, Restrepo A, White MJ, Borg A, Olendzki B, et al. Randomized trial of a literacy-sensitive, culturally tailored diabetes self-management intervention for low-income latinos: latinos en control. Diabetes Care. 2011;34:838-44.					x		x			x																										
Ruggiero L, Moadsiri A, Butler P, Oros SM, Berbaum ML, Whitman S, et al. Supporting diabetes self-care in underserved populations: a randomized pilot study using medical assistant coaches. Diabetes Educ. 2010;36:127-31.					x					x																										
Ruggiero L, Riley BB, Hernandez R, Quinn LT, Gerber BS, Castillo A, et al. Medical assistant coaching to support diabetes self-care among low-income racial/ethnic minority populations: randomized controlled trial. West J Nurs Res. 2014;36:1052-73					x																															
Sacco WP, Malone JI, Morrison AD, Friedman A, Wells K. Effect of a brief, regular telephone intervention by					x																															

paraprofessionals for type 2 diabetes. J Behav Med. 2009;32:349-59.																																				
Sarkadi A, Rosenqvist U. Experience-based group education in Type 2 diabetes: a randomised controlled trial. Patient Educ Couns. 2004;53:291-8					X												X					X						X								
Shibayama T, Kobayashi K, Takano A, Kadowaki T, Kazuma K. Effectiveness of lifestyle counseling by certified expert nurse of Japan for non-insulin-treated diabetic outpatients: a 1-year randomized controlled trial. Diabetes Res Clin Pract. 2007;76:265-8.					X																	X		X												
Sigurdardottir AK, Benediktsson R, Jonsdottir H. Instruments to tailor care of people with type 2 diabetes. J Adv Nurs. 2009;65:2118-30.					X																															
Siminerio L, Ruppert KM, Gabbay RA. Who can provide diabetes self-management support in primary care? Findings from a randomized controlled trial. Diabetes Educ. 2013;39:705-13.					X																															
Sinclair KA, Makahi EK, Shea-Solatorio C, Yoshimura SR, Townsend CK, Kaholokula JK. Outcomes from a diabetes self-management intervention for Native Hawaiians and Pacific People: Partners in Care. Ann Behav Med. 2013;45:24-32.					X																															
Smith DE, Heckemeyer CM, Kratt PP, Mason DA. Motivational interviewing to improve adherence to a behavioral weight-control program for older obese women with NIDDM. A pilot study. Diabetes Care. 1997;20:52-4.					X							X											X											X		
Sorkin DH, Mavandadi S, Rook KS, Biegler KA, Kilgore D, Dow E, et al. Dyadic collaboration in shared health behavior change: the effects of a randomized trial to test a lifestyle intervention for highrisk Latinas. Health Psychol. 2014;33:566-75.					X																															
Sperl-Hillen J, Beaton S, Fernandes O, Von Worley A, VazquezBenitez G, Hanson A, et al. Are benefits from diabetes self-management education sustained? Am J Manag Care. 2013;19:104-12					X																															
Steed L, Lankester J, Barnard M, Earle K, Hurel S, Newman S. Evaluation of the UCL diabetes self-management programme (UCLDSMP): a randomized controlled trial. J Health Psychol. 2005;10: 261-76					X																	X														
Sung K, Bae S. Effects of a regular walking exercise program on behavioral and biochemical aspects in elderly people with type II diabetes. Nurs Health Sci. 2012;14:438-45.					X																															
Tang TS, Funnell M, Sinco B, Piatt G, Palmisano G, Spencer MS, et al. Comparative effectiveness of peer leaders and community health workers in diabetes self-management support: results of a randomized controlled trial. Diabetes Care. 2014;37:1525-34					X																															
Thoolen B, De Ridder D, Bensing J, Maas C, Griffin S, Gorter K, et al. Effectiveness of a self-management intervention in patients with screen-detected type 2 diabetes. Diabetes Care. 2007;30:2832-7					X																															

For peer review only

Toobert DJ, Glasgow RE, Strycker LA, Barrera M Jr, Radcliffe JL, Wander RC, et al. Biologic and quality-of-life outcomes from the Mediterranean Lifestyle Program: a randomized clinical trial. Diabetes Care. 2003;26:2288-93					X												X					X														
Toobert DJ, Strycker LA, King DK, Barrera M Jr, Osuna D, Glasgow RE. Long-term outcomes from a multiple-risk-factor diabetes trial for Latinas: Viva Bien!. Transl Behav Med. 2011;1:416-426.					X					X																										
Trief P, Sandberg JG, Ploutz-Snyder R, Brittain R, Cibula D, Scales K, et al. Promoting couples collaboration in type 2 diabetes: the diabetes support project pilot data. Fam Syst Health. 2011;29:253-61.					X																															
Tucker CM, Lopez MT, Campbell K, Marsiske M, Daly K, Nghiem K, et al. The effects of a culturally sensitive, empowermentfocused, community-based health promotion program on health outcomes of adults with type 2 diabetes. J Health Care Poor Underserved. 2014;25:292-307.					X																															
Utz SW, Williams IC, Jones R, Hinton I, Alexander G, Yan G, et al. Culturally tailored intervention for rural African Americans with type 2 diabetes. Diabetes Educ. 2008;34:854-65					X																															
Vadstrup ES, Frølich A, Perrild H, Borg E, Røder M. Effect of a group-based rehabilitation programme on glycaemic control and cardiovascular risk factors in type 2 diabetes patients: the Copenhagen Type 2 Diabetes Rehabilitation Project. Patient Educ Couns. 2011;84:185-90.					X																															
Varney JE, Weiland TJ, Inder WJ, Jelinek GA. Effect of hospitalbased telephone coaching on glycaemic control and adherence to management guidelines in type 2 diabetes, a randomised controlled trial. Intern Med J. 2014;44:890-7.					X																															
Vazquez IM, Millen B, Bissett L, Levenson SM, Chipkin SR. Buena Alimentacion, Buena Salud: a preventive nutrition intervention in Caribbean Latinos with type 2 diabetes. Am J Health Promot. 1998;13:116-9.					X					X																										
Walker EA, Shmukler C, Ullman R, Blanco E, ScollanKoliopoulus M, Cohen HW. Results of a successful telephonic intervention to improve diabetes control in urban adults: a randomized trial. Diabetes Care. 2011;34:2-7.					X					X																										
Weinger K, Beverly EA, Lee Y, Sitnokov L, Ganda OP, Caballero AE. The effect of a structured behavioral intervention on poorly controlled diabetes: a randomized controlled trial. Arch Intern Med. 2011;171:1990-9.					X																															
Welch G, Zagarins SE, Feinberg RG, Garb JL. Motivational interviewing delivered by diabetes educators: does it improve blood glucose control among poorly controlled type 2 diabetes patients? Diabetes Res					X																															

For peer review only

Clin Pract. 2011;91:54-60																																				
Welschen LM, van Oppen P, Bot SD, Kostense PJ, Dekker JM, Nijpels G. Effects of a cognitive behavioural treatment in patients with type 2 diabetes when added to managed care; a randomised controlled trial. J Behav Med. 2013;36:556-66.					X																															
West DS, DiLillo V, Bursac Z, Gore SA, Greene PG. Motivational interviewing improves weight loss in women with type 2 diabetes. Diabetes Care. 2007;30:1081-7.					X	X						X										X														
Wierenga ME. Life-style modification for weight control to improve diabetes health status. Patient Educ Couns. 1994;23:33-40.					X																															X
Wing RR, Bolin P, Brancati FL, Bray GA, Clark JM, Coday M, et al; Look AHEAD Research Group. Cardiovascular effects of intensive lifestyle intervention in type 2 diabetes. N Engl J Med. 2013;369:145-54.					X	X																														
Wolever RQ, Dreusicke M, Fikkan J, Hawkins TV, Yeung S, Wakefield J, et al. Integrative health coaching for patients with type 2 diabetes: a randomized clinical trial. Diabetes Educ. 2010;36:629-39.					X																															
Wolf AM, Conaway MR, Crowther JQ, Hazen KY, L Nadler J, Oneida B, et al; Improving Control with Activity and Nutrition (ICAN) Study. Translating lifestyle intervention to practice in obese patients with type 2 diabetes: Improving Control with Activity and Nutrition (ICAN) study. Diabetes Care. 2004;27:1570-6.					X	X														X		X														
Yoo JS, Lee SJ, Lee HC, Kim MJ. The effect of a comprehensive lifestyle modification program on glycemic control and body composition in patients with type 2 diabetes. Asian Nurs Res (Korean Soc Nurs Sci). 2007;1:106-15.					X																															
Yuan C, Lai CW, Chan LW, Chow M, Law HK, Ying M. The effect of diabetes self-management education on body weight, glycemic control, and other metabolic markers in patients with type 2 diabetes mellitus. J Diabetes Res. 2014;2014:789761.					X																															
Dixon JB, O'Brien PE, Playfair J et al. Adjustable gastric banding and conventional therapy for type 2 diabetes: a randomized controlled trial. JAMA 2008; 299: 316–323.						X																														
Aas AM, Bergstad I, Thorsbyt PM, Johannesen O, Solberg M, Birkelandt KI. An intensified lifestyle intervention programme may be superior to insulin treatment in poorly controlled Type 2 diabetic patients on oral hypoglycaemic agents: results of a feasibility study. Diabet Med 2005; 22: 316–322.						X																														
Luley C, Blaik A, Reschke K, Klose S, Westphal S. Weight loss in obese patients with type 2 diabetes: effects of telemonitoring plus a diet combination-the Active Body Control (ABC) Program. Diabetes Res Clin						X																														

For peer review only - http://bmjopen.bmj.com/site/about/guidelines.xhtml

Pract 2011; 91: 286–292.																																				
Redmon JB, Raatz SK, Reck KP et al. One-year outcome of a combination of weight loss therapies for subjects with type 2 diabetes: a randomized trial. Diabetes Care 2003; 26: 2505–2511.						X																														
Eakin EG, Reeves MM, Winkler E et al. Six-month outcomes from living well with diabetes: a randomized trial of a telephone-delivered weight loss and physical activity intervention to improve glycemic control. Ann Behav Med 2013; 46: 193–203.						X																														
Snel M, Gastaldelli A, Ouwens DM et al. Effects of adding exercise to a 16-week very low-calorie diet in obese, insulin-dependent type 2 diabetes mellitus patients. J Clin Endocrinol Metab 2012; 97: 2512–2520.						X																														
Palmer AJ, Roze S, Valentine WJ, Spinas GA, Shaw JE, et al. (2004) Intensive lifestyle changes or metformin in patients with impaired glucose tolerance: modelling the long-term health economic implications of the diabetes prevention program in Australia, France, Germany, Switzerland, and the United Kingdom. Clin Ther 26:304–321								X																												
Lindgren P, Lindström J, Tuomilehto J, Uusitupa M, Peltonen M, et al. (2007) Lifestyle intervention to prevent diabetes in men and women with impaired glucose tolerance is cost-effective. Int J Technol Assess Health Care 23: 177-183.								X																												
Tuomilehto J, Lindström J, Eriksson JG, Valle TT, Hämäläinen H, et al. (2001) Finnish Diabetes Prevention Study Group. Prevention of type 2 diabetes mellitus by changes in lifestyle among subjects with impaired glucose tolerance. N Engl J Med 344:1343-1350								X																												
Lindström J, Eriksson JG, Valle TT, Aunola S, Cepaitis Z, et al. (2003) Prevention of diabetes mellitus in subjects with impaired glucose tolerance in the Finnish Diabetes Prevention Study: results from a randomized clinical trial. J Am Soc Nephrol. 14:S108-S113								X																												
Palmer AJ, Tucker DM (2012) Cost and clinical implications of diabetes prevention in an Australian setting: a long-term modeling analysis. Prim Care Diabetes 6: 109-121.								X																												
Diabetes Prevention Program Research Group, Knowler WC, Fowler SE, Hamman RF, Christophi CA, et al. (2009) 10-year follow-up of diabetes incidence and weight loss in the Diabetes Prevention Program Outcomes Study. Lancet 374:1677-1686.								X																												
Herman WH, Hoerger TJ, Brandle M, Hicks K, Sorensen S, et al. (2005) The cost-effectiveness of lifestyle modifcation or metformin in preventing type 2 diabetes in adults with impaired glucose tolerance. Ann Intern Med 142: 323-								X																												

For peer review only - http://bmjopen.bmj.com/site/about/guidelines.xhtml

332.																																				
CDC Diabetes Cost-effectiveness Group (2002) Cost-effectiveness of intensive glycemic control, intensifed hypertension control, and serum cholesterol level reduction for type 2 diabetes. JAMA 287: 2542-2551								X																												
Diabetes Prevention Program Research Group1 (2012) The 10-year costeffectiveness of lifestyle intervention or metformin for diabetes prevention: an intent-to-treat analysis of the DPP/DPPOS. Diabetes Care 35: 723-730								X																												
Herman WH, Edelstein SL, Ratner RE, Montez MG, Ackermann RT, et al. (2013) Diabetes Prevention Program Research Group. Effectiveness and costeffectiveness of diabetes prevention among adherent participants. Am J Manag Care 19:194-202								X																												
Diabetes Prevention Program Research Group (2003) Within-trial costeffectiveness of lifestyle intervention or metformin for the primary prevention of type 2 diabetes. Diabetes Care 26: 2518-2523								X																												
Eddy DM1, Schlessinger L (2003) Archimedes: a trial-validated model of diabetes. Diabetes Care 26: 3093-3101.								X																												
Mason JM, Freemantle N, Gibson JM, New JP (2005) SPLINT trial. Specialist nurse-led clinics to improve control of hypertension and hyperlipidemia in diabetes: economic analysis of the SPLINT trial. Diabetes Care 28:40–46								X																												
New JP, Mason JM, Freemantle N, Teasdale S, Wong LM, et al. (2003) Specialist nurse-led intervention to treat and control hypertension and hyperlipidemia in diabetes (SPLINT): a randomized controlled trial. Diabetes Care 26: 2250-2255.								X																												
Gillett M, Dallosso HM, Dixon S, Brennan A, Carey ME, et al. (2010) Delivering the diabetes education and self-management for ongoing and newly diagnosed (DESMOND) programme for people with newly diagnosed type 2 diabetes: cost effectiveness analysis. BMJ 341:c4093								X																												
Davies MJ, Heller S, Skinner TC, Campbell MJ, Carey ME, et al. (2008) Diabetes Education and Self-Management for Ongoing and Newly Diagnosed Collaborative. Effectiveness of the diabetes education and self-management for ongoing and newly diagnosed (DESMOND) programme for people with newly diagnosed type 2 diabetes: cluster randomised controlled trial. BMJ 336:491-495								X																												
Dijkstra RF, Niessen LW, Braspenning JC, Adang E, Grol RT (2006) Patientcentred and professional-directed implementation strategies for diabetes guidelines: a cluster-randomized trial-based cost-effectiveness analysis. Diabet Med 23:164-170.								X																												
Dijkstra RF, Braspenning JC, Huijsmans Z, Akkermans RP, van Ballegooie E, et al. (2005) Introduction of								X																												

For peer review only - http://bmjopen.bmj.com/site/about/guidelines.xhtml

diabetes passports involving both patients and professionals to improve hospital outpatient diabetes care. Diabetes Res Clin Pract 68: 126-134.																																				
Quinn CC, Clough SS, Minor JM, Lender D, Okafor MC, Gruber-Baldini A. WellDoc mobile diabetes management randomized controlled trial: change in clinical and behavioral outcomes and patient and physician satisfaction. Diabetes Technol Ther 2008;10:160–168									X				X																							
Smith L, Weinert C. Telecommunication support for rural women with diabetes. Diabetes Educ 2000;26:645–655									X				X																							
Glasgow RE, Nutting PA, King DK, et al. Randomized effectiveness trial of a computer-assisted intervention to improve diabetes care. Diabetes Care 2005;28:33–39									X				X																							
Christian JG, Bessesen DH, Byers TE, Christian KK, Goldstein MG, Bock BC. Clinic-based support to help overweight patients with type 2 diabetes increase physical activity and lose weight. Arch Intern Med 2008;168:141–146									X				X																							
Glasgow RE, La Chance PA, Toobert DJ, Brown J, Hampson SE, Riddle MC. Long-term effects and costs of brief behavioural dietary intervention for patients with diabetes delivered from the medical office. Patient Educ Couns 1997;32:175–184									X				X																					X		
Leu MG, Norris TE, Hummel J, Isaac M, Brogan MW. A randomized, controlled trial of an automated wireless messaging system for diabetes. Diabetes Technol Ther 2005;7:710–718; discussion 719–720									X				X																							
Lim S, Kang SM, Shin H, et al. Improved glycemic control without hypoglycemia in elderly diabetic patients using the ubiquitous healthcare service, a new medical information system. Diabetes Care 2011;34:308–313									X				X																							
Quinn CC, Shardell MD, Terrin ML, Barr EA, Ballew SH, Gruber-Baldini AL. Cluster-randomized trial of a mobile phone personalized behavioral intervention for blood glucose control. Diabetes Care 2011;34:1934–1942									X				X																							
Wise PH, Dowlatshahi DC, Farrant S, Fromson S, Meadows KA. Effect of computer-based learning on diabetes knowledge and control. Diabetes Care 1986;9:504–508									X				X																					X	X	X
Yoo HJ, Park MS, Kim TN, et al. A Ubiquitous Chronic Disease Care system using cellular phones and the internet. Diabet Med 2009;26:628–635pmid.									X		X		X																							
Glasgow RE, Kurz D, King D, et al. Outcomes of minimal and moderate support versions of an internet-based diabetes self-management support program. J Gen Intern Med 2010;25:1315–1322									X				X	X																						
Glasgow RE, Boles SM, McKay HG, Feil EG, Barrera M									X				X	X						X																

Jr. The D-Net diabetes self-management program: long-term implementation, outcomes, and generalization results. Prev Med 2003;36:410–419																																				
Zhou YD, Gu W. [Computer assisted nutrition therapy for patients with type 2 diabetes]. Zhejiang Da Xue Xue Bao Yi Xue Ban 2003;32:244–248									X																											
Lo R, Lo B, Wells E, Chard M, Hathaway J. The development and evaluation of a computer-aided diabetes education program. Aust J Adv Nurs 1996;13:19–27.									X				X																							X
Anderson-Loftin W, Barnett S, Sullivan P, Bunn PS, Anderson-Loftin W, Barnett S, Sullivan P, Bunn PS, Tavakoli A: Culturally competent dietary education for southern rural African Americans with diabetes. Diabetes Educ. 2002, 28 (2): 245-257.										X																										
Banister NA, Jastrow ST, Hodges V, Loop R, Gillham MB: Diabetes self-management training program in a community clinic improves patient outcomes at modest cost. J Am Diet Assoc. 2004, 104 (5): 807-810.										X																										
Basch CE, Walker EA, Howard CJ, Shamoon H, Zybert P: The effect of health education on the rate of ophthalmic examinations among African Americans with diabetes mellitus. Am J Public Health. 1999, 89 (12): 1878-1882.										X																										
Borges WJ, Ostwald SK: Improving foot self-care behaviors with Pies Sanos. West J Nurs Res. 2008, 30 (3): 325-341.										X																										
Brown SA, Hanis CL: A community-based, culturally sensitive education and group-support intervention for Mexican Americans with NIDDM: a pilot study of efficacy. Diabetes Educ. 1995, 21 (3): 203-210.										X																										
Culica D, Walton JW, Prezio EA: CoDE: Community Diabetes Education for uninsured Mexican Americans. Proc (Bayl Univ Med Cent). 2007, 20 (2): 111-117.										X																										
Davis RM, Hitch AD, Salaam MM, Herman WH, Zimmer-Galler IE, Mayer-Davis EJ: TeleHealth Improves Diabetes Self-Management in an Underserved Community: Diabetes TeleCare. Diabetes Care. 2010, 33 (8): 1712-1717.										X																										
Ebbesson SO, Ebbesson LO, Swenson M, Kennish JM, Robbins DC: A successful diabetes prevention study in Eskimos: the Alaska Siberia project. Int J Circumpolar Health. 2005, 64 (4): 409-424.										X																										
Elshaw EB, Young EA, Saunders MJ, McGurn WC, Lopez LC: Utilizing a 24-hour dietary recall and culturally specific diabetes education in Mexican Americans with diabetes. Diabetes Educ. 1994, 20 (3): 228-235.										X		X																								X
Long JA, Jahnle EC, Richardson DM, Loewenstein G, Volpp KG: Peer mentoring and financial incentives to improve glucose control in african american veterans: a randomized trial. Ann Intern Med. 2012, 156 (6): 416-424.										X																										

For peer review only

Mauldon M, Melkus GD, Cagganello M, Mauldon M, Melkus GDE, Cagganello M: Tomando Control: a culturally appropriate diabetes education program for Spanish-speaking individuals with type 2 diabetes mellitus–evaluation of a pilot project. Diabetes Educ. 2006, 32 (5): 751-760.										X																										
Melkus GD, Spollett G, Jefferson V, Chyun D, Tuohy B, Robinson T, Kaisen A: A culturally competent intervention of education and care for black women with type 2 diabetes. Appl Nurs Res. 2004, 17 (1): 10-20.										X																										
Rimmer JH, Silverman K, Braunschweig C, Quinn L, Liu Y, Feasibility of a health promotion intervention for a group of predominantly African American women with type 2 diabetes. Diabetes Educ. 2002, 28 (4): 571-580.										X																										
Schillinger D, Handley M, Wang F, Hammer H, Schillinger D, Handley M, Wang F, Hammer H: Effects of self-management support on structure, process, and outcomes among vulnerable patients with diabetes: a three-arm practical clinical trial. Diabetes Care. 2009, 32 (4): 559-566.										X																										
Two FJ, Kieffer EC, Palmisano G, Anderson M, Sinco B, Janz N, Heisler M, Spencer M, Guzman R, Thompson J, Wisdom K, James SA: Racial and Ethnic Approaches to Community Health (REACH) Detroit partnership: improving diabetes-related outcomes among African American and Latino adults. Am J Public Health. 2005, 95 (9): 1552-1560.										X																										
C.T. Liu, Y.T. Yeh, T.I. Lee, Y.C. Li Observations on online services for diabetes management Diab. Care, 28 (2005), pp. 2807–2808											X																									
H.S. Kim A randomized controlled trial of a nurse short-message service by cellular phone for people with diabetes Int. J. Nurs. Stud., 44 (2007), pp. 687–692											X																									
Z. Faridi, L. Liberti, K. Shuval, V. Northrup, A. Ali, D.L. Katz Evaluating the impact of mobile telephone technology on type 2 diabetic patients' self-management: the NICHE pilot study J. Evaluation Clin. Pract., 14 (2008), pp. 465–469											X																									
K.H. Yoon, H.S. Kim A short message service by cellular phone in type 2 diabetic patients for 12 months Diab. Res. Clin. Pract., 79 (2008), pp. 256–261											X																									
C.S. Kim, S.Y. Park, J.G. Kang, S.J. Lee, S.H. Ihm, M.G. Choi, H.J. Yoo Insulin dose titration system in diabetes patients using a short messaging service automatically produced by a knowledge matrix Diab. Technol. Therapeut., 12 (2010), pp. 663–669											X																									

For peer review only

A.S. Shetty, S. Chamukuttan, A. Nanditha, R.K. Raj, A. Ramachandran<br>Reinforcement of adherence to prescription recommendations in Asian Indian diabetes patients using short message service (SMS) – a pilot study<br>J. Assoc. Phys. India, 59 (2011), pp. 711–714											X																									
W.I. Hussein, K. Hasan, A.A. Jaradat<br>Effectiveness of mobile phone short message service on diabetes mellitus management; the SMS-DM study<br>Diab. Res. Clin. Pract., 94 (2011), pp. e24–e26											X																									
M. Goodarzi, I. Ebrahimzadeh, A. Rabi, B. Saedipoor, M.A. Jafarabadi<br>Impact of distance education via mobile phone text messaging on knowledge, attitude, practice and self efficacy of patients with type 2 diabetes mellitus in Iran<br>J. Diab. Metabolic Disorders, 11 (2012), p. 10											X																									
S. Arora, A.L. Peters, E. Burner, C.N. Lam, M. Menchine<br>Trial to Examine Text Message-based mHealth in emergency department patients with diabetes (TExT-MED): a randomized controlled trial<br>Ann. Emerg. Med. (2013) (Epub ahead of print)											X																									
R.M. Anderson, M.M. Funnell, M.S. Arnold, P.A. Barr, G.J. Edwards, J.T. Fitzgerald<br>Assessing the cultural relevance of an education program for urban African Americans with diabetes<br>Diabetes Educ, 26 (2000), pp. 280–289												X																								
T.L. Gary, L.R. Bone, M.N. Hill, D.M. Levine, M. McGuire, C. Saudek, et al.<br>Randomized controlled trial of the effects of nurse case manager and community health worker interventions on risk factors for diabetes-related complications in urban African Americans<br>Prev Med, 37 (2003), pp. 23–32												X										X														
L.A. Jaber, H. Halapy, M. Fernet, S. Tummalapalli, H. Diwakaran<br>Evaluation of a pharmaceutical care model on diabetes management<br>Ann Pharmacother, 30 (1996), pp. 238–243												X							X										X				X			
W.L. McNabb, M.T. Quinn, L. Rosing<br>Weight loss program for inner-city black women with non-insulin-dependent diabetes mellitus: pathways<br>J Am Diet Assoc, 93 (1993), pp. 75–77												X																								
Zhou Y, Wei GU. Computer-assisted nutrition therapy for patients with type 2 diabetes. Journal of Zhejiang University (Medical Sciences) 2003;32(3):244-8.													X																							
van Bastelaar KM, Pouwer F, Cuijpers P, Riper H, Snoek FJ. Web-based depression treatment for type 1 and type 2 diabetic patients: a randomized, controlled trial. Diabetes Care 2011 Feb;34(2):320-325														X																						
Glasgow RE, Kurz D, King D, Dickman JM, Faber AJ,														X																						

For peer review only

Halterman E, et al. Twelve-month outcomes of an Internet-based diabetes self-management support program. Patient Educ Couns 2012 Apr;87(1):81-92																																				
Bond GE, Burr RL, Wolf FM, Feldt K. The effects of a web-based intervention on psychosocial well-being among adults aged 60 and older with diabetes: a randomized trial. Diabetes Educ 2010;36(3):446-456.														X						X																
Bond GE, Burr R, Wolf FM, Price M, McCurry SM, Teri L. The effects of a web-based intervention on the physical outcomes associated with diabetes among adults age 60 and older: a randomized trial. Diabetes Technol Ther 2007 Feb;9(1):52-59														X						X																
Bond GE, Burr R, Wolf FM, Price M, McCurry SM, Teri L. Preliminary findings of the effects of comorbidities on a web-based intervention on self-reported blood sugar readings among adults age 60 and older with diabetes. Telemed J E Health 2006 Dec;12(6):707-710.														X																						
Barrera M, Glasgow RE, McKay HG, Boles SM, Feil EG. Do Internet-based support interventions change perceptions of social support?: An experimental trial of approaches for supporting diabetes self-management. Am J Community Psychol 2002 Oct;30(5):637-654.														X																						
McKay HG, Glasgow RE, Feil EG, Boles SM, Barrera MJ. Internet-based diabetes self-management and support: Initial outcomes from the Diabetes Network project. Rehabilitation Psychology 2002;47(1):31-48.														X						X																
Lorig KR, Ritter PL, Laurent DD, Plant K. Internet-based chronic disease self-management: a randomized trial. Med Care 2006 Nov;44(11):964-971														X																						
Kim CJ, Kang DH. Utility of a Web-based intervention for individuals with type 2 diabetes: the impact on physical activity levels and glycemic control. Comput Inform Nurs 2006;24(6):337-345.														X						X																
Wangberg SC. An Internet-based diabetes self-care intervention tailored to self-efficacy. Health Educ Res 2008 Feb;23(1):170-179.														X																						
Barth R, Campbell L V, Allen S, Jupp J J, Chisholm D J. Intensive education improves knowledge, compHance, and foot problems in type 2 diabetes. Diabetic Medicine 1991; 8(2):111-117															X																			X		X
Malone J M, Snyder M, Anderson G, Bernhard V M, Holloway G A (Jnr), Bunt T J. Prevention of amputation by diabetic education. American Journal of Surgeiy 1989; 158(6): 520-523; discussion 523-524.															X																			X		X
Sun P C, Jao S E, Lin H D, Chan R C, Chou C L, Wei S H. Improving preventive foot care for diabetic patients participating in group education. Journal of American Podiatric Medicine Association 2009; 99: 295-300.															X																					
Borges W J, Ostwald S K. Improving foot selfcare behaviors with Pies Sanos. Journal of the American Podiatric Medical Association 2008; 100(2): 101-104.															X																					
Corbett CEA Randomized pilot study of improving foot care in home health patients with diabetes. The Diabetes Educator 2003; 29: 273.															X																					

For peer review only - http://bmjopen.bmj.com/site/about/guidelines.xhtml
For peer review only

Lincoln N B, Radford K A, Game F L, Jeffcoate W J. Education for secondary prevention of foot ulcers in people with diabetes: a randomised controlled trial. Diabetologia 2008; 5 1 :1954-1961.														X																						
Litzelman D K, Slemenda S W Langefeld C D, Hays L M, Welch M A, et a!. Reduction of lower extremity clinical abnormalities in patients with non-insulin-dependent diabetes mellitus. A randomized, controlled trial. Annals of Internal Medicine 1993; 119(1): 36-41.														X																						
Viswanathan V, Madhavan S, Rajasekar S, Chamukuttan S, Ambady R. Amputation prevention initiative in South India Positive impact of foot care education. Diabetes Care 2005; 28:1019-1021.														X																						
Skelly AH, Carlson JR, Leeman J, Holditch-Davis D, Soward A. Symptoms-focused management for African American women with type 2 diabetes: a pilot study. Appl Nurs Res. 2005; 18 (4): 213–220.															X					X					X											
Cabrera-Pivaral CE, Gonzalez-Perez G, Vega-Lopez MG, Rias-Merino ED: Impact of participatory education on body mass index and blood glucose in obese type-2 diabetics. Cadernos de Saude Publica. 2004, 8: 275-281.																X																				
Clancy DE, Huang P, Okonofua E, Yeager D, Magruder KM: Group visits: promoting adherence to diabetes guidelines. Soc Gen Intern Med. 2007, 16: 620-624.																X	X																			
Cooper HC, Booth K, Gill G: Patients' perspectives on diabetes health care education. Theory Pract. 2003, 18: 191-206.																X											X									
Heller SR, Clarke P, Daly H, Davis I, McCulloch DK, Allison SP, et al: Group education for obese patients with type 2 diabetes: greater success at less cost. Diabet Med. 1988, 5: 552-556.																X											X				X		X	X	X
Holtrop JS, Hickner J, Dosh S, Noel M, Ettenhofer TL: "Sticking to it – diabetes mellitus": a pilot study of an innovative behavior change program for women with type 2 diabetes. Am J Heal Educ. 2002, 33: 161-166.																X															X				
Hornsten A, Lundman B, Stenlund H, Sandstrom H: Metabolic improvement after intervention focusing on personal understanding in type 2 diabetes. Diabetes Res Clin Pract. 2004, 68: 65-74.																X																				
Lozano ML, Armale MJ, Tena DI, Sanchez NC: The education of type-2 diabetics: why not in groups?. Aten Primaria. 1999, 23: 485-492.																X															X				
McKibbin CL, Patterson TL, Norman G, Patrick K, Jin H, Roesch S, et al: A lifestyle intervention for older schizophrenia patients with diabetes mellitus: a randomized controlled trial. Schizophr Res. 2006, 8: 36-44.																X																				
Pennings-van der Eerden LM, Ripken T, van Heijst MP, Schrijvers AP: Evaluation of the impact of a patient education programme for NIDDM patients on knowledge, self-care behaviour, metabolic control and lipid profiles. Gedran en gezondheid. 1991, 19: 246-260.																X																				

For peer review only - http://bmjopen.bmj.com/site/about/guidelines.xhtml

Toobert DJ, Glasgow RE, Strycker LA, Barrera M, Ritzwoller DP, Weidner G: Long-term effects of the Mediterranean lifestyle program: a randomized clinical trial for postmenopausal women with type 2 diabetes. Int J Behav Nutr Phys Act. 2007, 82: 1.																	X																			
Trento M, Passera P, Tomalino M, Pagnozzi F, Pomero F, Vaccari P, et al: Therapeutic group education in the follow-up of patients with non-insulin treated, non-insulin dependent diabetes mellitus. Diabetes Nutr Metab Clin Exp. 1998, 11: 212-216.																	X															X			X	X
Trento M, Passera P, Tomalino M, Bajardi M, Pomero F, Allione A, et al: Group visits improve metabolic control in type 2 diabetes: a 2-year follow-up. Diabetes Care. 2001, 24: 995-1000. 10.2337/diacare.24.6.995.																	X	X										X				X				
Trento M, Passera P, Borgo E, Tomalino M, Bajardi M, Cavallo F, et al: A 5-year randomized controlled study of learning, problem solving ability, and quality of life modifications in people with type 2 diabetes managed by group care. Diabetes Care. 2004, 112: 670-675.																	X	X										X								
Wattana C, Srisuphan W, Pothiban L, Upchurch SL: Effects of a diabetes self-management program on glycemic control, coronary heart disease risk, and quality of life among Thai patients with type 2 diabetes. Nurs Heal Sci. 2007, 9: 135-141.																	X					X														
Zapotoczky H, Semlitsch B, Herzog G, Bahadori B, Siebenhofer A, Pieber TR, et al: A controlled study of weight reduction in type 2 diabetics treated by two reinforcers. Int J Behav Med. 2001, 8: 42-49.																	X															X				
Deakin TA, Cade JE, Williams DDR, Greenwood DC: Empowered patients:better diabetes control, greater freedom to eat, no weight gain!. Diabetologia. 2003, 46 (suppl 2): A90.																	X											X				X				
Clancy D, Cope D, Magruder K, Peng H, Wolfman T. Evaluating Concordance to American Diabetes Association Standards of Care for Type 2 Diabetes Through Group Visits in an Uninsured or Inadequately Insured Patient Population. Diabetes Care. 2003; 26(7): 2032-2036.																		X																		
Kirsh S, Watts S, Pascuzzi K, O'Day M, Davidson D, Aron D, et al. Shared medical appointments based on the chronic care model: a quality improvement project to address the challenges of patients with diabetes with high cardiovascular risk. Qual Saf Health Care. 2007; 16(5): 349-353.																		X																		
Edelman D, Fredrickson S, Melnyk S, Coffman C, Jeffreys A, Weinberger M, et al. Medical clinics versus usual care for patients with both diabetes and hypertension: a randomized trial. Ann Intern Med. 2010; 152(11): 689-696.																		X																		
Taveira T, Friedmann P, Cohen L, Dooley A, Khatana S, Wu W, et al. Pharmacist-led group medical appointment model in type 2 diabetes. Diabetes Educ. 2010; 36(1): 109-117.																		X																		
Trento M, Basile M, Borgo E, Grassi G, Scuntero P,																		X																		

For peer review only

Porta M, et al. A randomised controlled clinical trial of nurse-, dietitian- and pedagogist-led Group Care for the management of Type 2 diabetes. J Endocrinol Invest. 2008; 31(11): 1038-1042.																																				
Pieber T, Holler A, Siebenhofer A, Brunner G, Semlitsch B, Krejs G, et al. Evaluation of a structured teaching and treatment programme for type 2 diabetes in general practice in a rural area of Austria. Diabet Med. 1995; 12(4): 349-354.																		X				X														
Trento M, Passera, P, Tomalino M, Pagnozzi F, Pomero F, Vaccari P, Bajardi M, Molinatti GM, Porta M. Theraputic group education in the follow-up of patients with non-insulin treated, non-insulin dependent diabetes mellitus. Diabetes Nutr Metab. 1998; 2(3):212-216.																		X																		
Trento M, Gamba S, Gentile L, Grassi G, Miselli V, Porta M, et al. Rethink Organization to iMprove Education and Outcomes (ROMEO): a multicenter randomized trial of lifestyle intervention by group care to manage type 2 diabetes. Diabetes Care. 2010; 33(4): 745-747.																		X																		
Clancy D, Brown S, Magruder K, Huang P. Group visits in medically and economically disadvantaged patients with type 2 diabetes and their relationships to clinical outcomes. Top Health Inf Manage. 2003; 24(1): 8-14.																		X													X					
Bray P, Thompson D, Wynn J, Cummings D, Whetstone L. Confronting disparities in diabetes care: the clinical effectiveness of redesigning care management for minority patients in rural primary care practices. J Rural Health. 2005; 21(4): 317-321.																		X																		
Culhane-Pera K, Peterson K, Crain A, Center B, Lee M, Xiong T, et al. Group visits for Hmong adults with type 2 diabetes mellitus: a pre-post analysis. J Health Care Poor Underserved. 2005; 16(2): 315-327.																		X																		
Mullan RJ, Montori VM, Shah ND, Christianson TJH, Bryant SC, Guyatt GH, et al. The diabetes mellitus medication choice decision aid: a randomized trial. Arch Intern Med. 2009;169(17):15608																			X																	
Fornos JA, Andres NF, Andres JC, Guerra MM, Egea B. A pharmacotherapy follow-up program in patients with type-2 diabetes in community pharmacies in Spain. Pharm World Sci. 2006;28(2):65-72.																			X																	
Al Mazroui NR, Kamal MM, Ghabash NM, Yacout TA, Kole PL, McElnay JC. Influence of pharmaceutical care on health outcomes in patients with Type 2 diabetes mellitus. Br J Clin Pharmacol. 2009;67(5):547-57.																			X																	
Farmer AJ, Wade AN, French DP, Simon J, Yudkin P, Gray A, et al. Blood glucose selfmonitoring in type 2 diabetes: a randomised controlled trial. Health Technol Assess. 2009;13(15):iii																			X																	
Kulzer B, Hermanns N, Reinecker H, Haak T. Effects of self-management training in Type 2 diabetes: a randomized, prospective trial. Diabet Med. 2007;24(4):415-23.																			X																	
H.-S. Kwon, J.-H. Cho, H.-S. Kim, B.-R. Song, S.-H. Ko, J.-M. Lee, et al.																				X																

For peer review only - http://bmjopen.bmj.com/site/about/guidelines.xhtml

Establishment of blood glucose monitoring system using the Internet<br>Diabetes Care, 27 (2) (2004), pp. 478–483																																				
J.-H. Cho, S.-A. Chang, H.-S. Kwon, Y.-H. Choi, S.-H. Ko, S.-D. Moon, et al.<br>Long-term effect of the Internet-based glucose monitoring system on HbA1c reduction and glucose stability<br>Diabetes Care, 29 (12) (2006), pp. 2625–2631																				X																
G.T. McMahon, H.E. Gomes, S. Hickson Hohne, T.M.-J. Hu, B.A. Levine, P.R. Conlin<br>Web-based care management in patients with poorly controlled diabetes<br>Diabetes Care, 28 (7) (2005), pp. 1624–1629																				X																
G.E. Bond, R. Burr, F.M. Wolf, M. Price, S.M. McCurry, L. Teri<br>Preliminary findings of the fffects of comorbidities on a web-based intervention on self-reported blood sugar readings among adults age 60 and older with diabetes<br>Telemed. J. e-Health, 12 (6) (2006), pp. 707–710																				X																
H.-S. Kim, H.-Y. Son<br>Technological intervention for obese patients with type 2 diabetes<br>Appl. Nurs. Res., 21 (2) (2008), pp. 84–89																				X																
S.-I. Kim, H.-S. Kim<br>Effectiveness of mobile and internet intervention in patients with obese type 2 diabetes<br>Int. J. Med. Inf., 77 (6) (2008), pp. 399–404																				X																
J.D. Ralston, I.B. Hirsch, J. Hoath, M. Mullen, A. Cheadle, H.I. Goldberg<br>Web-based collaborative care for type 2 diabetes<br>Diabetes Care, 32 (2) (2009), pp. 234–239																				X																
J.-H. Noh, Y.-J. Cho, H.-W. Nam, J.-H. Kim, D.-J. Kim, H.-S. Yoo, et al.<br>Web-based comprehensive information system for self-management of diabetes mellitus<br>Diabetes Technol. Ther., 12 (5) (2010), pp. 333–337																				X																
E.G. Fell, R.E. Glasgow, S. Boles, H.G. McKay<br>Who participates in Internet-based self-management programs? A study among novice computer users in a primary care setting<br>Diabetes Educ., 26 (5) (2000), pp. 806–811																				X																
H.G. McKay, D. King, E.G. Eakin, J.R. Seeley, R.E. Glasgow<br>The Diabetes Network Internet-based physical activity intervention: a randomized pilot study<br>Diabetes Care, 24 (8) (2001), pp. 1328–1334																				X			X													
C. Richardson, K. Mehari, L. McIntyre, A. Janney, L. Fortlage, A. Sen, et al.																				X																

For peer review only

A randomized trial comparing structured and lifestyle goals in an internet-mediated walking program for people with type 2 diabetes<br>Int. J. Behav. Nutr. Phys. Act., 4 (1) (2007), p. 59																																				
T. Liebreich, R. Plotnikoff, K. Courneya, N. Boule<br>Diabetes NetPLAY: a physical activity website and linked email counselling randomized intervention for individuals with type 2 diabetes<br>Int. J. Behav. Nutr. Phys. Act., 6 (1) (2009), p. 18																				X																
A. Holbrook, L. Thabane, K. Keshavjee, L. Dolovich, B. Bernstein, D. Chan, et al.<br>Individualized electronic decision support and reminders to improve diabetes care in the community: COMPETE II randomized trial<br>CMAJ, 181 (1–2) (2009), pp. 37–44																				X																
Keyserling TC, Samuel-Hodge CD, Ammerman AS, Ainsworth BE, Henriquez-Roldan CF, Elasy TA et al. A randomised trial of an intervention to improve self-care behaviours of African-American women with type 2 diabetes. Diabetes Care 2002; 25: 1576–1583.																					X									X						
K. Samaras, S. Ashwell, A.M. Mackintosh, A.C. Fleury, L.V. Campbell, D.J. Chisholm<br>Will older sedentary people with non-insulin-dependent diabetes mellitus start exercising? A health promotion model<br>Diabetes Res Clin Pract, 37 (1997), pp. 121–128																						X								X						
C.K. Miller, L. Edwards, G. Kissling, L. Sanville<br>Nutrition education improves metabolic outcomes among older adults with diabetes mellitus: results from a randomized controlled trial<br>Prev Med, 34 (2002), pp. 252–259																						X														
P. Gaede, M. Beck, P. Vedel, O. Pedersen<br>Limited impact of lifestyle education in patients with type 2 diabetes mellitus and microalbuminuria: results from a randomized intervention study<br>Diabetic Med, 18 (2001), pp. 104–108																						X														
C. Di Loreto, C. Fanelli, P. Lucidi, G. Murdolo, C.A. De, N. Parlanti, F. Santeusanio, P. Brunetti, P. De Feo<br>Validation of a counseling strategy to promote the adoption and the maintenance of physical activity by type 2 diabetic subjects<br>Diabetes Care, 26 (2003), pp. 404–408																						X														
R.A. Gabbay, I. Lendel, T.M. Saleem, G. Shaeffer, A.M. Adelman, D.T. Mauger, M. Collins, R.C. Polomano<br>Nurse case management improves blood pressure, emotional distress and diabetes complication screening<br>Diabetes Res Clin Pract, 71 (2006), pp. 28–35																						X														
S.H. Ko, K.H. Song, S.R. Kim, J.M. Lee, J.S. Kim, J.H. Shin<br>Long-term effects of a structured intensive diabetes education programme (SIDEP) in patients with type 2																						X						X								

For peer review only - http://bmjopen.bmj.com/site/about/guidelines.xhtml

diabetes mellitus—a 4-year follow-up study<br>Diabetic Med, 24 (2007), pp. 55–62																																				
P. Kronsbein, V. Jorgens, I. Muhlhauser, V. Scholz, A. Venhaus, M. Berger<br>Evaluation of a structured treatment and teaching programme on non-insulin-dependent diabetes<br>Lancet, 2 (1988), pp. 1407–1411																						X						X				X				
A.L. Estey, M.H. Tan, K. Mann<br>Follow-up intervention: its effect on compliance behavior to a diabetes regimen<br>Diabetes Educ, 16 (1990), pp. 291–295																						X												X		
M.J. Franz, A. Monk, B. Barry, K. McClain, T. Weaver, N. Cooper<br>Effectiveness of medical nutrition therapy provided by dieticians in the management of non-insulin-dependent diabetes mellitus: a randomized, controlled clinical trial<br>J Am Diet Assoc, 95 (1995), pp. 1009–1017																						X														
M.I. Uusitupa<br>Early lifestyle intervention in patients with non-insulin-dependent diabetes mellitus and impaired glucose tolerance<br>Ann Med, 28 (1996), pp. 445–449																						X								X						X
B.P. Krier, R.D. Parker, D. Grayson, G. Byrd<br>Effect of diabetes education on glucose control<br>J La State Med Soc, 151 (1999), pp. 86–92																						X							X				X			
H. Sone, A. Katagiri, S. Ishibashi, R. Abe, Y. Saito, T. Murase, R. Abe, Y. Ohashi, Y. Akanuma, N. Yamada, Japan Diabetes Complication Study Group<br>Effects of lifestyle modifications on patients with type 2 diabetes: the Japan Diabetes Complications Study (JDCS) study design, baseline analysis and three year-interim report<br>Horm Metab Res, 34 (2002), pp. 509–515																						X														
C.B. Taylor, N.H. Miller, K.R. Reilly, G. Greenwald, D. Cunning, A. Deeter, L. Abascal<br>Evaluation of nurse-care management system to improve outcomes in patients with complicated diabetes<br>Diabetes Care, 26 (2003), pp. 1058–1063																						X														
J.A. Oh, H.S. Kim, K.H. Yoon, E.S. Choi<br>A telephone-delivered intervention to improve glycemic control in type 2 diabetic patients<br>Yonsei Med J, 44 (2003), pp. 1–8																						X														
R. Whittemore, G.D. Melkus, A. Sullivan, M. Grey<br>A nurse-coaching intervention for women with type 2 diabetes<br>Diabetes Educ, 30 (2004), pp. 795–804																						X	X	X												
M. Maislos, D. Weisman<br>Multidisciplinary approach to patients with poorly																						X														

For peer review only

For peer review only - http://bmjopen.bmj.com/site/about/guidelines.xhtml

controlled type 2 diabetes mellitus: a prospective, randomized study<br>Acta Diabetol, 41 (2004), pp. 44–48																																				
R. Rachiman, I. Slavacheski, M. Berla, R. Frommer-Shapira, M. Ravid<br>Treatment of high-risk patients with diabetes: motivation and teaching intervention: a randomized, prospective 8-year follow-up study<br>J Am Soc Nephrol, 16 (2005), pp. S22–S26																						X														
R.J. Young, J. Taylor, T. Friede, S. Hollis, J.M. Mason, P. Lee, E. Burns, A.F. Long, T. Gambling, J.P. New, J.M. Gibson<br>Pro-active call center treatment support (PACCTS) to improve glucose control in type 2 diabetes: a randomized controlled trial<br>Diabetes Care, 28 (2005), pp. 278–282																						X														
M.S. Song, H.S. Kim<br>Effect of the diabetes outpatient intensive management programme on glycaemic control for type 2 diabetic patients<br>J Clin Nurs, 16 (2007), pp. 1367–1373																						X														
Z.W. Ma, M.X. Li, C.S. Wang<br>Effects of comprehensive psycho-intervention on life quality and carbohydrate metabolism in patients with type 2 diabetes mellitus<br>Zhongguo Linchuang Kangfu, 10 (2006), pp. 15–17																							X													
L. Zhao, Y.J. Sun, Q.G. Liu, M.M. Huan, J. Wu, L. Zhou, H.J. Shen, C. Jiang<br>Intervention effects of music relaxation therapy on the quality of life in patients with diabetic retinopathy<br>Zhongguo Linchuang Kangfu, 9 (2005), pp. 50–53																							X													
Z. Zeng, L. Ma, L. Tang, G. Luo, H. Zhou, L. Zhou<br>Comprehensive intervention on diabetes mellitus<br>Chin Ment Health J, 17 (2003), pp. 253–255																							X													
A. Kirk, N. Mutrie, P. MacIntyre, M. Fisher<br>Effects of a 12-month activity counselling intervention on glycaemic control and on the status of cardiovascular risk factors in people with type 2 diabetes<br>Diabetologia, 47 (2004), pp. 821–832																							X													
F.L. Huang, J.H. Ye, F. Huang<br>Effect of mental intervention on type 2 diabetics: randomized controlled study<br>Zhongguo Linchuang Kangfu, 8 (2004), pp. 5795–5798																							X													
R.A. McGinnis, A. McGrady, S.A. Cox, K.A. Grower-Dowling<br>Biofeedback-assisted relaxation in type 2 diabetes<br>Diabetes Care, 28 (2005), pp. 2145–2149																							X													
M. Siebolds, O. Gaedeke, U. Schwedes<br>Self-monitoring of blood glucose-psychological aspects																							X													

relevant to changes in hba1c in type 2 diabetic patients treated with diet or diet plus oral antidiabetic medication<br>Patient Educ Couns, 62 (2006), pp. 104–110																																				
R. Jackson, K. Asimakopoulou, A. Scammell<br>Assessment of the transtheoretical model as used by dietitians in promoting physical activity in people with type 2 diabetes<br>J Hum Nutr Diet, 20 (2007), pp. 27–36																							X													
P.J. Lustman, L.S. Griffith, K.E. Freedland, S.S. Kissel, R.E. Clouse<br>Cognitive behavior therapy for depression in type 2 diabetes mellitus: a randomized, controlled trial<br>Ann Intern Med, 129 (1998), pp. 613–621																							X													
C.A. Heitzmann, R.M. Kaplan, D.K. Wilson, J. Sandler<br>Sex differences in weight loss among adults with type ii diabetes mellitus<br>J Behav Med, 10 (1987), pp. 197–211																							X													X
L.V. Campbell, R. Barth, J.K. Gosper, J.J. Jupp, L.A. Simons, D.J. Chisholm<br>Impact of intensive educational approach to dietary change in NIDDM<br>Diabetes Care, 13 (1990), pp. 841–847																							X							X						X
J.D. Lane, C.C. McCaskill, S.L. Ross, M.N. Feinglos, R.S. Surwit<br>Relaxation training for NIDDM: predicting who may benefit<br>Diabetes Care, 16 (1993), pp. 1087–1094																							X											X		
E.M. Campbell, S. Redman, P.S. Moffitt, R.W. Sanson-Fisher<br>The relative effectiveness of educational and behavioral instruction programs for patients with NIDDM: a randomized trial<br>Diabetes Educator, 22 (1996), pp. 379–386																							X	X				X						X	X	X
J. Kenardy, M. Mensch, K. Bowen, B. Green, J. Walton<br>Group therapy for binge eating in type 2 diabetes: a randomized trial<br>Diabet Med, 19 (2002), pp. 234–239																							X													
S.W. Rabkin, E. Boyko, A. Wilson, D.A. Streja<br>A randomized clinical trial comparing behavior modification and individual counseling in the nutritional therapy of non-insulin-dependent diabetes mellitus: comparison of the effect on blood sugar, body weight, and serum lipids<br>Diabetes Care, 6 (1983), pp. 50–56																							X													
R.R. Wing, L.H. Epstein, M.P. Nowalk, R. Koeske, S. Hagg<br>Behavior change, weight loss, and physiological improvements in type ii diabetic patients<br>J Consult Clin Psych, 53 (1985), pp. 111–122																							X											X		X

For peer review only - http://bmjopen.bmj.com/site/about/guidelines.xhtml

S. Hartwell, R. Kaplan, J. Wallace<br>Comparison of behavioral interventions for control of type ii diabetes mellitus<br>Behav Ther, 17 (1986), pp. 447–461																							X							X						X
N. White, J. Carnahan, C.A. Nugent, T. Iwaoka, M.A. Dodson<br>Management of obese patients with diabetes mellitus: comparison of advice education with group management<br>Diabetes Care, 9 (1986), pp. 490–496																							X						X				X	X	X	X
F. Rodriguez, M. Alvarez<br>Modificacion de conducta y mejora en el cumplimiento del tratamiento en diabeticos tipo ii<br>Rev Esp Ter Comportamiento, 5 (1987), pp. 223–247																							X													
R.R. Wing, M.D. Marcus, L.H. Epstein, A. Jawad<br>A "Family-based" approach to the treatment of obese type ii diabetic patients<br>J Consult Clin Psych, 59 (1991), pp. 156–162																							X											X		
G.A. D'Eramo-Melkus, J. Wylie-Rosett, J.A. Hagan<br>Metabolic impact of education in NIDDM<br>Diabetes Care, 15 (1992), pp. 864–869																							X											X	X	X
S. Boehm, E.A. Schlenk, E. Raleigh, D. Ronis<br>Behavioral analysis and behavioral strategies to improve self-management of type ii diabetes<br>Clin Nurs Res, 2 (1993), pp. 327–344																							X											X		X
J.E. Aikens, T.A. Kiolbasa, R. Sobel<br>Psychological predictors of glycemic change with relaxation training in non-insulin-dependent diabetes mellitus<br>Psychother Psychosom, 66 (1997), pp. 302–306																							X											X		
J. Henry, P. Wilson, D. Bruce, D. Chisholm, P. Rawling<br>Cognitive-behavioural stress management for patients with non-insulin dependent diabetes mellitus<br>Psychol Health Med, 2 (1997), pp. 109–118																							X											X		
S.L. Jablon, B.D. Naliboff, S.L. Gilmore, M.J. Rosenthal<br>Effects of relaxation training on glucose tolerance and diabetic control in type ii diabetes<br>Appl Psychophys Biof, 22 (1997), pp. 155–169																							X													
X. Huang, L. Song, T. Li<br>The effect of social support on type ii diabetes with depression<br>Chin J Clin Psychol, 9 (2001), pp. 187–189																							X													
X. Zhu, Y. Gong, S. Yao<br>The effect of biofeedback assisted relaxation training on cytokines in patients with type ii diabetes<br>Chin J Clin Psychol, 9 (2001), pp. 170–172																							X													
R.S. Surwit, M.A. van Tilburg, N. Zucker, C.C. McCaskill, P. Parekh, M.N. Feinglos, C.L. Edwards, P. Williams,																							X													

For peer review only

J.D. Lane<br>Stress management improves long-term glycemic control in type 2 diabetes<br>Diabetes Care, 25 (2002), pp. 30–34																																				
T. Tsujiuchi, H. Kumano, K. Yoshiuchi, D. He, Y. Tsujiuchi, T. Kuboki, H. Suematsu, K. Hirao<br>The effect of qi-gong relaxation exercise on the control of type 2 diabetes mellitus: a randomized controlled trial<br>Diabetes Care, 25 (2002), pp. 241–242																							X													
Hiss RG, Lou Gillard M, Armbruster BA, McClure LA. Comprehensive evaluation of community-based diabetic patients: Effect of feedback to patients and their physicians: A randomized controlled trial. Diabetes Care 2001;24(4):690-4.																								X												
Dalmau Llorca MR, Garcia Bernal G, Aguilar Martin C, Palau Galindo A. [Group versus individual education for type-2 diabetes patients]. Atencion Primaria 2003;32(1):36-41.																								X												
Deakin T, Cade JE, Williams DDR, Greenwood DC. EXpert patient education versus routine treatment: process evaluation. Diabetologia 2002;45(Suppl 2): 317.																												X								
Brown SA, Hanis CL. Culturally competent diabetes education for Mexican Americans: the Starr County Study. Diabetes Educ 1999;25:226–36																												X								X
Cooper H, Booth K, Gill G. A randomised controlled study of education for people with type 2 diabetes. Liverpool: University of Liverpool; 2002.																												X								
Cooper H, Booth K, Gill G. Using combined research methods for exploring diabetes patient education. Patient Educ Couns 2003;51:45–52.																												X								
Raz I, Soskolne V, Stein P. Influence of small-group education sessions on glucose homeostasis in NIDDM. Diabetes Care 1988;11:67–71.																												X						X	X	X
Domenech MI, Assad D, Mazzei ME, Kronsbein P, Gagliardino JJ. Evaluation of the effectiveness of an ambulatory teaching/treatment programme for noninsulin dependent (type 2) diabetic patients. Acta Diabetol 1995;32:143–7.																												X			X	X				
Keyserling TC, Ammerman AS, Samuel-Hodge CD, Ingram AF, Skelly AH, et al. A diabetes management program for African American woment with type 2 diabetes. The diabetes educator 2000;26(5):796-805.																										X				X						
Vyas A, Haidery AZ, Wiles PG, Gill S, Roberts C, Cruikshank JK. A pilot randomised trial in primary care to investigate and improve knowledge, awareness and self-management among South Asians with diabetes in Manchester. Diabet Med 2003; 20: 1022–1026.																											X									
Anon (reporting study by Kerr L et al .). Tailored approach overcomes cultural barriers. Pharm J 2004; 273: 14.																											X									

For peer review only

Samanta A, Campbell JE, Spalding DL, Panja KK, Neogi SK, Burden AC. Dietary habits of Asian diabetics in a general practice clinic. Hum Nutr Appl Nutr 1987; 41A: 160–163.																											x									
Wallace P, Awan A, Talbot J. Health Advice for Asian women with diabetes. Prof Nurse 1996; 11: 794–796.																											x									
Simmons D. Diabetes self help facilitated by local diabetes research: The Coventry Asian Diabetes Support Group. Diabet Med 1992; 9: 866–869.																											x									
A. Mease, W.L. Whitlock, A. Brown, K. Moore, H. Pavliscsak, A. Dingbaum, et al. Telemedicine improved diabetic management Mil. Med., 165 (8) (2000), pp. 579–584																													x				x			
J.D. Piette, M. Weinberger, F.B. Kraemer, S.J. McPhee Impact of automated calls with nurse follow-up on diabetes treatment outcomes in a department of veterans affairs Health Care System Diab. Care, 24 (2) (2001), pp. 202–208																													x				x			
R. Rachmani, Z. Levi, I. Slavachevski, M. Avin, M. Ravid Teaching patients to monitor their risk factors retards the progression of vascular complications in high-risk patients with Type 2 diabetes mellitus. A randomized prospective study Diab. Med., 19 (2002), pp. 385–392																													x				x			
E.A. Coast-Senior, B.A. Kroner, C.L. Kelley, L.E. Trilli Management of patients with type 2 diabetes by pharmacists in primary care clinics The Ann. Pharmacother., 32 (1998), pp. 636–641																													x				x			
Y.-D. Jiang, L.-M. Chuang, H.-P. Wu, S.-J. Shiau, C.-H. Wang, Y.-J. Lee, et al. Assessment of the function and effect of diabetes education programs in Taiwan Diab. Res. Clin. Pract., 46 (1999), pp. 177–182																													x				x			
de Bont AJ, Baker IA, St Leger AS, Sweetnam PM, Wragg KG, Stephens SM, Hayes TM. A randomised controlled trial of the effect of low fat diet advice on dietary response in insulin independent diabetic women. Diabetologia 1981;21(6):529-33.																														x					x	
Gallagher A, Henderson W, Abraira C. Dietary patterns and metabolic control in diabetic diets: a prospective study of 51 outpatient men on unmeasured and exchange diets. Journal of the American College of Nutrition 1987;6(6):525-32																														x						
Gallagher AM, Abraira C, Henderson WG. A four-year prospective trial of unmeasured diet in lean diabetic adults. Diabetes Care 1984;7(6):557-65.																														x						
Glasgow RE, La Chance PA, Toobert DJ, Brown J, Hampson SE, Riddle MC. Long term effects and costs of brief behavioural dietary intervention for patients with diabetes delivered from the medical office. Patient																														x						

For peer review only

Education and Counseling 1997;32(3):175-184.																																				
Glasgow RE, Toobert DJ, Hampson SE. Effects of a Brief Office-Based Intervention to Facilitate Diabetes Dietary Self-Management. Diabetes Care 1996;19(8):835-42.																													X					X	X
Hockaday TDR, Hockaday JM, Mann JI, Turner RC. Prospective comparison of modified-fat-high-carbohydrate with standard low-carbohydrate dietary advice in the treatment of diabetes: a one year follow-up study. British Journal of Nutrition 1978;39:357-62.																													X						
Hockaday TDR, Pandher KS, Bron A, et al. Progression of established retinopathy is unrelated to glycosylated hemoglobin in non-insulin-dependent diabetes. Transplantation Proceedings 1986;18(6):1574-1575.																													X						
Howard-Williams J, Hillson RM, Bron A, Awdry P, Mann JI, Hockaday TDR. Retinopathy is associated with higher glycaemia in maturity-onset type diabetes. Diabetologia 1984;27:198-202.																													X						
Howard-Williams J, Patel P, Jelfs R, Carter RD, Awdry P, Bron A, et al. Polyunsaturated fatty acids and diabetic retinopathy. British Journal of Ophthalmology 1985;69(1):15-8.																													X						
Lopez-Espinoza I, Howard-Williams J, Mann JI, Carter RD, Hockaday TD. Fatty acid composition of platelet phospholipids in non-insulin-dependent diabetics randomized for dietary advice. British Journal of Nutrition 1984;52(1):41-7.																													X						
Mann JI, Hockaday TD, Hockaday JM, Turner RC. A prospective study of modified-fat and low-carbohydrate dietary advice in the treatment of maturity-onset diabetes [proceedings]. Proceedings of the Nutrition Society 1976;35(2):72A-73A.																													X						
Kaplan RM, Atkins CJ, Wilson DK. The cost-utility of diet and exercise interventions in non-insulin-dependent diabetes mellitus. Health Promotion 1987;2(4):331-340.																													X						
Kaplan RM, Hartwell SL, Wilson DK, Wallace JP. Effects of diet and exercise interventions on control and quality of life in non-insulin-dependent diabetes mellitus. Journal of General Internal Medicine 1987;2(4):220-8.																													X				X		X
Ligtenberg PC, Hoekstra JB, Bol E, Zonderland ML, Erkelens DW. Effects of physical training on metabolic control in elderly type 2 diabetes mellitus patients. Clinical Science 1997;93(2):127-35.																													X				X		
Milne RM, Mann JI, Chisholm AW, Williams SM. Long-term comparison of three dietary prescriptions in the treatment of NIDDM. Diabetes Care 1994;17(1):74-80.																													X						
Pascale RW, Wing RR, Butler BA, Mullen M, Bononi P. Effects of a behavioral weight loss program stressing calorie restriction versus calorie plus fat restriction in obese individuals with NIDDM or a family history of diabetes. Diabetes Care 1995;18(9):1241-8.																													X						
Tsihlias EB, Gibbs AL, McBurney MI, Wolever TMS. Comparison of high- and low-glycemic-index breakfast cereals with monounsaturated fat in the long-term																													X						

For peer review only - http://bmjopen.bmj.com/site/about/guidelines.xhtml

dietary management of type 2 diabetes. American Journal of Clinical Nutrition 2000;72(2):439-49																																				
Wolever TM, Schrade KB, Vogt JA, Tsihlias EB, McBurney MI. Do colonic short-chain fatty acids contribute to the long-term adaptation of blood lipids in subjects with type 2 diabetes consuming a high-fiber diet?. American Journal of Clinical Nutrition 2002;75(6):1023-30.																														X						
Laitinen J, Uusitupa M, Ahola I, Laakso M, Siitonen O. Metabolic and dietary variables associated with glycaemic control in patients with recently diagnosed Type II diabetes mellitus. Diabetes, Nutrition & Metabolism - Clinical & Experimental 1994;7(2):77-87.																														X						
Laitinen J, Uusitupa M, Ahola I, Siitonen O. Metabolic and dietary determinants of serum lipids in obese patients with recently diagnosed non-insulin-dependent diabetes. Annals of Medicine 1994;26(2):119-24.																														X						
Vanninen E, Laitinen J, Uusitupa M. Physical activity and fibrinogen concentration in newly diagnosed NIDDM. Diabetes Care 1994;17(9):1031-8.																														X						
Vanninen E, Uusitupa M, Lansimies E, Siitonen O, Laitinen J. Effect of metabolic control on autonomic function in obese patients with newly diagnosed type 2 diabetes. Diabetic Medicine 1993;10(1):66-73.																														X						
Vanninen E, Uusitupa M, Siitonen O, Laitinen J, Lansimies E. Habitual physical activity, aerobic capacity and metaboic control in patients with newly-diagnosed Type 2 (non-insulin-dependent) diabetes mellitus: Effect of 1-year diet and exercise intervention. Diabetologia 1992;35(4):340-346.																														X				X		
Wing RR, Epstein LH, Paternostro-Bayles M, Kriska A, Nowalk MP, Gooding W. Exercise in a behavioural weight control programme for obese patients with Type 2 (non-insulin-dependent) diabetes. Diabetologia 1988;31(12):902-9.																														X						
Marcus MD, Wing RR, Guare J, Blair EH, Jawad A. Lifetime prevalence of major depression and its effect on treatment outcome in obese type II diabetic patients. Diabetes Care 1992;15(2):253-5.																														X						
Wing RR, Marcus MD, Salata R, Epstein LH, Miaskiewicz S, Blair EH. Effects of a very-low-calorie diet on long-term glycemic control in obese type 2 diabetic subjects. see comments. Archives of Internal Medicine 1991;151(7):1334-40.																														X						
Wing RR, Blair E, Marcus M, Epstein LH, Harvey J. Year-long weight loss treatment for obese patients with type II diabetes: does including an intermittent very-low-calorie diet improve outcome?. American Journal of Medicine 1994;97(4):354-62.																														X						
Kronsbein P, Miihlhauser I,Venhaus A,Jorgens V, ScholzV. Berger M (1988).Evaluation of a structured treatment and teaching programme on noninsulin-dependent diabetes. Lancet 2: 1407-11.																															X					
Pieber TR, Brunner GA, Schnedl WJ, Schattenberg S,																																X				

For peer review only - http://bmjopen.bmj.com/site/about/guidelines.xhtml

For peer review only

Kaufmenn P, Krejs GJ. Evaluation of a structured outpatient group education program for intensive insulin therapy. Diabetes Care 1995;18:625-30.																																				
Canga N, De Irala J, Vara E, Duaso MJ, Ferrer A, Martinez-Gonzalez MA. Intervention study for smoking cessation in diabetic patients. Diabetes Care 2000;23(10):1455-60.																																	X			
Hopper SV, Miller JP, Birge C, Swift J. A randomized study of the impact of home health aides on diabetic control and utilization patterns. American Journal Public Health 1984;74(6):600-2.																																	X	X		
Matsuyama JR, Mason BJ, Jue SG. Pharmacists' interventions using an electronic medication-event monitoring device's adherence data versus pill counts. The Annals of Pharmacotherpay 1993;27:851-5.																																	X			
Pullar T, Birtwell AJ, Wiles PG, Hay A, Feely MP. Use of a pharmacologic indicator to compare compliance with tablets prescribed to be taken once, twice, or three times daily. Clinical pharmacology and therapeutics 1988;44:540-5																																	X			
Rosenkranz S. Polaroid-Fundusfotografie erhöht die Patientencompliance beim Screening auf diabetische Retinopathie. Diabetes und Stoffwechsel 1006;5:69-75																																	X			
Simmons D, Upjohn M, Gamble GD. Can medication packaging improve glycemic contol and blood pressure in type 2 diabetes?. Diabetes Care 2000;23(2):153-6																																	X			
Skaer TL, Sclar DA, Markowski DJ, Won JKH. Effect of value-added utilities on prescription refill compliance and Medicaid health care expenditures - a study of patients with non-insulin-dependent diabe. 1993;18:295-9.																																	X			
Smith DM, Norton JA, Weinberger M, McDonald CJ, Katz BP. Increasing prescribed office visits. A controlled trial in patients with diabetes mellitus. Medical Care 1986;24(3):189-99.																																	X			
Diehl AK, Sugarek NJ, Bauer RL. Medication compliance in non-insulin-dependent diabetes: a randomized comparison of chlorpropamide and insulin. Diabetes Care 1985;8(3):219-23																																	X			
Davidson MB, Karlan VJ, Hair TL. Effect of a pharmacist-managed diabetes care program in a free medical clinic. American Journal of Medical Quality 2000;15(4):137-42.																																	X			
Bradshaw C, McColl E, Eccles M, Bryce C, Sampson R. Can we improve the education for type 2 diabetes patients in general practice?. Practical Diabetes International 1999;16(8):241-5																																	X			
Clarke J, Crawford A, Nash DB. Evaluation of a comprehensive diabetes disease management program: progress in the struggle for sustained behavior change. Disease management 2002;5(2):77-86.																																	X			
Paes AHP, Bakker A, Soe-Agnie CJ. Impact of dosage frequency on patient compliance. Diabetes Care 1997;20(10):1512-7.																																	X			
Allen BT, DeLong ER, Feussner JR. Impact of glucose																																		X		

For peer review only - http://bmjopen.bmj.com/site/about/guidelines.xhtml

self-monitoring on non-insulin-treated patients with type 11 diabetes mellitus. Randomized controlled trial comparing blood and urine testing. Diabetes Care. 1990;13:1044-1050.																																				
Arseneau DL, Mason AC, Wood OB, Schwab E, Green D. A comparison of learning activity packages and classroom instruction for diet management of patients with non-insulin-dependent diabetes mellitus. Diabetes Educ. 1994;20:509-514.																																	X		X
Aubert RE, Herman WH, Waters J, et al. Nurse case management to improve glycemic control in diabetic patients in a health maintenance organization: a randomized controlled trial. Ann Intern Med. 1998;129:605-612																																	X		
Bloomgarden ZT, Karmally W, Metzger MJ, et al. Randomized, controlled trial of diabetic patient education: improved knowledge without improved metabolic status. Diabetes Care. 1987;10:263-272.																																	X	X	X
Campbell LV, Barth R, Gosper JK, Jupp JJ, Simons LA, Chisholm DJ. Impact of an intensive educational approach to dietary change in NIDDM. Diabetes Care. 1990;13:841-847.																																	X		
Falkenberg MG, Elwing BE, Goransson AM, Hellstrand BE, Riis UM. Problem oriented participatory education in the guidance of adults with non-insulintreated type 11 diabetes mellitus. Scand J Primary Health Care. 1986;4:157-164.																																	X	X	
Franz MJ, Monk A, Barry B, et al. Effectiveness of medical nutrition therapy provided by dietitians in the management of non-insulindependent diabetes mellitus: a randomized, controlled clinical trial. J Am Diet Assoc. 1995;95:1009-1017.																																	X	X	X
Genev NM, McGill M, Hoskins PL, et al. Continuing diabetes education by telephone. Diabetic Med. 1990;7:920-921.																																	X		
Glasgow RE, Toobert DJ, Mitchell DL, Donnelly JE, Calder D. Nutrition education and social learning interventions for type 11 diabetes. Diabetes Care. 1989;12:150-152.																																	X		X
Glasgow RE, Toobert DJ, Hampson SE, Brown JE, Lewinsohn PM, Donnelly J. Improving self-care among older patients with type 11 diabetes: the "Sixty Something..." Study. Patient Educ Counsel. 1992;19:61-74.																																	X	X	X
Greenfield S, Kaplan SH, Ware JE Jr, Yano EM, Frank HJ. Patients' participation in medical care: effects on blood sugar control and quality of life in diabetes. J Gen Intern Med. 1988;3:448-457.																																	X		
Hassell J, Medved E. Group/audiovisual instruction for patients with diabetes. Learning achievements and time economics. J Am Diet Assoc. 1975;66:465-470.																																	X		
Hayes TM, Harries J. Randomised controlled trial of routine hospital clinic care versus routine general practice care for type 11 diabetics. Br Med J (Clin Res																																	X		

ed.). 1984;289:728-730																																				
Hurwitz B, Goodman C, Yudkin J. Prompting the clinical care of non-insulin dependent (type 11) diabetic patients in an inner city area: one model of community care. Br Med J. 1993;306:624-630.																																		X		
Jones PM. Use of a course on self-control behavior techniques to increase adherence to prescribed frequency for self-monitoring of blood glucose. Diabetes Educ. 1990;16:296-303.																																		X		X
Koproski J, Pretto Z, Poretsky L. Effects of an intervention by a diabetes team in hospitalized patients with diabetes. Diabetes Care. 1997;20:1553-1555.																																		X		
Kumana CR, Ma JT, Kung A, Kou M, Lauder 1. An assessment of drug information sheets for diabetic patients: only active involvement by patients is helpful. Diabetes Res Clin Pract. 1988;5:225-231.																																		X		X
Miller CK, Jensen GL, Achterberg CL. Evaluation of a food label nutrition intervention for women with type 2 diabetes mellitus. J Am Diet Assoc. 1999;99:323-328																																		X		
Morgan BS, Littell DH. A closer look at teaching and contingency contracting with type 11 diabetes. Patient Educ Counsel. 1988;12:145-158.																																		X		
Morrish NJ, Cohen DL, Hicks B, Keen H. A controlled study of the effect of computer-aided analysis of home blood glucose monitoring on blood glucose control. Diabetic Med. 1989;6:591-594																																		X		
Mulrow C, Bailey S, Sonksen PH, Slavin B. Evaluation of an audiovisual diabetes education program: negative results of a randomized trial of patients with non-insulin-dependent diabetes mellitus. J Gen Intern Med. 1987;2:215-219.																																		X	X	X
Rabkin SW, Boyko E, Wilson A, Streja DA. A randomized clinical trial comparing behavior modification and individual counseling in the nutritional therapy of non-insulin-dependent diabetes mellitus: comparison of the effect on blood sugar, body weight, and serum lipids. Diabetes Care. 1983;6:50-56.																																		X		X
Rettig BA, Shrauger DG, Recker RR, Gallagher TF, Wiltse H. A randomized study of the effects of a home diabetes education program. Diabetes Care. 1986;9:173-178.																																		X		X
Rost KM, Flavin KS, Cole K, McGill JB. Change in metabolic control and functional status after hospitalization. Impact of patient activation intervention in diabetic patients. Diabetes Care. 1991;14:881-889.																																		X		
Sadur CN, Moline N, Costa M, et al. Diabetes management in a health maintenance organization. Efficacy of care management using cluster visits. Diabetes Care. 1999;22:2011-2017																																		X		
Sawicki PT, Didjurgeit U, Muhlhauser 1, Berger M. Behaviour therapy versus doctor's anti-smoking advice in diabetic patients. J Intern Med. 1993;234:407-409																																		X		
Scott RS, Beaven DW, Stafford JM. The effectiveness of diabetes education for non-insulin-dependent diabetic																																		X	X	X

For peer review only - http://bmjopen.bmj.com/site/about/guidelines.xhtml

persons. Diabetes Educ. 1984:36-39.																																				
Smith DM, Weinberger M, Katz BP A controlled trial to increase office visits and reduce hospitalizations of diabetic patients. J Gen Intern Med. 1987;2:232-238.																																		X		
Tu K, McDaniel G, Gay JT. Diabetes self-care knowledge, behaviors, and metabolic control of older adults-the effect of a posteducational follow-up program. Diabetes Educ. 1993; 19:25-30																																		X	X	X
Vinicor F, Cohen SJ, Mazzuca SA, et al. DIA-BEDS: a randomized trial of the effects of physician and/or patient education on diabetes patient outcomes. J Chron Dis. 1987;40:345-356.																																		X		X
Ward WK, Haas LB, Beard JC. A randomized, controlled comparison of instruction by a diabetes educator versus self-instruction in self-monitoring of blood glucose. Diabetes Care. 1985;8:284-286.																																		X		X
Weinberger M, Kirkman MS, Samsa GE et al. A nurse-coordinated intervention for primary care patients with non-insulindependent diabetes mellitus. J Gen Intern Med. 1995;10:59-66																																		X		
Wheeler LA, Wheeler ML, Ours P, Swider C. Evaluation of computer-based diet education in persons with diabetes mellitus and limited educational background. Diabetes Care. 1985;8:537-544.																																		X		X
Wing RR, Epstein LH, Nowalk MP, Scott N, Koeske R, Hagg S. Does self-monitoring of blood glucose levels improve dietary compliance for obese patients with type 11 diabetes? Am J Med. 1986;81:830-836																																		X	X	X
Wing RR, Epstein LH, Paternostro-Bayles M, Kriska A, Nowalk MP, Gooding W. Exericise in a behavioural weight control programme for obese patients with type 2 (non-insulin-dependent) diabetes. Diabetologia. 1988;31:902-909.																																		X		
Wood ER. Evaluation of a hospital-based education program for patients with diabetes. J Am Diet Assoc. 1989;89:354-358																																		X		X
de Weerdt I, Visser A, Kok G, de Weerdt O, van der Veen E: Randomized controlled multicentre evaluation of an education programme for insulin-treated diabetic patients: effects on metabolic control, quality of life, and costs of therapy. Diabet Med 8:338–345, 1991																																			X	X
Estey A, Tan M, Mann K: Follow-up intervention: its effect on compliance behavior to diabetes regimen. Diabetes Educ 16:291–295, 1990																																			X	X
Kaplan R, Wilson D, Hartwell S, Merino K, Wallace J: Prospective evaluation of HDL cholesterol changes after diet and physical conditioning programs for patients with type II diabetes mellitus. Diabetes Care 8:343–348,																																			X	X
Korhonen T, Uusitupa M, Aro A, Kumpulainen T, Siitonen O, Voutilainen E, Pyorala K: Efficacy of dietary instructions in newly diagnosed non-insulin-dependent diabetic patients. Acta Med Scand 222:323–331, 1987																																			X	X

For peer review only

Mazzuca SA, Moorman NH, Wheeler ML, Norton JA, Fineberg NS, Vinicor F, Cohen SJ, Clark CM Jr: The diabetes education study: a controlled trial of the effects of diabetes patient education. Diabetes Care 9:1–10,																																			X	X
McCulloch D, Mitchell R, Ambler J, Tattersall R: Influence of imaginative teaching of diet on compliance and metabolic control in insulin dependent diabetes. Br Med J 287:1858–1861, 1983																																			X	
Perry T, Mann J, Lewis-Barned N, Duncan A, Waldron M, Thompson C: Lifestyle intervention in people with insulin-dependent diabetes mellitus (IDDM). Eur J Clin Nutr 51:757–763, 1997																																			X	X
Turnin M-C, Beddok R, Clottes J, Martini P, Abadie R, Buisson J-C, Soule-Cupuy C, Bonneu M, Camare R, Anton J-P, Chrisment C, Farreny H, Bayard F, Tauber J-P: Telematic expert system Diabeto: new tool for diet self-monitoring for diabetic patients. Diabetes Care 15:204–212, 1992																																			X	X
Wing R, Epstein L, Nowalk M, Scott N: Self-regulation in the treatment of type II diabetes. Behav Ther 19:11–23, 1988																																			X	X
Fernando D: Knowledge about diabetes and metabolic control in diabetic patients. Ceylon Med J 38:18-21, 1993																																				X
Falkenberg M, Elwing B, Goransson A, Hellstrand B, Riis U: Problem-oriented participatory education in the guidance of adults with non-insulin-treated type-II diabetes mellitus. Scand J Prim Health Care 4:157-164, 1986																																				X
McCulloch D, Mitchell R, Ambler J, Tattersall R: Influence of imaginative teaching of diet on compliance and metabolic control in insulin-dependent diabetes. BMJ 287:1858-1861, 1983																																				X
Korhonen T, Huttunen J, Aro A, Hentinen M, Ihalainen O, Majander H, Siitonen O, Uusitupa M, Pyorala K: A controlled trial on the effects of patient education in the treatment of insulin-dependent diabetes. Diabetes Care 6:256- 261, 1983																																				X
Kendall P. Jansen G: Educating patients with diabetes: comparison of nutrient-based and exchange group methods. J Am Diet Assoc 90:238-243, 1990																																				X
de Weerdt I, Visser A, Kok G, van der Veen E: Randomized controlled evaluation of an education programme for insulin treated patients with diabetes: effects on psychosocial variables. Patient Educ Counsel 14:191-215, 1989																																				X
Brown S, Duchin S, Villagomez E: Diabetes education in a Mexican-American population: pilot testing of a research-based videotape. Diabetes Educator 18:47-51, 1992																																				X
Kim J-Y, Phillips T: The effectiveness of two forms of																																				X

For peer review only

corrective feedback in diabetes education. J Comput Based Instruct 18:14-18, 1991																																				
Colagiuri R, Colagiuri S, Naidu V: Can patients set their own educational priorities? Diabetes Res Clin Pract 30:131-136, 1995																																				X
Small R, Hill Hopper J: Evaluation of videotape teaching of self-monitoring of blood glucose by elderly diabetic patients. Consultant Pharmacist 7:24-27, 1992																																				X
Van Veldhuizen-Scott M, Widmer L, Stacey S, Popovich N: Developing and implementing a pharmaceutical care model in an ambulatory care setting for patients with diabetes. Diabetes Educator 21:117-123, 1995																																				X
Genev N, McGill M, Hoskins P, Constantino M, Plehwe W, Yue D, Turtle J: Continuing diabetes education by telephone. Diabet Med 7:920-921, 1990																																				X
Litzelman D, Slemenda C, Langefeld C, Hays L, Welch M, Bild D, Ford E, Vinicor F: Reduction of lower extremity clinical abnormalities in patients with noninsulin-dependent diabetes mellitus: a randomized, controlled trial. Ann Intern Med 119:36-41, 1993																																				X
Kruger S, Guthrie D: Foot-care: knowledge retention and self-care practices. Diabetes Educator 18:487-490, 1992																																				X
Hanefeld M, Fischer S, Schmechel H, Rothe G, Schulze J, Dude H, Schwanebeck U, Julius U: Diabetes intervention study: multi-intervention trial in newly diagnosed NIDDM. Diabetes Care 14:308-317, 1991																																				X
de Bont A, Baker I, St. Leger A, Sweetnam P, Wragg K, Stephens S, Hayes T: A randomised controlled trial of the effect of low-fat diet advice on dietary response in insulin-independent diabetic women. Diabetologia 21:529-533, 1981																																				X
Glasgow R, La Chance P-A, Toobert D, Brown J, Hampson S, Riddle M: Long term effects and costs of brief behavioral dietary intervention for patients with diabetes delivered from the medical office. Patient Educ Couns 32:175-184, 1997																																				X
Mengham L, Morris B, Palmer C, White A: Is intensive dietetic intervention effective for overweight patients with diabetes mellitus? A randomised controlled study in a general practice. Practical Diabetes International 16:5-8, 1999																																				X
Frost G, Wilding J, Beecham J: Dietary advice based on the glycaemic index improves dietary profile and metabolic control in type 2 diabetic patients. Diabet Med 11:397-401, 1994																																				X
Pratt C, Wilson W, Leklem J, Kingsley L: Peer support and nutrition education for older adults with diabetes. J Nutr Elderly 6:31-43, 1987																																				X
Hitchcock Noel P, Larme A, Meyer J, Marsh G, Correa A, Pugh J: Patient choice in diabetes education curriculum: nutritional versus standard content for type 2 diabetes. Diabetes Care 21: 896-901, 1998																																				X
Werdier D, Jesdinsky H, Helmich P: A randomized, controlled study on the effect of diabetes counseling in																																				X

For peer review only

the offices of 12 general practitioners. Rev Epidemiol Sante Publique 32:225-229, 1984																																				
Gilden J, Hendryx M, Clar S, Casia C, Singh S: Diabetes support groups improve health care of older diabetic patients. J Am Geriatr Soc 40:147-150, 1992																																				X
Laitinen J, Ahola I, Sarkkinen E, Winberg R, Harmaakorph-Livonen P, Uusitupa M: Impact of intensified dietary therapy on energy and nutrient intakes and fatty acid composition of serum lipids in patients with recently diagnosed non-insulin-dependent mellitus. J Am Diet Assoc 93:276-283, 1993																																				X
Rainwater N, Ayllon T, Frederiksen L, Moore E, Bonar J: Teaching self-management skills to increase diet compliance in diabetics. In Adherence, Compliance, and Generalization in Behavioral Medicine. Stuart R, Ed. New York, Brummer/Mazel, 1982, p. 304 -328																																				X
Wilson W, Pratt C: The impact of diabetes education and peer support upon weight and glycemic control of elderly persons with non-insulin dependent diabetes mellitus (NIDDM). Am J Public Health 77:634-635, 1987																																				X
Mazzuca K, Farris N, Mendenball J, Stoupa R: Demonstrating the added value of community health nursing for clients with insulin-dependent diabetes. J Community Health Nurs 14:211-224, 1997																																				X
Franz M, Splett F, Monk A, Barry B, McClam K, Weaker T, Upham P, Bergenstal R, Mazze R: Cost-effectiveness of medical nutrition therapy provided by dietitians for persons with non-insulin-dependent diabetes mellitus. J Am Diet Assoc 95:1018-1024, 1995																																				X
Vanninen E, Uusitupa M, Siitonen O, Laitinen J, Lansimies E: Habitual physical activity, aerobic capacity and metabolic control in patients with newly-diagnosed type 2 (non-insulin-dependent) diabetes mellitus: effect of 1-year diet and exercise intervention. Diabetologia 35:340-346, 1992																																				X
Wing R, Nowalk M, Epstein L, Koeske R: Calorie-counting compared to exchange system diets in the treatment of over-weight patients with type II diabetes. Addict Behav 11:163-168, 1986																																				X
Basch C, Walker E, Howard C, Shamoon H, Zybert P: The effect of health education on the rate of opthalmic examinations among African Americans with diabetes mellitus. Am J Public Health 89: 1878-1882, 1999																																				X
Julius U, Gross P, Hanefeld M, DIS-Group: Work absenteeism in type 2 diabetes mellitus: results of the prospective diabetes intervention study. Diabete Metab 19:202-206, 1993																																				X
Lockington T, Farrant S, Meadown K, Dowlatshahi D, Wise P: Knowledge profile and control in diabetic patients. Diabet Med 5:381-386, 1988																																				X
	53	28	17	16	132	13	33	17	20	39	10	13	16	13	8	12	26	15	7	20	10	47	35	9	NP	11	11	20	8	36	3	14	21	62	31	84

1= Choi, Davidson, Walker, Lee, & Palermo, 2016; 2=Creamer, Attridge, Ramsden, Cannings-John, & Hawthorne, 2016; 3=Huang et al., 2016; 4=Chen et al., 2015; 5=Pillay et al., 2015; 6= Terranova,

For peer review only - http://bmjopen.bmj.com/site/about/guidelines.xhtml

Brakenridge, Lawler, Eakin, & Reeves, 2015; 7= Attridge, Creamer, Ramsden, Cannings-John, & Hawthorne, 2014; 8= Odnoletkova et al., 2014; 9 = Pal et al., 2014; 10 = Ricci-Cabello et al., 2014; 11= Saffari, Ghanizadeh, & Koenig, 2014; 12=Gucciardi, Chan, Manuel, & Sidani, 2013; 13=Pal et al., 2013; 14=Vugt, Wit, Cleijne, & Snoek, 2013; 15= Amaeshi, 2012 ; 16= Nam, Janson, Stotts, Chesla, & Kroon, 2012 ; 17= Steinsbekk, Rygg, Lisulo, Rise, & Fretheim, 2012; 18= Burke et al., 2011; 19= Kai Lun Gan, Brammer, & Creedy, 2011; 20=Ramadas, Quek, Chan, & Oldenburg, 2011; 21=Hawthorne, Robles, Cannings-John, & Edwards, 2010; 22= Minet, Moller, Vach, Wagner, & Henriksen, 2010; 23=Alam, Sturt, Lall, & Winkley, 2009; 24= Duke, Colagiuri, & Colagiuri, 2009; 25= Fan & Sidani, 2009; 26=Hawthorne, Robles, Cannings-John, & Edwards, 2008 ; 27=Khunti, Camosso-Stefinovic, Carey, Davies, & Stone, 2008; 28=Loveman, Frampton, & Clegg, 2008 ; 29=Wens et al., 2008; 30 = Nield et al., 2007; 31=Zabaleta & Forbes, 2007; 32=Deakin, McShane, Cade, & Williams, 2005; 33=Vermeire et al., 2005; 34=Gary, Genkinger, Guallar, Peyrot, & Brancati, 2003; 35=Norris, Lau, Smith, Schmid, & Engelgau, 2002; 36=Norris, Engelgau, & Narayan, 2001. T2DM = type 2 diabetes mellitus; NP = not applicable.

% overlap 177/532= 33.3%

CA= Covered Area =

(53+28+17+16+132+13+33+17+20+39+10+13+16+13+8+12+26+15+7+20+10+47+35+9+11+11+20+8+36+3+14+21+62+31+84)/(35x532) = 910/18620=4.8% (one review did not report the included studies list)

CCA= Corrected Covered Area = (910-532)/ (18620-532) = 378/18088= 2.1% (slight)

For peer review only
